# Forging 1,1′-Bicyclopropenyls by Synergistic Au/Ag Dual-Catalyzed Cyclopropenyl Cross-Coupling

**DOI:** 10.1021/jacs.4c10996

**Published:** 2024-10-18

**Authors:** Xiangdong Li, Jérôme Waser

**Affiliations:** Laboratory of Catalysis and Organic Synthesis, Institute of Chemical Sciences and Engineering, Ecole Polytechnique Fédérale de Lausanne, Lausanne CH-1015, Switzerland

## Abstract

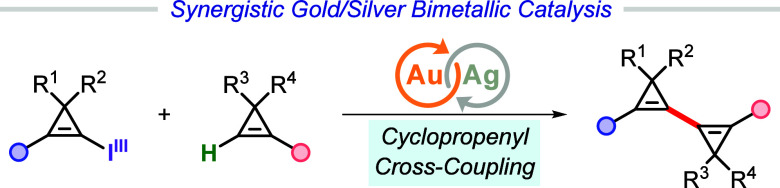

1,1′-Bicyclopropenyl is a constitutional isomer of benzene comprising two coupled cyclopropene units with the endocyclic double bonds in conjugation. Due to the intrinsic high strain energy, it remains a long-standing challenge to prepare 1,1′-bicyclopropenyl derivatives, particularly multisubstituted, nonsymmetrical ones, in an efficient and modular manner. Herein a straightforward Au/Ag bimetallic-catalyzed cyclopropenyl cross-coupling has been developed, providing a robust and versatile strategy for the rapid assembly of symmetrical and unsymmetrical 1,1′-bicyclopropenyl derivatives from cyclopropenyl benziodoxoles (CpBXs) and terminal cyclopropenes. Advantages of this strategy include tolerance to a wide range of synthetically useful functional groups, mild reaction conditions, and a simple catalytic system. The obtained 1,1′-bicyclopropenyl derivatives were shown to be valuable synthetic intermediates through selective downstream manipulations.

## Introduction

**Scheme 1:**
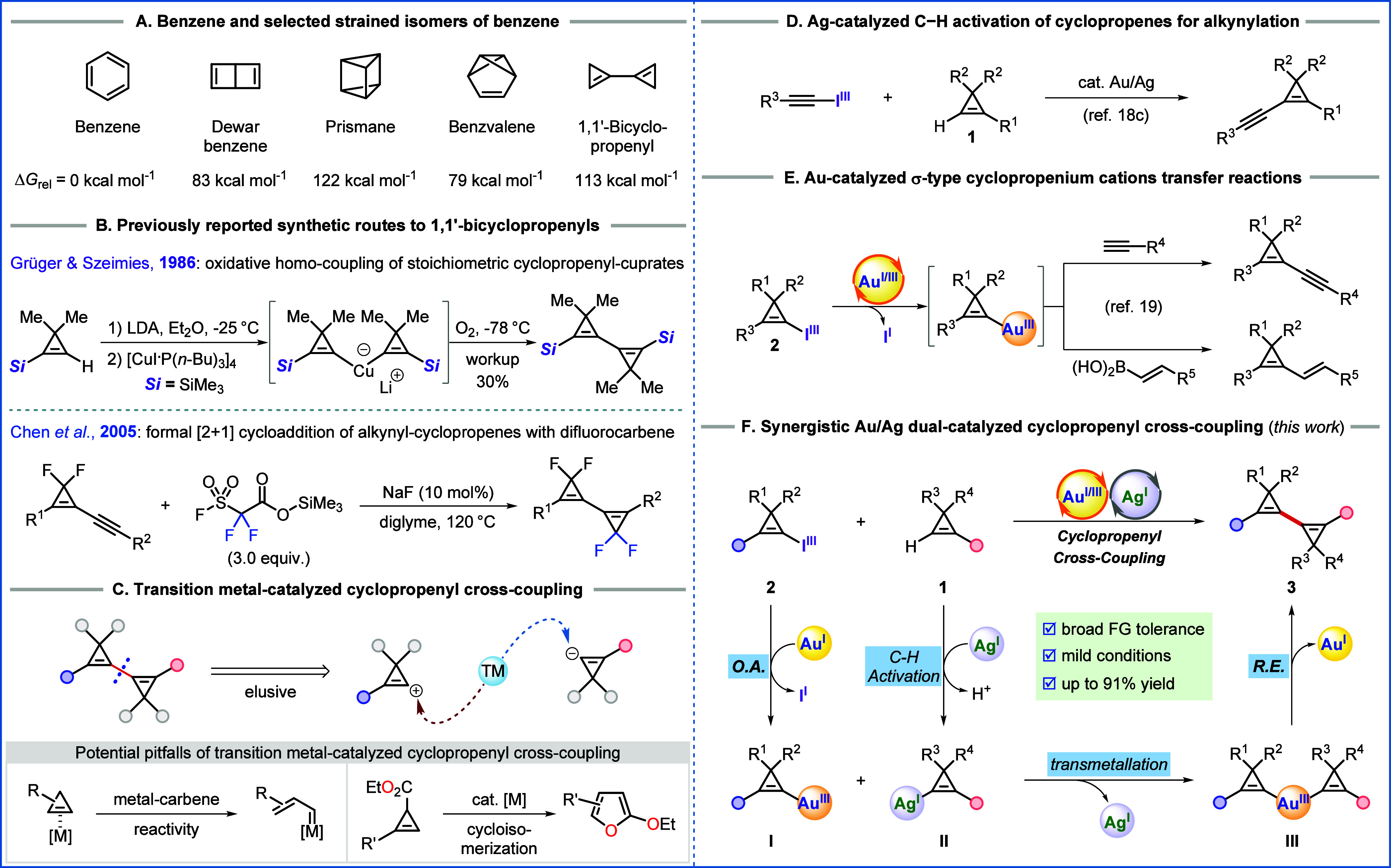
State of the Art in the Synthesis of 1,1′-Bicyclopropenyls and Our Work of Cyclopropenyl Cross-Coupling (A) Structure of benzene and its selected strained isomers. (B) Reported synthetic pathways to 1,1′-bicyclopropenyl derivatives. (C) Challenges in synthesizing 1,1′-bicyclopropenyls by transition metal-catalyzed cyclopropenyl cross-coupling. (D) Hashmi’s work: Gold-catalyzed direct alkynylation of cyclopropenes by Au/Ag dual-catalysis. (E) Our previous work: Gold-catalyzed σ-type cyclopropenium cations transfer reactions. (F) This work: Cyclopropenyl cross-coupling by synergistic Au/Ag dual-catalysis.

Since Faraday’s discovery of the first compound with the formula C_6_H_6_ in 1825,^1^ benzene and its isomers have been the subject of intensive theoretical and synthetic studies (Scheme 1A).^2^ For example, the investigation of the strained structures in Dewar benzene and prismane have been pivotal to our understanding of resonance theory and aromaticity.^3^ Even 200 years after the first isolation of benzene, the research on strained isomers of benzene and their derivatives continues to transform modern organic chemistry.^4^

Bicyclopropenyls are highly strained benzene isomers which have remained significantly underexplored.^4f,5^ Among the possible isomers, 1,1′-bicyclopropenyls were synthesized for the first time in 1986.^6^ This bicyclic isomer of benzene consists of two connected cyclopropenyl rings with the two C=C bonds in conjugation. Considering the significant strain energy (54.6 kcal/mol) of cyclopropene,^7^ the two connected cyclopropenyl rings, coupled with the lack of aromatic stabilization present in benzene, results in substantially higher free energy (+113 kcal/mol (Δ*G*_rel_) higher than benzene as calculated by density functional theory (DFT)).^8^ This high ring strain can be expected to be exploitable in follow-up transformations. However, 1,1′-bicyclopropenyls have only been rarely used as starting materials,^5a,6,9^ probably due to the lack of efficient synthetic methods to access these building blocks and their instability. The pioneering report by Grüger and Szeimies in 1986 showed that the oxidative dimerization of stoichiometric cyclopropenyl-cuprates can be employed to access symmetrical 1,1′-bicyclopropenyl derivatives in 30% yield over three steps (Scheme 1B, top).^6^ An elimination protocol was used to access the unsubstituted system,^5a^ but the compound was unstable and polymerized immediately. Alternatively, Chen et al. disclosed a formal [2 + 1] cycloaddition between alkynyl-substituted 3,3-difluoro cyclopropenes and a difluorocarbene precursor at 120 °C (Scheme 1B, bottom).^10^ These transformations all showed limited scope with moderate yields. Therefore, there is currently no general method to access multisubstituted 1,1′-bicyclopropenyl derivatives.

Transition metal–catalyzed cross-coupling plays a central role in contemporary organic synthesis.^11^ However, the high strain in cyclopropene units facilitates ring opening of cyclopropenyl-metal intermediates,^12^ making cyclopropenyl cross-coupling challenging (Scheme 1C).^13,14^ In fact, accessing 1,1’-bicyclopropenyls via a cross-coupling reaction has not been reported so far. Attempts to achieve this transformation via a well-established Pd-catalyzed Stille coupling were also not successful in our hands (see Supporting Information Section 8 for details).

Over the past decade, Au(I/III) redox catalysis has emerged as an alternative for cross-couplings, especially for those difficult to be achieved under classical Pd(0/II) catalysis.^15^ In addition, synergistic catalysis, wherein two catalysts in two catalytic cycles work in concert, has emerged as a powerful approach to reaction engineering.^16^ Synergistic bimetallic catalysis involving a gold catalyst has recently attracted increasing attention due to the advantages of enabling unconventional bond disconnections that are impossible or inefficient using monometallic gold-catalyzed conditions.^17^ Silver salts are commonly used as halogen scavengers in homogeneous gold(I) catalysis, but recent studies showed that they can play an active role in gold-catalyzed cross-coupling reactions.^18^ Elegant studies in 2019 by Hashmi and co-workers established that terminal cyclopropene substrates **1** engaged with silver(I) salts to enable direct alkynylation with ethynylbenziodoxoles via redox Au(I/III) catalysis processes (Scheme 1D).^18c^ The role of silver(I) salts, namely catalytic C–H activation of terminal cyclopropenes, was convincingly demonstrated in this work by rigorous experimental investigations. This breakthrough established Au/Ag synergistic catalysis as a promising approach for cross-coupling of cyclopropenes using easily accessible terminal cyclopropenes **1** as nucleophilic cyclopropenyl synthons.

Recently, we disclosed that the oxidative addition of newly developed hypervalent iodine reagents–the Cyclopropenyl BenziodoXoles (CpBXs)–to Au(I) occurs under mild conditions,^19^ thus giving access to a transient electrophilic cyclopropenyl-Au(III) species, which could be used in cross-coupling reactions with nucleophilic alkynes and vinyl boronic acids (Scheme 1E). In view of the appealing attributes of (i) gold-catalyzed cyclopropenyl-based cross-coupling using CpBXs and (ii) silver-catalyzed C–H activation of terminal cyclopropenes, we then decided to merge the two technologies to enable the synthesis of 1,1′-bicyclopropenyl derivatives (Scheme 1F). More specifically, it was envisioned that an initial oxidative addition (O.A.) of CpBXs **2** to the gold(I) catalyst with the concomitant transfer of the cyclopropenyl moiety from iodine to gold would generate the Au(III)-cyclopropenyl species **I**. Concurrently, the C–H bond activation of terminal cyclopropenes **1** by the silver catalyst would afford the cyclopropenylsilver species **II** in situ. Subsequently, transmetalation of **II** to **I** would lead to the dicyclopropenylgold(III) species **III**, which upon reductive elimination (R.E.) would deliver the desired cross-coupled product **3** and regenerate the gold catalyst. Considering the potential ring-opening rearrangement of cyclopropenes under gold-catalysis,^20^ we hypothesized that mild conditions would be critical to the success of the reaction. Herein, we describe our successful development of the first cyclopropenyl cross-coupling reaction via synergistic gold/silver bimetallic catalysis. This strategy enables a modular assembly of multisubstituted 1,1′-bicyclopropenyls with diverse functional groups in a straightforward manner under mild conditions. This efficient synthesis enabled us to study more in depth the synthetic potential of 1,1′-bicyclopropenyl derivatives and discover a direct conversion to bifurans, enhancing the synthetic utility of this neglected benzene isomer.

## Results and Discussion

### Reaction Development

Our investigation into the synergistic Au/Ag dual-catalyzed cyclopropenyl cross-coupling began using readily available dimethyl 2-phenylcycloprop-2-ene-1,1-dicarboxylate (**1a**) and CpBX **2a** (Table 1). After extensive exploration of various reaction parameters (see the Supporting Information for details), we identified that the commercial complexes (Me_2_S)AuCl and AgNTf_2_ act as an effective catalyst combination, together with ligand **L1**, to deliver the cross-coupled product **3a** in 92% NMR yield in CH_3_CN at 40 °C (entry 1). Control experiments revealed that all of the components used were essential: Without (Me_2_S)AuCl, no desired product was observed (entry 2), without AgNTf_2_ or **L1**, dramatic drops in yields were observed (entries 3–4). These results are consistent with our hypothesis that the reaction proceeds through a synergistic gold/silver bimetallic catalysis process (Scheme 1F). Surprisingly, a PPh_3_-ligated gold catalyst, previously shown to be the optimal catalyst for the direct alkynylation of terminal cyclopropenes using ethynylbenziodoxoles (EBXs),^18c^ was found to be much less effective in catalyzing the reaction (entry 5). In addition, when the ligand loading was reduced to 15 mol %, the desired product could still be obtained in 87% yield (entry 6). Further reducing the ligand loading led to lower conversion (entry 7), although most of the starting material could be recovered. When only 1.0 equiv of **2a** was used, the yield of **3a** slightly dropped to 81% (entry 8). Electron-deficient ligand **L1** was found to be superior in promoting the reaction than the more electron-rich analogue **L2**, which is consistent with previous reports showing that weaker σ-donor ligands can increase the rate of oxidative addition on gold(I) (entry 9).^21^ Other bidentate ligands with a flexible rotation axis, such as 2,2′-bipyridine (**L3**), were ineffective (entry 10). These results confirmed the superiority of phenanthroline-type ligands, presumably due to their significant backbone rigidity compared to the nonfused analogues. Moreover, a comparable outcome was observed when AgSbF_6_ was used instead of AgNTf_2_ (entry 11). However, when AgCl was used instead, the yield dropped to 61% (entry 12), presumably due to a reduced concentration of Ag^+^ species in the reaction mixture compared to the ones using more soluble silver salts. Generally, silver salts with weakly coordinating anions gave good to excellent yields of the cross-coupled products, while silver salts with strongly coordinating anions showed poor performance (see Section 3.4 in the Supporting Information for details). The screening of solvents revealed that THF was slightly less effective than CH_3_CN (entry 13), while using less coordinating solvents, such as DCM, resulted in lowered reaction efficiency (entry 14). Finally, an assessment of the effect of temperature on the cyclopropenyl cross-coupling reaction showed 40 °C to be optimal (entries 15–17).

**Table 1:**
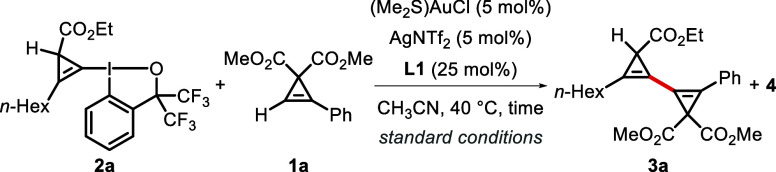

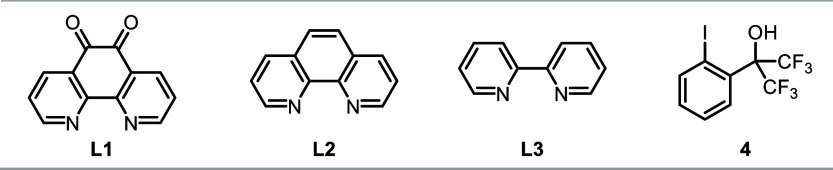
Optimization of the Synergistic Au/Ag Dual-Catalyzed Cyclopropenyl Cross-Coupling

a Standard conditions: **1a** (100 μmol), **2a** (130 μmol), (Me_2_S)AuCl (5.00 μmol), AgNTf_2_ (5.00 μmol), **L1** (25.0 μmol), CH_3_CN (2.0 mL), 40 °C.

b Determined by ^1^H NMR using dibromomethane as the internal standard.

### Scope of CpBXs

**Scheme 2:**
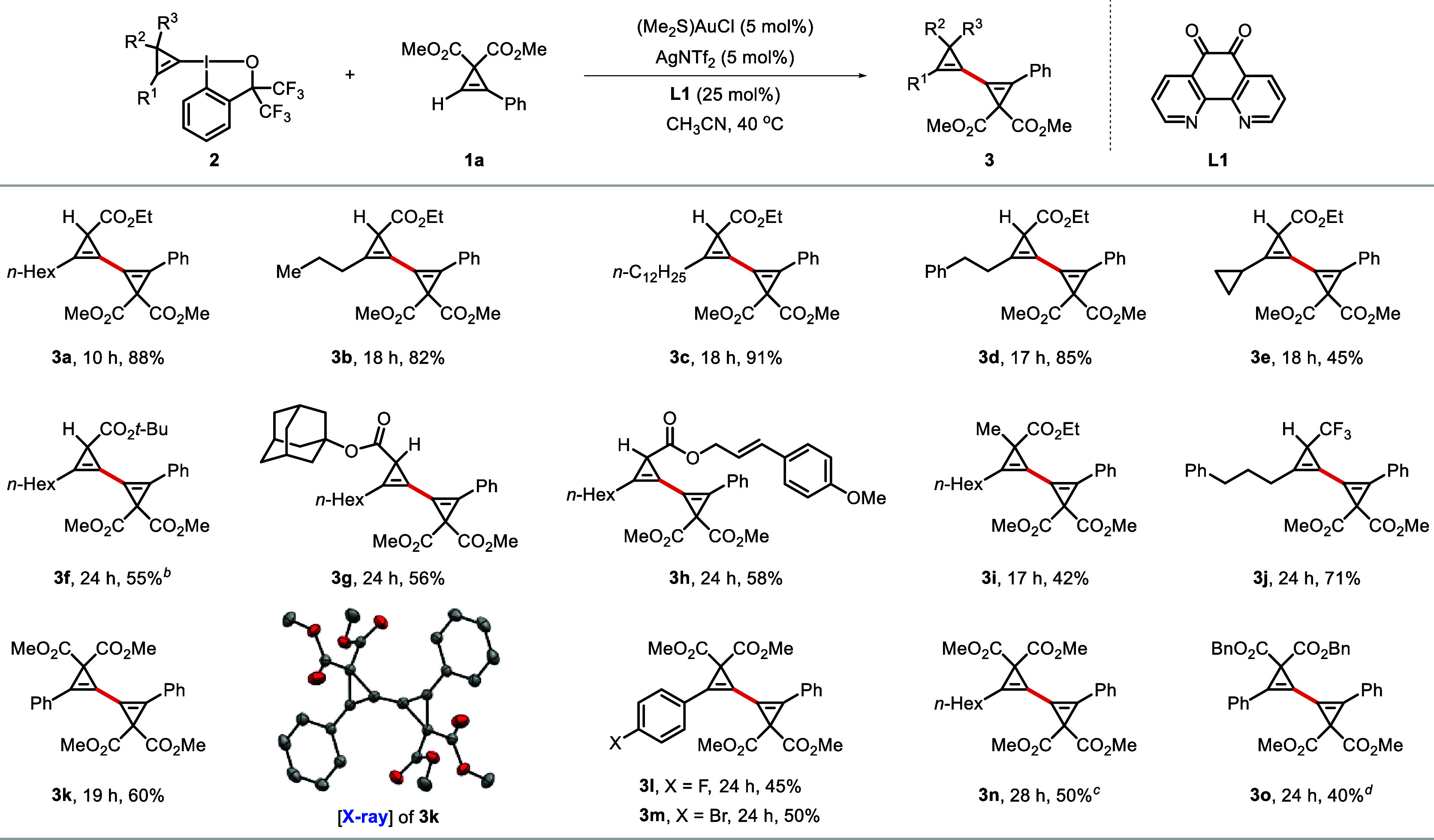
Scope of Cyclopropenyl Benziodoxoles in the Cyclopropenyl Cross-Coupling Reaction Reaction conditions: CpBX **2** (130 μmol, 1.30 equiv), terminal cyclopropene **1a** (100 μmol, 1.00 equiv), (Me_2_S)AuCl (5.00 mol %), AgNTf_2_ (5.00 mol %) and **L1** (25.0 mol %) were stirred in CH_3_CN (2.0 mL) at 40 °C for the indicated time, unless noted otherwise. The reaction mixture was stirred until complete consumption of the terminal cyclopropene was observed by TLC analysis, or until no further reaction progress could be seen. Isolated yields are given. CpBX **2f** (1.03 equiv) was used instead. CpBX **2n** (2.00 equiv) was used instead. CpBX **2o** (1.15 equiv) was used instead.

Having established optimized conditions, the scope and limitations of our cyclopropenyl cross-coupling protocol were investigated. We first examined the scope of cyclopropenyl benziodoxoles (CpBXs) using cyclopropene **1a** as the representative coupling partner (Scheme 2). CpBXs bearing different alkyl substituents (R^1^) attached to the cyclopropene were suitable reaction partners and furnished the desired products (**3b** and **3c**) in excellent yields. Introduction of a phenyl group on the alkyl chain of R^1^ was well tolerated, and the cross-coupled product **3d** was obtained in 85% yield. CpBX **2e** with a cyclopropyl group attached to the cyclopropene underwent the cross-coupling reaction smoothly to deliver the desired product **3e** with three connected three-membered rings. CpBXs featuring bulkier ester substituents, such as *tert*-butyl (**3f**) and adamantyl (**3g**), still underwent the reaction. The inclusion of an allylic group as the ester substituent of CpBX was also possible (**3h**). A CpBX bearing an additional alkyl substituent at the C3 position of the cyclopropene underwent smooth cross-coupling reaction to give fully substituted 1,1′-bicyclopropenyl **3i** in 42% yield. Remarkably, trifluoromethyl-substituted CpBX could also be used in our Au/Ag dual-catalyzed cyclopropenyl cross-coupling protocol to give **3j** in 71% yield. Furthermore, CpBXs derived from cyclopropene substrates bearing two methyl ester groups at the C3 position were also excellent substrates, as showcased by the formation of phenyl-(**3k**), fluoroaryl-(**3l**), bromoaryl-(**3m**) and alkyl-(**3n**) substituted 1,1′-bicyclopropenyls. A CpBX derivative with two benzyl ester substituents at the C3 position, afforded the corresponding cross-coupled product **3o** in 40% yield. In the case of product **3k**, it was possible to unambiguously establish the molecular connectivity by single-crystal X-ray diffraction, which revealed an extended flat π-conjugated system (Scheme 2; CCDC 2321753).

### Scope of Terminal Cyclopropenes

**Scheme 3:**
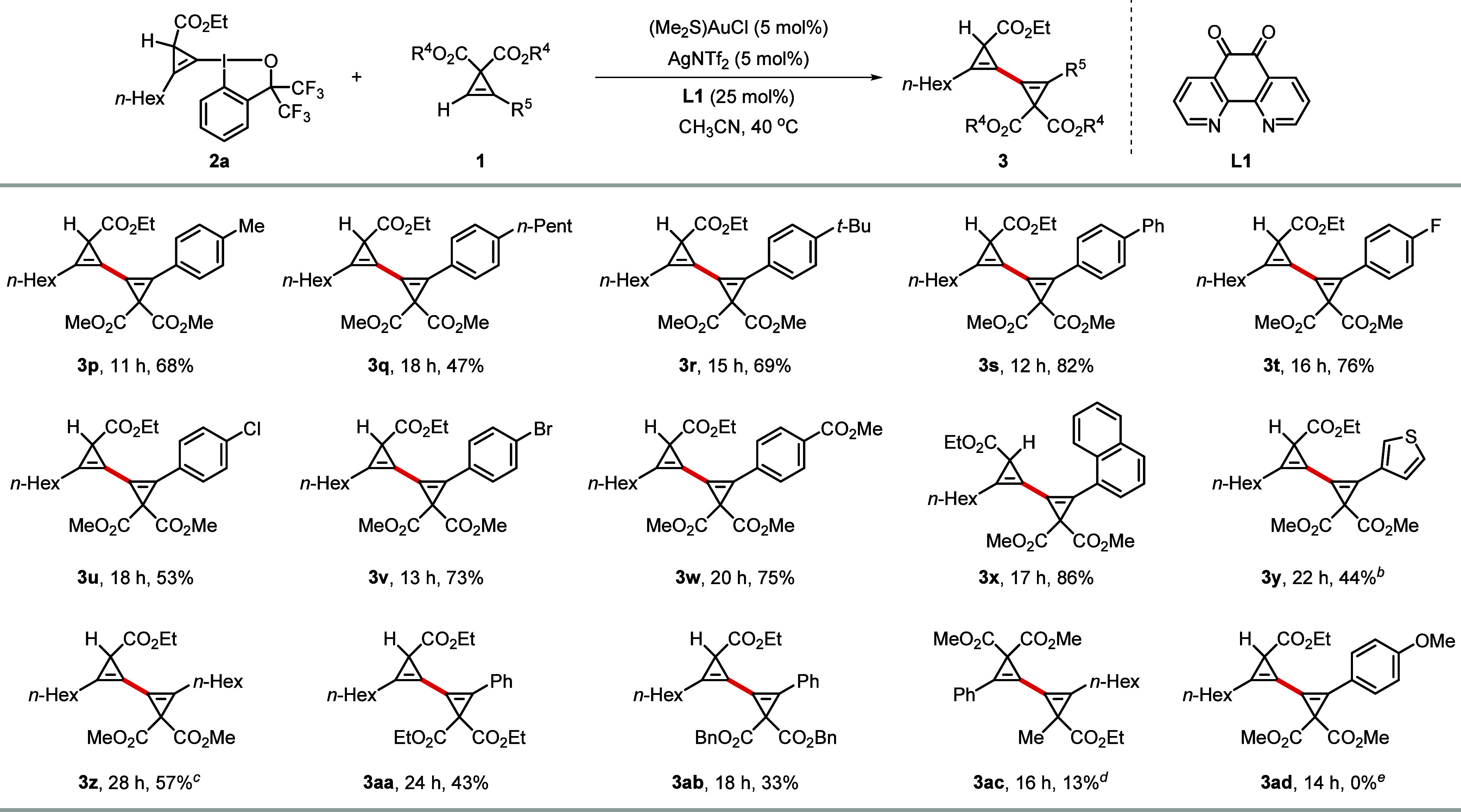
Scope of Terminal Cyclopropenes in the Cyclopropenyl Cross-Coupling Reaction Reaction conditions: CpBX **2a** (130 μmol, 1.30 equiv), terminal cyclopropene **1** (100 μmol, 1.00 equiv), (Me_2_S)AuCl (5.00 mol %), AgNTf_2_ (5.00 mol %) and **L1** (25.0 mol %) were stirred in CH_3_CN (2.0 mL) at 40 °C for the indicated time, unless noted otherwise. Isolated yields are given. CpBX **2a** (1.50 equiv) was used instead. CpBX **2a** (2.00 equiv) was used instead. CpBX **2k** (2.00 equiv), (Me_2_S)AuCl (10.0 mol %), AgNTf_2_ (10.0 mol %) and **L1** (50.0 mol %) were used instead. Cyclopropene **1p** was recovered in 98% yield.

Next, we examined the scope of terminal cyclopropenes using CpBX **2a** as the representative cross-coupling partners (Scheme 3). Our process worked well for terminal cyclopropenes with aryl rings substituted with alkyl or aryl functionalities such as methyl (**3p**), *n*-pentyl (**3q**), *tert*-butyl (**3r**) and phenyl (**3s**) in the *para* position and gave the cross-coupled products in 47–82% yield. The presence of fluorine in the *para*-position of the phenyl-substituted terminal cyclopropene (**3t**) was well tolerated. Similarly, the halogens Cl (**3u**) and Br (**3v**) were compatible with the coupling conditions, thus offering powerful handles for further derivations through established C-halogen cross-coupling protocols. An ester-substituted terminal cyclopropene gave **3w** in 75% yield. Polyaromatic or heteroaryl-substituted terminal cyclopropenes were also tolerated, as exemplified by the synthesis of 1,1′-bicyclopropenyls substituted with naphthalene (**3x**) and thiophene (**3y**) rings. Gratifyingly, an *n*-hexyl-substituted terminal cyclopropene was also a viable coupling partner and underwent cross coupling to give **3z** in 57% yield. Encouragingly, terminal cyclopropenes with other *gem*-diester substituents at the C3 position, such as ethyl (**3aa**) and benzyl (**3ab**) ester groups, were also suitable for this coupling protocol, affording the corresponding cross-coupled products in 43% and 33% yield, respectively. A terminal cyclopropene with only one acceptor group on the C3 position was less reactive presumably due to the reduced efficiency of silver-catalyzed C–H activation of monoester-substituted terminal cyclopropenes, but product **3ac** could still be obtained in 13% yield using a higher catalyst loading. Aryl-substituted terminal cyclopropenes with strong electron-donating groups, such as a methoxy group (**3ad**), were not competent coupling partners under the current optimal conditions. Generally, terminal cyclopropenes substituted with electron-withdrawing functional groups react faster and give higher yields, while electron-rich terminal cyclopropenes suffer from slower reaction rate or even no reaction at all. For the reactivity of CpBXs, the monoester substituted CpBXs are more reactive and react faster than the *gem*-diester substituted CpBXs.

### Synthetic Transformations

**Scheme 4:**
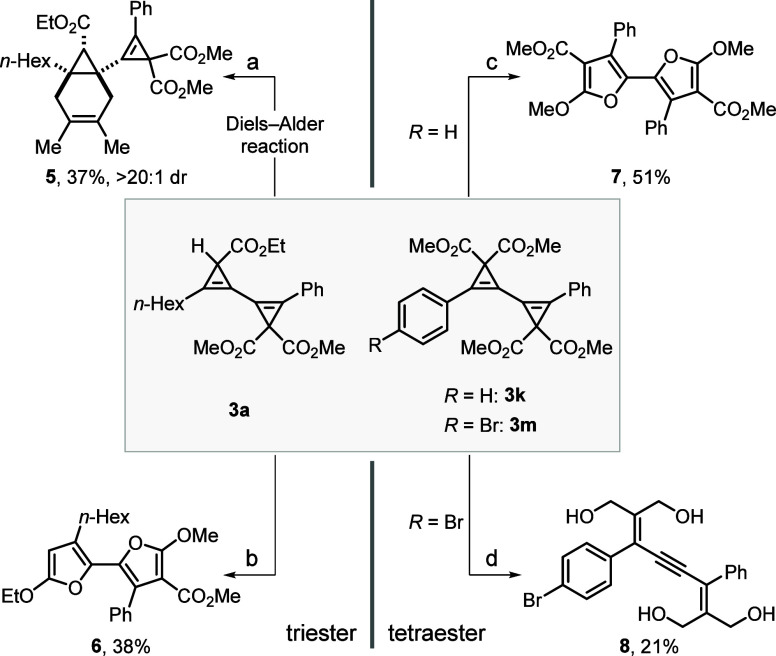
Synthetic Transformations of 1,1′-Bicyclopropenyl Derivatives Reaction conditions: (a) 2,3-dimethylbuta-1,3-diene, 80 °C, 24 h; (b) [Rh(cod)Cl]_2_ (6.00 mol %), (*rac*)-BINAP (12.0 mol %), THF, 100 °C, 10 h; (c) [Rh(cod)Cl]_2_ (6.00 mol %), (*rac*)-BINAP (12.0 mol %), THF, 80 °C, 9 h; (d) DIBAL-H (12.0 equiv), THF, −78 °C to rt, 16 h.

In order to explore the suitability of the obtained 1,1′-bicyclopropenyls for downstream manipulations, a series of synthetic modifications were executed (Scheme 4). A Diels–Alder reaction of the triester-substituted 1,1′-bicyclopropenyl **3a** with 2,3-dimethylbutadiene gave fused bicycle **5** in 37% yield and >20:1 dr (Scheme 4a). Interestingly, the intermolecular [4 + 2] cycloaddition takes place preferentially on the monoester-substituted cyclopropenyl moiety instead of the one substituted by the *gem*-diester, presumably due to the lower steric hindrance. It is well-known that cyclopropenes can undergo metal-catalyzed cycloisomerization reactions involving the cleavage of the cyclopropene C–C bond, forming an electrophilic vinyl metal-carbene species.^12,20^ The unsymmetrical bifuran **6** could be obtained in 38% yield by a rhodium-catalyzed cycloisomerization of **3a** (Scheme 4b).^22^ It is worth noting that multifunctionalized bifuran structures possess interesting optoelectronic properties and have potential applications in organic electronic materials^23^ and our protocol highlights a rare example of forging unsymmetrical bifuran units via transition metal catalysis. Under similar reaction conditions, tetraester-substituted 1,1′-bicyclopropenyl **3k** underwent the same rhodium-catalyzed cycloisomerization, giving bifuran **7** in 51% yield (Scheme 4c). Moreover, an attempt of selective reduction of the ester functionalities in **3m** with diisobutylaluminum hydride (DIBAL-H) afforded conjugated enyne **8**,^24^ instead of the expected reduced 1,1′-bicyclopropenyl derivative (Scheme 4d). The isolation of compound **8** as the sole product of the reaction (21% yield) indicated the reduction of the *gem*-diesters and the concomitant ring-opening reaction of the cyclopropenes.

### Mechanistic Investigations

**Scheme 5:**
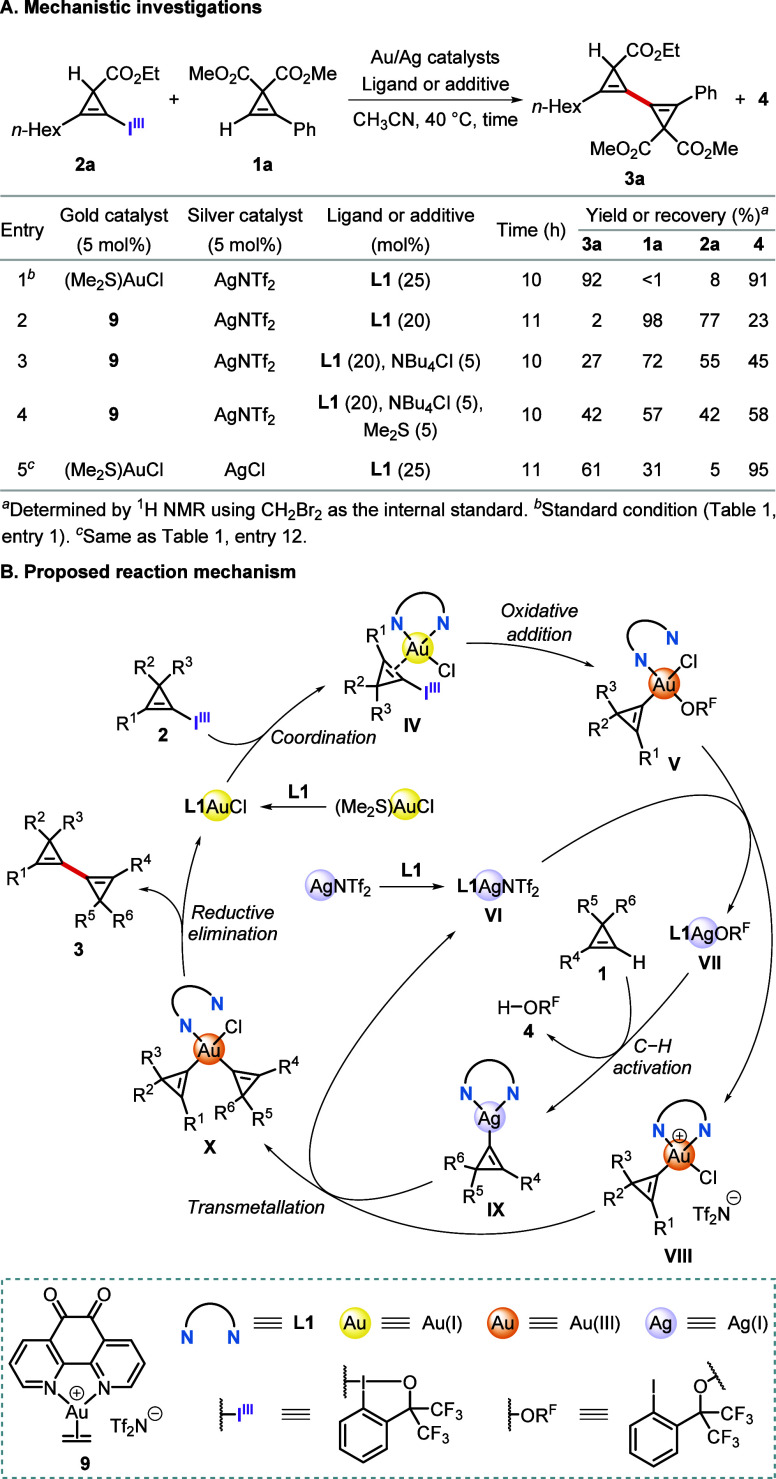
Mechanistic Studies of Synergistic Au/Ag Dual-Catalyzed Cyclopropenyl Cross-Coupling

Finally, we performed further mechanistic experiments to gain some insights into the reaction mechanism (Scheme 5). The cationic gold(I)–ethylene complex **9** was first prepared to probe the effect of chloride in this reaction. When used instead of (Me_2_S)AuCl, cationic gold(I) complex **9** gave only 2% yield of product **3a** with most **1a** (98%) recovered (Scheme 5A, entry 1 vs 2). In contrast, when 5 mol % of **9** and 5 mol % of NBu_4_Cl were used, **3a** was obtained in 27% yield (entry 3). The yield of **3a** could be further improved to 42% by adding 5 mol % of Me_2_S as an additive (entry 4). These control experiments confirmed the critical role of chloride in this reaction as a supporting ligand of the gold catalyst. The poor catalytic performance of adding external chloride source with **9** could be attributed to the competing consumption of chloride by the existing silver cation, such detrimental effect could be attenuated by adding the dummy ligand Me_2_S (entry 3 vs 4). Next, we sought to gain a deeper understanding of the silver-catalyzed C–H activation of terminal cyclopropenes to confirm the proposal of Hashmi and co-workers.^18c^ The replacement of cationic AgNTf_2_ with neutral AgCl resulted in a dramatic drop in yield of **3a** (entry 5), implying a dominant role of cationic silver species in catalyzing the C–H activation of cyclopropenes. Thus, the lower yields of **3a** in Scheme 5A, entries 3 and 4 can be further attributed to the formation of neutral AgCl, which resulted in a decreased efficiency of the C–H activation of cyclopropenes.

Based on these observations, a plausible reaction mechanism is proposed (Scheme 5B). After forming the catalytically active species **L1**AuCl via a facile ligand exchange of (Me_2_S)AuCl with **L1**, the subsequent coordination of CpBXs **2** should also be relatively fast and lead to a transient π-type Au(I) species **IV**. Next, oxidation of gold(I) to gold(III) with the concomitant transfer of the cyclopropenyl moiety would generate the square-planar Au(III)–cyclopropenyl species **V**. The viability of this oxidative addition step had been demonstrated computationally in our previous work.^19^ As proposed by Hashmi and co-workers,^18c^ the alkoxy anion (R^F^O^–^) is then transferred from gold(III) to silver(I) to form **L1**AgOR^F^ (**VII**). C–H activation of terminal cyclopropenes **1** then affords Ag(I)–cyclopropenyl species **IX**. The cyclopropenyl group is then transferred from **IX** to the highly electrophilic Au(III)–cyclopropenyl species **VIII** to generate dicyclopropenylgold(III) species **X**, which, upon reductive elimination, would yield the cross-coupled product **3** with regeneration of the catalysts.

## Conclusions

In summary, we have developed the first cyclopropenyl cross-coupling reaction through a synergistic Au/Ag bimetallic catalysis strategy. This cross-coupling protocol has been validated as a general, modular and straightforward method to access diverse symmetrical and unsymmetrical multisubstituted 1,1′-bicyclopropenyl derivatives from readily available staring materials, which could not be achieved with reported methods. Thanks to the mild conditions, the reaction exhibits a broad substrate scope and good functional-group compatibility. The potential synthetic utility of the obtained products has been demonstrated by their further derivatization to bifurans. We hope that this study will not only reduce the barrier for applications of these fascinating benzene’s isomers in functional small molecule discovery, but also stimulate interest in leveraging synergistic bimetallic catalysis to unlock other remaining challenges in cross-coupling reactions.

## Supporting Information Available

Experimental procedures, characterization of new compounds, CIF file, and spectroscopic data (PDF)

## Notes

Raw NMR, MS, and IR data are available at zenodo.org: https://doi.org 10.5281/zenodo.13831798.
