# Supplementary material for: Forging 1,1′-Bicyclopropenyls by Synergistic Au/Ag Dual-Catalyzed Cyclopropenyl Cross-Coupling
Source: J Am Chem Soc. 2024 Oct 18;146(43):29712–9. doi: 10.1021/jacs.4c10996 (PMC11528445; doi:10.1021/jacs.4c10996)
Supplement: Supplementary file 1 — ja4c10996_si_001.pdf [file ja4c10996_si_001.pdf]

Supporting Information for

Forging 1,1'-Bicyclopropenyls by Synergistic Au/Ag Dual-Catalyzed Cyclopropenyl Cross-Coupling

Xiangdong Li and Jérôme Waser*

Institute of Chemical Sciences and Engineering, Ecole Polytechnique Fédérale de Lausanne, 1015 Lausanne, Switzerland

*To whom correspondence should be addressed: jerome.waser@epfl.ch

Table of Contents

1. General Information 3
2. Development of Cyclopropenyl Hypervalent Iodine Reagents 4
 2.1. Synthesis of Terminal Cyclopropenes by Rhodium Catalysis 4
 2.2. Synthesis of Hypervalent Iodine Precursors 13
 2.3. Synthesis of Cyclopropenyl Benziodoxoles (CpBXs) 14
3. Optimization of the Synergistic Au/Ag Dual-Catalyzed Cyclopropenyl Cross-Coupling 22
 3.1. Evaluation of Bidentate Ligands 22
 3.2. Evaluation of the Loading of the Silver Catalyst 23
 3.3. Evaluation of the Loading of the Ligand and Reaction Temperature 24
 3.4. Evaluation of the Silver Salts 25
 3.5. Variations from the Standard Conditions 26
4. Substrate Scope of Au/Ag Dual-Catalyzed Cyclopropenyl Cross-Coupling 27
 4.1. Substrate Scope of Cyclopropenyl Benziodoxoles (CpBXs) 28
 4.2. Substrate Scope of Terminal Cyclopropenes 35
5. Transformations of Products and Applications 42
 5.1. Diels–Alder Reaction of **3a** with 2,3-Dimethylbutadiene 42
 5.2. Rh(I)-Catalyzed Cycloisomerization of **3a** 42
 5.3. Rh(I)-Catalyzed Cycloisomerization of **3k** 43
 5.4. Reduction of 1,1'-Bicyclopropenyl Derivative **3m** Using DIBAL-H 43
6. Mechanistic Investigations 44
 6.1 Preparation of Cationic Gold(I)-Ethylene Complex (**9**) 44
 6.2 Control Experiments for Determining the Catalytically Active Species 44
7. Computational Section 45
 7.1 Computational Methods 45
 7.2 Calculated Energies of Selected Strained C_6H_6 Isomers 45
 7.3 Structure of 1,1'-Bicyclopropenyl 46
 7.4 Frontier Molecular Orbitals of 1,1'-Bicyclopropenyl 46
 7.5 Cartesian Coordinates of Optimized Geometries 47
8. Attempts of Using Stille Coupling to Synthesize 1,1'-Bicyclopropenes 48
 8.1 Synthesis of Stannylcyclopropene 48
 8.2 Synthesis of Cyclopropenyl Iodide 49
 8.3 Attempts of Stille Coupling Using Stannylcyclopropene and Cyclopropenyl Iodide 49
9. Single Crystal X-ray Diffraction Analysis 51
 9.1. Crystal Data and Structure Refinement for **3k** 51
10. NMR Spectra 52
11. References 142

1. General Information

All reactions were carried out using standard Schlenk technique under nitrogen unless otherwise stated. All reagents were purchased from major commercial suppliers (Sigma-Aldrich, Merck, Fluorochem, Combi-blocks, Fluka, Apollo Scientific, Fischer Scientific, Tokyo Chemical Industry, Acros Organics) and used as such unless otherwise noted. Dry solvents (DCM, THF, MeCN, toluene and Et_2O) were obtained fresh from an Innovative Technology solvent purification system having been passed through anhydrous alumina columns. 1,2-Dichloroethane (99.5%, extra dry over molecular sieve, AcrosSeal®) was purchased from Thermo Scientific Chemicals. Unless otherwise stated, solvents were used without further drying or degassing. (Me_2S)AuCl and 1,10-phenanthroline-5,6-dione (**L1**) used in the σ-type CPC transfer reactions were purchased from Sigma-Aldrich and Combi-Blocks, respectively. The AuCl (97%, 99.99%-Au) was purchased from abcr GmbH. Gold(III) chloride (99%) was purchased from Sigma-Aldrich. Ethyl diazoacetate (contains ≥13 wt. % dichloromethane) was purchased from Sigma-Aldrich (E22201-100G) and used without further purification. Reactions were monitored by thin layer chromatography (TLC) on Merck silica gel 60 F_{254} TLC glass plates and visualized with ultraviolet irradiation (254 nm) and/or potassium permanganate stain. Flash column chromatography (FCC) was carried out using Biotage Isolera One with pre-packaged silica cartridges (EcoFlex Silica 4 g, 12 g, 25 g, 40g, 80g, 120g) purchased from Büchi or C18 reverse phase chromatography (Aquarius C18AQ 20 g; 100 Å, Spherical, 30 μm, Flow rate: 10-25 mL/min). ^1H NMR spectra were recorded on a Bruker Ascend™ 400 400 MHz spectrometer and reported as chemical shifts (δ) in parts per million (ppm) relative to the residual non-deuterated solvent signal as internal reference (chloroform-*d*: 7.26 ppm; DMSO-d_6: 2.50 ppm; acetoned-d_6: 2.06 ppm; CD_3CN: 1.94 ppm). ^{13}C NMR spectra were recorded with {^1H} decoupling on a Bruker Ascend™ 400 101 MHz spectrometer and reported in ppm using the residual solvent signal as internal reference (chloroform-*d*: 77.16 ppm; DMSO-d_6: 39.52 ppm; acetoned-d_6: 29.84 ppm; CD_3CN: 1.32 ppm). ^{19}F-NMR spectra were recorded with {^1H} decoupling on a Bruker Ascend™ 400 376 MHz spectrometer. Data are reported as follows: chemical shift, multiplicity (s = singlet, d = doublet, t = triplet, q = quartet, p = pentet, sept = septet, br = broad, m = multiplet), coupling constants (Hz) and integration. NMR spectra were processed with MestReNova (version 14.0.1). High resolution mass spectrometric measurements were performed by the mass spectrometry service of ISIC at the EPFL. Electrospray-ionisation HRMS data were acquired on a Q-Tof Ultima mass spectrometer (Waters) or a Q-Tof 6530 Accurate mass spectrometer (Agilent) operated in the positive ionization mode and fitted with a standard Z-spray ion source equipped with the Lock-Spray interface. Data from the Lock-Spray were used to calculate a correction factor for the mass scale and provide accurate mass information of the analyte. Data were processed using the MassLynx 4.1 software. Atmospheric pressure photo-ionisation (APPI) HRMS measurements were done on an LTQ Orbitrap Elite instrument (Thermofisher) operated in the positive ionization mode. The raw data obtained from the Q-TOF Waters instrument does not consider the mass of the electron for the ion, the obtained raw data has been corrected by removing (positive ionization) or adding (negative ionization) the mass of the electron (0.5 mDa). Infrared spectra were recorded using a JASCO FT/IR-4100 Fourier Transform Infrared Spectrometer at room temperature, and the stretching frequencies are reported in wavenumbers (cm^{-1}) (s = strong, m = medium, w = weak). Melting points were measured using a Büchi Melting Point B-540 and were uncorrected.

2. Development of Cyclopropenyl Hypervalent Iodine Reagents

2.1. Synthesis of Terminal Cyclopropenes by Rhodium Catalysis

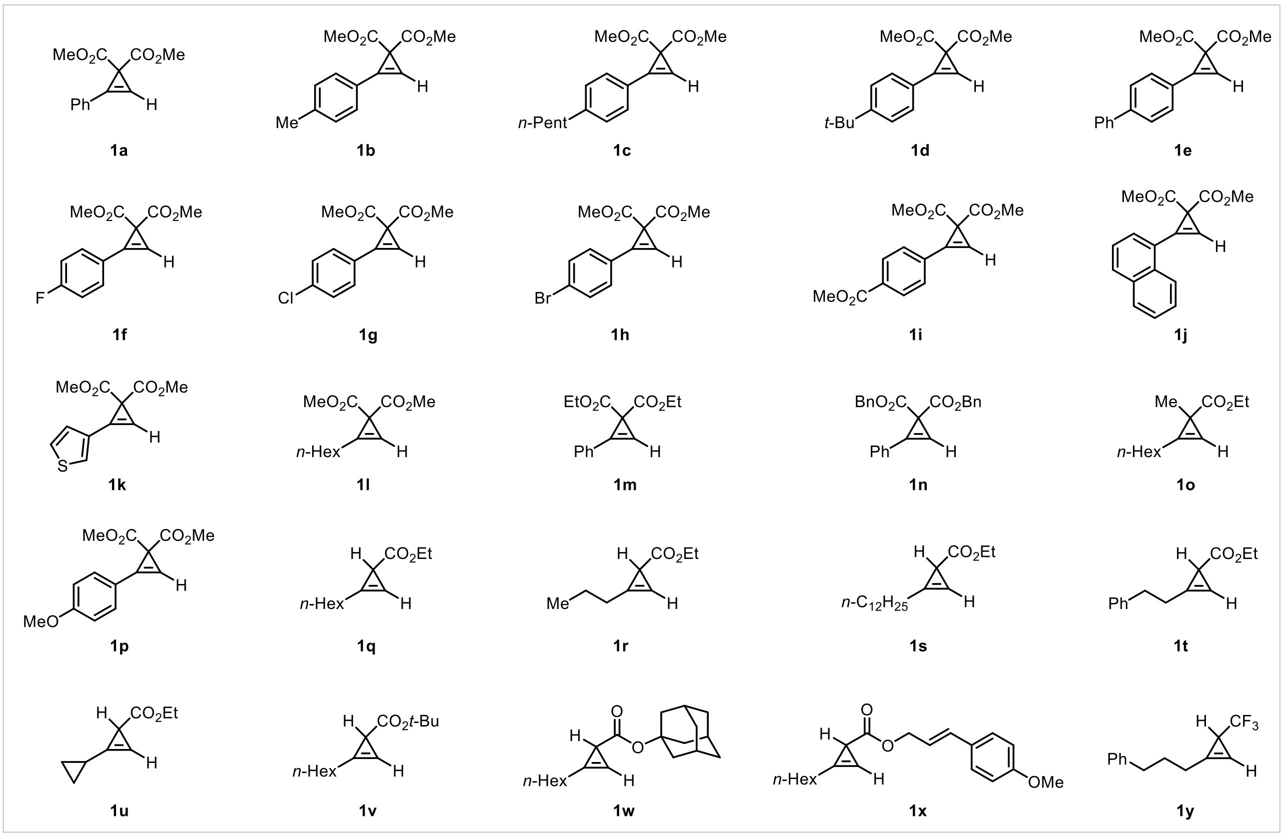

General Procedure A (**GPA**)[1]:

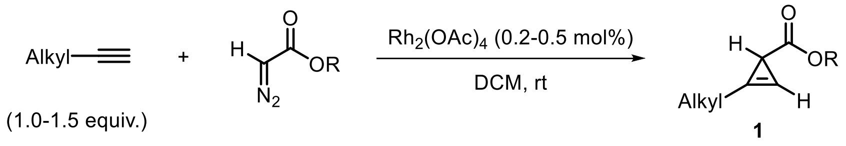

GPA: To a stirred solution of $Rh_2(OAc)_4$ (0.2-0.5 mol%) and terminal alkyne (1.0-1.5 equiv.) in CH_2Cl_2 (1.00-1.50 M) at room temperature was added a solution of diazo ester (1.0 equiv.) in CH_2Cl_2 (1.00 M) via syringe pump over 8 hours under nitrogen. After the addition was complete, the mixture was stirred for additional 8 hours, filtered through a short pad of Celite® eluting with CH_2Cl_2, and concentrated under reduced pressure. Purification of the residue by column chromatography (pentane/ethyl acetate) to afford **1**.

2.1.1. Synthesis and characterization of dimethyl 2-phenylcycloprop-2-ene-1,1-dicarboxylate (1a)

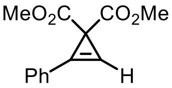

1a

Following **GPA**, phenylacetylene (0.99 mL, 9.00 mmol, 1.50 equiv.), dimethyl diazomalonate (949 mg, 6.00 mmol, 1.00 equiv.), $Rh_2(OAc)_4$ (13.3 mg, 30.0 µmol, 0.500 mol%) and CH_2Cl_2 (10 mL) were used. Column chromatography on silica gel (eluent: pentane/ethyl acetate = 5:1) afforded **1a** in 70% yield (976 mg, 4.20 mmol) as a colorless solid. **TLC**: R_f (*n*-hexane: EtOAc = 5:1) = 0.24; **^{1}H NMR** (400 MHz, $CDCl_3$) δ 7.64 – 7.61 (m, 2H, Ar*H*), 7.54 – 7.30 (m, 3H, Ar*H*), 6.89 (s, 1H, C=C*H*), 3.73 (s, 6H, OC*H*$_3$); **^{13}C**

NMR (101 MHz, $CDCl_3$) δ 171.3, 130.7, 130.5, 129.0, 124.1, 112.4, 95.4, 52.5, 32.9; **HRMS** (ESI/QTOF) m/z: $[M + Na]^+$ Calcd for $C_{13}H_{12}NaO_4^+$ 255.0628; Found 255.0630. The NMR spectroscopic data is consistent with previous report[2].

2.1.2. Synthesis and characterization of dimethyl 2-(*p*-tolyl)cycloprop-2-ene-1,1-dicarboxylate (1b)

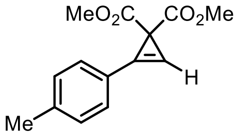

1b

Following **GPA**, 1-ethynyl-4-methylbenzene (523 mg, 4.50 mmol, 1.50 equiv.), dimethyl 2-diazomalonate (474 mg, 3.00 mmol, 1.00 equiv.), $Rh_2(OAc)_4$ (6.63 mg, 15.0 µmol, 0.500 mol%) and CH_2Cl_2 (10 mL) were used. Column chromatography on silica gel (eluent: pentane/ethyl acetate = 4:1) afforded **1b** in 32% yield (239 mg, 969 µmol) as a colorless solid. **TLC**: R_f (*n*-hexane: EtOAc = 3:1) = 0.38; **^{1}H NMR** (400 MHz, $CDCl_3$) δ 7.56 – 7.46 (m, 2H, Ar*H*), 7.28 – 7.18 (m, 2H, Ar*H*), 6.81 (s, 1H, C=C*H*), 3.72 (s, 6H, CO_2CH_3), 2.39 (s, 3H, Ar-CH_3); **^{13}C NMR** (101 MHz, $CDCl_3$) δ 171.4, 141.3, 130.5, 129.8, 121.3, 112.2, 94.1, 52.5, 32.9, 21.8; **HRMS** (ESI/QTOF) m/z: $[M + Na]^+$ Calcd for $C_{14}H_{14}NaO_4^+$ 269.0784; Found 269.0791. The NMR spectroscopic data is consistent with previous report[3].

2.1.3. Synthesis and characterization of dimethyl 2-(4-pentylphenyl)cycloprop-2-ene-1,1-dicarboxylate (1c)

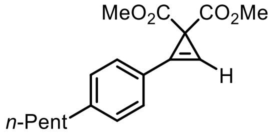

1c

Following **GPA**, 1-ethynyl-4-pentylbenzene (775 mg, 4.50 mmol, 1.50 equiv.), dimethyl 2-diazomalonate (474 mg, 3.00 mmol, 1.00 equiv.), $Rh_2(OAc)_4$ (6.63 mg, 15.0 µmol, 0.500 mol%) and CH_2Cl_2 (10 mL) were used. Column chromatography on silica gel (eluent: pentane/ethyl acetate = 4:1) afforded **1c** in 25% yield (223 mg, 736 µmol) as a colorless oil. **TLC**: R_f (*n*-hexane: EtOAc = 4:1) = 0.36; **^{1}H NMR** (400 MHz, $CDCl_3$) δ 7.58 – 7.47 (m, 2H, Ar*H*), 7.27 – 7.23 (m, 2H, Ar*H*), 6.81 (s, 1H, C=C*H*), 3.72 (s, 6H, CO_2CH_3), 2.63 (dd, *J* = 8.7, 6.8 Hz, 2H, Ar-CH_2), 1.68 – 1.54 (m, 2H, CH_2), 1.32 (qt, *J* = 5.9, 2.5 Hz, 4H, CH_2), 0.94 – 0.80 (m, 3H, CH_3); **^{13}C NMR** (101 MHz, $CDCl_3$) δ 171.4, 146.3, 130.5, 129.2, 121.4, 112.2, 94.1, 52.5, 36.1, 32.9, 31.5, 31.1, 22.6, 14.1; **IR** (ν_{max}, cm^{-1}) 2953 (w), 2930 (w), 2857 (w), 1732 (s), 1643 (m), 1607 (w), 1436 (m), 1240 (s), 1139 (m), 1065 (w), 1023 (w), 982 (w), 843 (w), 799 (w), 731 (w); **HRMS** (ESI/QTOF) m/z: $[M + Na]^+$ Calcd for $C_{18}H_{22}NaO_4^+$ 325.1410; Found 325.1407. The NMR spectroscopic data is consistent with previous report[4].

2.1.4. Synthesis and characterization of dimethyl 2-(4-(*tert*-butyl)phenyl)cycloprop-2-ene-1,1-dicarboxylate (1d)

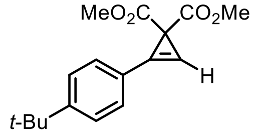

1d

Following **GPA**, 1-(*tert*-butyl)-4-ethynylbenzene (550 mg, 3.48 mmol, 1.16 equiv.), dimethyl 2-diazomalonate (474 mg, 3.00 mmol, 1.00 equiv.), $Rh_2(OAc)_4$ (6.63 mg, 15.0 µmol, 0.500 mol%) and CH_2Cl_2 (10 mL) were used. Column chromatography on silica gel (eluent: pentane/ethyl acetate = 5:1) afforded **1d** in 40% yield (348 mg, 1.21 mmol) as a colorless solid. **TLC**: R_f (*n*-hexane: EtOAc = 4:1) =

0.39; **^{1}H NMR** (400 MHz, $CDCl_3$) δ 7.61 – 7.52 (m, 2H, Ar*H*), 7.51 – 7.41 (m, 2H, Ar*H*), 6.82 (s, 1H, C=C*H*), 3.73 (s, 6H, CO_2CH_3), 1.33 (s, 9H, *t-Bu*); **^{13}C NMR** (101 MHz, $CDCl_3$) δ 171.4, 154.3, 130.3, 126.1, 121.2, 112.1, 94.3, 52.5, 35.2, 32.8, 31.3; **HRMS** (ESI/QTOF) m/z: $[M + Na]^+$ Calcd for $C_{17}H_{20}NaO_4^+$ 311.1254; Found 311.1258. The NMR spectroscopic data is consistent with previous report[2].

2.1.5. Synthesis and characterization of dimethyl 2-([1,1'-biphenyl]-4-yl)cycloprop-2-ene-1,1-dicarboxylate (1e)

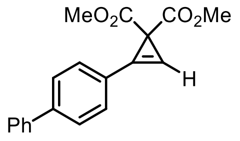

1e

Following **GPA**, 4-ethynyl-1,1'-biphenyl (535 mg, 3.00 mmol, 1.00 equiv.), dimethyl 2-diazomalonate (474 mg, 3.00 mmol, 1.00 equiv.), $Rh_2(OAc)_4$ (6.63 mg, 15.0 µmol, 0.500 mol%) and CH_2Cl_2 (10 mL) were used. Column chromatography on silica gel (eluent: pentane/ethyl acetate = 4:1) afforded **1e** in 47% yield (432 mg, 1.40 mmol) as an off-white amorphous solid. **TLC**: R_f (*n*-hexane: EtOAc = 4:1) = 0.24; **^{1}H NMR** (400 MHz, $CDCl_3$) δ 7.77 – 7.65 (m, 4H, Ar*H*), 7.63 – 7.57 (m, 2H, Ar*H*), 7.53 – 7.43 (m, 2H, Ar*H*), 7.43 – 7.34 (m, 1H, Ar*H*), 6.92 (s, 1H, C=C*H*), 3.75 (s, 6H, CO_2CH_3); **^{13}C NMR** (101 MHz, $CDCl_3$) δ 171.3, 143.6, 140.2, 130.9, 129.1, 128.1, 127.8, 127.3, 122.9, 112.1, 95.4, 52.6, 33.0; **IR** (ν_{max}, cm^{-1}) 3149 (w), 2955 (w), 1732 (s), 1484 (w), 1438 (m), 1405 (w), 1280 (s), 1248 (s), 1189 (m), 1122 (s), 1063 (w), 1005 (w), 981 (w), 913 (w), 846 (w), 764 (m), 736 (m); **HRMS** (ESI/QTOF) m/z: $[M + Na]^+$ Calcd for $C_{19}H_{16}NaO_4^+$ 331.0941; Found 331.0942.

2.1.6. Synthesis and characterization of dimethyl 2-(4-fluorophenyl)cycloprop-2-ene-1,1-dicarboxylate (1f)

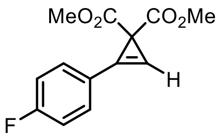

1f

Following **GPA**, 1-ethynyl-4-fluorobenzene (961 mg, 8.00 mmol, 1.00 equiv.), dimethyl diazomalonate (1.27 g, 8.00 mmol, 1.00 equiv.), $Rh_2(OAc)_4$ (17.7 mg, 40.0 µmol, 0.5 mol%) and CH_2Cl_2 (8.0 mL) were used. Column chromatography on silica gel (eluent: pentane/ethyl acetate = 4:1) afforded **1f** in 48% yield (961 mg, 3.84 mmol) as a colorless solid. **TLC**: R_f (*n*-hexane: EtOAc = 4:1) = 0.25; **^{1}H NMR** (400 MHz, $CDCl_3$) δ 7.68 – 7.57 (m, 2H, Ar*H*), 7.21 – 7.07 (m, 2H, Ar*H*), 6.86 (s, 1H, C=C*H*), 3.73 (s, 6H, OC*H*$_3$); **^{13}C NMR** (101 MHz, $CDCl_3$) δ 171.2, 165.3, 162.8, 132.6 (d, *J* = 8.8 Hz), 120.4 (d, *J* = 3.3 Hz), 116.4 (d, *J* = 22.3 Hz), 111.5, 95.0 (d, *J* = 2.6 Hz), 52.6, 33.0; **^{19}F NMR** (376 MHz, $CDCl_3$) δ -108.1. The NMR spectroscopic data is consistent with previous report[5].

2.1.7. Synthesis and characterization of dimethyl 2-(4-chlorophenyl)cycloprop-2-ene-1,1-dicarboxylate (1g)

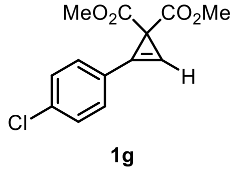

1g

Following **GPA**, 1-chloro-4-ethynylbenzene (410 mg, 3.00 mmol, 1.00 equiv.), dimethyl 2-diazomalonate (474 mg, 3.00 mmol, 1.00 equiv.), $Rh_2(OAc)_4$ (6.63 mg, 15.0 µmol, 0.500 mol%) and CH_2Cl_2 (10 mL) were used. Column chromatography on silica gel (eluent: pentane/ethyl acetate = 5:1) afforded **1g** in 26% yield (207 mg, 778 µmol) as an off-white amorphous solid. **TLC**: R_f (*n*-hexane: EtOAc = 4:1) = 0.34; **^{1}H**

NMR (400 MHz, $CDCl_3$) δ 7.61 – 7.50 (m, 2H, Ar*H*), 7.47 – 7.38 (m, 2H, Ar*H*), 6.92 (s, 1H, C=C*H*), 3.73 (s, 6H, CO_2CH_3); **^{13}C NMR** (101 MHz, $CDCl_3$) δ 171.1, 137.0, 131.7, 129.4, 122.6, 111.6, 96.1, 52.6, 33.0; **IR** (ν_{max}, cm^{-1}) 3149 (w), 3000 (w), 2953 (w), 2845 (w), 1725 (s), 1592 (w), 1485 (m), 1435 (w), 1400 (w), 1280 (s), 1247 (s), 1194 (w), 1142 (w), 1061 (s), 1015 (w), 986 (w), 914 (w), 833 (m), 724 (w); **HRMS** (ESI/QTOF) m/z: $[M + Na]^+$ Calcd for $C_{13}H_{11}ClNaO_4^+$ 289.0238; Found 289.0243.

2.1.8. Synthesis and characterization of dimethyl 2-(4-bromophenyl)cycloprop-2-ene-1,1-dicarboxylate (1h)

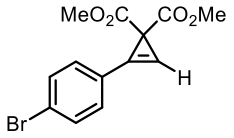

1h

Following **GPA**, 1-bromo-4-ethynylbenzene (1.21 g, 6.70 mmol, 1.00 equiv.), dimethyl 2-diazomalonate (1.33 g, 8.43 mmol, 1.26 equiv.), $Rh_2(OAc)_4$ (14.8 mg, 33.5 µmol, 0.500 mol%) and CH_2Cl_2 (20 mL) were used. Column chromatography on silica gel (eluent: pentane/ethyl acetate = 4:1) afforded **1h** in 43% yield (896 mg, 2.88 mmol) as a colorless solid. **TLC**: R_f (*n*-hexane: EtOAc = 4:1) = 0.28; **^{1}H NMR** (400 MHz, $CDCl_3$) δ 7.63 – 7.55 (m, 2H, Ar*H*), 7.53 – 7.43 (m, 2H, Ar*H*), 6.93 (s, 1H, C=C*H*), 3.73 (s, 6H, CO_2CH_3); **^{13}C NMR** (101 MHz, $CDCl_3$) δ 171.0, 132.4, 131.8, 125.3, 123.1, 111.7, 96.3, 52.6, 33.0; **HRMS** (ESI/QTOF) m/z: $[M + Na]^+$ Calcd for $C_{13}H_{11}BrNaO_4^+$ 332.9733; Found 332.9734. The NMR spectroscopic data is consistent with previous report[2].

2.1.9. Synthesis and characterization of dimethyl 2-(4-(methoxycarbonyl)phenyl)cycloprop-2-ene-1,1-dicarboxylate (1i)

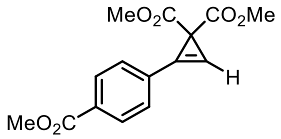

1i

Following **GPA**, methyl 4-ethynylbenzoate (481 mg, 3.00 mmol, 1.00 equiv.), dimethyl 2-diazomalonate (474 mg, 3.00 mmol, 1.00 equiv.), $Rh_2(OAc)_4$ (6.63 mg, 15.0 µmol, 0.500 mol%) and CH_2Cl_2 (10 mL) were used. Column chromatography on silica gel (eluent: pentane/ethyl acetate = 4:1) afforded **1i** in 36% yield (311 mg, 1.07 mmol) as an off-white amorphous solid. **TLC**: R_f (*n*-hexane: EtOAc = 4:1) = 0.26; **^{1}H NMR** (400 MHz, $CDCl_3$) δ 8.18 – 8.00 (m, 2H, Ar*H*), 7.73 – 7.61 (m, 2H, Ar*H*), 7.05 (s, 1H, C=C*H*), 3.93 (s, 3H, Ar-CO_2CH_3), 3.73 (s, 6H, $C(CO_2CH_3)_2$); **^{13}C NMR** (101 MHz, $CDCl_3$) δ 170.9, 166.4, 131.8, 130.3, 130.2, 128.2, 111.9, 98.3, 52.6, 52.5, 33.1; **IR** (ν_{max}, cm^{-1}) 3136 (w), 2955 (w), 1723 (s), 1607 (w), 1436 (m), 1408 (w), 1280 (s), 1192 (w), 1110 (m), 1064 (m), 1019 (w), 963 (w), 863 (w), 774 (w), 713 (w); **HRMS** (ESI/QTOF) m/z: $[M + Na]^+$ Calcd for $C_{15}H_{14}NaO_6^+$ 313.0683; Found 313.0687.

2.1.10. Synthesis and characterization of dimethyl 2-(naphthalen-1-yl)cycloprop-2-ene-1,1-dicarboxylate (1j)

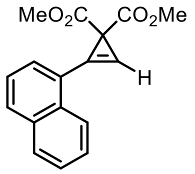

1j

Following **GPA**, 1-ethynylnaphthalene (0.85 mL, 6.00 mmol, 3.00 equiv.), dimethyl 2-diazomalonate (316 mg, 2.00 mmol, 1.00 equiv.), $Rh_2(OAc)_4$ (8.84 mg, 20.0 µmol, 1.00 mol%) and CH_2Cl_2 (20 mL) were

used. Column chromatography on silica gel (eluent: pentane/ethyl acetate = 4:1) afforded **1j** in 51% yield (288 mg, 1.02 mmol) as a colorless solid. **TLC**: R_f (*n*-hexane: EtOAc = 4:1) = 0.29; **^{1}H NMR** (400 MHz, $CDCl_3$) δ 8.42 – 8.29 (m, 1H, Ar*H*), 7.93 (ddt, *J* = 15.2, 8.2, 1.0 Hz, 2H, Ar*H*), 7.75 (dd, *J* = 7.1, 1.2 Hz, 1H, Ar*H*), 7.65 (ddd, *J* = 8.3, 6.9, 1.4 Hz, 1H, Ar*H*), 7.61 – 7.46 (m, 2H, Ar*H*), 7.22 (s, 1H, C=C*H*), 3.75 (s, 6H, CO_2CH_3); **^{13}C NMR** (101 MHz, $CDCl_3$) δ 171.3, 133.6, 132.3, 131.6, 131.0, 128.8, 127.7, 126.8, 125.6, 124.5, 120.5, 110.8, 97.5, 52.6, 31.5; **HRMS** (ESI/QTOF) m/z: $[M + Na]^+$ Calcd for $C_{17}H_{14}NaO_4^+$ 305.0784; Found 305.0784. The NMR spectroscopic data is consistent with previous report[2].

2.1.11. Synthesis and characterization of dimethyl 2-(thiophen-3-yl)cycloprop-2-ene-1,1-dicarboxylate (1k)

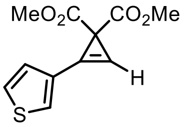

1k

Following **GPA**, 3-ethynylthiophene (325 mg, 3.00 mmol, 1.00 equiv.), dimethyl 2-diazomalonate (474 mg, 3.00 mmol, 1.00 equiv.), $Rh_2(OAc)_4$ (6.63 mg, 15.0 µmol, 0.500 mol%) and CH_2Cl_2 (10 mL) were used. Column chromatography on silica gel (eluent: pentane/ethyl acetate = 4:1) afforded **1k** in 9% yield (64.6 mg, 271 µmol) as a pale-yellow sticky oil. **TLC**: R_f (*n*-hexane: EtOAc = 4:1) = 0.27; **^{1}H NMR** (400 MHz, $CDCl_3$) δ 7.66 (dd, *J* = 3.0, 1.2 Hz, 1H, Ar*H*), 7.37 (dd, *J* = 5.0, 3.0 Hz, 1H, Ar*H*), 7.31 (dd, *J* = 5.1, 1.2 Hz, 1H, Ar*H*), 6.72 (s, 1H, C=C*H*), 3.71 (s, 6H, CO_2CH_3); **^{13}C NMR** (101 MHz, $CDCl_3$) δ 171.1, 130.0, 128.2, 127.0, 125.3, 107.1, 92.8, 52.5, 32.8; **IR** (ν_{max}, cm^{-1}) 3147 (w), 2953 (w), 2844 (w), 1723 (s), 1435 (m), 1247 (s), 1223 (s), 1190 (w), 1149 (w), 1061 (s), 989 (w), 862 (m), 793 (m), 734 (m); **HRMS** (ESI/QTOF) m/z: $[M + Na]^+$ Calcd for $C_{11}H_{10}NaO_4S^+$ 261.0192; Found 261.0192.

2.1.12. Synthesis and characterization of dimethyl 2-hexylcycloprop-2-ene-1,1-dicarboxylate (1l)

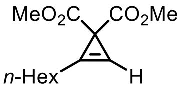

1l

Following **GPA**, oct-1-yne (1.1 mL, 7.50 mmol, 1.50 equiv.), dimethyl 2-diazomalonate (791 mg, 5.00 mmol, 1.00 equiv.), $Rh_2(OAc)_4$ (4.40 mg, 10.0 µmol, 0.2 mol%) and CH_2Cl_2 (10 mL) were used. Column chromatography on silica gel (eluent: pentane/ethyl acetate = 4:1) afforded **1l** in 69% yield (832 mg, 3.46 mmol) as a colorless oil. **TLC**: R_f (*n*-hexane: EtOAc = 4:1) = 0.28; **^{1}H NMR** (400 MHz, $CDCl_3$) δ 6.34 (t, *J* = 1.5 Hz, 1H, C=C*H*), 3.70 (s, 6H, CO_2CH_3), 2.53 (td, *J* = 7.4, 1.4 Hz, 2H, CH_2CH_2C), 1.66 – 1.51 (m, 2H, CH_2CH_2C), 1.44 – 1.17 (m, 6H, CH_2), 0.87 (t, *J* = 6.8 Hz, 3H, CH_3); **^{13}C NMR** (101 MHz, $CDCl_3$) δ 172.0, 114.7, 93.5, 52.3, 32.5, 31.5, 28.8, 26.5, 24.1, 22.6, 14.1; **HRMS** (ESI/QTOF) m/z: $[M + Na]^+$ Calcd for $C_{13}H_{20}NaO_4^+$ 263.1254; Found 263.1256. The NMR spectroscopic data is consistent with previous report[6].

2.1.13. Synthesis and characterization of diethyl 2-phenylcycloprop-2-ene-1,1-dicarboxylate (1m)

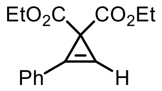

1m

Following **GPA**, ethynylbenzene (799 µL, 7.26 mmol, 1.50 equiv.), diethyl 2-diazomalonate (901 mg, 4.84 mmol, 1.00 equiv.), $Rh_2(OAc)_4$ (10.7 mg, 24.2 µmol, 0.500 mol%) and CH_2Cl_2 (15 mL) were used. Column chromatography on silica gel (eluent: pentane/ethyl acetate = 10:1) afforded **1m** in 60% yield (759 mg, 2.92 mmol) as a pale-yellow sticky oil. **TLC**: R_f (*n*-hexane: EtOAc = 5:1) = 0.38; **^{1}H NMR** (400

MHz, $CDCl_3$) δ 7.68 – 7.58 (m, 2H, Ar*H*), 7.47 – 7.38 (m, 3H, Ar*H*), 6.89 (s, 1H, C=C*H*), 4.20 (q, *J* = 7.1 Hz, 4H, $CO_2CH_2CH_3$), 1.25 (t, *J* = 7.1 Hz, 6H, $CO_2CH_2CH_3$); **^{13}C NMR** (101 MHz, $CDCl_3$) δ 171.0, 130.6, 130.5, 129.0, 124.3, 112.7, 95.5, 61.3, 33.4, 14.3; **IR** (ν_{max}, cm^{-1}) 3149 (w), 2983 (w), 2906 (w), 1723 (s), 1447 (w), 1390 (w), 1368 (w), 1274 (s), 1241 (s), 1199 (w), 1174 (w), 1144 (w), 1095 (w), 1058 (s), 1022 (m), 926 (w), 863 (w), 766 (w); **HRMS** (ESI/QTOF) m/z: $[M + Na]^+$ Calcd for $C_{15}H_{16}NaO_4^+$ 283.0941; Found 283.0944. Found 261.0192.

2.1.14. Synthesis and characterization of dibenzyl 2-phenylcycloprop-2-ene-1,1-dicarboxylate (1n)

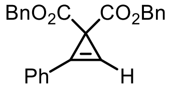

1n

Following **GPA**, phenylacetylene (613 mg, 6.00 mmol, 1.50 equiv.), dibenzyl 2-diazomalonate (1.24 g, 4.00 mmol, 1.00 equiv.), $Rh_2(OAc)_4$ (8.80 mg, 20.0 μmol, 0.5 mol%) and CH_2Cl_2 (20 mL) were used. Column chromatography on silica gel (eluent: pentane/ethyl acetate = 5:1) afforded **1n** in 48% yield (734 mg, 1.91 mmol) as a colorless oil. **TLC**: R_f (*n*-hexane: EtOAc = 5:1) = 0.32; **^{1}H NMR** (400 MHz, $CDCl_3$) δ 7.64 – 7.55 (m, 2H, Ar*H*), 7.40 (dd, *J* = 5.0, 2.0 Hz, 3H, Ar*H*), 7.31 – 7.19 (m, 10H, Ar*H*), 6.89 (s, 1H, C=C*H*), 5.16 (s, 4H, OC*H*$_2$Ph); **^{13}C NMR** (101 MHz, $CDCl_3$) δ 170.7, 135.9, 130.7, 130.5, 129.0, 128.6, 128.2, 128.0, 124.0, 112.5, 95.3, 67.0, 33.5; **HRMS** (ESI/QTOF) m/z: $[M + Na]^+$ Calcd for $C_{25}H_{20}NaO_4^+$ 407.1254; Found 407.1256. The NMR spectroscopic data is consistent with previous report[2].

2.1.15. Synthesis and characterization of ethyl 2-hexyl-1-methylcycloprop-2-ene-1-carboxylate (1o)

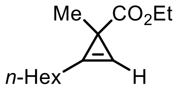

1o

Following **GPA**, oct-1-yne (579 mg, 5.25 mmol, 1.50 equiv.), ethyl diazoalaninate (448 mg, 3.50 mmol, 1.00 equiv.), $Rh_2(OAc)_4$ (7.74 mg, 17.5 μmol, 0.5 mol%) and CH_2Cl_2 (15 mL) were used. Column chromatography on silica gel (eluent: pentane/ethyl acetate = 50:1) afforded **1o** in 42% yield (313 mg, 1.49 mmol) as a colorless oil. **TLC**: R_f (*n*-hexane: EtOAc = 20:1) = 0.34; **^{1}H NMR** (400 MHz, $CDCl_3$) δ 6.38 (td, *J* = 1.4, 0.7 Hz, 1H, C=C*H*), 4.15 – 3.97 (m, 2H, OCH_2CH_3), 2.44 (td, *J* = 7.3, 1.4 Hz, 2H, CH_2CH_2C), 1.59 – 1.47 (m, 2H, CH_2CH_2C), 1.42 – 1.23 (m, 9H, CH_2 & CCH_3), 1.20 (t, *J* = 7.1 Hz, 3H, OCH_2CH_3), 0.97 – 0.77 (m, 3H, CH_3); **^{13}C NMR** (101 MHz, $CDCl_3$) δ 177.8, 121.5, 100.0, 60.3, 31.6, 29.0, 27.0, 24.5, 24.3, 22.6, 20.8, 14.5, 14.1. The NMR spectroscopic data is consistent with previous report[7].

2.1.16. Synthesis and characterization of dimethyl 2-(4-methoxyphenyl)cycloprop-2-ene-1,1-dicarboxylate (1p)

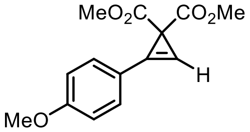

1p

Following **GPA**, 1-ethynyl-4-methoxybenzene (397 mg, 3.00 mmol, 1.00 equiv.), dimethyl 2-diazomalonate (474 mg, 3.00 mmol, 1.00 equiv.), $Rh_2(OAc)_4$ (6.63 mg, 15.0 μmol, 0.500 mol%) and CH_2Cl_2 (10 mL) were used. Column chromatography on silica gel (eluent: pentane/ethyl acetate = 5:1) afforded **1p** in 50% yield (394 mg, 1.50 mmol) as a colorless solid. **TLC**: R_f (*n*-hexane: EtOAc = 4:1) = 0.30; **^{1}H NMR** (400 MHz, $CDCl_3$) δ 7.54 – 7.43 (m, 2H, Ar*H*), 6.95 – 6.85 (m, 2H, Ar*H*), 6.70 (s, 1H, C=C*H*),

4.19 (s, 3H, Ar-OCH_3), 3.83 (s, 3H, CO_2CH_3), 3.82 (s, 3H, CO_2CH_3); **^{13}C NMR** (101 MHz, $CDCl_3$) δ 163.6, 161.5, 159.1, 143.8, 124.5, 122.9, 114.4, 104.6, 93.1, 58.3, 55.5, 51.4; **HRMS** (ESI/QTOF) m/z: [M + Na]$^+$ Calcd for $C_{14}H_{14}NaO_5^+$ 285.0733; Found 285.0737. The NMR spectroscopic data is consistent with previous report[2].

2.1.17. Synthesis and characterization of ethyl 2-hexylcycloprop-2-ene-1-carboxylate (1q)

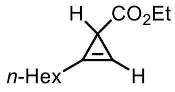

1q

Following **GPA**, oct-1-yne (1.65 g, 15.0 mmol, 1.50 equiv.), ethyl diazoacetate (1.21 mL, 10.0 mmol, 1.00 equiv.), $Rh_2(OAc)_4$ (22.1 mg, 50.0 μmol, 0.5 mol%) and CH_2Cl_2 (20 mL) were used. Column chromatography on silica gel (eluent: pentane/ethyl acetate = 50:1) afforded **1q** in 79% yield (1.55 g, 7.88 mmol) as a colorless oil. **TLC**: R_f (*n*-hexane: EtOAc = 40:1) = 0.23; **^{1}H NMR** (400 MHz, $CDCl_3$) δ 6.32 (q, *J* = 1.5 Hz, 1H, C=C*H*), 4.21 – 4.05 (m, 2H, OCH_2CH_3), 2.49 (td, *J* = 7.3, 1.4 Hz, 2H, CH_2CH_2C), 2.12 (d, *J* = 1.5 Hz, 1H, C*H*CO_2Et), 1.64 – 1.51 (m, 2H, CH_2CH_2C), 1.44 – 1.20 (m, 9H, CH_2 & OCH_2CH_3), 0.96 – 0.82 (m, 3H, CH_3); **^{13}C NMR** (101 MHz, $CDCl_3$) δ 176.8, 115.8, 94.0, 60.3, 31.6, 28.9, 26.8, 25.1, 22.7, 19.9, 14.5, 14.2; **HRMS** (ESI/QTOF) m/z: [M + H]$^+$ Calcd for $C_{12}H_{21}O_2^+$ 197.1536; Found 197.1537. The NMR spectroscopic data is consistent with previous report[8].

2.1.18. Synthesis and characterization of ethyl 2-propylcycloprop-2-ene-1-carboxylate (1r)

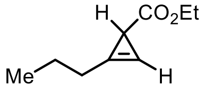

1r

Following **GPA**, pent-1-yne (1.02 g, 15.0 mmol, 1.50 equiv.), ethyl diazoacetate (1.21 mL, 10.0 mmol, 1.00 equiv.), $Rh_2(OAc)_4$ (22.1 mg, 50.0 μmol, 0.500 mol%) and CH_2Cl_2 (10 mL) were used. Column chromatography on silica gel (eluent: pentane/ethyl acetate = 50:1) afforded **1r** in 66% yield (1.01 g, 6.58 mmol) as a colorless oil. **TLC**: R_f (*n*-hexane: EtOAc = 20:1) = 0.36; **^{1}H NMR** (400 MHz, $CDCl_3$) δ 6.33 (q, *J* = 1.4 Hz, 1H, C=C*H*), 4.19 – 4.03 (m, 2H, OCH_2CH_3), 2.46 (td, *J* = 7.2, 1.4 Hz, 2H, CH_2CH_2C), 2.11 (d, *J* = 1.5 Hz, 1H, C*H*CO_2Et), 1.67 – 1.52 (m, 2H, CH_2CH_2C), 1.24 (t, *J* = 7.1 Hz, 3H, OCH_2CH_3), 0.96 (t, *J* = 7.4 Hz, 3H, CH_3); **^{13}C NMR** (101 MHz, $CDCl_3$) δ 176.8, 115.6, 94.2, 60.3, 27.0, 20.2, 19.8, 14.5, 13.8; **HRMS** (ESI/QTOF) m/z: [M + H]$^+$ Calcd for $C_9H_{15}O_2^+$ 155.1067; Found 155.1062. The NMR spectroscopic data is consistent with previous report[9].

2.1.19. Synthesis and characterization of ethyl 2-dodecylcycloprop-2-ene-1-carboxylate (1s)

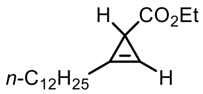

1s

Following **GPA**, tetradec-1-yne (1.94 g, 10.0 mmol, 1.0 equiv.), ethyl diazoacetate (1.21 mL, 10.0 mmol, 1.0 equiv.), $Rh_2(OAc)_4$ (22.1 mg, 50.0 μmol, 0.5 mol%) and CH_2Cl_2 (20 mL) were used. Column chromatography on silica gel (eluent: pentane/ethyl acetate = 50:1) afforded **1s** in 70% yield (1.97 g, 7.03 mmol) as a colorless oil. **TLC**: R_f (*n*-hexane: EtOAc = 20:1) = 0.44; **^{1}H NMR** (400 MHz, $CDCl_3$) δ 6.31 (q, *J* = 1.5 Hz, 1H, C=C*H*), 4.18 – 4.06 (m, 2H, OCH_2CH_3), 2.48 (td, *J* = 7.3, 1.4 Hz, 2H, CH_2CH_2C), 2.12 (d, *J* = 1.5 Hz, 1H, C*H*CO_2Et), 1.61 – 1.54 (m, 2H, CH_2CH_2C), 1.39 – 1.19 (m, 21H, CH_2 & OCH_2CH_3), 0.88 (t, *J* = 6.8 Hz, 3H, CH_3); **^{13}C NMR** (101 MHz, $CDCl_3$) δ 176.8, 115.8, 94.0, 60.3, 32.1, 29.80, 29.78, 29.76, 29.7, 29.5, 29.4, 29.3, 26.8, 25.1, 22.8, 19.9, 14.5, 14.3; **IR** (ν_{max}, cm^{-1}) 2955 (m), 2925 (s), 2855 (s), 1725 (s), 1465 (m), 1370 (w), 1339 (w), 1253 (m), 1183 (s), 1038 (m), 960 (w), 745 (w); **HRMS** (ESI/QTOF) m/z: [M + Na]$^+$ Calcd for $C_{18}H_{32}NaO_2^+$ 303.2295; Found 303.2295.

2.1.20. Synthesis and characterization of ethyl 2-phenethylcycloprop-2-ene-1-carboxylate (1t)

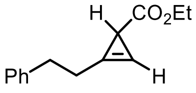

1t

Following **GPA**, but-3-yn-1-ylbenzene (1.95 g, 15.0 mmol, 1.50 equiv.), ethyl diazoacetate (1.21 mL, 10.0 mmol, 1.00 equiv.), $Rh_2(OAc)_4$ (8.84 mg, 20.0 µmol, 0.2 mol%) and CH_2Cl_2 (20 mL) were used. Column chromatography on silica gel (eluent: pentane/ethyl acetate = 50:1) afforded **1t** in 67% yield (1.46 g, 6.74 mmol) as a colorless oil. **TLC**: R_f (*n*-hexane: EtOAc = 20:1) = 0.29; **^{1}H NMR** (400 MHz, $CDCl_3$) δ 7.33 – 7.24 (m, 2H, Ar*H*), 7.24 – 7.16 (m, 3H, Ar*H*), 6.36 (q, *J* = 1.4 Hz, 1H, C=C*H*), 4.20 – 4.04 (m, 2H, OCH_2CH$_3$), 2.95 – 2.88 (m, 2H, CH$_2$CH_2), 2.86 – 2.78 (m, 2H, CH_2CH$_2$), 2.14 (d, *J* = 1.5 Hz, 1H, C*H*CO$_2$Et), 1.25 (t, *J* = 7.1 Hz, 3H, OCH$_2$CH_3); **HRMS** (ESI/QTOF) m/z: [M + Na]$^+$ Calcd for $C_{14}H_{16}NaO_2^+$ 239.1043; Found 239.1047. The NMR spectroscopic data is consistent with previous report[8].

2.1.21. Synthesis and characterization of ethyl [1,1'-bi(cyclopropan)]-3-ene-2-carboxylate (1u)

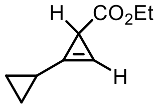

1u

Following **GPA**, ethynylcyclopropane (793 mg, 12.0 mmol, 1.50 equiv.), ethyl diazoacetate (967 µL, 8.00 mmol, 1.00 equiv.), $Rh_2(OAc)_4$ (17.7 mg, 40.0 µmol, 0.500 mol%) and CH_2Cl_2 (10 mL) were used. Column chromatography on silica gel (eluent: pentane/ethyl acetate = 20:1) afforded **1u** in 37% yield (446 mg, 2.93 mmol) as a colorless oil. **TLC**: R_f (*n*-hexane: EtOAc = 40:1) = 0.13; **^{1}H NMR** (400 MHz, $CDCl_3$) δ 6.22 (d, *J* = 1.5 Hz, 1H, C=C*H*), 4.18 – 4.00 (m, 2H, OCH_2CH$_3$), 2.02 (d, *J* = 1.5 Hz, 1H, C*H*CO$_2$Et), 1.83 (tt, *J* = 7.9, 4.7 Hz, 1H, C*H*(cyclopropyl)), 1.22 (t, *J* = 7.1 Hz, 3H, OCH$_2$CH_3), 0.99 – 0.85 (m, 2H, CH_2(cyclopropyl)), 0.85 – 0.77 (m, 1H, CH_2(cyclopropyl)), 0.65 – 0.56 (m, 1H, CH_2(cyclopropyl)); **^{13}C NMR** (101 MHz, $CDCl_3$) δ 176.4, 117.6, 91.8, 60.3, 18.8, 14.5, 7.0, 6.1, 6.0. The NMR spectroscopic data is consistent with previous report[10].

2.1.22. Synthesis and characterization of *tert*-butyl 2-hexylcycloprop-2-ene-1-carboxylate (1v)

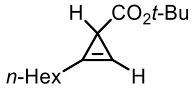

1v

Following **GPA**, oct-1-yne (482 mg, 4.37 mmol, 1.5 equiv.), *tert*-butyl 2-diazoacetate (414 mg, 2.92 mmol, 1.0 equiv.), $Rh_2(OAc)_4$ (6.44 mg, 14.6 µmol, 0.5 mol%) and CH_2Cl_2 (10 mL) were used. Column chromatography on silica gel (eluent: pentane/ethyl acetate = 50:1) afforded **1v** in 53% yield (350 mg, 1.56 mmol) as a colorless oil. **TLC**: R_f (*n*-hexane: EtOAc = 20:1) = 0.41; **^{1}H NMR** (400 MHz, $CDCl_3$) δ 6.29 (q, *J* = 1.5 Hz, 1H, C=C*H*), 2.47 (tt, *J* = 7.2, 1.6 Hz, 2H, CH$_2$CH_2C), 2.02 (d, *J* = 1.6 Hz, 1H, C*H*CO$_2$), 1.61 – 1.53 (m, 2H, CH_2CH$_2$C), 1.43 (s, 9H, C(CH_3)$_3$), 1.40 – 1.25 (m, 6H, CH_2), 0.88 (t, *J* = 6.8 Hz, 3H, CH_3); **^{13}C NMR** (101 MHz, $CDCl_3$) δ 176.2, 116.1, 94.3, 79.7, 31.7, 29.0, 28.3, 26.9, 25.1, 22.7, 20.8, 14.2; **IR** (ν_{max}, cm^{-1}) 2958 (m), 2930 (m), 2860 (w), 1801 (w), 1720 (s), 1458 (w), 1391 (w), 1367 (m), 1346 (m), 1273 (w), 1254 (m), 1213 (m), 1153 (s), 963 (m), 858 (w), 741 (m); **HRMS** (ESI/QTOF) m/z: [M + Na]$^+$ Calcd for $C_{14}H_{24}NaO_2^+$ 247.1669; Found 247.1677.

2.1.23. Synthesis and characterization of adamantan-1-yl 2-hexylcycloprop-2-ene-1-carboxylate (1w)

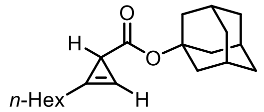

1w

Following **GPA**, oct-1-yne (331 mg, 3.00 mmol, 1.50 equiv.), adamantan-1-yl 2-diazoacetate[7] (459 mg, 2.00 mmol, 1.00 equiv.), $Rh_2(OAc)_4$ (4.40 mg, 10.0 μmol, 0.5 mol%) and CH_2Cl_2 (10 mL) were used. Column chromatography on silica gel (eluent: pentane/ethyl acetate = 50:1) afforded **1w** in 70% yield (421 mg, 1.39 mmol) as a colorless oil. **TLC**: R_f (*n*-hexane: EtOAc = 40:1) = 0.22; **^{1}H NMR** (400 MHz, $CDCl_3$) δ 6.28 (q, *J* = 1.4 Hz, 1H, C=C*H*), 2.46 (tt, *J* = 7.2, 1.6 Hz, 2H, CH_2CH_2C), 2.17 – 2.11 (m, 3H, C*H*(adamantyl)), 2.11 – 2.07 (m, 6H, CH_2(adamantyl)), 2.01 (d, *J* = 1.6 Hz, 1H, $CHCO_2$), 1.69 – 1.60 (m, 6H, CH_2(adamantyl)), 1.59 – 1.52 (m, 2H, CH_2CH_2C), 1.40 – 1.23 (m, 6H, CH_2), 0.88 (t, *J* = 6.8 Hz, 3H, CH_3); **^{13}C NMR** (101 MHz, $CDCl_3$) δ 175.9, 116.1, 94.3, 79.8, 41.6, 36.4, 31.6, 31.0, 28.9, 26.9, 25.1, 22.7, 20.9, 14.2; **IR** (ν_{max}, cm^{-1}) 2954 (w), 2912 (m), 2855 (m), 1800 (w), 1712 (m), 1456 (m), 1346 (m), 1256 (m), 1181 (s), 1103 (w), 1056 (s), 971 (m), 734 (s); **HRMS** (ESI/QTOF) m/z: $[M + Na]^+$ Calcd for $C_{20}H_{30}NaO_2^+$ 325.2138; Found 325.2136.

2.1.24. Synthesis and characterization of (*E*)-3-(4-methoxyphenyl)allyl 2-hexylcycloprop-2-ene-1-carboxylate (1x)

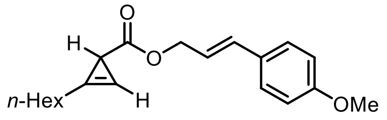

1x

Following **GPA**, oct-1-yne (260 mg, 2.36 mmol, 1.50 equiv.), (*E*)-3-(4-methoxyphenyl)allyl 2-diazoacetate[11] (366 mg, 1.57 mmol, 1.00 equiv.), $Rh_2(OAc)_4$ (3.50 mg, 7.85 μmol, 0.5 mol%) and CH_2Cl_2 (10 mL) were used. Column chromatography on silica gel (eluent: pentane/ethyl acetate = 10:1) afforded **1x** in 65% yield (321 mg, 1.02 mmol) as a colorless oil. **TLC**: R_f (*n*-hexane: EtOAc = 5:1) = 0.49; **^{1}H NMR** (400 MHz, $CDCl_3$) δ 7.37 – 7.29 (m, 2H, Ar*H*), 6.92 – 6.78 (m, 2H, Ar*H*), 6.68 – 6.50 (m, 1H, C=C*H*), 6.34 (q, *J* = 1.4 Hz, 1H, CH_2CH=CH), 6.16 (dt, *J* = 15.8, 6.6 Hz, 1H, CH_2CH=C*H*), 4.71 (ddd, *J* = 6.7, 3.4, 1.3 Hz, 2H, CH_2CH=CH), 3.81 (s, 3H, OCH_3), 2.50 (td, *J* = 7.4, 1.4 Hz, 2H, CH_2CH_2C), 2.17 (d, *J* = 1.6 Hz, 1H, $CHCO_2$), 1.63 – 1.53 (m, 2H, CH_2CH_2C), 1.43 – 1.19 (m, 6H, CH_2), 0.87 (t, *J* = 6.8 Hz, 3H, CH_3); **^{13}C NMR** (101 MHz, $CDCl_3$) δ 176.6, 159.6, 133.7, 129.3, 128.0, 121.5, 115.7, 114.1, 94.0, 65.3, 55.4, 31.6, 28.9, 26.8, 25.1, 22.7, 19.9, 14.2; **IR** (ν_{max}, cm^{-1}) 3144 (w), 2957 (m), 2930 (m), 2859 (m), 1721 (m), 1607 (m), 1512 (s), 1464 (m), 1378 (w), 1337 (w), 1305 (w), 1248 (s), 1169 (s), 1032 (m), 966 (m), 836 (m), 808 (w), 719 (w); **HRMS** (ESI/QTOF) m/z: $[M + Na]^+$ Calcd for $C_{20}H_{26}NaO_3^+$ 337.1774; Found 337.1788.

2.1.25. Synthesis and characterization of (3-(3-(trifluoromethyl)cycloprop-1-en-1-yl)propyl)benzene (1y)[12]

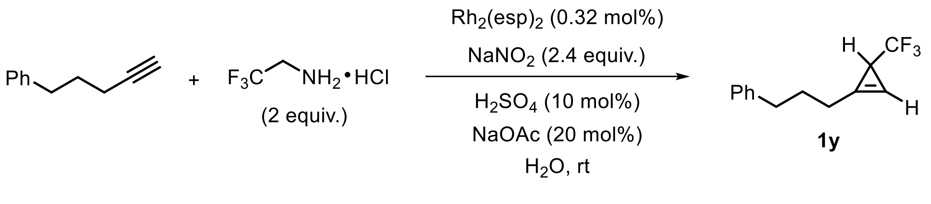

An oven-dried 100 mL Schlenk tube was sequentially charged with a magnetic stir-bar, $Rh_2(esp)_2$ (24.2 mg, 31.7 μmol, 0.32 mol%) and NaOAc (164 mg, 2.00 mmol, 20 mol%). The Schlenk tube was then evacuated and backfilled with nitrogen three times. Subsequently, distilled water (36 mL) was added under nitrogen. To this stirring mixture was added trifluoroethylamine hydrochloride (2.71 g, 20.0

mmol, 2.00 equiv.), H_2SO_4 (53.6 μL, 1.00 mmol, 10 mol%) and pent-4-yn-1-ylbenzene (1.44 g, 10.0 mmol, 1.0 equiv.) sequentially. Then, aqueous $NaNO_2$ (1.66 g, 24.0 mmol, 2.40 equiv.; dissolved in 20 mL of water) was added by syringe pump over 10 hours. After additional 4 hours, CH_2Cl_2 and water were added, and the water layer was extracted with CH_2Cl_2 (3 × 20 mL). The combined organic portions were dried with Na_2SO_4 and evaporated under reduced pressure. The resulting crude residue was purified by column chromatography on silica gel (eluent: pentane) to afford **1y** in 34% yield (774 mg, 3.42 mmol) as a colorless oil. **TLC**: R_f (*n*-hexane: EtOAc = 40:1) = 0.47; **^{1}H NMR** (400 MHz, $CDCl_3$) δ 7.35 – 7.27 (m, 2H, Ar*H*), 7.25 – 7.15 (m, 3H, Ar*H*), 6.43 (hept, *J* = 1.6 Hz, 1H, C=C*H*), 2.70 (dd, *J* = 8.4, 6.8 Hz, 2H, CH_2CH_2C), 2.53 (td, *J* = 7.3, 1.3 Hz, 2H, CH_2CH_2C), 2.01 – 1.92 (m, 3H, C*H*CF_3 & C*H*$_2$); **^{13}C NMR** (101 MHz, $CDCl_3$) δ 141.6, 128.6, 128.6, 126.6 (q, *J* = 275.6 Hz), 126.2, 116.7 (q, *J* = 2.7 Hz), 95.0 (q, *J* = 3.3 Hz), 35.3, 28.4, 24.5, 19.2 (q, *J* = 39.3 Hz); **^{19}F NMR** (377 MHz, $CDCl_3$) δ -67.0; **IR** (ν_{max}, cm^{-1}) 3151 (w), 3029 (w), 2944 (w), 2864 (w), 1497 (w), 1455 (w), 1366 (w), 1275 (s), 1120 (s), 953 (w), 829 (m), 745 (m); **HRMS** (APPI/LTQ-Orbitrap) m/z: $[M]^+$ Calcd for $C_{13}H_{13}F_3^+$ 226.0964; Found 226.0967.

2.2. Synthesis of Hypervalent Iodine Precursors

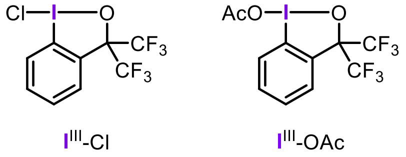

2.2.1. Synthesis and characterization of 1-chloro-3,3-bis(trifluoromethyl)-1,3-dihydro-1λ^3-benzo[*d*][1,2]iodaoxole (I^{III}-Cl)

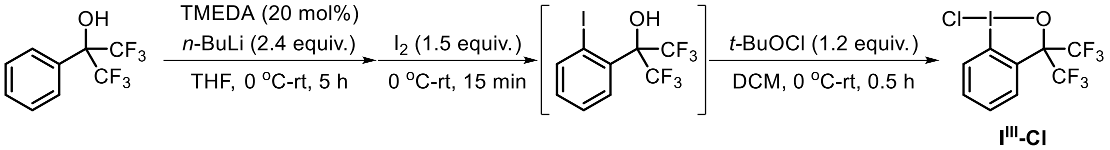

Under nitrogen, TMEDA (1.2 mL, 8.00 mmol, 20.0 mol%) was added to a solution of *n*-BuLi (2.5 M in hexane, 38.4 mL, 96 mmol, 2.40 equiv.). After 15 min, the cloudy solution was cooled to 0 °C and 1,1,1,3,3,3-hexafluoro-2-phenylpropan-2-ol (9.77 g, 40.0 mmol, 1.00 equiv.) in THF (10 mL) was added dropwise. The reaction was stirred at 0 °C for 30 min and then at room temperature for 5 hours. I_2 (15.2 g, 60.0 mmol, 1.50 equiv.) was then added in several portions at 0 °C and the mixture was stirred at 0 °C for 5 min and room temperature for 10 min. The reaction was quenched with saturated NH_4Cl (sat. aq., 10 mL). Et_2O (20 mL) was added and the layers were separated. The aqueous layer was then extracted with Et_2O (10 mL × 2). The organic layers were combined, washed twice with sodium bisulfite solution ($NaHSO_3$, ⩾37% in water; 10 mL), dried over Na_2SO_4, and filtered. The resulting solvent was evaporated under the reduced pressure to afford 1,1,1,3,3,3-hexafluoro-2-(2-iodophenyl)propan-2-ol as an brown oil which was used without further purification. The crude product was dissolved in CH_2Cl_2 (10 mL) under air. *t*-BuOCl (5.21 g, 48.0 mmol, 1.20 equiv.) was then added dropwise at 0 °C. The resulting suspension was stirred under room temperature for 30 min. Then, the reaction mixture was filtered and washed with CH_2Cl_2 (10 mL) and pentane (10 mL) to afford **I^{III}-Cl** in 58% yield (9.38 g, 23.2 mmol) as a yellow solid. **^{1}H NMR** (400 MHz, Acetone-d_6) δ 8.17 (dd, *J* = 8.4, 1.1 Hz, 1H), 8.08 – 8.04 (m, 1H), 7.96 – 7.92 (m, 1H), 7.86 – 7.83 (m, 1H); **^{13}C NMR** (101 MHz, Acetone-d_6) δ 135.2, 133.1, 132.9, 130.5 (m), 130.0, 124.1 (q, *J* = 288.7 Hz), 114.1, 86.2 (m); **^{19}F NMR** (377 MHz, Acetone-d_6) δ -76.5. The NMR spectroscopic data is consistent with previous report[13].

2.2.2. Synthesis and characterization of 3,3-bis(trifluoromethyl)-1λ³-benzo[*d*][1,2]iodaoxol-1(3*H*)-yl acetate (I^{III}-OAc)

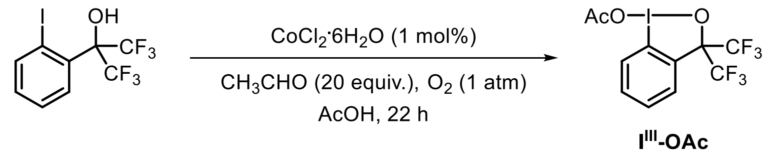

An oven-dried Schlenk tube was charged with a magnetic stir-bar and $CoCl_2\cdot6H_2O$ (32.5 mg, 137 μmol, 1.00 mol%). The Schlenk tube was then evacuated and backfilled with oxygen for three times. After that, 1,1,1,3,3,3-hexafluoro-2-(2-iodophenyl)propan-2-ol (5.05 g, 13.7 mmol, 1.00 equiv. dissolved in 68 mL glacial AcOH) and acetaldehyde (137 mmol, 7.7 mL, 10.0 equiv.) were added by syringe. The reaction mixture was stirred under 1 atm O_2, delivered by inflated balloon at 21 °C for 12 hours. After that, additional acetaldehyde (137 mmol, 7.7 mL, 10.0 equiv.) was added by syringe. The reaction mixture was stirred under 1 atm O_2 and 21 °C for additional 10 hours. The solvent was removed in vacuo and the residue was dissolved in CH_2Cl_2 (20 mL). The organic layer was washed with distilled water (20 mL) and extracted with CH_2Cl_2 (3 × 10 mL). The organic layer was dried over $MgSO_4$ and solvent was removed in vacuo to afford the oily product. Pentane (150 mL) was added gradually to the flask containing the product, which caused a precipitation. Filtration of the resulting suspension afforded the **I^{III}-OAc** as a white solid in 56% yield (3.28 g, 7.67 mmol). **^{1}H NMR** (400 MHz, $CDCl_3$) δ 7.94 – 7.92 (m, 1H), 7.86 – 7.45 (m, 3H), 2.18 (s, 3H); **^{13}C NMR** (101 MHz, $CDCl_3$) δ 176.7, 133.6, 131.7, 131.2, 130.4, 129.8 (m), 123.2 (q, *J* = 289.3 Hz), 115.9, 85.8 (m), 20.6; **^{19}F NMR** (377 MHz, $CDCl_3$) δ -75.9. The NMR spectroscopic data is consistent with previous report[14].

2.3. Synthesis of Cyclopropenyl Benziodoxoles (CpBXs)

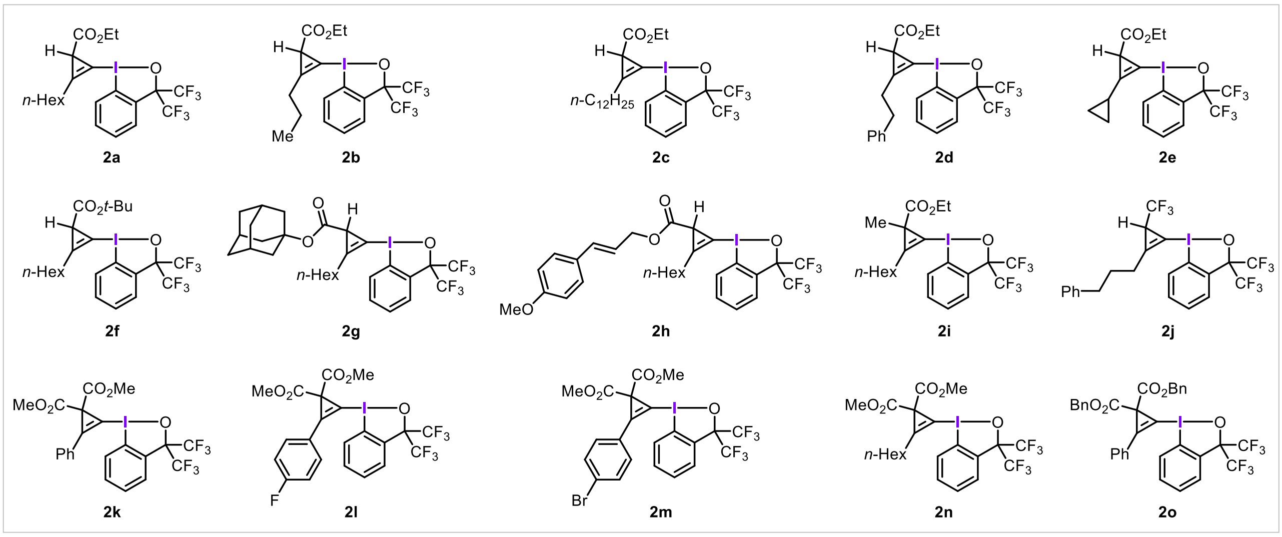

General Procedure B (**GPB**): Synthesis of cyclopropenyl benziodoxoles (CpBXs). **GPB** was applied for the synthesis of **2a**, **2b**, **2c**, **2d**, **2e**, **2f**, **2g**, **2h**, **2k**, **2l** and **2n**.

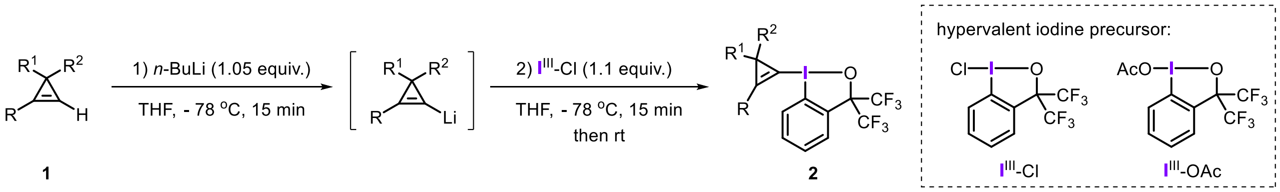

GPB: An oven-dried Schlenk tube was charged with a magnetic stir-bar and terminal cyclopropene **1** (1.00 equiv.). The Schlenk tube was then evacuated and backfilled with nitrogen three times. After that, THF (typically, 0.10 M) was added by syringe and the Schlenk tube was placed at −78 °C in a dry

ice/acetone bath. *n*-Butyllithium (2.5 M in hexane; 1.05 equiv.) was added dropwise by a syringe pump over 5 min and the reaction mixture was stirred at −78 °C for additional 10 min. Then, hypervalent iodine precursor **I**^{III}-Cl or **I**^{III}-OAc (1.10 equiv.) was added in one portion under nitrogen. The reaction mixture was stirred at −78 °C for 15 min, then the cooling bath was removed. The reaction mixture was allowed to warm to room temperature gradually (typically, ca. 15 min) while keeping stirring. The reaction mixture was then quenched by adding saturated aqueous $NaHCO_3$ (10 ml/mmol). The organic phase was removed, and the remaining aqueous portion was extracted with EtOAc. The combined organic portions were dried over Na_2SO_4, filtered and the volatiles removed under reduced pressure. The crude product was purified via flash chromatography on silica gel, and the fractions that contained the product were collected and concentrated by rotary evaporation to afford the purified compound **2**.

General Procedure C (**GPC**): Synthesis of cyclopropenyl benziodoxoles (CpBXs). **GPC** was applied for the synthesis of **2i**, **2j**, **2m** and **2o**.

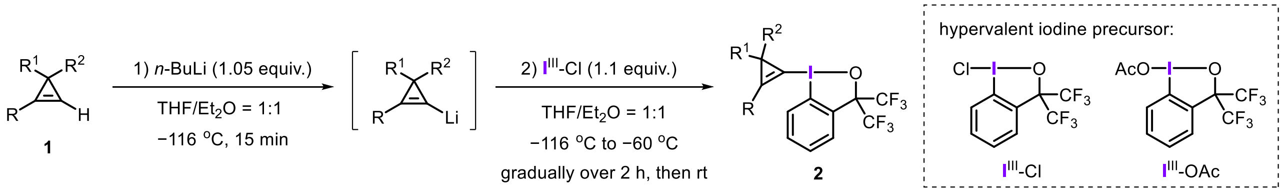

GPC: An oven-dried Schlenk tube was charged with a magnetic stir-bar and terminal cyclopropene **1** (1.00 equiv.). The Schlenk tube was then evacuated and backfilled with nitrogen three times. After that, THF/Et_2O = 1:1 was added by syringe and the Schlenk tube was placed at −116 °C in a liquid nitrogen/ethanol bath. *n*-Butyllithium (2.5 M in hexane; 1.05 equiv.) was added dropwise by a syringe pump over 5 min and the reaction mixture was stirred at −116 °C for additional 10 min. Then, hypervalent iodine precursor **I**^{III}-Cl or **I**^{III}-OAc (1.1 equiv.) was added in one portion under nitrogen. The reaction mixture was stirred in the cooling bath without adding more liquid nitrogen for additional 2 hours, thus resulting a gradual warm-up of the stirring mixture to ca. −60 °C. Then, the cooling bath was removed. The reaction mixture was allowed to warm further to room temperature naturally (typically, ca. 15 min) while keeping stirring. The reaction mixture was then quenched by adding saturated aqueous $NaHCO_3$ (10 ml/mmol). The organic phase was removed, and the remaining aqueous portion was extracted with EtOAc (3 × 10 mL). The combined organic portions were dried over Na_2SO_4, filtered and the volatiles removed under reduced pressure. The crude product was purified via flash chromatography on silica gel, and the fractions that contained the product were collected and concentrated by rotary evaporation to afford the purified compound **2**.

2.3.1. Synthesis and characterization of ethyl 2-(3,3-bis(trifluoromethyl)-1λ^3-benzo[*d*][1,2]iodaoxol-1(3*H*)-yl)-3-hexylcycloprop-2-ene-1-carboxylate (2a)

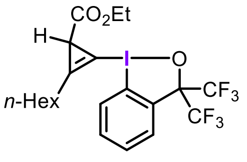

2a

Following **GPB**, **1q** (1.18 g, 6.00 mmol, 1.00 equiv.), *n*-butyllithium (2.5 M in hexane; 2.5 mL, 6.30 mmol, 1.05 equiv.), **I**^{III}-Cl (2.67 g, 6.60 mmol, 1.10 equiv.) and THF (50 mL) were used. Column chromatography on silica gel (eluent: pentane/ethyl acetate = 8:1) afforded **2a** in 77% yield (2.60 g, 4.61 mmol) as a colorless oil, which turned to be solidified when stored in the freezer. **M.p.** 54 – 55 °C. **TLC**: R_f (*n*-hexane: EtOAc = 5:1) = 0.28; ^{1}**H NMR** (400 MHz, $CDCl_3$) δ 7.84 – 7.81 (m, 1H, Ar*H*), 7.71 (dd, *J* = 8.2, 1.2 Hz, 1H, Ar*H*), 7.64 (td, *J* = 7.4, 1.2 Hz, 1H, Ar*H*), 7.57 (ddd, *J* = 8.6, 7.1, 1.6 Hz, 1H, Ar*H*), 4.17 (q, *J* = 7.1 Hz, 2H, OCH_2CH_3), 2.70 (t, *J* = 7.3 Hz, 2H, CH_2CH_2C), 2.64 (s, 1H, C*H*CO_2), 1.73 – 1.57 (m, 2H, CH_2CH_2C), 1.44 – 1.32 (m, 2H, C*H*$_2$), 1.29 – 1.23 (m, 7H, C*H*$_2$ & OCH_2CH_3), 0.94 – 0.76 (m, 3H, C*H*$_3$); ^{13}**C NMR** (101 MHz, $CDCl_3$) δ

174.6, 133.8, 132.8, 131.1, 130.9, 130.3 (hept, *J* = 2.5 Hz), 129.4, 123.8 (q, *J* = 291.6 Hz), 111.7, 81.1 (hept, *J* = 29.2 Hz), 80.4, 61.1, 31.5, 29.0, 27.0, 26.3, 26.1, 22.6, 14.4, 14.0; **^{19}F NMR** (376 MHz, $CDCl_3$) δ -76.1 (m); **IR** (ν_{max}, cm^{-1}) 2960 (w), 2932 (w), 2861 (w), 1809 (w), 1717 (m), 1565 (w), 1465 (w), 1441 (w), 1370 (w), 1337 (w), 1264 (m), 1178 (s), 1150 (s), 1021 (w), 965 (m), 949 (s), 761 (m), 754 (m), 730 (s); **HRMS** (ESI/QTOF) m/z: $[M + H]^+$ Calcd for $C_{21}H_{24}F_6IO_3^+$ 565.0669; Found 565.0686.

2.3.2. Synthesis and characterization of ethyl 2-(3,3-bis(trifluoromethyl)-1λ^3-benzo[*d*][1,2]iodaoxol-1(3*H*)-yl)-3-propylcycloprop-2-ene-1-carboxylate (2b)

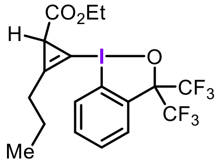

2b

Following **GPB**, **1r** (308 mg, 2.00 mmol, 1.00 equiv.), *n*-butyllithium (2.5 M in hexane; 0.84 mL, 2.10 mmol, 1.05 equiv.), **I**^{III}-OAc (942 mg, 2.20 mmol, 1.10 equiv.) and THF (20 mL) were used. Column chromatography on silica gel (eluent: pentane/ethyl acetate = 4:1) afforded **2b** in 57% yield (595 mg, 1.14 mmol) as a colorless sticky oil. **TLC**: R_f (*n*-hexane: EtOAc = 4:1) = 0.25; **^{1}H NMR** (400 MHz, $CDCl_3$) δ 7.90 – 7.79 (m, 1H, Ar*H*), 7.71 (dd, *J* = 8.1, 1.2 Hz, 1H, Ar*H*), 7.65 (td, *J* = 7.4, 1.2 Hz, 1H, Ar*H*), 7.58 (ddd, *J* = 8.5, 7.2, 1.6 Hz, 1H, Ar*H*), 4.18 (q, *J* = 7.1 Hz, 2H, OCH_2CH_3), 2.69 (t, *J* = 7.2 Hz, 2H, CH_2CH_2C), 1.74 – 1.65 (m, 2H, CH_2CH_2C), 1.27 (t, *J* = 7.1 Hz, 3H, OCH_2CH_3), 1.02 (t, *J* = 7.4 Hz, 3H, CH_3); **^{13}C NMR** (101 MHz, $CDCl_3$) δ 174.5, 133.6, 132.8, 131.0, 130.8, 130.3 (hept, *J* = 2.5 Hz), 129.3, 123.8 (q, *J* = 291.1 Hz), 111.6, 81.1 (hept, *J* = 29.3 Hz), 80.5, 61.1, 28.0, 26.9, 19.8, 14.3, 13.9; **^{19}F NMR** (376 MHz, $CDCl_3$) δ -76.1 (m); **IR** (ν_{max}, cm^{-1}) 3072 (w), 2969 (w), 2938 (w), 2906 (w), 2877 (w), 1810 (w), 1717 (m), 1565 (w), 1464 (w), 1442 (w), 1371 (w), 1335 (w), 1263 (s), 1179 (s), 1150 (s), 1097 (w), 1044 (w), 1019 (m), 964 (m), 949 (s), 866 (w), 802 (w), 757 (m), 731 (m); **HRMS** (ESI/QTOF) m/z: $[M + H]^+$ Calcd for $C_{18}H_{18}F_6IO_3^+$ 523.0199; Found 523.0215.

2.3.3. Synthesis and characterization of ethyl 2-(3,3-bis(trifluoromethyl)-1λ^3-benzo[*d*][1,2]iodaoxol-1(3*H*)-yl)-3-dodecylcycloprop-2-ene-1-carboxylate (2c)

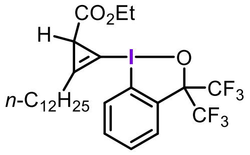

2c

Following **GPB**, **1s** (1.35 g, 4.83 mmol, 1.00 equiv.), *n*-butyllithium (2.5 M in hexane; 2.0 mL, 5.07 mmol, 1.05 equiv.), **I**^{III}-Cl (2.15 g, 5.31 mmol, 1.10 equiv.) and THF (20 mL) were used. Column chromatography on silica gel (eluent: pentane/ethyl acetate = 8:1) afforded **2c** in 73% yield (2.27 g, 3.51 mmol) as a colorless oil, which turned to be solidified when stored in the freezer. **M.p.** 39 – 40 °C. **TLC**: R_f (*n*-hexane: EtOAc = 3:1) = 0.55; **^{1}H NMR** (400 MHz, $CDCl_3$) δ 7.84 (dq, *J* = 7.8, 1.5 Hz, 1H, Ar*H*), 7.72 (dd, *J* = 8.2, 1.2 Hz, 1H, Ar*H*), 7.65 (td, *J* = 7.4, 1.2 Hz, 1H, Ar*H*), 7.58 (ddd, *J* = 8.5, 7.1, 1.6 Hz, 1H, Ar*H*), 4.18 (q, *J* = 7.1 Hz, 2H, OCH_2CH_3), 2.71 (t, *J* = 7.3 Hz, 2H, CH_2CH_2C), 2.64 (s, 1H, $CHCO_2$), 1.72 – 1.58 (m, 2H, CH_2CH_2C), 1.43 – 1.33 (m, 2H, CH_2), 1.33 – 1.14 (m, 19H, CH_2 & OCH_2CH_3), 0.87 (t, *J* = 6.8 Hz, 3H, CH_3); **^{13}C NMR** (101 MHz, $CDCl_3$) δ 174.6, 133.8, 132.9, 131.1, 130.9, 130.4 (m), 129.4, 123.8 (q, *J* = 290.8 Hz), 111.7, 81.2 (hept, *J* = 29.3 Hz), 80.5, 61.2, 32.0, 29.73 (2C), 29.69, 29.6, 29.5, 29.4, 29.3, 27.0, 26.4, 26.2, 22.8, 14.5, 14.2; **^{19}F NMR** (376 MHz, $CDCl_3$) δ -76.1 (m); **IR** (ν_{max}, cm^{-1}) 2928 (m), 2855 (m), 1811 (w), 1721 (m), 1565 (w), 1465 (w), 1442 (w), 1371 (w), 1335 (w), 1264 (s), 1180 (s), 1149 (s), 1020 (w), 964 (m), 950 (s), 867 (w), 757 (m), 727 (m); **HRMS** (ESI/QTOF) m/z: $[M + Na]^+$ Calcd for $C_{27}H_{35}F_6INaO_3^+$ 671.1427; Found 671.1435.

2.3.4. Synthesis and characterization of ethyl 2-(3,3-bis(trifluoromethyl)-1λ³-benzo[*d*][1,2]iodaoxol-1(3*H*)-yl)-3-phenethylcycloprop-2-ene-1-carboxylate (2d)

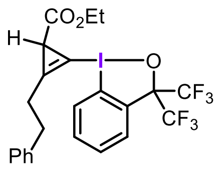

2d

Following **GPB**, **1t** (865 mg, 4.00 mmol, 1.00 equiv.), *n*-butyllithium (2.5 M in hexane; 1.6 mL, 4.00 mmol, 1.00 equiv.), **I**^{III}-Cl (1.62 g, 4.00 mmol, 1.00 equiv.) and THF (24 mL) were used. Column chromatography on silica gel (eluent: pentane/ethyl acetate = 8:1) afforded **2d** in 71% yield (1.66 g, 2.84 mmol) as a colorless sticky oil. **TLC**: R_f (*n*-hexane: EtOAc = 4:1) = 0.26; **^{1}H NMR** (400 MHz, $CDCl_3$) δ 7.83 (d, *J* = 7.6 Hz, 1H, Ar*H*), 7.71 – 7.58 (m, 1H, Ar*H*), 7.58 – 7.44 (m, 2H, Ar*H*), 7.36 – 7.12 (m, 5H, Ar*H*), 4.19 (q, *J* = 7.1 Hz, 2H, OCH_2CH_3), 3.19 – 3.08 (m, 2H, CH_2), 3.08 – 2.95 (m, 2H, CH_2), 2.62 (s, 1H, C*H*CO_2), 1.30 (t, *J* = 7.2 Hz, 3H, OCH_2CH_3); **^{13}C NMR** (101 MHz, $CDCl_3$) δ 174.5, 139.5, 132.8, 132.5, 130.9, 130.7, 130.2 (m), 129.3, 128.9, 128.3, 126.9, 123.8 (q, *J* = 290.8 Hz), 111.6, 81.6, 81.1 (hept, *J* = 29.4 Hz), 61.2, 32.1, 27.3, 26.9, 14.4; **^{19}F NMR** (376 MHz, $CDCl_3$) δ -76.0; **IR** (ν_{max}, cm^{-1}) 3066 (w), 3030 (w), 2982 (w), 2933 (w), 1810 (w), 1715 (m), 1440 (w), 1264 (m), 1178 (s), 1149 (s), 1133 (s), 1021 (m), 964 (m), 949 (s), 866 (w), 795 (w), 753 (m), 730 (s); **HRMS** (ESI/QTOF) m/z: $[M + H]^+$ Calcd for $C_{23}H_{20}F_6IO_3^+$ 585.0356; Found 585.0351.

2.3.5. Synthesis and characterization of ethyl 3-(3,3-bis(trifluoromethyl)-1λ³-benzo[*d*][1,2]iodaoxol-1(3*H*)-yl)-[1,1'-bi(cyclopropan)]-3-ene-2-carboxylate (2e)

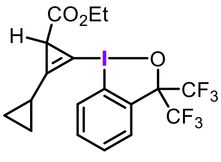

2e

Following **GPB**, **1u** (288 mg, 1.89 mmol, 1.00 equiv.), *n*-butyllithium (2.5 M in hexane; 0.79 mL, 1.98 mmol, 1.05 equiv.), **I**^{III}-OAc (971 mg, 2.27 mmol, 1.20 equiv.) and THF (15 mL) were used. Column chromatography on silica gel (eluent: pentane/ethyl acetate = 2:1) afforded **2e** in 45% yield (439 mg, 845 μmol) as a colorless sticky oil. **TLC**: R_f (*n*-hexane: EtOAc = 4:1) = 0.12; **^{1}H NMR** (400 MHz, $CDCl_3$) δ 7.86 – 7.82 (m, 1H, Ar*H*), 7.75 – 7.70 (m, 1H, Ar*H*), 7.67 – 7.62 (m, 1H, Ar*H*), 7.61 – 7.56 (m, 1H, Ar*H*), 4.17 (q, *J* = 7.1 Hz, 2H, OCH_2CH_3), 2.53 (s, 1H, C*H*CO_2), 2.06 – 1.99 (m, 1H, C*H*(cyclopropyl)), 1.27 (t, *J* = 7.1 Hz, 3H, OCH_2CH_3), 1.22 – 1.09 (m, 2H, CH_2(cyclopropyl)), 1.08 – 0.92 (m, 1H, CH_2(cyclopropyl)), 0.87 – 0.68 (m, 1H, CH_2(cyclopropyl)); **^{13}C NMR** (101 MHz, $CDCl_3$) δ 174.3, 135.8, 132.8, 131.0, 130.9, 130.4 (m), 129.3, 123.9 (q, *J* = 290.8 Hz), 112.0, 81.2(hept, *J* = 29.3 Hz), 76.4, 61.2, 25.4, 14.4, 8.8, 7.9, 7.3; **^{19}F NMR** (376 MHz, $CDCl_3$) δ -76.1 (m); **IR** (ν_{max}, cm^{-1}) 2986 (w), 1813 (m), 1706 (m), 1564 (w), 1463, (w) 1438 (w), 1374 (w), 1334 (s), 1261 (s), 1179 (s), 1149 (w), 1020 (m), 1000 (m), 965 (m), 948 (s), 858 (w), 810 (w), 788 (w), 762 (m), 731 (s); **HRMS** (ESI/QTOF) m/z: $[M + Na]^+$ Calcd for $C_{18}H_{15}F_6INaO_3^+$ 542.9862; Found 542.9881.

2.3.6. Synthesis and characterization of *tert*-butyl 2-(3,3-bis(trifluoromethyl)-1λ³-benzo[*d*][1,2]iodaoxol-1(3*H*)-yl)-3-hexylcycloprop-2-ene-1-carboxylate (2f)

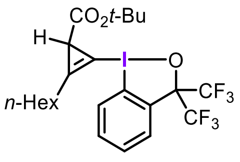

2f

Following **GPB**, **1v** (329 mg, 1.47 mmol, 1.00 equiv.), *n*-butyllithium (2.5 M in hexane; 0.62 mL, 1.54 mmol, 1.05 equiv.), **I**^{III}-Cl (593 mg, 1.47 mmol, 1.00 equiv.) and THF (15 mL) were used. Column chromatography on silica gel (eluent: pentane/ethyl acetate = 8:1) afforded **2f** in 72% yield (627 mg, 1.06 mmol) as a colorless oil. **TLC**: R_f (*n*-hexane: EtOAc = 4:1) = 0.43; **^{1}H NMR** (400 MHz, $CDCl_3$) δ 7.84 (dq, *J* = 7.7, 1.4 Hz, 1H, Ar*H*), 7.71 – 7.60 (m, 2H, Ar*H*), 7.56 (ddd, *J* = 8.5, 7.1, 1.6 Hz, 1H, Ar*H*), 2.70 (td, *J* = 7.2, 1.4 Hz, 2H, CH_2CH_2C), 2.56 (s, 1H, C*H*CO_2), 1.68 – 1.61 (m, 2H, CH_2CH_2C), 1.46 (s, 9H, $C(CH_3)_3$), 1.43 – 1.34 (m, 2H, CH_2), 1.33 – 1.24 (m, 4H, CH_2), 0.93 – 0.79 (m, 3H, CH_3); **^{13}C NMR** (101 MHz, $CDCl_3$) δ 173.8, 134.5, 132.8, 131.1, 130.9, 130.4 (m), 129.5, 123.9 (q, *J* = 290.4 Hz), 111.7, 81.3, 81.1 (hept, *J* = 29.2 Hz), 81.0, 31.5, 29.1, 28.28, 28.25, 26.5, 26.2, 22.6, 14.1; **^{19}F NMR** (376 MHz, $CDCl_3$) δ -76.1 (m); **IR** (ν_{max}, cm^{-1}) 2961 (w), 2934 (w), 2861 (w), 1807 (w), 1713 (m), 1462 (w), 1369 (w), 1265 (m), 1179 (s), 1148 (s), 965 (m), 950 (s), 762 (m); **HRMS** (ESI/QTOF) m/z: $[M + Na]^+$ Calcd for $C_{23}H_{27}F_6INaO_3^+$ 615.0801; Found 615.0810.

2.3.7. Synthesis and characterization of adamantan-1-yl 2-(3,3-bis(trifluoromethyl)-1λ³-benzo[*d*][1,2]iodaoxol-1(3*H*)-yl)-3-hexylcycloprop-2-ene-1-carboxylate (2g)

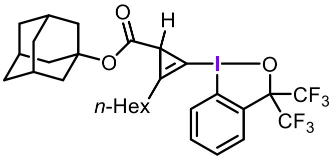

2g

Following **GPB**, **1w** (367 mg, 1.21 mmol, 1.0 equiv.), *n*-butyllithium (2.5 M in hexane; 1.3 mL, 1.27 mmol, 1.05 equiv.), **I**^{III}-Cl (489 mg, 1.21 mmol, 1.00 equiv.) and THF (20 mL) were used. Column chromatography on silica gel (eluent: pentane/ethyl acetate = 8:1) afforded **2g** in 74% yield (598 mg, 892 µmol) as a colorless oil, which turned to be solidified when stored in the freezer. **M.p.** 73 – 75 °C. **TLC**: R_f (*n*-hexane: EtOAc = 4:1) = 0.50; **^{1}H NMR** (400 MHz, $CDCl_3$) δ 7.86 – 7.76 (m, 1H, Ar*H*), 7.66 (dd, *J* = 8.0, 1.2 Hz, 1H, Ar*H*), 7.61 (td, *J* = 7.4, 1.2 Hz, 1H, Ar*H*), 7.54 (ddd, *J* = 8.4, 7.1, 1.5 Hz, 1H, Ar*H*), 2.67 (td, *J* = 7.2, 2.3 Hz, 2H, CH_2CH_2C), 2.53 (s, 1H, C*H*CO_2), 2.17 – 2.10 (m, 3H, C*H*(adamantyl)), 2.10 – 2.04 (m, 6H, C*H$_2$*(adamantyl)), 1.73 – 1.52 (m, 8H, C*H*$_2$ & C*H*$_2$(adamantyl)), 1.46 – 1.31 (m, 2H, CH_2), 1.26 (dt, *J* = 7.5, 3.8 Hz, 4H, CH_2), 0.89 – 0.76 (m, 3H, CH_3); **^{13}C NMR** (101 MHz, $CDCl_3$) δ 173.4, 134.5, 132.8, 131.1, 130.9, 130.4, 129.5, 123.9 (q, *J* = 290.8 Hz), 111.7, 81.4, 81.06, 81.14 (hept, *J* = 29.3 Hz), 41.6, 36.3, 31.5, 31.0, 29.1, 28.3, 26.5, 26.2, 22.6, 14.1; **^{19}F NMR** (376 MHz, $CDCl_3$) δ -76.1 (m); **IR** (ν_{max}, cm^{-1}) 2917 (m), 2857 (w), 1810 (w), 1712 (m), 1457 (w), 1347 (w), 1264 (m), 1179 (s), 1151 (s), 1051 (m), 965 (s), 950 (s), 760 (m), 730 (s); **HRMS** (ESI/QTOF) m/z: $[M + Na]^+$ Calcd for $C_{29}H_{33}F_6INaO_3^+$ 693.1271; Found 693.1271.

2.3.8. Synthesis and characterization of (*E*)-3-(4-methoxyphenyl)allyl 2-(3,3-bis(trifluoromethyl)-1λ³-benzo[*d*][1,2]iodaoxol-1(3*H*)-yl)-3-hexylcycloprop-2-ene-1-carboxylate (2h)

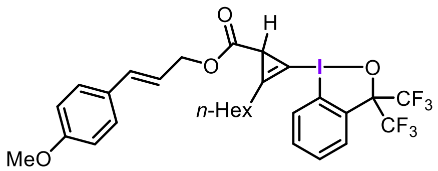

2h

Following **GPB**, **1x** (297 mg, 946 µmol, 1.00 equiv.), *n*-butyllithium (2.5 M in hexane; 0.40 mL, 993 µmol, 1.05 equiv.), **I**^{III}-Cl (383 mg, 946 µmol, 1.00 equiv.) and THF (20 mL) were used. Column chromatography on silica gel (eluent: pentane/ethyl acetate = 5:1) afforded **2h** in 59% yield (382 mg, 559 µmol) as a colorless oil. **TLC**: R_f (*n*-hexane: EtOAc = 4:1) = 0.30; **^{1}H NMR** (400 MHz, $CDCl_3$) δ 7.87 – 7.79 (m, 1H, Ar*H*), 7.72 (dd, *J* = 8.1, 1.2 Hz, 1H, Ar*H*), 7.60 (td, *J* = 7.4, 1.2 Hz, 1H, Ar*H*), 7.53 (ddd, *J* = 8.5, 7.1, 1.6 Hz,

1H, Ar*H*), 7.36 – 7.27 (m, 2H, Ar*H*), 6.90 – 6.80 (m, 2H, Ar*H*), 6.60 (d, *J* = 15.8 Hz, 1H, $CH_2CH{=}CH$), 6.14 (dt, *J* = 15.8, 6.7 Hz, 1H, $CH_2CH{=}CH$), 4.76 (dd, *J* = 6.7, 1.3 Hz, 2H, $CH_2CH{=}CH$), 3.79 (s, 3H, OCH_3), 2.72 (t, *J* = 7.3 Hz, 2H, CH_2CH_2C), 2.69 (s, 1H, $CHCO_2$), 1.81 – 1.56 (m, 2H, CH_2CH_2C), 1.43 – 1.32 (m, 2H, CH_2), 1.32 – 1.20 (m, 4H, CH_2), 0.93 – 0.72 (m, 3H, CH_3); **^{13}C NMR** (101 MHz, $CDCl_3$) δ 174.4, 159.8, 134.5, 133.7, 132.9, 131.0, 130.8, 130.3, 129.4, 128.8, 128.0, 123.8 (q, *J* = 290.9 Hz), 120.6, 114.1, 111.6, 81.1 (hept, *J* = 29.4 Hz), 80.3, 66.0, 55.3, 31.4, 29.0, 27.0, 26.3, 26.1, 22.5, 14.0; **^{19}F NMR** (376 MHz, $CDCl_3$) δ -76.1 (m); **IR** (ν_{max}, cm^{-1}) 2956 (w), 2933 (w), 2860 (w), 1811 (w), 1717 (m), 1608 (w), 1512 (m), 1464 (w), 1441 (w), 1264 (m), 1179 (s), 1150 (s), 1035 (w), 965 (m), 943 (m), 739 (s); **HRMS** (ESI/QTOF) m/z: [M + Na]$^+$ Calcd for $C_{29}H_{33}F_6INaO_3^+$ 693.1271; Found 693.1271.

2.3.9. Synthesis and characterization of ethyl 2-(3,3-bis(trifluoromethyl)- 1λ^3-benzo[*d*][1,2]iodaoxol-1(3*H*)-yl)-3-hexyl-1-methylcycloprop-2-ene-1-carboxylate (2i)

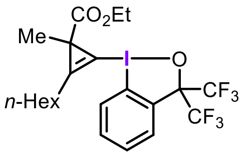

2i

Following **GPC**, **1o** (377 mg, 1.79 mmol, 1.00 equiv.), *n*-butyllithium (2.5 M in hexane; 0.75 mL, 1.88 mmol, 1.05 equiv.), **I**^{III}-Cl (724 mg, 1.79 mmol, 1.00 equiv.), THF (10 mL) and Et_2O (10 mL) were used. Flash column chromatography on silica gel (eluent: pentane/ethyl acetate = 8:1) afforded **2i** in 63% yield (657 mg, 1.14 mmol) as a colorless oil. **TLC**: R_f (*n*-hexane: EtOAc = 5:1) = 0.35; **^{1}H NMR** (400 MHz, $CDCl_3$) δ 7.85 (dq, *J* = 7.7, 1.4 Hz, 1H, Ar*H*), 7.74 (dd, *J* = 8.2, 1.1 Hz, 1H, Ar*H*), 7.65 (ddd, *J* = 7.8, 7.2, 1.1 Hz, 1H, Ar*H*), 7.55 (ddd, *J* = 8.5, 7.2, 1.5 Hz, 1H, Ar*H*), 4.15 (q, *J* = 7.1 Hz, 2H, OCH_2CH_3), 2.67 (t, *J* = 7.3 Hz, 2H, CH_2CH_2C), 1.68 – 1.58 (m, 2H, CH_2CH_2C), 1.50 (s, 3H, $C(CH_3)$), 1.45 – 1.33 (m, 2H, CH_2), 1.33 – 1.20 (m, 7H, CH_2 & OCH_2CH_3), 0.95 – 0.79 (m, 3H, CH_3); **^{13}C NMR** (101 MHz, $CDCl_3$) δ 175.4, 140.9, 132.7, 131.13, 131.06, 130.4 (m), 129.5, 123.9 (q, *J* = 291.3 Hz), 111.4, 87.8, 81.0 (hept, *J* = 29.4 Hz), 61.4, 32.7, 31.5, 29.1, 26.6, 25.6, 22.6, 20.7, 14.5, 14.1; **^{19}F NMR** (376 MHz, $CDCl_3$) δ -76.1 (m); **IR** (ν_{max}, cm^{-1}) 3073 (w), 2959 (w), 2931 (w), 2861 (w), 1800 (w), 1714 (m), 1565 (w), 1465 (w), 1441 (w), 1380 (w), 1260 (s), 1215 (m), 1178 (s), 1151 (s), 1132 (m), 1116 (s), 1025 (w), 965 (m), 950 (s), 795 (w), 759 (m), 730 (s); **HRMS** (ESI/QTOF) m/z: [M + H]$^+$ Calcd for $C_{22}H_{26}F_6IO_3^+$ 579.0825; Found 579.0827.

2.3.10. Synthesis and characterization of 1-(2-(3-phenylpropyl)-3-(trifluoromethyl)cycloprop-1-en-1-yl)-3,3-bis(trifluoromethyl)-1,3-dihydro-1λ^3-benzo[*d*][1,2]iodaoxole (2j)

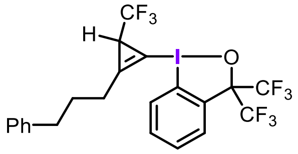

2j

Following **GPC**, **1y** (385 mg, 1.70 mmol, 1.00 equiv.), *n*-butyllithium (2.5 M in hexane; 0.71 mL, 1.79 mmol, 1.05 equiv.), **I**^{III}-Cl (688mg, 1.70 mmol, 1.00 equiv.), THF (10 mL) and Et_2O (10 mL) were used. Flash column chromatography on silica gel (eluent: pentane/ethyl acetate = 10:1) afforded **2j** in 68% yield (682 mg, 1.15 mmol) as a colorless oil, which turned to be solidified when stored in the freezer. **M.p.** 102 – 104 °C. **TLC**: R_f (*n*-hexane: EtOAc = 5:1) = 0.37; **^{1}H NMR** (400 MHz, $CDCl_3$) δ 7.88 (dq, *J* = 7.8, 1.4 Hz, 1H, Ar*H*), 7.68 (td, *J* = 7.5, 1.1 Hz, 1H, Ar*H*), 7.57 (ddd, *J* = 8.5, 7.1, 1.5 Hz, 1H, Ar*H*), 7.39 (dd, *J* = 8.3, 1.0 Hz, 1H, Ar*H*), 7.35 – 7.27 (m, 2H, Ar*H*), 7.25 – 7.19 (m, 1H, Ar*H*), 7.19 – 7.12 (m, 2H, Ar*H*), 2.78 – 2.71 (m, 4H, CH_2), 2.46 (q, *J* = 4.5 Hz, 1H, $CH(CF_3)$), 2.03 (pent, *J* = 7.4 Hz, 2H, CH_2); **^{13}C NMR** (101 MHz, $CDCl_3$) δ 140.7, 132.94, 132.91, 131.3, 130.8, 130.7 (m), 128.8, 128.6, 128.4, 126.5, 125.7 (q, *J* = 275.5 Hz), 123.8 (q, *J* = 290.8 Hz), 111.5, 81.4 (d, *J* = 2.6 Hz), 81.3 (hept, *J* = 29.4 Hz), 35.4, 27.9, 26.4 (q, *J* = 39.6

Hz), 25.6; **^{19}F NMR** (376 MHz, $CDCl_3$) δ -66.5 (CHCF_3), -76.0 (m, C(CF_3)$_2$); **IR** (ν_{max}, cm^{-1}) 3030 (w), 2943 (w), 2864 (w), 1797 (w), 1605 (w), 1497 (w), 1364 (w), 1266 (s), 1217 (m), 1183 (s), 1150 (s), 1131 (s), 965 (s), 952 (s), 829 (w), 754 (m), 730 (s); **HRMS** (ESI/QTOF) m/z: [M + H]$^+$ Calcd for $C_{22}H_{17}F_9IO^+$ 595.0175; Found 595.0184.

2.3.11. Synthesis and characterization of dimethyl 2-(3,3-bis(trifluoromethyl)-1λ^3-benzo[*d*][1,2]iodaoxol-1(3*H*)-yl)-3-phenylcycloprop-2-ene-1,1-dicarboxylate (2k)

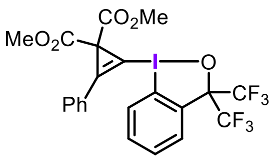

2k

Following **GPB**, **1a** (994 mg, 4.28 mmol, 1.00 equiv.), *n*-butyllithium (2.5 M in hexane; 1.8 mL, 4.49 mmol, 1.05 equiv.), **I**^{III}-Cl (1.73 g, 4.28 mmol, 1.00 equiv.) and THF (40 mL) were used. Column chromatography on silica gel (eluent: pentane/ethyl acetate = 4:1) afforded **2k** in 79% yield (2.02 g, 3.37 mmol) as a colorless solid. **M.p.** 121 – 124 °C. **TLC**: R_f (*n*-hexane: EtOAc = 4:1) = 0.23; **^{1}H NMR** (400 MHz, $CDCl_3$) δ 7.92 – 7.86 (m, 1H, Ar*H*), 7.84 (dd, *J* = 8.4, 1.0 Hz, 1H, Ar*H*), 7.71 – 7.66 (m, 1H, Ar*H*), 7.66 – 7.61 (m, 2H, Ar*H*), 7.60 – 7.46 (m, 4H, Ar*H*), 3.79 (s, 6H, OCH_3); **^{13}C NMR** (101 MHz, $CDCl_3$) δ 170.2, 133.2, 132.3, 131.4, 130.9, 130.8, 130.5 (m), 129.5, 129.4, 126.8, 123.7 (q, *J* = 290.5 Hz), 123.4, 111.8, 81.4 (hept, *J* = 29.6 Hz), 80.1, 53.0, 37.7; **^{19}F NMR** (376 MHz, $CDCl_3$) δ -76.0; **IR** (ν_{max}, cm^{-1}) 3069 (w), 2956 (w), 2847 (w), 1923 (w), 1793 (w), 1729 (m), 1437 (m), 1265 (s), 1181 (s), 1147 (s), 1062 (m), 950 (s), 756 (s), 730 (s); **HRMS** (ESI/QTOF) m/z: [M + Na]$^+$ Calcd for $C_{22}H_{15}F_6INaO_5^+$ 622.9761; Found 622.9778.

2.3.12. Synthesis and characterization of dimethyl 2-(3,3-bis(trifluoromethyl)-1λ^3-benzo[*d*][1,2]iodaoxol-1(3*H*)-yl)-3-(4-fluorophenyl)cycloprop-2-ene-1,1-dicarboxylate (2l)

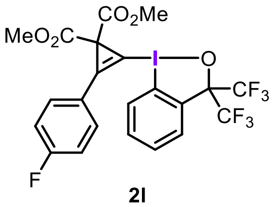

2l

Following **GPB**, **1f** (751 mg, 3.00 mmol, 1.00 equiv.), *n*-butyllithium (2.5 M in hexane; 1.3 mL, 3.15 mmol, 1.05 equiv.), **I**^{III}-Cl (1.21 g, 3.00 mmol, 1.00 equiv.) and THF (15 mL) were used. Column chromatography on silica gel (eluent: pentane/ethyl acetate = 4:1) afforded **2l** in 58% yield (1.08 g, 1.75 mmol) as a colorless oil, which turned to be an amorphous solid when stored in the freezer. **TLC**: R_f (*n*-hexane: EtOAc = 4:1) = 0.17; **^{1}H NMR** (400 MHz, $CDCl_3$) δ 7.90 – 7.85 (m, 1H, Ar*H*), 7.80 (dd, *J* = 8.4, 1.0 Hz, 1H, Ar*H*), 7.72 – 7.60 (m, 3H, Ar*H*), 7.59 – 7.54 (m, 1H, Ar*H*), 7.23 – 7.15 (m, 2H, Ar*H*), 3.79 (s, 6H, OCH_3); **^{13}C NMR** (101 MHz, $CDCl_3$) δ 170.1, 165.0 (d, *J* = 255.7 Hz), 133.2, 133.1 (d, *J* = 9.2 Hz), 131.4, 130.9, 130.6 (m), 129.3, 125.9, 123.7 (q, *J* = 290.5 Hz), 119.8 (d, *J* = 3.3 Hz), 117.0 (d, *J* = 22.4 Hz), 111.7, 81.4 (hept, *J* = 29.7 Hz), 79.7 (d, *J* = 3.2 Hz), 53.1, 37.7; **^{19}F NMR** (376 MHz, $CDCl_3$) δ -75.9 (C(CF_3)$_2$), -104.7 (Ar*F*); **IR** (ν_{max}, cm^{-1}) 3076 (w), 2957 (w), 2849 (w), 1926 (w), 1794 (w), 1732 (s), 1602 (m), 1504 (m), 1465 (w), 1438 (m), 1263 (s), 1237 (s), 1183 (s), 1148 (s), 1065 (m), 1013 (w), 965 (m), 950 (s), 842 (m), 798 (w), 759 (m); **HRMS** (ESI/QTOF) m/z: [M + Na]$^+$ Calcd for $C_{22}H_{14}F_7INaO_5^+$ 640.9666; Found 640.9677.

2.3.13. Synthesis and characterization of dimethyl 2-(3,3-bis(trifluoromethyl)-1λ³-benzo[*d*][1,2]iodaoxol-1(3*H*)-yl)-3-(4-bromophenyl)cycloprop-2-ene-1,1-dicarboxylate (2m)

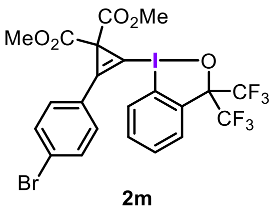

2m

Following **GPC**, **1h** (350 mg, 1.12 mmol, 1.00 equiv.), *n*-butyllithium (2.5 M in hexane; 0.47 mL, 1.18 mmol, 1.05 equiv.), **I**^{III}-Cl (455mg, 1.12 mmol, 1.00 equiv.), THF (10 mL) and Et_2O (10 mL) were used. Flash column chromatography on silica gel (eluent: pentane/ethyl acetate = 5:1) afforded **2m** in 62% yield (475 mg, 699 µmol) as a colorless solid. **M.p.** 150 – 152 °C. **TLC**: R_f (*n*-hexane: EtOAc = 4:1) = 0.20; **^{1}H NMR** (400 MHz, $CDCl_3$) δ 7.88 (d, *J* = 7.7 Hz, 1H, Ar*H*), 7.78 (dd, *J* = 8.3, 1.1 Hz, 1H, Ar*H*), 7.74 – 7.61 (m, 3H, Ar*H*), 7.56 (ddd, *J* = 8.5, 7.1, 1.5 Hz, 1H, Ar*H*), 7.52 – 7.43 (m, 2H, Ar*H*), 3.79 (s, 6H, OCH_3); **^{13}C NMR** (101 MHz, $CDCl_3$) δ 170.0, 133.3, 132.9, 132.0, 131.5, 130.8, 130.6 (m), 129.3, 127.2, 125.9, 123.7 (q, *J* = 290.5 Hz), 122.3, 111.8, 81.4 (hept, *J* = 29.7 Hz), 81.3, 53.1, 37.6; **^{19}F NMR** (376 MHz, $CDCl_3$) δ -75.9; **IR** (ν_{max}, cm^{-1}) 2955 (w), 1797 (w), 1730 (m), 1585 (w), 1482 (m), 1464 (w), 1438 (m), 1398 (w), 1288 (s), 1267 (s), 1184 (s), 1150 (s), 1134 (m), 1068 (m), 1012 (m), 967 (m), 953 (m), 829 (m), 755 (m), 731 (m); **HRMS** (ESI/QTOF) m/z: $[M + Na]^+$ Calcd for $C_{22}H_{14}BrF_6INaO_5^+$ 700.8866; Found 700.8885.

2.3.14. Synthesis and characterization of dimethyl 2-(3,3-bis(trifluoromethyl)-1λ³-benzo[*d*][1,2]iodaoxol-1(3*H*)-yl)-3-hexylcycloprop-2-ene-1,1-dicarboxylate (2n)

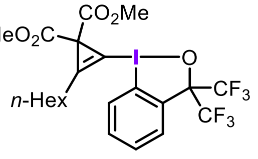

2n

Following **GPB**, **1l** (240 mg, 1.00 mmol, 1.00 equiv.), *n*-butyllithium (2.5 M in hexane; 0.42 mL, 1.05 mmol, 1.05 equiv.), **I**^{III}-Cl (405 g, 1.00 mmol, 1.00 equiv.) and THF (10 mL) were used. Column chromatography on silica gel (eluent: pentane/ethyl acetate = 4:1) afforded **2n** in 78% yield (475 mg, 781 µmol) as a colorless oil. **TLC**: R_f (*n*-hexane: EtOAc = 4:1) = 0.27; **^{1}H NMR** (400 MHz, $CDCl_3$) δ 7.82 – 7.77 (m, 1H, Ar*H*), 7.75 (dd, *J* = 8.2, 1.1 Hz, 1H, Ar*H*), 7.62 (td, *J* = 7.4, 1.1 Hz, 1H, Ar*H*), 7.54 (ddd, *J* = 8.5, 7.2, 1.5 Hz, 1H, Ar*H*), 3.72 (s, 6H, OCH_3), 2.70 (t, *J* = 7.3 Hz, 2H, CH_2CH_2C), 1.61 (pent, *J* = 7.3 Hz, 2H, CH_2CH_2C), 1.44 – 1.28 (m, 2H, CH_2), 1.28 – 1.13 (m, 4H, CH_2), 0.83 – 0.77 (m, 3H, CH_3); **^{13}C NMR** (101 MHz, $CDCl_3$) δ 170.6, 132.9, 131.2, 131.0, 130.8, 130.2 (m), 129.5, 123.6 (q, *J* = 290.6 Hz), 111.4, 81.1 (hept, *J* = 29.5 Hz), 79.1, 52.7, 37.5, 31.3, 28.9, 26.0, 25.1, 22.4, 13.9; **^{19}F NMR** (376 MHz, $CDCl_3$) δ -76.0; **IR** (ν_{max}, cm^{-1}) 2956 (w), 2931 (w), 2859 (w), 1813 (w), 1726 (m), 1464 (w), 1437 (m), 1281 (m), 1265 (s), 1216 (m), 1181 (s), 1149 (s), 1134 (m), 1065 (m), 966 (m), 950 (s), 837 (w), 754 (m), 730 (s); **HRMS** (ESI/QTOF) m/z: $[M + H]^+$ Calcd for $C_{22}H_{24}F_6IO_5^+$ 609.0567; Found 609.0576.

2.3.15. Synthesis and characterization of dibenzyl 2-(3,3-bis(trifluoromethyl)-1λ³-benzo[*d*][1,2]iodaoxol-1(3*H*)-yl)-3-phenylcycloprop-2-ene-1,1-dicarboxylate (2o)

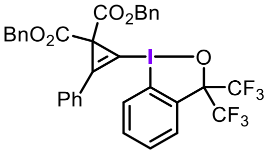

2o

Following **GPC**, **1n** (550 mg, 1.43 mmol, 1.00 equiv.), *n*-butyllithium (2.5 M in hexane; 0.60 mL, 1.50 mmol, 1.05 equiv.), **I**^{III}-Cl (578 mg, 1.43 mmol, 1.00 equiv.), THF (20 mL) and Et_2O (20 mL) were used. Flash column chromatography on silica gel (eluent: pentane/ethyl acetate = 5:1) afforded **2o** in 43% yield (467 mg, 620 µmol) as a colorless oil. **TLC**: R_f (*n*-hexane: EtOAc = 4:1) = 0.33; **^{1}H NMR** (400 MHz, $CDCl_3$) δ 7.86 – 7.77 (m, 1H, Ar*H*), 7.68 (dd, *J* = 8.3, 0.9 Hz, 1H, Ar*H*), 7.59 – 7.52 (m, 3H, Ar*H*), 7.51 – 7.40 (m, 3H, Ar*H*), 7.30 – 7.12 (m, 11H, Ar*H*), 5.26 – 5.09 (m, 4H, OC*H*$_2$Ph); **^{13}C NMR** (101 MHz, $CDCl_3$) δ 169.6, 135.4, 133.2, 132.3, 131.3, 130.8, 130.6, 130.4 (m), 129.4, 129.3, 128.7, 128.5, 128.1, 126.9, 123.7 (q, *J* = 290.6 Hz), 123.3, 111.7, 81.4 (hept, *J* = 29.4 Hz), 80.0, 67.7, 38.1; **^{19}F NMR** (376 MHz, $CDCl_3$) δ -75.9; **IR** (ν_{max}, cm^{-1}) 3068 (w), 3034 (w), 2957 (w), 2891 (w), 2237 (w), 2139 (w), 1959 (w), 1736 (m), 1584 (w), 1498 (w), 1455 (w), 1257 (s), 1213 (s), 1193 (s), 1180 (s), 1147 (m), 1109 (m), 1015 (w), 963 (m), 946 (m), 926 (m), 755 (s), 729 (s); **HRMS** (ESI/QTOF) m/z: [M + Na]$^+$ Calcd for $C_{34}H_{23}F_6INaO_5^+$ 775.0387; Found 775.0394.

3. Optimization of the Synergistic Au/Ag Dual-Catalyzed Cyclopropenyl Cross-Coupling

3.1. Evaluation of Bidentate Ligands

An oven-dried 10 mL Schlenk tube was sequentially charged with a magnetic stirring bar, (Me_2S)AuCl (1.47 mg, 5.00 µmol, 5.00 mol%), the bidentate ligand (25.0 µmol, 25.0 mol%), terminal cyclopropene **1a** (23.2 mg, 100 µmol, 1.00 equiv.) and CpBX **2a** (56.4 mg, 100 µmol, 1.00 equiv.). The Schlenk tube was then introduced into a nitrogen-filled glovebox and $AgNTf_2$ (1.94 mg, 5.00 µmol, 5.00 mol%) was added. The tube was then tightly sealed with a rubber cap and brought out of the glovebox. After that, CH_3CN (0.05 M; 2.0 mL) was added under N_2 atmosphere. The reaction mixture was stirred at 50 °C for the specified time. The resulting reaction mixture was diluted with CH_2Cl_2 (5.0 mL) and filtered through a short pad of silica gel by eluting with CH_2Cl_2 (3 × 5.0 mL). The filtrate was then concentrated to dryness and the residue was subjected to flash column chromatography on silica gel (eluent: pentane/EtOAc = 20:1 to 5:1). The fractions that contained the product **3a** and **4** were collected and concentrated by rotary evaporation. The yields of **3a** [^{1}H NMR δ 2.58 (s, 1H)] and **4** [^{1}H NMR δ 8.09 (dd, *J* = 8.0, 1.4 Hz, 1H)] were obtained by quantitative ^{1}H NMR analysis using CH_2Br_2 [^{1}H NMR δ 4.92 (s, 2H)] as the internal standard.

Table S1. Evaluation of Bidentate Ligands

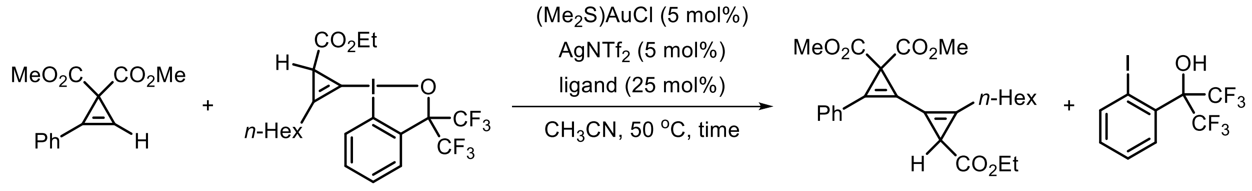

1a 2a 3a 4

Entry	Ligand	Time (h)	Yield or recovery (%)[a]			
			**3a**	**1a**	**2a**	**4**
1	none	9	13	70	40	41
2	**L1**	9	71	14	0	97
3	**L2**	9	52	21	11	76
4	**L3**	9	22	64	52	6
5	**L4**	9	17	66	60	35
6	**L5**	9	35	31	21	31
7	**L6**	9	1	99	89	1
8	**L7**	9	4	90	64	17
9	**L8**	13	46	39	24	53
10	**L9**	9	40	44	34	57
11	**L10**	9	10	88	74	15

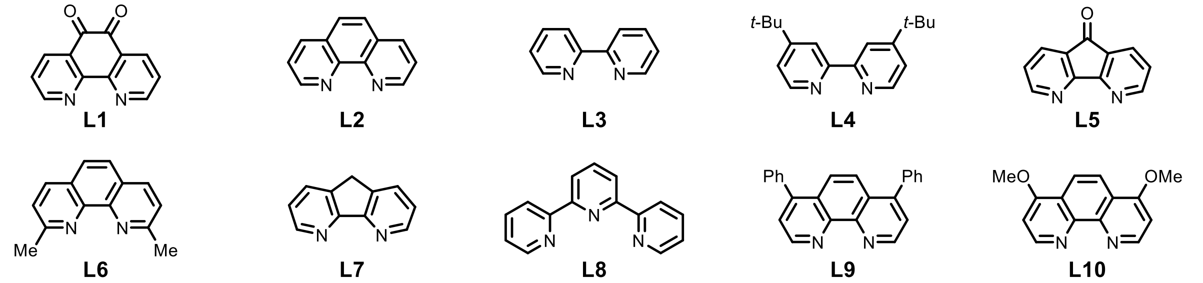

[a]Reaction conditions: **1a** (100 μmol), **2a** (100 μmol), $(Me_2S)AuCl$ (5.00 μmol), $AgNTf_2$ (5.00 μmol), ligand (25.0 μmol), CH_3CN (2.0 mL), 50 °C. Yields or recoveries were determined by ^1H NMR using dibromomethane as the internal standard.

3.2. Evaluation of the Loading of the Silver Catalyst

An oven-dried 10 mL Schlenk tube was sequentially charged with a magnetic stirring bar, $(Me_2S)AuCl$ (1.47 mg, 5.00 μmol, 5.00 mol%), ligand **L1** (5.25 mg, 25.0 μmol, 25.0 mol%), terminal cyclopropene **1a** (23.2 mg, 100 μmol, 1.00 equiv.) and CpBX **2a** (56.4 mg, 100 μmol, 1.00 equiv.). The Schlenk tube was then introduced into a nitrogen-filled glovebox and $AgNTf_2$ (0-15.0 mol%) was added. The tube was then tightly sealed with a rubber cap and brought out of the glovebox. After that, CH_3CN (0.05 M; 2.0 mL) was added under N_2 atmosphere. The reaction mixture was stirred at 50 °C for the specified time. The resulting reaction mixture was diluted with CH_2Cl_2 (5.0 mL) and filtered through a short pad of silica gel by eluting with CH_2Cl_2 (3 × 5.0 mL). The filtrate was then concentrated to dryness and the residue was subjected to flash column chromatography on silica gel (eluent: pentane/EtOAc = 20:1 to 5:1). The fractions that contained the product **3a** [^1H NMR δ 2.58 (s, 1H)] and **4** [^1H NMR δ 8.09 (dd, J = 8.0, 1.4 Hz, 1H)] were collected and concentrated by rotary evaporation. The yields of **3a** and **4** were obtained by quantitative ^1H NMR analysis using CH_2Br_2 [^1H NMR δ 4.92 (s, 2H)] as the internal standard.

Table S2. Evaluation of the Loading of the Silver Catalyst

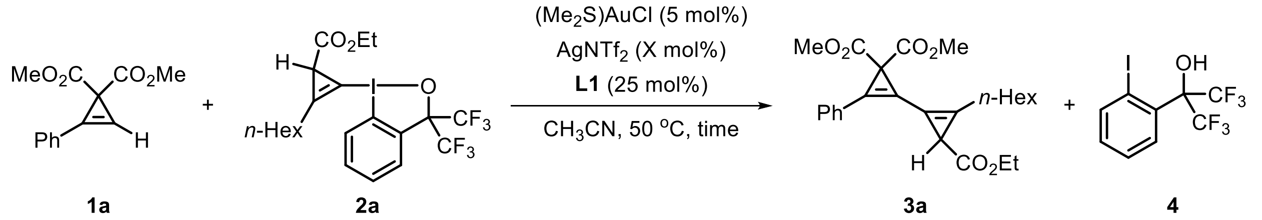

Entry	$AgNTf_2$ (X mol%)	Time (h)	Yield or recovery (%)[a]			
			**3a**	**1a**	**2a**	**4**
1	0	21	1	94	15	58
2	5	9	71	14	0	97
3	10	21	67	14	<1	89
4	15	11	45	42	16	61

[a]Reaction conditions: **1a** (100 µmol), **2a** (100 µmol), $(Me_2S)AuCl$ (5.00 µmol), $AgNTf_2$ (0-15.0 µmol), **L1** (25.0 µmol), CH_3CN (2.0 mL), 50 °C. Yields or recoveries were determined by ^{1}H NMR using dibromomethane as the internal standard.

3.3. Evaluation of the Loading of the Ligand and Reaction Temperature

An oven-dried 10 mL Schlenk tube was sequentially charged with a magnetic stirring bar, $(Me_2S)AuCl$ (1.47 mg, 5.00 µmol, 5.00 mol%), ligand **L1** (15.0-50.0 mol%), terminal cyclopropene **1a** (23.2 mg, 100 µmol, 1.00 equiv.) and CpBX **2a** (73.4 mg, 130 µmol, 1.30 equiv.). The Schlenk tube was then introduced into a nitrogen-filled glovebox and $AgNTf_2$ (1.94 mg, 5.00 µmol, 5.00 mol%) was added. The tube was then tightly sealed with a rubber cap and brought out of the glovebox. After that, CH_3CN (0.05 M; 2.0 mL) was added under N_2 atmosphere. The reaction mixture was stirred at the indicated temperature for the specified time. The resulting reaction mixture was diluted with CH_2Cl_2 (5.0 mL) and filtered through a short pad of silica gel by eluting with CH_2Cl_2 (3 × 5.0 mL). The filtrate was then concentrated to dryness and the residue was subjected to flash column chromatography on silica gel (eluent: pentane/EtOAc = 20:1 to 5:1). The fractions that contained the product **3a** and **4** were collected and concentrated by rotary evaporation. The yields of **3a** [^{1}H NMR δ 2.58 (s, 1H)] and **4** [^{1}H NMR δ 8.09 (dd, *J* = 8.0, 1.4 Hz, 1H)] were obtained by quantitative ^{1}H NMR analysis using CH_2Br_2 [^{1}H NMR δ 4.92 (s, 2H)] as the internal standard. The analysis of the yield of **3a** and the recovery of **1a** are based on the starting **1a**, while the analysis of the yield of **4** and the recovery of **2a** are based on the starting **2a**.

Table S3. Evaluation of the Loading of the Ligand and Reaction Temperature

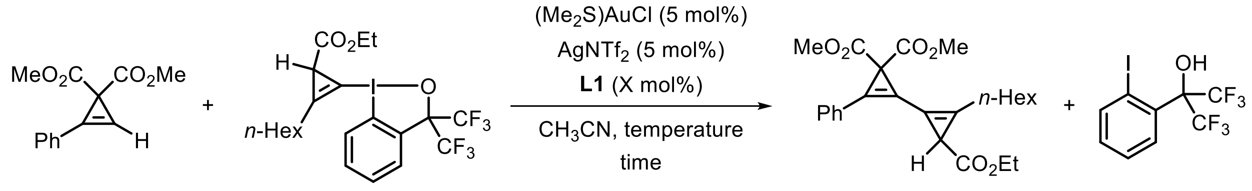

Entry	**L1** (X mol%)	Temperature (°C)	Time (h)	Yield or Recovery (%)[a]			
				**3a**	**1a**	**2a**	**4**
1	50	50	9	80	4	5	91
2	25	50	13	85	1	<1	86
3	15	50	13	77	1	3	92
4[b]	25	50	10	84	4	4	95
5	25	40	10	92	<1	5	92
6	25	30	10	83	7	12	83
7	25	20	10	58	35	34	61

[a]Reaction conditions: **1a** (100 μmol), **2a** (130 μmol), $(Me_2S)AuCl$ (5.00 μmol), $AgNTf_2$ (5.00 μmol), **L1** (15.0-50.0 μmol), CH_3CN (2.0 mL), 20-50 °C. Yields or recoveries were determined by ^1H NMR using dibromomethane as the internal standard. [b]$AgSbF_6$ was used instead of $AgNTf_2$.

3.4. Evaluation of the Silver Salts

An oven-dried 10 mL Schlenk tube was sequentially charged with a magnetic stirring bar, $(Me_2S)AuCl$ (1.47 mg, 5.00 μmol, 5.00 mol%), ligand **L1** (5.25 mg, 25.0 μmol, 25.0 mol%), terminal cyclopropene **1a** (23.2 mg, 100 μmol, 1.00 equiv.) and CpBX **2a** (73.4 mg, 130 μmol, 1.30 equiv.). The Schlenk tube was then introduced into a nitrogen-filled glovebox and the corresponding silver salt (5.00 μmol, 5.00 mol%) was added. The tube was then tightly sealed with a rubber cap and brought out of the glovebox. After that, CH_3CN (0.05 M; 2.0 mL) was added under N_2 atmosphere. The reaction mixture was stirred at 40 °C for the specified time. The resulting reaction mixture was diluted with CH_2Cl_2 (5.0 mL) and filtered through a short pad of silica gel by eluting with CH_2Cl_2 (3 × 5.0 mL). The filtrate was then concentrated to dryness and the residue was subjected to flash column chromatography on silica gel (eluent: pentane/EtOAc = 20:1 to 5:1). The fractions that contained the product **3a** and **4** were collected and concentrated by rotary evaporation. The yields of **3a** [^1H NMR δ 2.58 (s, 1H)] and **4** [^1H NMR δ 8.09 (dd, *J* = 8.0, 1.4 Hz, 1H)] were obtained by quantitative ^1H NMR analysis using CH_2Br_2 [^1H NMR δ 4.92 (s, 2H)] as the internal standard. The analysis of the yields of **3a**, **3k** and the recovery of **1a** are based on the starting **1a**, while the analysis of the yield of **4** and the recovery of **2a** are based on the starting **2a**.

Table S4. Evaluation of the Silver Salts

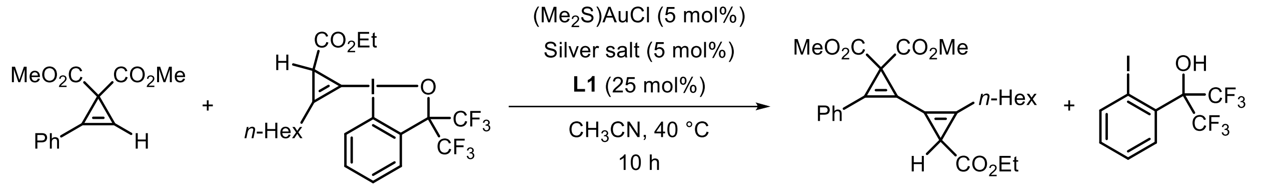

Entry	Silver salt	Yield or recovery (%)[a]			
		**3a**	**1a**	**2a**	**4**
1[b]	AgOAc	43	19	33	67
2	Ag_2CO_3	27	58	66	33
3[c]	AgF	47	18	29	69
4	$AgPF_6$	77	16	22	76
5	$AgNO_3$	79	13	22	78
6	$AgClO_4$	90	5	9	88
7	$AgBF_4$	72	19	19	76
8	CF_3CO_2Ag	29	64	62	35
9	AgOTf	64	30	32	66
10	$AgNTf_2$	92	<1	8	91
11	$AgSbF_6$	91	1	9	90
12[d]	AgCl	61	31	5	95

[a]Reaction conditions: **1a** (100 μmol), **2a** (130 μmol), $(Me_2S)AuCl$ (5.00 μmol), silver salt (5.00 μmol), **L1** (25.0 μmol), CH_3CN (2.0 mL), 40 °C. Yield or recovery was determined by ^1H NMR using dibromomethane as the internal standard. [b]**3k** was observed in 22% ^1H NMR yield. [c]**3k** was observed in 13% ^1H NMR yield. [d]Reaction time: 11 h.

3.5. Variations from the Standard Conditions

An oven-dried 10 mL Schlenk tube was sequentially charged with a magnetic stirring bar, the gold catalyst (5.00 μmol, 5.00 mol%), the bidentate ligand (25.0 μmol, 25.0 mol%), terminal cyclopropene **1a** (23.2 mg, 100 μmol, 1.00 equiv.) and CpBX **2a** (73.4 mg, 130 μmol, 1.30 equiv.). The Schlenk tube was then introduced into a nitrogen-filled glovebox and the silver salt (5.00 μmol, 5.00 mol%) was added. The tube was then tightly sealed with a rubber cap and brought out of the glovebox. After that, CH_3CN (0.05 M; 2.0 mL) was added under N_2 atmosphere. The reaction mixture was stirred at the indicated temperature for the specified time. The resulting reaction mixture was diluted with CH_2Cl_2 (5.0 mL) and filtered through a short pad of silica gel by eluting with CH_2Cl_2 (3 × 5.0 mL). The filtrate was then concentrated to dryness and the residue was subjected to flash column chromatography on silica gel (eluent: pentane/EtOAc = 20:1 to 5:1). The fractions that contained the product **3a**, **4** and the remaining CpBX **2a** or terminal cyclopropene **1a** were collected separately and concentrated by rotary evaporation. The yields of **3a** [^1H NMR δ 2.58 (s, 1H)], **4** [^1H NMR δ 8.09 (dd, *J* = 8.0, 1.4 Hz, 1H)] and the recoveries of **2a** [^1H NMR δ 2.62 (s, 1H)] and **1a** [^1H NMR δ 6.87 (s, 1H)] were obtained by quantitative ^1H NMR analysis using CH_2Br_2 [^1H NMR δ 4.92 (s, 2H)] as the internal standard. The analysis of the yield of **3a**

and the recovery of **1a** are based on the starting **1a**, while the analysis of the yield of **4** and the recovery of **2a** are based on the starting **2a**.

Table S5. Variations from the Standard Conditions

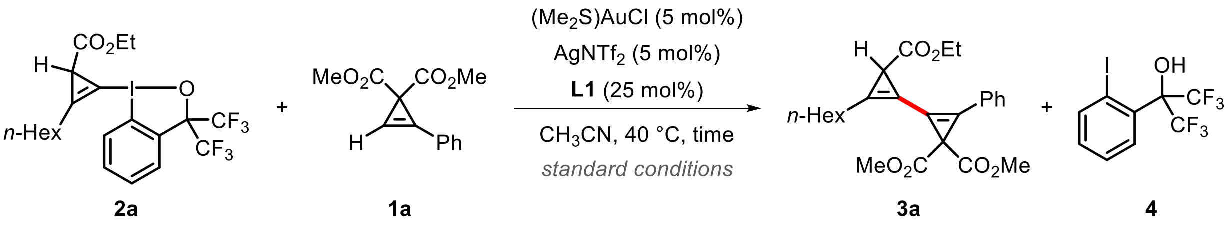

Entry	Variations from the standard conditions	Time (h)	Yield or Recovery/%[a]			
			**3a**	**1a**	**2a**	**4**
1	none	10	92	<1	8	91
2	without $(Me_2S)AuCl$	24	0	99	95	5
3	without $AgNTf_2$	10	3	96	32	61
4	without **L1**	24	15	75	63	37
5	$(Ph_3P)AuCl$ instead of $(Me_2S)AuCl$	24	9	80	78	22
6	15 mol% **L1** was used	10	87	<1	11	89
7	5 mol% **L1** was used	10	42	52	48	50
8	1.0 equiv. **2a** was used	10	81	11	0	99
9	**L2** instead of **L1**	10	56	29	38	62
10	**L3** instead of **L1**	10	21	66	68	32
11	$AgSbF_6$ instead of $AgNTf_2$	10	91	1	9	90
12	AgCl instead of $AgNTf_2$	11	61	31	5	95
13	THF instead of CH_3CN	10	90	1	12	85
14	DCM instead of CH_3CN	10	36	62	58	42
15	50 °C was used	10	85	1	<1	94
16	30 °C was used	10	83	7	12	83
17	20 °C was used	10	58	35	34	61

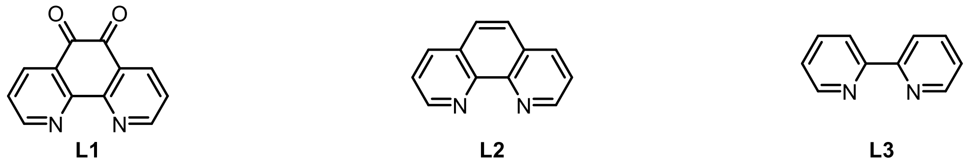

[a]Reaction conditions: **1a** (100 μmol), **2a** (130 μmol), $(Me_2S)AuCl$ (5.00 μmol), $AgNTf_2$ (5.00 μmol), **L1** (25.0 μmol), CH_3CN (2.0 mL), 40 °C. Yields or recoveries were determined by ^1H NMR using dibromomethane as the internal standard.

4. Substrate Scope of Au/Ag Dual-Catalyzed Cyclopropenyl Cross-Coupling

General Procedure D (**GPD**) for the Au/Ag dual-catalyzed cyclopropenyl cross-coupling:

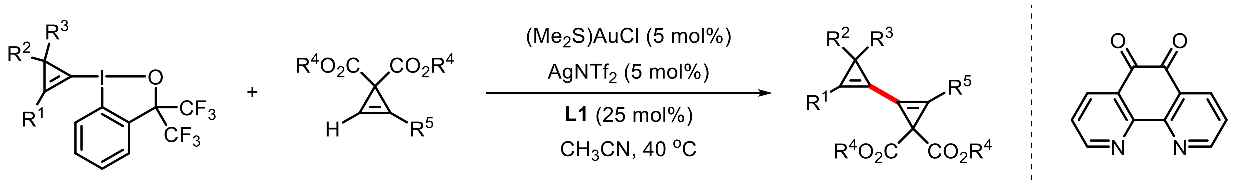

2 **1** **3** **L1**

GPD: An oven-dried 10 mL Schlenk tube was sequentially charged with a magnetic stirring bar, $(Me_2S)AuCl$ (1.47 mg, 5.00 µmol, 5.00 mol%), **L1** (5.25 mg, 25.0 µmol, 25.0 mol%), terminal cyclopropene **1** (100 µmol, 1.00 equiv.) and CpBX **2** (130 µmol, 1.30 equiv.). The Schlenk tube was then introduced into a nitrogen-filled glovebox and $AgNTf_2$ (1.94 mg, 5.00 µmol, 5.00 mol%) was added. The tube was then tightly sealed with a rubber cap and brought out of the glovebox. After that, CH_3CN (0.05 M; 2.0 mL) was added under N_2 atmosphere. The reaction mixture was stirred at 40 °C until complete consumption of the terminal cyclopropene as observed by TLC analysis, or until no further reaction progress could be seen. The reaction mixture was then filtered through a silica gel pad and washed with CH_2Cl_2 (3 × 5.0 mL). Excess solvent was removed under reduced pressure and the desired product **3** was obtained by column chromatography on silica gel. The by-product **4** is a slightly volatile colorless liquid. Thus, the fractions that contained **4** were collected and concentrated by rotary evaporation (vacuum pressure higher than 100 mBar, 40 °C water bath). The yield of **4**[15] was determined by quantitative ^1H NMR analysis of the collected residue using CH_2Br_2 (^1H NMR δ 4.92) as the internal standard.

4.1. Substrate Scope of Cyclopropenyl Benziodoxoles (CpBXs)

4.1.1. Synthesis and characterization of 2'-ethyl 2,2-dimethyl 3'-hexyl-3-phenyl-[1,1'-bi(cyclopropane)]-3,3'-diene-2,2,2'-tricarboxylate (3a)

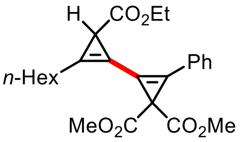

3a

Following **GPD**, a mixture of $(Me_2S)AuCl$ (1.47 mg, 5.00 µmol, 5.00 mol%), $AgNTf_2$ (1.94 mg, 5.00 µmol, 5.00 mol%), **L1** (5.25 mg, 25.0 µmol, 25.0 mol%), CpBX **2a** (73.4 mg, 130 µmol, 1.30 equiv.), terminal cyclopropene **1a** (23.2 mg, 100 µmol, 1.00 equiv.) and CH_3CN (2.0 mL) was stirred at 40 °C for 10 hours. Flash column chromatography on silica gel (eluent: pentane/ethyl acetate = 6:1) afforded **3a** in 88% yield (37.4 mg, 87.7 µmol) as a colorless oil and **4** in 91% NMR yield. **TLC**: R_f (*n*-hexane/EtOAc = 5:1) = 0.31; **^{1}H NMR** (400 MHz, $CDCl_3$) δ 7.64 – 7.58 (m, 2H, Ar*H*), 7.47 – 7.39 (m, 3H, Ar*H*), 4.16 (q, *J* = 7.1 Hz, 2H, $CO_2CH_2CH_3$), 3.73 (s, 3H, CO_2CH_3), 3.72 (s, 3H, CO_2CH_3), 2.72 – 2.63 (m, 2H, CH_2CH_2C), 2.59 (s, 1H, $CHCO_2$), 1.74 – 1.66 (m, 2H, CH_2CH_2C), 1.51 – 1.14 (m, 9H, CH_2 & $CO_2CH_2CH_3$), 1.03 – 0.75 (m, 3H, CH_3); **^{13}C NMR** (101 MHz, $CDCl_3$) δ 174.2, 170.1, 169.9, 130.6, 130.5, 129.0, 124.6, 119.4, 109.0, 94.7, 92.6, 60.5, 52.5, 52.4, 35.3, 31.5, 28.8, 26.8, 26.1, 23.8, 22.5, 14.4, 14.1; **IR** (ν_{max}, cm^{-1}) 2932 (w), 2862 (w), 1807 (m), 1723 (w), 1442 (w), 1392 (m), 1372 (m), 1312 (s), 1266 (s), 1183 (s), 1153 (w), 1065 (w), 1022 (w), 951 (w), 762 (w), 732 (w); **HRMS** (ESI/QTOF) m/z: $[M + Na]^+$ Calcd for $C_{25}H_{30}NaO_6^+$ 449.1935; Found 449.1931.

4.1.2. Synthesis and characterization of 2'-ethyl 2,2-dimethyl 3-phenyl-3'-propyl-[1,1'-bi(cyclopropane)]-3,3'-diene-2,2,2'-tricarboxylate (3b)

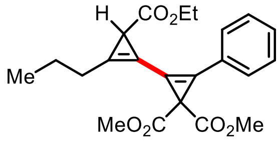

3b

Following **GPD**, a mixture of (Me_2S)AuCl (1.47 mg, 5.00 µmol, 5.00 mol%), $AgNTf_2$ (1.94 mg, 5.00 µmol, 5.00 mol%), **L1** (5.25 mg, 25.0 µmol, 25.0 mol%), CpBX **2b** (67.9 mg, 130 µmol, 1.30 equiv.), terminal cyclopropene **1a** (23.2 mg, 100 µmol, 1.00 equiv.) and CH_3CN (2.0 mL) was stirred at 40 °C for 18 hours. Flash column chromatography on silica gel (eluent: pentane/ethyl acetate = 4:1) afforded **3b** in 82% yield (31.4 mg, 81.7 µmol) as a colorless oil and **4** in 97% NMR yield. **TLC**: R_f (*n*-hexane/EtOAc = 4:1) = 0.28; **^{1}H NMR** (400 MHz, $CDCl_3$) δ 7.66 – 7.55 (m, 2H, Ar*H*), 7.45 – 7.40 (m, 3H, Ar*H*), 4.19 – 4.12 (m, 2H, $CO_2CH_2CH_3$), 3.72 (s, 3H, CO_2CH_3), 3.71 (s, 3H, CO_2CH_3), 2.67 – 2.63 (m, 2H, CH_2CH_2C), 2.59 (s, 1H, C*H*CO_2), 1.78 – 1.69 (m, 2H, CH_2CH_2C), 1.24 (t, *J* = 7.1 Hz, 3H, $CO_2CH_2CH_3$), 1.02 (t, *J* = 7.4 Hz, 3H, CH_3); **^{13}C NMR** (101 MHz, $CDCl_3$) δ 174.3, 170.2, 170.0, 130.8, 130.6, 129.1, 124.6, 119.2, 109.0, 94.8, 92.9, 60.6, 52.6, 52.5, 35.4, 28.2, 23.9, 20.4, 14.4, 13.8; **IR** (ν_{max}, cm^{-1}) 2959 (w), 2936 (w), 2875 (w), 1894 (w), 1804 (w), 1729 (s), 1490 (w), 1436 (m), 1370 (w), 1334 (w), 1285 (s), 1247 (s), 1185 (s), 1064 (s), 1032 (w), 1005 (w), 765 (m); **HRMS** (ESI/QTOF) m/z: $[M + Na]^+$ Calcd for $C_{22}H_{24}NaO_6^+$ 407.1465; Found 407.1464.

4.1.3. Synthesis and characterization of 2'-ethyl 2,2-dimethyl 3'-dodecyl-3-phenyl-[1,1'-bi(cyclopropane)]-3,3'-diene-2,2,2'-tricarboxylate (3c)

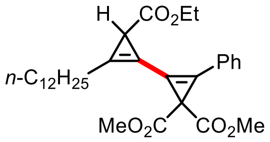

3c

Following **GPD**, a mixture of (Me_2S)AuCl (1.47 mg, 5.00 µmol, 5.00 mol%), $AgNTf_2$ (1.94 mg, 5.00 µmol, 5.00 mol%), **L1** (5.25 mg, 25.0 µmol, 25.0 mol%), CpBX **2c** (84.3 mg, 130 µmol, 1.30 equiv.), terminal cyclopropene **1a** (23.2 mg, 100 µmol, 1.00 equiv.) and CH_3CN (2.0 mL) was stirred at 40 °C for 18 hours. Flash column chromatography on silica gel (eluent: pentane/ethyl acetate = 6:1) afforded **3c** in 91% yield (46.7 mg, 91.4 µmol) as a colorless oil and **4** in 95% NMR yield. **TLC**: R_f (*n*-hexane/EtOAc = 5:1) = 0.36; **^{1}H NMR** (400 MHz, $CDCl_3$) δ 7.65 – 7.56 (m, 2H, Ar*H*), 7.48 – 7.38 (m, 3H, Ar*H*), 4.21 – 4.10 (m, 2H, $CO_2CH_2CH_3$), 3.721 (s, 3H, CO_2CH_3), 3.715 (s, 3H, CO_2CH_3), 2.72 – 2.63 (m, 2H, CH_2CH_2C), 2.58 (s, 1H, C*H*CO_2), 1.77 – 1.62 (m, 2H, CH_2CH_2C), 1.49 – 1.10 (m, 21H, CH_2 & $CO_2CH_2CH_3$), 0.87 (t, *J* = 6.8 Hz, 3H, CH_3); **^{13}C NMR** (101 MHz, $CDCl_3$) δ 174.2, 170.2, 170.0, 130.7, 130.6, 129.1, 124.7, 119.6, 109.2, 94.8, 92.8, 60.6, 52.54, 52.53, 35.4, 32.0, 29.78 (2C), 29.75, 29.6, 29.5, 29.4, 29.3, 27.0, 26.2, 23.9, 22.8, 14.5, 14.2; **IR** (ν_{max}, cm^{-1}) 2950 (w), 2925 (m), 2854 (m), 1894 (w), 1804 (w), 1728 (s), 1490 (w), 1461 (w), 1436 (m), 1369 (w), 1332 (w), 1281 (s), 1243 (s), 1182 (s), 1063 (s), 1004 (w), 917 (w), 835 (w), 763 (m), 733 (m); **HRMS** (ESI/QTOF) m/z: $[M + Na]^+$ Calcd for $C_{31}H_{42}NaO_6^+$ 533.2874; Found 533.2872.

4.1.4. Synthesis and characterization of 2'-ethyl 2,2-dimethyl 3'-phenethyl-3-phenyl-[1,1'-bi(cyclopropane)]-3,3'-diene-2,2,2'-tricarboxylate (3d)

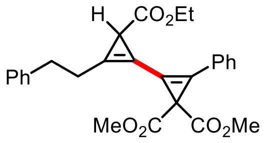

3d

Following **GPD**, a mixture of $(Me_2S)AuCl$ (1.47 mg, 5.00 µmol, 5.00 mol%), $AgNTf_2$ (1.94 mg, 5.00 µmol, 5.00 mol%), **L1** (5.25 mg, 25.0 µmol, 25.0 mol%), CpBX **2d** (76.0 mg, 130 µmol, 1.30 equiv.), terminal cyclopropene **1a** (23.2 mg, 100 µmol, 1.00 equiv.) and CH_3CN (2.0 mL) was stirred at 40 °C for 17 hours. Flash column chromatography on silica gel (eluent: pentane/ethyl acetate = 4:1) afforded **3d** in 85% yield (38.1 mg, 85.3 µmol) as a colorless oil and **4** in 88% NMR yield. **TLC**: R_f (*n*-hexane/EtOAc = 4:1) = 0.23; **^{1}H NMR** (400 MHz, $CDCl_3$) δ 7.67 – 7.58 (m, 2H, Ar*H*), 7.49 – 7.43 (m, 3H, Ar*H*), 7.36 – 7.30 (m, 2H, Ar*H*), 7.30 – 7.22 (m, 3H, Ar*H*), 4.22 – 4.15 (m, 2H, $CO_2CH_2CH_3$), 3.75 (s, 3H, CO_2CH_3), 3.74 (s, 3H, CO_2CH_3), 3.17 – 2.96 (m, 4H, CH_2CH_2), 2.64 (s, 1H, $CHCO_2$), 1.27 (t, J = 7.1 Hz, 3H, $CO_2CH_2CH_3$); **^{13}C NMR** (101 MHz, $CDCl_3$) δ 174.1, 170.1, 170.0, 140.5, 130.8, 130.7, 129.1, 128.6, 128.3, 126.4, 124.5, 118.5, 109.8, 94.6, 93.5, 60.7, 52.6 (2C), 35.5, 33.0, 27.8, 24.1, 14.4; **IR** (ν_{max}, cm^{-1}) 3061 (w), 3029 (w), 2980 (w), 2954 (w), 2851 (w), 1892 (w), 1804 (w), 1725 (s), 1603 (w), 1494 (w), 1449 (m), 1435 (m), 1369 (w), 1335 (w), 1284 (s), 1244 (s), 1184 (s), 1062 (s), 1027 (m), 979 (w), 921 (w), 763 (m); **HRMS** (APCI/QTOF) m/z: $[M + Na]^+$ Calcd for $C_{27}H_{26}NaO_6^+$ 469.1622; Found 469.1623.

4.1.5. Synthesis and characterization of 3'-ethyl 2,2-dimethyl 3-phenyl-[1,1':2',1''-tercyclopropane]-1',3-diene-2,2,3'-tricarboxylate (3e)

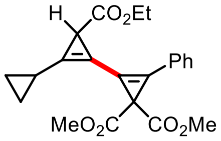

3e

Following **GPD**, a mixture of $(Me_2S)AuCl$ (1.47 mg, 5.00 µmol, 5.00 mol%), $AgNTf_2$ (1.94 mg, 5.00 µmol, 5.00 mol%), **L1** (5.25 mg, 25.0 µmol, 25.0 mol%), CpBX **2e** (67.6 mg, 130 µmol, 1.30 equiv.), terminal cyclopropene **1a** (23.2 mg, 100 µmol, 1.00 equiv.) and CH_3CN (2.0 mL) was stirred at 40 °C for 18 hours. Flash column chromatography on silica gel (eluent: pentane/ethyl acetate = 3:1) afforded **3e** in 45% yield (17.1 mg, 44.7 µmol) as a colorless oil and **4** in 97% NMR yield. **TLC**: R_f (*n*-hexane/EtOAc = 3:1) = 0.26; **^{1}H NMR** (400 MHz, $CDCl_3$) δ 7.64 – 7.55 (m, 2H, Ar*H*), 7.51 – 7.35 (m, 3H, Ar*H*), 4.22 – 4.10 (m, 2H, $CO_2CH_2CH_3$), 3.74 (s, 3H, CO_2CH_3), 3.73 (s, 3H, CO_2CH_3), 2.52 (s, 1H, $CHCO_2$), 2.08 – 2.02 (m, 1H, C*H*(cyclopropyl)), 1.24 (t, J = 7.1 Hz, 3H, $CO_2CH_2CH_3$), 1.16 – 1.11 (m, 2H, CH_2(cyclopropyl)), 1.06 – 1.00 (m, 1H, CH_2(cyclopropyl)), 0.93 – 0.82 (m, 1H, CH_2(cyclopropyl)); **^{13}C NMR** (101 MHz, $CDCl_3$) δ 174.0, 170.3, 170.2, 130.6, 130.5, 129.1, 124.8, 122.2, 107.9, 94.7, 89.8, 60.7, 52.6 (2C), 35.5, 23.0, 14.5, 9.4, 8.8, 7.7; **IR** (ν_{max}, cm^{-1}) 2978 (w), 2956 (w), 2930 (w), 1889 (w), 1805 (w), 1728 (s), 1490 (w), 1436 (m), 1369 (w), 1334 (w), 1284 (m), 1249 (s), 1186 (m), 1063 (m), 1028 (w), 940 (w), 875 (w), 765 (m); **HRMS** (ESI/QTOF) m/z: $[M + Na]^+$ Calcd for $C_{22}H_{22}NaO_6^+$ 405.1309; Found 405.1314.

4.1.6. Synthesis and characterization of 2'-(*tert*-butyl) 2,2-dimethyl 3'-hexyl-3-phenyl-[1,1'-bi(cyclopropane)]-3,3'-diene-2,2,2'-tricarboxylate (3f)

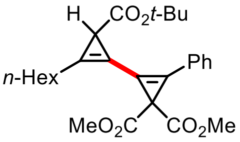

3f

Following **GPD**, a mixture of $(Me_2S)AuCl$ (1.47 mg, 5.00 µmol, 5.00 mol%), $AgNTf_2$ (1.94 mg, 5.00 µmol, 5.00 mol%), **L1** (5.25 mg, 25.0 µmol, 25.0 mol%), CpBX **2f** (61.2 mg, 103 µmol, 1.03 equiv.), terminal cyclopropene **1a** (23.2 mg, 100 µmol, 1.00 equiv.) and CH_3CN (2.0 mL) was stirred at 40 °C for 24 hours. Flash column chromatography on silica gel (eluent: pentane/ethyl acetate = 5:1) afforded **3f** in 55% yield (24.8 mg, 54.6 µmol) as a colorless oil and **4** in 92% NMR yield. **TLC**: R_f (*n*-hexane/EtOAc = 4:1) = 0.54; **^{1}H NMR** (400 MHz, $CDCl_3$) δ 7.65 – 7.55 (m, 2H, Ar*H*), 7.47 – 7.36 (m, 3H, Ar*H*), 3.72 (s, 3H, CO_2CH_3),

3.71 (s, 3H, CO_2CH_3), 2.66 (t, *J* = 7.2 Hz, 2H, CH_2CH_2C), 2.49 (s, 1H, $CHCO_2$), 1.72 – 1.65 (m, 2H, CH_2CH_2C), 1.51 – 1.21 (m, 15H, CH_2 & $C(CH_3)_3$), 0.94 – 0.81 (m, 3H, CH_3); **^{13}C NMR** (101 MHz, $CDCl_3$) δ 173.6, 170.3, 170.0, 130.6, 130.5, 129.1, 124.8, 119.8, 108.7, 95.1, 93.1, 80.3, 52.6, 52.5, 35.5, 31.6, 28.9, 28.2, 27.0, 26.2, 25.0, 22.6, 14.2; **IR** (ν_{max}, cm^{-1}) 2956 (m), 2932 (m), 2860 (w), 1894 (w), 1804 (w), 1730 (s), 1600 (w), 1451 (m), 1436 (m), 1368 (m), 1284 (s), 1248 (s), 1216 (m), 1154 (s), 1064 (s), 1005 (w), 957 (w), 852 (w), 763 (m); **HRMS** (ESI/QTOF) m/z: $[M + Na]^+$ Calcd for $C_{27}H_{34}NaO_6^+$ 477.2248; Found 477.2263.

4.1.7. Synthesis and characterization of 2'-(adamantan-1-yl) 2,2-dimethyl 3'-hexyl-3-phenyl-[1,1'-bi(cyclopropane)]-3,3'-diene-2,2,2'-tricarboxylate (3g)

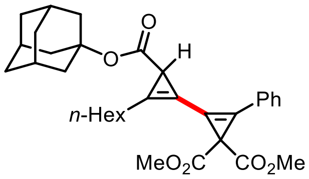

3g

Following **GPD**, a mixture of $(Me_2S)AuCl$ (1.47 mg, 5.00 μmol, 5.00 mol%), $AgNTf_2$ (1.94 mg, 5.00 μmol, 5.00 mol%), **L1** (5.25 mg, 25.0 μmol, 25.0 mol%), CpBX **2g** (87.2 mg, 130 μmol, 1.30 equiv.), terminal cyclopropene **1a** (23.2 mg, 100 μmol, 1.00 equiv.) and CH_3CN (2.0 mL) was stirred at 40 °C for 24 hours. Flash column chromatography on silica gel (eluent: pentane/ethyl acetate = 8:1) afforded **3g** in 56% yield (30.0 mg, 56.3 μmol) as a colorless oil and **4** in 92% NMR yield. **TLC**: R_f (*n*-hexane/EtOAc = 5:1) = 0.41; **^{1}H NMR** (400 MHz, $CDCl_3$) δ 7.65 – 7.57 (m, 2H, Ar*H*), 7.48 – 7.39 (m, 3H, Ar*H*), 3.724 (s, 3H, CO_2CH_3), 3.716 (s, 3H, CO_2CH_3), 2.66 (t, *J* = 7.2 Hz, 2H, CH_2CH_2C), 2.48 (s, 1H, $CHCO_2$), 2.17 – 2.11 (m, 3H, C*H*(adamantyl)), 2.10 – 2.07 (m, 6H, CH_2(adamantyl)), 1.71 – 1.66 (m, 2H, CH_2CH_2C), 1.65 – 1.61 (m, 6H, CH_2(adamantyl)), 1.45 – 1.23 (m, 6H, CH_2), 0.96 – 0.83 (m, 3H, CH_3); **^{13}C NMR** (101 MHz, $CDCl_3$) δ 173.4, 170.3, 170.1, 130.63, 130.60, 129.1, 124.8, 119.8, 108.6, 95.1, 93.1, 80.4, 52.6, 52.5, 41.5, 36.3, 35.5, 31.6, 30.9, 29.0, 27.0, 26.3, 25.0, 22.6, 14.2; **IR** (ν_{max}, cm^{-1}) 3429 (w), 2915 (s), 2856 (m), 2360 (w), 1891 (w), 1804 (w), 1732 (s), 1598 (w), 1491 (w), 1455 (m), 1436 (m), 1346 (m), 1246 (s), 1184 (s), 1105 (w), 1057 (s), 1004 (w), 970 (w), 922 (w), 838 (w), 763 (m), 733 (m); **HRMS** (ESI/QTOF) m/z: $[M + Na]^+$ Calcd for $C_{33}H_{40}NaO_6^+$ 555.2717; Found 555.2733.

4.1.8. Synthesis and characterization of (*E*)-2'-(3-(4-methoxyphenyl)allyl) 2,2-dimethyl 3'-hexyl-3-phenyl-[1,1'-bi(cyclopropane)]-3,3'-diene-2,2,2'-tricarboxylate (3h)

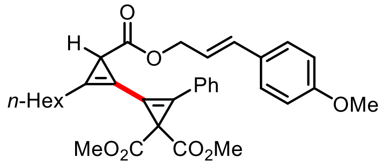

3h

Following **GPD**, a mixture of $(Me_2S)AuCl$ (1.47 mg, 5.00 μmol, 5.00 mol%), $AgNTf_2$ (1.94 mg, 5.00 μmol, 5.00 mol%), **L1** (5.25 mg, 25.0 μmol, 25.0 mol%), CpBX **2h** (88.7 mg, 130 μmol, 1.30 equiv.), terminal cyclopropene **1a** (23.2 mg, 100 μmol, 1.00 equiv.) and CH_3CN (2.0 mL) was stirred at 40 °C for 24 hours. Flash column chromatography on silica gel (eluent: pentane/ethyl acetate = 5:1) afforded **3h** in 58% yield (31.7 mg, 58.2 μmol) as a colorless oil and **4** in 88% NMR yield. **TLC**: R_f (*n*-hexane/EtOAc = 3:1) = 0.38; **^{1}H NMR** (400 MHz, $CDCl_3$) δ 7.67 – 7.55 (m, 2H, Ar*H*), 7.44 – 7.33 (m, 3H, Ar*H*), 7.30 – 7.20 (m, 2H, Ar*H*), 6.88 – 6.76 (m, 2H, Ar*H*), 6.57 (d, *J* = 15.9 Hz, 1H, $CH_2CH{=}CH$), 6.14 (dt, *J* = 15.9, 6.4 Hz, 1H, $CH_2CH{=}CH$), 4.85 – 4.66 (m, 2H, $CH_2CH{=}CH$), 3.80 (s, 3H, Ar-OCH_3), 3.71 (s, 3H, CO_2CH_3), 3.69 (s, 3H, CO_2CH_3), 2.75 – 2.62 (m, 2H, CH_2CH_2C), 2.64 (s, 1H, $CHCO_2$), 1.74 – 1.67 (m, 2H, CH_2CH_2C), 1.51 – 1.35 (m, 2H, CH_2), 1.34 – 1.19 (m, 4H, CH_2), 0.92 – 0.82 (m, 3H, CH_3); **^{13}C NMR** (101 MHz, $CDCl_3$) δ 174.1, 170.2, 170.0, 159.6, 133.5, 130.8, 130.7, 129.2, 129.1, 127.9, 124.6, 121.3, 119.3, 114.1, 109.2, 94.7, 92.7, 65.4,

55.4, 52.58, 52.55, 35.5, 31.6, 28.9, 26.9, 26.2, 23.9, 22.6, 14.2; **IR** (ν_{max}, cm^{-1}) 2999 (w), 2954 (m), 2934 (m), 2859 (w), 2359 (w), 1894 (w), 1801 (w), 1730 (s), 1607 (m), 1577 (w), 1512 (m), 1438 (w), 1379 (w), 1334 (w), 1285 (s), 1249 (s), 1172 (s), 1116 (w), 1064 (m), 1034 (w), 971 (m), 842 (w), 802 (w), 764 (m), 731 (w); **HRMS** (ESI/QTOF) m/z: $[M + Na]^+$ Calcd for $C_{33}H_{36}NaO_7^+$ 567.2353; Found 567.2359.

4.1.9. Synthesis and characterization of 2'-ethyl 2,2-dimethyl 3'-hexyl-2'-methyl-3-phenyl-[1,1'-bi(cyclopropane)]-3,3'-diene-2,2,2'-tricarboxylate (3i)

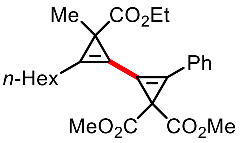

3i

Following **GPD**, a mixture of $(Me_2S)AuCl$ (1.47 mg, 5.00 µmol, 5.00 mol%), $AgNTf_2$ (1.94 mg, 5.00 µmol, 5.00 mol%), **L1** (5.25 mg, 25.0 µmol, 25.0 mol%), CpBX **2i** (75.2 mg, 130 µmol, 1.30 equiv.), terminal cyclopropene **1a** (23.2 mg, 100 µmol, 1.00 equiv.) and CH_3CN (2.0 mL) was stirred at 40 °C for 17 hours. Flash column chromatography on silica gel (eluent: pentane/ethyl acetate = 5:1) afforded **3i** in 42% yield (18.7 mg, 42.4 µmol) as a colorless oil and **4** in 85% NMR yield. **TLC**: R_f (*n*-hexane/EtOAc = 4:1) = 0.39; **^{1}H NMR** (400 MHz, $CDCl_3$) δ 7.66 – 7.53 (m, 2H, Ar*H*), 7.49 – 7.35 (m, 3H, Ar*H*), 4.10 (q, *J* = 7.1 Hz, 2H, $CO_2CH_2CH_3$), 3.72 (s, 6H, CO_2CH_3), 2.71 – 2.58 (m, 2H, CH_2CH_2C), 1.72 – 1.64 (m, 2H, CH_2CH_2C), 1.50 (s, 3H, $C(CH_3)$), 1.47 – 1.37 (m, 2H, CH_2), 1.36 – 1.25 (m, 4H, CH_2), 1.18 (t, *J* = 7.1 Hz, 3H, $CO_2CH_2CH_3$), 0.97 – 0.83 (m, 3H, CH_3); **^{13}C NMR** (101 MHz, $CDCl_3$) δ 175.6, 170.21, 170.20, 130.6, 130.5, 129.2, 125.7, 124.9, 108.7, 98.4, 94.7, 60.7, 52.52, 52.48, 35.3, 31.6, 29.0, 28.5, 27.1, 25.5, 22.7, 19.3, 14.4, 14.2; **IR** (ν_{max}, cm^{-1}) 2955 (m), 2931 (m), 2861 (w), 1890 (w), 1798 (w), 1734 (s), 1488 (w), 1448 (w), 1436 (m), 1374 (w), 1281 (s), 1246 (s), 1115 (m), 1064 (m), 1027 (w), 937 (w), 764 (m); **HRMS** (ESI/QTOF) m/z: $[M + Na]^+$ Calcd for $C_{26}H_{32}NaO_6^+$ 463.2091; Found 463.2102.

4.1.10. Synthesis and characterization of dimethyl 3-phenyl-2'-(3-phenylpropyl)-3'-(trifluoromethyl)-[1,1'-bi(cyclopropane)]-1',3-diene-2,2-dicarboxylate (3j)

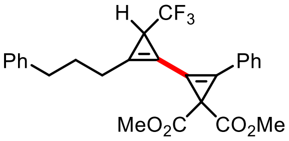

3j

Following **GPD**, a mixture of $(Me_2S)AuCl$ (1.47 mg, 5.00 µmol, 5.00 mol%), $AgNTf_2$ (1.94 mg, 5.00 µmol, 5.00 mol%), **L1** (5.25 mg, 25.0 µmol, 25.0 mol%), CpBX **2j** (77.3 mg, 130 µmol, 1.30 equiv.), terminal cyclopropene **1a** (23.2 mg, 100 µmol, 1.00 equiv.) and CH_3CN (2.0 mL) was stirred at 40 °C for 17 hours. Flash column chromatography on silica gel (eluent: pentane/ethyl acetate = 5:1) afforded **3j** in 71% yield (32.2 mg, 70.5 µmol) as a colorless oil and **4** in 81% NMR yield. **TLC**: R_f (*n*-hexane/EtOAc = 10:1) = 0.15; **^{1}H NMR** (400 MHz, $CDCl_3$) δ 7.73 – 7.60 (m, 2H, Ar*H*), 7.49 – 7.44 (m, 3H, Ar*H*), 7.33 – 7.27 (m, 2H, Ar*H*), 7.23 – 7.17 (m, 3H, Ar*H*), 3.72 (s, 3H, CO_2CH_3), 3.70 (s, 3H, CO_2CH_3), 2.76 – 2.69 (m, 4H, CH_2), 2.47 (q, *J* = 4.4 Hz, 1H, $CH(CF_3)$), 2.11 – 2.01 (m, 2H, CH_2); **^{13}C NMR** (101 MHz, $CDCl_3$) δ 170.1, 169.9, 141.2, 131.2, 130.9, 129.2, 128.61, 128.59, 126.2, 125.7 (q, *J* = 275.5 Hz), 124.4, 117.8 (q, *J* = 2.7 Hz), 110.0, 94.0, 93.0 (q, *J* = 3.6 Hz), 52.7, 52.6, 35.3, 35.0, 28.4, 25.4, 22.9 (q, *J* = 39.3 Hz); **^{19}F NMR** (376 MHz, $CDCl_3$) δ -66.5 ($CHCF_3$); **IR** (ν_{max}, cm^{-1}) 3085 (w), 3062 (w), 3030 (w), 2953 (w), 2845 (w), 1888 (w), 1796 (w), 1732 (s), 1603 (w), 1494 (w), 1449 (w), 1436 (w), 1358 (w), 1270 (s), 1246 (s), 1129 (s), 1062 (m), 1002 (w), 975 (w), 921 (w), 831 (w), 762 (m); **HRMS** (ESI/QTOF) m/z: $[M + Na]^+$ Calcd for $C_{26}H_{23}F_3NaO_4^+$ 479.1441; Found 479.1442.

4.1.11. Synthesis and characterization of tetramethyl 3,3'-diphenyl-[1,1'-bi(cyclopropane)]-3,3'-diene-2,2,2',2'-tetracarboxylate (3k)

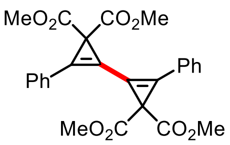

3k

Following **GPD**, a mixture of (Me_2S)AuCl (1.47 mg, 5.00 µmol, 5.00 mol%), $AgNTf_2$ (1.94 mg, 5.00 µmol, 5.00 mol%), **L1** (5.25 mg, 25.0 µmol, 25.0 mol%), CpBX **2k** (78.0 mg, 130 µmol, 1.30 equiv.), terminal cyclopropene **1a** (23.2 mg, 100 µmol, 1.00 equiv.) and CH_3CN (2.0 mL) was stirred at 40 °C for 19 hours. Flash column chromatography on silica gel (eluent: pentane/ethyl acetate = 3:1) afforded **3k** in 60% yield (27.6 mg, 59.7 µmol) as a colorless solid and **4** in 81% NMR yield. **M.p.** 151 – 153 °C. **TLC**: R_f (*n*-hexane/EtOAc = 3:1) = 0.18; **^{1}H NMR** (400 MHz, $CDCl_3$) δ 7.83 – 7.70 (m, 4H, Ar*H*), 7.59 – 7.41 (m, 6H, Ar*H*), 3.76 (s, 12H, CO_2CH_3); **^{13}C NMR** (101 MHz, $CDCl_3$) δ 169.8, 131.4, 131.2, 129.3, 124.5, 112.2, 93.3, 52.8, 35.8; **IR** (ν_{max}, cm^{-1}) 3026 (w), 2958 (w), 1791 (w), 1736 (s), 1727 (s), 1596 (w), 1487 (w), 1436 (m), 1281 (s), 1240 (s), 1205 (m), 1146 (m), 1056 (s), 971 (w), 937 (w), 914 (w), 836 (w), 759 (m), 734 (m); **HRMS** (ESI/QTOF) m/z: $[M + Na]^+$ Calcd for $C_{26}H_{22}NaO_8^+$ 485.1207; Found 485.1201.

4.1.12. Synthesis and characterization of tetramethyl 3-(4-fluorophenyl)-3'-phenyl-[1,1'-bi(cyclopropane)]-3,3'-diene-2,2,2',2'-tetracarboxylate (3l)

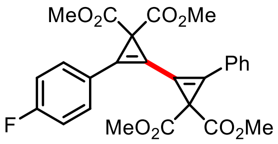

3l

Following **GPD**, a mixture of (Me_2S)AuCl (1.47 mg, 5.00 µmol, 5.00 mol%), $AgNTf_2$ (1.94 mg, 5.00 µmol, 5.00 mol%), **L1** (5.25 mg, 25.0 µmol, 25.0 mol%), CpBX **2l** (80.4 mg, 130 µmol, 1.30 equiv.), terminal cyclopropene **1a** (23.2 mg, 100 µmol, 1.00 equiv.) and CH_3CN (2.0 mL) was stirred at 40 °C for 24 hours. Flash column chromatography on silica gel (eluent: pentane/ethyl acetate = 3:1) afforded **3l** in 45% yield (21.5 mg, 44.8 µmol) as a light yellow solid and **4** in 88% NMR yield. **M.p.** 170 – 172 °C. **TLC**: R_f (*n*-hexane/EtOAc = 3:1) = 0.20; **^{1}H NMR** (400 MHz, $CDCl_3$) δ 7.83 – 7.69 (m, 4H, Ar*H*), 7.55 – 7.42 (m, 3H, Ar*H*), 7.25 – 7.11 (m, 2H, Ar*H*), 3.764 (s, 6H, CO_2CH_3), 3.760 (s, 6H, CO_2CH_3); **^{13}C NMR** (101 MHz, $CDCl_3$) δ 169.8, 169.7, 164.5 (d, *J* = 254.5 Hz), 133.4 (d, *J* = 8.9 Hz), 131.5, 131.3, 129.3, 124.5, 120.9 (d, *J* = 3.2 Hz), 116.8 (d, *J* = 22.5 Hz), 112.1, 111.1, 93.2, 93.0 (d, *J* = 2.8 Hz), 52.89, 52.86, 35.9, 35.8; **^{19}F NMR** (377 MHz, Chloroform-*d*) δ -106.2; **IR** (ν_{max}, cm^{-1}) 3006 (w), 2956 (w), 2845 (w), 1795 (w), 1729 (s), 1598 (m), 1506 (m), 1491 (w), 1435 (m), 1281 (s), 1236 (s), 1153 (m), 1098 (w), 1056 (s), 971 (w), 936 (w), 918 (w), 840 (m), 815 (w), 763 (m), 736 (w); **HRMS** (ESI/QTOF) m/z: $[M + Na]^+$ Calcd for $C_{26}H_{21}FNaO_8^+$ 503.1113; Found 503.1125.

4.1.13. Synthesis and characterization of tetramethyl 3-(4-bromophenyl)-3'-phenyl-[1,1'-bi(cyclopropane)]-3,3'-diene-2,2,2',2'-tetracarboxylate (3m)

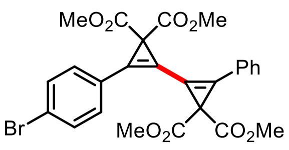

3m

Following **GPD**, a mixture of (Me_2S)AuCl (1.47 mg, 5.00 µmol, 5.00 mol%), $AgNTf_2$ (1.94 mg, 5.00 µmol, 5.00 mol%), **L1** (5.25 mg, 25.0 µmol, 25.0 mol%), CpBX **2m** (88.3 mg, 130 µmol, 1.30 equiv.), terminal

cyclopropene **1a** (23.2 mg, 100 μmol, 1.00 equiv.) and CH_3CN (2.0 mL) was stirred at 40 °C for 24 hours. Flash column chromatography on silica gel (eluent: pentane/ethyl acetate = 3:1) afforded **3m** in 50% yield (27.0 mg, 49.9 μmol) as a light yellow solid and **4** in 97% NMR yield. **M.p.** 189 – 190 °C. **TLC**: R_f (*n*-hexane/EtOAc = 3:1) = 0.22; **^{1}H NMR** (400 MHz, $CDCl_3$) δ 7.77 – 7.72 (m, 2H, Ar*H*), 7.64 – 7.60 (m, 4H, Ar*H*), 7.52 – 7.46 (m, 3H, Ar*H*), 3.76 (s, 12H, CO_2CH_3); **^{13}C NMR** (101 MHz, $CDCl_3$) δ 169.7, 169.5, 132.7, 132.4, 131.6, 131.3, 129.3, 126.2, 124.4, 123.5, 112.7, 111.1, 94.2, 93.1, 52.88, 52.85, 35.8 (2C); **IR** (ν_{max}, cm^{-1}) 3007 (w), 2953 (w), 2844 (w), 1794 (w), 1731 (s), 1583 (w), 1481 (w), 1434 (m), 1398 (w), 1278 (s), 1238 (s), 1205 (m), 1147 (m), 1055 (s), 1010 (w), 968 (m), 946 (w), 917 (m), 829 (m), 763 (m), 736 (m); **HRMS** (ESI/QTOF) m/z: $[M + Na]^+$ Calcd for $C_{26}H_{21}BrNaO_8^+$ 563.0312; Found 563.0317.

4.1.14. Synthesis and characterization of tetramethyl 3-hexyl-3'-phenyl-[1,1'-bi(cyclopropane)]-3,3'-diene-2,2,2',2'-tetracarboxylate (3n)

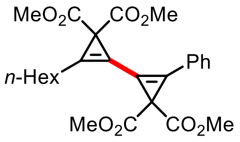

3n

Following **GPD**, a mixture of $(Me_2S)AuCl$ (1.47 mg, 5.00 μmol, 5.00 mol%), $AgNTf_2$ (1.94 mg, 5.00 μmol, 5.00 mol%), **L1** (5.25 mg, 25.0 μmol, 25.0 mol%), CpBX **2n** (122 mg, 200 μmol, 2.00 equiv.), terminal cyclopropene **1a** (23.2 mg, 100 μmol, 1.00 equiv.) and CH_3CN (2.0 mL) was stirred at 40 °C for 28 hours. Flash column chromatography on silica gel (eluent: pentane/ethyl acetate = 4:1) afforded **3n** in 50% yield (23.6 mg, 50.2 μmol) as a colorless oil and **4** in 49% NMR yield. **TLC**: R_f (*n*-hexane/EtOAc = 3:1) = 0.28; **^{1}H NMR** (400 MHz, $CDCl_3$) δ 7.69 – 7.64 (m, 2H, Ar*H*), 7.51 – 7.37 (m, 3H, Ar*H*), 3.73 (s, 6H, CO_2CH_3), 3.72 (s, 6H, CO_2CH_3), 2.72 (t, *J* = 7.3 Hz, 2H, CH_2CH_2C), 1.71 (p, *J* = 7.3 Hz, 2H, CH_2CH_2C), 1.58 – 1.35 (m, 2H, CH_2), 1.35 – 1.17 (m, 4H, CH_2), 1.03 – 0.76 (m, 3H, CH_3); **^{13}C NMR** (101 MHz, $CDCl_3$) δ 170.3, 169.7, 131.2, 131.0, 129.2, 124.3, 117.1, 111.4, 93.1, 93.0, 52.63, 52.57, 35.7, 35.5, 31.5, 28.8, 26.7, 25.2, 22.6, 14.2; **IR** (ν_{max}, cm^{-1}) 2999 (w), 2953 (m), 2931 (w), 2860 (w), 1805 (w), 1732 (s), 1490 (w), 1435 (m), 1283 (s), 1242 (s), 1191 (w), 1146 (w), 1114 (w), 1063 (s), 975 (w), 932 (w), 841 (w), 766 (m), 741 (w); **HRMS** (ESI/QTOF) m/z: $[M + Na]^+$ Calcd for $C_{26}H_{30}NaO_8^+$ 493.1833; Found 493.1847.

4.1.15. Synthesis and characterization of 2,2-dibenzyl 2',2'-dimethyl 3,3'-diphenyl-[1,1'-bi(cyclopropane)]-3,3'-diene-2,2,2',2'-tetracarboxylate (3o)

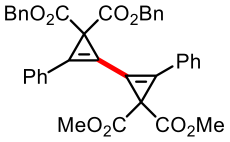

3o

Following **GPD**, a mixture of $(Me_2S)AuCl$ (1.47 mg, 5.00 μmol, 5.00 mol%), $AgNTf_2$ (1.94 mg, 5.00 μmol, 5.00 mol%), **L1** (5.25 mg, 25.0 μmol, 25.0 mol%), CpBX **2o** (86.5 mg, 115 μmol, 1.15 equiv.), terminal cyclopropene **1a** (23.2 mg, 100 μmol, 1.00 equiv.) and CH_3CN (2.0 mL) was stirred at 40 °C for 24 hours. Flash column chromatography on silica gel (eluent: pentane/ethyl acetate = 3:1) afforded **3o** in 40% yield (24.3 mg, 39.5 μmol) as a colorless oil and **4** in 94% NMR yield. **TLC**: R_f (*n*-hexane/EtOAc = 3:1) = 0.27; **^{1}H NMR** (400 MHz, $CDCl_3$) δ 7.77 – 7.74 (m, 2H, Ar*H*), 7.69 – 7.59 (m, 2H, Ar*H*), 7.53 – 7.39 (m, 4H, Ar*H*), 7.37 – 7.32 (m, 2H, Ar*H*), 7.26 – 7.10 (m, 10H, Ar*H*), 5.31 – 5.09 (m, 4H, OCH_2Ph), 3.68 (s, 6H, CO_2CH_3); **^{13}C NMR** (101 MHz, $CDCl_3$) δ 169.7, 169.1, 135.8, 131.4, 131.31, 131.28, 131.2, 129.3, 129.2, 128.5, 128.1, 127.7, 124.5, 124.3, 111.9, 111.6, 93.3, 93.2, 67.2, 52.7, 36.3, 35.9; **IR** (ν_{max}, cm^{-1}) 3065 (w), 3034 (w), 2953 (w), 2848 (w), 1797 (w), 1730 (s), 1599 (w), 1491 (w), 1449 (m), 1437 (w), 1375 (w), 1276 (s), 1245 (s), 1176 (w), 1149 (w), 1060 (s), 1029 (w), 972 (w), 917 (w), 761 (m), 745 (m); **HRMS** (ESI/QTOF) m/z: $[M + K]^+$ Calcd for $C_{38}H_{30}KO_8^+$ 653.1572; Found 653.1578.

4.2. Substrate Scope of Terminal Cyclopropenes

4.2.1. Synthesis and characterization of 2'-ethyl 2,2-dimethyl 3'-hexyl-3-(*p*-tolyl)-[1,1'-bi(cyclopropane)]-3,3'-diene-2,2,2'-tricarboxylate (3p)

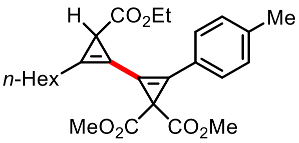

3p

Following **GPD**, a mixture of $(Me_2S)AuCl$ (1.47 mg, 5.00 µmol, 5.00 mol%), $AgNTf_2$ (1.94 mg, 5.00 µmol, 5.00 mol%), **L1** (5.25 mg, 25.0 µmol, 25.0 mol%), CpBX **2a** (73.4 mg, 130 µmol, 1.30 equiv.), terminal cyclopropene **1b** (24.6 mg, 100 µmol, 1.00 equiv.) and CH_3CN (2.0 mL) was stirred at 40 °C for 11 hours. Flash column chromatography on silica gel (eluent: pentane/ethyl acetate = 5:1) afforded **3p** in 68% yield (30.1 mg, 68.3 µmol) as a colorless oil and **4** in 83% NMR yield. **TLC**: R_f (*n*-hexane/EtOAc = 4:1) = 0.38; **^{1}H NMR** (400 MHz, $CDCl_3$) δ 7.54 – 7.45 (m, 2H, Ar*H*), 7.26 – 7.20 (m, 2H, Ar*H*), 4.18 – 4.11 (m, 2H, $CO_2CH_2CH_3$), 3.71 (s, 3H, CO_2CH_3), 3.70 (s, 3H, CO_2CH_3), 2.71 – 2.61 (m, 2H, CH_2CH_2C), 2.57 (s, 1H, C*H*CO_2), 2.38 (s, 3H, Ar-CH_3), 1.69 (p, *J* = 7.3 Hz, 2H, CH_2CH_2C), 1.46 – 1.19 (m, 9H, CH_2 & $CO_2CH_2CH_3$), 0.94 – 0.83 (m, 3H, CH_3); **^{13}C NMR** (101 MHz, $CDCl_3$) δ 174.4, 170.3, 170.1, 141.4, 130.6, 129.9, 121.8, 118.7, 109.2, 93.6, 92.8, 60.5, 52.50, 52.49, 35.3, 31.6, 28.9, 26.9, 26.2, 23.9, 22.6, 21.8, 14.5, 14.2; **IR** (ν_{max}, cm^{-1}) 3029 (w), 2954 (m), 2931 (m), 2860 (w), 1804 (w), 1730 (s), 1605 (w), 1508 (w), 1459 (w), 1436 (m), 1370 (w), 1334 (w), 1282 (m), 1245 (s), 1182 (m), 1112 (w), 1063 (m), 1038 (w), 930 (w), 821 (m), 737 (w); **HRMS** (ESI/QTOF) m/z: $[M + Na]^+$ Calcd for $C_{26}H_{32}NaO_6^+$ 463.2091; Found 463.2103.

4.2.2. Synthesis and characterization of 2'-ethyl 2,2-dimethyl 3'-hexyl-3-(4-pentylphenyl)-[1,1'-bi(cyclopropane)]-3,3'-diene-2,2,2'-tricarboxylate (3q)

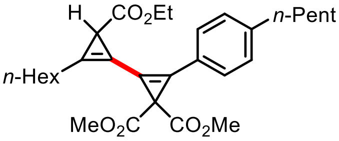

3q

Following **GPD**, a mixture of $(Me_2S)AuCl$ (1.47 mg, 5.00 µmol, 5.00 mol%), $AgNTf_2$ (1.94 mg, 5.00 µmol, 5.00 mol%), **L1** (5.25 mg, 25.0 µmol, 25.0 mol%), CpBX **2a** (73.4 mg, 130 µmol, 1.30 equiv.), terminal cyclopropene **1c** (30.2 mg, 100 µmol, 1.00 equiv.) and CH_3CN (2.0 mL) was stirred at 40 °C for 18 hours. Flash column chromatography on silica gel (eluent: pentane/ethyl acetate = 5:1) afforded **3q** in 47% yield (23.5 mg, 47.3 µmol) as a colorless oil and **4** in 81% NMR yield. **TLC**: R_f (*n*-hexane/EtOAc = 4:1) = 0.46; **^{1}H NMR** (400 MHz, $CDCl_3$) δ 7.57 – 7.46 (m, 2H, Ar*H*), 7.25 – 7.22 (m, 2H, Ar*H*), 4.19 – 4.11 (m, 2H, $CO_2CH_2CH_3$), 3.72 (s, 3H, CO_2CH_3), 3.71 (s, 3H, CO_2CH_3), 2.71 – 2.59 (m, 4H, CH_2CH_2C & $ArCH_2CH_2$), 2.57 (s, 1H, C*H*CO_2), 1.73 – 1.53 (m, 4H, CH_2CH_2C & $ArCH_2CH_2$), 1.47 – 1.16 (m, 13H, CH_2 & $CO_2CH_2CH_3$), 0.93 – 0.82 (m, 6H, CH_3 × 2); **^{13}C NMR** (101 MHz, $CDCl_3$) δ 174.3, 170.2, 170.1, 146.3, 130.5, 129.1, 121.9, 118.6, 109.1, 93.5, 92.7, 60.4, 52.40, 52.39, 36.0, 35.3, 31.5, 31.4, 30.9, 28.8, 26.8, 26.1, 23.8, 22.5 (2C), 14.4, 14.1, 14.0; **IR** (ν_{max}, cm^{-1}) 2955 (m), 2931 (m), 2859 (m), 1897 (w), 1803 (w), 1731 (s), 1605 (w), 1508 (w), 1462 (w), 1436 (m), 1415 (w), 1370 (w), 1334 (w), 1282 (s), 1245 (s), 1182 (m), 1114 (w), 1064 (m), 933 (w), 841 (w), 800 (w), 740 (w); **HRMS** (ESI/QTOF) m/z: $[M + Na]^+$ Calcd for $C_{30}H_{40}NaO_6^+$ 519.2717; Found 519.2735.

4.2.3. Synthesis and characterization of 2'-ethyl 2,2-dimethyl 3-(4-(*tert*-butyl)phenyl)-3'-hexyl-[1,1'-bi(cyclopropane)]-3,3'-diene-2,2,2'-tricarboxylate (3r)

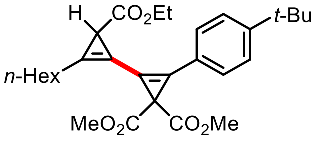

3r

Following **GPD**, a mixture of $(Me_2S)AuCl$ (1.47 mg, 5.00 μmol, 5.00 mol%), $AgNTf_2$ (1.94 mg, 5.00 μmol, 5.00 mol%), **L1** (5.25 mg, 25.0 μmol, 25.0 mol%), CpBX **2a** (73.4 mg, 130 μmol, 1.30 equiv.), terminal cyclopropene **1d** (28.8 mg, 100 μmol, 1.00 equiv.) and CH_3CN (2.0 mL) was stirred at 40 °C for 15 hours. Flash column chromatography on silica gel (eluent: pentane/ethyl acetate = 6:1) afforded **3r** in 69% yield (33.4 mg, 69.2 μmol) as a colorless oil and **4** in 84% NMR yield. **TLC**: R_f (*n*-hexane/EtOAc = 4:1) = 0.47; **^{1}H NMR** (400 MHz, $CDCl_3$) δ 7.57 – 7.52 (m, 2H, Ar*H*), 7.48 – 7.43 (m, 2H, Ar*H*), 4.19 – 4.11 (m, 2H, $CO_2CH_2CH_3$), 3.714 (s, 3H, CO_2CH_3), 3.706 (s, 3H, CO_2CH_3), 2.72 – 2.60 (m, 2H, CH_2CH_2C), 2.57 (s, 1H, $CHCO_2$), 1.69 (p, *J* = 7.3 Hz, 2H, CH_2CH_2C), 1.48 – 1.19 (m, 18H, CH_2 & *t-Bu* & $CO_2CH_2CH_3$), 1.00 – 0.84 (m, 3H, CH_3); **^{13}C NMR** (101 MHz, $CDCl_3$) δ 174.4, 170.3, 170.2, 154.5, 130.4, 126.2, 121.8, 118.7, 109.1, 93.7, 92.8, 60.6, 52.5 (2C), 35.3, 35.2, 31.6, 31.2, 28.9, 26.9, 26.2, 23.9, 22.6, 14.5, 14.2; **IR** (ν_{max}, cm^{-1}) 2957 (m), 2934 (m), 2867 (w), 1804 (w), 1731 (s), 1605 (w), 1510 (w), 1463 (w), 1436 (m), 1409 (w), 1368 (w), 1334 (w), 1281 (s), 1269 (s), 1244 (s), 1182 (m), 1099 (w), 1064 (m), 1027 (w), 927 (w), 842 (w), 734 (w); **HRMS** (ESI/QTOF) m/z: $[M + Na]^+$ Calcd for $C_{29}H_{38}NaO_6^+$ 505.2561; Found 505.2564.

4.2.4. Synthesis and characterization of 2'-ethyl 2,2-dimethyl 3-([1,1'-biphenyl]-4-yl)-3'-hexyl-[1,1'-bi(cyclopropane)]-3,3'-diene-2,2,2'-tricarboxylate (3s)

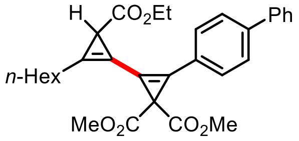

3s

Following **GPD**, a mixture of $(Me_2S)AuCl$ (1.47 mg, 5.00 μmol, 5.00 mol%), $AgNTf_2$ (1.94 mg, 5.00 μmol, 5.00 mol%), **L1** (5.25 mg, 25.0 μmol, 25.0 mol%), CpBX **2a** (73.4 mg, 130 μmol, 1.30 equiv.), terminal cyclopropene **1e** (30.8 mg, 100 μmol, 1.00 equiv.) and CH_3CN (2.0 mL) was stirred at 40 °C for 12 hours. Flash column chromatography on silica gel (eluent: pentane/ethyl acetate = 7:1) afforded **3s** in 82% yield (41.3 mg, 82.2 μmol) as a colorless oil and **4** in 92% NMR yield. **TLC**: R_f (*n*-hexane/EtOAc = 4:1) = 0.43; **^{1}H NMR** (400 MHz, $CDCl_3$) δ 7.73 – 7.64 (m, 4H, Ar*H*), 7.63 – 7.56 (m, 2H, Ar*H*), 7.50 – 7.43 (m, 2H, Ar*H*), 7.41 – 7.32 (m, 1H, Ar*H*), 4.26 – 4.08 (m, 2H, $CO_2CH_2CH_3$), 3.75 (s, 3H, CO_2CH_3), 3.74 (s, 3H, CO_2CH_3), 2.74 – 2.65 (m, 2H, CH_2CH_2C), 2.61 (s, 1H, $CHCO_2$), 1.72 (p, *J* = 7.3 Hz, 2H, CH_2CH_2C), 1.49 – 1.19 (m, 9H, CH_2 & $CO_2CH_2CH_3$), 1.00 – 0.81 (m, 3H, CH_3); **^{13}C NMR** (101 MHz, $CDCl_3$) δ 174.3, 170.2, 170.0, 143.6, 140.2, 131.1, 129.1, 128.2, 127.8, 127.3, 123.5, 119.5, 108.8, 94.8, 92.8, 60.6, 52.58, 52.57, 35.4, 31.6, 28.9, 26.9, 26.2, 23.9, 22.6, 14.5, 14.2; **IR** (ν_{max}, cm^{-1}) 3032 (w), 2953 (m), 2931 (m), 2859 (w), 1891 (w), 1804 (w), 1728 (s), 1602 (w), 1486 (w), 1459 (w), 1435 (m), 1407 (w), 1370 (w), 1335 (w), 1281 (m), 1244 (s), 1182 (s), 1114 (w), 1063 (s), 1028 (w), 1007 (w), 978 (w), 927 (w), 844 (m), 767 (m), 727 (w); **HRMS** (ESI/QTOF) m/z: $[M + Na]^+$ Calcd for $C_{31}H_{34}NaO_6^+$ 525.2248; Found 525.2266.

4.2.5. Synthesis and characterization of 2'-ethyl 2,2-dimethyl 3-(4-fluorophenyl)-3'-hexyl-[1,1'-bi(cyclopropane)]-3,3'-diene-2,2,2'-tricarboxylate (3t)

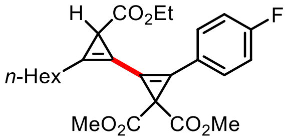

3t

Following **GPD**, a mixture of $(Me_2S)AuCl$ (1.47 mg, 5.00 μmol, 5.00 mol%), $AgNTf_2$ (1.94 mg, 5.00 μmol, 5.00 mol%), **L1** (5.25 mg, 25.0 μmol, 25.0 mol%), CpBX **2a** (84.7 mg, 150 μmol, 1.50 equiv.), terminal cyclopropene **1f** (25.0 mg, 100 μmol, 1.00 equiv.) and CH_3CN (2.0 mL) was stirred at 40 °C for 16 hours. Flash column chromatography on silica gel (eluent: pentane/ethyl acetate = 8:1) afforded **3t** in 76% yield (33.6 mg, 75.6 μmol) as a colorless oil and **4** in 81% NMR yield. **TLC**: R_f (*n*-hexane/EtOAc = 3:1) = 0.53; **^{1}H NMR** (400 MHz, $CDCl_3$) δ 7.66 – 7.53 (m, 2H, Ar*H*), 7.22 – 7.02 (m, 2H, Ar*H*), 4.15 (q, *J* = 7.1 Hz, 2H, $CO_2CH_2CH_3$), 3.722 (s, 3H, CO_2CH_3), 3.716 (s, 3H, CO_2CH_3), 2.71 – 2.62 (m, 2H, CH_2CH_2C), 2.57 (s, 1H, $CHCO_2$), 1.68 (p, *J* = 7.3 Hz, 2H, CH_2CH_2C), 1.44 – 1.15 (m, 9H, CH_2 & $CO_2CH_2CH_3$), 1.03 – 0.74 (m, 3H, CH_3); **^{13}C NMR** (101 MHz, $CDCl_3$) δ 174.2, 170.1, 169.9, 164.1 (d, *J* = 253.2 Hz), 132.7 (d, *J* = 8.7 Hz), 121.1 (d, *J* = 3.2 Hz), 119.5, 116.5 (d, *J* = 22.4 Hz), 108.1, 94.4 (d, *J* = 2.8 Hz), 92.6, 60.6, 52.60, 52.59, 35.5, 31.6, 28.9, 26.9, 26.2, 23.9, 22.6, 14.5, 14.2; **^{19}F NMR** (377 MHz, Chloroform-*d*) δ -107.5; **IR** (ν_{max}, cm^{-1}) 2955 (m), 2934 (m), 2859 (w), 1898 (w), 1805 (w), 1732 (s), 1599 (m), 1506 (m), 1461 (w), 1436 (m), 1370 (w), 1333 (w), 1283 (s), 1239 (s), 1185 (s), 1159 (m), 1114 (w), 1096 (w), 1064 (m), 1037 (w), 981 (w), 928 (w), 842 (m), 817 (w), 744 (w); **HRMS** (ESI/QTOF) m/z: $[M + Na]^+$ Calcd for $C_{25}H_{29}FNaO_6^+$ 467.1840; Found 467.1842.

4.2.6. Synthesis and characterization of 2'-ethyl 2,2-dimethyl 3-(4-chlorophenyl)-3'-hexyl-[1,1'-bi(cyclopropane)]-3,3'-diene-2,2,2'-tricarboxylate (3u)

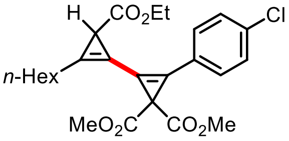

3u

Following **GPD**, a mixture of $(Me_2S)AuCl$ (1.47 mg, 5.00 μmol, 5.00 mol%), $AgNTf_2$ (1.94 mg, 5.00 μmol, 5.00 mol%), **L1** (5.25 mg, 25.0 μmol, 25.0 mol%), CpBX **2a** (73.4 mg, 130 μmol, 1.30 equiv.), terminal cyclopropene **1g** (26.7 mg, 100 μmol, 1.00 equiv.) and CH_3CN (2.0 mL) was stirred at 40 °C for 18 hours. Flash column chromatography on silica gel (eluent: pentane/ethyl acetate = 6:1) afforded **3u** in 53% yield (24.5 mg, 53.2 μmol) as a colorless oil and **4** in 69% NMR yield. **TLC**: R_f (*n*-hexane/EtOAc = 4:1) = 0.42; **^{1}H NMR** (400 MHz, $CDCl_3$) δ 7.56 – 7.49 (m, 2H, Ar*H*), 7.44 – 7.37 (m, 2H, Ar*H*), 4.15 (q, *J* = 7.1 Hz, 2H, $CO_2CH_2CH_3$), 3.723 (s, 3H, CO_2CH_3), 3.716 (s, 3H, CO_2CH_3), 2.72 – 2.62 (m, 2H, CH_2CH_2C), 2.58 (s, 1H, $CHCO_2$), 1.69 (p, *J* = 7.3 Hz, 2H, CH_2CH_2C), 1.48 – 1.19 (m, 9H, CH_2 & $CO_2CH_2CH_3$), 0.93 – 0.81 (m, 3H, CH_3); **^{13}C NMR** (101 MHz, $CDCl_3$) δ 174.1, 170.0, 169.8, 136.9, 131.7, 129.5, 123.2, 120.2, 108.0, 95.4, 92.6, 60.7, 52.64, 52.62, 35.5, 31.6, 28.9, 26.9, 26.3, 23.9, 22.6, 14.5, 14.2; **IR** (ν_{max}, cm^{-1}) 2954 (m), 2932 (m), 2860 (w), 1894 (w), 1804 (w), 1727 (s), 1590 (w), 1486 (m), 1458 (w), 1436 (m), 1402 (w), 1369 (w), 1335 (w), 1281 (s), 1245 (s), 1183 (s), 1088 (m), 1063 (s), 1014 (m), 978 (w), 929 (w), 833 (m), 734 (w); **HRMS** (ESI/QTOF) m/z: $[M + Na]^+$ Calcd for $C_{25}H_{29}ClNaO_6^+$ 483.1545; Found 483.1541.

4.2.7. Synthesis and characterization of 2'-ethyl 2,2-dimethyl 3-(4-bromophenyl)-3'-hexyl-[1,1'-bi(cyclopropane)]-3,3'-diene-2,2,2'-tricarboxylate (3v)

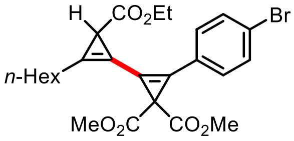

3v

Following **GPD**, a mixture of (Me_2S)AuCl (1.47 mg, 5.00 µmol, 5.00 mol%), $AgNTf_2$ (1.94 mg, 5.00 µmol, 5.00 mol%), **L1** (5.25 mg, 25.0 µmol, 25.0 mol%), CpBX **2a** (73.4 mg, 130 µmol, 1.30 equiv.), terminal cyclopropene **1h** (31.1 mg, 100 µmol, 1.00 equiv.) and CH_3CN (2.0 mL) was stirred at 40 °C for 13 hours. Flash column chromatography on silica gel (eluent: pentane/ethyl acetate = 7:1) afforded **3v** in 73% yield (36.7 mg, 72.6 µmol) as a colorless oil and **4** in 77% NMR yield. **TLC**: R_f (*n*-hexane/EtOAc = 4:1) = 0.48; **^{1}H NMR** (400 MHz, $CDCl_3$) δ 7.61 – 7.52 (m, 2H, Ar*H*), 7.51 – 7.41 (m, 2H, Ar*H*), 4.15 (q, *J* = 7.1 Hz, 2H, $CO_2CH_2CH_3$), 3.72 (s, 3H, CO_2CH_3), 3.71 (s, 3H, CO_2CH_3), 2.71 – 2.62 (m, 2H, CH_2CH_2C), 2.57 (s, 1H, C*H*CO_2), 1.68 (p, *J* = 7.3 Hz, 2H, CH_2CH_2C), 1.47 – 1.15 (m, 9H, CH_2 & $CO_2CH_2CH_3$), 0.99 – 0.79 (m, 3H, CH_3); **^{13}C NMR** (101 MHz, $CDCl_3$) δ 174.1, 169.9, 169.8, 132.5, 131.9, 125.4, 123.6, 120.3, 108.1, 95.6, 92.6, 60.7, 52.63, 52.62, 35.4, 31.6, 28.9, 26.9, 26.3, 23.9, 22.6, 14.5, 14.2; **IR** (ν_{max}, cm^{-1}) 2955 (m), 2932 (m), 2858 (w), 1894 (w), 1801 (w), 1729 (s), 1585 (w), 1484 (m), 1460 (w), 1436 (m), 1397 (w), 1369 (w), 1333 (w), 1280 (s), 1247 (s), 1187 (s), 1097 (w), 1065 (s), 1030 (w), 1011 (m), 975 (w), 930 (w), 829 (m), 797 (w), 737 (w); **HRMS** (ESI/QTOF) m/z: $[M + Na]^+$ Calcd for $C_{25}H_{29}BrNaO_6^+$ 527.1040; Found 527.1041.

4.2.8. Synthesis and characterization of 2'-ethyl 2,2-dimethyl 3'-hexyl-3-(4-(methoxycarbonyl)phenyl)-[1,1'-bi(cyclopropane)]-3,3'-diene-2,2,2'-tricarboxylate (3w)

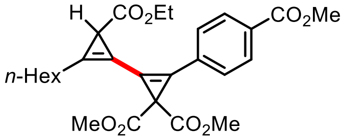

3w

Following **GPD**, a mixture of (Me_2S)AuCl (1.47 mg, 5.00 µmol, 5.00 mol%), $AgNTf_2$ (1.94 mg, 5.00 µmol, 5.00 mol%), **L1** (5.25 mg, 25.0 µmol, 25.0 mol%), CpBX **2a** (73.4 mg, 130 µmol, 1.30 equiv.), terminal cyclopropene **1i** (29.0 mg, 100 µmol, 1.00 equiv.) and CH_3CN (2.0 mL) was stirred at 40 °C for 20 hours. Flash column chromatography on silica gel (eluent: pentane/ethyl acetate = 4:1) afforded **3w** in 75% yield (36.1 mg, 74.5 µmol) as a colorless oil and **4** in 86% NMR yield. **TLC**: R_f (*n*-hexane/EtOAc = 4:1) = 0.27; **^{1}H NMR** (400 MHz, $CDCl_3$) δ 8.11 – 8.04 (m, 2H, Ar*H*), 7.69 – 7.61 (m, 2H, Ar*H*), 4.19 – 4.12 (m, 2H, $CO_2CH_2CH_3$), 3.92 (s, 3H, Ar-CO_2CH_3), 3.724 (s, 3H, CO_2CH_3), 3.717 (s, 3H, CO_2CH_3), 2.73 – 2.64 (m, 2H, CH_2CH_2C), 2.60 (s, 1H, C*H*CO_2), 1.69 (p, *J* = 7.3 Hz, 2H, CH_2CH_2C), 1.49 – 1.15 (m, 9H, CH_2 & $CO_2CH_2CH_3$), 0.96 – 0.80 (m, 3H, CH_3); **^{13}C NMR** (101 MHz, $CDCl_3$) δ 174.0, 169.8, 169.7, 166.4, 131.6, 130.4, 130.2, 128.8, 121.5, 108.1, 97.5, 92.6, 60.7, 52.7 (2C), 52.5, 35.5, 31.5, 28.9, 26.9, 26.3, 24.0, 22.6, 14.4, 14.1; **IR** (ν_{max}, cm^{-1}) 2955 (m), 2934 (w), 2860 (w), 1890 (w), 1801 (w), 1727 (s), 1606 (w), 1564 (w), 1458 (w), 1436 (m), 1408 (w), 1370 (w), 1335 (w), 1279 (s), 1248 (s), 1185 (m), 1107 (m), 1064 (m), 1019 (w), 975 (w), 861 (w), 833 (w), 772 (w), 736 (w); **HRMS** (ESI/QTOF) m/z: $[M + Na]^+$ Calcd for $C_{27}H_{32}NaO_8^+$ 507.1989; Found 507.1999.

4.2.9. Synthesis and characterization of 2'-ethyl 2,2-dimethyl 3'-hexyl-3-(naphthalen-1-yl)-[1,1'-bi(cyclopropane)]-3,3'-diene-2,2,2'-tricarboxylate (3x)

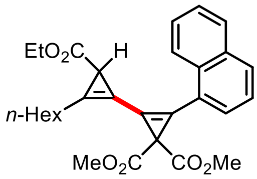

3x

Following **GPD**, a mixture of $(Me_2S)AuCl$ (1.47 mg, 5.00 µmol, 5.00 mol%), $AgNTf_2$ (1.94 mg, 5.00 µmol, 5.00 mol%), **L1** (5.25 mg, 25.0 µmol, 25.0 mol%), CpBX **2a** (73.4 mg, 130 µmol, 1.30 equiv.), terminal cyclopropene **1j** (28.2 mg, 100 µmol, 1.00 equiv.) and CH_3CN (2.0 mL) was stirred at 40 °C for 17 hours. Flash column chromatography on silica gel (eluent: pentane/ethyl acetate = 7:1) afforded **3x** in 86% yield (41.1 mg, 86.2 µmol) as a colorless oil and **4** in 99% NMR yield. **TLC**: R_f (*n*-hexane/EtOAc = 4:1) = 0.41; **^{1}H NMR** (400 MHz, $CDCl_3$) δ 8.40 (dd, *J* = 8.4, 1.2 Hz, 1H, Ar*H*), 7.92 (ddt, *J* = 15.4, 8.0, 1.0 Hz, 2H, Ar*H*), 7.79 (dd, *J* = 7.2, 1.2 Hz, 1H, Ar*H*), 7.66 (ddd, *J* = 8.3, 6.8, 1.4 Hz, 1H, Ar*H*), 7.61 – 7.47 (m, 2H, Ar*H*), 4.18 (q, *J* = 7.1 Hz, 2H, $CO_2CH_2CH_3$), 3.741 (s, 3H, CO_2CH_3), 3.737 (s, 3H, CO_2CH_3), 2.79 (s, 1H, $CHCO_2$), 2.81 – 2.69 (m, 2H, CH_2CH_2C), 1.75 (p, *J* = 7.3 Hz, 2H, CH_2CH_2C), 1.48 – 1.40 (m, 2H, CH_2), 1.36 – 1.29 (m, 4H, CH_2), 1.24 (t, *J* = 7.1 Hz, 3H, $CO_2CH_2CH_3$), 0.92 – 0.88 (m, 3H, CH_3); **^{13}C NMR** (101 MHz, $CDCl_3$) δ 174.1, 170.2, 170.0, 133.5, 132.0, 131.8, 131.2, 128.8, 127.9, 126.9, 125.7, 125.0, 121.8, 119.1, 106.5, 95.6, 93.3, 60.7, 52.6, 52.5, 34.1, 31.6, 28.9, 26.9, 26.3, 24.1, 22.6, 14.5, 14.2; **IR** (ν_{max}, cm^{-1}) 3054 (w), 2954 (m), 2933 (m), 2859 (w), 1887 (w), 1796 (w), 1729 (s), 1588 (w), 1509 (w), 1460 (w), 1435 (m), 1395 (w), 1370 (w), 1332 (w), 1285 (s), 1247 (s), 1183 (s), 1141 (w), 1064 (s), 978 (w), 923 (w), 861 (w), 806 (m), 775 (m), 737 (w); **HRMS** (ESI/QTOF) m/z: $[M + Na]^+$ Calcd for $C_{29}H_{32}NaO_6^+$ 499.2091; Found 499.2100.

4.2.10. Synthesis and characterization of 2'-ethyl 2,2-dimethyl 3'-hexyl-3-(thiophen-3-yl)-[1,1'-bi(cyclopropane)]-3,3'-diene-2,2,2'-tricarboxylate (3y)

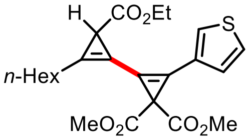

3y

Following **GPD**, a mixture of $(Me_2S)AuCl$ (1.47 mg, 5.00 µmol, 5.00 mol%), $AgNTf_2$ (1.94 mg, 5.00 µmol, 5.00 mol%), **L1** (5.25 mg, 25.0 µmol, 25.0 mol%), CpBX **2a** (84.7 mg, 150 µmol, 1.50 equiv.), terminal cyclopropene **1k** (23.8 mg, 100 µmol, 1.00 equiv.) and CH_3CN (2.0 mL) was stirred at 40 °C for 22 hours. Flash column chromatography on silica gel (eluent: pentane/ethyl acetate = 7:1) afforded **3y** in 44% yield (19.1 mg, 44.2 µmol) as a colorless oil and **4** in 56% NMR yield. **TLC**: R_f (*n*-hexane/EtOAc = 4:1) = 0.39; **^{1}H NMR** (400 MHz, $CDCl_3$) δ 7.68 (dd, *J* = 3.0, 1.2 Hz, 1H, Ar*H*), 7.38 (dd, *J* = 5.0, 3.0 Hz, 1H, Ar*H*), 7.30 (dd, *J* = 5.0, 1.2 Hz, 1H, Ar*H*), 4.15 (q, *J* = 7.1 Hz, 2H, $CO_2CH_2CH_3$), 3.73 (s, 3H, CO_2CH_3), 3.72 (s, 3H, CO_2CH_3), 2.70 – 2.61 (m, 2H, CH_2CH_2C), 2.55 (s, 1H, $CHCO_2$), 1.73 – 1.63 (m, 2H, CH_2CH_2C), 1.48 – 1.27 (m, 6H, CH_2), 1.24 (t, *J* = 7.1 Hz, 3H, $CO_2CH_2CH_3$), 0.94 – 0.84 (m, 3H, CH_3); **^{13}C NMR** (101 MHz, $CDCl_3$) δ 174.3, 170.1, 170.0, 130.2, 128.4, 127.2, 125.8, 118.9, 104.0, 92.7, 92.1, 60.6, 52.6 (2C), 35.4, 31.6, 28.9, 26.9, 26.2, 23.8, 22.6, 14.5, 14.2; **IR** (ν_{max}, cm^{-1}) 3109 (w), 2954 (m), 2932 (m), 2859 (w), 1807 (w), 1727 (s), 1604 (w), 1512 (w), 1460 (w), 1435 (m), 1370 (w), 1332 (w), 1283 (s), 1246 (s), 1182 (s), 1109 (w), 1063 (s), 1029 (w), 979 (w), 866 (m), 791 (m), 730 (w); **HRMS** (ESI/QTOF) m/z: $[M + Na]^+$ Calcd for $C_{23}H_{28}NaO_6S^+$ 455.1499; Found 455.1511.

4.2.11. Synthesis and characterization of 2'-ethyl 2,2-dimethyl 3,3'-dihexyl-[1,1'-bi(cyclopropane)]-3,3'-diene-2,2,2'-tricarboxylate (3z)

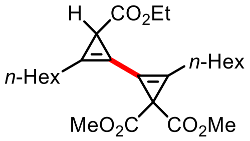

3z

Following **GPD**, a mixture of (Me_2S)AuCl (1.47 mg, 5.00 µmol, 5.00 mol%), $AgNTf_2$ (1.94 mg, 5.00 µmol, 5.00 mol%), **L1** (5.25 mg, 25.0 µmol, 25.0 mol%), CpBX **2a** (113 mg, 200 µmol, 2.00 equiv.), terminal cyclopropene **1l** (24.0 mg, 100 µmol, 1.00 equiv.) and CH_3CN (2.0 mL) was stirred at 40 °C for 28 hours. Flash column chromatography on silica gel (eluent: pentane/ethyl acetate = 7:1) afforded **3z** in 57% yield (24.7 mg, 56.8 µmol) as a colorless oil and **4** in 64% NMR yield. **TLC**: R_f (*n*-hexane/EtOAc = 4:1) = 0.47; **^{1}H NMR** (400 MHz, $CDCl_3$) δ 4.11 (q, *J* = 7.1 Hz, 2H, $CO_2CH_2CH_3$), 3.70 (s, 3H, CO_2CH_3), 3.69 (s, 3H, CO_2CH_3), 2.67 – 2.51 (m, 4H, CH_2CH_2C), 2.42 (s, 1H, $CHCO_2$), 1.64 – 1.57 (m, 4H, CH_2CH_2C), 1.48 – 1.11 (m, 15H, CH_2 & $CO_2CH_2CH_3$), 0.87 (t, *J* = 6.7 Hz, 6H, CH_3); **^{13}C NMR** (101 MHz, $CDCl_3$) δ 174.4, 170.9, 170.7, 117.3, 112.8, 93.8, 92.2, 60.5, 52.3, 52.3, 35.3, 31.6, 31.5, 28.9, 28.8, 26.8, 26.7, 25.8, 24.7, 23.6, 22.6 (2C), 14.4, 14.2 (2C); **IR** (ν_{max}, cm^{-1}) 2955 (m), 2932 (m), 2860 (w), 1818 (w), 1730 (s), 1462 (w), 1436 (w), 1368 (w), 1333 (w), 1282 (m), 1245 (s), 1182 (s), 1105 (w), 1065 (m), 1025 (w), 943 (w), 837 (w), 730 (w); **HRMS** (ESI/QTOF) m/z: [M + Na]$^+$ Calcd for $C_{25}H_{38}NaO_6^+$ 457.2561; Found 457.2560.

4.2.12. Synthesis and characterization of triethyl 3'-hexyl-3-phenyl-[1,1'-bi(cyclopropane)]-3,3'-diene-2,2,2'-tricarboxylate (3aa)

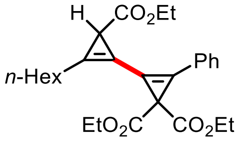

3aa

Following **GPD**, a mixture of (Me_2S)AuCl (1.47 mg, 5.00 µmol, 5.00 mol%), $AgNTf_2$ (1.94 mg, 5.00 µmol, 5.00 mol%), **L1** (5.25 mg, 25.0 µmol, 25.0 mol%), CpBX **2a** (73.4 mg, 130 µmol, 1.30 equiv.), terminal cyclopropene **1m** (26.0 mg, 100 µmol, 1.00 equiv.) and CH_3CN (2.0 mL) was stirred at 40 °C for 24 hours. Flash column chromatography on silica gel (eluent: pentane/ethyl acetate = 8:1) afforded **3aa** in 43% yield (19.7 mg, 43.3 µmol) as a colorless oil and **4** in 89% NMR yield. **TLC**: R_f (*n*-hexane/EtOAc = 4:1) = 0.51; **^{1}H NMR** (400 MHz, $CDCl_3$) δ 7.68 – 7.61 (m, 2H, Ar*H*), 7.49 – 7.40 (m, 3H, Ar*H*), 4.36 – 3.99 (m, 6H, $CO_2CH_2CH_3$), 2.74 – 2.64 (m, 2H, CH_2CH_2C), 2.61 (s, 1H, $CHCO_2$), 1.79 – 1.68 (m, 2H, CH_2CH_2C), 1.47 – 1.17 (m, 15H, CH_2 & $CO_2CH_2CH_3$), 0.91 (t, *J* = 6.7 Hz, 3H, CH_3); **^{13}C NMR** (101 MHz, $CDCl_3$) δ 174.3, 169.9, 169.6, 130.6 (2C), 129.0, 124.9, 119.1, 109.4, 95.0, 92.9, 61.4, 61.3, 60.5, 35.9, 31.6, 29.0, 27.0, 26.2, 24.0, 22.6, 14.5, 14.29, 14.27, 14.2; **IR** (ν_{max}, cm^{-1}) 2955 (w), 1889 (w), 1805 (w), 1725 (s), 1435 (m), 1369 (w), 1333 (s), 1246 (s), 1182 (s), 1061 (s), 1025 (w), 975 (w), 940 (w), 764 (m); **HRMS** (ESI/QTOF) m/z: [M + Na]$^+$ Calcd for $C_{27}H_{34}NaO_6^+$ 477.2248; Found 477.2244.

4.2.13. Synthesis and characterization of 2,2-dibenzyl 2'-ethyl 3'-hexyl-3-phenyl-[1,1'-bi(cyclopropane)]-3,3'-diene-2,2,2'-tricarboxylate (3ab)

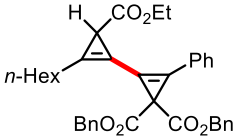

3ab

Following **GPD**, a mixture of (Me_2S)AuCl (1.47 mg, 5.00 µmol, 5.00 mol%), $AgNTf_2$ (1.94 mg, 5.00 µmol, 5.00 mol%), **L1** (5.25 mg, 25.0 µmol, 25.0 mol%), CpBX **2a** (73.4 mg, 130 µmol, 1.30 equiv.), terminal

cyclopropene **1n** (38.4 mg, 100 μmol, 1.00 equiv.) and CH_3CN (2.0 mL) was stirred at 40 °C for 18 hours. Flash column chromatography on silica gel (eluent: pentane/ethyl acetate = 8:1) afforded **3ab** in 33% yield (19.3 mg, 33.4 μmol) as a colorless oil and **4** in 72% NMR yield. **TLC**: R_f (*n*-hexane/EtOAc = 5:1) = 0.34; **^{1}H NMR** (400 MHz, $CDCl_3$) δ 7.57 – 7.48 (m, 2H, Ar*H*), 7.38 – 7.28 (m, 3H, Ar*H*), 7.22 – 7.16 (m, 10H, Ar*H*), 5.20 – 5.02 (m, 4H, OCH_2Ph), 4.10 – 3.99 (m, 2H, $CO_2CH_2CH_3$), 2.50 (s, 1H, $CHCO_2$), 2.58 – 2.43 (m, 2H, CH_2CH_2C), 1.58 – 1.45 (m, 2H, CH_2CH_2C), 1.29 – 1.08 (m, 9H, CH_2 & $CO_2CH_2CH_3$), 0.78 (t, *J* = 6.9 Hz, 3H, CH_3); **^{13}C NMR** (101 MHz, $CDCl_3$) δ 174.2, 169.6, 169.3, 136.01, 135.97, 130.8, 130.7, 129.1, 128.53, 128.51, 128.1, 128.0, 127.8, 127.7, 124.7, 119.4, 108.8, 94.8, 92.8, 67.0, 66.9, 60.6, 36.0, 31.5, 29.0, 26.9, 26.1, 24.0, 22.6, 14.5, 14.2; **IR** (ν_{max}, cm^{-1}) 3065 (w), 3033 (w), 2955 (w), 2932 (m), 2859 (w), 1893 (w), 1804 (w), 1727 (s), 1605 (w), 1497 (w), 1455 (w), 1372 (w), 1335 (w), 1272 (s), 1232 (m), 1183 (m), 1055 (m), 1028 (w), 914 (w), 760 (m), 739 (m); **HRMS** (ESI/QTOF) m/z: $[M + Na]^+$ Calcd for $C_{37}H_{38}NaO_6^+$ 601.2561; Found 601.2579.

4.2.14. Synthesis and characterization of 2'-ethyl 2,2-dimethyl 3'-hexyl-2'-methyl-3-phenyl-[1,1'-bi(cyclopropane)]-3,3'-diene-2,2,2'-tricarboxylate (3ac)

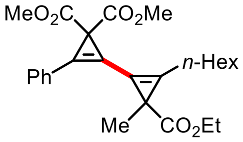

3ac

Following **GPD**, a mixture of $(Me_2S)AuCl$ (2.94 mg, 10.0 μmol, 10.0 mol%), $AgNTf_2$ (3.88 mg, 10.0 μmol, 10.0 mol%), **L1** (10.51 mg, 50.0 μmol, 50.0 mol%), CpBX **2k** (120 mg, 200 μmol, 2.00 equiv.), terminal cyclopropene **1o** (21.0 mg, 100 μmol, 1.00 equiv.) and CH_3CN (2.0 mL) was stirred at 40 °C for 16 hours. Flash column chromatography on silica gel (eluent: pentane/ethyl acetate = 7:1) afforded **3ac** in 13% yield (5.8 mg, 13 μmol) as a colorless oil and **4** in 62% NMR yield. **TLC**: R_f (*n*-hexane/EtOAc = 4:1) = 0.46; **^{1}H NMR** (400 MHz, $CDCl_3$) δ 7.63 – 7.54 (m, 2H, Ar*H*), 7.49 – 7.38 (m, 3H, Ar*H*), 4.10 (q, *J* = 7.1 Hz, 2H, $CO_2CH_2CH_3$), 3.72 (s, 6H, CO_2CH_3), 2.69 – 2.60 (m, 2H, CH_2CH_2C), 1.76 – 1.62 (m, 2H, CH_2CH_2C), 1.50 (s, 3H, $C(CH_3)$), 1.46 – 1.37 (m, 2H, CH_2), 1.35 – 1.27 (m, 4H, CH_2), 1.18 (t, *J* = 7.1 Hz, 3H, $CO_2CH_2CH_3$), 0.94 – 0.84 (m, 3H, CH_3); **^{13}C NMR** (101 MHz, $CDCl_3$) δ 175.6, 170.22, 170.21, 130.6, 130.5, 129.2, 125.7, 124.9, 108.7, 98.5, 94.7, 60.7, 52.52, 52.48, 35.3, 31.6, 29.0, 28.5, 27.1, 25.5, 22.7, 19.3, 14.4, 14.2; **IR** (ν_{max}, cm^{-1}) 2954 (m), 2931 (m), 2860 (w), 1890 (w), 1800 (w), 1733 (s), 1721 (s), 1490 (w), 1446 (m), 1436 (m), 1371 (w), 1279 (s), 1245 (s), 1192 (w), 1115 (s), 1064 (s), 1027 (w), 977 (w), 950 (w), 921 (w), 868 (w), 797 (w), 764 (m), 735 (w); **HRMS** (ESI/QTOF) m/z: $[M + Na]^+$ Calcd for $C_{26}H_{32}NaO_6^+$ 463.2091; Found 463.2102.

4.2.15. Attempt for the Synthesis of 2'-ethyl 2,2-dimethyl 3'-hexyl-3-(4-methoxyphenyl)-[1,1'-bi(cyclopropane)]-3,3'-diene-2,2,2'-tricarboxylate (3ad)

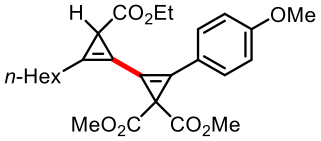

3ad

Following **GPD**, a mixture of $(Me_2S)AuCl$ (1.47 mg, 5.00 μmol, 5.00 mol%), $AgNTf_2$ (1.94 mg, 5.00 μmol, 5.00 mol%), **L1** (5.25 mg, 25.0 μmol, 25.0 mol%), CpBX **2a** (73.4 mg, 130 μmol, 1.30 equiv.), terminal cyclopropene **1p** (26.2 mg, 100 μmol, 1.00 equiv.) and CH_3CN (2.0 mL) was stirred at 40 °C for 14 hours. TLC showed the cyclopropene was mostly remained. Flash column chromatography on silica gel (eluent: pentane/ethyl acetate = 4:1) afforded no desired product **3ad**. The terminal cyclopropene **1y** was recovered in 98% NMR yield and **4** was determined in 64% NMR yield.

5. Transformations of Products and Applications

5.1. Diels–Alder Reaction of 3a with 2,3-Dimethylbutadiene[16]

5.1.1. Synthesis and characterization of dimethyl 2-(7-(ethoxycarbonyl)-6-hexyl-3,4-dimethylbicyclo[4.1.0]hept-3-en-1-yl)-3-phenylcycloprop-2-ene-1,1-dicarboxylate (5)

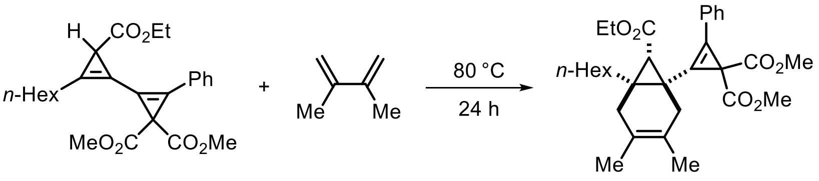

3a **5**

A solution of **3a** (21.3 mg, 50.0 μmol, 1.00 equiv.) in 2,3-dimethylbutadiene (0.50 mL) was heated at 80 °C in a sealed tube for 24 hours. After being cooled to room temperature, the mixture was concentrated and purified by column chromatography on silica gel (eluent: pentane/EtOAc = 10:1) to give the cycloaddition product **5** in 37% yield (9.4 mg, 18 μmol, dr. >20:1 based on ^{1}H NMR) as a colorless oil. **TLC**: R_f (*n*-hexane/EtOAc = 4:1) = 0.42; **^{1}H NMR** (400 MHz, $CDCl_3$) δ 7.77 – 7.65 (m, 2H, Ar*H*), 7.45 – 7.30 (m, 3H, Ar*H*), 4.09 – 4.02 (m, 2H, $CO_2CH_2CH_3$), 3.71 (s, 3H, CO_2CH_3), 3.69 (s, 3H, CO_2CH_3), 2.63 – 2.52 (m, 2H, C*H*$_2$(allylic)), 2.41 – 2.29 (m, 2H, C*H*$_2$(allylic)), 2.18 (s, 1H, C*H*(cyclopropyl)), 2.17 – 2.06 (m, 1H, CH_2CH_2-△), 1.83 – 1.75 (m, 1H, CH_2CH_2-△), 1.61 (s, 6H, C=CC*H*$_3$), 1.51 – 1.42 (m, 1H, CH_2CH_2-△), 1.27 – 1.15 (m, 10H, C*H*$_2$ & $CO_2CH_2CH_3$ & CH_2CH_2-△), 0.91 – 0.75 (m, 3H, C*H*$_3$); **^{13}C NMR** (101 MHz, $CDCl_3$) δ 171.8, 171.5, 170.3, 130.5, 129.5, 128.8, 125.5, 122.1, 121.8, 107.8, 104.5, 60.4, 52.2 (2C), 36.8, 36.6, 36.2, 35.1, 32.0, 31.3, 30.8, 30.6, 29.7, 26.5, 22.7, 19.2, 18.9, 14.4, 14.2; **IR** (ν_{max}, cm^{-1}) 2937 (w), 2926 (w), 2860 (w), 1732 (s), 1436 (w), 1278 (m), 1241 (m), 1168 (m), 1064 (m), 914 (w), 835 (w), 765 (w), 733 (w); **HRMS** (ESI/QTOF) m/z: [M + Na]$^+$ Calcd for $C_{31}H_{40}NaO_6^+$ 531.2717; Found 531.2719.

5.2. Rh(I)-Catalyzed Cycloisomerization of 3a[17]

5.2.1. Synthesis and characterization of methyl 5'-ethoxy-3'-hexyl-5-methoxy-3-phenyl-[2,2'-bifuran]-4-carboxylate (6)

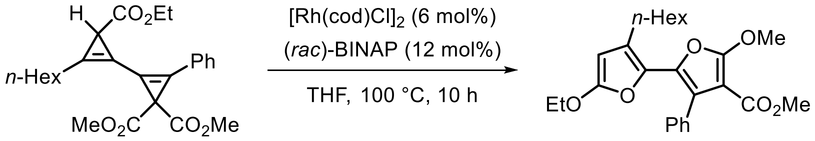

3a **6**

An oven-dried 8 mL microwave reaction vial was sequentially charged with **3a** (21.3 mg, 50.0 μmol, 1.00 equiv.), $[Rh(cod)Cl]_2$ (1.48 mg, 3.00 μmol, 6.00 mol%), (*rac*)-BINAP (3.74 mg, 6.00 μmol, 12.0 mol%) and a magnetic stirring bar. The vial was then evacuated and backfilled with nitrogen three times. Subsequently, THF (0.05 M; 1.0 mL) was added under N_2. The vial was capped tightly, and the reaction mixture was stirred at 100 °C for 10 hours. The resulting reaction mixture was then filtered through a silica gel pad and eluted with CH_2Cl_2 (3 × 5.0 mL). The solvent was removed under reduced pressure, and the resulting crude residue was purified by flash column chromatography on silica gel (eluent: pentane/EtOAc = 10:1) to give the cycloisomerization product **6** in 38% yield (8.1 mg, 19 μmol) as a colorless oil. **TLC**: R_f (*n*-hexane/EtOAc = 10:1) = 0.23; **^{1}H NMR** (400 MHz, $CDCl_3$) δ 7.49 – 7.32 (m, 5H, Ar*H*), 6.16 (s, 1H, Ar*H*), 4.17 (q, *J* = 7.1 Hz, 2H, OCH_2CH_3), 3.86 (s, 3H, OCH_3), 3.60 (s, 3H, OCH_3), 2.87 – 2.68 (m, 2H, CH_2CH_2C), 1.66 – 1.53 (m, 2H, CH_2CH_2C), 1.40 – 1.20 (m, 9H, C*H*$_2$ & OCH_2CH_3), 0.92 – 0.80 (m, 3H, C*H*$_3$); **^{13}C NMR** (101 MHz, $CDCl_3$) δ 165.9, 165.5, 163.7, 141.9, 136.8, 136.6, 131.5, 129.9, 128.6, 128.0, 126.3, 104.6, 91.4, 60.4, 52.72, 52.68, 32.0, 31.7, 29.1, 28.5, 22.7, 14.4, 14.2; **IR** (ν_{max}, cm^{-1}) 2954 (w), 2930 (w), 2859 (w), 1734 (s), 1714 (s), 1608 (m), 1570 (w), 1435 (m), 1335 (w), 1239 (m), 1152

(s), 1118 (s), 1087 (m), 1045 (w), 877 (w), 764 (w); **HRMS** (ESI/QTOF) m/z: [M + Na]$^+$ Calcd for $C_{25}H_{30}NaO_6^+$ 449.1935; Found 449.1941.

5.3. Rh(I)-Catalyzed Cycloisomerization of 3k[17]

5.3.1. Synthesis and characterization of dimethyl 5,5'-dimethoxy-3,3'-diphenyl-[2,2'-bifuran]-4,4'-dicarboxylate (7)

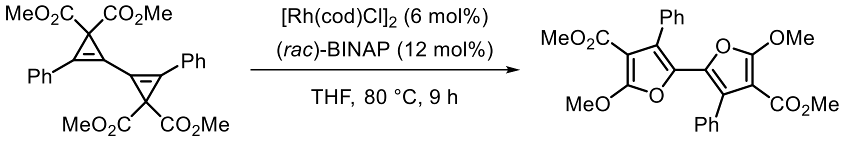

3k **7**

An oven-dried 8 mL microwave reaction vial was sequentially charged with **3k** (23.1 mg, 50.0 µmol, 1.00 equiv.), $[Rh(cod)Cl]_2$ (1.48 mg, 3.00 µmol, 6.00 mol%), (*rac*)-BINAP (3.74 mg, 6.00 µmol, 12.0 mol%) and a magnetic stirring bar. The vial was then evacuated and backfilled with nitrogen three times. Subsequently, THF (0.05 M; 1.0 mL) was added under N_2. The vial was capped tightly, and the reaction mixture was stirred at 80 °C for 9 hours. The resulting reaction mixture was then filtered through a silica gel pad and eluted with CH_2Cl_2 (3 × 5.0 mL). The solvent was removed under reduced pressure, and the resulting crude residue was purified by flash column chromatography on silica gel (eluent: pentane/EtOAc = 3:1) to give the cycloisomerization product **7** in 51% yield (11.7 mg, 25.3 µmol) as an off-white amorphous solid. **TLC**: R_f (*n*-hexane/EtOAc = 2:1) = 0.36; **^{1}H NMR** (400 MHz, $CDCl_3$) δ 7.25 – 7.17 (m, 6H, Ar*H*), 7.16 – 7.07 (m, 4H, Ar*H*), 3.66 (s, 6H, OC*H*$_3$), 3.53 (s, 6H, OC*H*$_3$); **^{13}C NMR** (101 MHz, $CDCl_3$) δ 163.2, 162.0, 132.0, 131.2, 130.2, 127.5 (2C), 125.6, 92.0, 57.6, 51.1; **IR** (ν_{max}, cm^{-1}) 2956 (w), 1707 (s), 1598 (s), 1545 (w), 1488 (m), 1466 (w), 1435 (s), 1403 (w), 1315 (w), 1257 (w), 1162 (m), 1103 (s), 998 (m), 951 (w), 915 (w), 806 (w), 780 (w), 769 (w), 727 (w); **HRMS** (ESI/QTOF) m/z: [M + Na]$^+$ Calcd for $C_{26}H_{22}NaO_8^+$ 485.1207; Found 485.1216. The NMR spectroscopic data is consistent with the previous report[18].

5.4. Reduction of 1,1'-Bicyclopropenyl Derivative 3m Using DIBAL-H[19]

5.4.1. Synthesis and characterization of 3-(4-bromophenyl)-2,7-bis(hydroxymethyl)-6-phenylocta-2,6-dien-4-yne-1,8-diol (8)

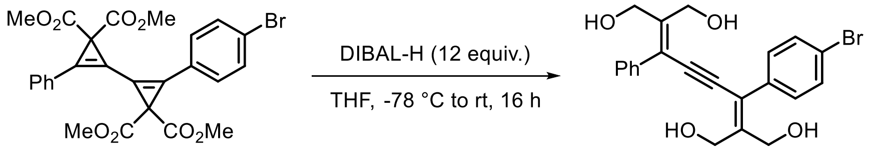

3m **8**

A 25 mL Schlenk tube was charged with **3m** (27.1 mg, 50.0 µmol, 1.00 equiv.) and a magnetic stirring bar. The Schlenk tube was then evacuated and backfilled with nitrogen three times. Subsequently, THF (0.025 M; 2.0 mL) was added by syringe and the Schlenk tube was placed at −78 °C in a dry ice/acetone bath. After that, diisobutylaluminum hydride (DIBAL-H, 1.0 M in cyclohexane; 600 µmol, 0.60 mL, 12.0 equiv.) was added dropwise by a syringe pump over 5 min and then the reaction mixture was stirred in a dry ice/acetone bath. The reaction mixture was allowed to warm to room temperature naturally over 16 hours while keeping stirring. The reaction mixture was then quenched by adding saturated aqueous NH_4Cl (2.0 mL) and extracted with EtOAc (5.0 mL × 3). The combined organic layers were successively washed with brine (10 mL) and H_2O (10 mL), then dried over anhydrous Na_2SO_4, filtered, and concentrated under reduced pressure. The residue was purified by column chromatography on silica gel (eluent: EtOAc) to give the ring-opening product **8** in 21% yield (4.5 mg, 10 µmol) as a colorless oil. **TLC**: R_f (EtOAc) = 0.15; **^{1}H NMR** (400 MHz, DMSO-*d*$_6$) δ 7.60 – 7.56 (m, 2H, Ar*H*), 7.43 – 7.30 (m, 7H, Ar*H*), 4.89 (bs, 4H, CH_2O*H*), 4.39 (s, 2H, C*H*$_2$OH), 4.38 (s, 2H, C*H*$_2$OH), 4.02 (s, 2H, C*H*$_2$OH), 4.00 (s, 2H,

CH_2OH); **^{13}C NMR** (101 MHz, DMSO-d_6) δ 149.1, 148.7, 137.5, 136.8, 131.2, 130.8, 128.6, 128.2, 127.7, 121.1, 121.0, 120.1, 94.0, 93.1, 60.6, 60.5, 57.1, 56.9; **IR** (ν_{max}, cm^{-1}) 3264 (s), 2923 (s), 2853 (m), 1709 (m), 1485 (w), 1460 (w), 1361 (w), 1331 (w), 1259 (w), 1223 (w), 1057 (m), 1008 (s), 829 (w), 767 (w); **HRMS** (ESI/QTOF) m/z: [M + Na]$^+$ Calcd for $C_{22}H_{21}BrNaO_4^+$ 451.0515; Found 451.0500.

6. Mechanistic Investigations

6.1 Preparation of Cationic Gold(I)-Ethylene Complex (9)

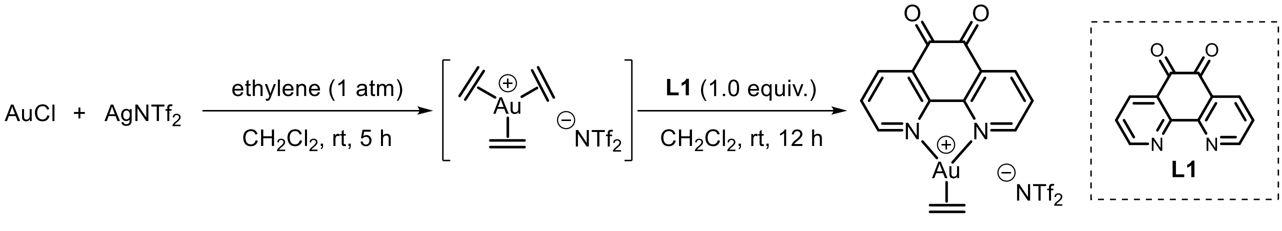

In a nitrogen-filled glovebox,[20] a 100 ml Schlenk tube was sequentially charged with AuCl (93.0 mg, 0.400 mmol, 1.00 equiv.) and a magnetic stir bar then wrapped with foil to protect the contents from light. The Schlenk tube was then sealed with a rubber cap and brought out of the glovebox. Subsequently, the Schlenk tube was evacuated and backfilled with ethylene gas for three times by a balloon filled with ethylene (ca. 1 atm.). To the Schlenk tube was added dry CH_2Cl_2 (2.0 mL). The resulting solution was stirred for 5 min at room temperature. After that, a solution of $AgNTf_2$ (155 mg, 0.400 mmol, 1.00 equiv.) in CH_2Cl_2 (25 mL) was added via a syringe pump over 30 min. After the addition was complete, the mixture was stirred for additional 4.5 hours at room temperature. The resulting suspension[21] was filtered through a syringe filter (membrane-⌀: 25 mm; pore size: 0.2 μm). The filtrate was directly introduced into another 100 ml Schlenk tube (wrapped with aluminium foil and filled with nitrogen) charged with **L1** (84.1 mg, 0.400 mmol, 1.00 equiv.) and a magnetic stir bar. The resulting mixture was stirred at room temperature for 12 hours and then filtered through a syringe filter (membrane-⌀: 25 mm; pore size: 0.2 μm). The filtrate was directly introduced into a third 100 ml Schlenk tube (wrapped with aluminium foil and filled with nitrogen). Dry Et_2O (25 mL) was added slowly as a layer. The Schlenk tube was kept standing at room temperature with a balloon filled with nitrogen for 24 h, which gave a crystalline solid. After decanting the solvent, the solid was washed with Et_2O (3 × 10 mL) and dried in vacuo to afford the gold(I)-ethylene complex **9** (107 mg, 149 μmol, 37% yield) as a light-yellow crystal. **^{1}H NMR** (400 MHz, CD_2Cl_2) δ 9.09 (d, *J* = 4.9 Hz, 2H, Ar*H*), 8.83 (dd, *J* = 8.0, 1.6 Hz, 2H, Ar*H*), 8.06 (dd, *J* = 8.0, 5.2 Hz, 2H, Ar*H*), 4.01 (s, 4H, CH_2=CH_2); **^{13}C NMR** (101 MHz, CD_2Cl_2) δ 175.2, 156.8, 151.2, 141.1, 130.2, 129.8, 120.0 (q, *J* = 321.6 Hz), 65.0; **^{19}F NMR** (376 MHz, CD_2Cl_2) δ -79.3; **IR** (ν_{max}, cm^{-1}) 3100 (w), 2982 (w), 2939 (w), 2888 (w), 1701 (m), 1577 (m), 1478 (w), 1432 (w), 1350 (s), 1335 (m), 1300 (w), 1194 (s), 1136 (m), 1053 (m), 934 (w), 818 (w), 791 (w), 734 (m), 710 (w); **HRMS** (ESI$^+$) m/z Calcd. for $C_{14}H_{10}AuN_2O_2$ [M-NTf_2]$^+$ 435.0402, found 435.0413; **Anal.** Calcd for $C_{12}H_6N_2O_2$ • $C_2AuF_6NO_4S_2$ • C_2H_4: C, 26.86; H, 1.41; N, 5.87; S, 8.96. Found: C, 28.09; H, 1.47; N, 6.14; S, 8.64.

6.2 Control Experiments for Determining the Catalytically Active Species

An oven-dried 10 mL Schlenk tube was sequentially charged with a magnetic stirring bar, the gold catalyst [(Me_2S)AuCl or **9**; 5.00 μmol, 5.00 mol%], **L1** (20.0 or 25.0 μmol, 20.0 or 25.0 mol%) or additive (if used; NBu_4Cl, 5.00 μmol, 5.00 mol%; Me_2S, 5.00 μmol, 5.00 mol%), terminal cyclopropene **1a** (23.2 mg, 100 μmol, 1.00 equiv.) and CpBX **2a** (73.4 mg, 130 μmol, 1.30 equiv.). The Schlenk tube was then introduced into a nitrogen-filled glovebox and the silver salt (5.00 μmol, 5.00 mol%) was added. The tube was then tightly sealed with a rubber cap and brought out of the glovebox. After that, CH_3CN (0.05 M; 2.0 mL) was added under N_2 atmosphere. The reaction mixture was stirred at the indicated temperature for the specified time. The resulting reaction mixture was diluted with CH_2Cl_2 (5.0 mL) and filtered

through a short pad of silica gel by eluting with CH_2Cl_2 (3 × 5.0 mL). The filtrate was then concentrated to dryness and the residue was subjected to flash column chromatography on silica gel (eluent: pentane/EtOAc = 20:1 to 5:1). The fractions that contained the product **3a**, **4** and the remaining CpBX **2a** or terminal cyclopropene **1a** were collected separately and concentrated by rotary evaporation. The yields of **3a** [^{1}H NMR δ 2.58 (s, 1H)], **4** [^{1}H NMR δ 8.09 (dd, *J* = 8.0, 1.4 Hz, 1H)] and the recoveries of **2a** [^{1}H NMR δ 2.62 (s, 1H)] and **1a** [^{1}H NMR δ 6.87 (s, 1H)] were obtained by quantitative ^{1}H NMR analysis using CH_2Br_2 [^{1}H NMR δ 4.92 (s, 2H)] as the internal standard. The analysis of the yield of **3a** and the recovery of **1a** are based on the starting **1a**, while the analysis of the yield of **4** and the recovery of **2a** are based on the starting **2a**.

Table S6. Control Experiments for Determining the Catalytically Active Species

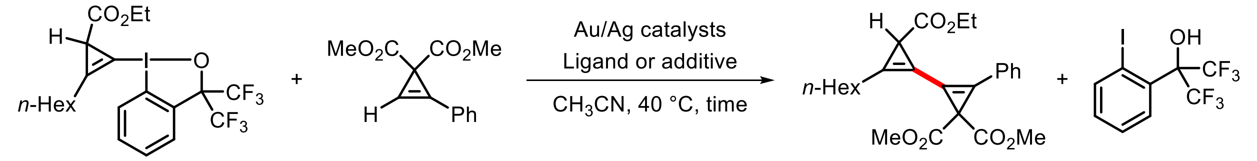

2a **1a** **3a** **4**

Entry	Gold catalyst (5 mol%)	Silver catalyst (5 mol%)	Ligand or additive (mol%)	Time (h)	Yield or recovery (%)[a]			
					**3a**	**1a**	**2a**	**4**
1	$(Me_2S)AuCl$	$AgNTf_2$	**L1** (25)	10	92	<1	8	91
2	**9**	$AgNTf_2$	**L1** (20)	11	2	98	77	23
3	**9**	$AgNTf_2$	**L1** (20), NBu_4Cl (5)	10	27	72	55	45
4	**9**	$AgNTf_2$	**L1** (20), NBu_4Cl (5), Me_2S (5)	10	42	57	42	58
5	$(Me_2S)AuCl$	AgCl	**L1** (25)	11	61	31	5	95

[a]Reactions were performed on a 0.100 mmol scale. Yields or recoveries were determined by ^{1}H NMR spectroscopy using CH_2Br_2 as the internal standard.

These control experiments confirmed the critical role of chloride in this reaction as a supporting ligand of the gold catalyst. The dominant role of cationic silver species in catalyzing the C–H activation of cyclopropenes instead of the neutral ones was also demonstrated.

7. Computational Section

7.1 Computational Methods

Conformers were optimized in Q-Chem v6 at the B3LYP -D3/6-311+G(d,p), SMD(acetonitrile) level of theory.

7.2 Calculated Energies of Selected Strained C_6H_6 Isomers

Table S7. Computed energies of selected strained C_6H_6 isomers at 25 °C at the B3LYP-D3/6-311+G(d,p), SMD(acetonitrile) level of theory.

Structure	EE[a] (Hartrees)	H (kcal/mol)	S (cal/molK)	G (kcal/mol)	EE+H (kcal/mol)	EE+G (kcal/mol)	ΔG_{rel} (kcal/mol)
Benzene	-232.32805	66.064	69.098	45.4624313	-145720.49	-145741.09	0.0
Dewar benzene	-232.19187	64.068	69.475	43.3540288	-145637.03	-145657.74	83.3
Benzvalene	-232.19924	64.006	68.28	43.648318	-145641.71	-145662.07	79.0

Prismane	-232.13059	63.51	67.325	43.4370513	-145599.13	-145619.21	121.9
1,1'-Bicyclopropenyl	-232.13921	63.119	77.426	40.0344381	-145604.93	-145628.01	113.1

[a]EE, electronic energy.

7.3 Structure of 1,1'-Bicyclopropenyl

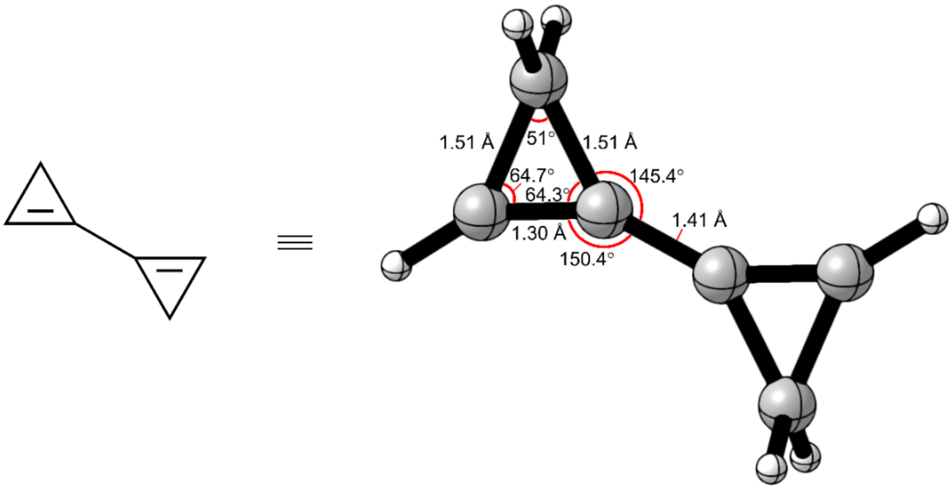

Figure S1. Geometry of 1,1'-bicyclopropenyl at the B3LYP-D3/6-311+G(d,p), SMD(acetonitrile) level of theory.

7.4 Frontier Molecular Orbitals of 1,1'-Bicyclopropenyl

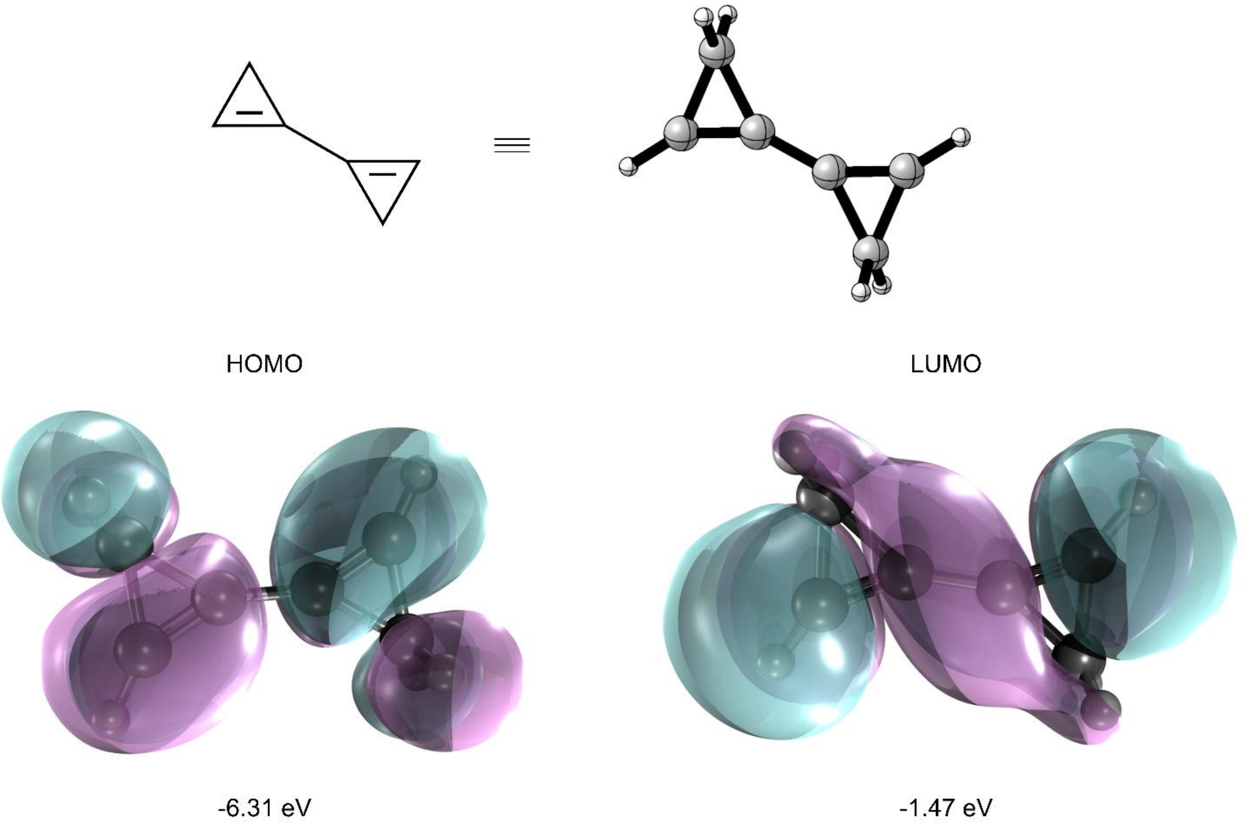

Figure S2. Geometry-optimized structures and frontier molecular orbitals of 1,1'-bicyclopropenyl (B3LYP-D3/6-311+G(d,p), SMD(acetonitrile)).

7.5 Cartesian Coordinates of Optimized Geometries

Benzene

C	0.28015	1.01034	-0.47860
C	-0.96420	0.61554	0.01770
C	1.36457	0.13167	-0.42443
C	-1.12407	-0.65794	0.56850
C	-0.03966	-1.53660	0.62267
C	1.20470	-1.14178	0.12636
H	0.40422	1.99922	-0.90690
H	-1.80639	1.29792	-0.02439
H	2.33090	0.43883	-0.81001
H	-2.09044	-0.96504	0.95406
H	-0.16366	-2.52550	1.05095
H	2.04688	-1.82419	0.16847

Dewar benzene

C	1.18677	-1.81251	1.48694
C	0.31413	-1.65402	0.23889
C	2.11193	-0.93236	1.07969
C	1.40298	-0.61820	-0.24147
C	1.64109	-1.58766	-1.40269
C	0.71603	-2.46765	-0.99484
H	1.09447	-2.45989	2.35205
H	-0.73121	-1.36814	0.36145
H	3.05715	-0.59220	1.48821
H	1.14639	0.41781	-0.46598
H	2.35407	-1.57091	-2.21964
H	0.39147	-3.43800	-1.35421

Prismane

C	-1.89520	2.50443	0.93196
C	-1.82756	1.10155	1.52403
C	-0.34335	2.50911	0.76688
C	-0.27596	1.10650	1.35949
C	-1.73497	2.31863	2.43542
C	-0.18273	2.32446	2.27063
H	-2.69452	2.98037	0.37880
H	-2.56671	0.31113	1.50549
H	0.31870	2.98687	0.05641
H	0.44737	0.32084	1.18389
H	-2.38979	2.62526	3.24078
H	0.62399	2.63715	2.92088

Benzvalene

C	-1.28905	0.78770	-1.06638
C	-1.99785	0.69981	0.28723
C	-0.47536	0.70382	0.12807
C	-2.45480	-0.72403	0.49929
C	-1.57159	-1.49000	-0.15690
C	-0.57548	-0.53324	-0.76865
H	-1.31546	1.47353	-1.90224
H	-2.50935	1.56280	0.69540
H	0.33557	1.30344	0.51868
H	-3.32413	-1.02156	1.07000
H	-1.54153	-2.56715	-0.25057
H	0.27120	-0.84748	-1.36639

1,1'-Bicyclopropenyl

C	-0.55318	0.15000	-0.71899
C	-1.43757	-1.05386	-0.51121
C	-0.51321	-0.88188	0.38865
C	0.13910	-1.29189	1.57017
C	1.06443	-1.12175	2.46888
C	0.17678	-2.32285	2.67900
H	-0.94408	1.14639	-0.50663
H	0.21051	0.12738	-1.49806
H	-2.26879	-1.62160	-0.89722
H	1.90025	-0.55969	2.85333
H	-0.58617	-2.29635	3.45865
H	0.56433	-3.32053	2.46694

8. Attempts of Using Stille Coupling to Synthesize 1,1'-Bicyclopropenes

8.1 Synthesis of Stannylcyclopropene

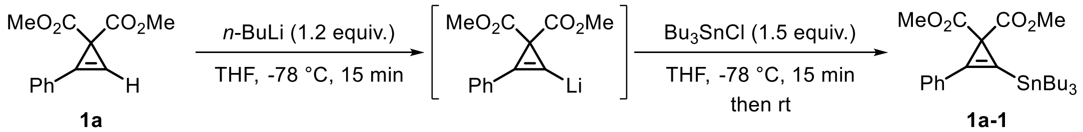

An oven-dried Schlenk tube was charged with a magnetic stirring bar and terminal cyclopropene **1a** (752 mg, 3.24 mmol, 1.00 equiv.). The Schlenk tube was then evacuated and backfilled with nitrogen three times. After that, THF (32 mL, 0.10 M) was added by syringe and the Schlenk tube was placed at −78 °C in a dry ice/acetone bath. *n*-Butyllithium (1.6 M in hexane; 2.4 mL, 3.9 mmol, 1.2 equiv.) was added dropwise by a syringe pump over 5 min and the reaction mixture was stirred at −78 °C for additional 10 min. Then, tributyltin chloride (1.3 mL, 4.9 mmol, 1.5 equiv.) was added in one portion under nitrogen. The reaction mixture was stirred at −78 °C for 15 min, then the cooling bath was removed. The reaction mixture was allowed to warm to room temperature gradually (ca. 15 min) while keeping stirring. The reaction mixture was then quenched by adding saturated aqueous $NaHCO_3$ (10 ml/mmol). The organic layer was removed, and the remaining aqueous portion was extracted with EtOAc. The combined organic portions were dried over Mg_2SO_4 and filtered through Celite®. The solvent was removed under reduced pressure, and the resulting crude residue was purified by flash column chromatography on silica gel (eluent: pentane/EtOAc = 10:1) to give stannylcyclopropene **1a-1** in 65% yield (1.10 g, 2.11 mmol) as a colorless oil. **TLC**: R_f (*n*-hexane/EtOAc = 10:1) = 0.38; **^{1}H NMR** (400 MHz, $CDCl_3$) δ 7.60 – 7.51 (m, 2H, Ar*H*), 7.47 – 7.32 (m, 3H, Ar*H*), 3.67 (s, 6H, OCH_3), 1.64 – 1.54 (m, 6H, CH_2),

1.42 – 1.29 (m, 6H, C*H*$_2$), 1.26 – 1.07 (m, 6H, SnC*H*$_2$), 0.90 (t, *J* = 7.3 Hz, 9H, C*H*$_3$); **^{13}C NMR** (101 MHz, $CDCl_3$) δ 172.7, 129.8, 129.6, 128.9, 126.5, 124.0, 52.1, 33.8, 29.0 ($^3J_{Sn\text{-}C}$ = 22.4 Hz), 27.3 ($^2J_{Sn\text{-}C}$ = 60.0 Hz), 13.8, 11.3 ($^1J_{^{119}Sn\text{-}C}$ = 363.7 Hz, $^1J_{^{117}Sn\text{-}C}$ = 347.5 Hz); **IR** (ν_{max}, cm^{-1}) 2953 (m), 2923 (m), 2871 (w), 2851 (w), 1799 (w), 1720 (s), 1488 (w), 1447 (w), 1432 (m), 1377 (w), 1277 (s), 1231 (s), 1062 (s), 982 (w), 876 (w), 760 (s), 689 (s), 599 (w), 566 (w), 518 (w); **HRMS** (ESI/QTOF) m/z: $[M + H]^+$ Calcd for $C_{25}H_{39}O_4Sn^+$ 523.1865; Found 523.1863. The NMR spectroscopic data is consistent with the previous report[22].

8.2 Synthesis of Cyclopropenyl Iodide

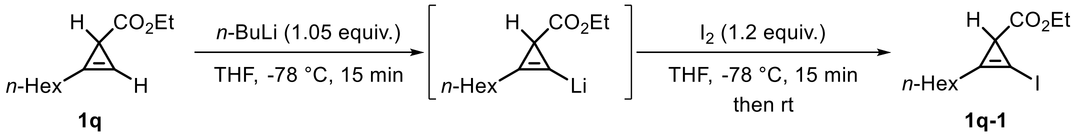

An oven-dried Schlenk tube was charged with a magnetic stirring bar and terminal cyclopropene **1q** (994 mg, 5.06 mmol, 1.00 equiv.). The Schlenk tube was then evacuated and backfilled with nitrogen three times. After that, THF (30 mL) was added by syringe and the Schlenk tube was placed at −78 °C in a dry ice/acetone bath. *n*-Butyllithium (1.6 M in hexane; 3.3 mL, 1.05 equiv.) was added dropwise by a syringe pump over 5 min and the reaction mixture was stirred at −78 °C for additional 10 min. Then, elemental iodine (1.54 g, 6.07 mmol, 1.20 equiv.) was added in one portion under nitrogen. The reaction mixture was stirred at −78 °C for 15 min, then the cooling bath was removed. The reaction mixture was allowed to warm to room temperature gradually (ca. 15 min) while keeping stirring. The reaction mixture was then quenched by adding a sodium bisulfite solution ($NaHSO_3$, ⩾37% in water; 10 mL). The organic layer was separated, and the remaining aqueous portion was extracted with EtOAc. The combined organic portions were dried over Mg_2SO_4 and filtered through Celite®. The solvent was removed under reduced pressure, and the resulting crude residue was purified by flash column chromatography on silica gel (eluent: pentane/EtOAc = 20:1) to give cyclopropenyl iodide **1q-1** in 71% yield (1.17 g, 3.62 mmol) as a colorless liquid. **TLC**: R_f (*n*-hexane/EtOAc = 20:1) = 0.30; **^{1}H NMR** (400 MHz, $CDCl_3$) δ 4.14 (q, *J* = 7.1 Hz, 2H, OC*H*$_2$CH$_3$), 2.49 (t, *J* = 7.2 Hz, 2H, CH$_2$C*H*$_2$C), 2.44 (s, 1H, C*H*CO$_2$), 1.66 – 1.52 (m, 2H, C*H*$_2$CH$_2$C), 1.43 – 1.25 (m, 6H, C*H*$_2$), 1.25 (t, *J* = 7.1 Hz, 3H, OCH$_2$C*H*$_3$), 0.95 – 0.82 (m, 3H, C*H*$_3$); **^{13}C NMR** (101 MHz, $CDCl_3$) δ 174.7, 125.3, 60.7, 46.7, 31.5, 29.0, 26.7, 26.0, 25.0, 22.6, 14.5, 14.2. The NMR spectroscopic data is consistent with the previous report[23].

8.3 Attempts of Stille Coupling Using Stannylcyclopropene and Cyclopropenyl Iodide

The reported Stille coupling conditions of stannylcyclopropenes with a wide selection of electrophiles was chosen for the attempts.[22] A solution of $Pd_2(dba)_3$ (2.3 mg, 2.5 μmol, 2.5 mol%) and $AsPh_3$ (3.1 mg, 10 μmol, 10 mol%) in THF (0.5 mL) was stirred at room temperature under N_2 atmosphere for 15 min. A solution of the stannylcyclopropene **1a-1** (52.1 mg, 100 μmol, 1.00 equiv.) and cyclopropenyl iodide **1q-1** (35.4 mg, 110 μmol, 1.10 equiv.) in THF (0.25 mL + 0.25 mL×3 rinse) was then added via syringe under N_2 and the reaction mixture was stirred at either room temperature, 40 °C, or 60 °C for 12 hours. The reaction mixture was cooled to room temperature (if necessary) and filtered through a short plug of silica gel (ca. 3 cm high, 1 cm diameter) using dichloromethane as the eluent (ca. 10 mL in total). The filtrate was then concentrated in vacuo to afford the crude reaction residue. The yields of **3a** [^{1}H NMR δ 2.58 (s, 1H)] and the recoveries of **1a-1** [^{1}H NMR δ 3.67 (s, 6H)] and **1q-1** [^{1}H NMR δ 2.45 (s, 1H)] were obtained by quantitative ^{1}H NMR analysis of the crude reaction residue using CH_2Br_2 [^{1}H NMR δ 4.93 (s, 2H)] as the internal standard. The analysis of the yield of **3a** and the recovery of **1a-1** are based on the starting **1a-1**, while the analysis of the recovery of **1q-1** is based on the starting **1q-1**.

Table S8. Attempts of Stille Coupling Using Stannylcyclopropene and Cyclopropenyl Iodide

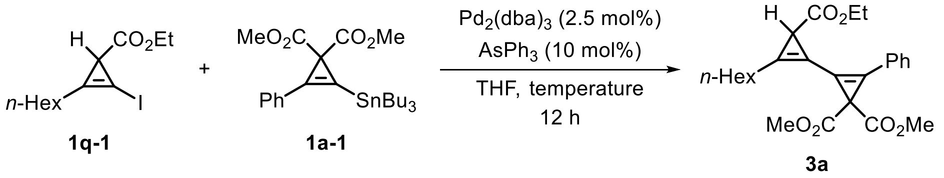

Entry	Temperature	Yield of **3a** (%)[a]	Recovery of **1q-1** (%)[a]	Recovery of **1a-1** (%)[a]
1	RT	0	85	86
2	40 °C	0	75	69
3	60 °C	0	48	29

Reaction conditions: **1q-1** (110 μmol), **1a-1** (100 μmol), $Pd_2(dba)_3$ (2.50 μmol), $AsPh_3$ (10.0 μmol), THF (1.5 mL). [a]Yields or recoveries were determined by ^1H NMR using dibromomethane as the internal standard.

9. Single Crystal X-ray Diffraction Analysis

9.1. Crystal Data and Structure Refinement for 3k

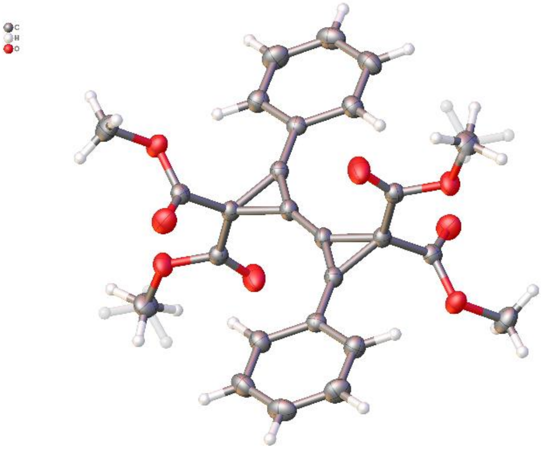

Preparation. Monocrystal suitable for X-ray diffraction analysis was grown by slow evaporation of a solution of **3k** in $CDCl_3$.

Experimental. Single clear pale colorless irregular-shaped crystals of **3k** were used as supplied. A suitable crystal with dimensions 0.58 × 0.34 × 0.21 mm^3 was selected and mounted on a SuperNova, Dual, Cu at home/near, AtlasS2 diffractometer. The crystal was kept at a steady T = 139.99(10) K during data collection. The structure was solved with the ShelXT (Sheldrick, 2015) solution program using dual methods and by using Olex2 1.5 (Dolomanov et al., 2009) as the graphical interface. The model was refined with ShelXL 2019/3 (Sheldrick, 2015) using full matrix least squares minimisation on $\boldsymbol{F}^2$.

Crystal Data. $C_{26}H_{22}O_8$, M_r = 462.43, triclinic, P-1 (No. 2), a = 7.9855(4) Å, b = 8.7411(4) Å, c = 9.4612(5) Å, α = 107.729(4)°, β = 110.526(5)°, γ = 100.586(4)°, V = 557.00(5) Å^3, T = 139.99(10) K, Z = 1, Z' = 0.5, μ(Cu K$_\alpha$) = 0.859, 4601 reflections measured, 2262 unique (R_{int} = 0.0165) which were used in all calculations. The final wR_2 was 0.1115 (all data) and R_1 was 0.0405 (I≥2 σ(I)).

**Compound**	**3k**
CCDC code	2321753
Formula	$C_{26}H_{22}O_8$
$D_{calc.}$/ g cm^{-3}	1.379
μ/mm^{-1}	0.859
Formula Weight	462.43
Colour	clear pale colorless
Shape	irregular-shaped
Size/mm^3	0.58×0.34×0.21
T/K	139.99(10)
Crystal System	triclinic
Space Group	P-1
a/Å	7.9855(4)
b/Å	8.7411(4)
c/Å	9.4612(5)
α/°	107.729(4)
β/°	110.526(5)
γ/°	100.586(4)
V/Å^3	557.00(5)
Z	1
Z'	0.5
Wavelength/Å	1.54184
Radiation type	Cu K$_\alpha$
Θ_{min}/°	5.449
Θ_{max}/°	75.805
Measured Refl's.	4601
Indep't Refl's	2262
Refl's I≥2 σ(I)	2183
R_{int}	0.0165
Parameters	212
Restraints	21
Largest Peak	0.337
Deepest Hole	-0.215
GooF	1.062
wR_2 (all data)	0.1115
wR_2	0.1103
R_1 (all data)	0.0414
R_1	0.0405

10. NMR Spectra

^{1}H NMR (400 MHz, $CDCl_3$) of 1a

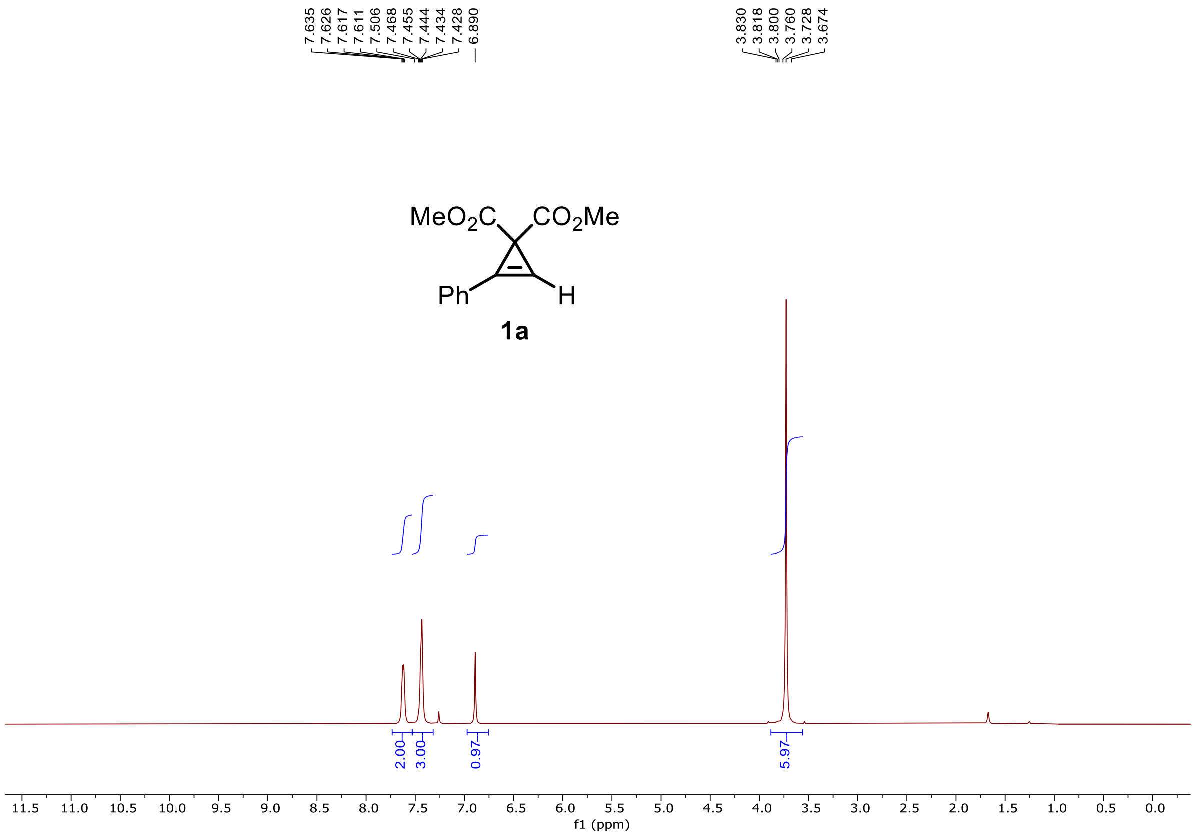

^{13}C NMR (101 MHz, $CDCl_3$) of 1a

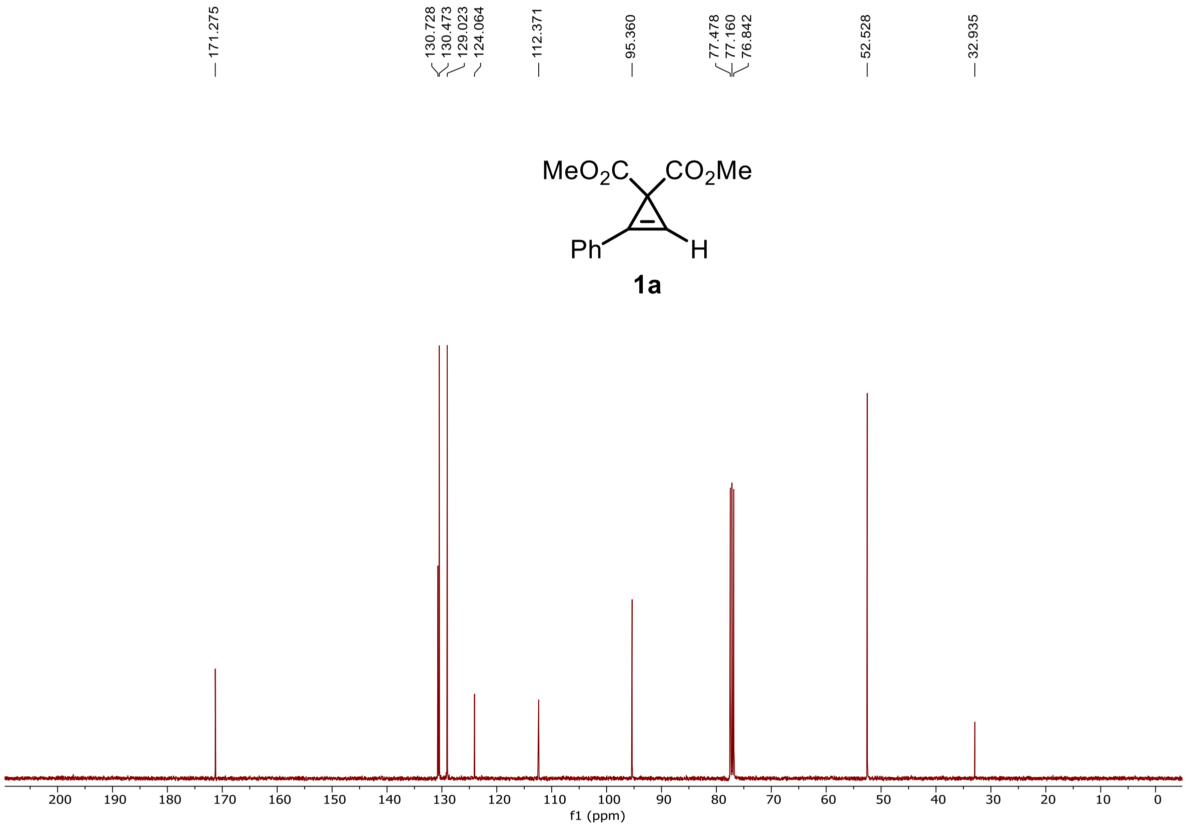

^{1}H NMR (400 MHz, $CDCl_3$) of 1b

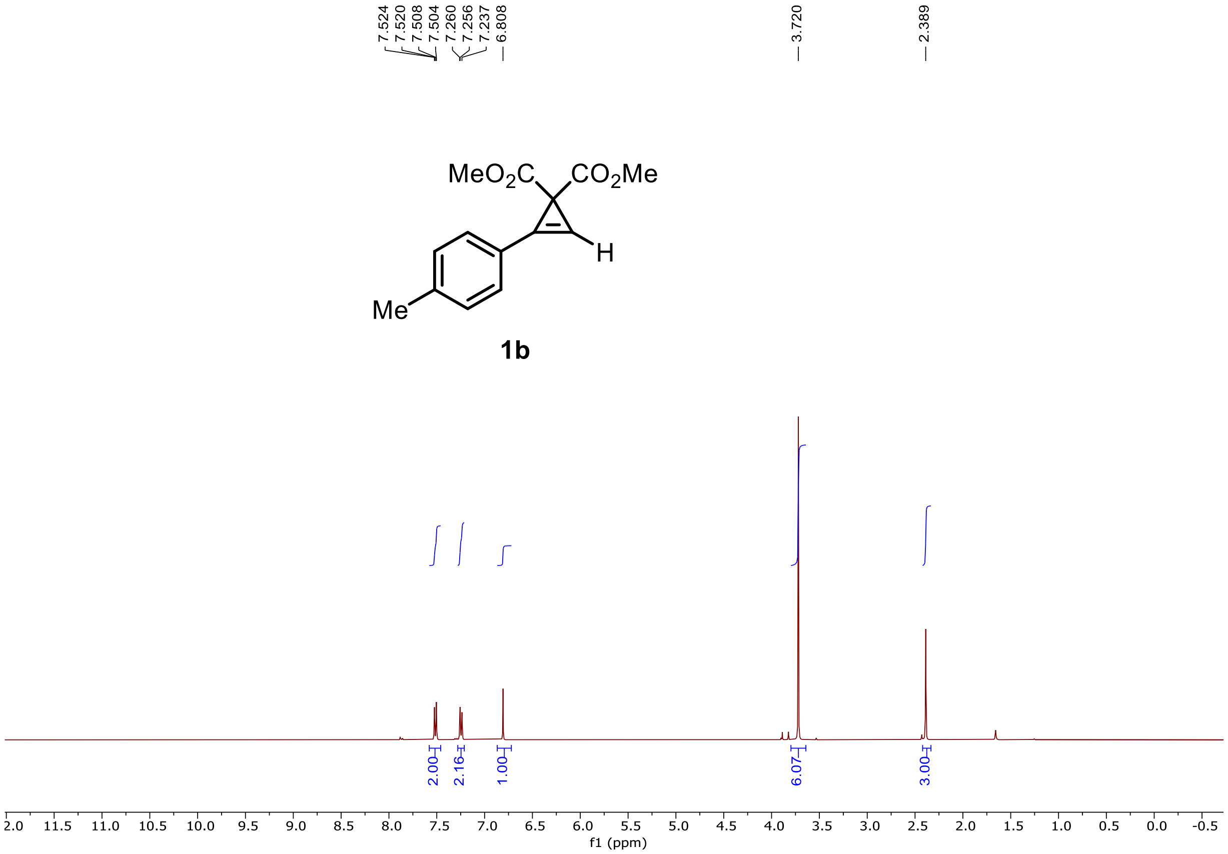

^{13}C NMR (101 MHz, $CDCl_3$) of 1b

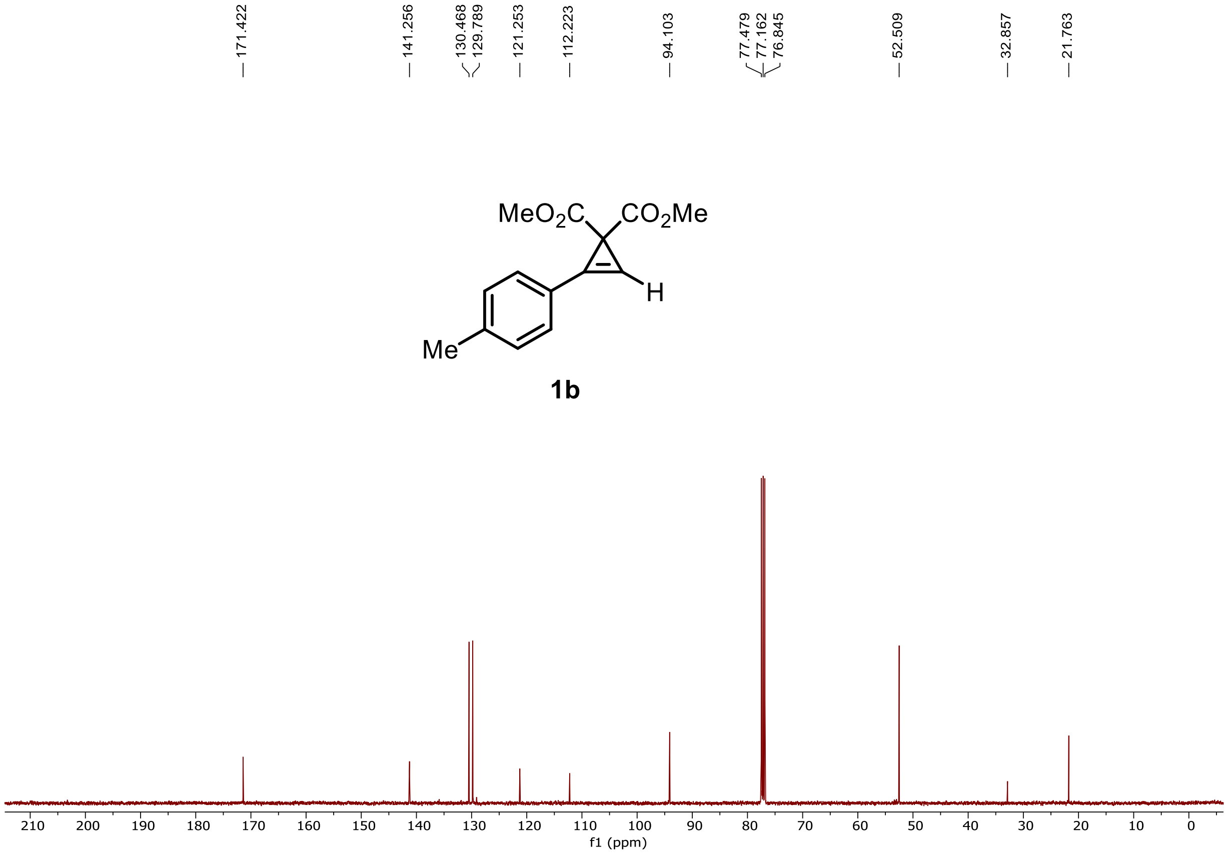

^{1}H NMR (400 MHz, $CDCl_3$) of 1c

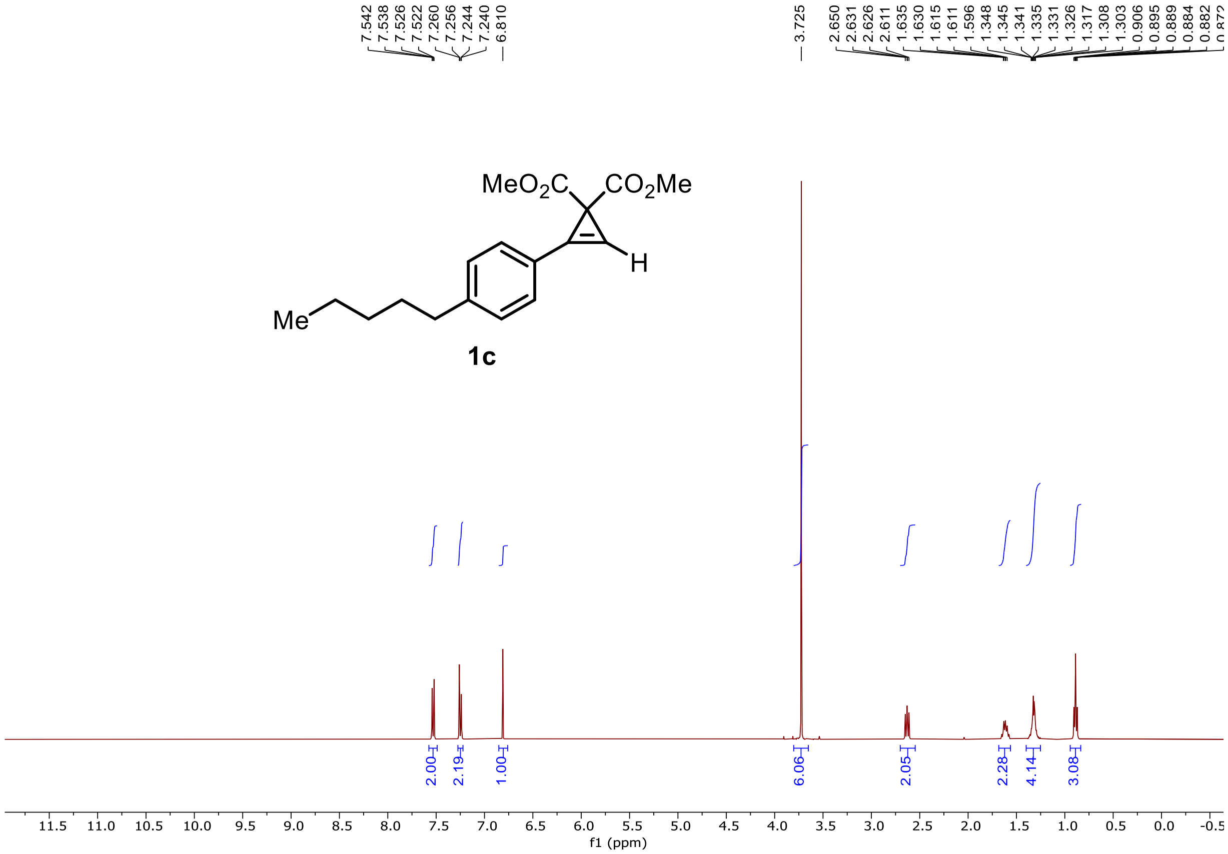

^{13}C NMR (101 MHz, $CDCl_3$) of 1c

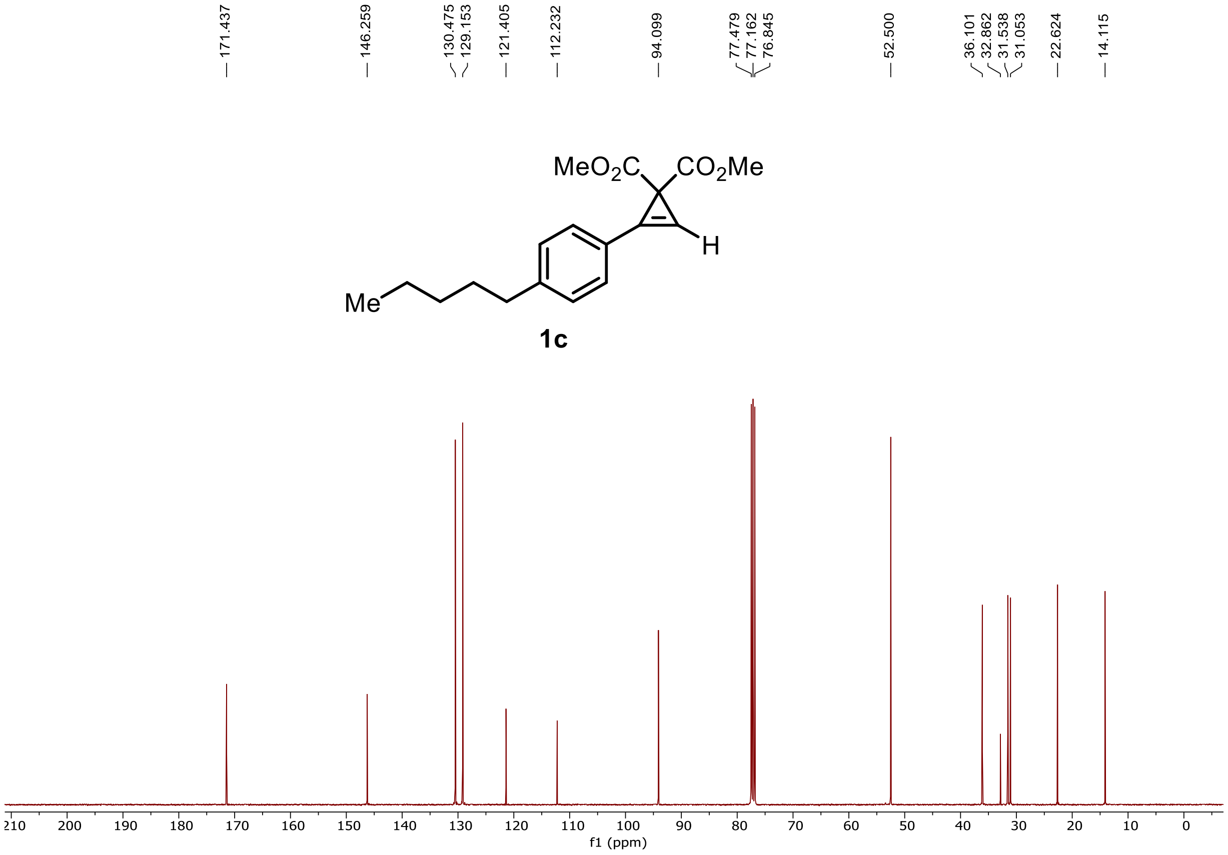

^{1}H NMR (400 MHz, $CDCl_3$) of 1d

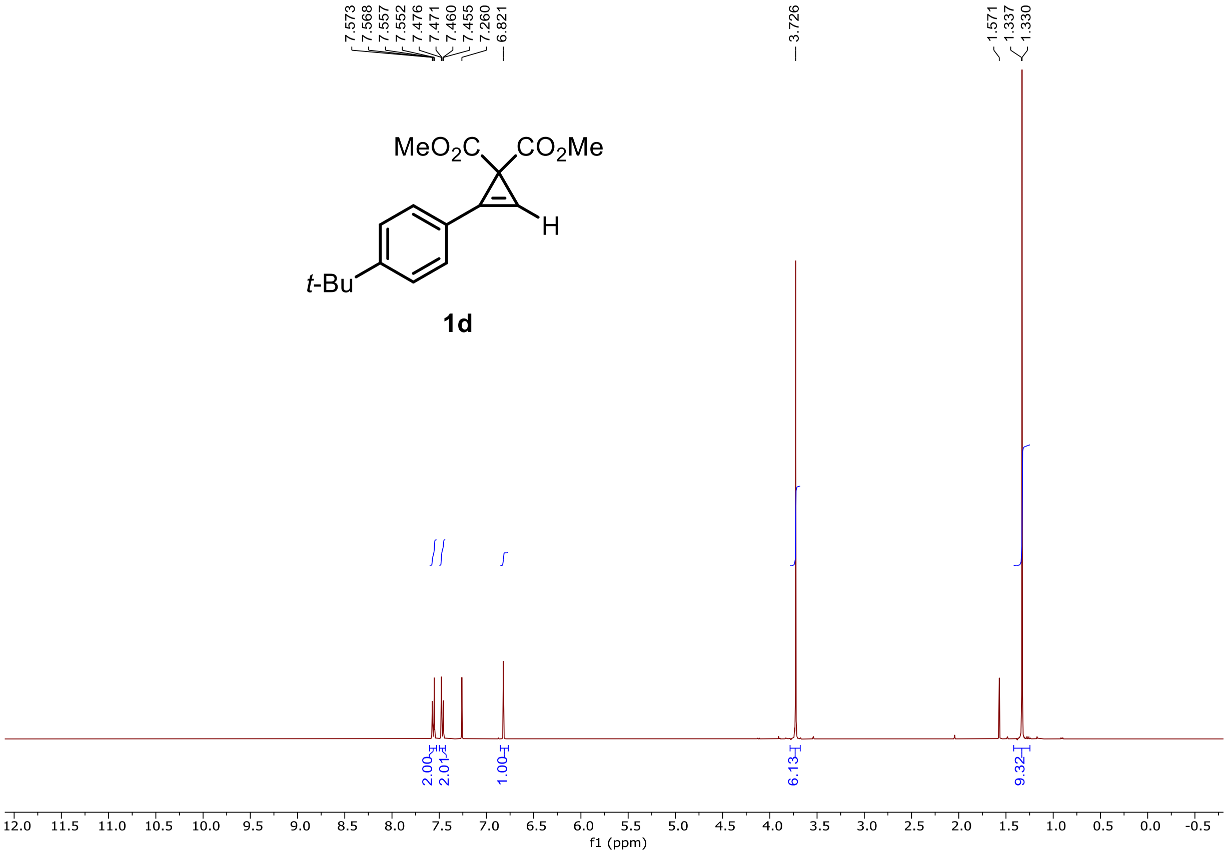

^{13}C NMR (101 MHz, $CDCl_3$) of 1d

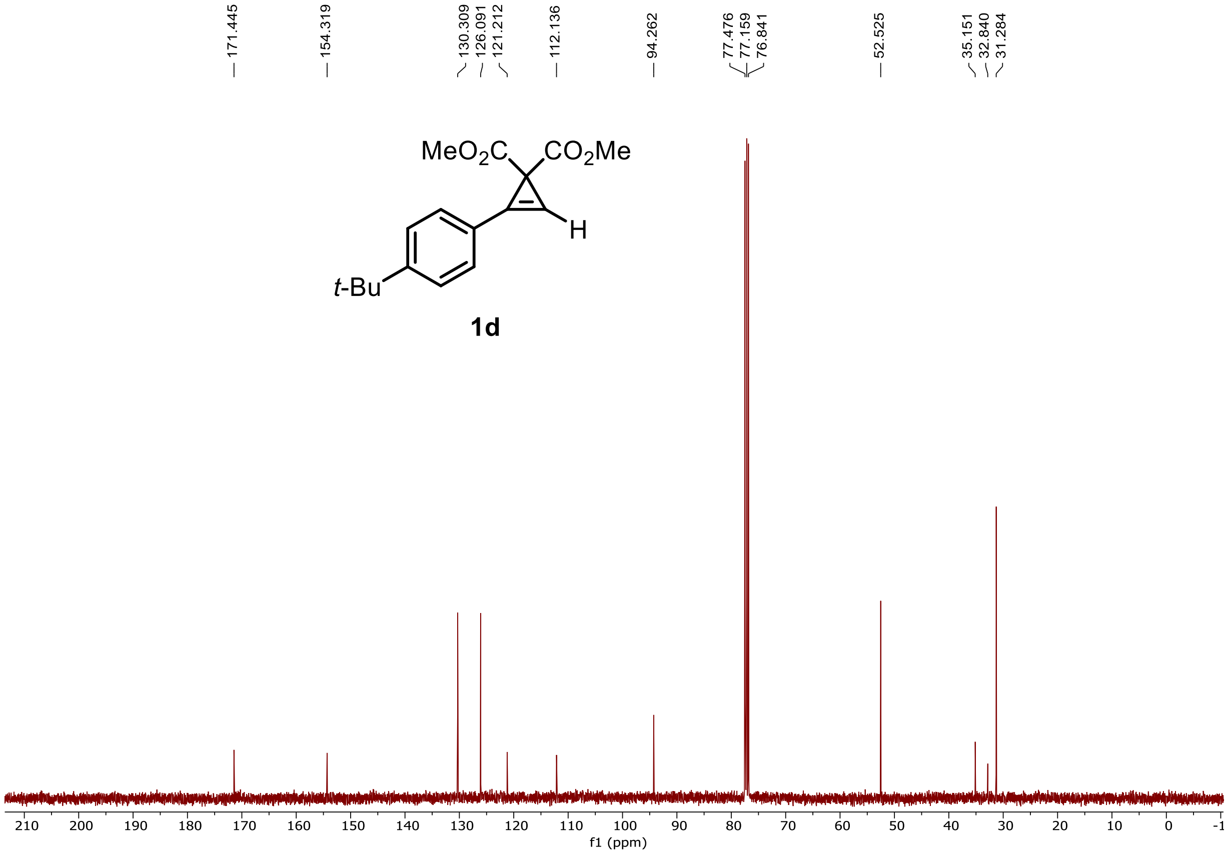

^{1}H NMR (400 MHz, $CDCl_3$) of 1e

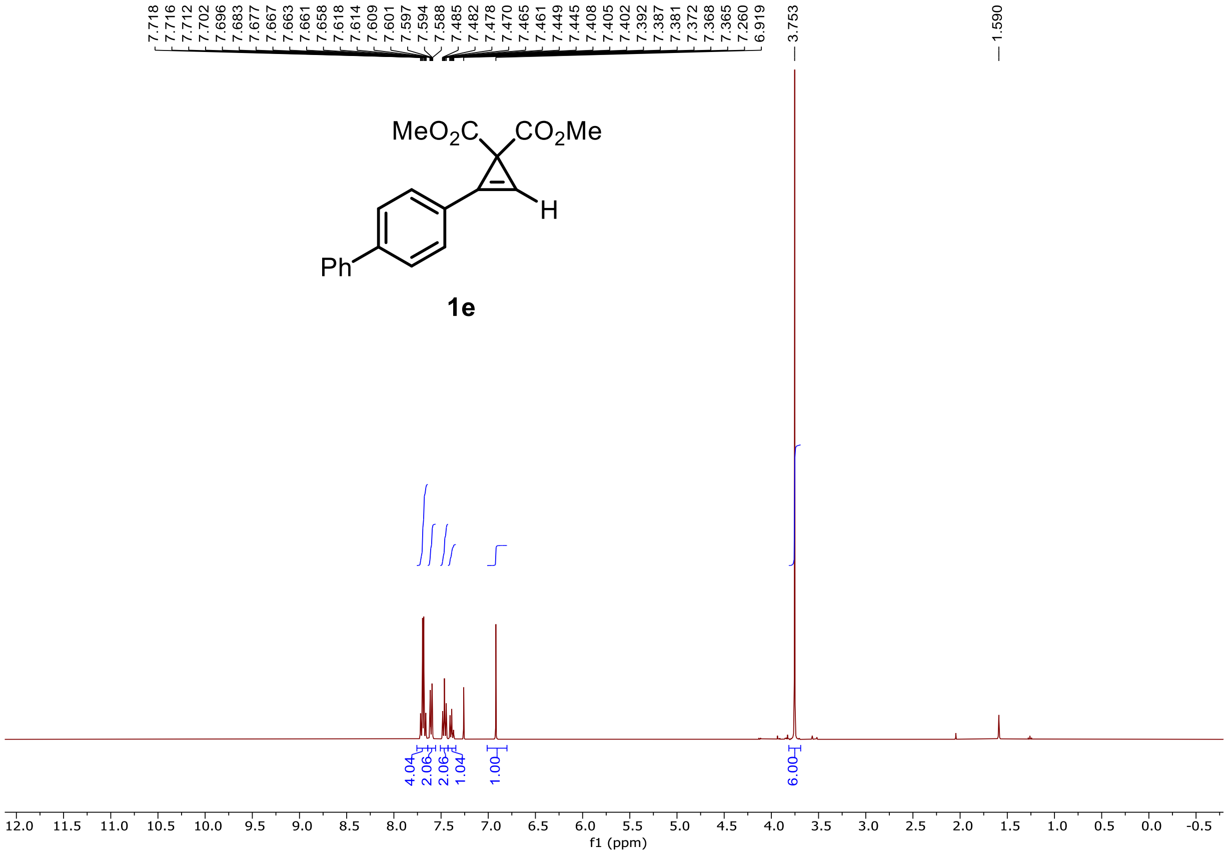

^{13}C NMR (101 MHz, $CDCl_3$) of 1e

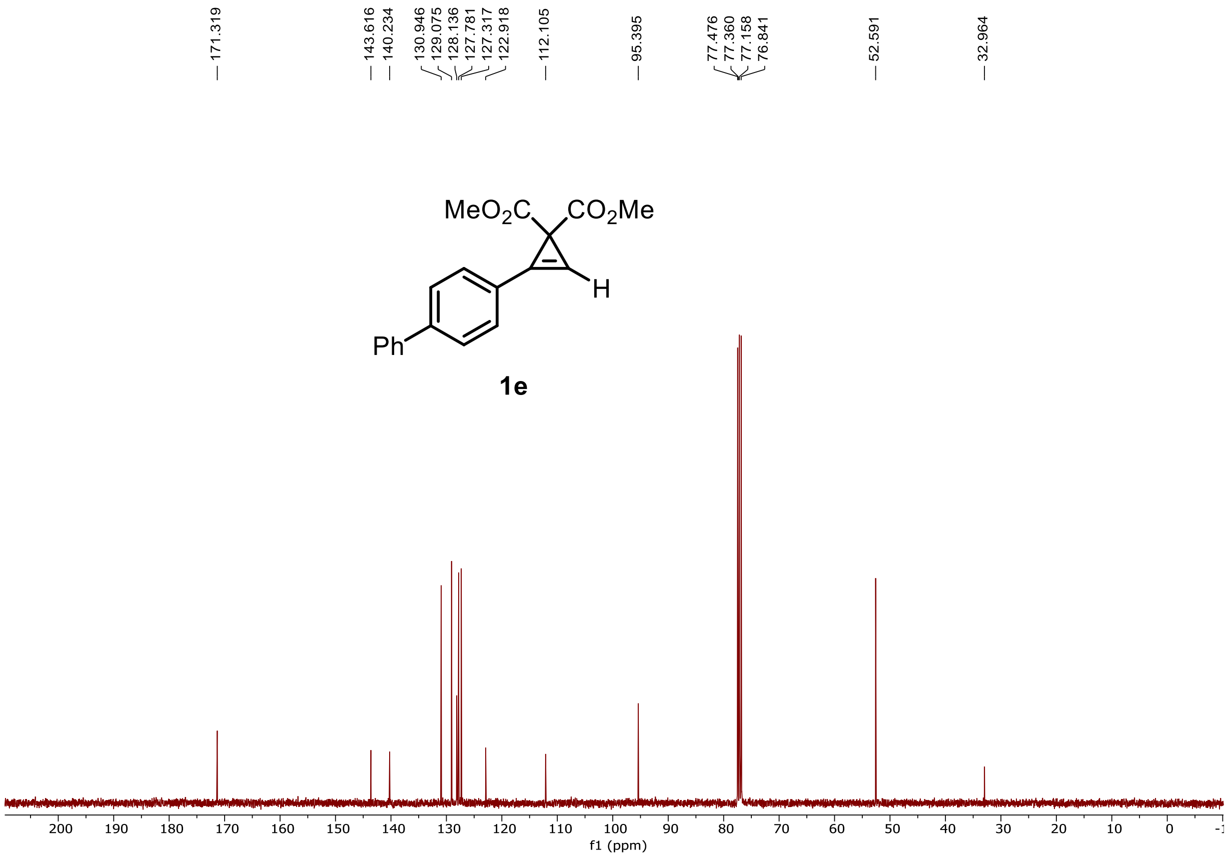

^{1}H NMR (400 MHz, $CDCl_3$) of 1f

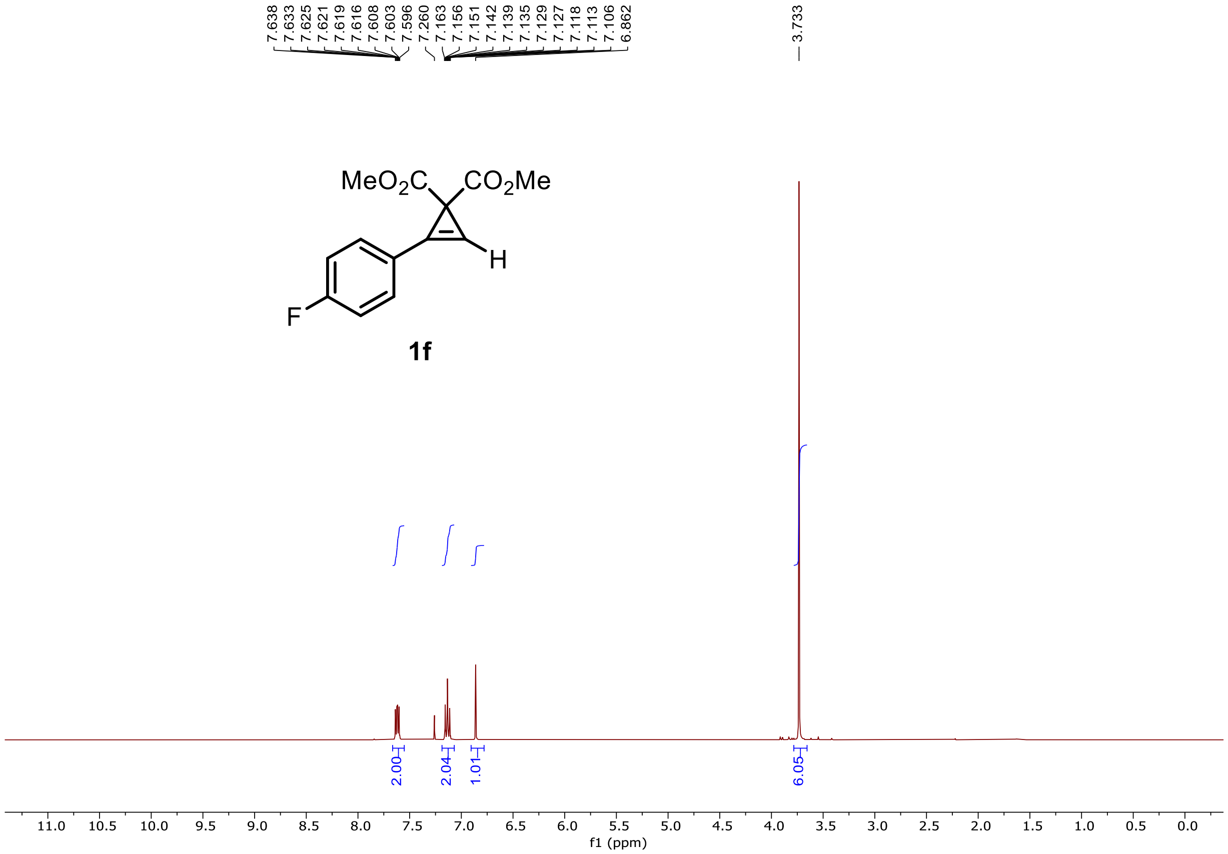

^{13}C NMR (101 MHz, $CDCl_3$) of 1f

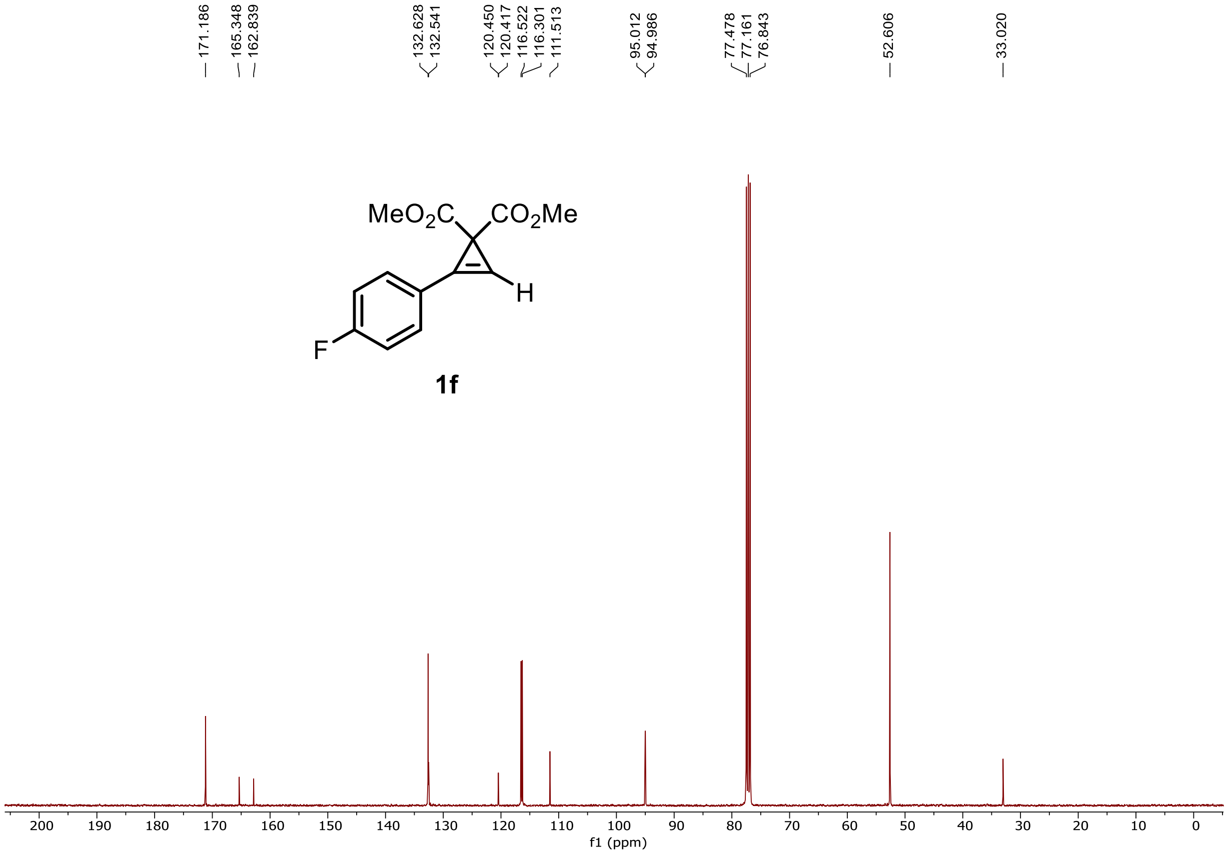

^{19}F NMR (377 MHz, $CDCl_3$) of 1f

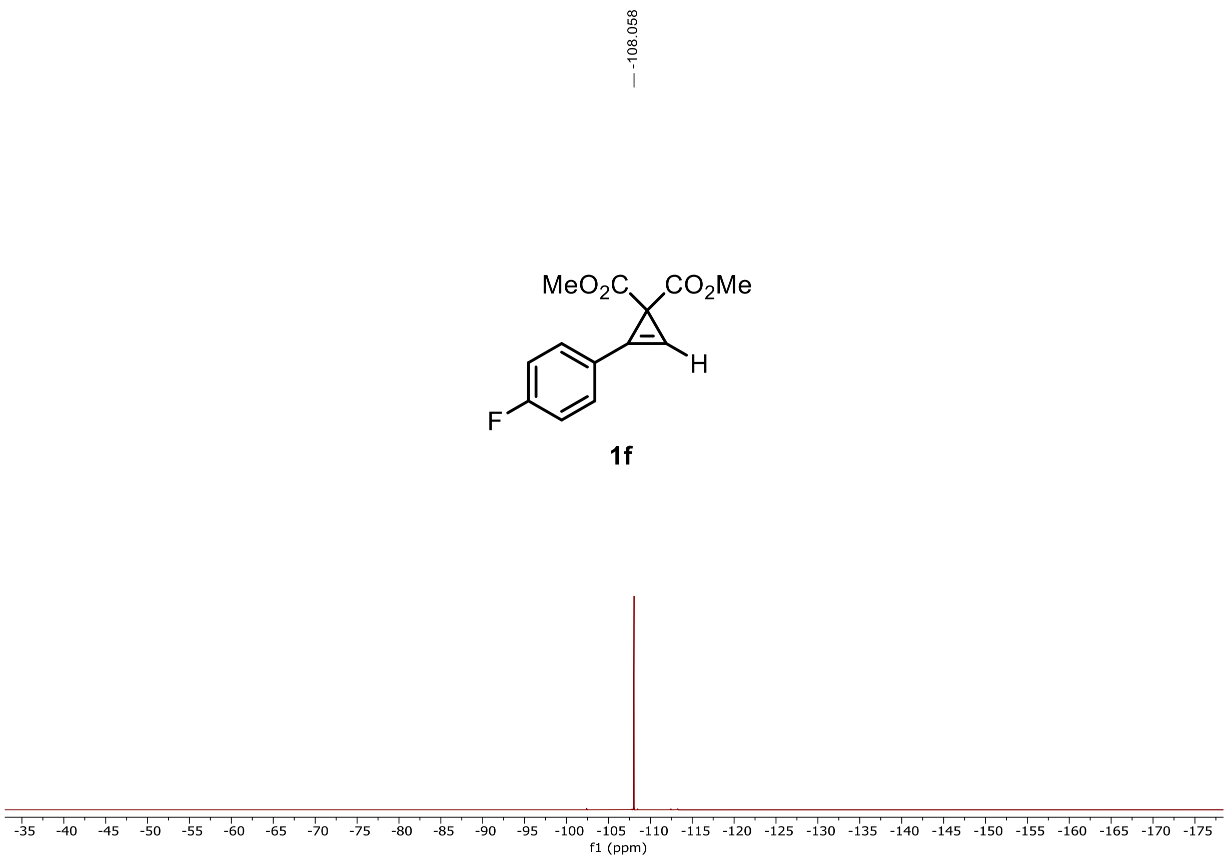

^{1}H NMR (400 MHz, $CDCl_3$) of 1g

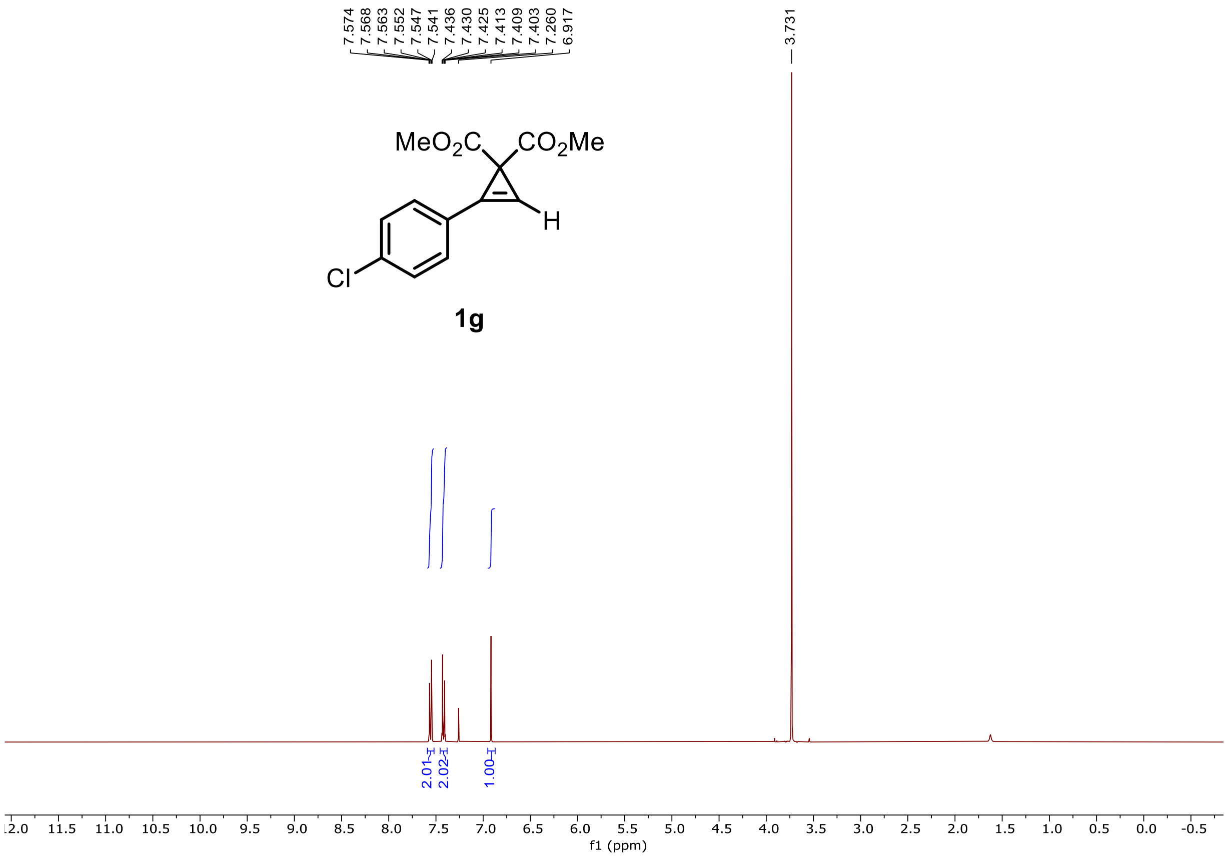

^{13}C NMR (101 MHz, $CDCl_3$) of 1g

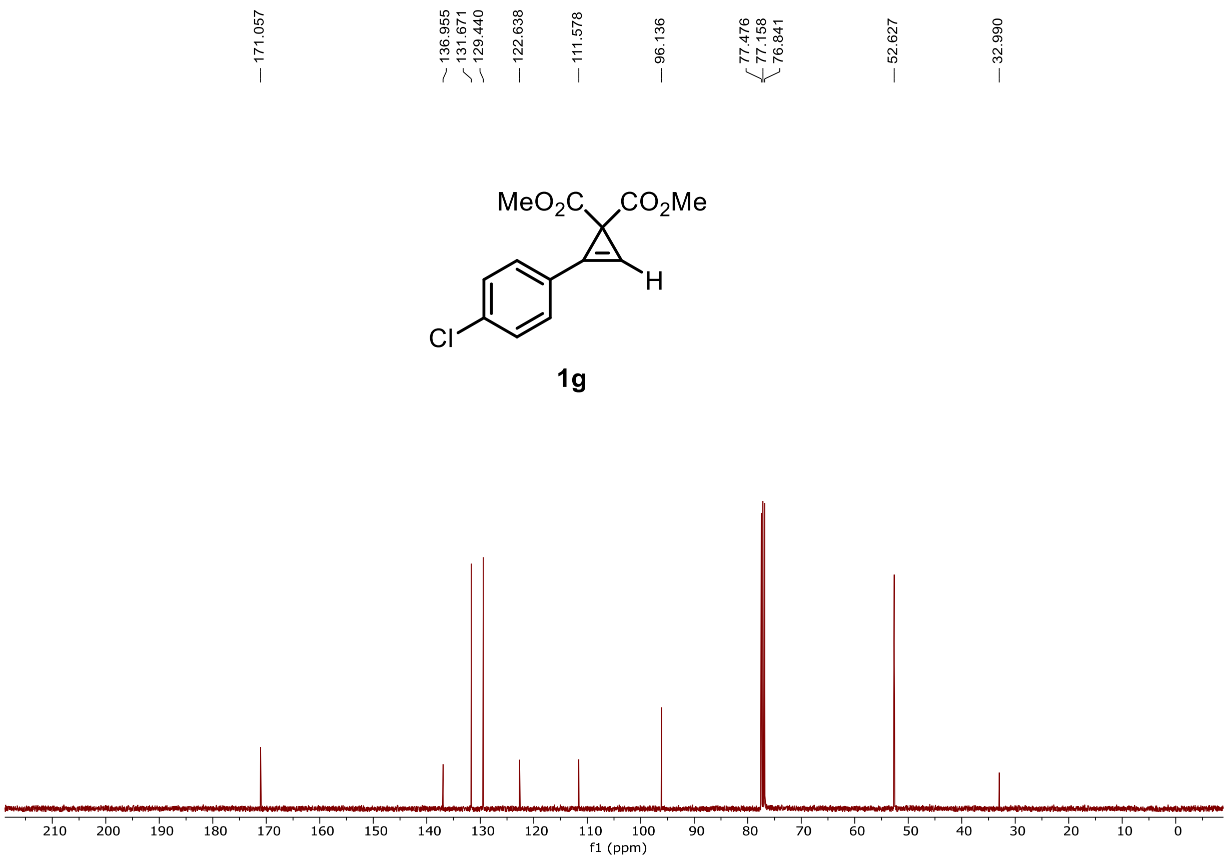

^{1}H NMR (400 MHz, $CDCl_3$) of 1h

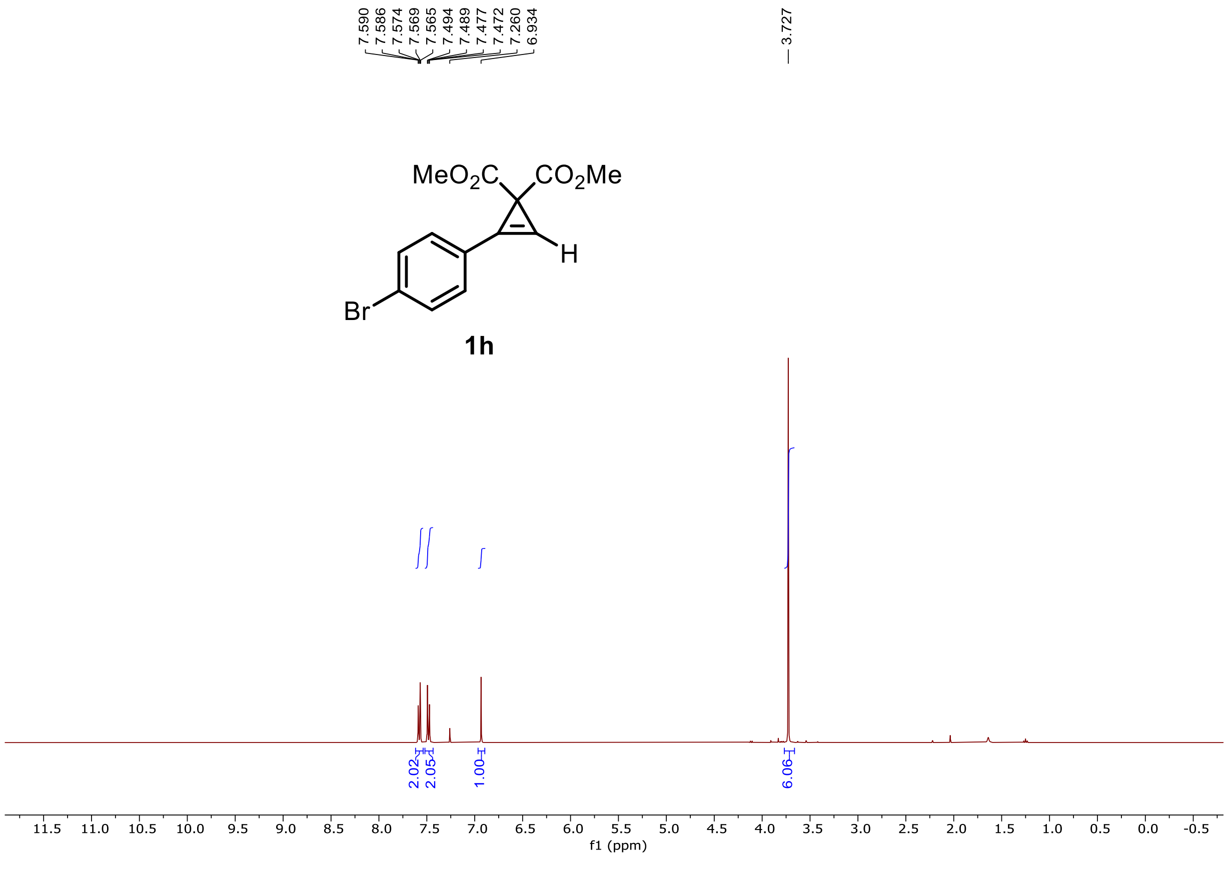

^{13}C NMR (101 MHz, $CDCl_3$) of 1h

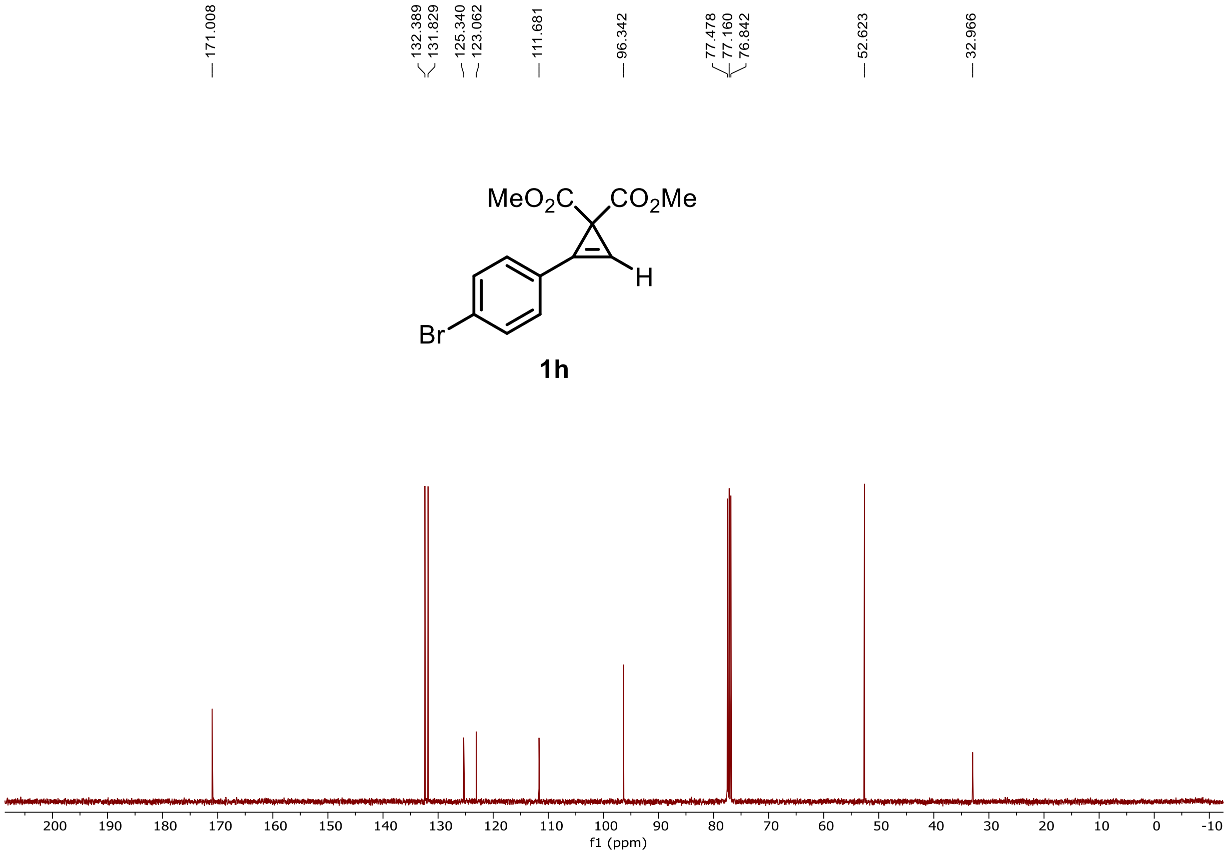

^{1}H NMR (400 MHz, $CDCl_3$) of 1i

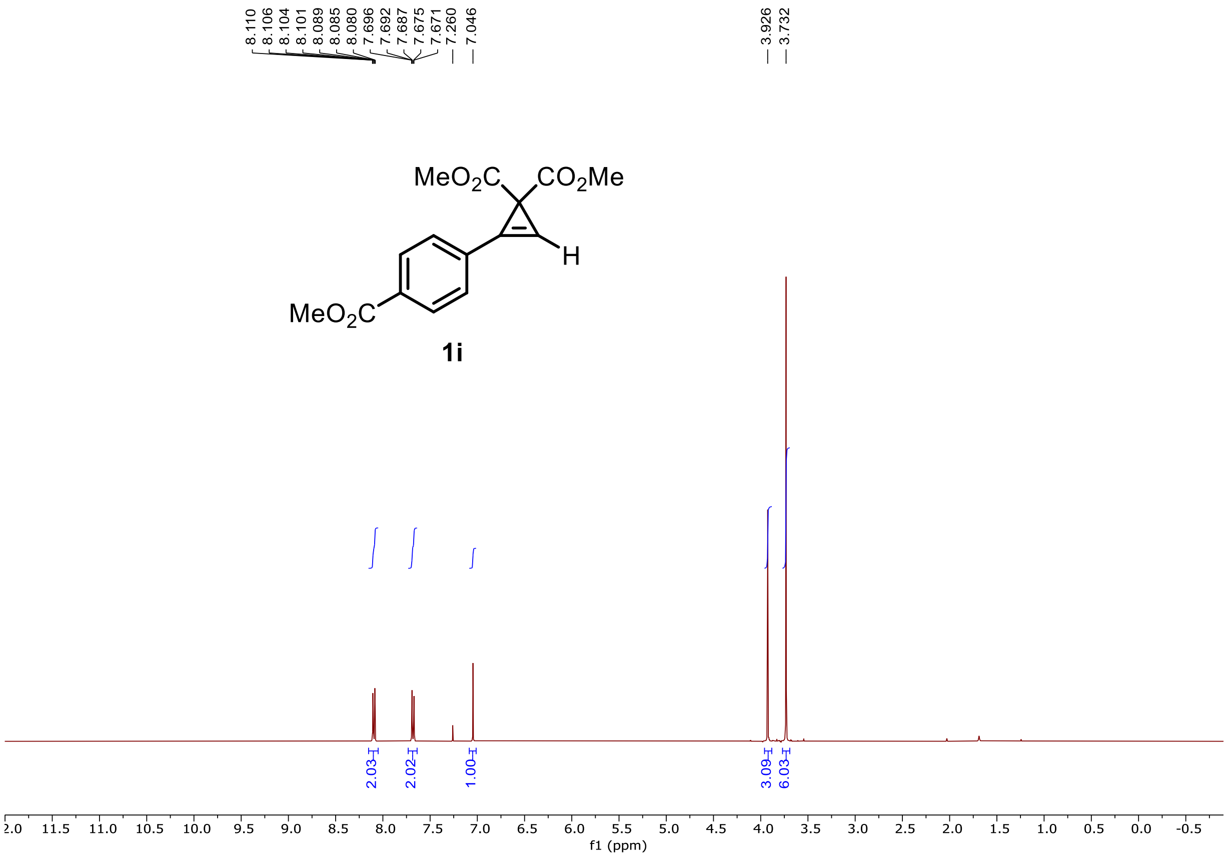

^{13}C NMR (101 MHz, $CDCl_3$) of 1i

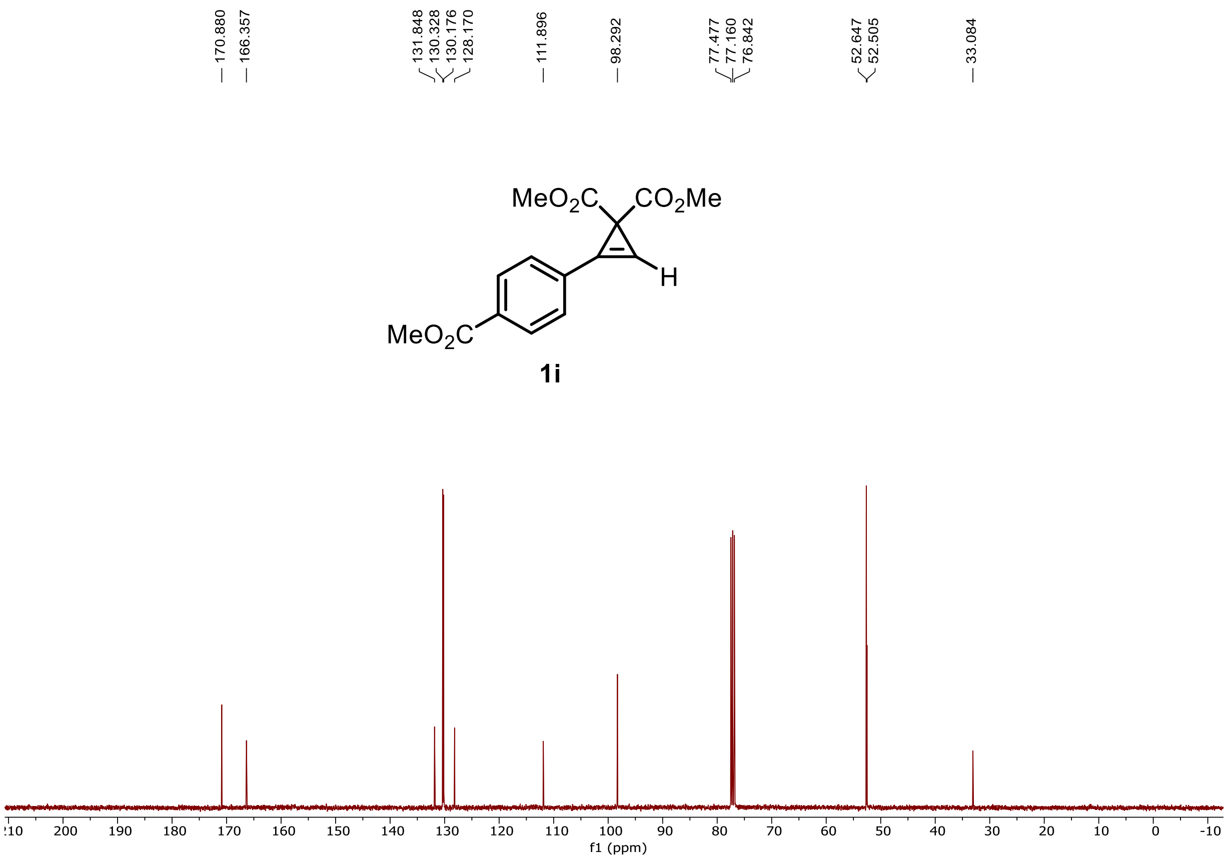

^{1}H NMR (400 MHz, $CDCl_3$) of 1j

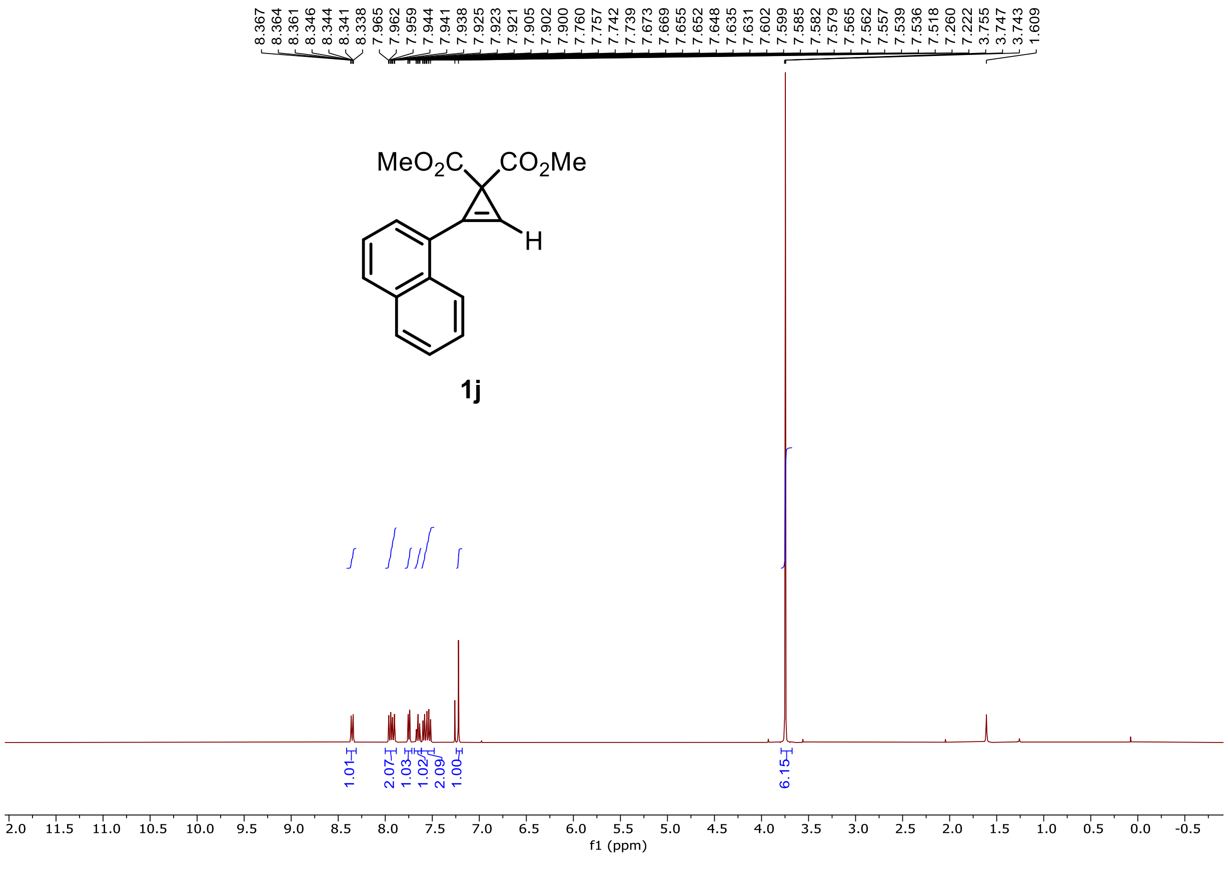

^{13}C NMR (101 MHz, $CDCl_3$) of 1j

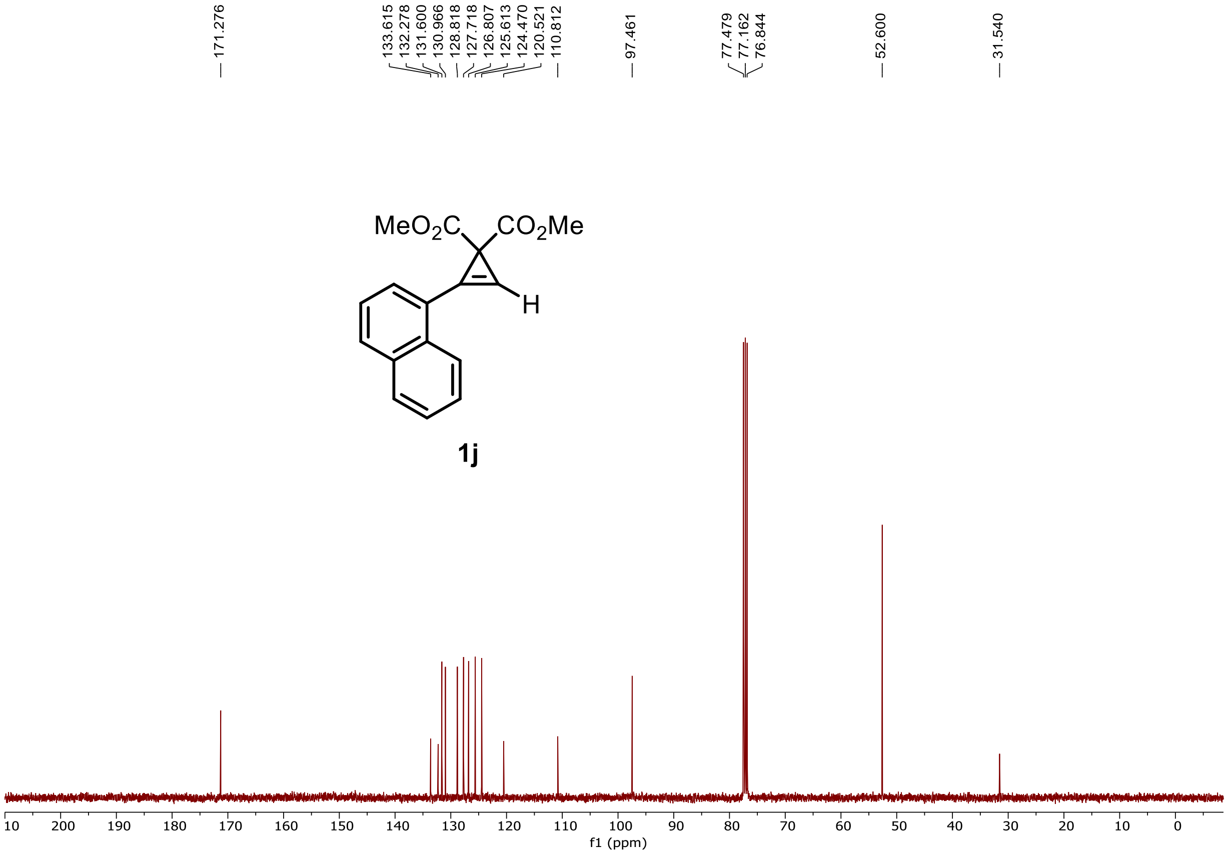

^{1}H NMR (400 MHz, $CDCl_3$) of 1k

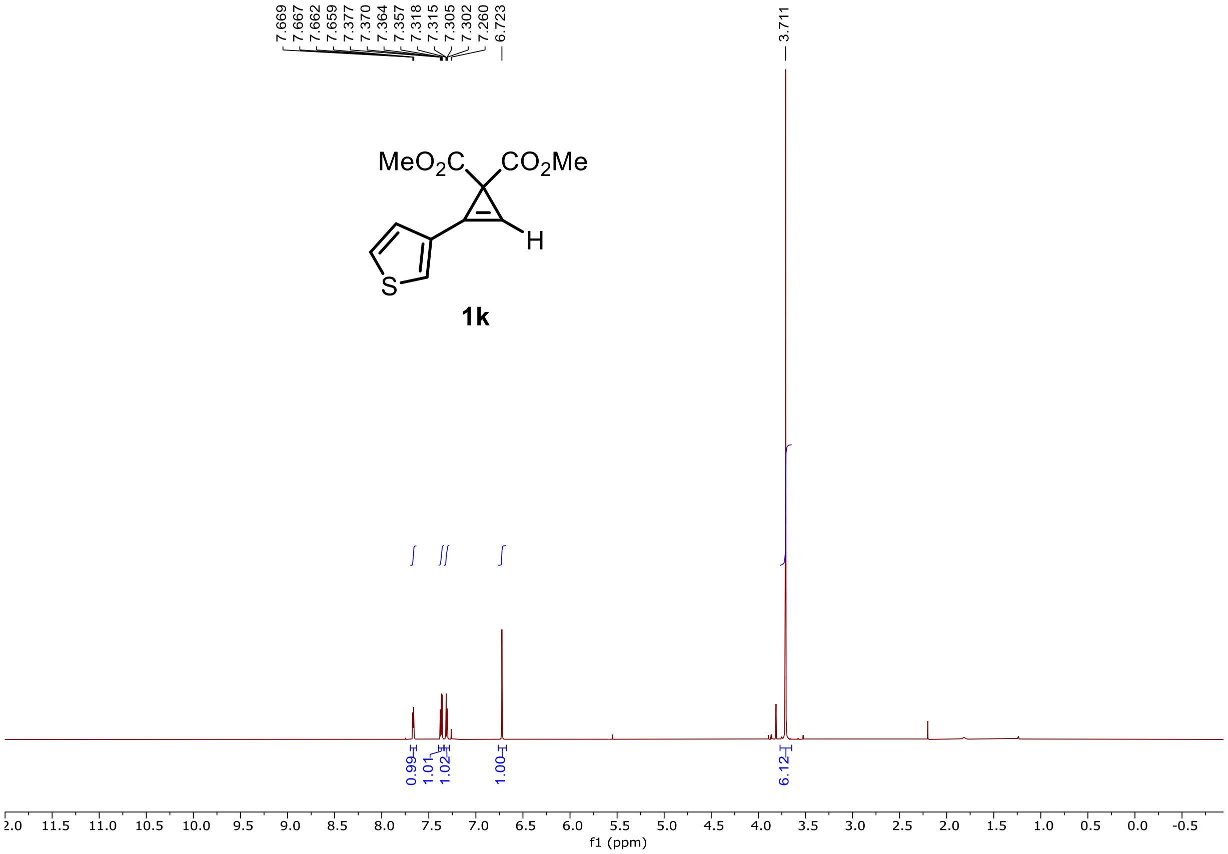

^{13}C NMR (101 MHz, $CDCl_3$) of 1k

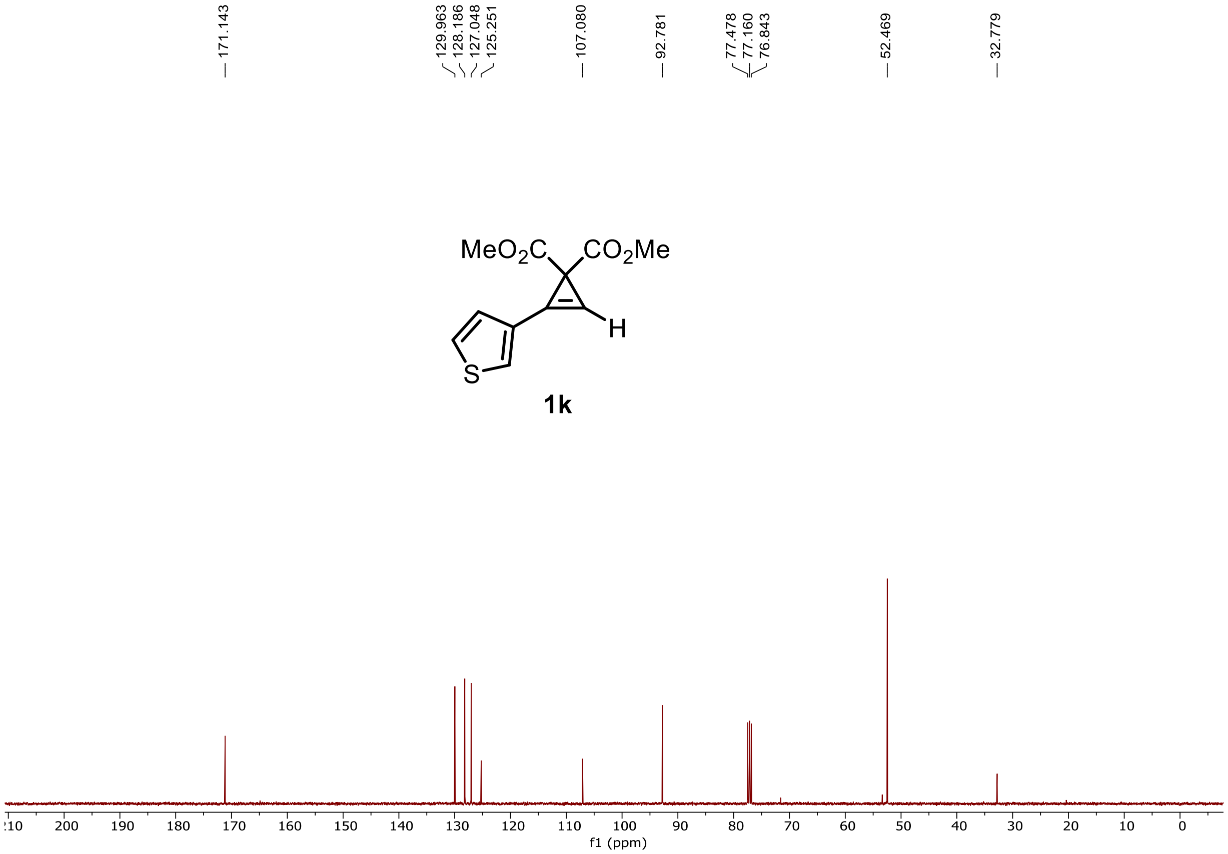

^{1}H NMR (400 MHz, $CDCl_3$) of 1l

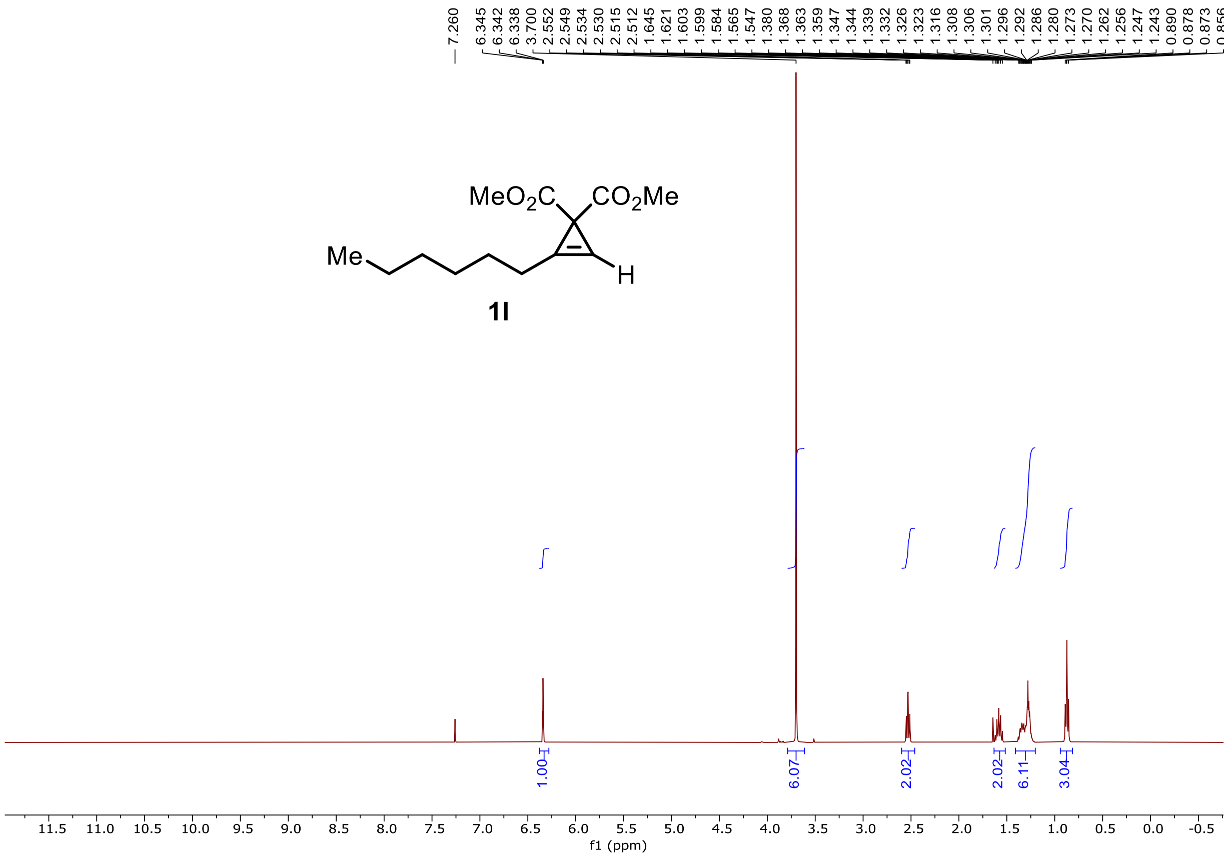

^{13}C NMR (101 MHz, $CDCl_3$) of 1l

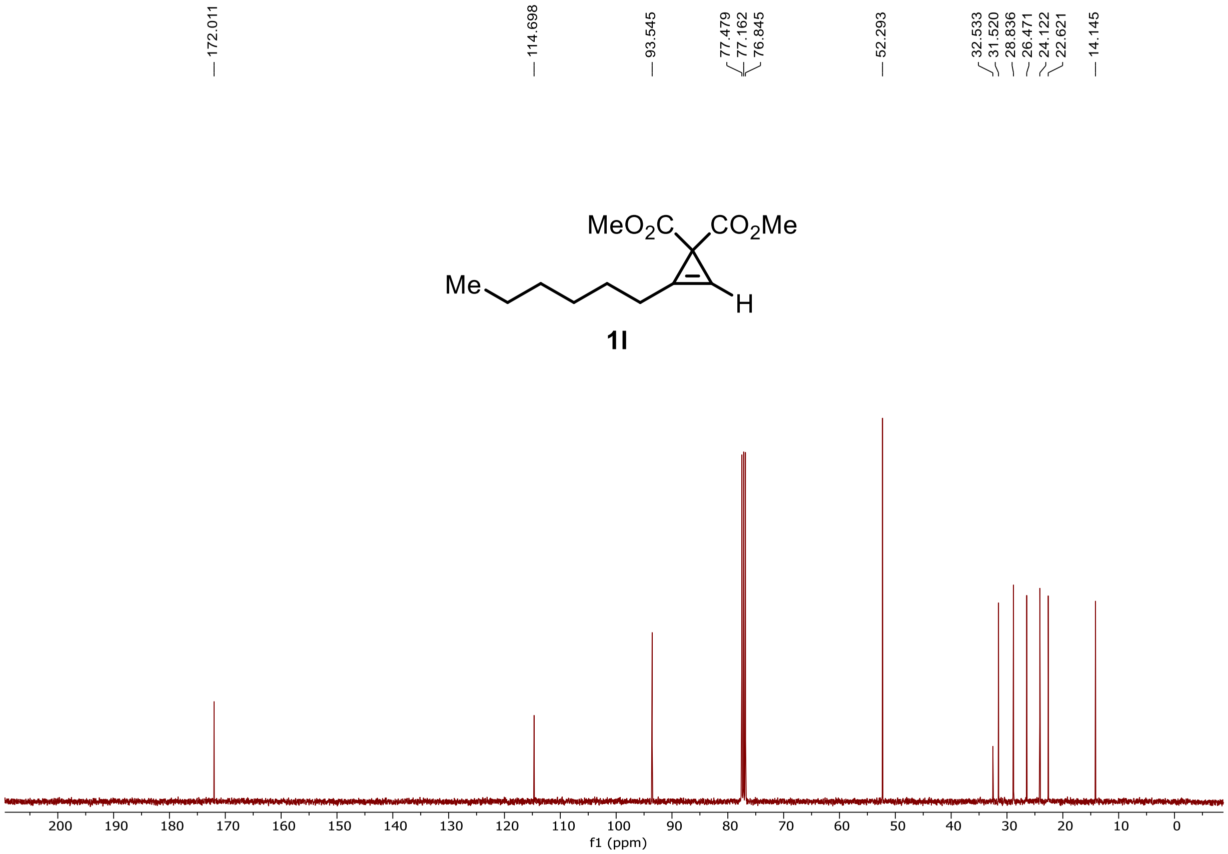

^{1}H NMR (400 MHz, $CDCl_3$) of 1m

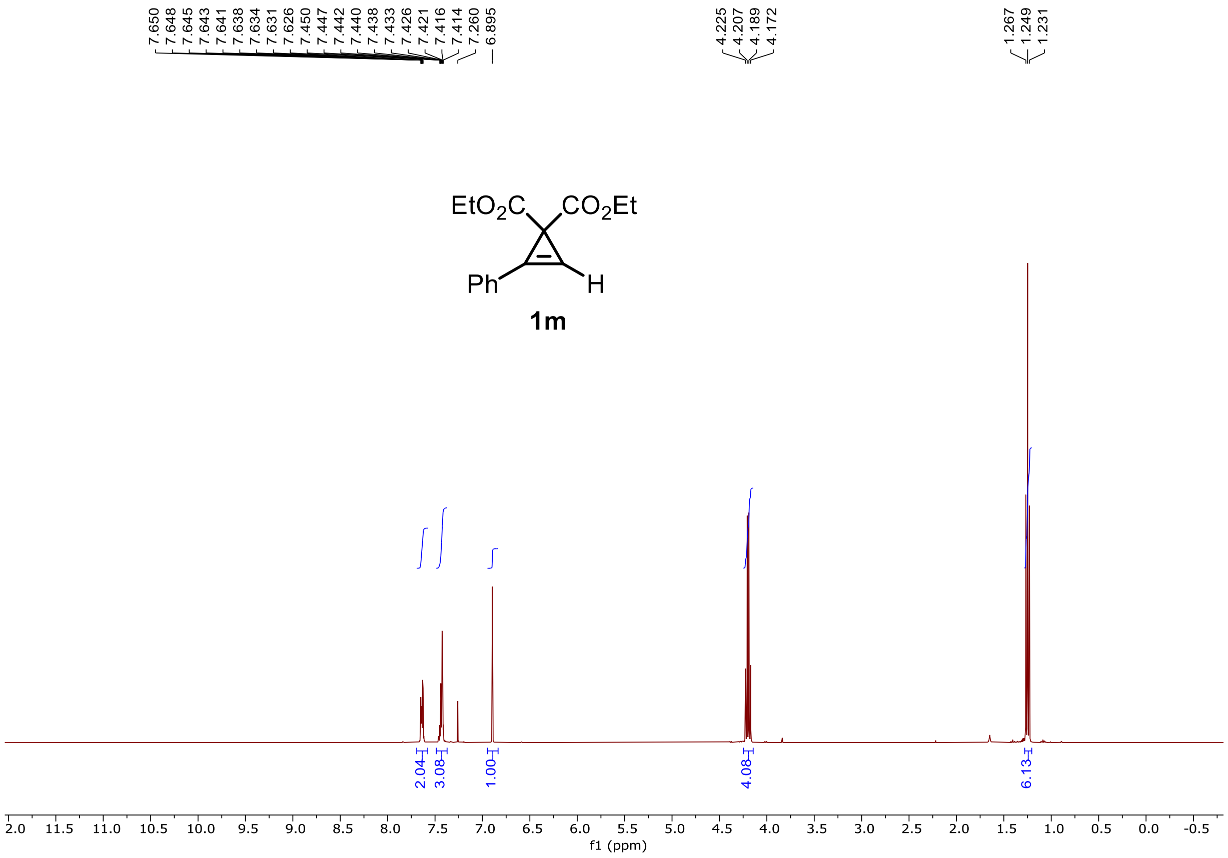

^{13}C NMR (101 MHz, $CDCl_3$) of 1m

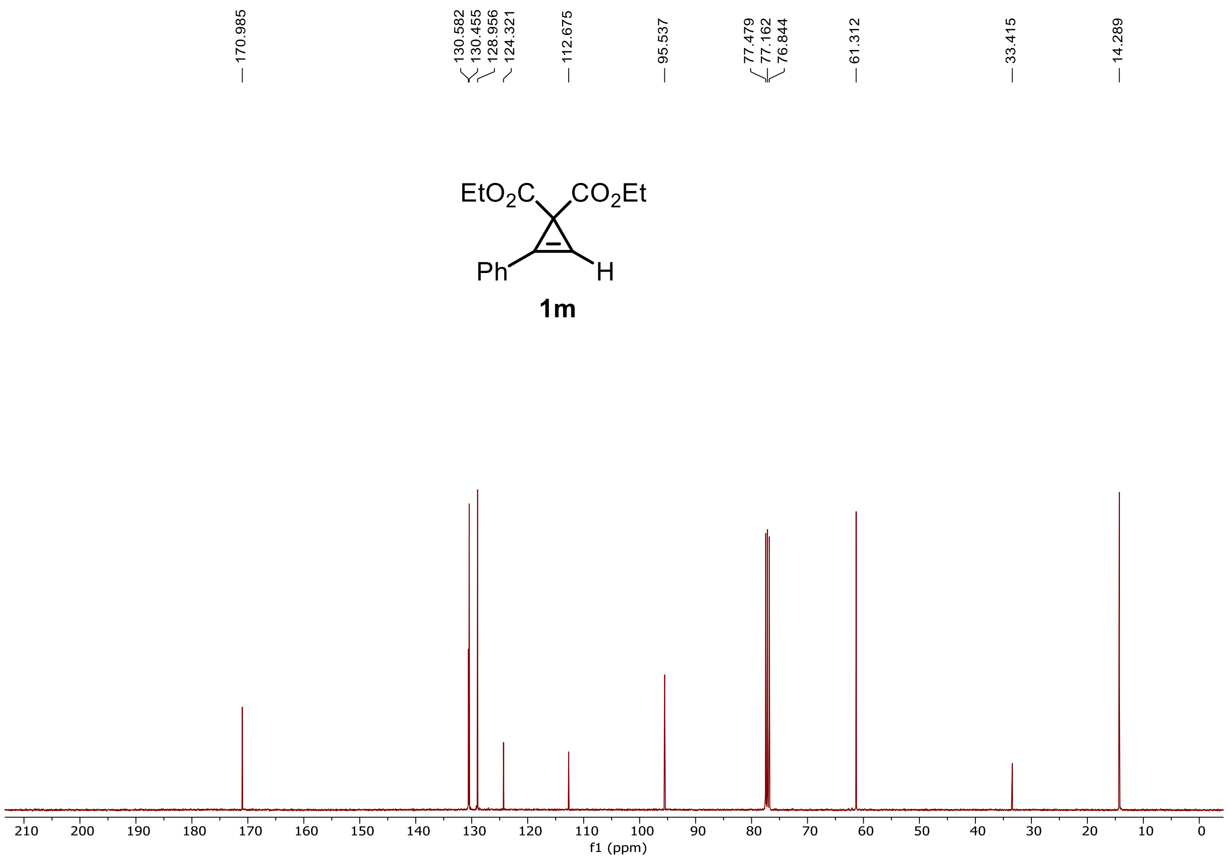

^{1}H NMR (400 MHz, $CDCl_3$) of 1n

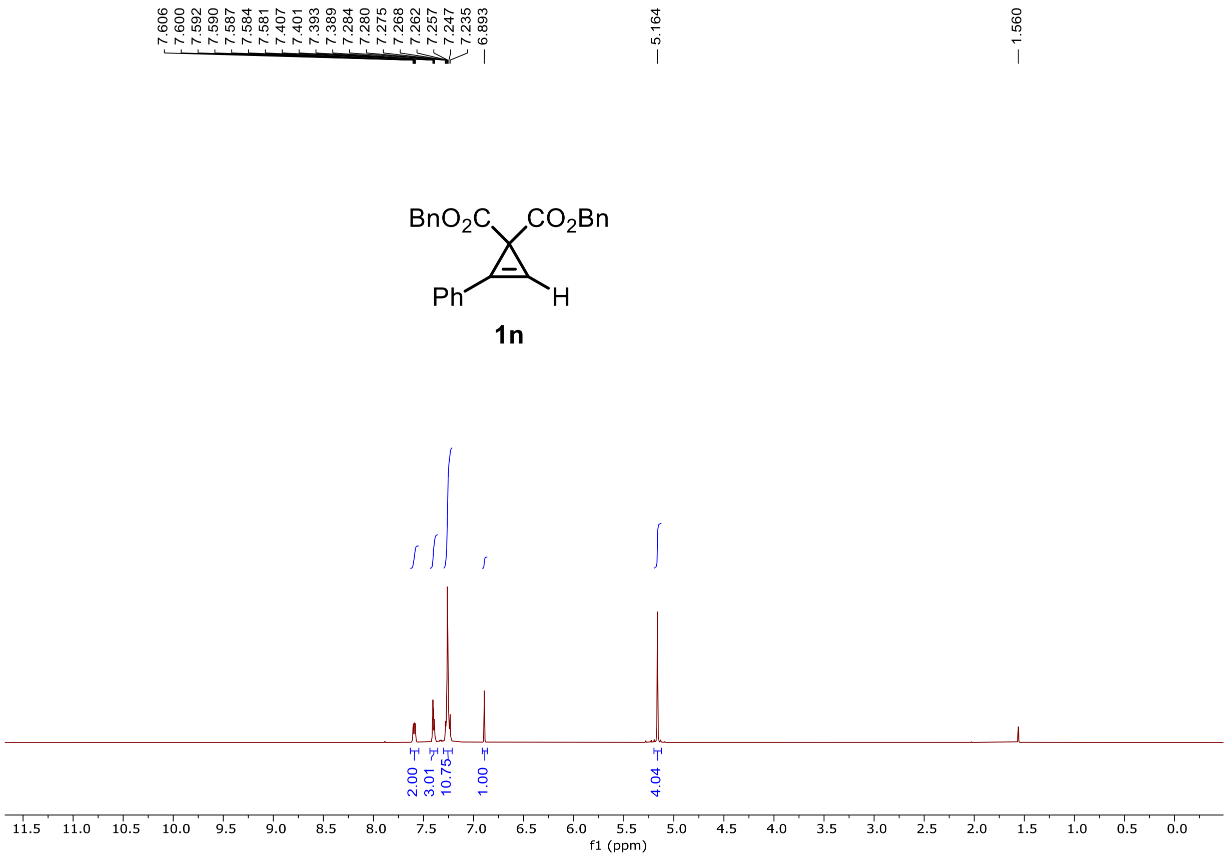

^{13}C NMR (101 MHz, $CDCl_3$) of 1n

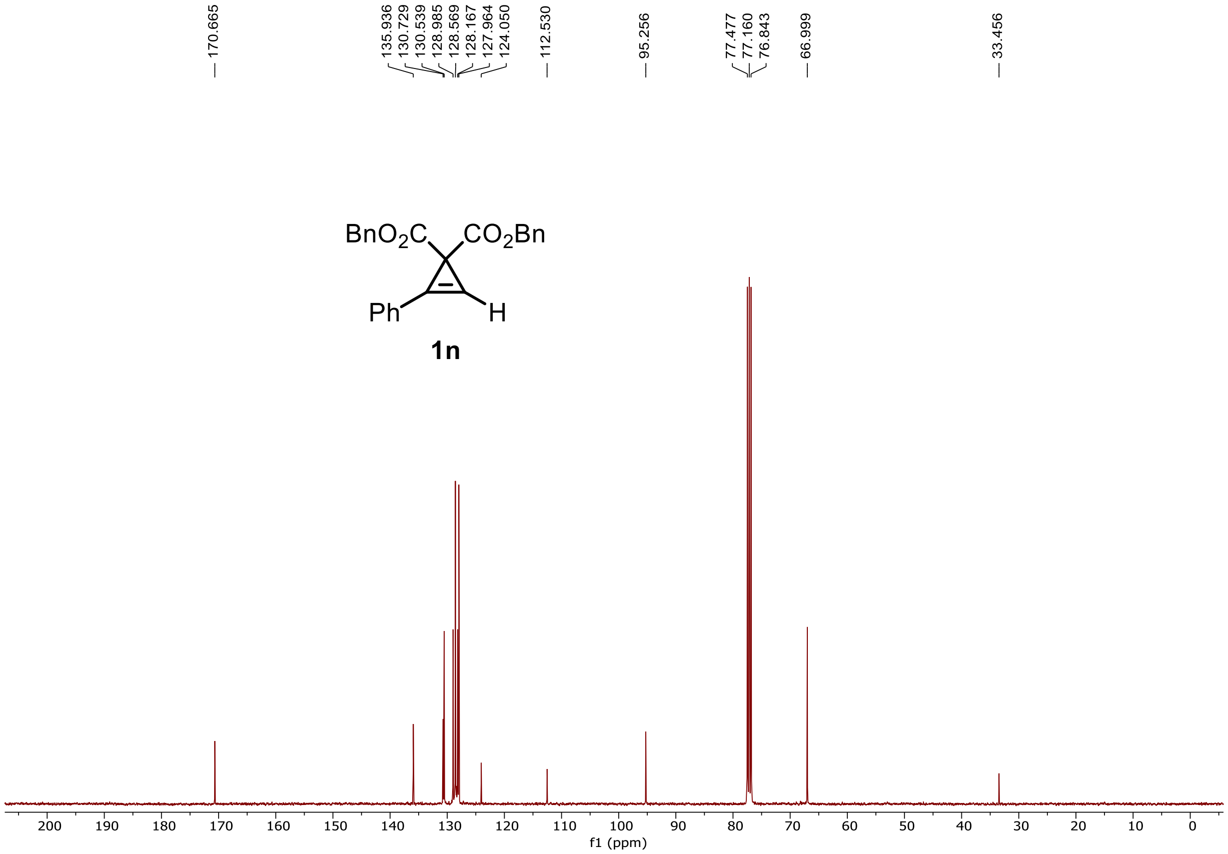

^{1}H NMR (400 MHz, $CDCl_3$) of 1o

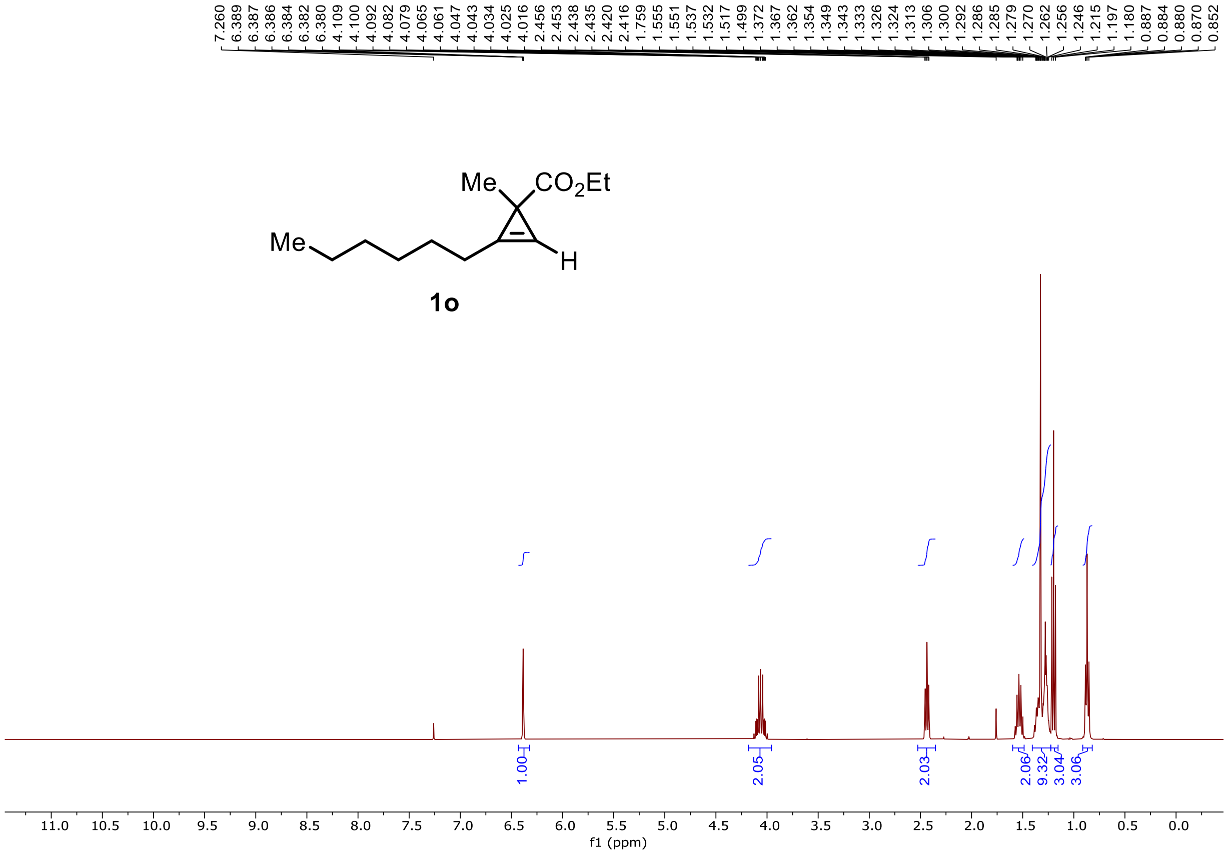

^{13}C NMR (101 MHz, $CDCl_3$) of 1o

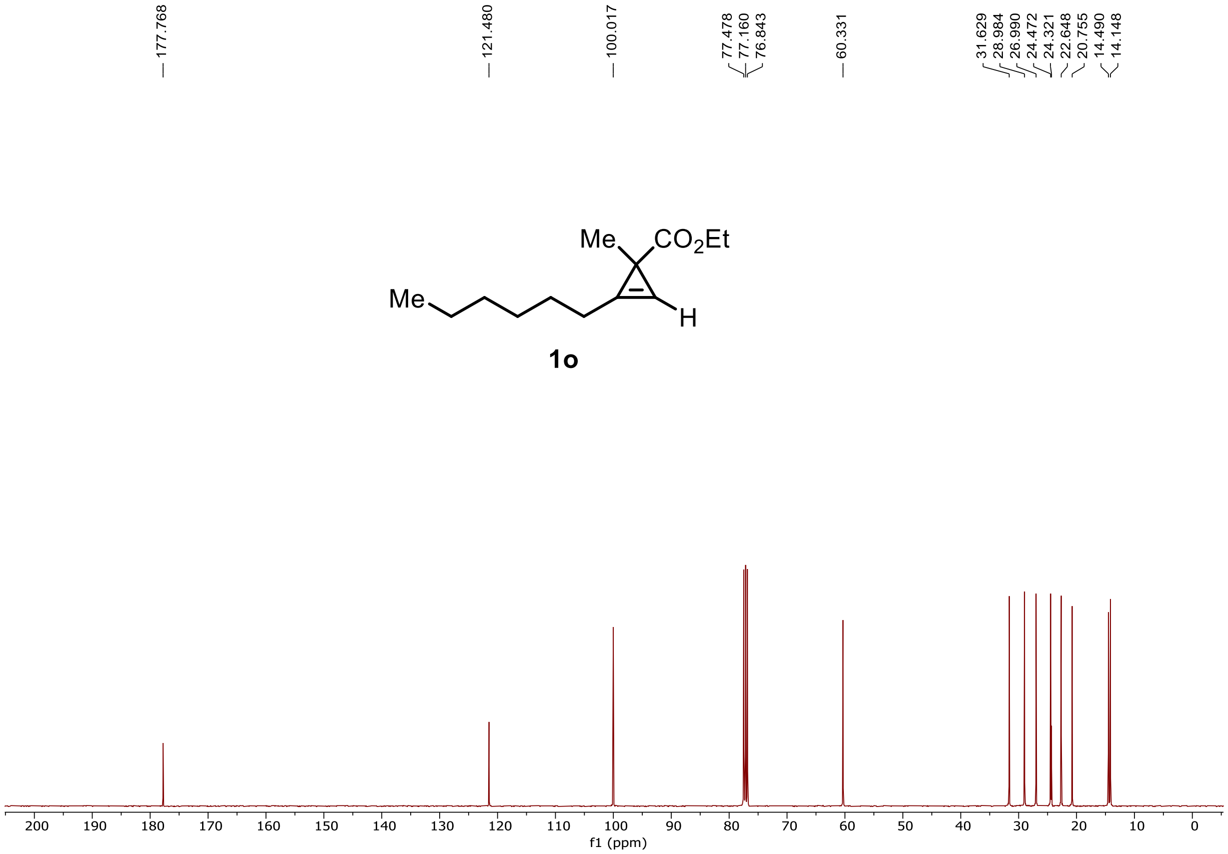

^{1}H NMR (400 MHz, $CDCl_3$) of 1p

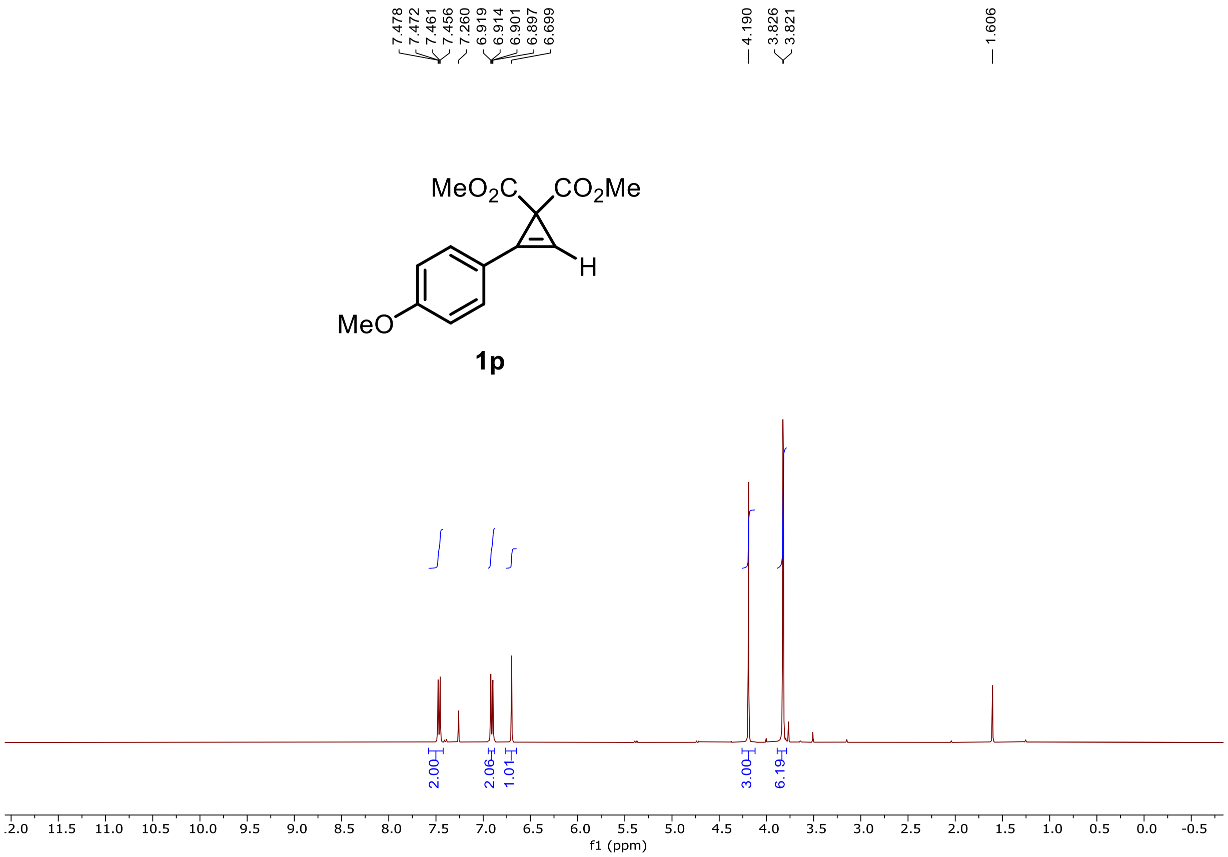

^{13}C NMR (101 MHz, $CDCl_3$) of 1p

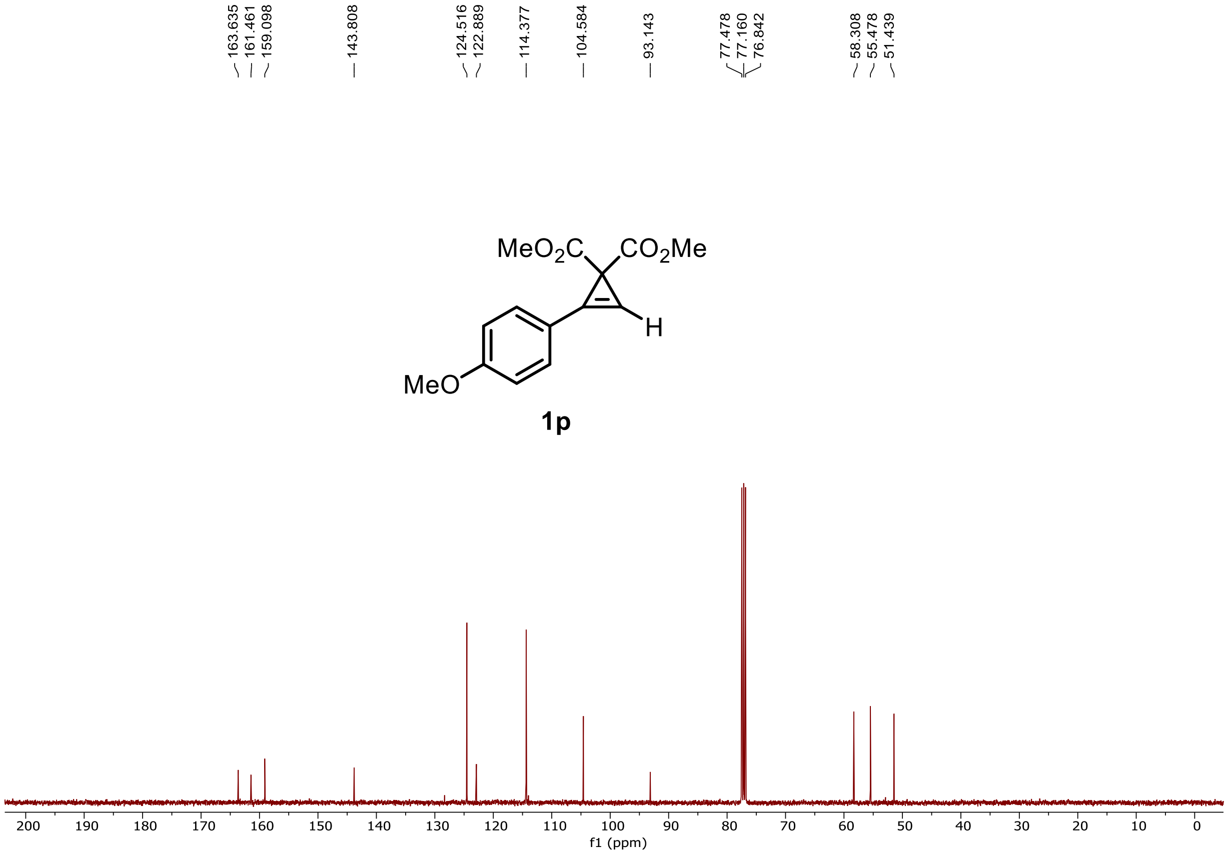

^{1}H NMR (400 MHz, $CDCl_3$) of 1q

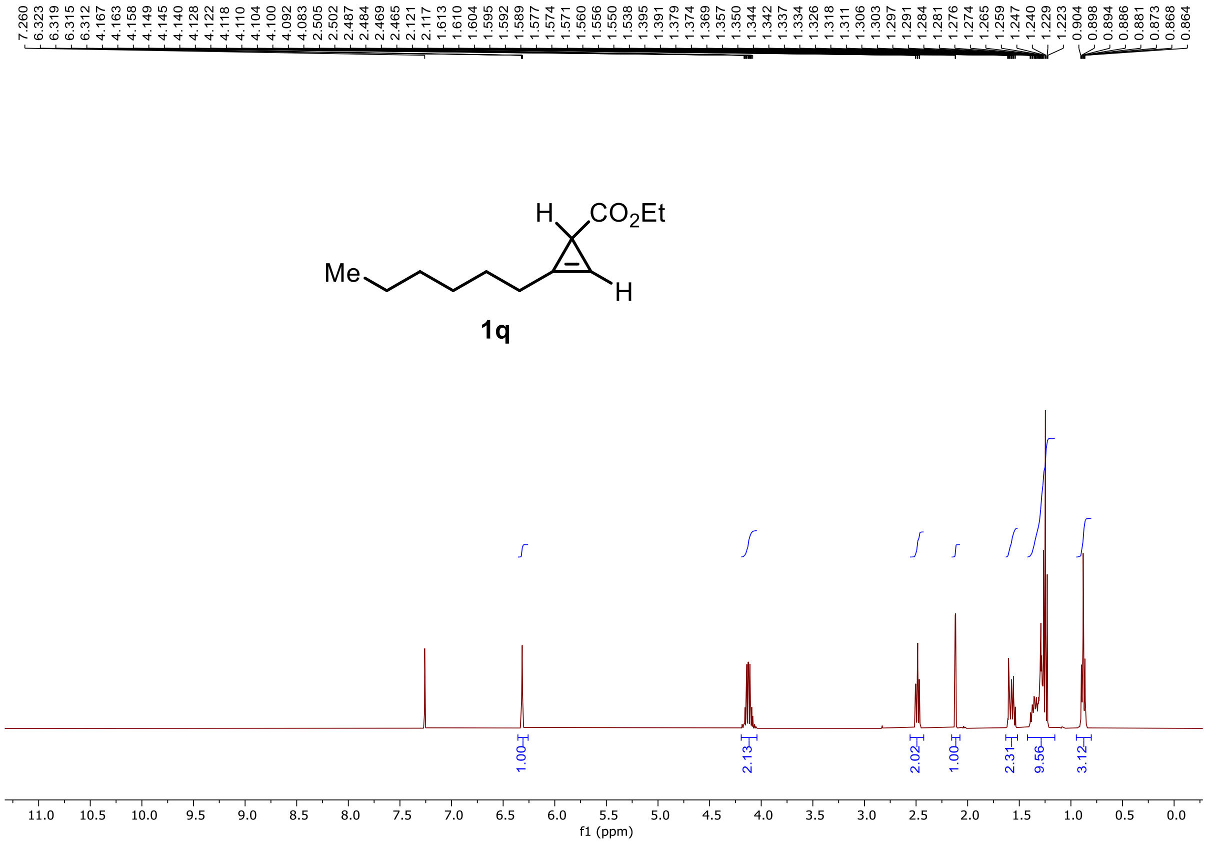

^{13}C NMR (101 MHz, CDCl$_3$) of 1q

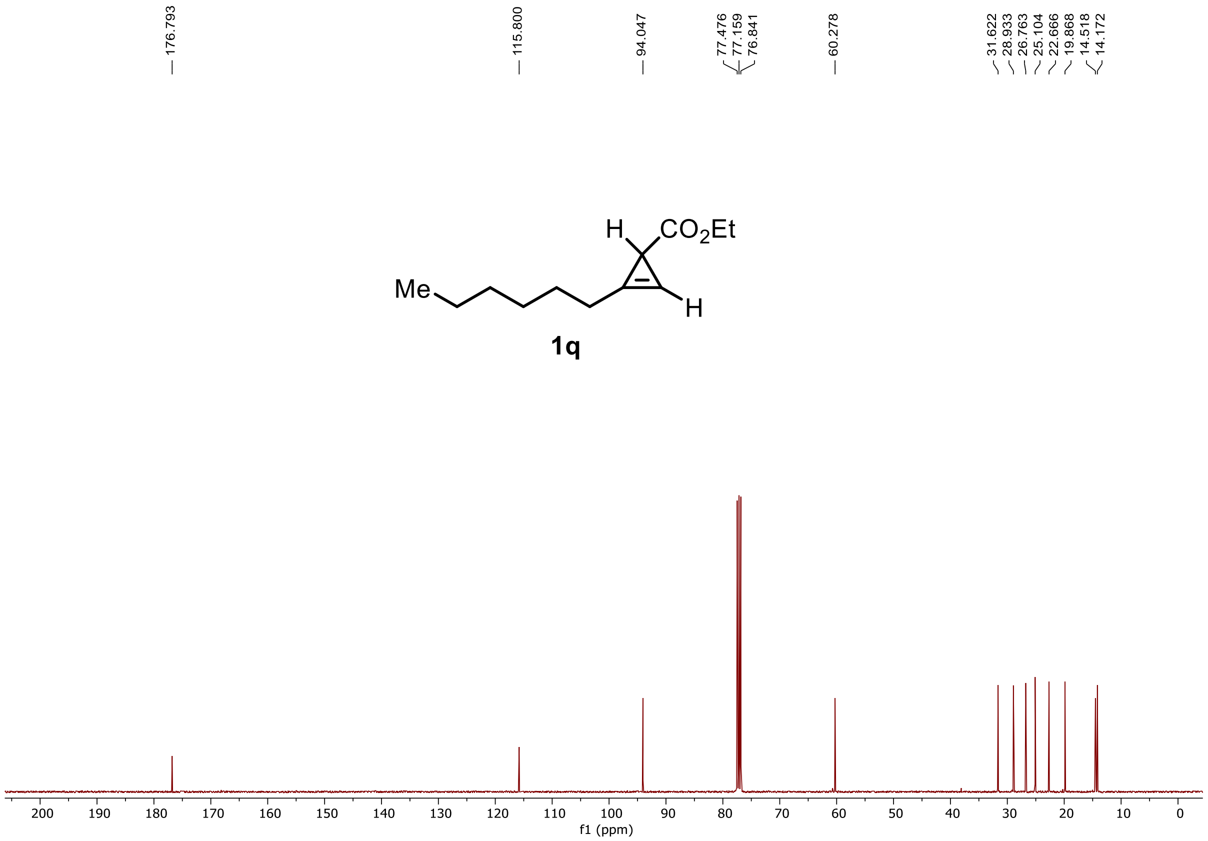

^{1}H NMR (400 MHz, CDCl$_3$) of 1r

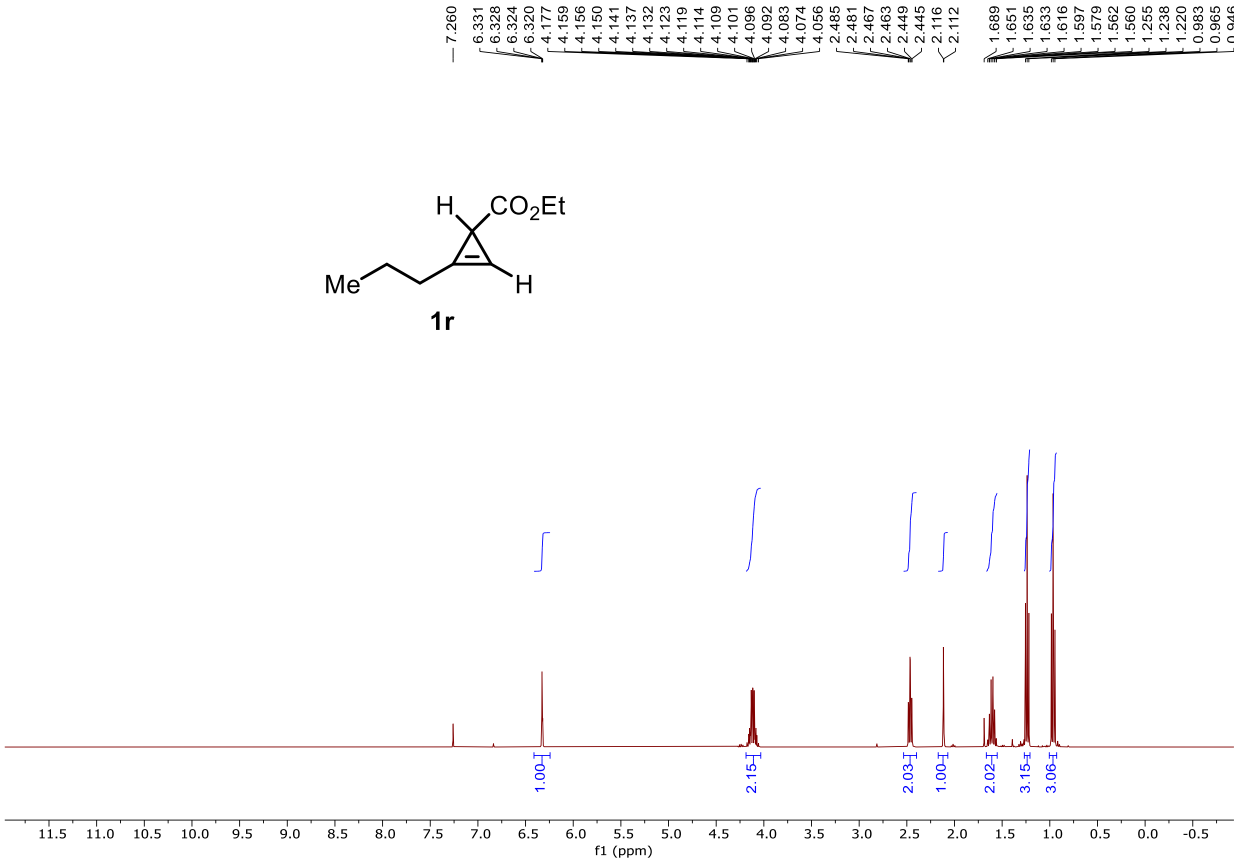

^{13}C NMR (101 MHz, $CDCl_3$) of 1r

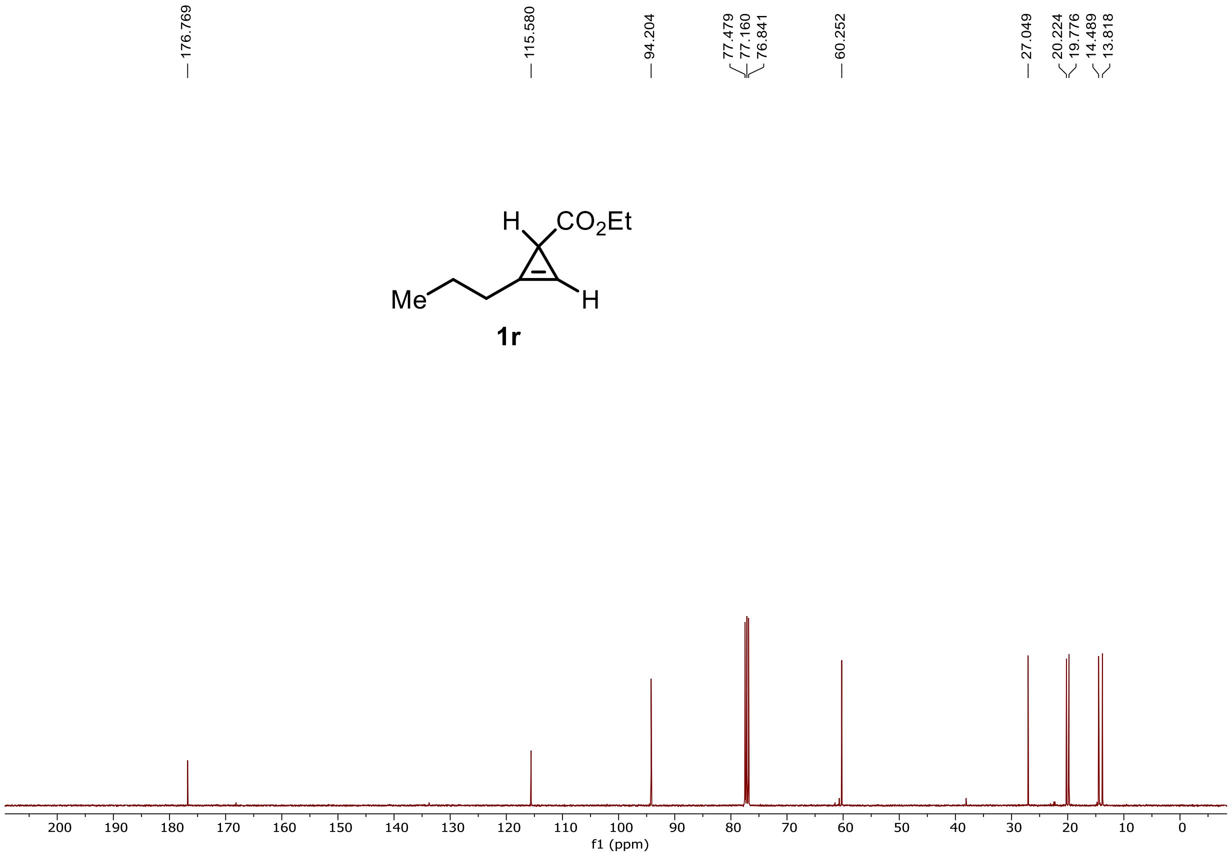

^{1}H NMR (400 MHz, $CDCl_3$) of 1s

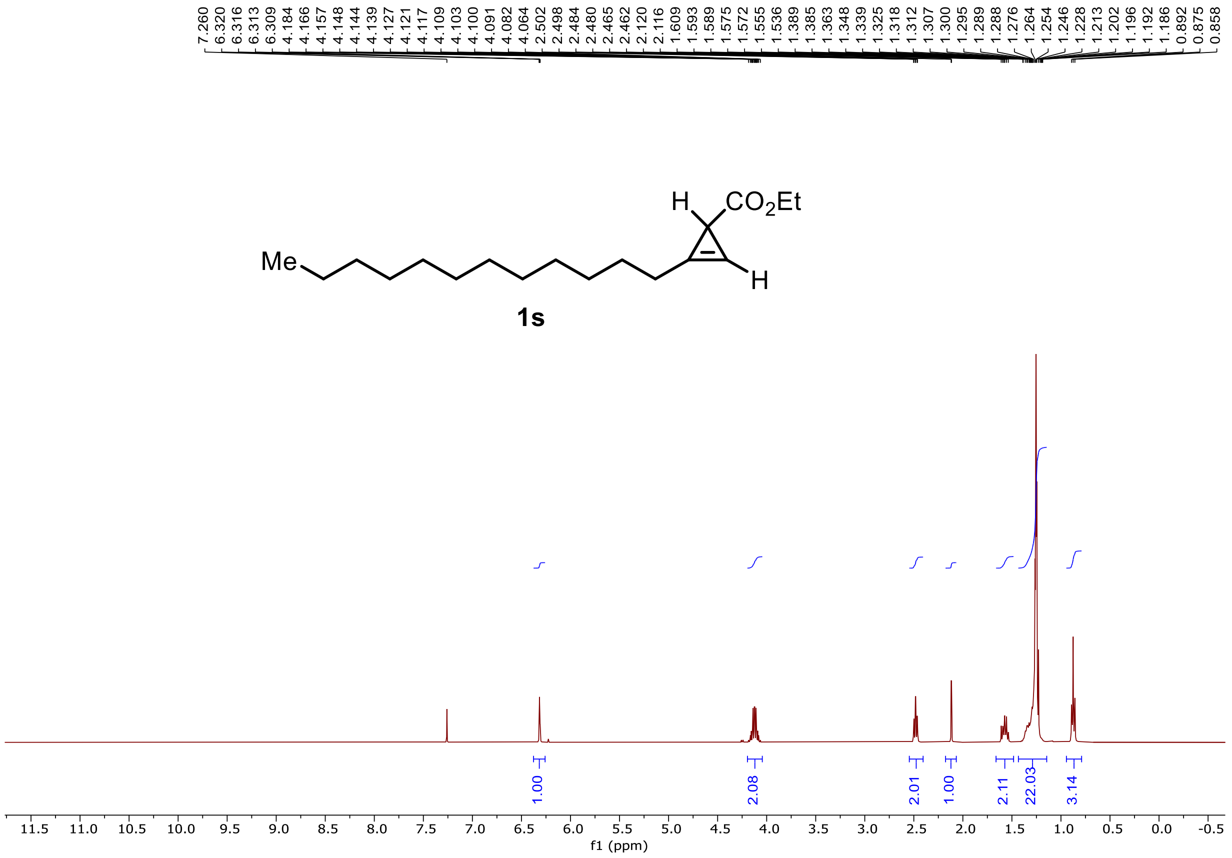

^{13}C NMR (101 MHz, $CDCl_3$) of 1s

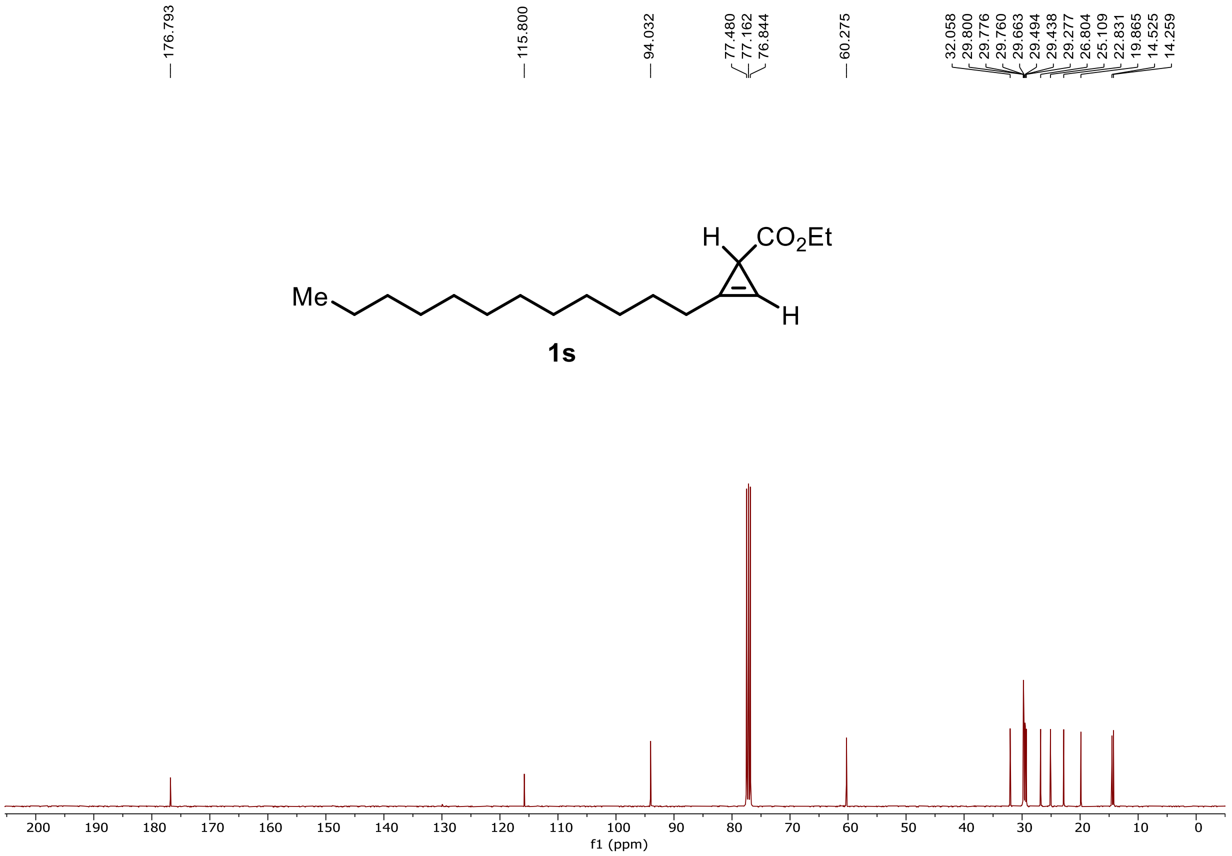

^{1}H NMR (400 MHz, $CDCl_3$) of 1t

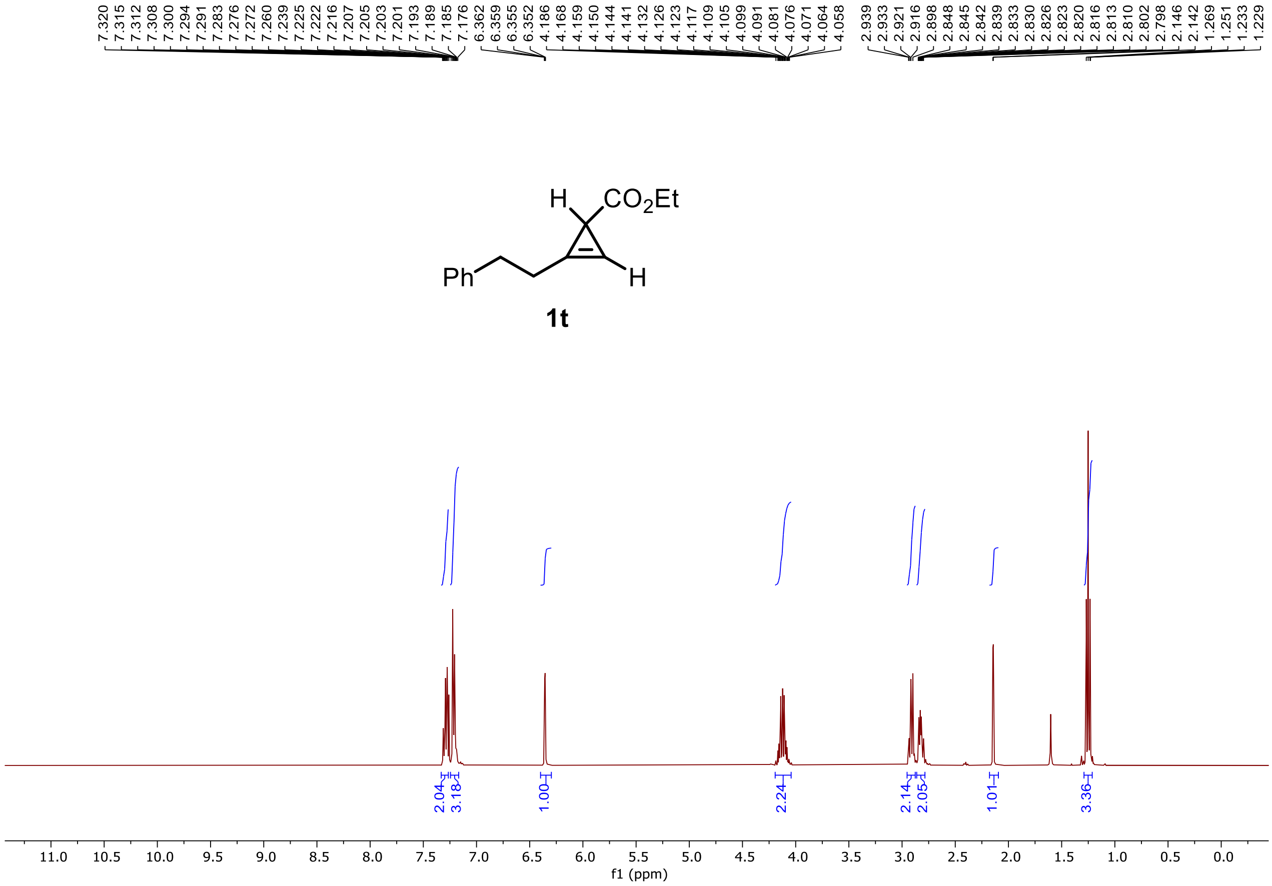

^{1}H NMR (400 MHz, $CDCl_3$) of 1u

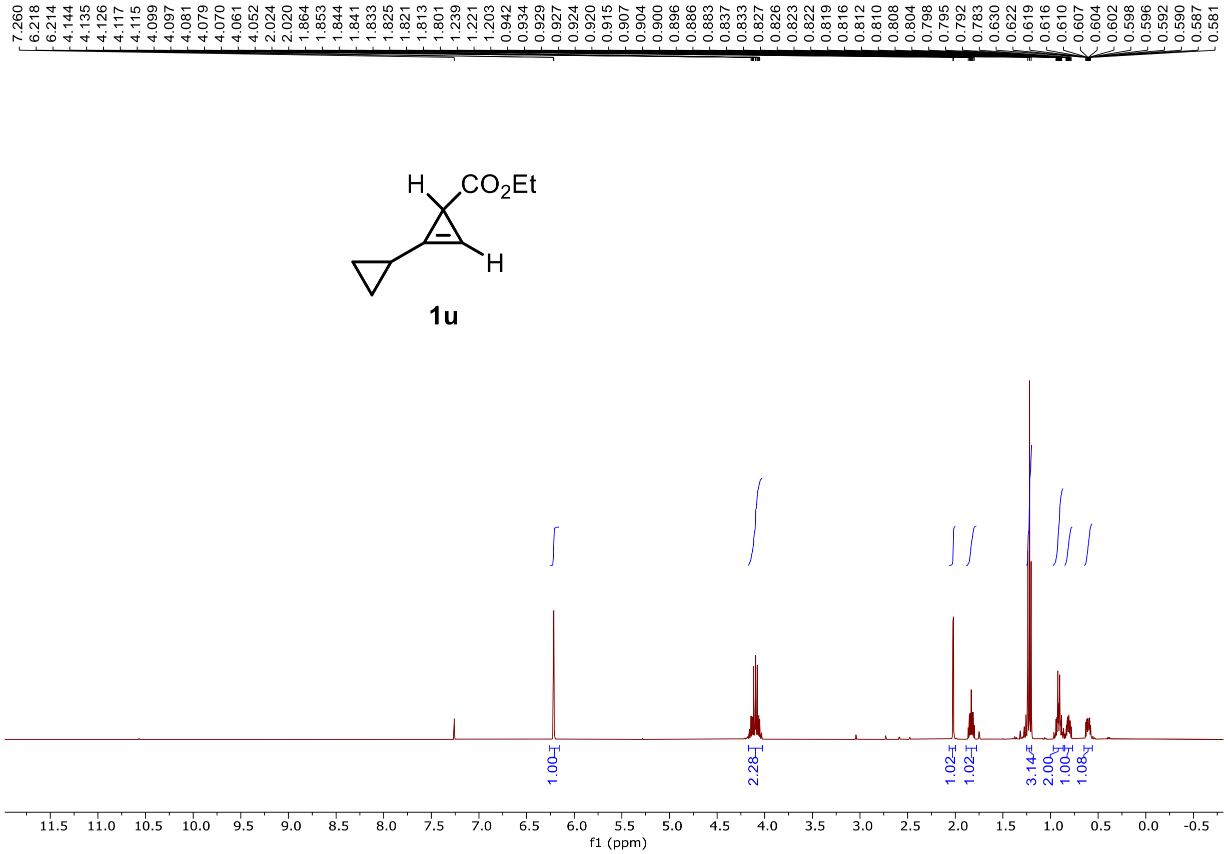

^{13}C NMR (101 MHz, $CDCl_3$) of 1u

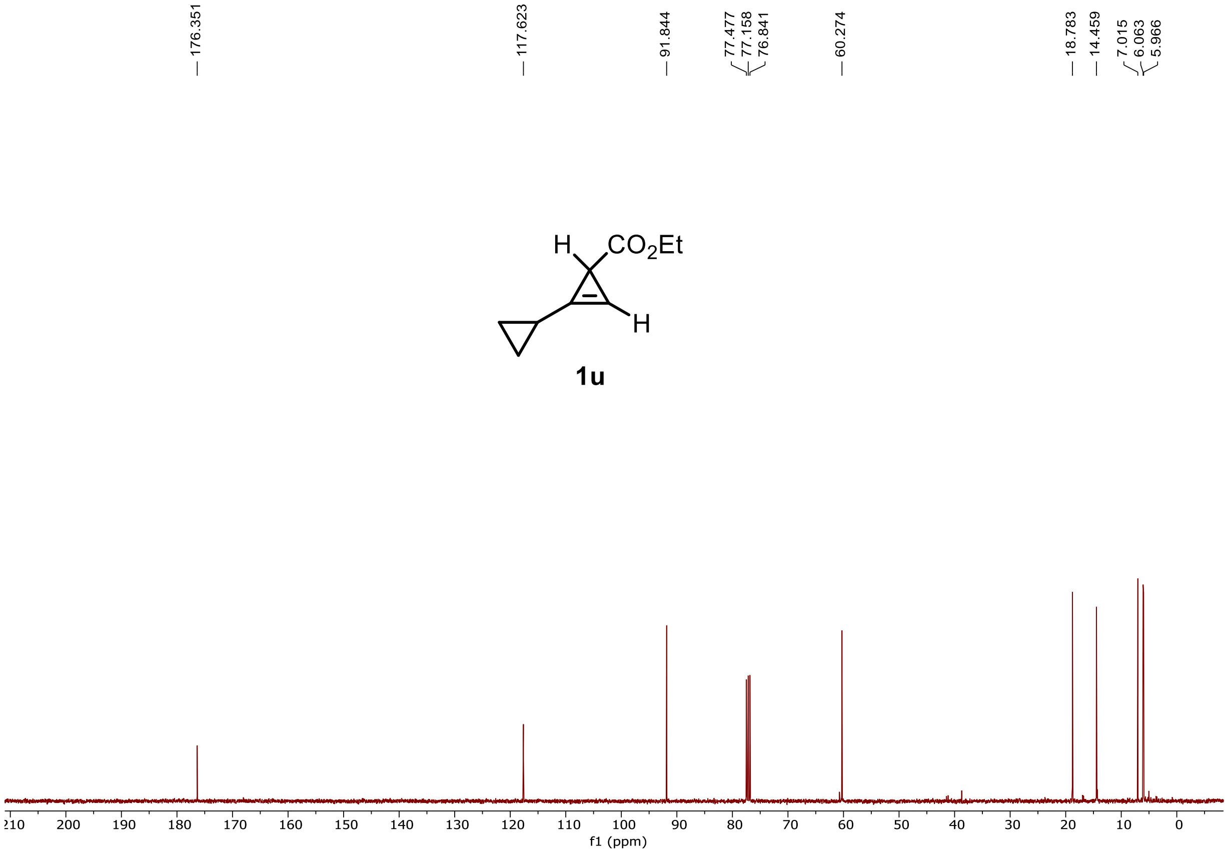

^{1}H NMR (400 MHz, $CDCl_3$) of 1v

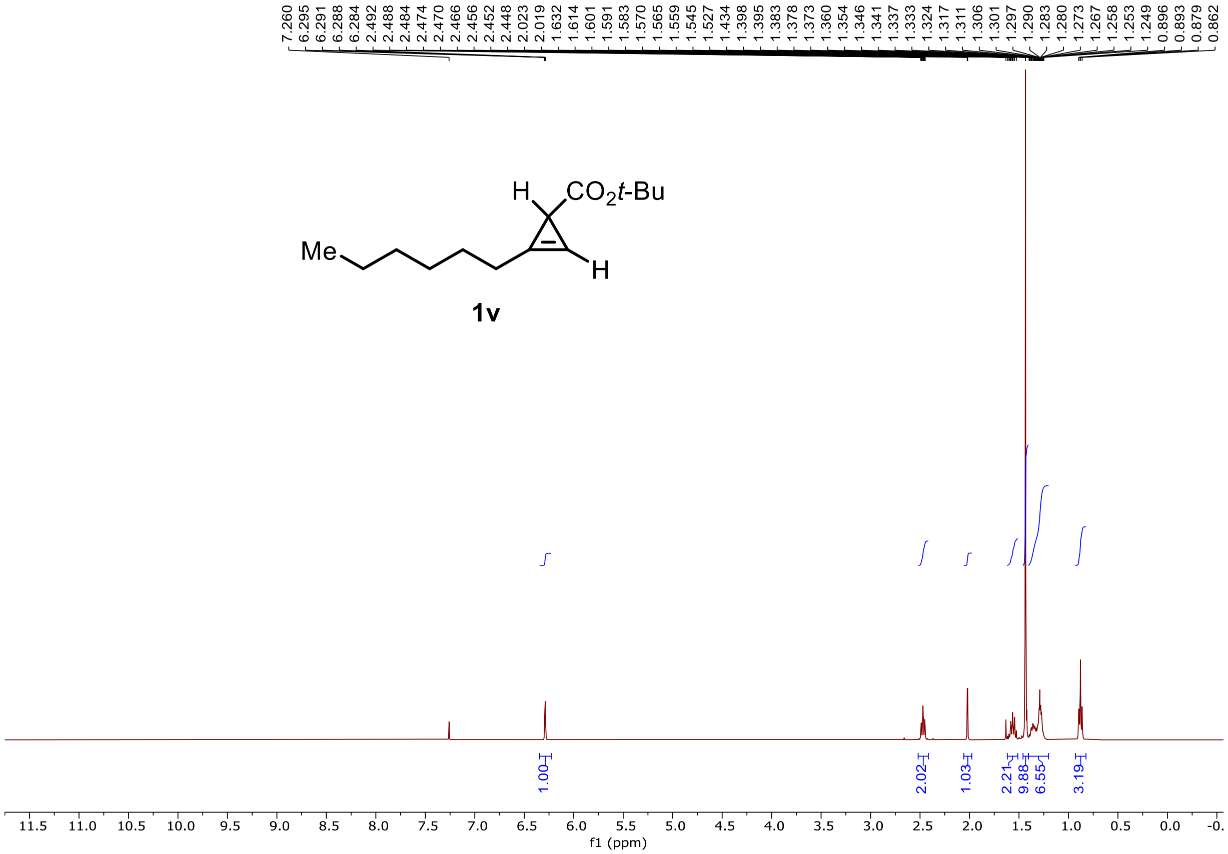

^{13}C NMR (101 MHz, $CDCl_3$) of 1v

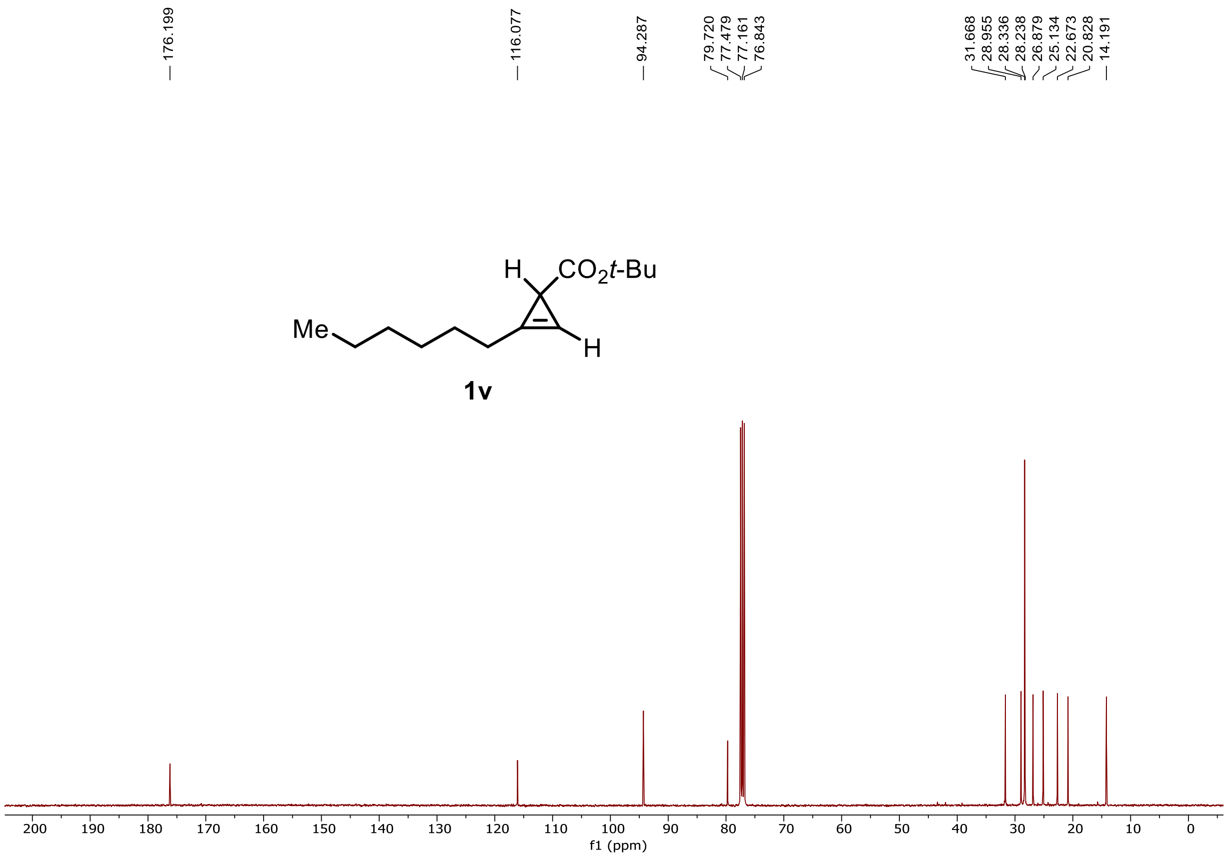

^{1}H NMR (400 MHz, $CDCl_3$) of 1w

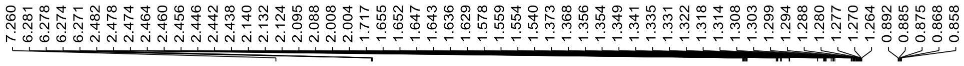

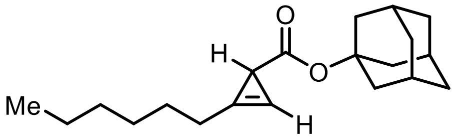

1w

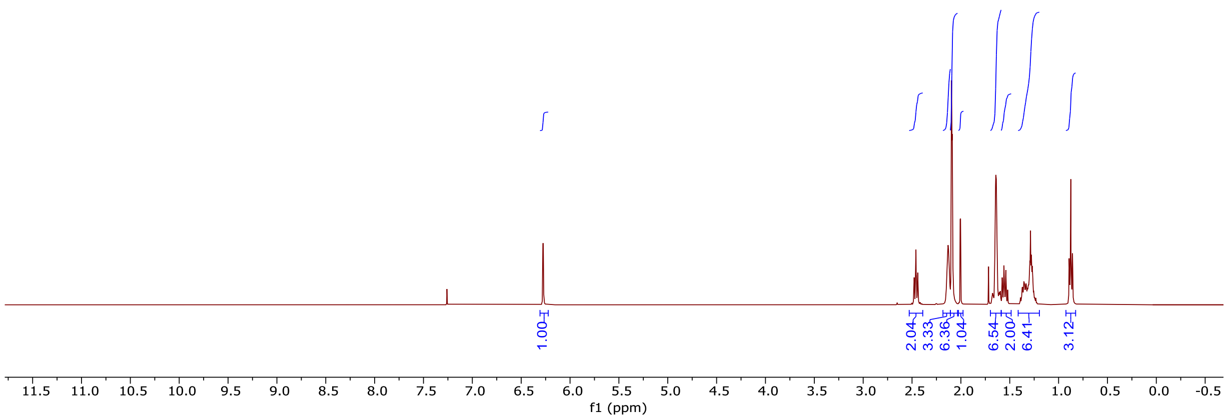

^{13}C NMR (101 MHz, $CDCl_3$) of 1w

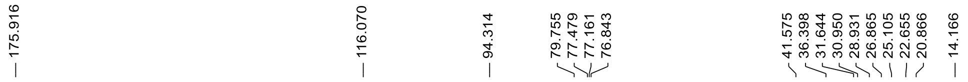

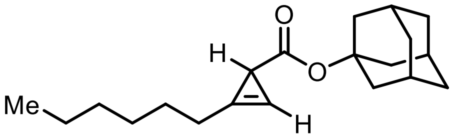

1w

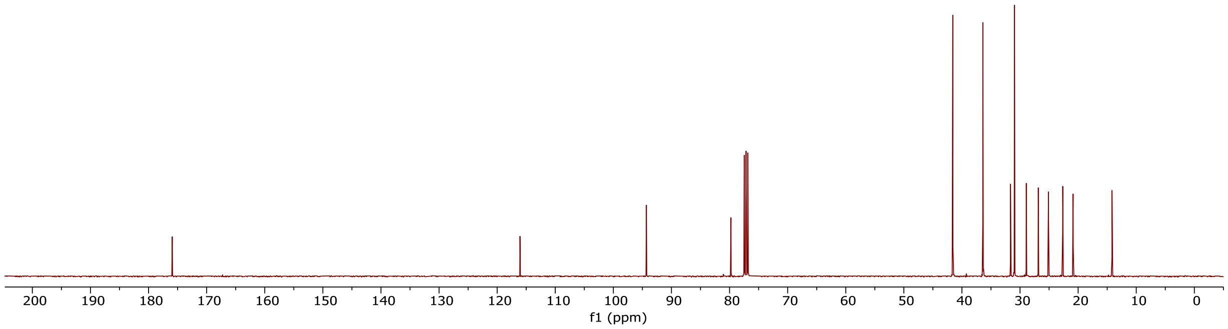

^{1}H NMR (400 MHz, CDCl$_3$) of 1x

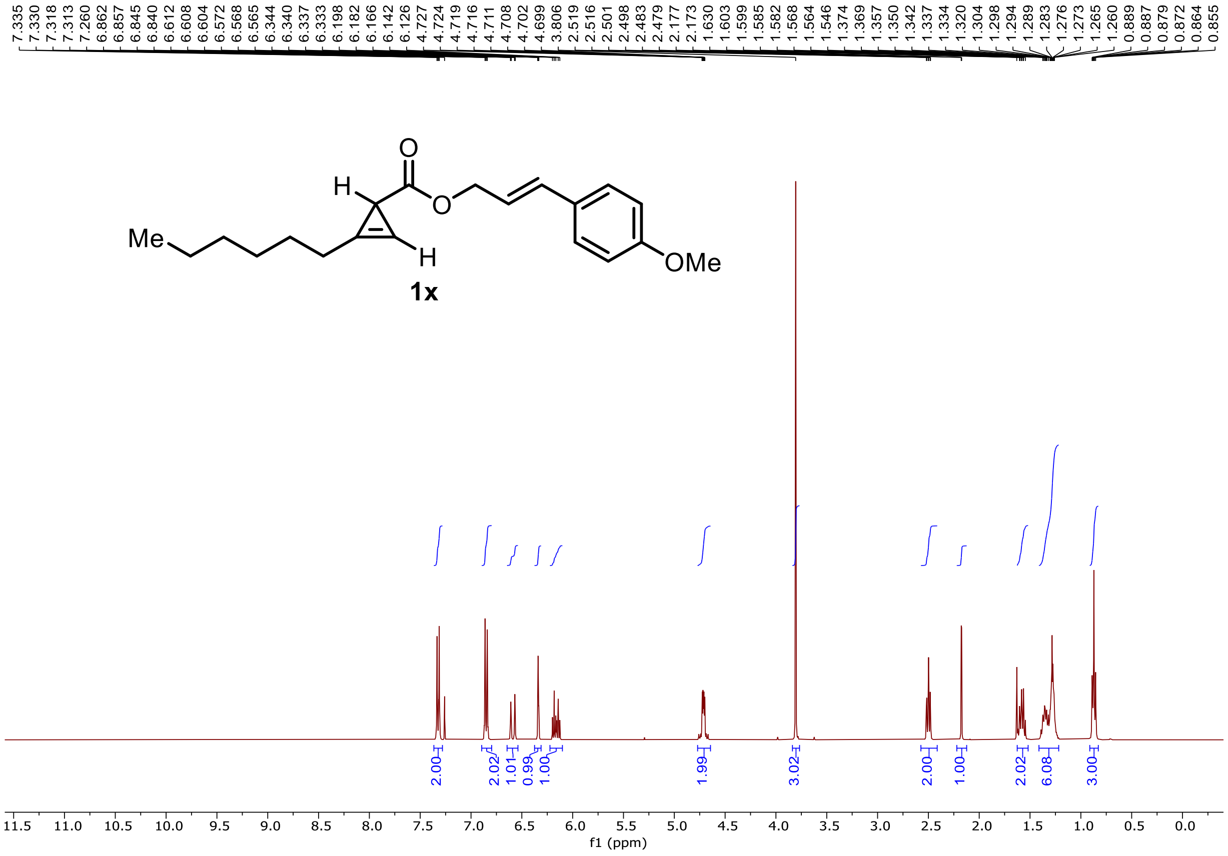

^{13}C NMR (101 MHz, CDCl$_3$) of 1x

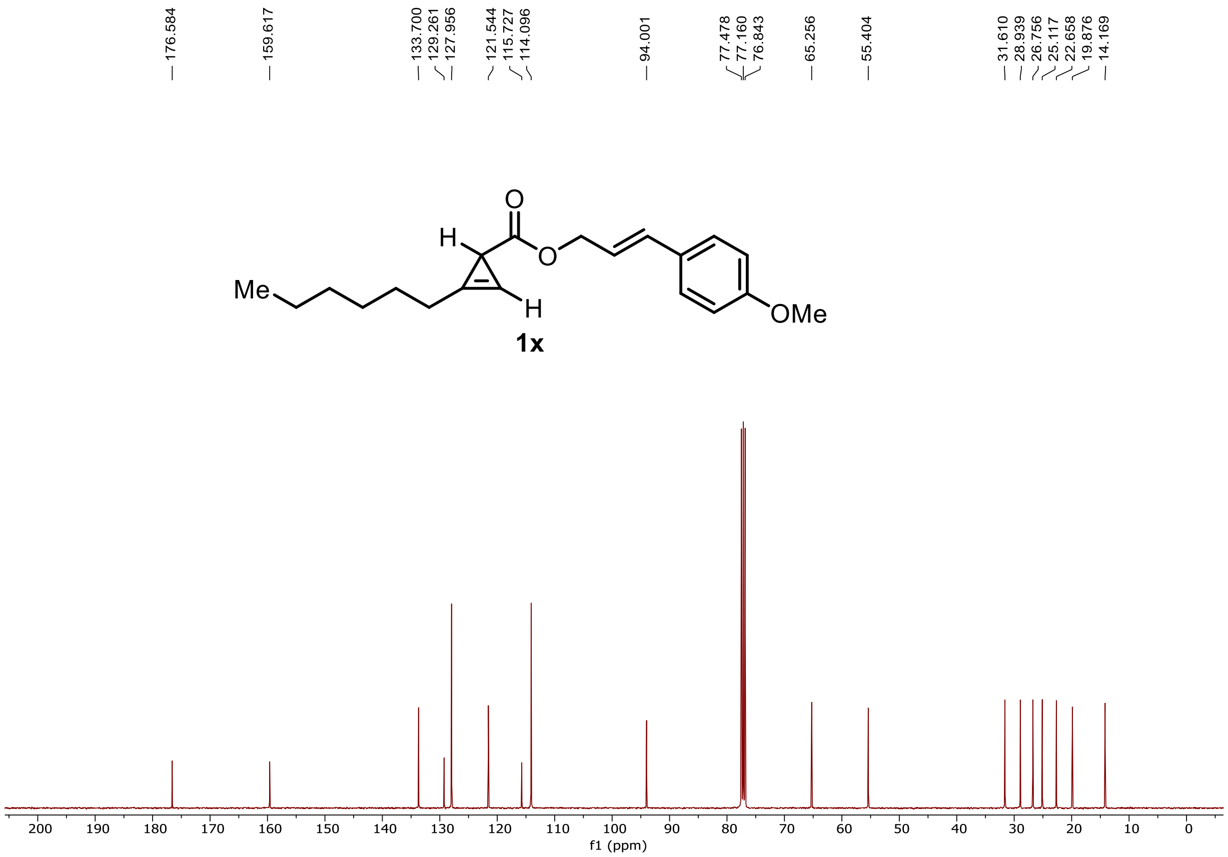

^{1}H NMR (400 MHz, $CDCl_3$) of 1y

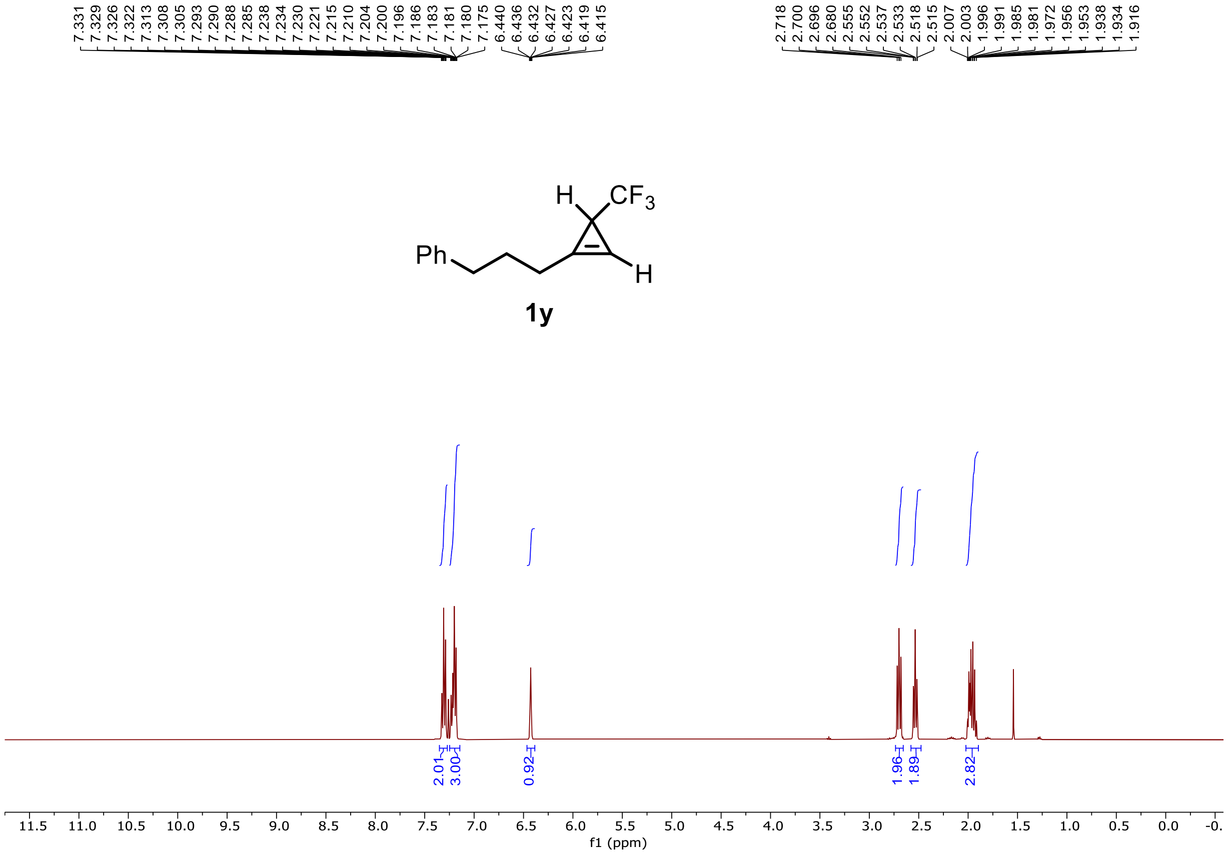

^{13}C NMR (101 MHz, $CDCl_3$) of 1y

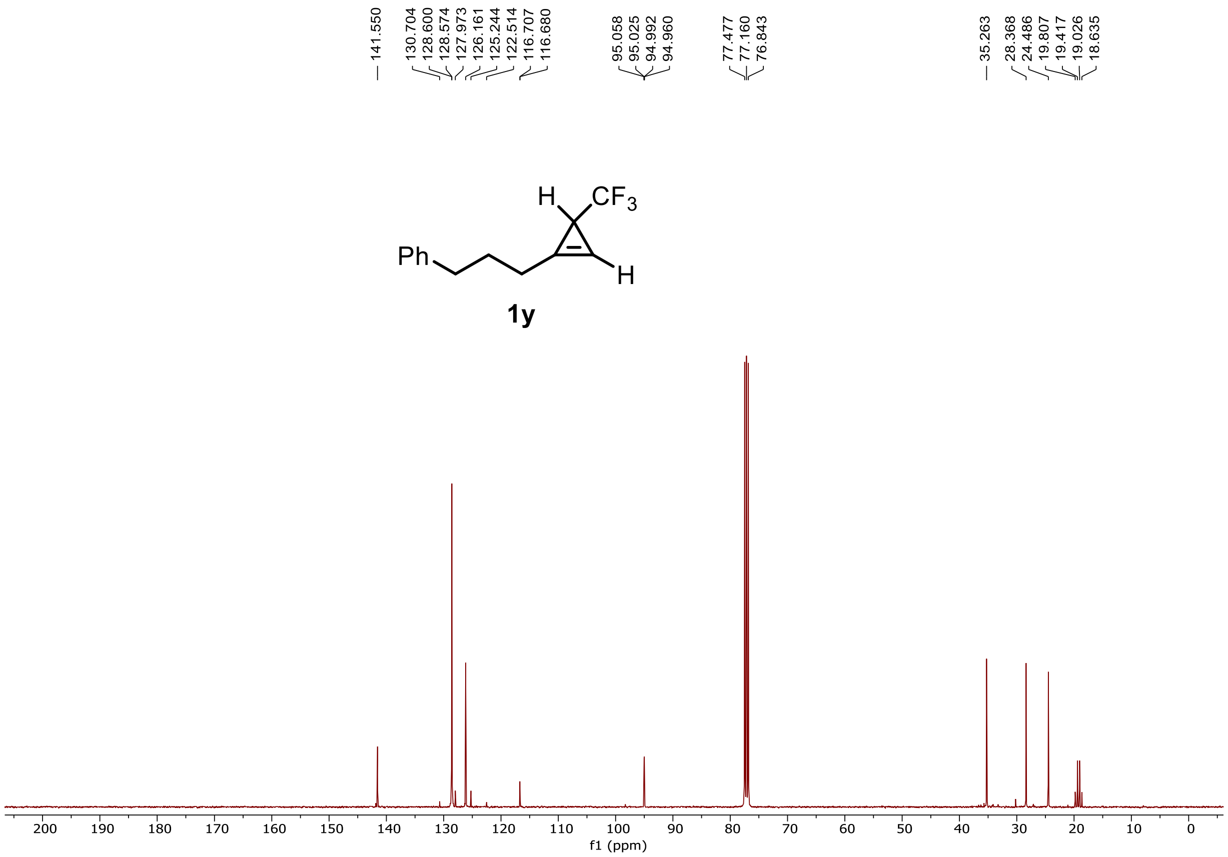

^{19}F NMR (377 MHz, CDCl$_3$) of 1y

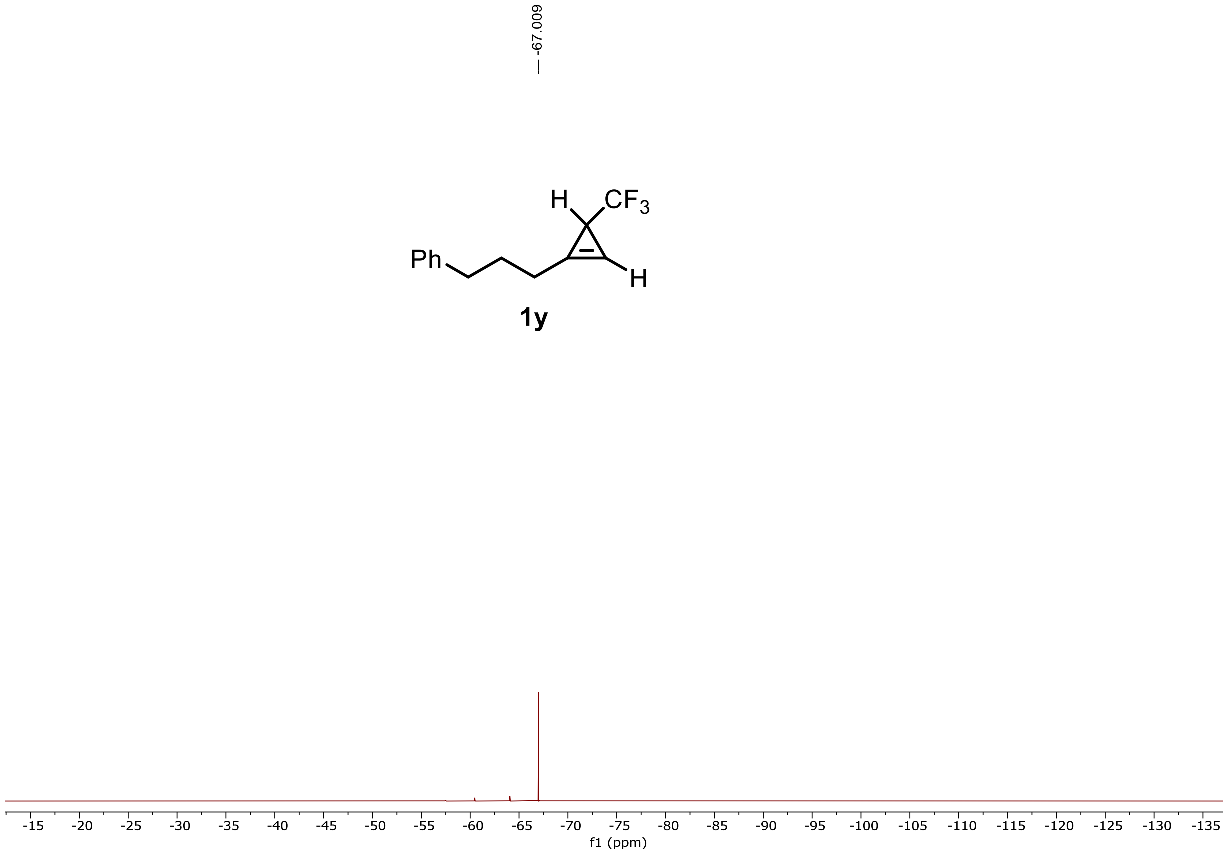

^{1}H NMR (400 MHz, Acetone-d_6) of hypervalent iodine precursor I^{III}-Cl

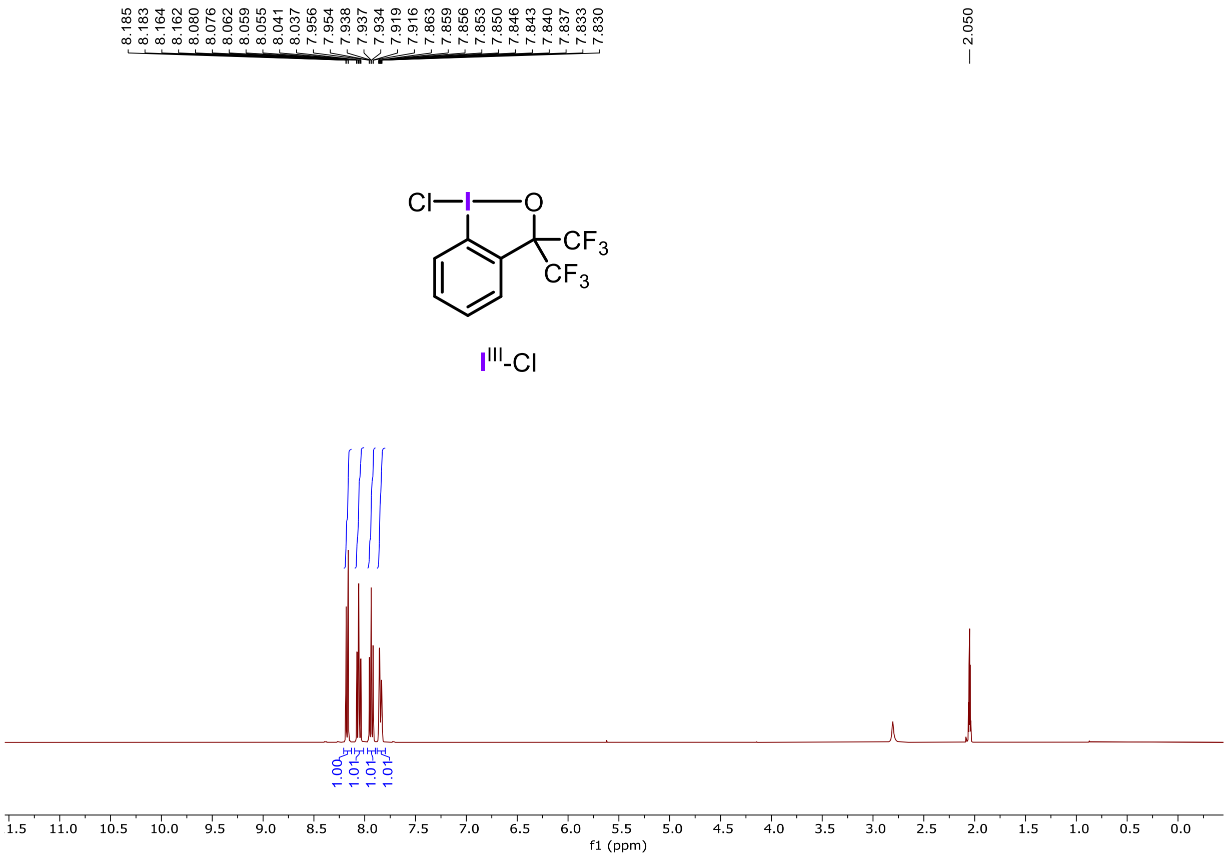

^{13}C NMR (101 MHz, Acetone-d_6) of hypervalent iodine precursor I^{III}-Cl

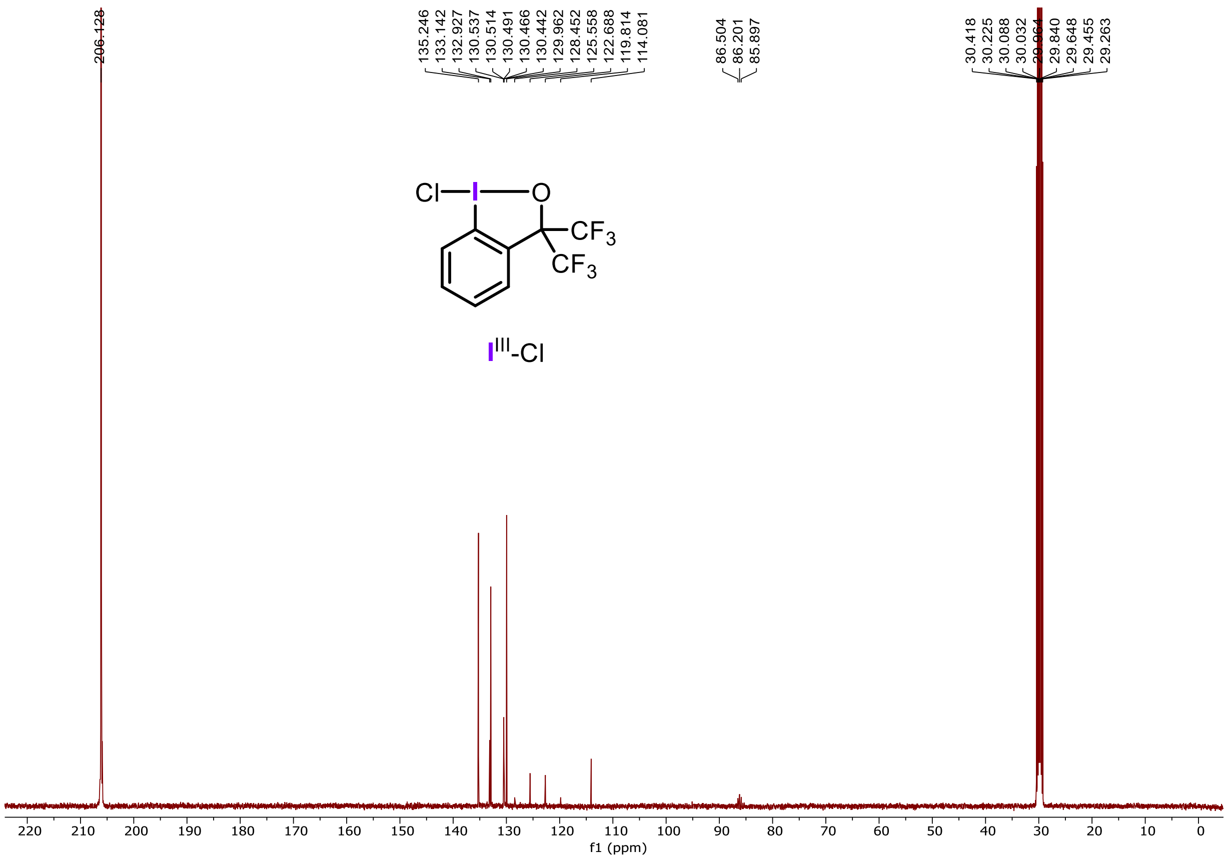

^{19}F NMR (377 MHz, Acetone-d_6) of hypervalent iodine precursor I^{III}-Cl

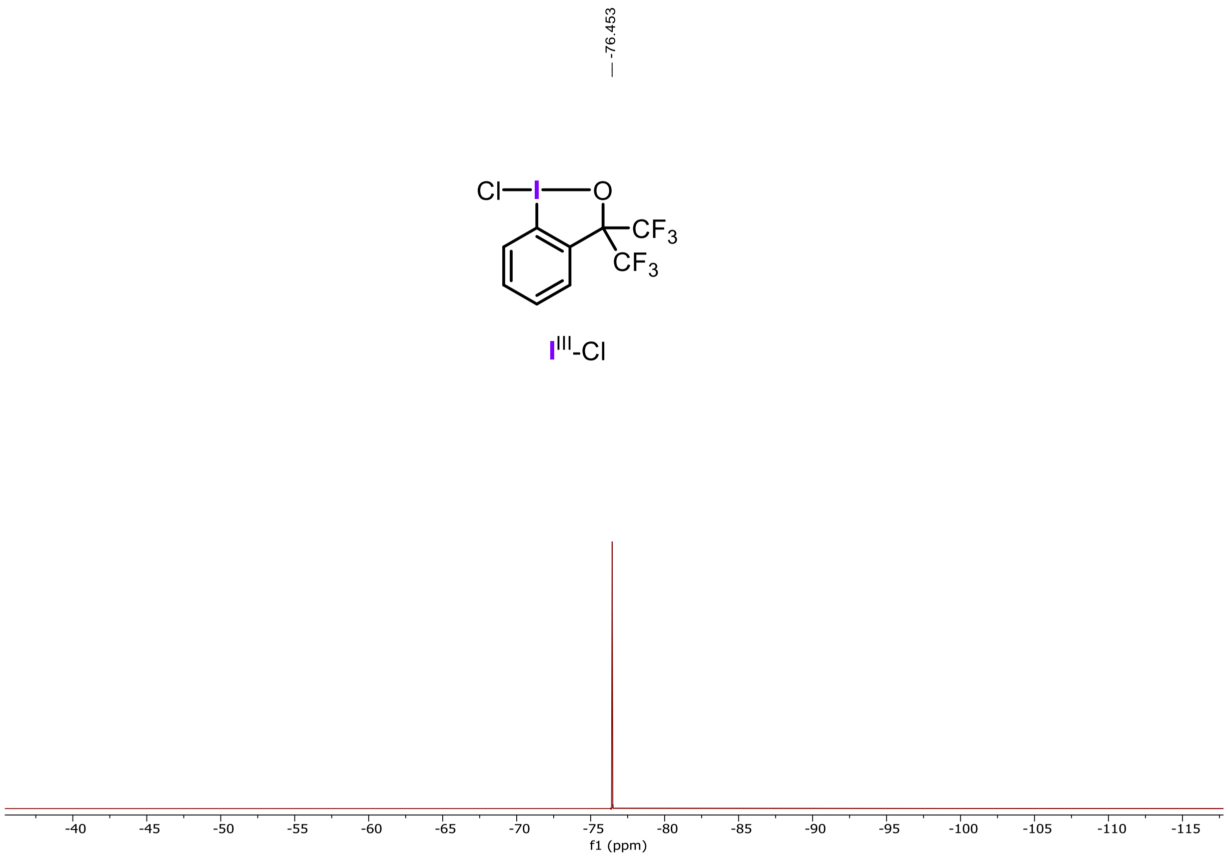

^{1}H NMR (400 MHz, $CDCl_3$) of hypervalent iodine precursor I^{III}-OAc

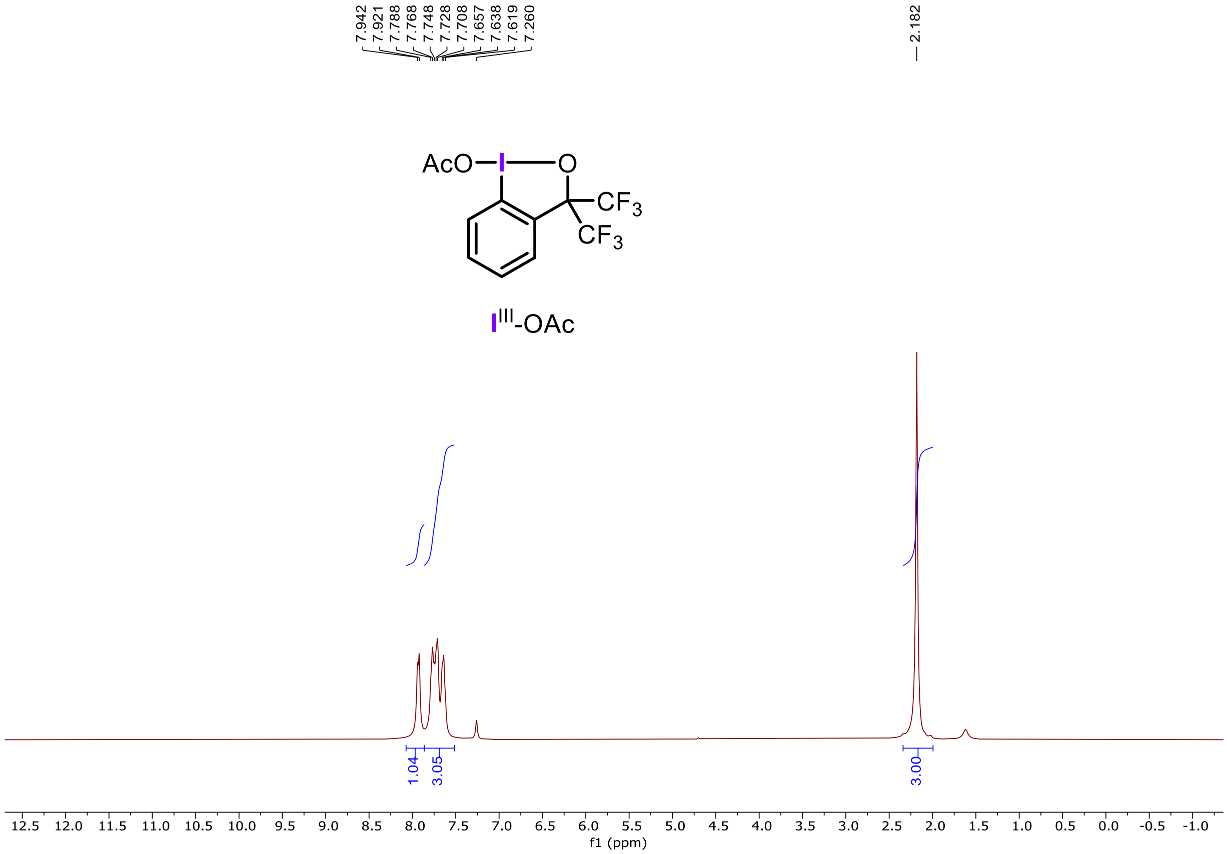

^{13}C NMR (101 MHz, $CDCl_3$) of hypervalent iodine precursor I^{III}-OAc

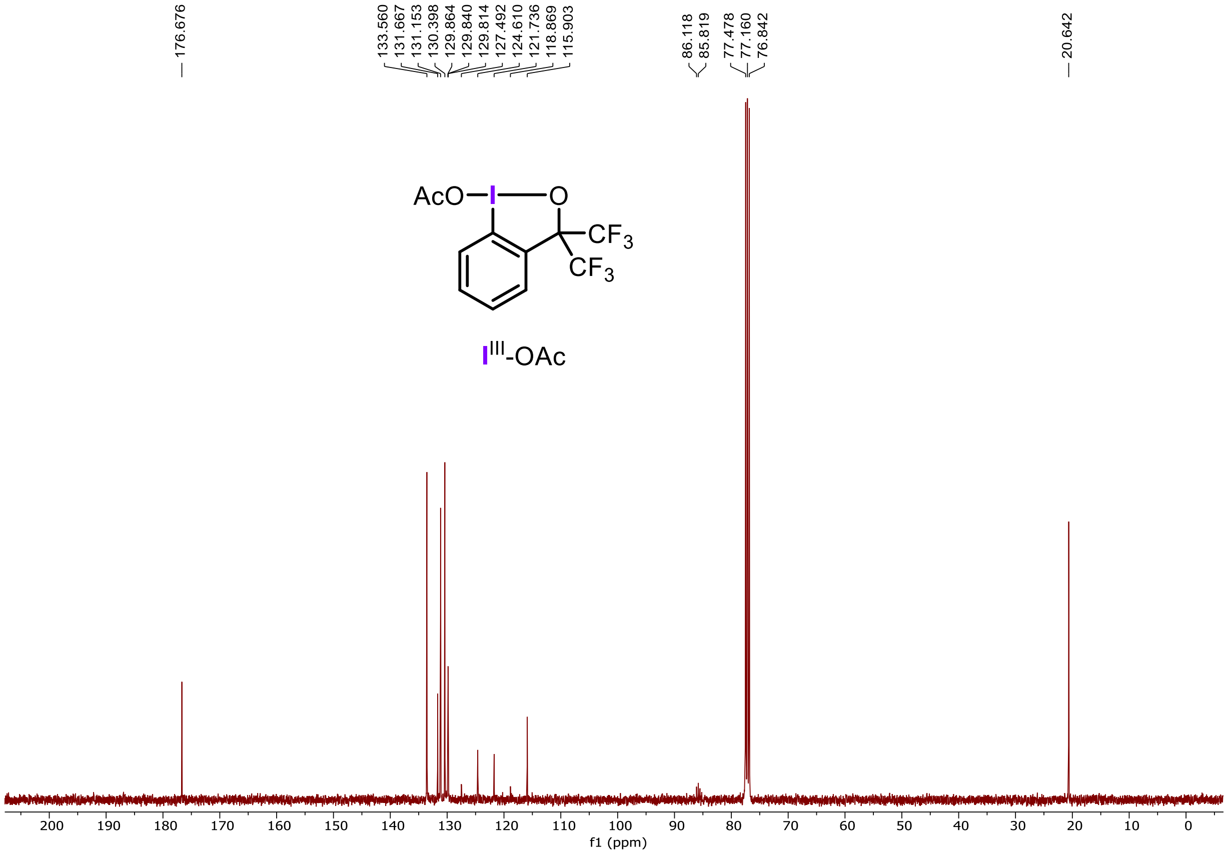

^{19}F NMR (377 MHz, $CDCl_3$) of hypervalent iodine precursor I^{III}-OAc

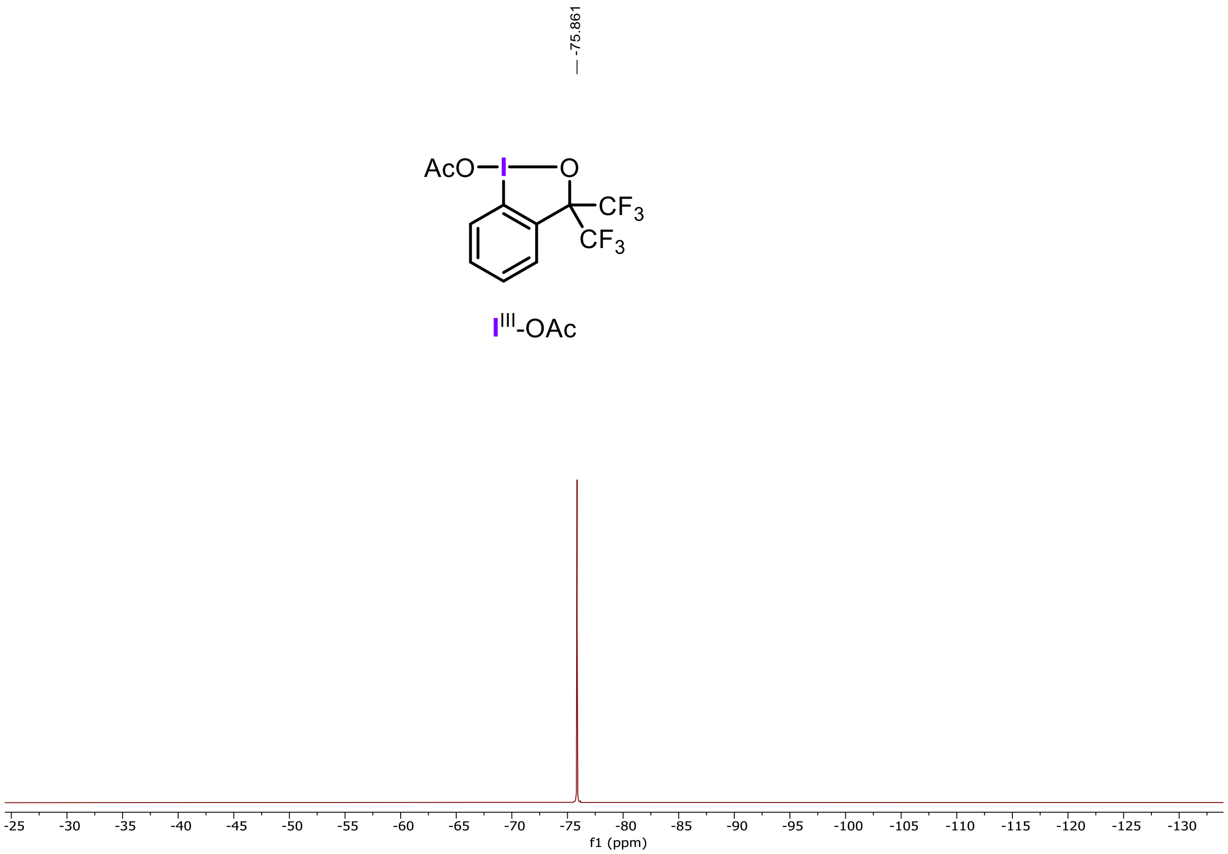

^{1}H NMR (400 MHz, $CDCl_3$) of 2a

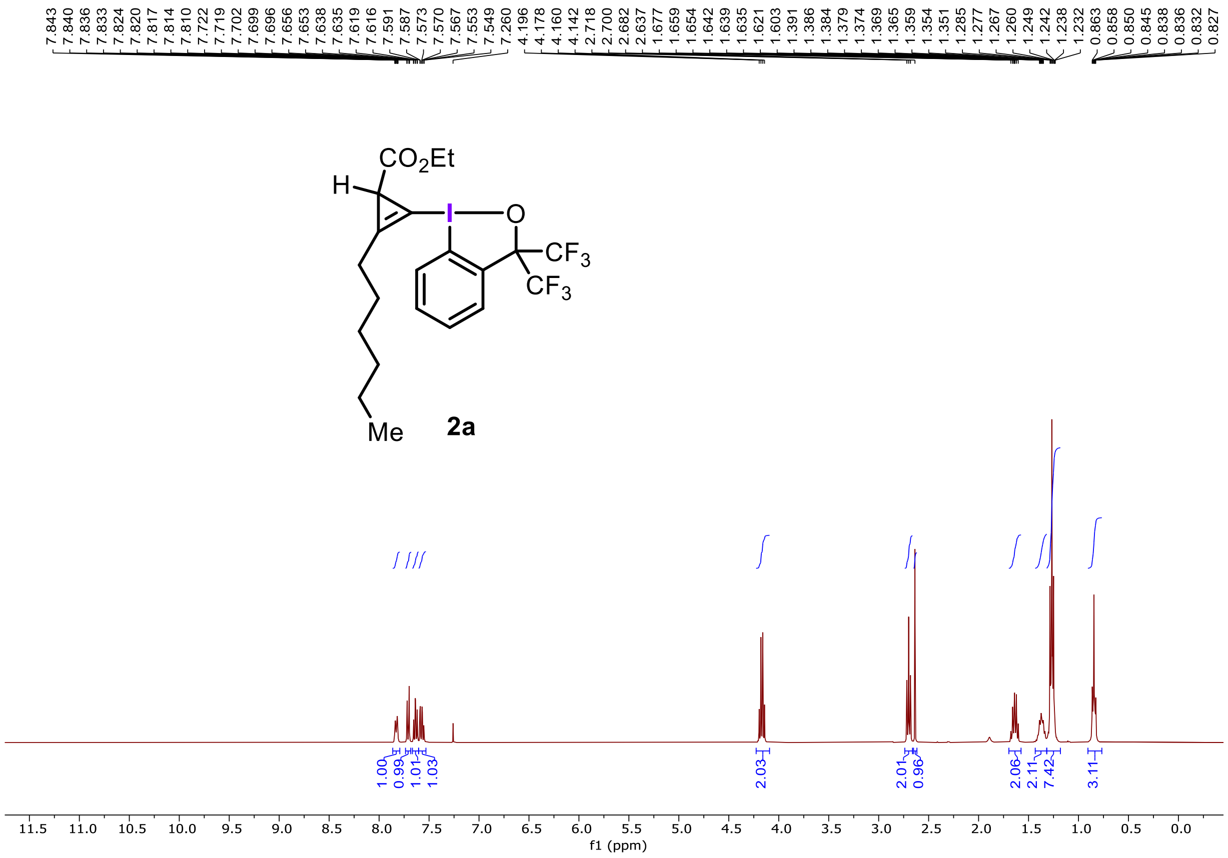

^{13}C NMR (101 MHz, $CDCl_3$) of 2a

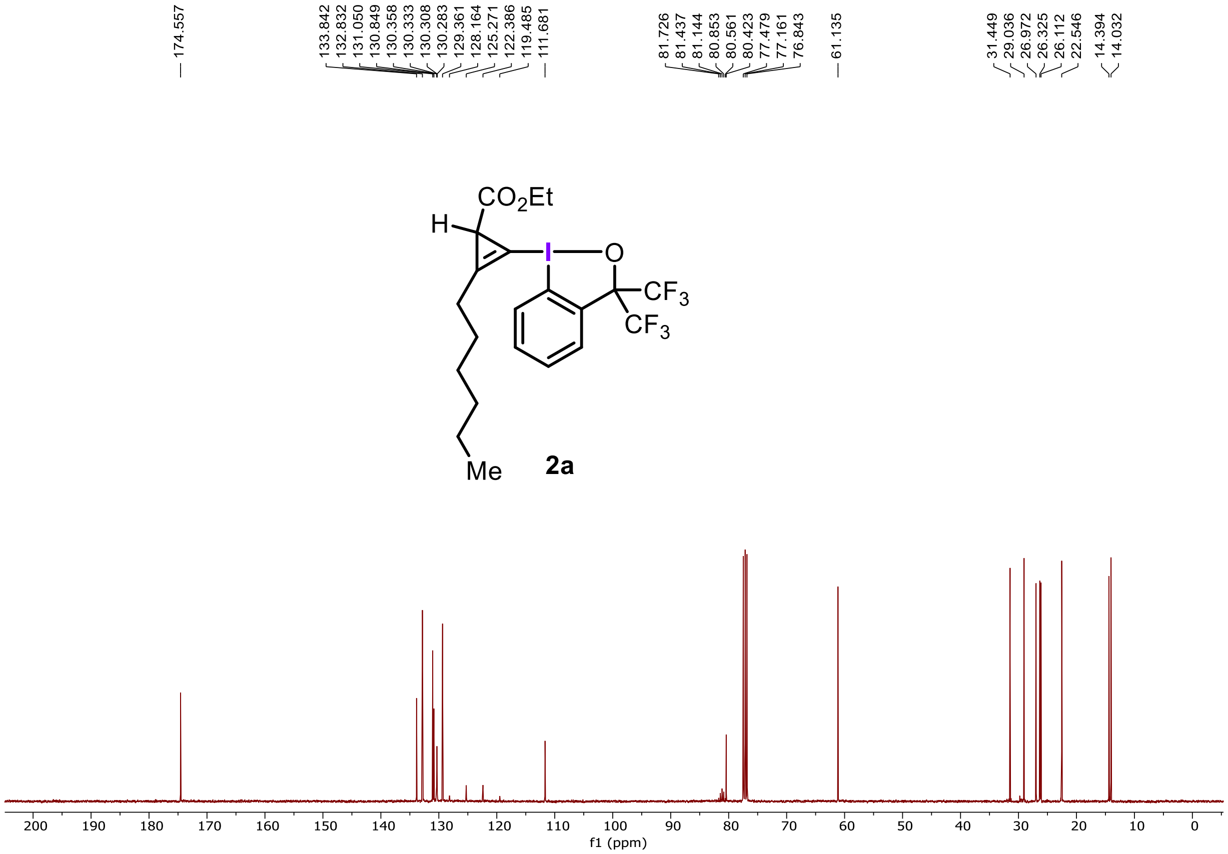

^{19}F NMR (377 MHz, $CDCl_3$) of 2a

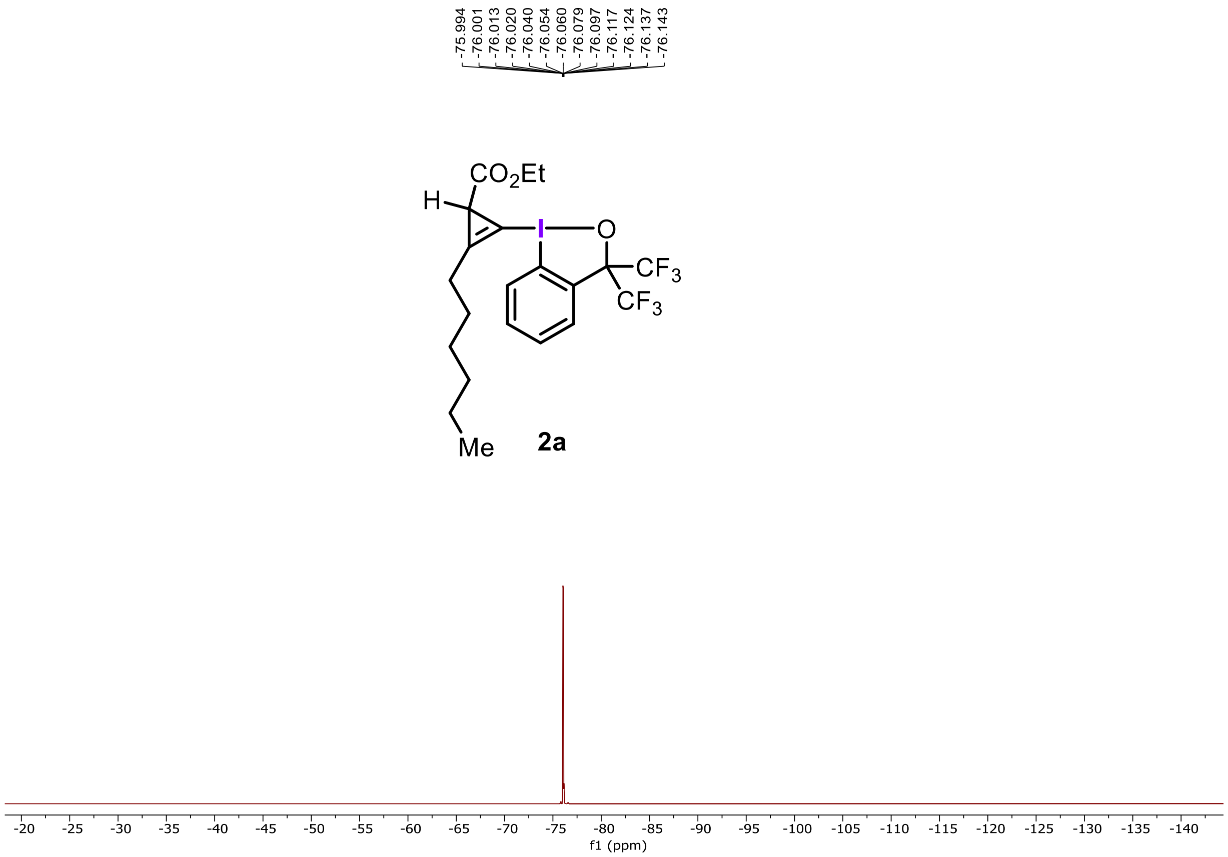

^{1}H NMR (400 MHz, $CDCl_3$) of 2b

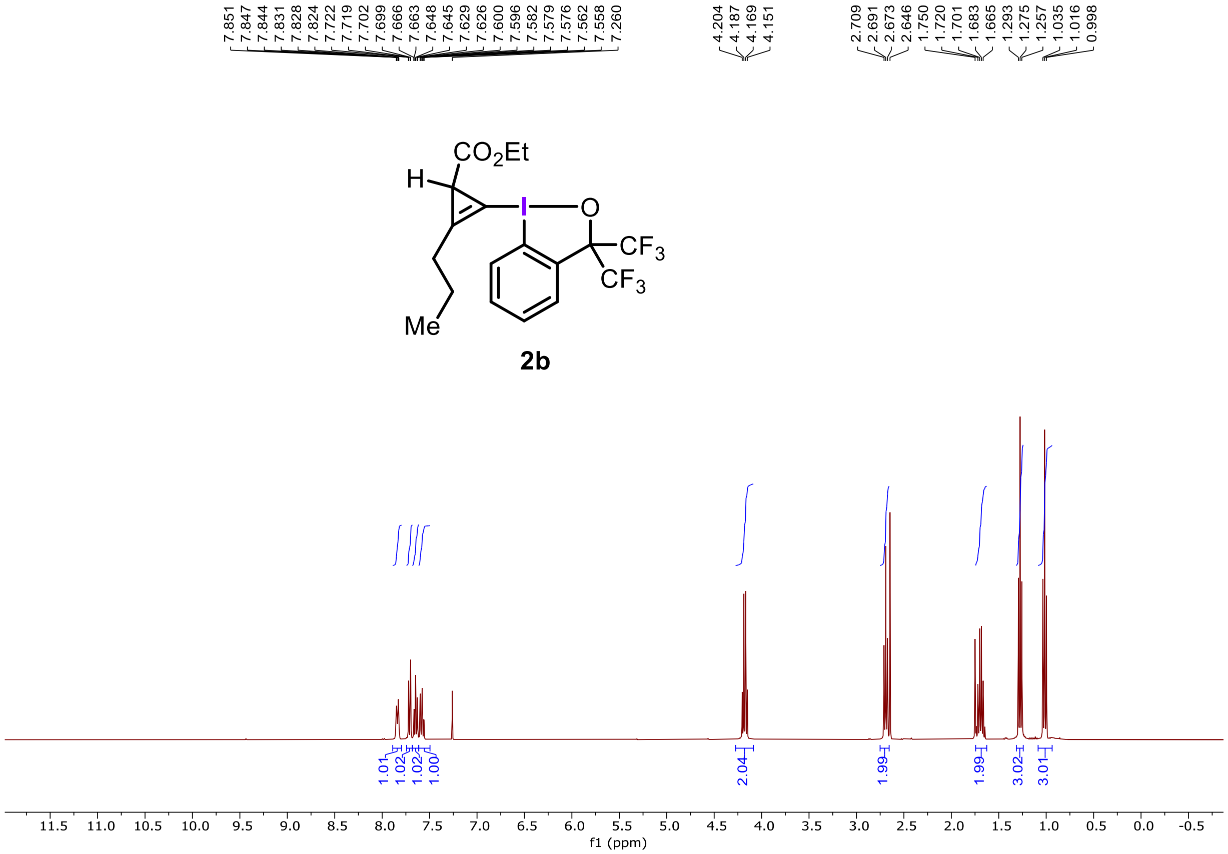

^{13}C NMR (101 MHz, $CDCl_3$) of 2b

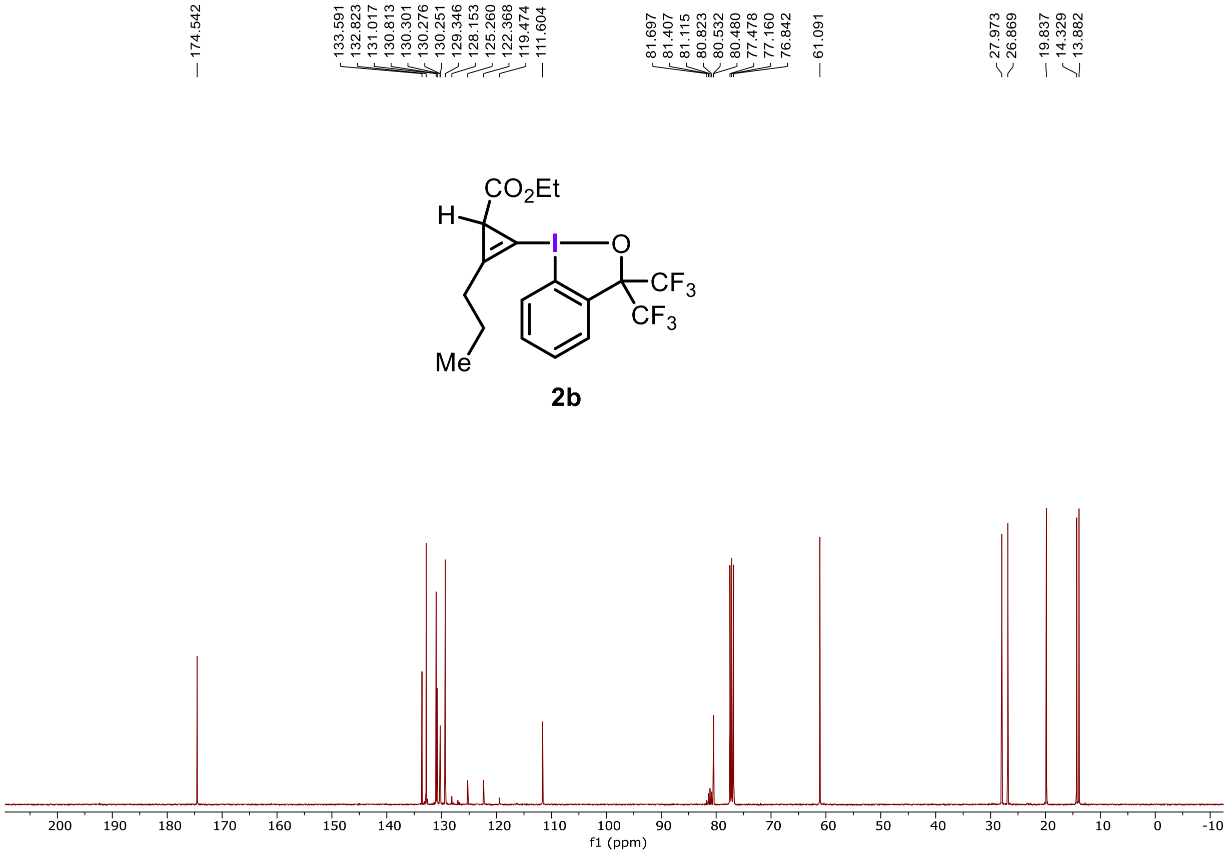

^{19}F NMR (377 MHz, $CDCl_3$) of 2b

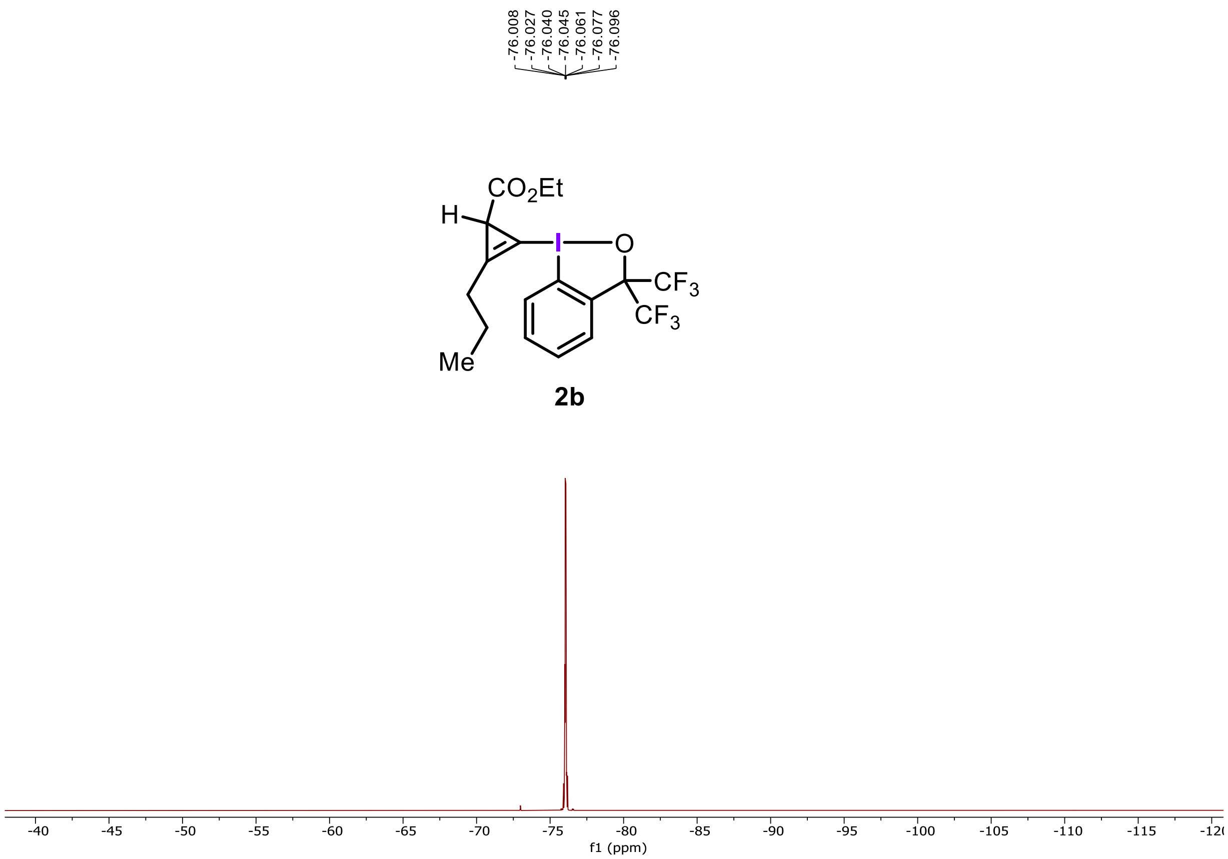

^{1}H NMR (400 MHz, $CDCl_3$) of 2c

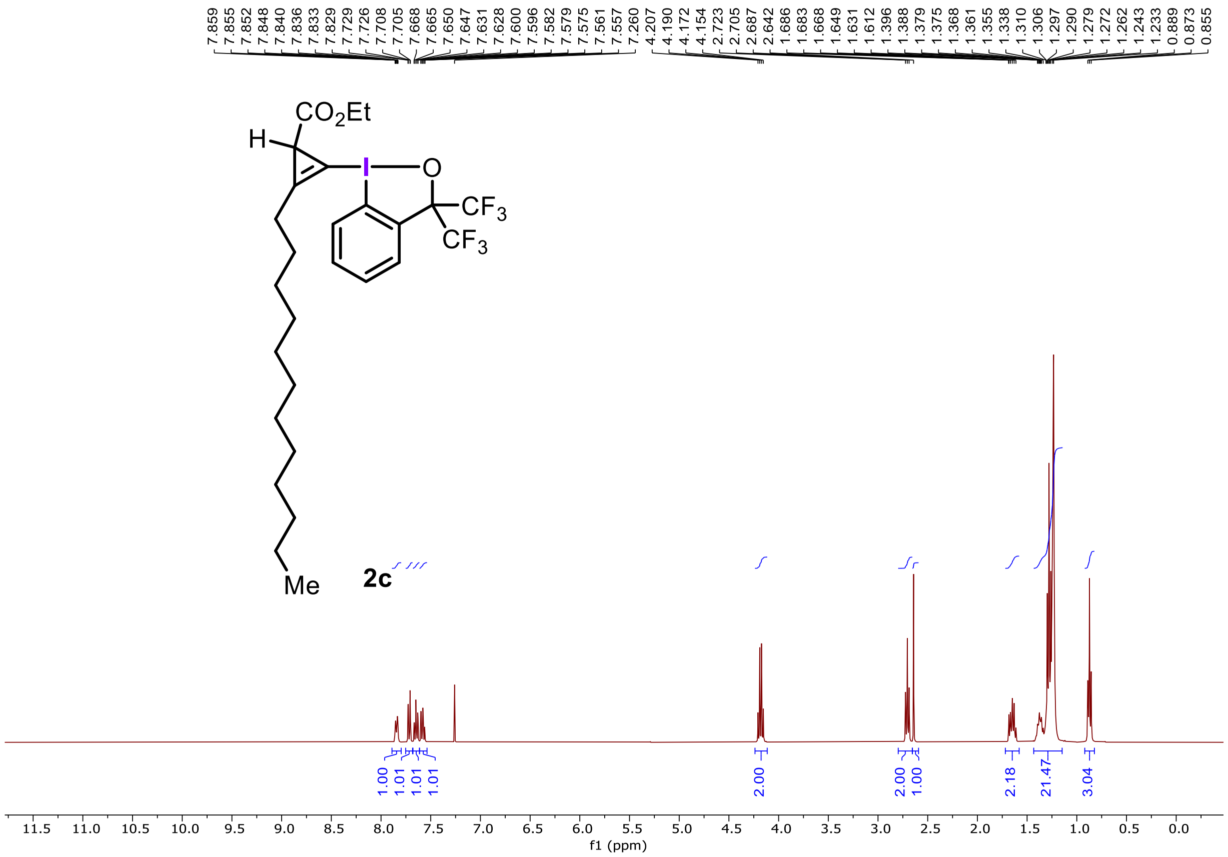

^{13}C NMR (101 MHz, $CDCl_3$) of 2c

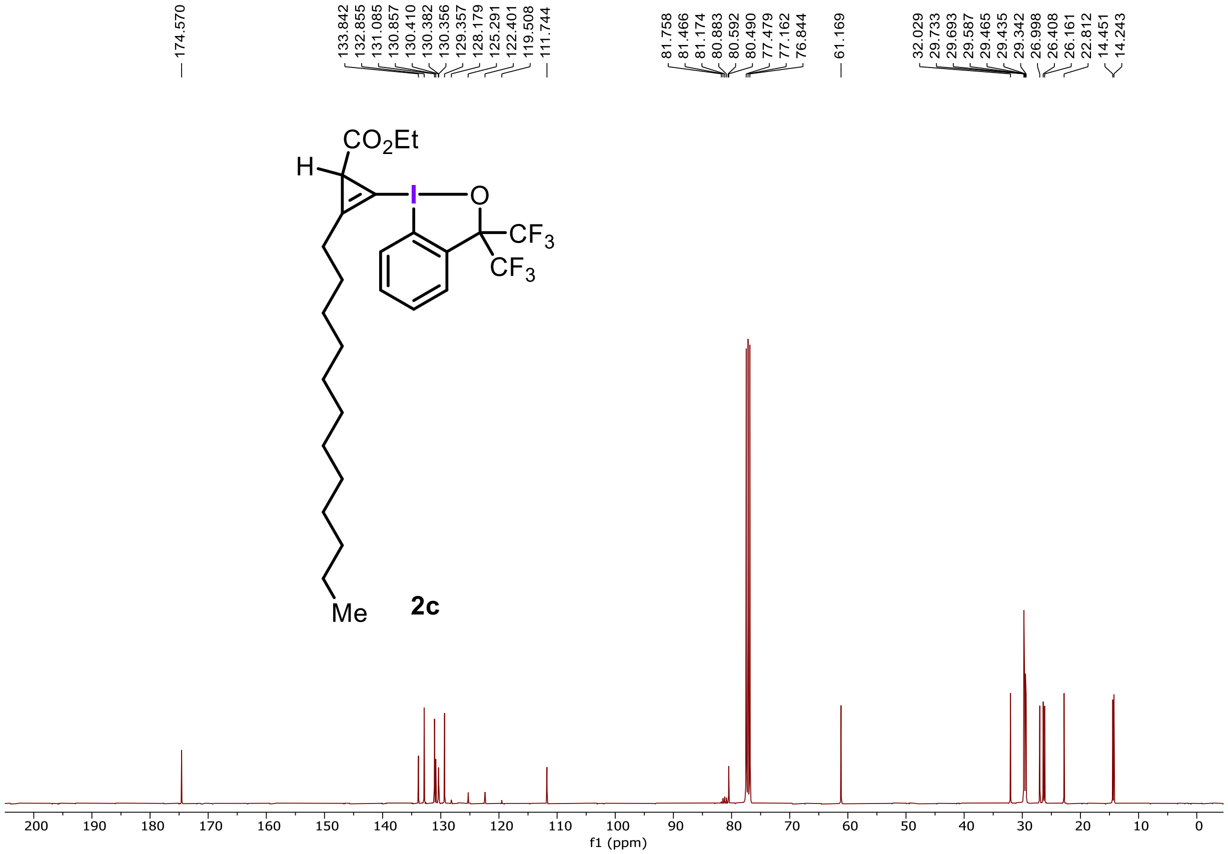

^{19}F NMR (377 MHz, $CDCl_3$) of 2c

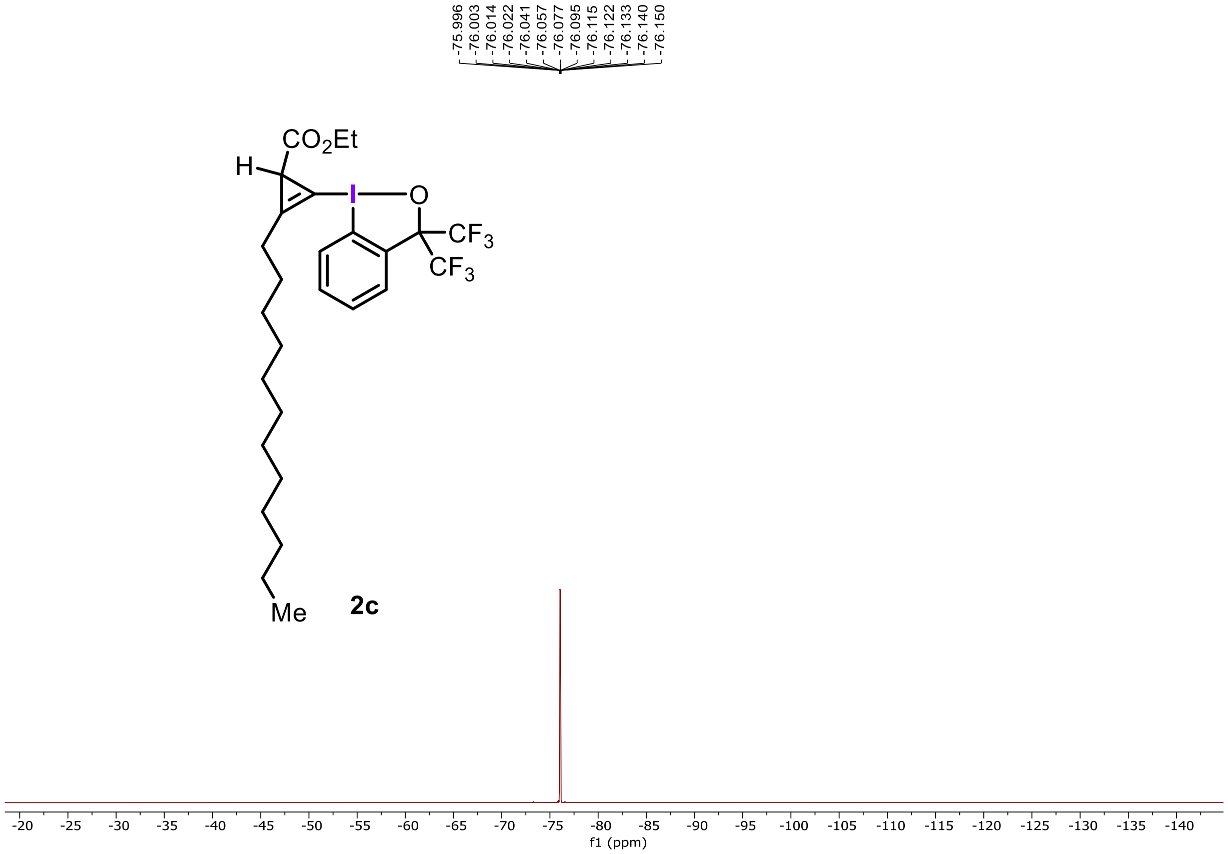

^{1}H NMR (400 MHz, $CDCl_3$) of 2d

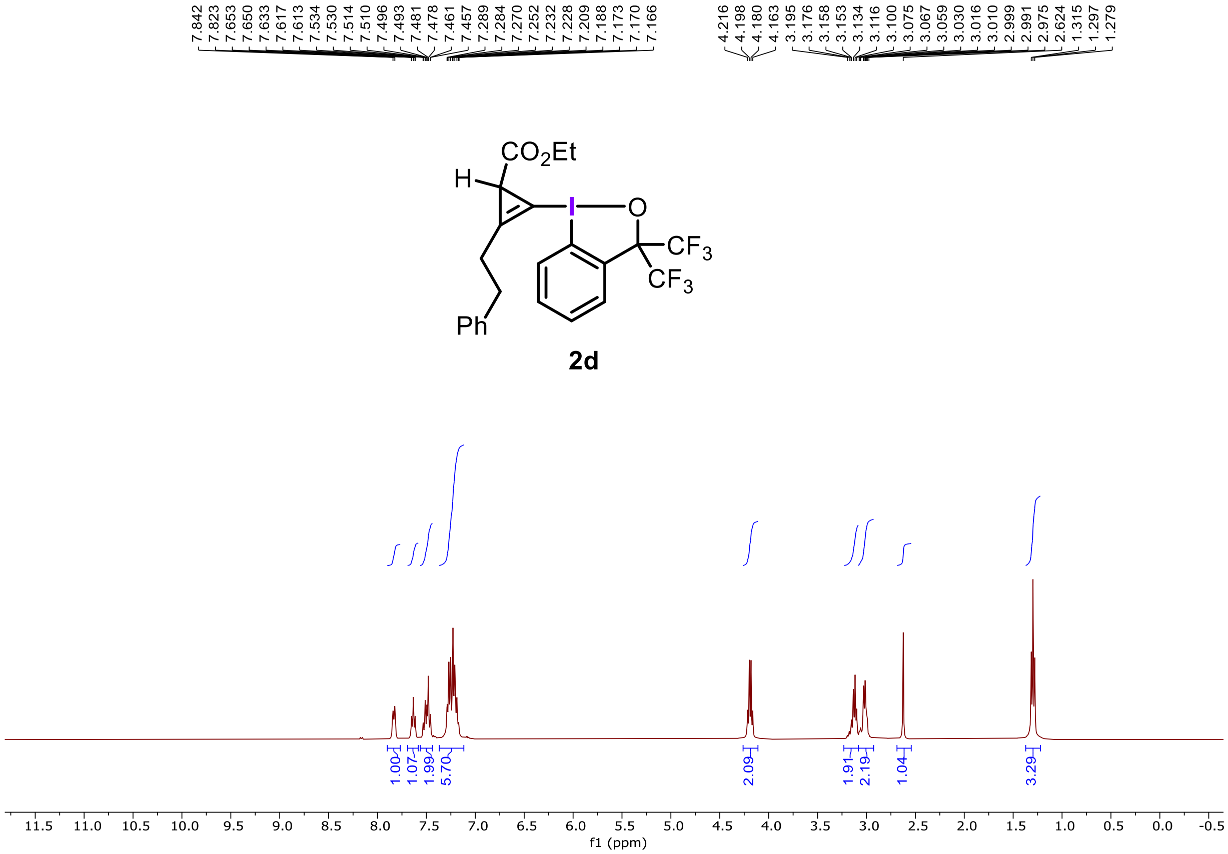

^{13}C NMR (101 MHz, $CDCl_3$) of 2d

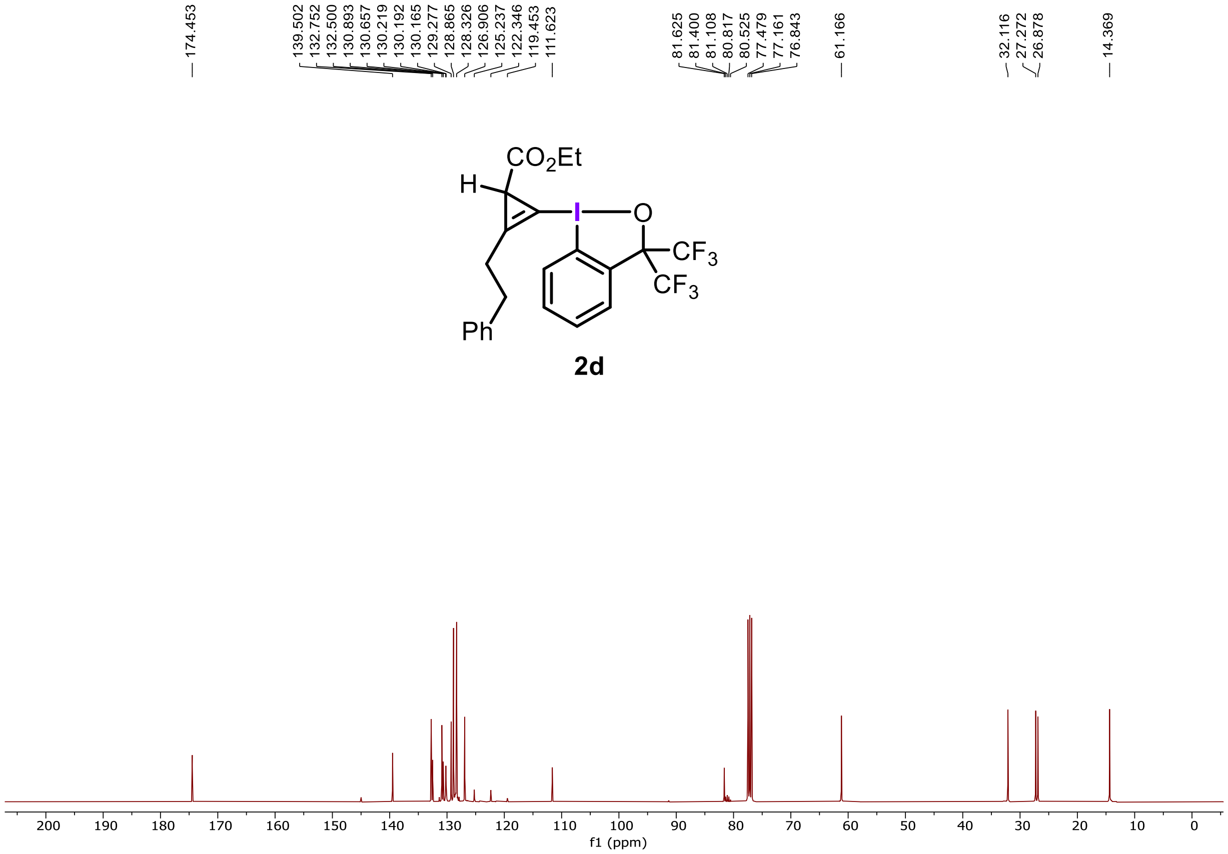

^{19}F NMR (377 MHz, $CDCl_3$) of 2d

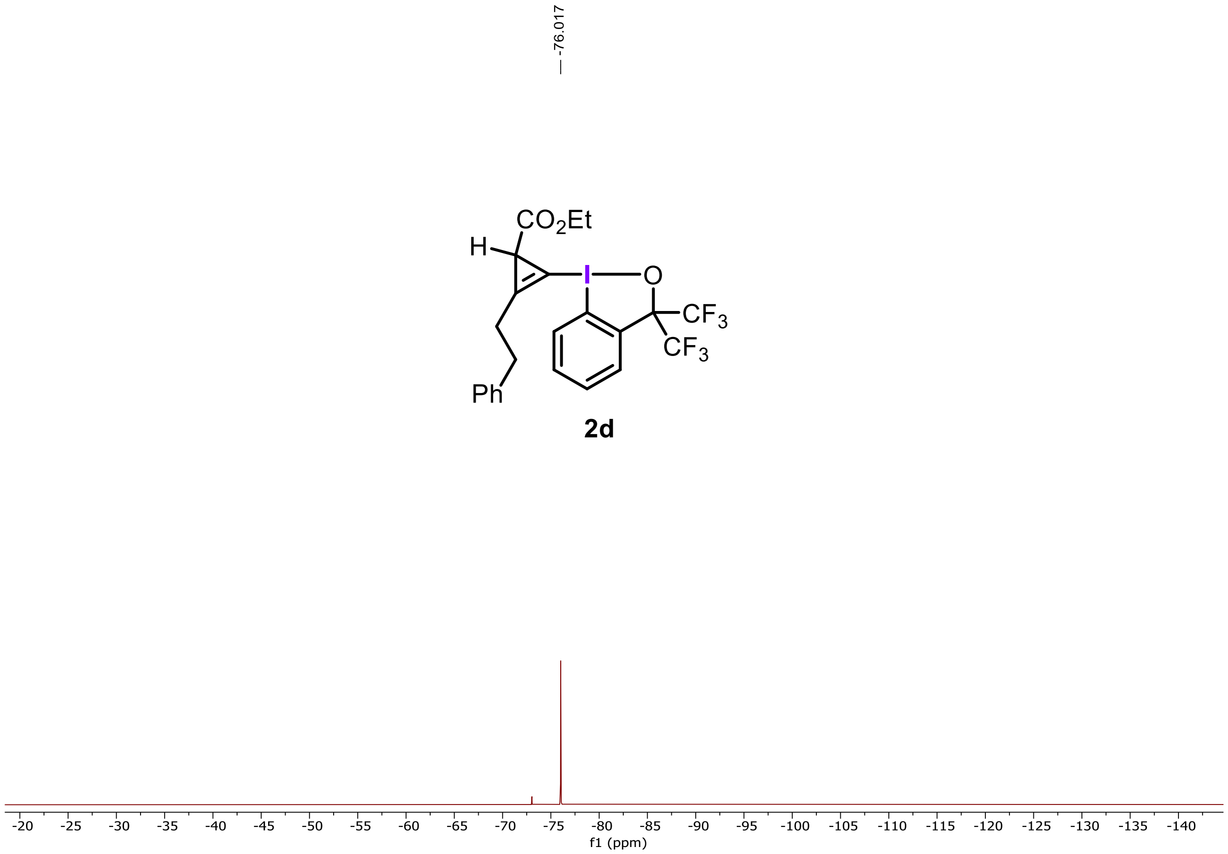

^{1}H NMR (400 MHz, $CDCl_3$) of 2e

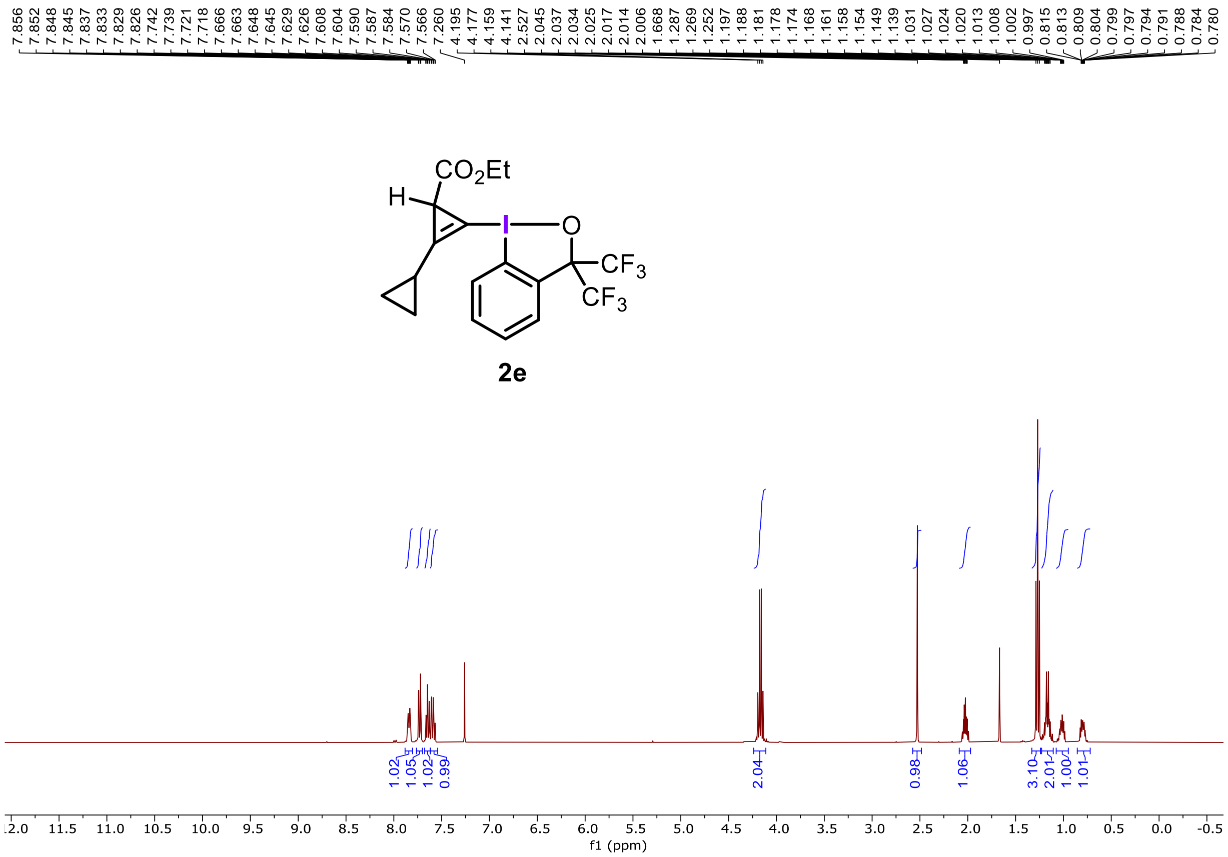

^{13}C NMR (101 MHz, $CDCl_3$) of 2e

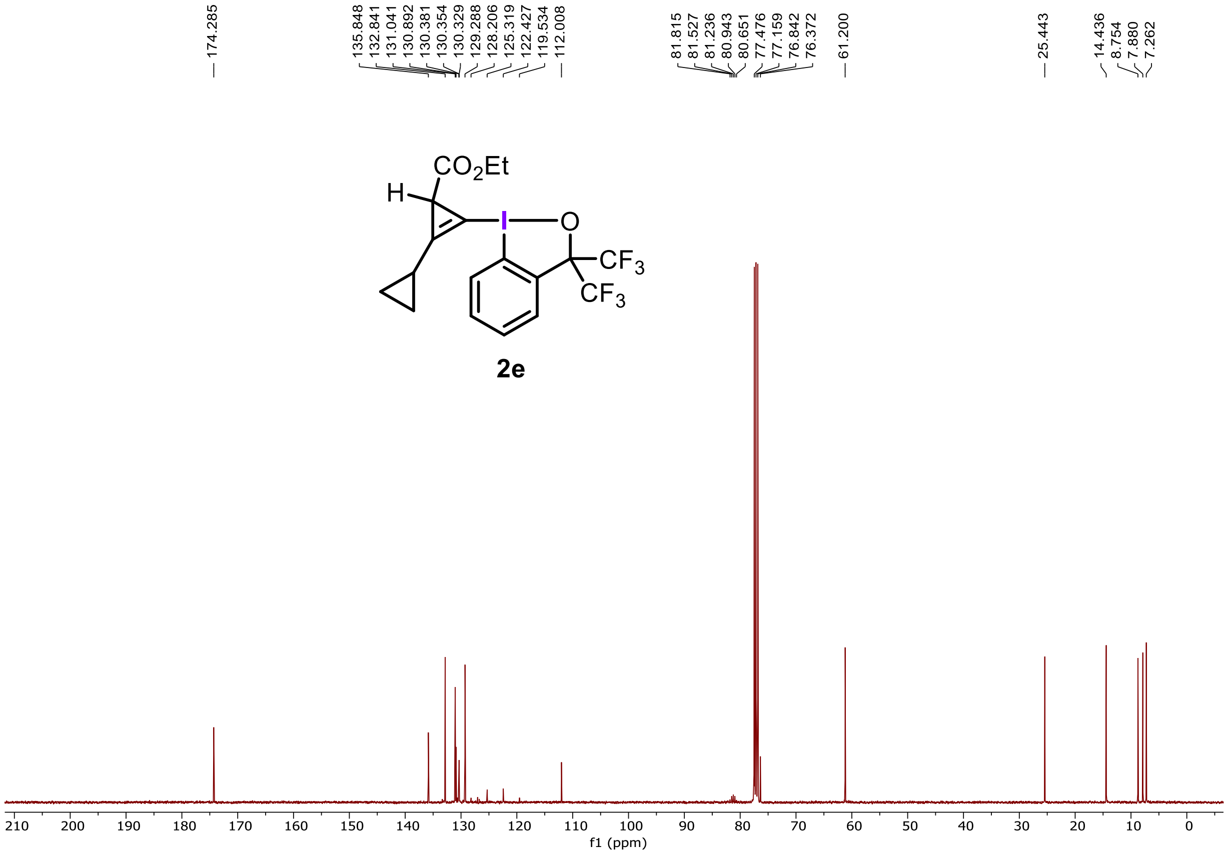

^{19}F NMR (377 MHz, $CDCl_3$) of 2e

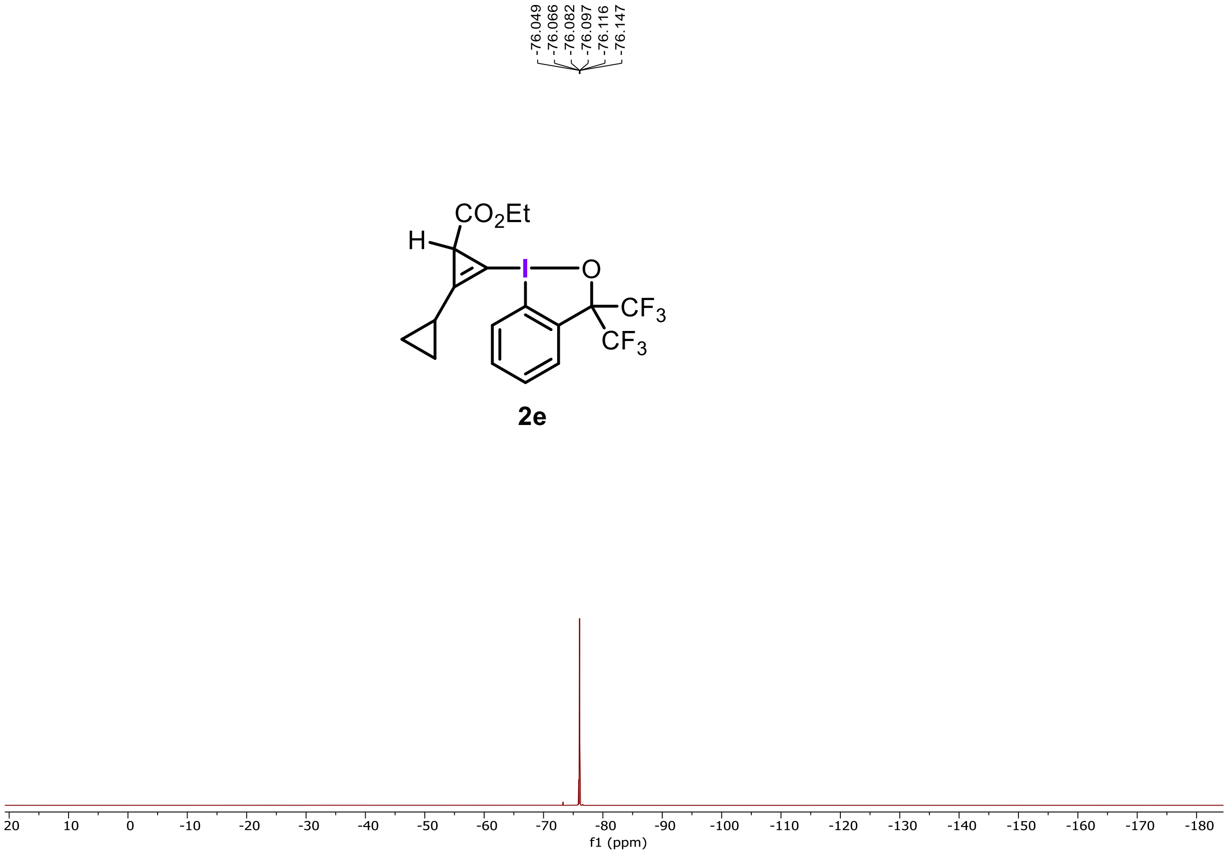

^{1}H NMR (400 MHz, $CDCl_3$) of 2f

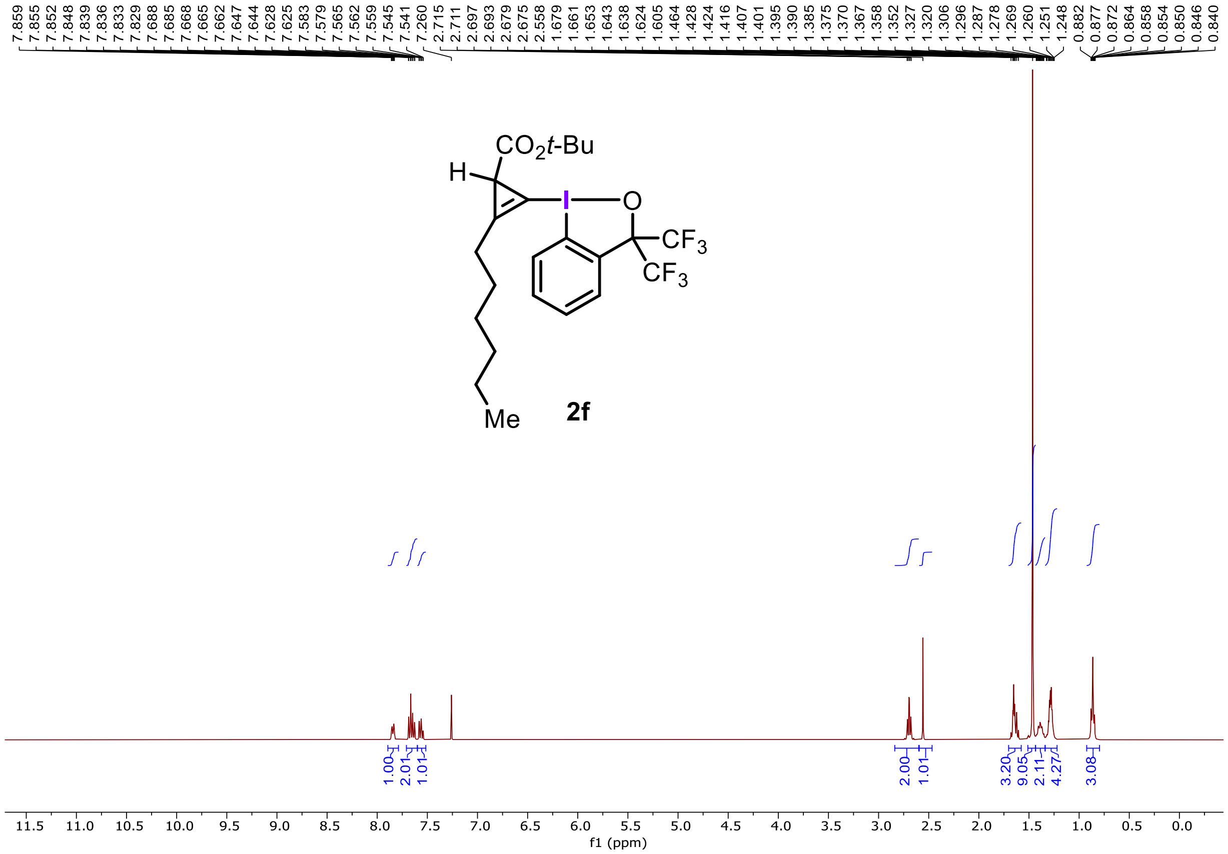

^{13}C NMR (101 MHz, $CDCl_3$) of 2f

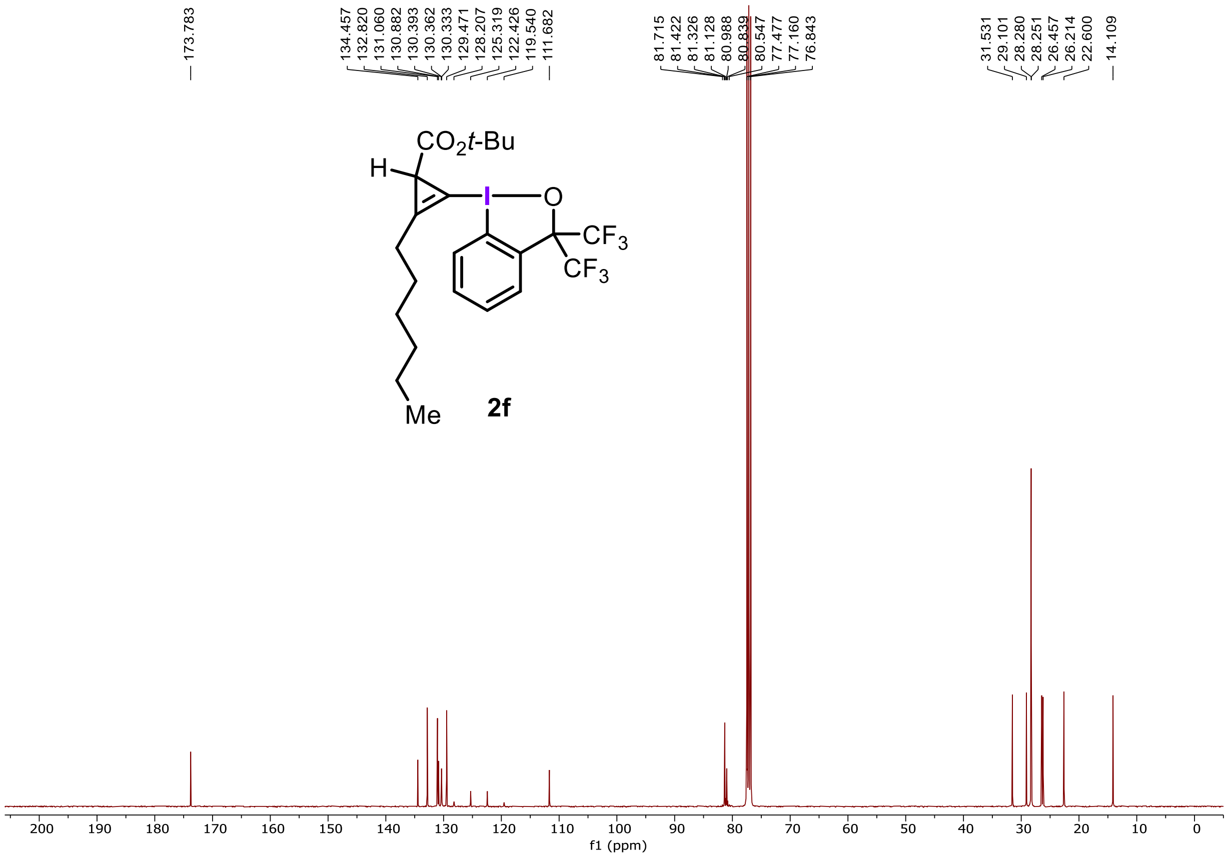

^{19}F NMR (377 MHz, $CDCl_3$) of 2f

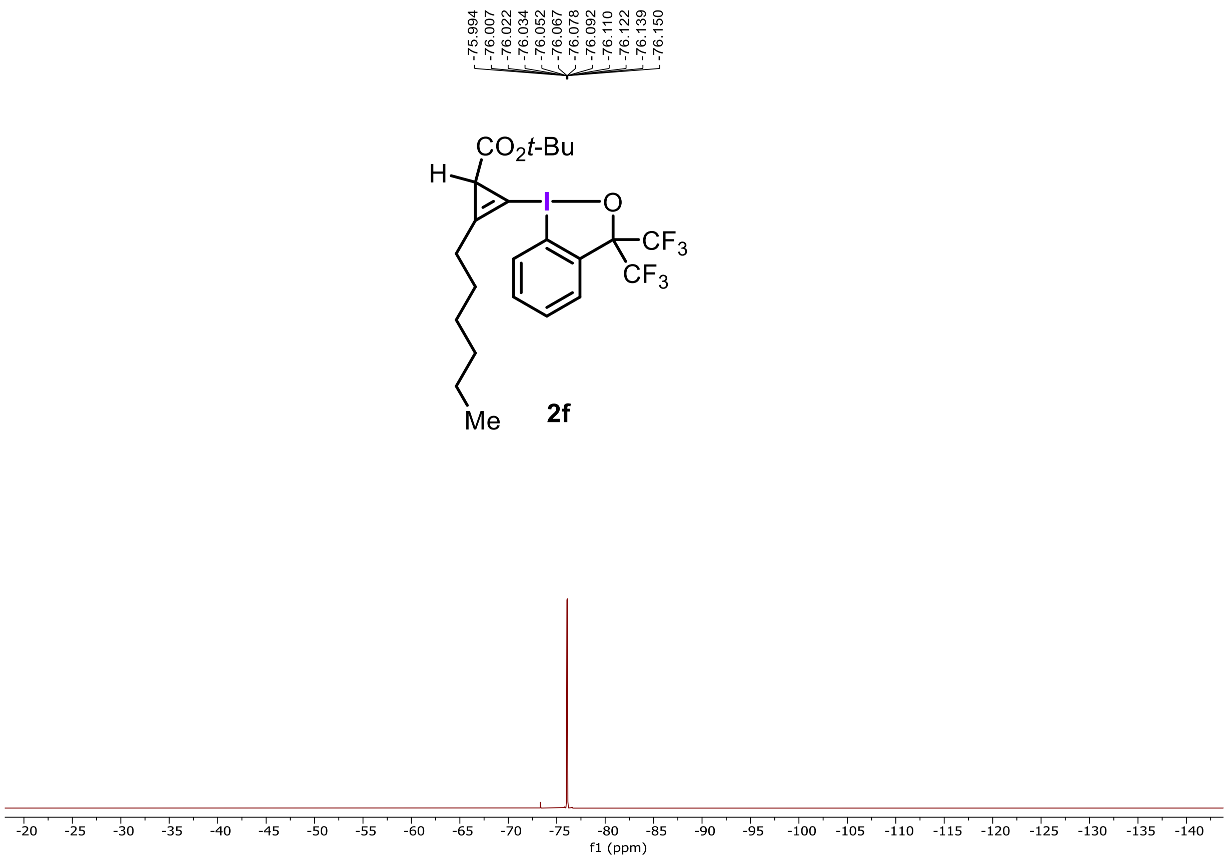

^{1}H NMR (400 MHz, $CDCl_3$) of 2g

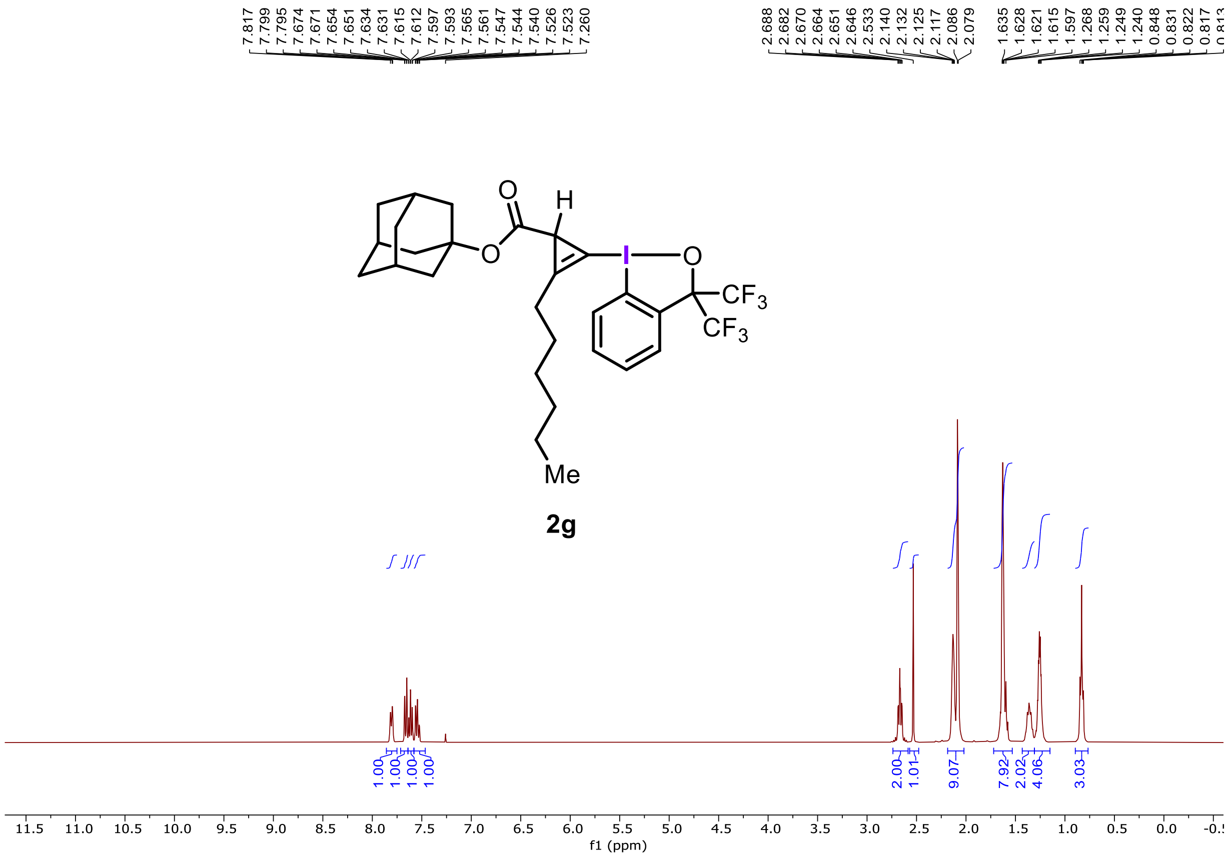

^{13}C NMR (101 MHz, $CDCl_3$) of 2g

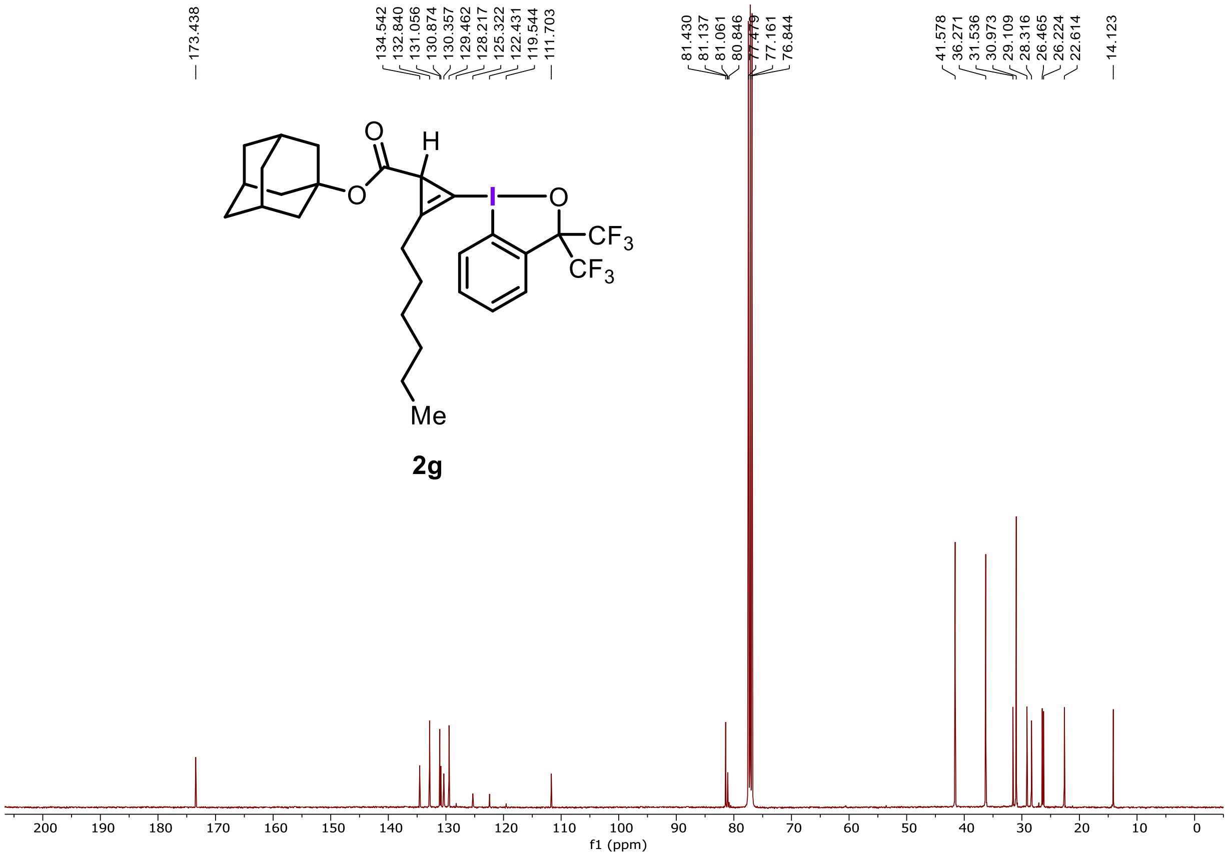

^{19}F NMR (377 MHz, $CDCl_3$) of 2g

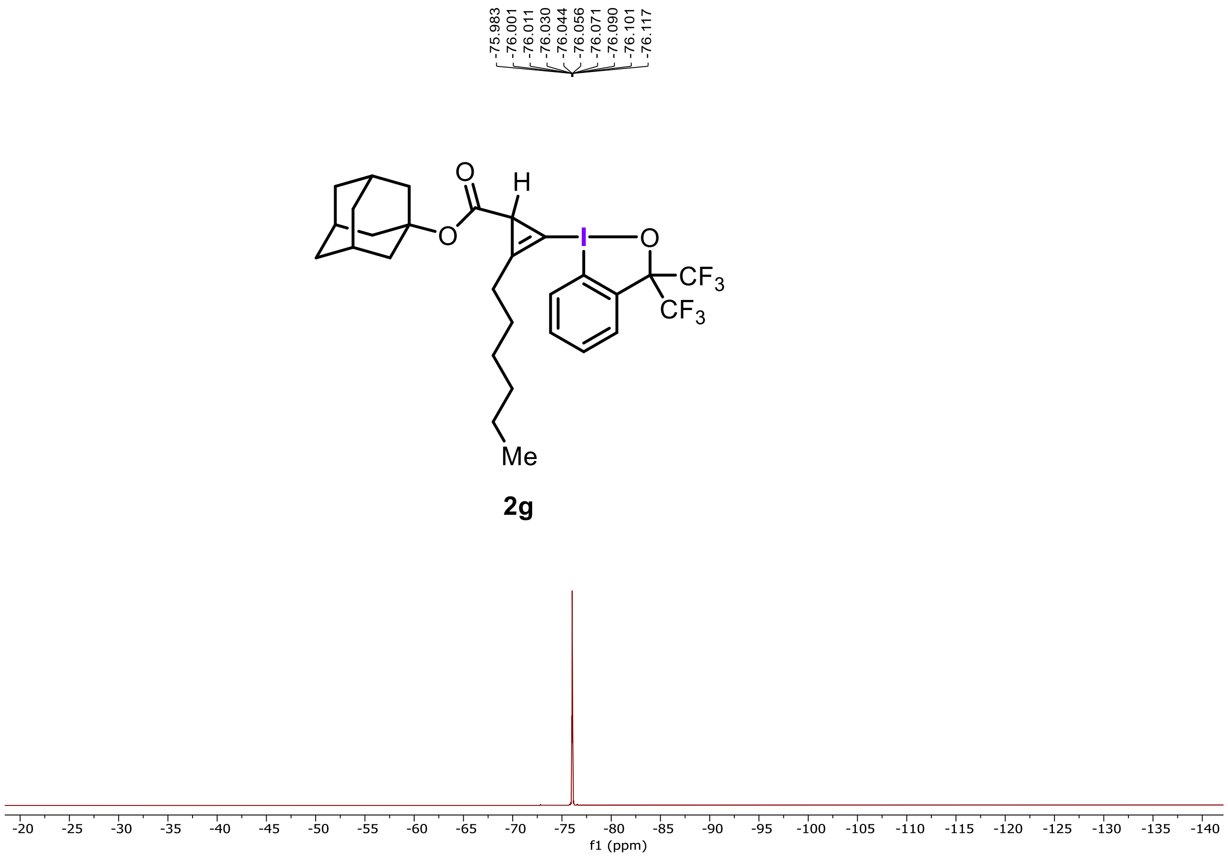

^{1}H NMR (400 MHz, $CDCl_3$) of 2h

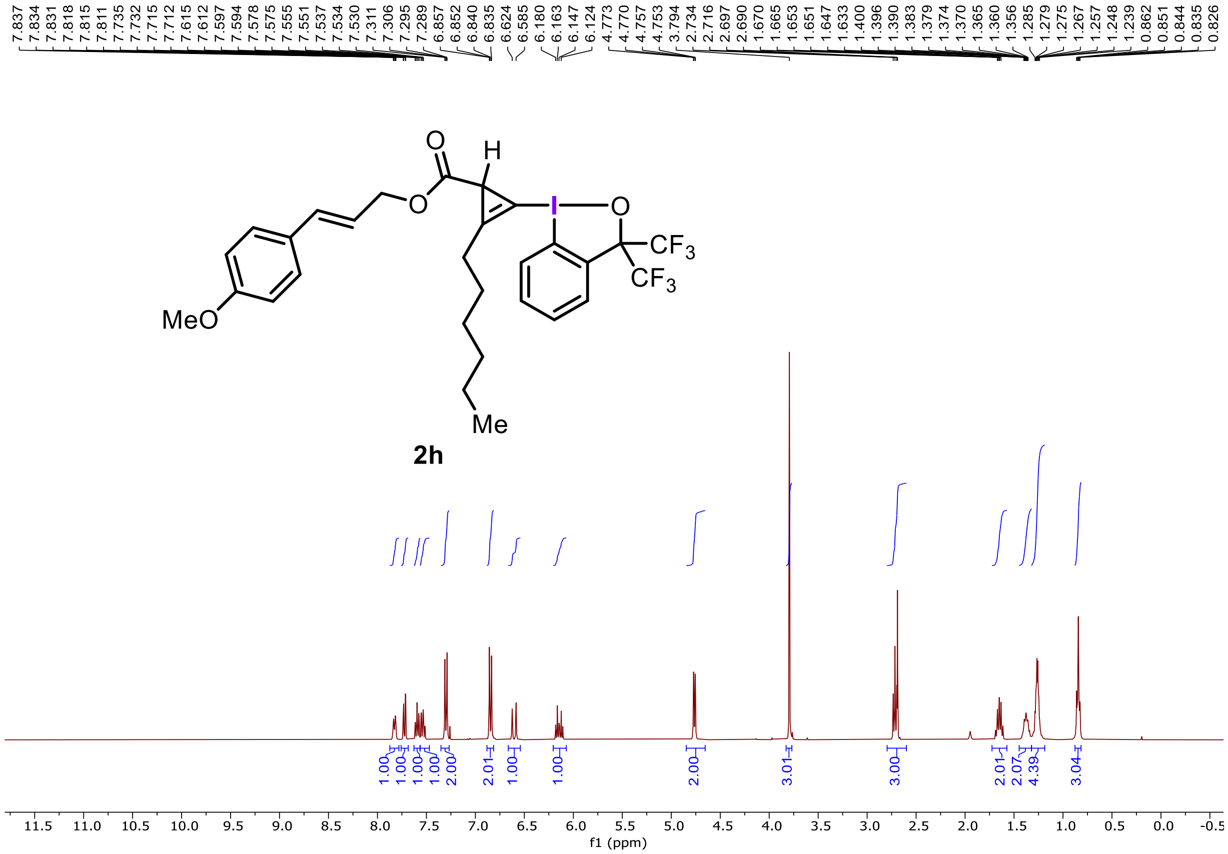

^{13}C NMR (101 MHz, $CDCl_3$) of 2h

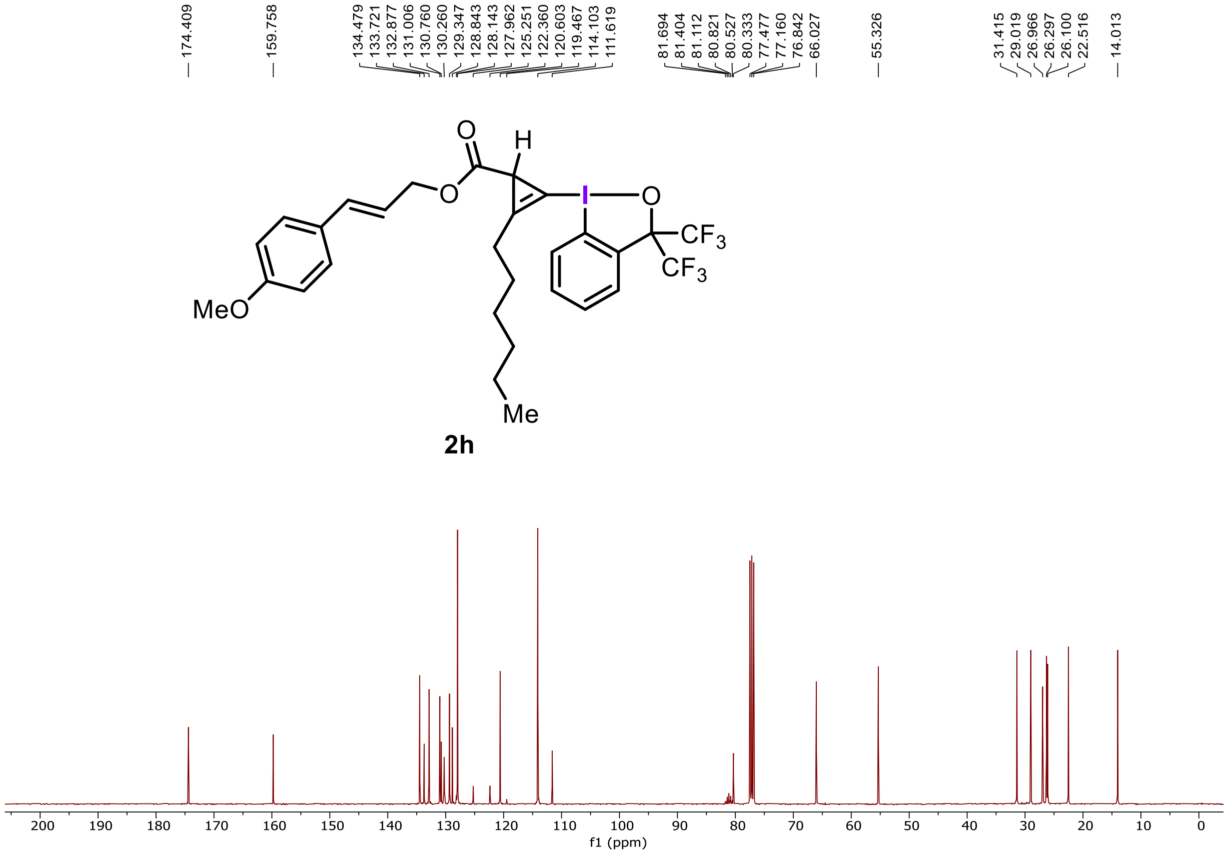

^{19}F NMR (377 MHz, $CDCl_3$) of 2h

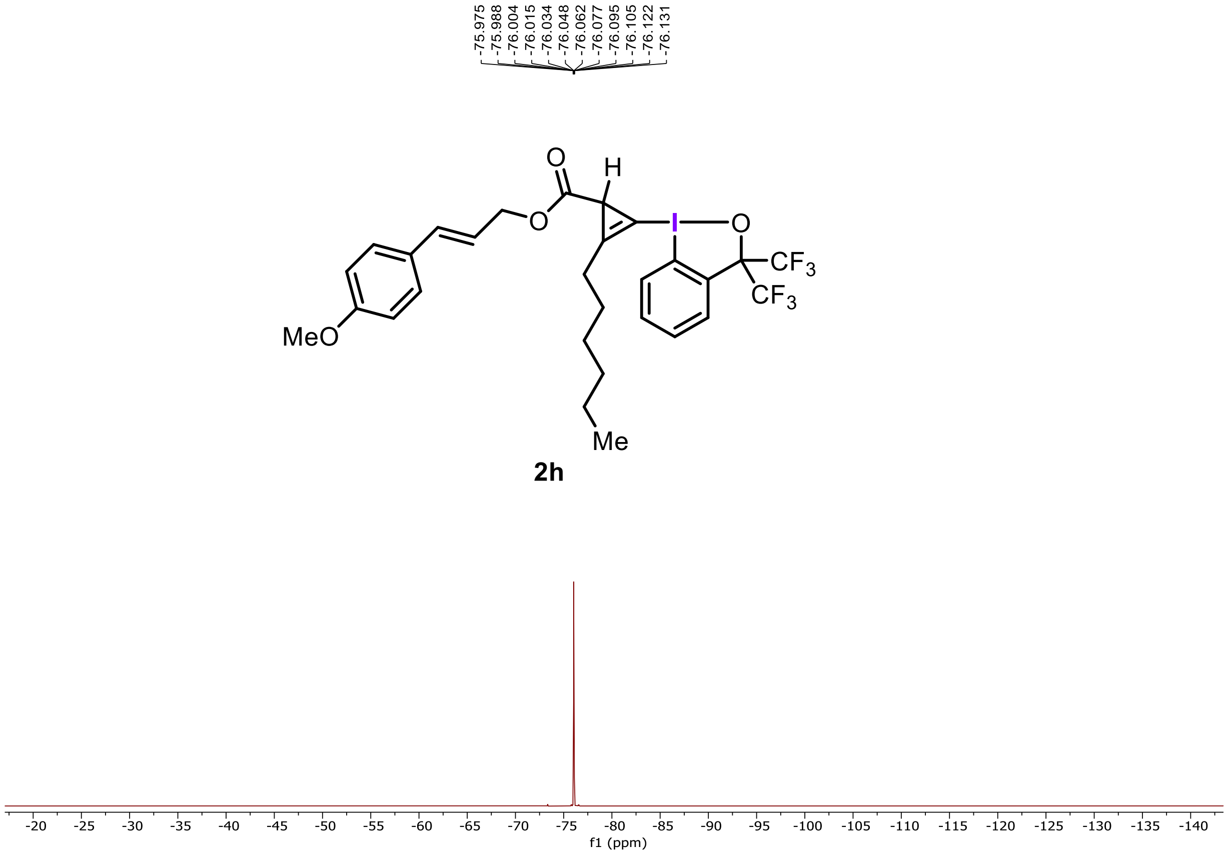

^{1}H NMR (400 MHz, $CDCl_3$) of 2i

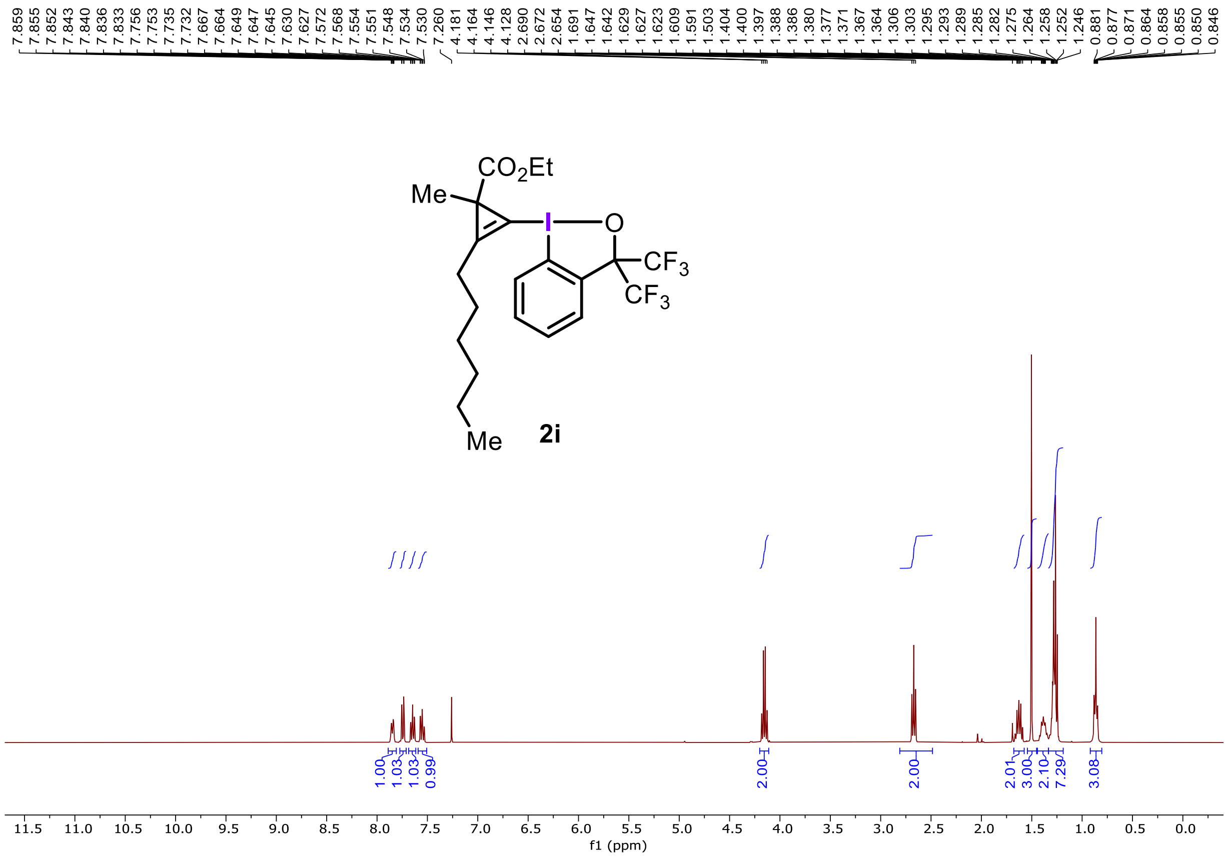

^{13}C NMR (101 MHz, $CDCl_3$) of 2i

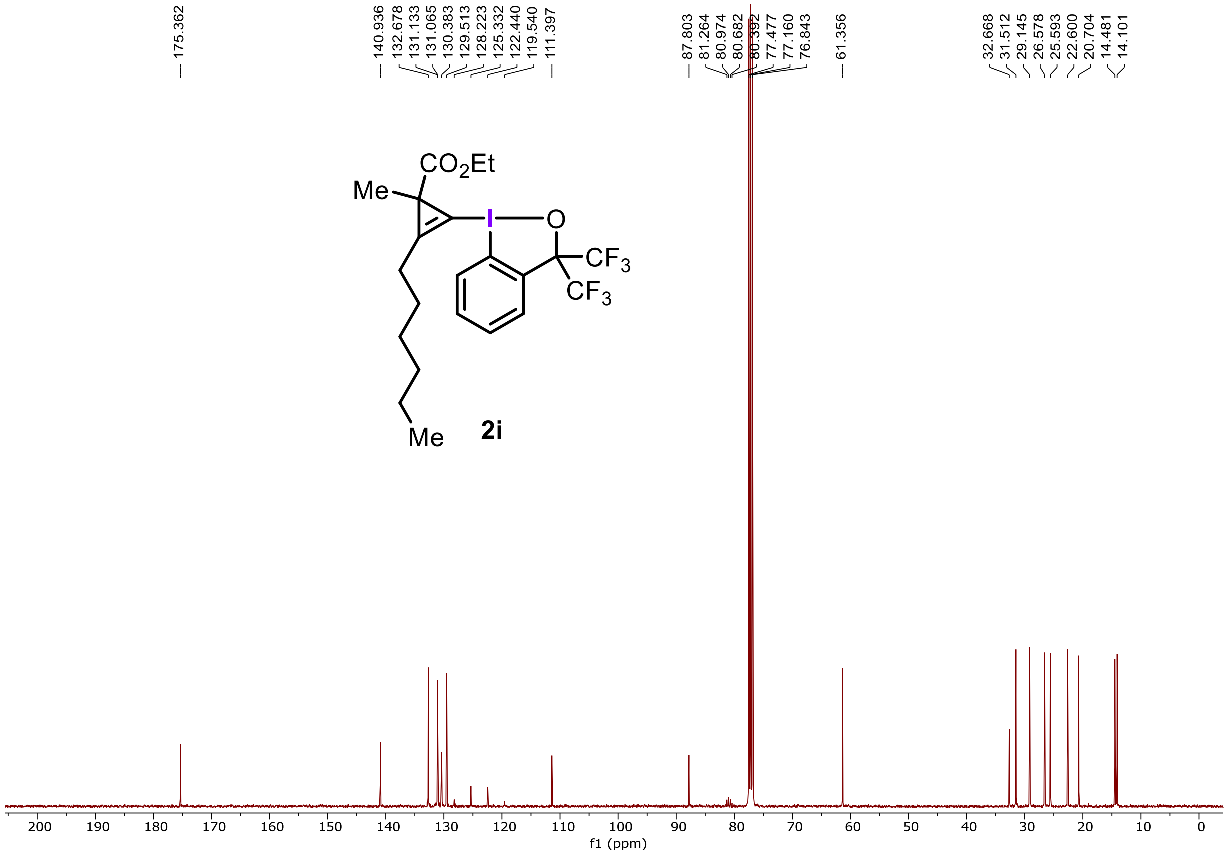

^{19}F NMR (377 MHz, $CDCl_3$) of 2i

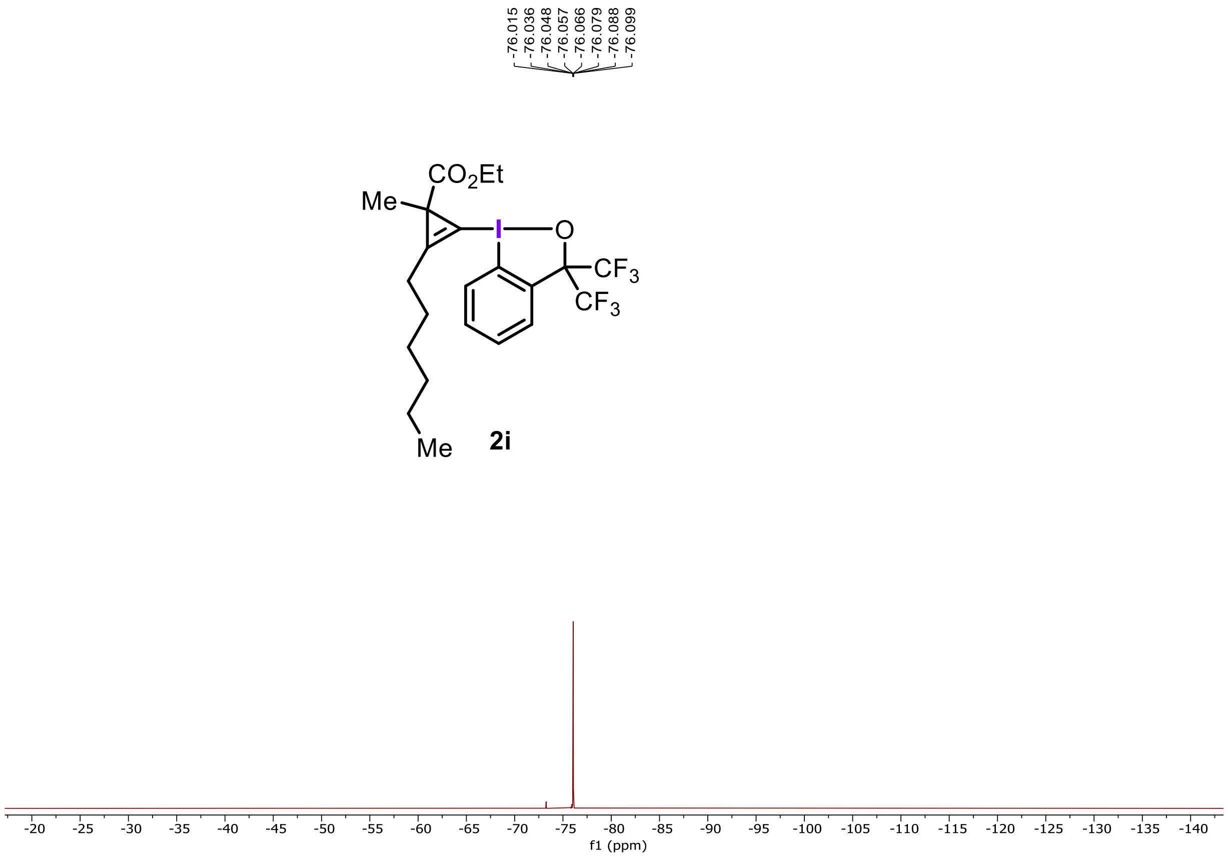

^{1}H NMR (400 MHz, $CDCl_3$) of 2j

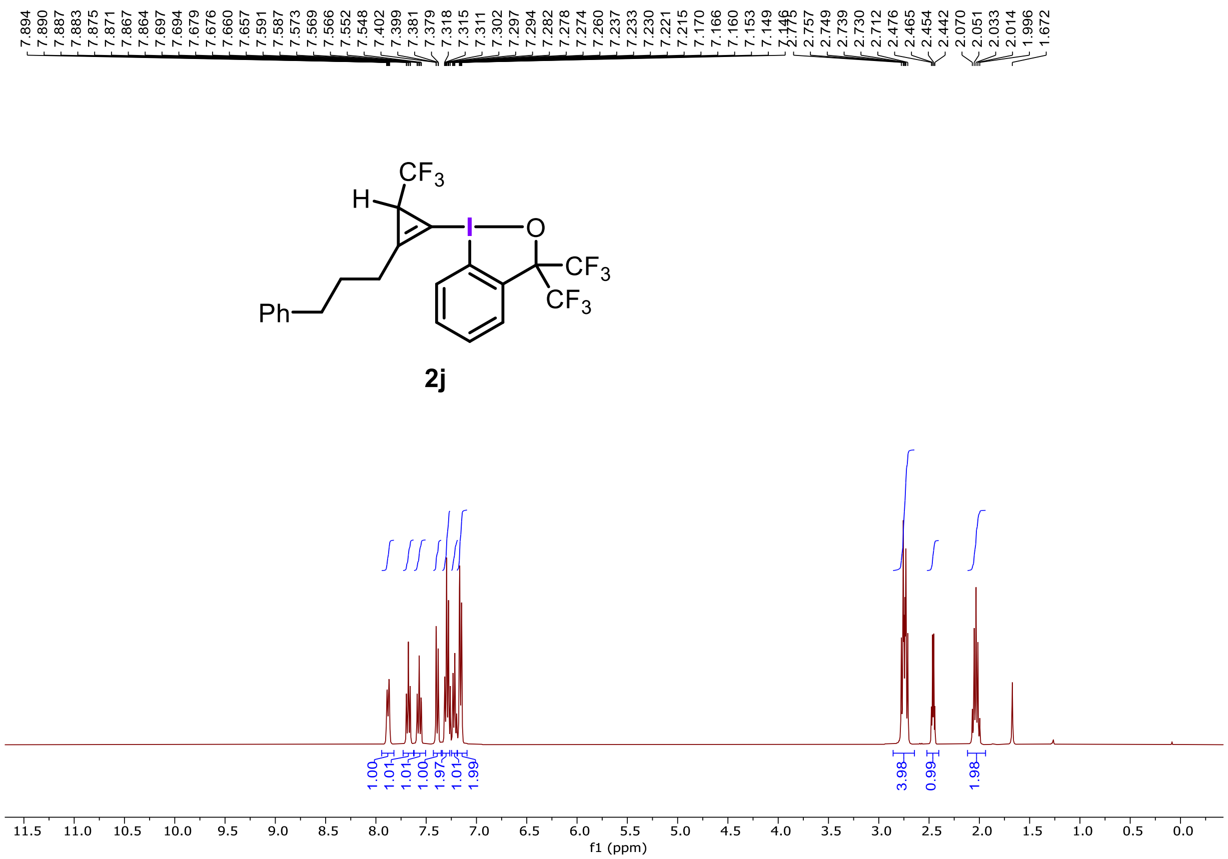

^{13}C NMR (101 MHz, $CDCl_3$) of 2j

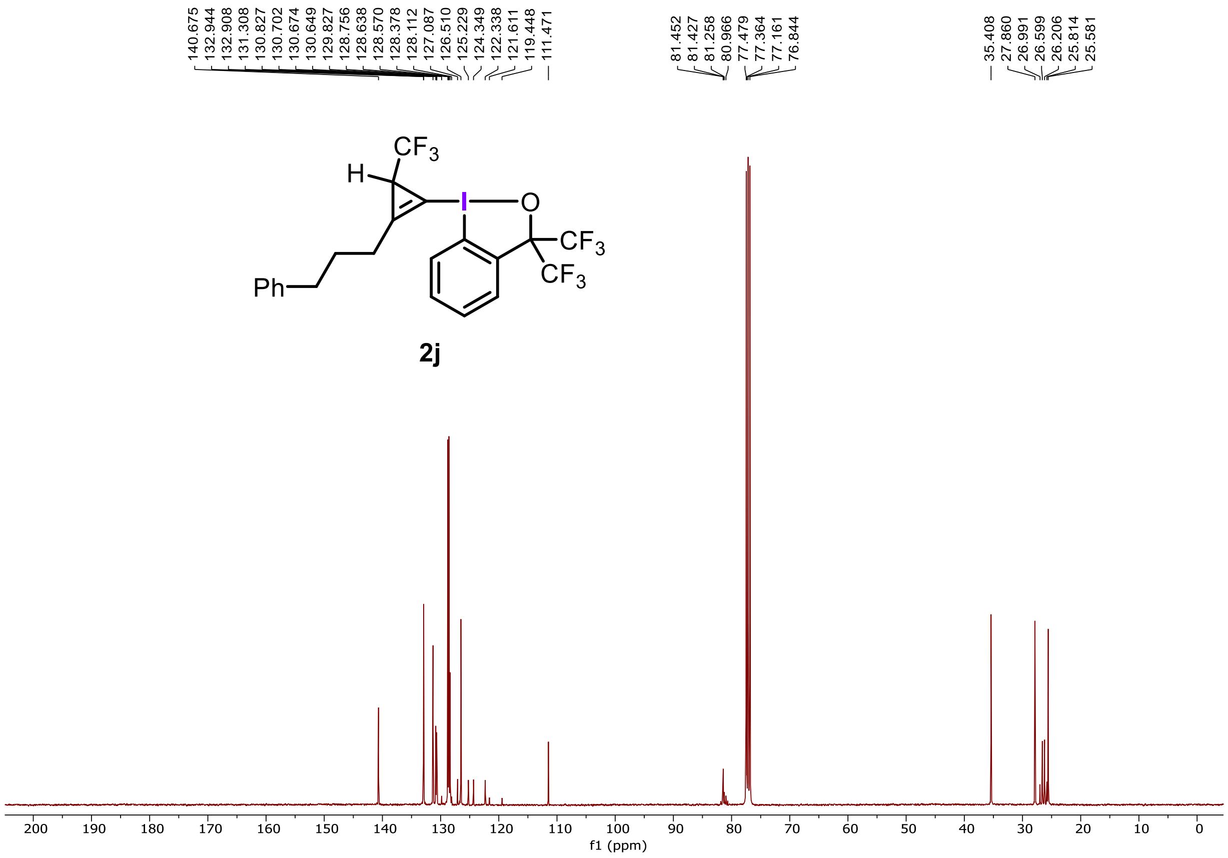

^{19}F NMR (377 MHz, $CDCl_3$) of 2j

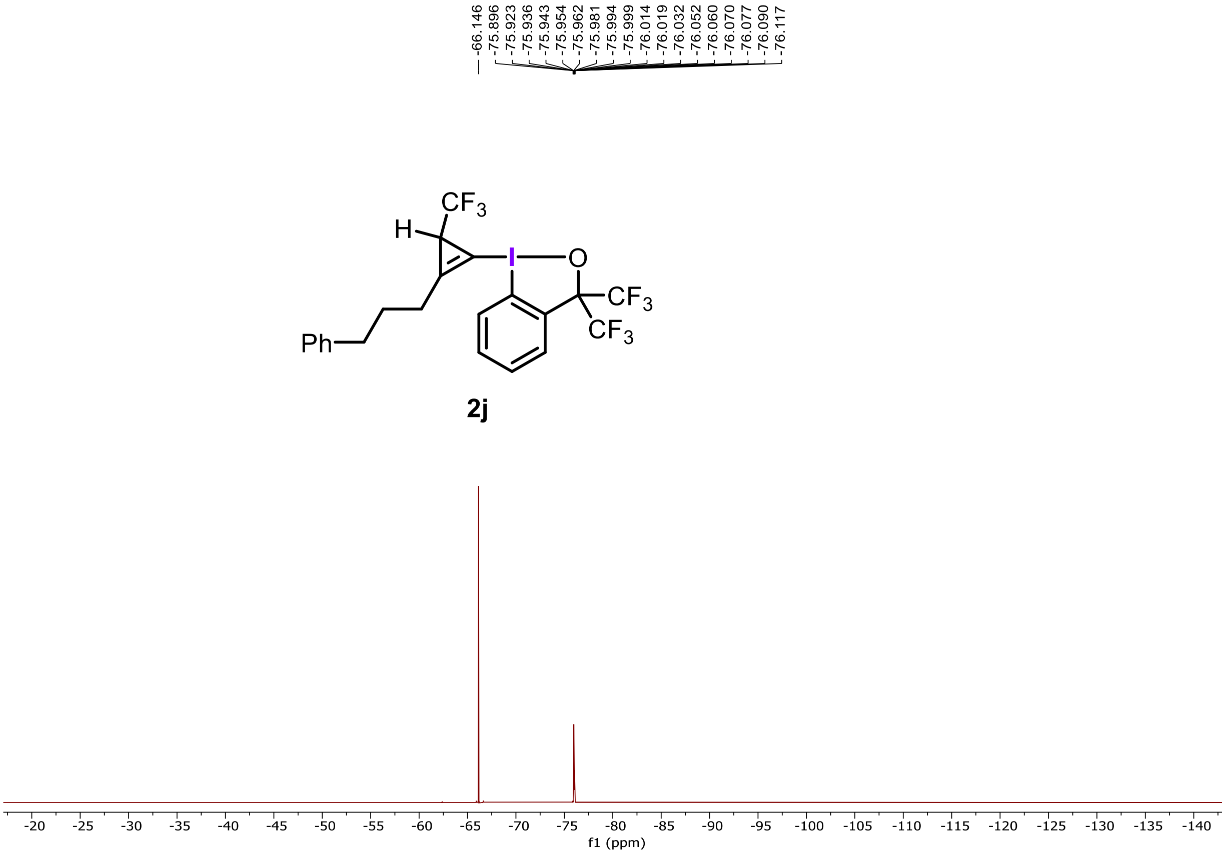

^{1}H NMR (400 MHz, $CDCl_3$) of 2k

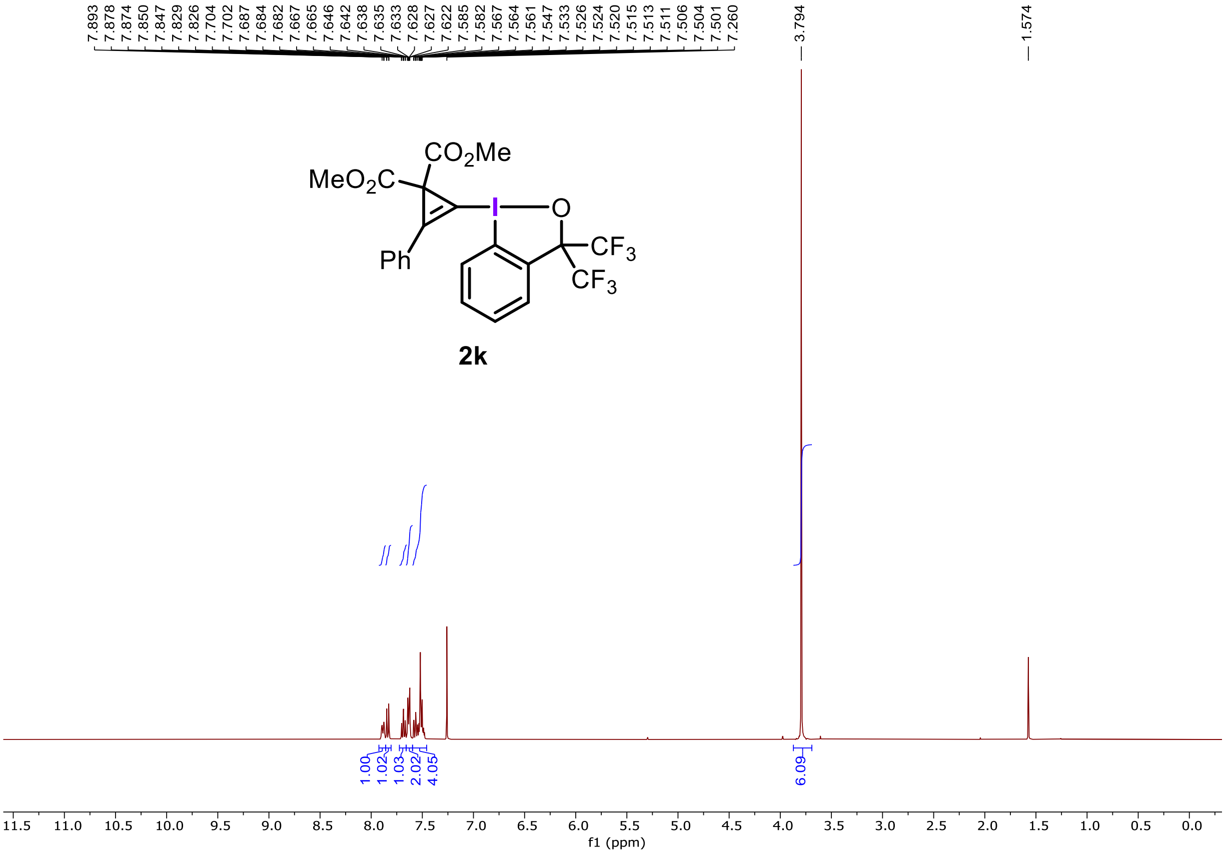

^{13}C NMR (101 MHz, $CDCl_3$) of 2k

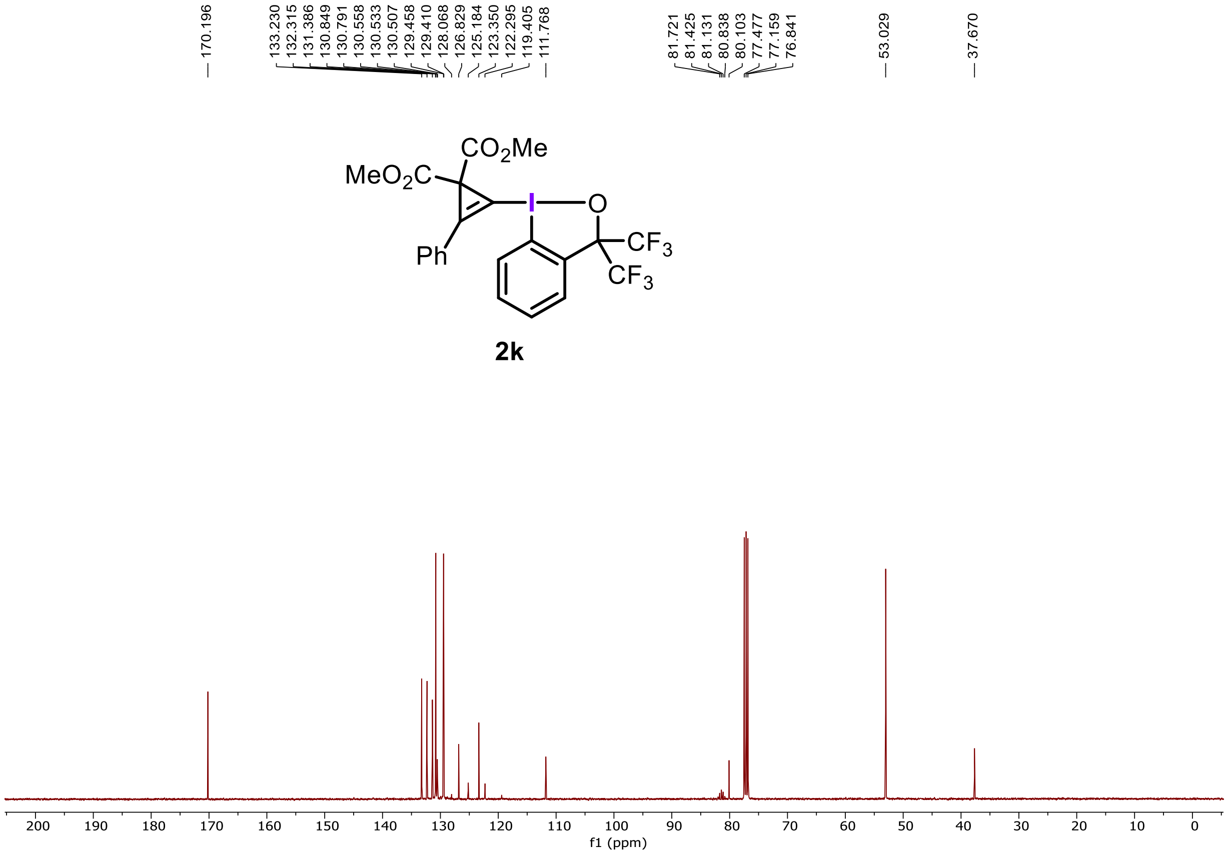

^{19}F NMR (377 MHz, $CDCl_3$) of 2k

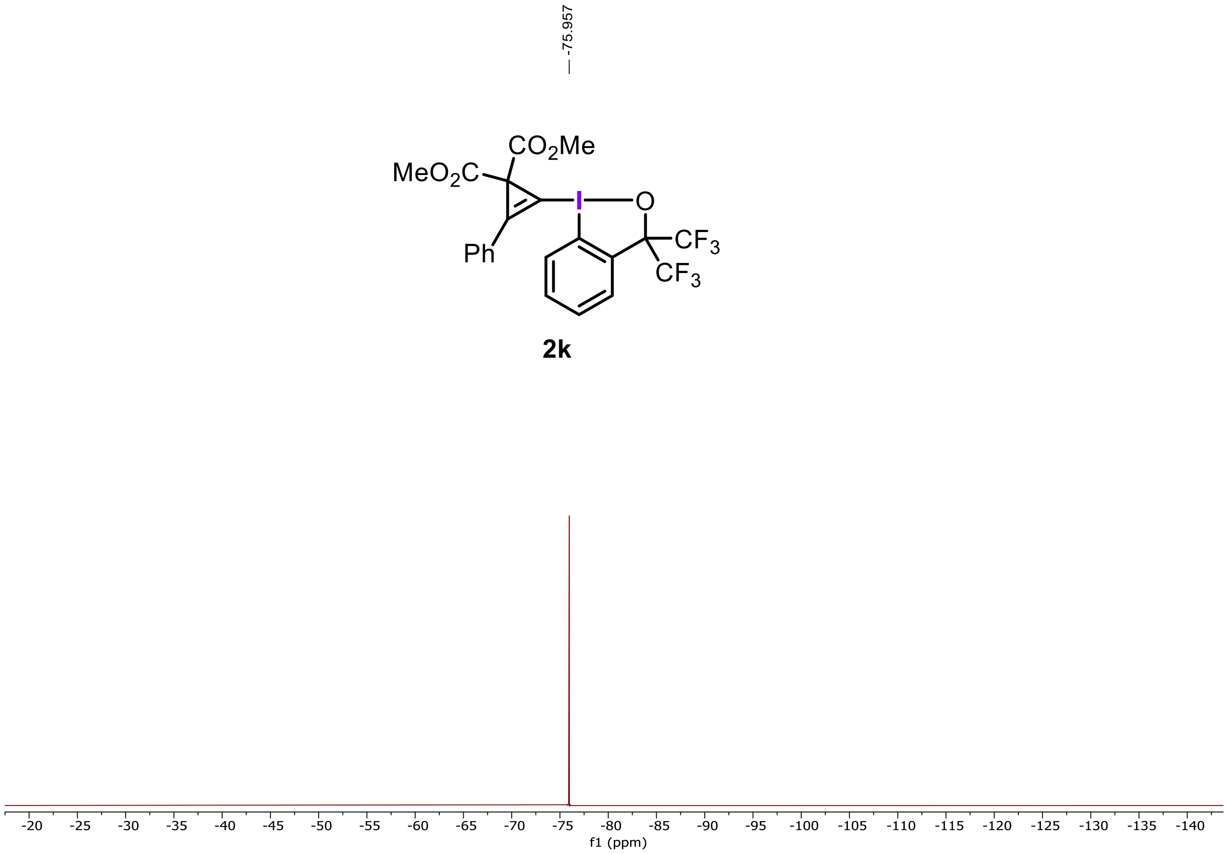

^{1}H NMR (400 MHz, $CDCl_3$) of 2l

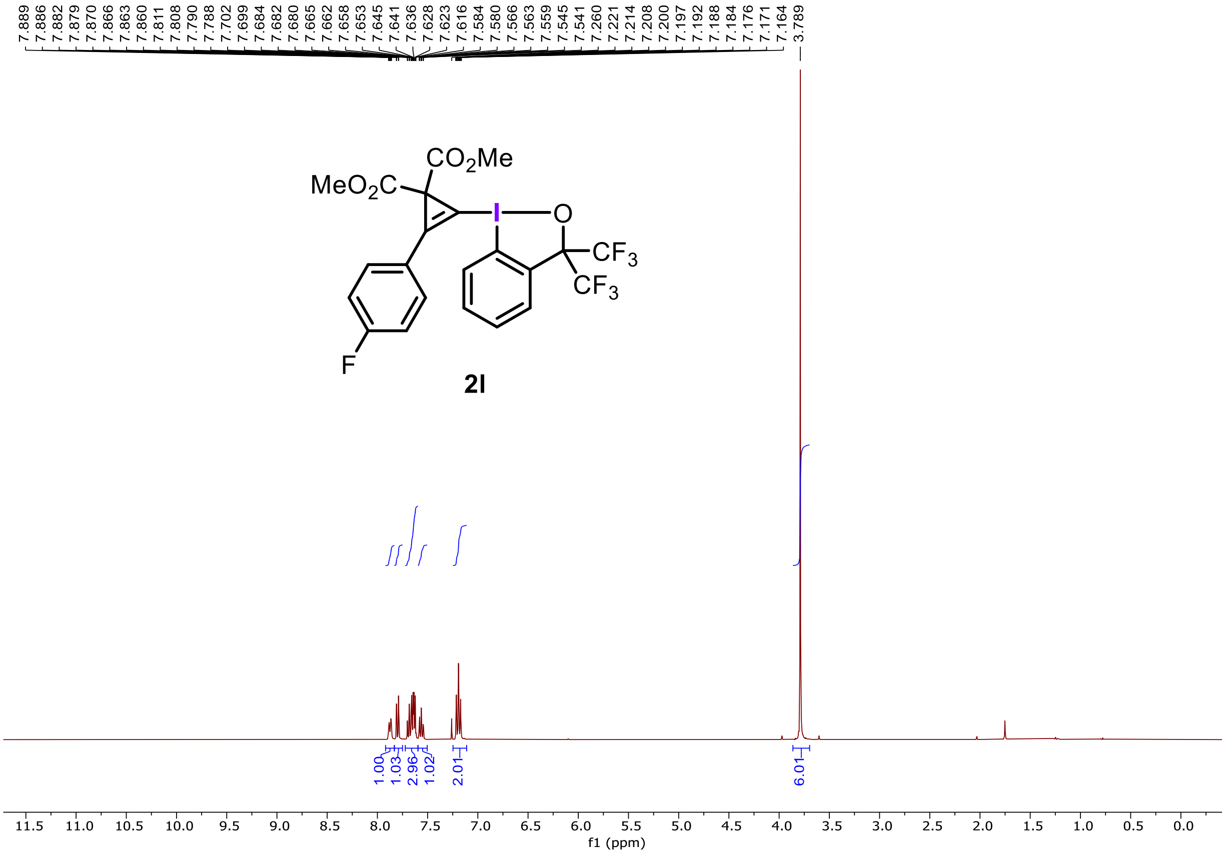

^{13}C NMR (101 MHz, $CDCl_3$) of 2l

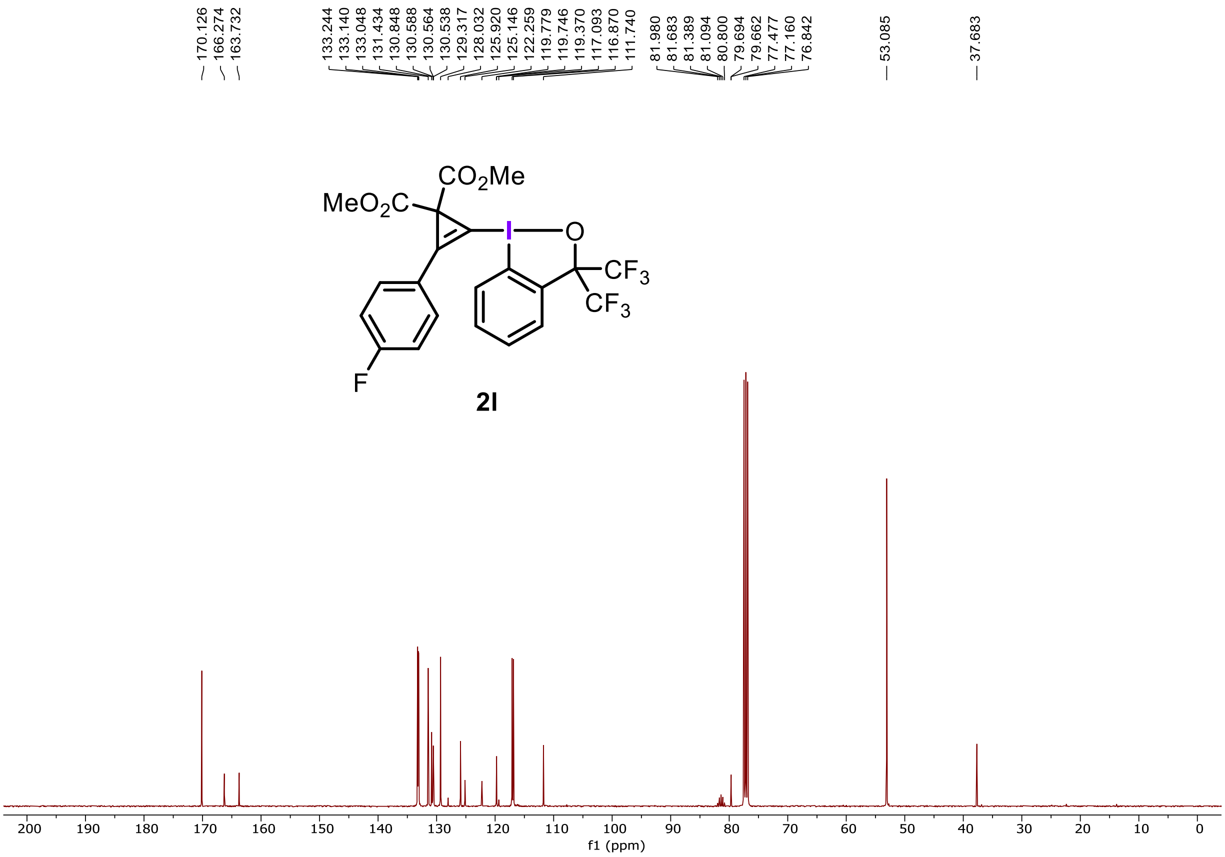

^{19}F NMR (377 MHz, $CDCl_3$) of 2l

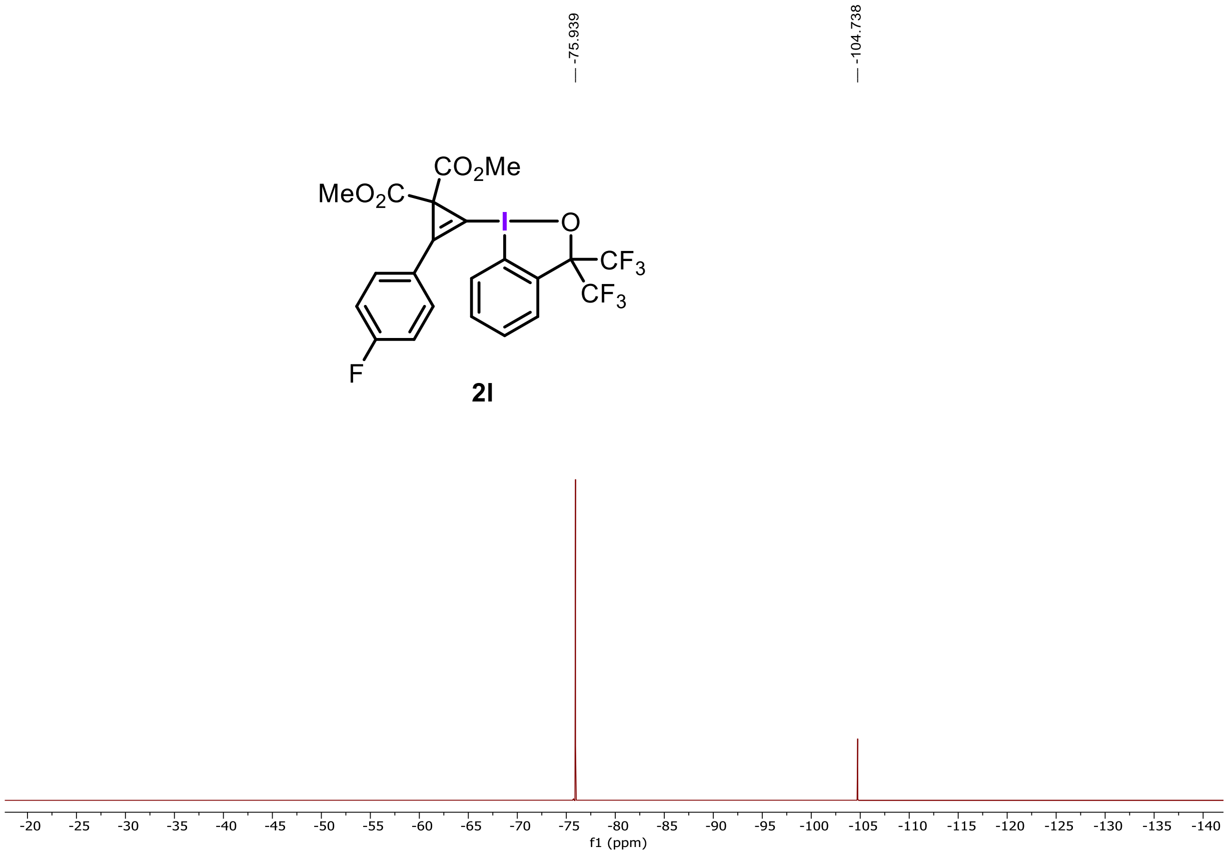

^{1}H NMR (400 MHz, $CDCl_3$) of 2m

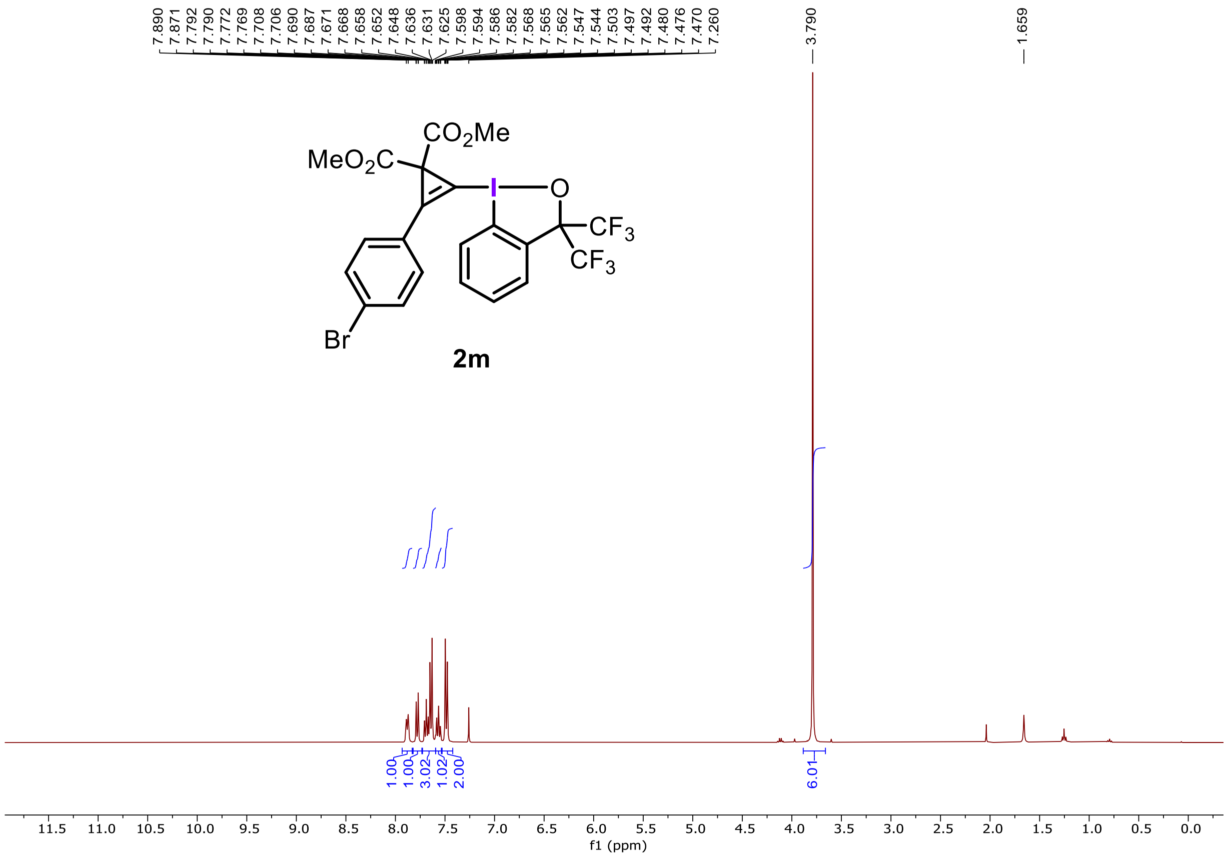

^{13}C NMR (101 MHz, $CDCl_3$) of 2m

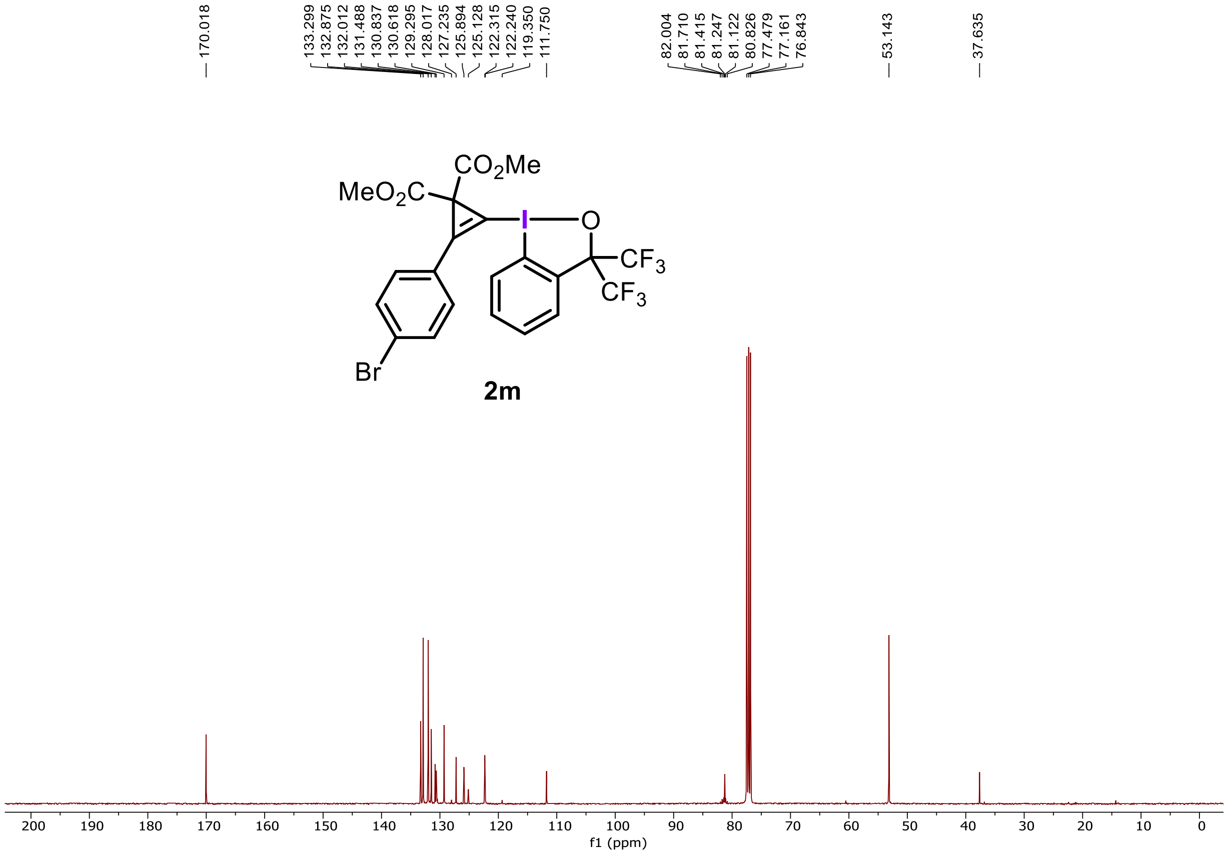

^{19}F NMR (377 MHz, $CDCl_3$) of 2m

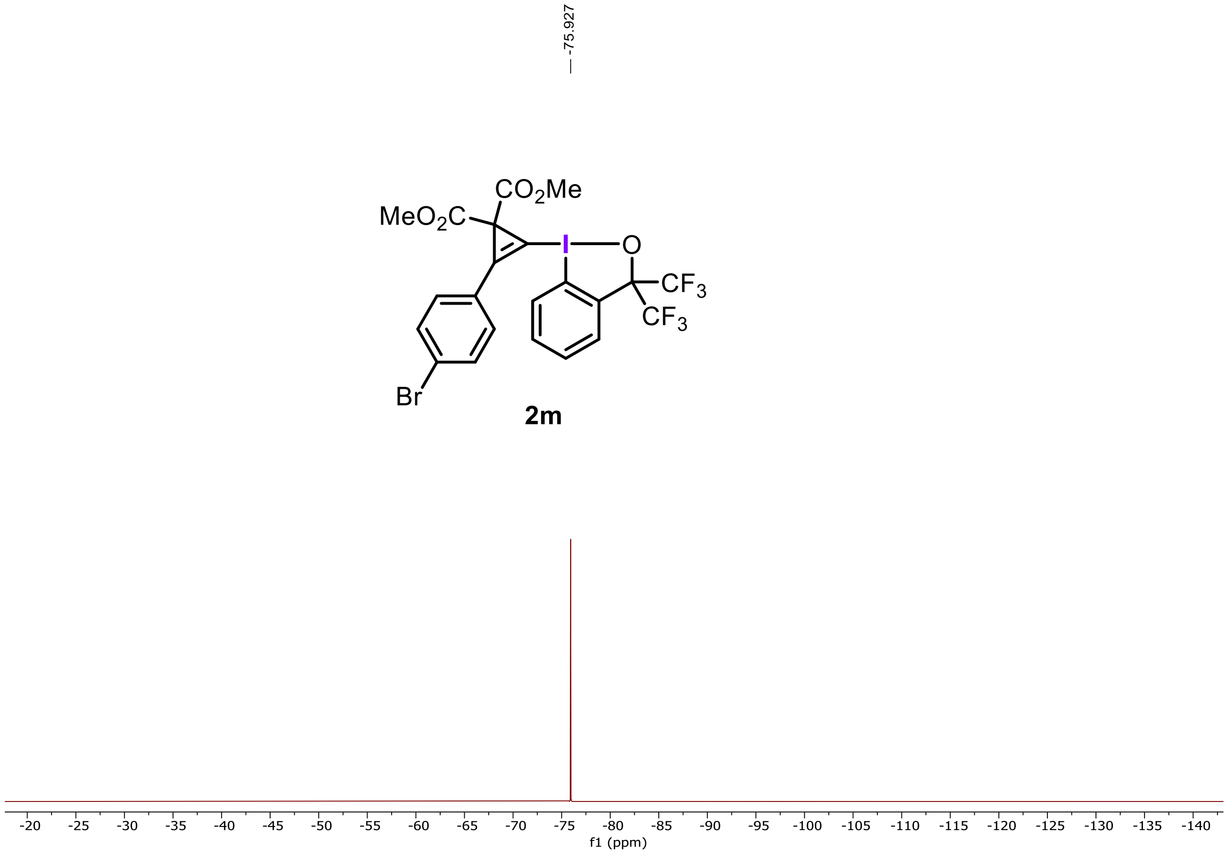

^{1}H NMR (400 MHz, $CDCl_3$) of 2n

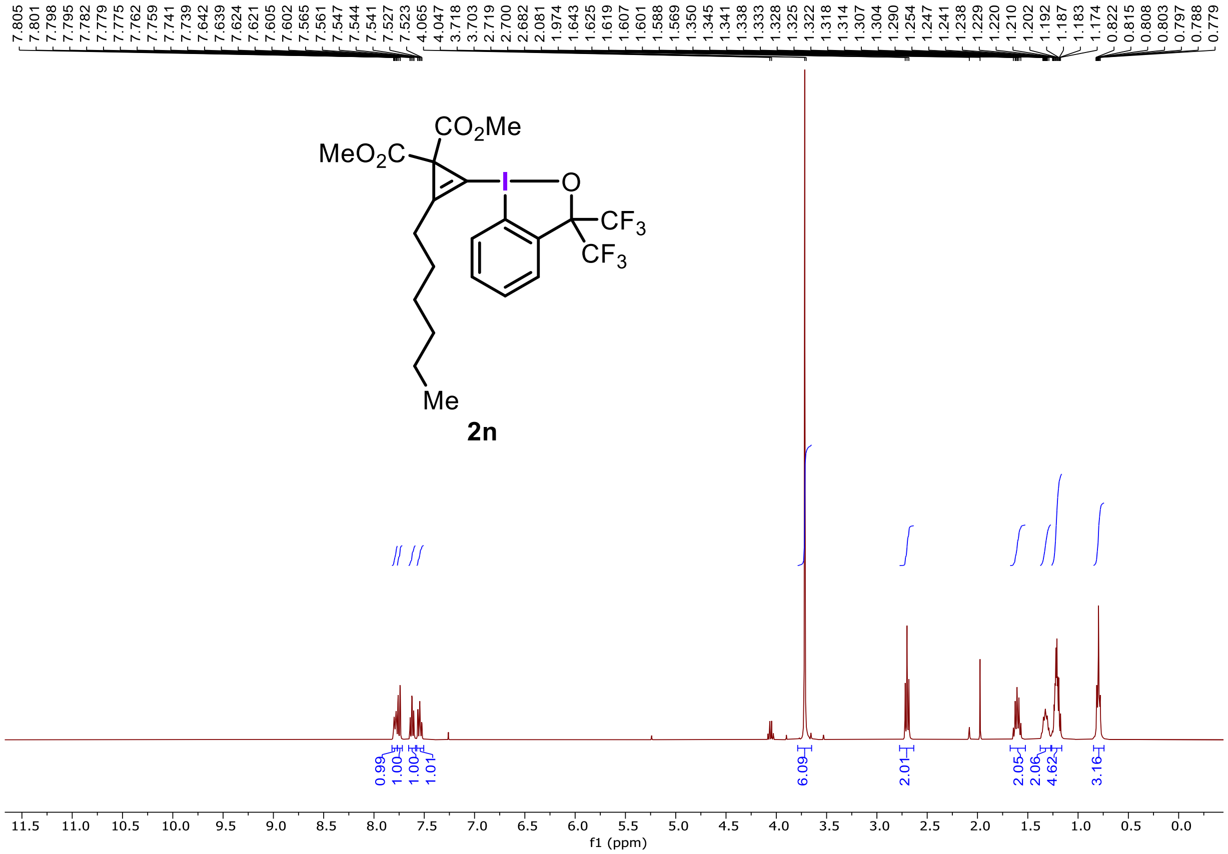

^{13}C NMR (101 MHz, $CDCl_3$) of 2n

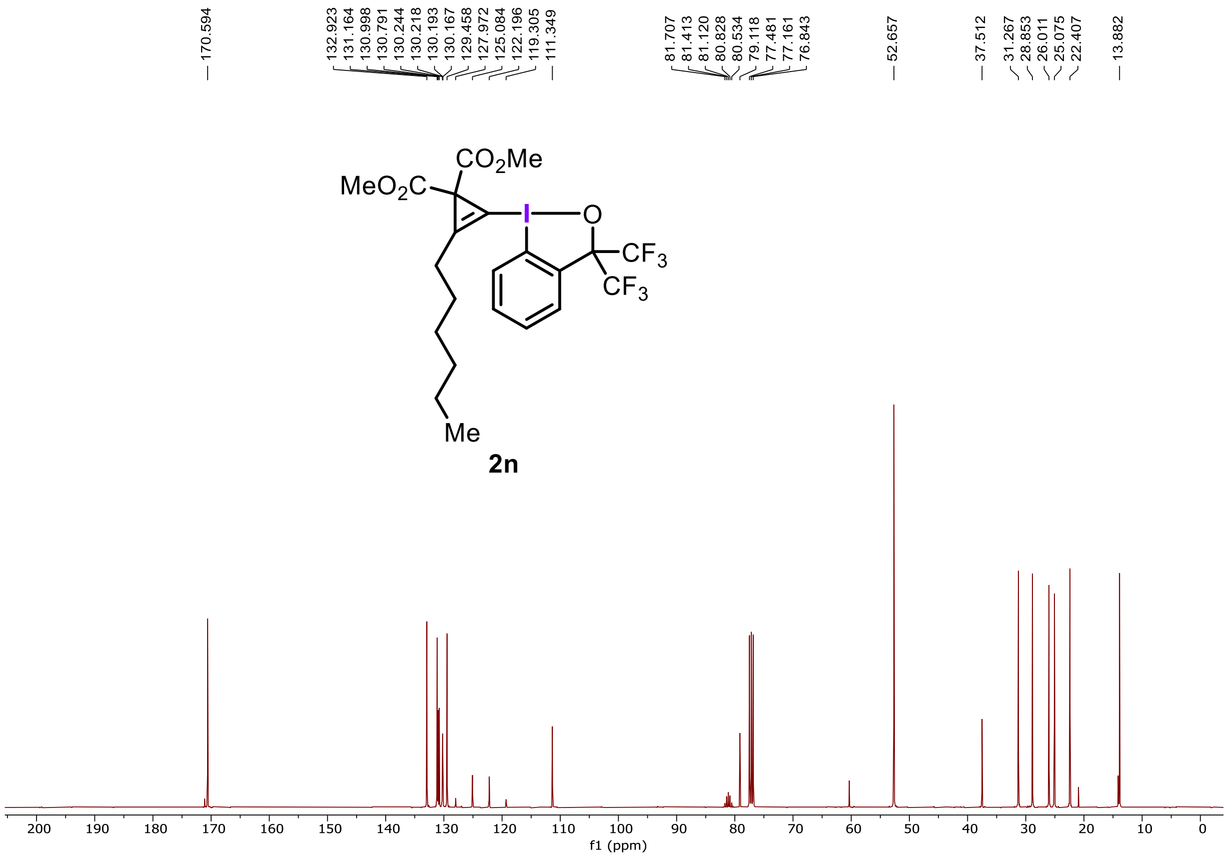

^{19}F NMR (377 MHz, $CDCl_3$) of 2n

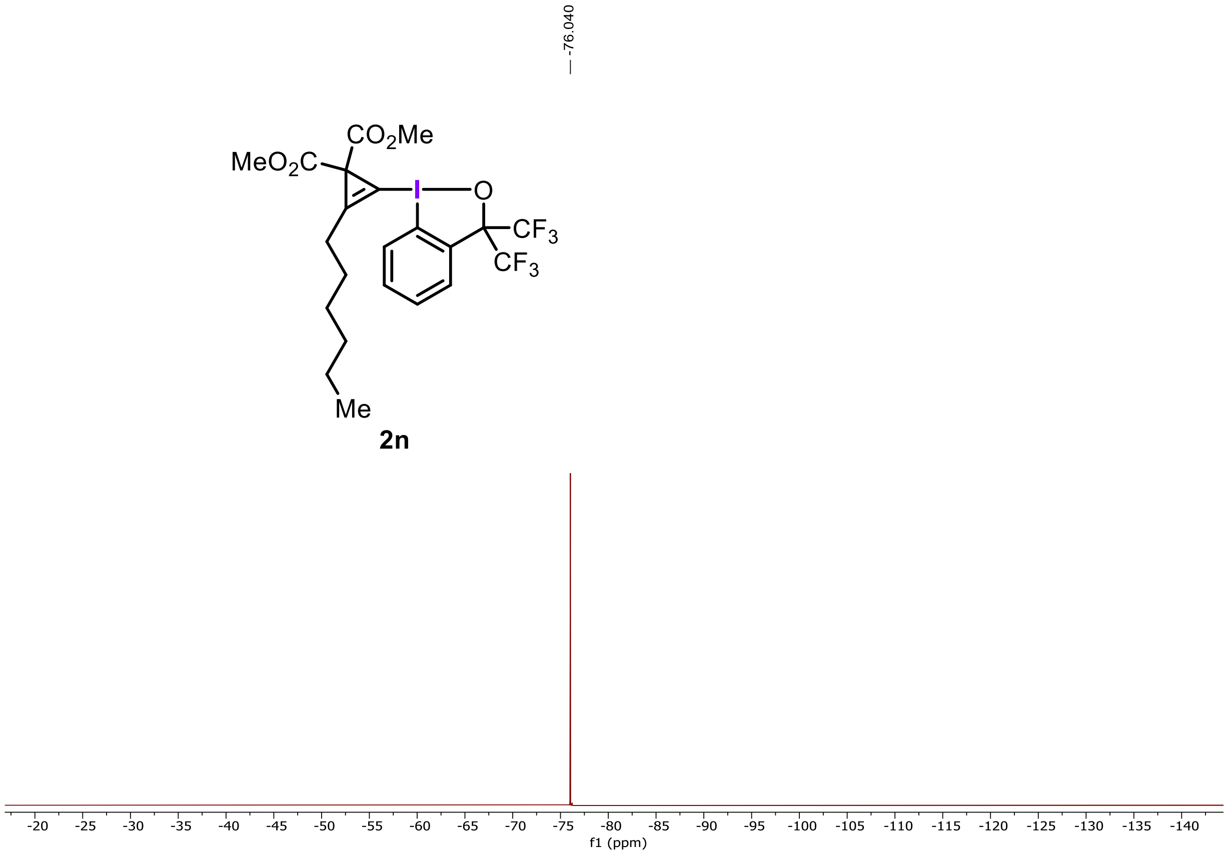

^{1}H NMR (400 MHz, $CDCl_3$) of 2o

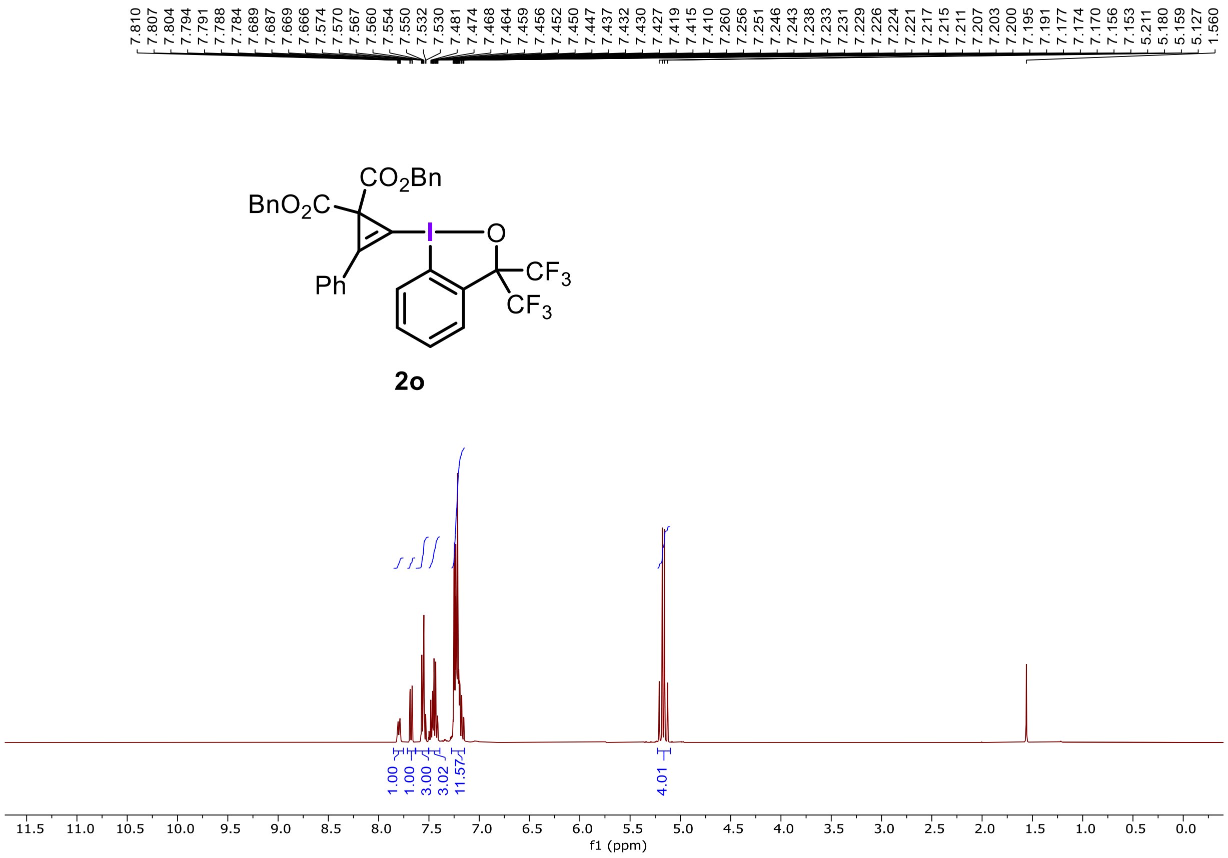

^{13}C NMR (101 MHz, $CDCl_3$) of 2o

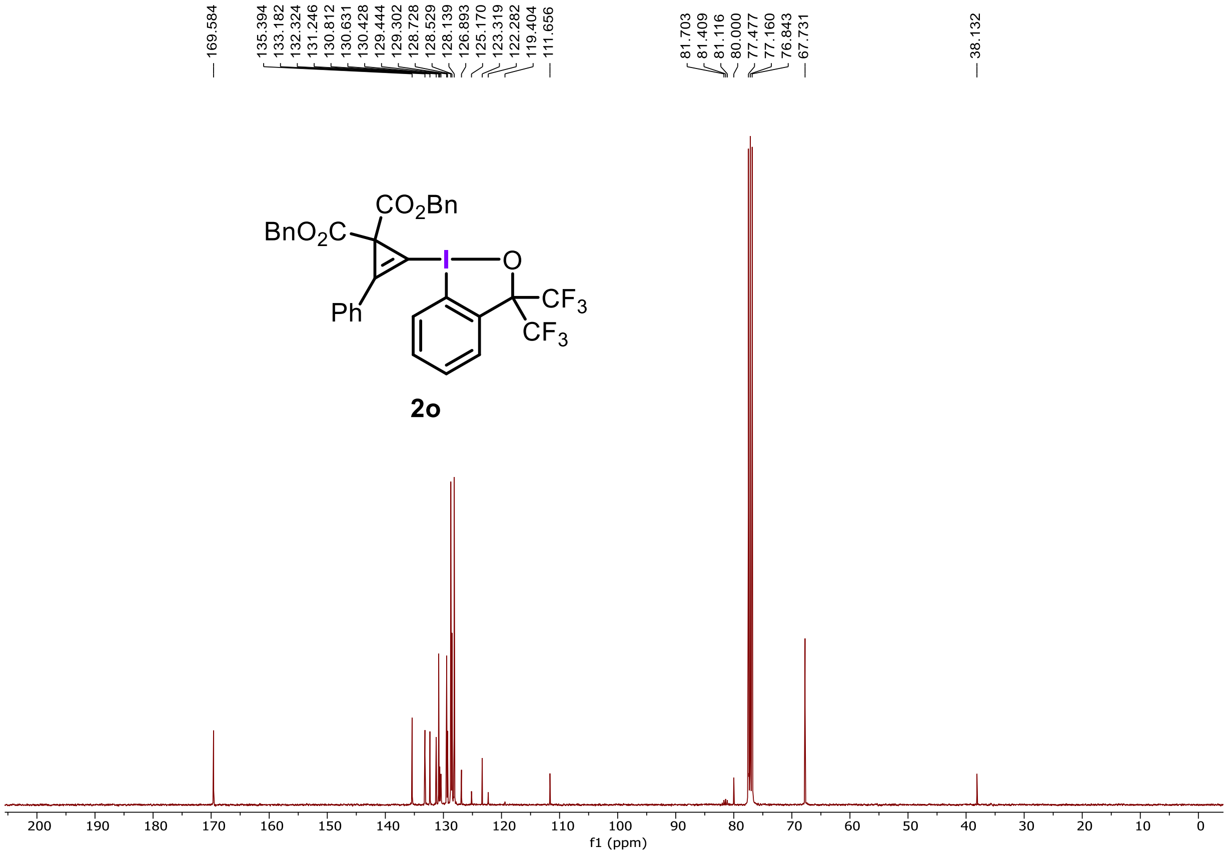

^{19}F NMR (377 MHz, $CDCl_3$) of 2o

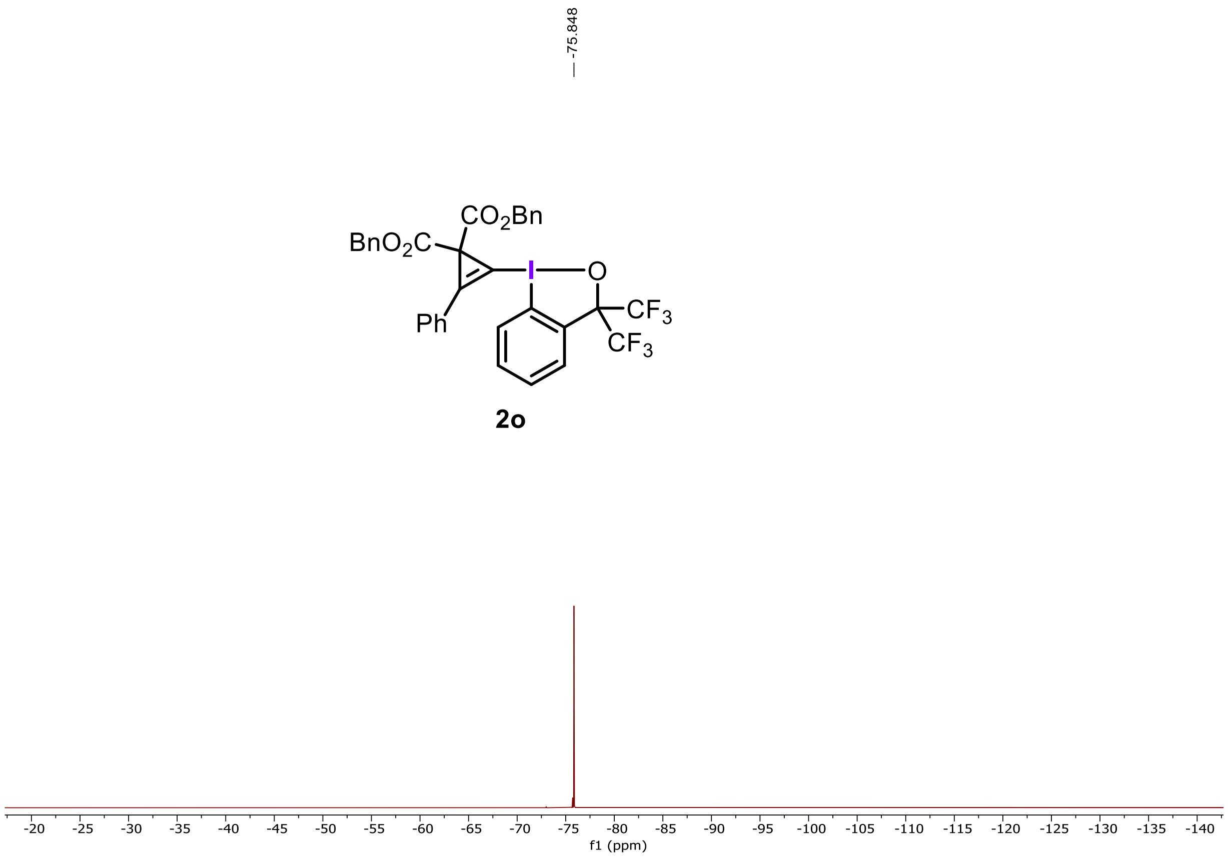

^{1}H NMR (400 MHz, $CDCl_3$) of 3a

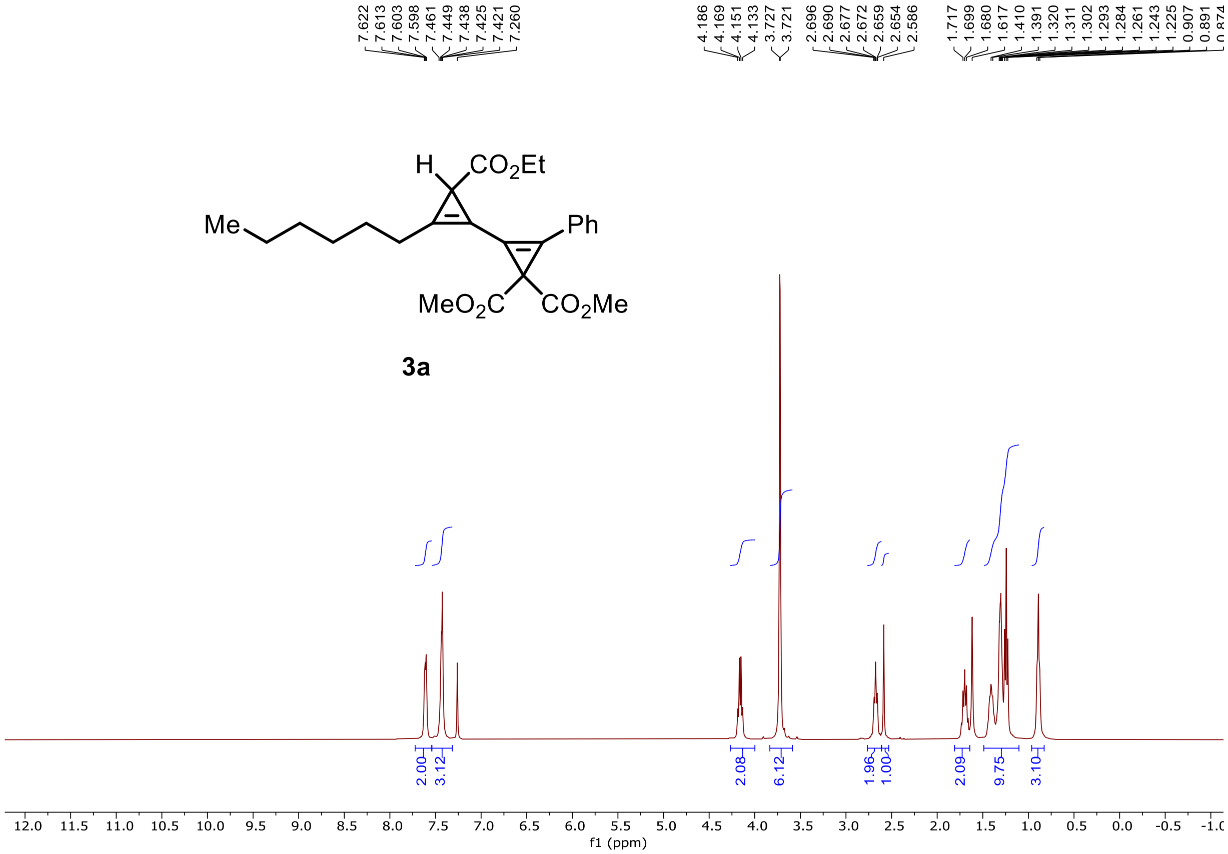

^{13}C NMR (101 MHz, $CDCl_3$) of 3a

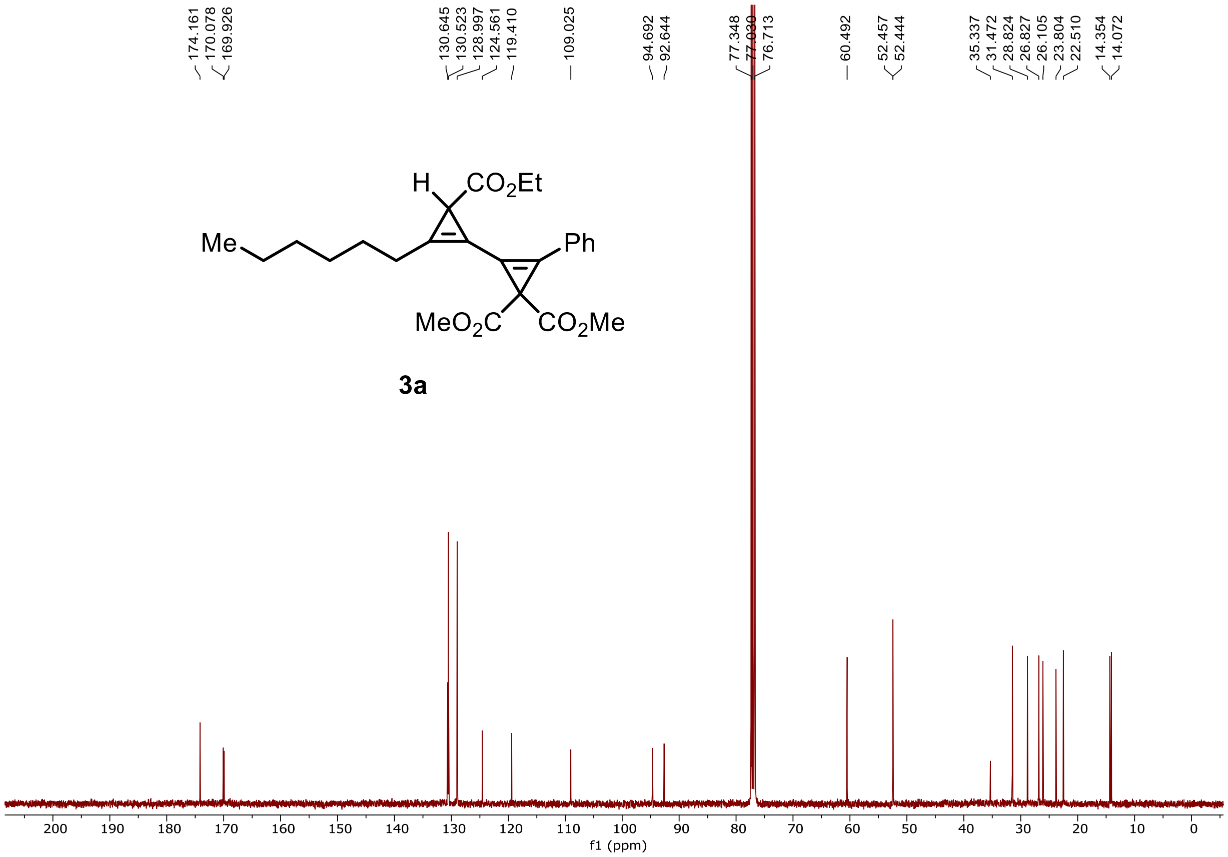

^{1}H NMR (400 MHz, $CDCl_3$) of 3b

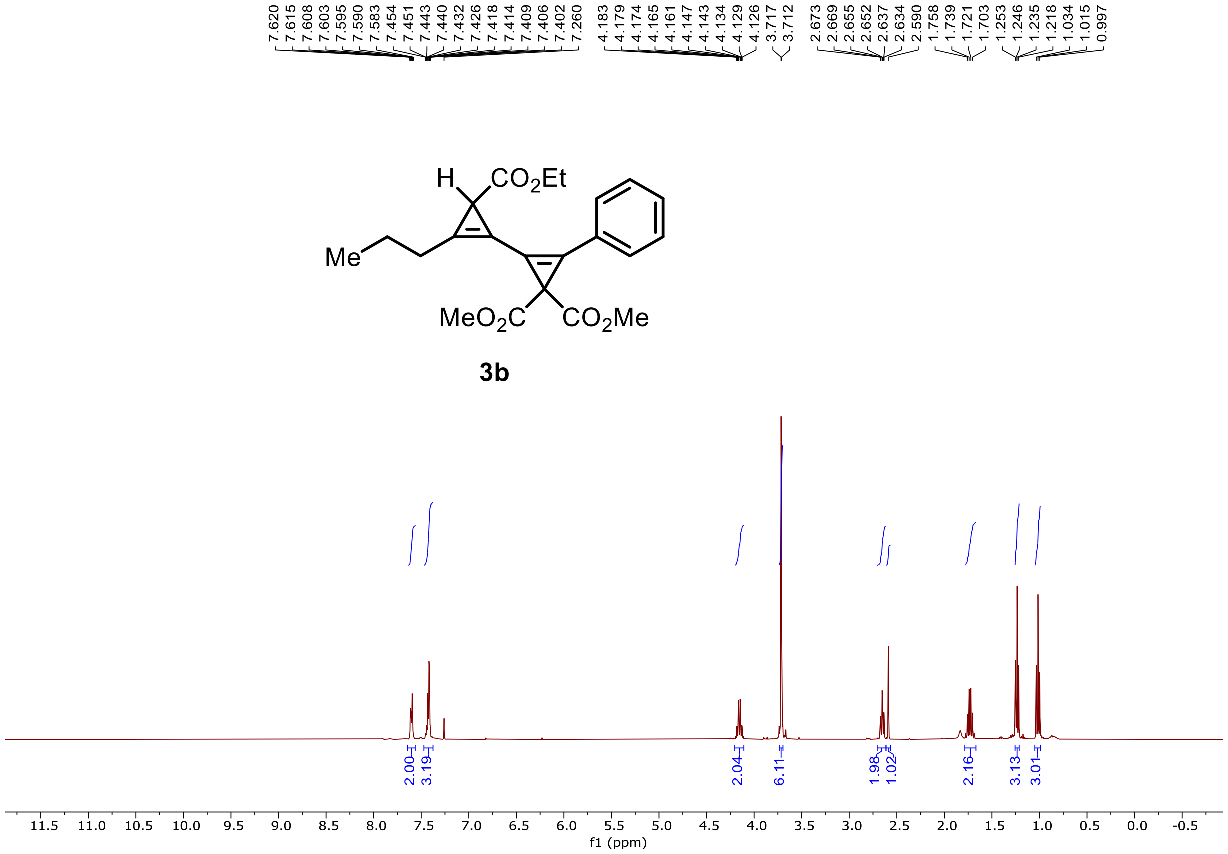

^{13}C NMR (101 MHz, $CDCl_3$) of 3b

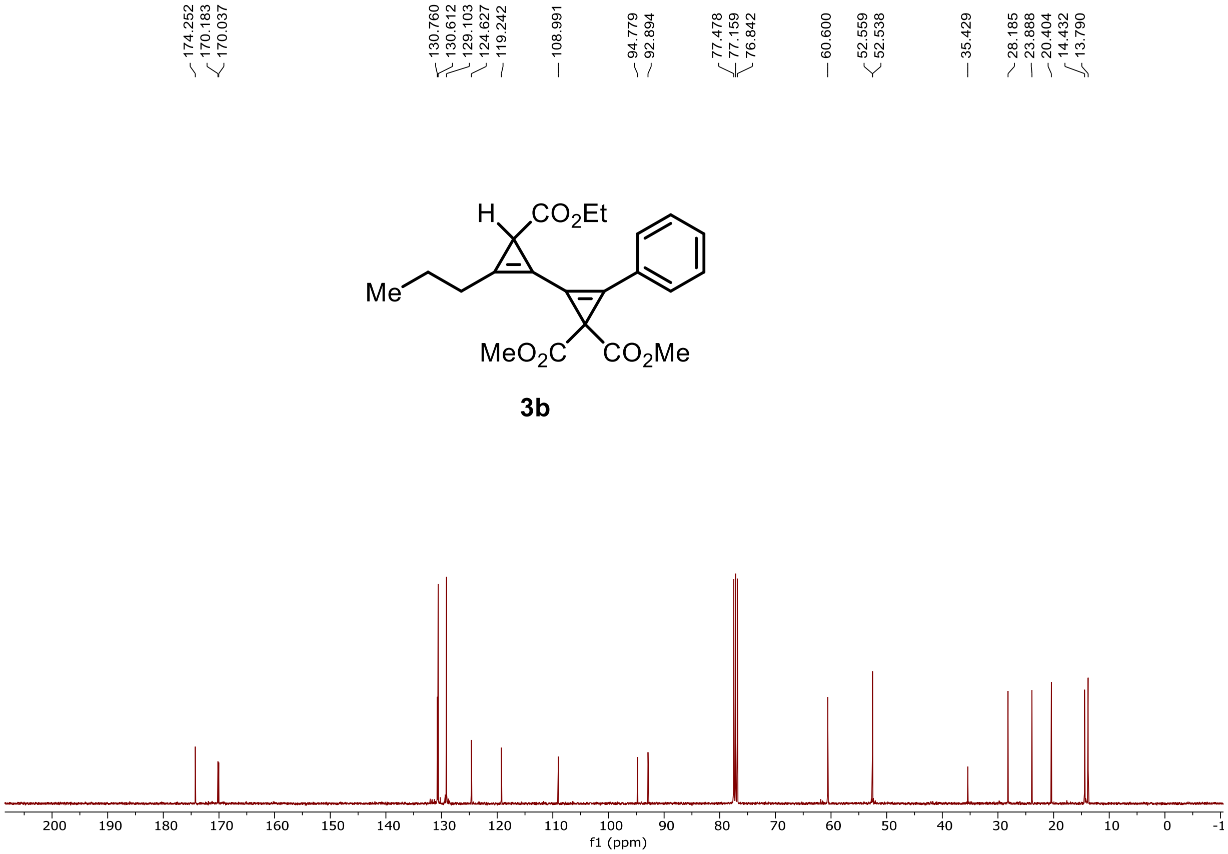

^{1}H NMR (400 MHz, $CDCl_3$) of 3c

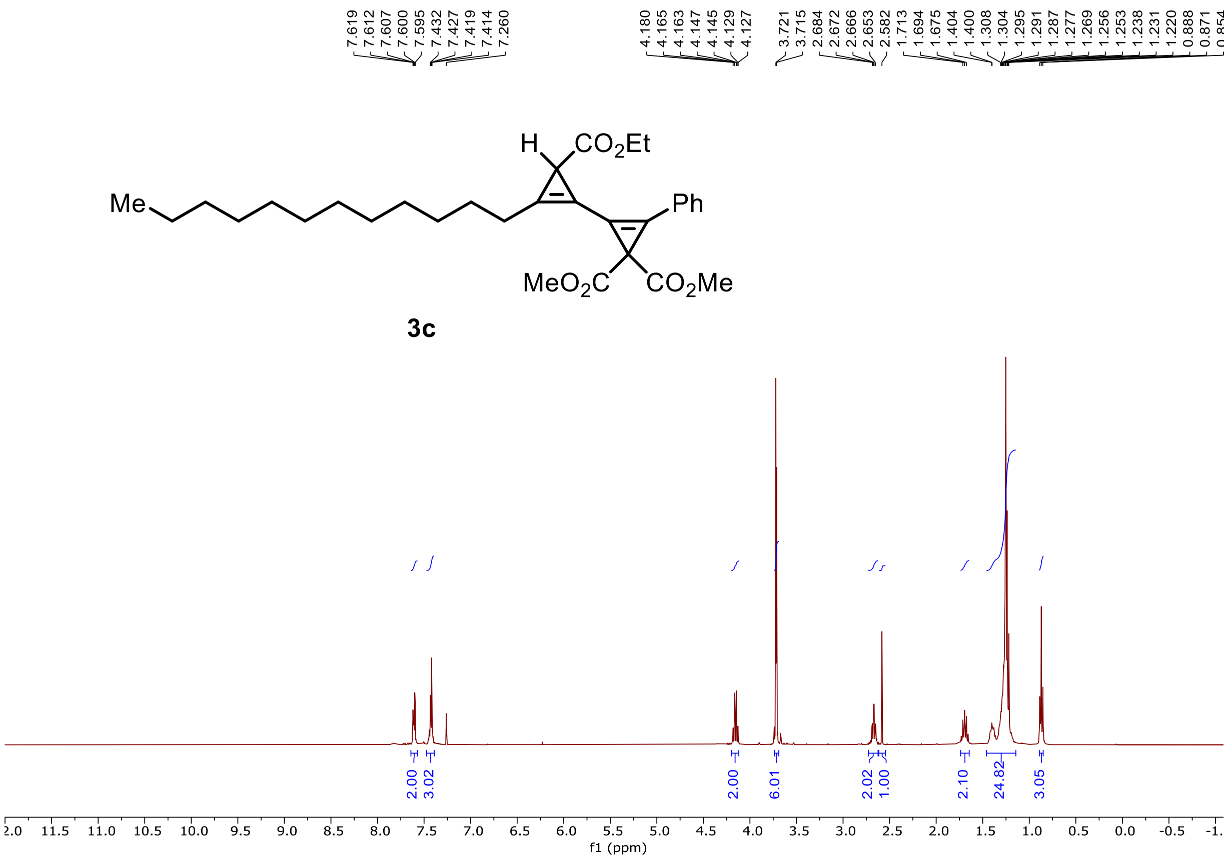

3c

^{13}C NMR (101 MHz, $CDCl_3$) of 3c

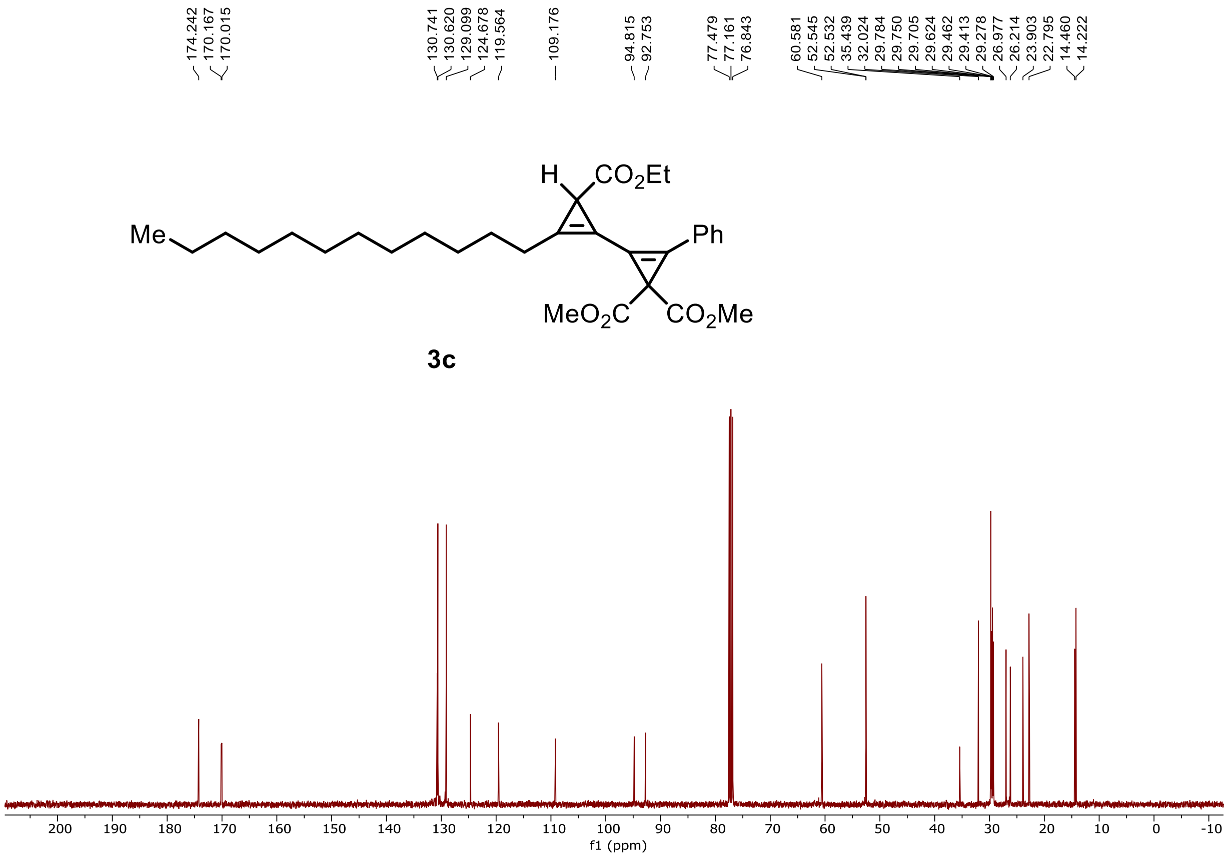

3c

^{1}H NMR (400 MHz, CDCl$_3$) of 3d

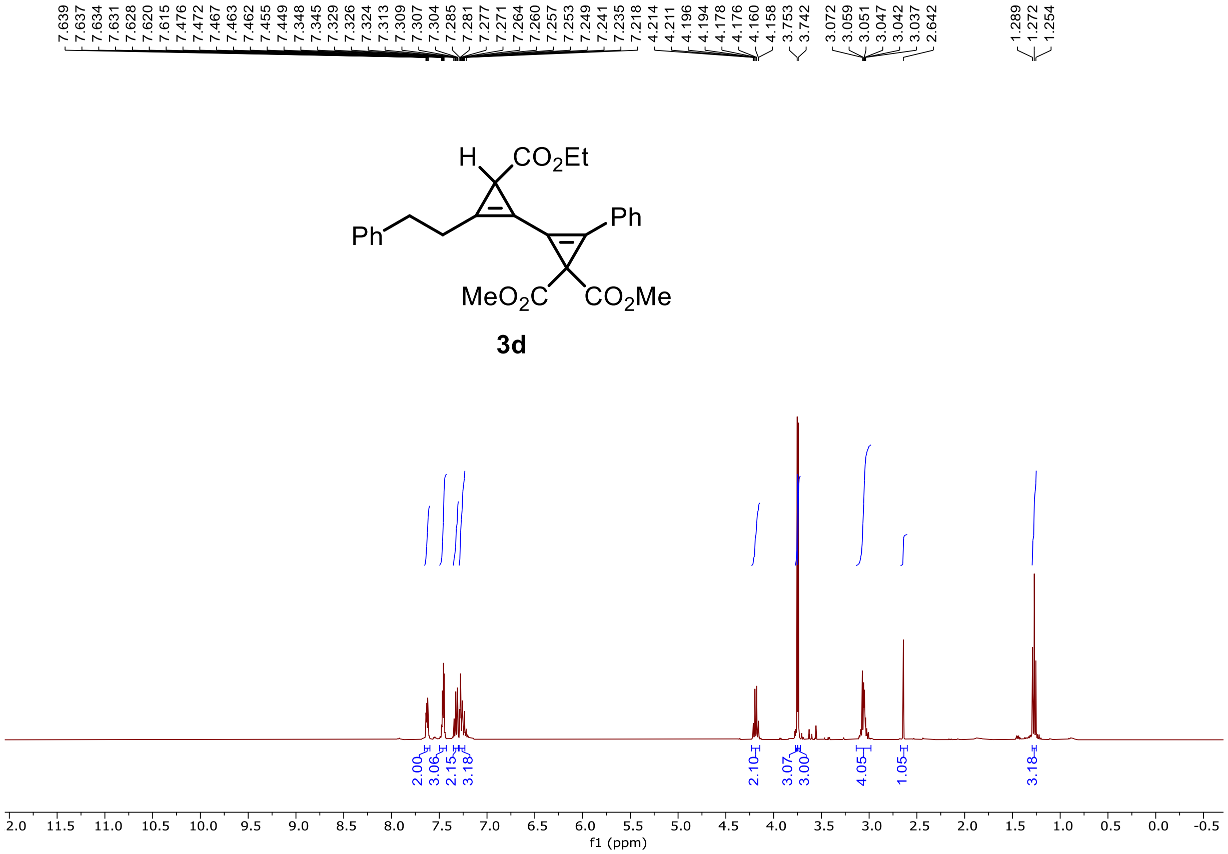

^{13}C NMR (101 MHz, CDCl$_3$) of 3d

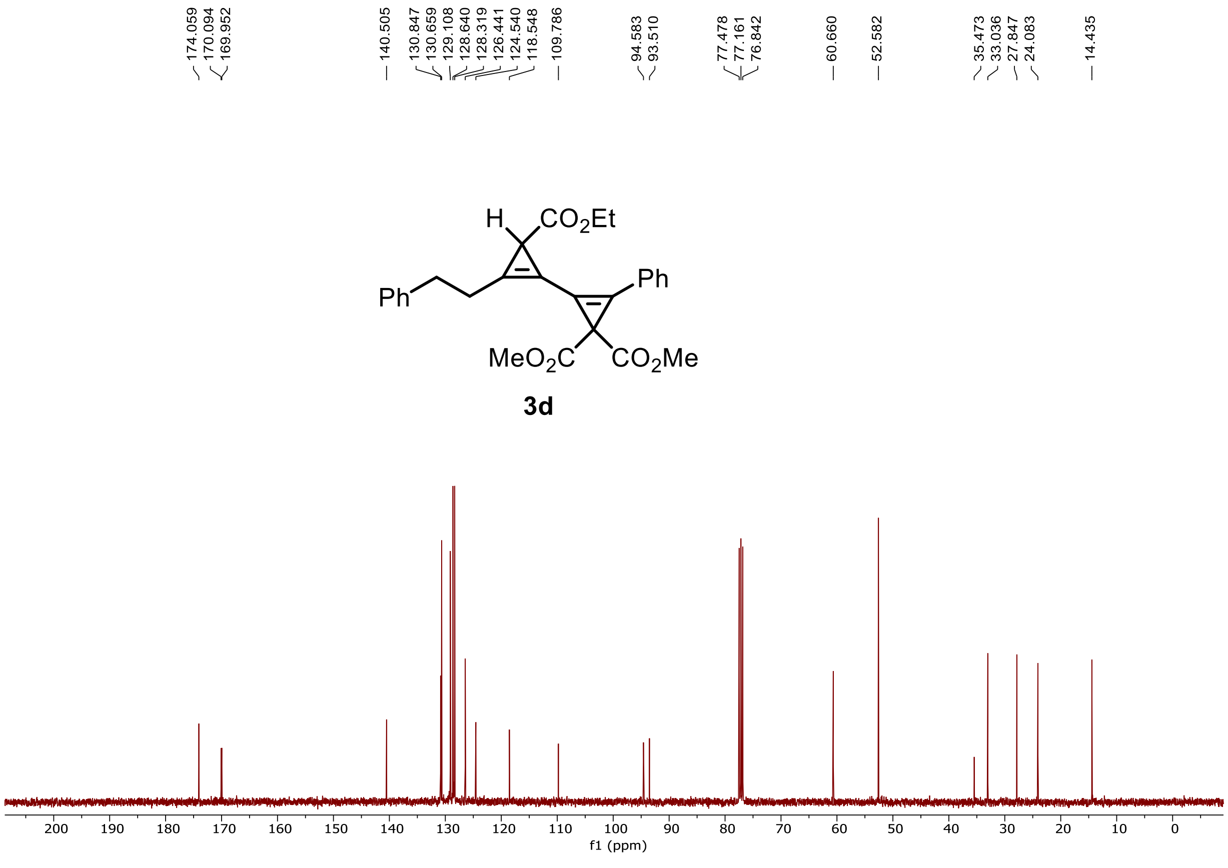

^{1}H NMR (400 MHz, $CDCl_3$) of 3e

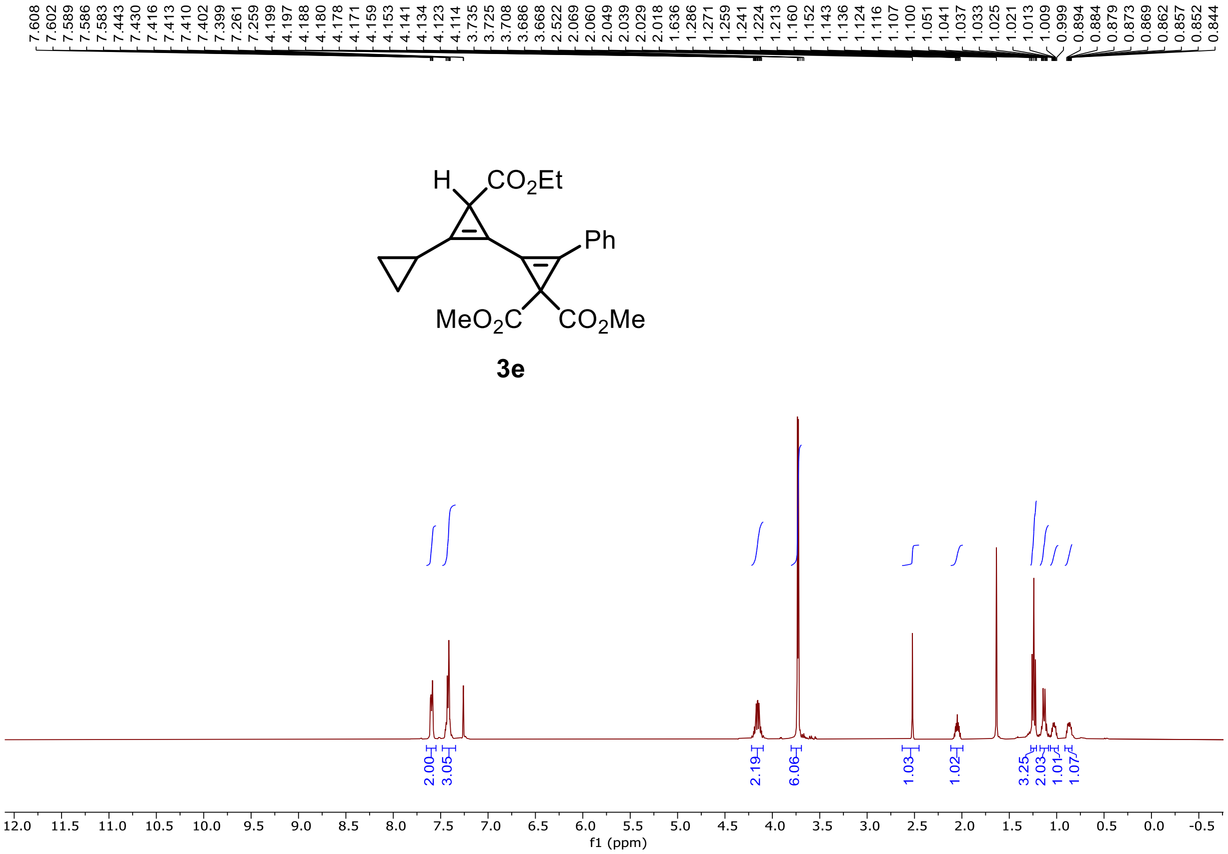

^{13}C NMR (101 MHz, $CDCl_3$) of 3e

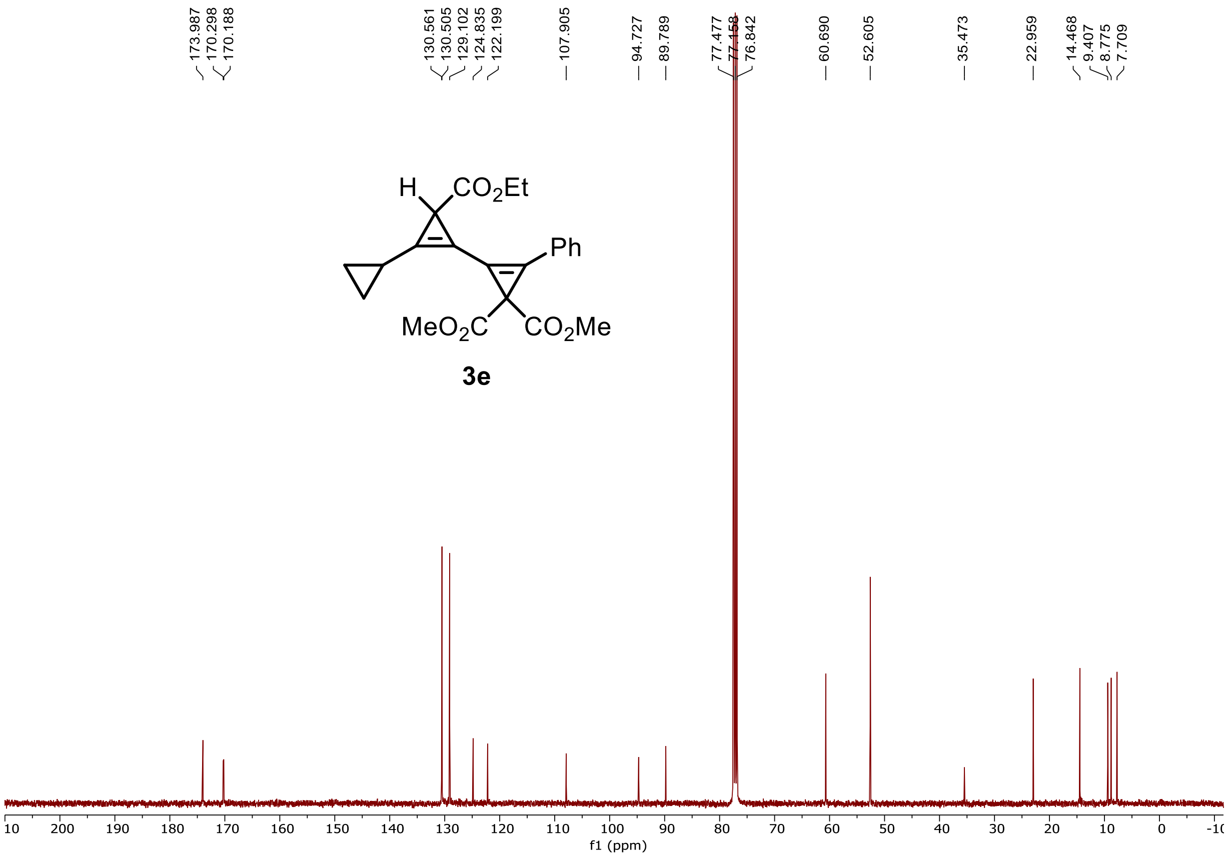

^{1}H NMR (400 MHz, CDCl$_3$) of 3f

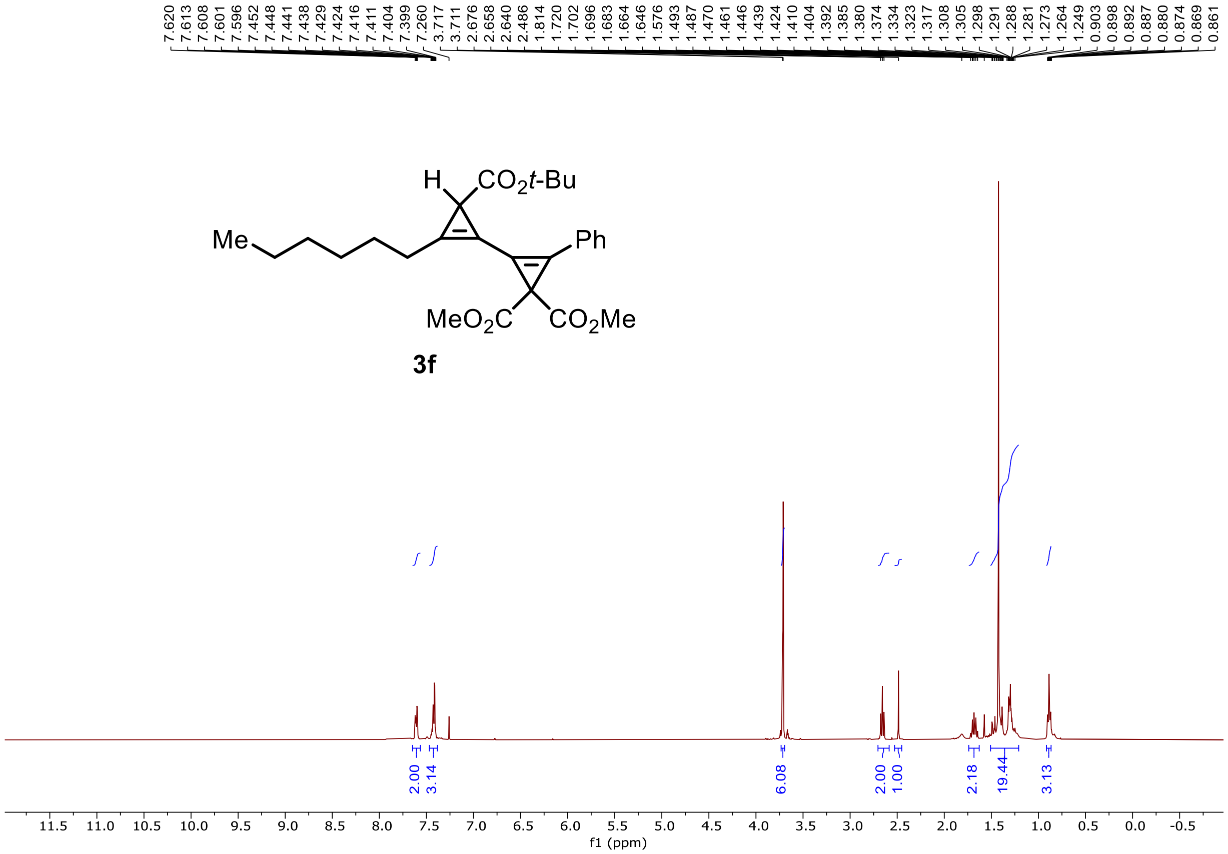

^{13}C NMR (101 MHz, CDCl$_3$) of 3f

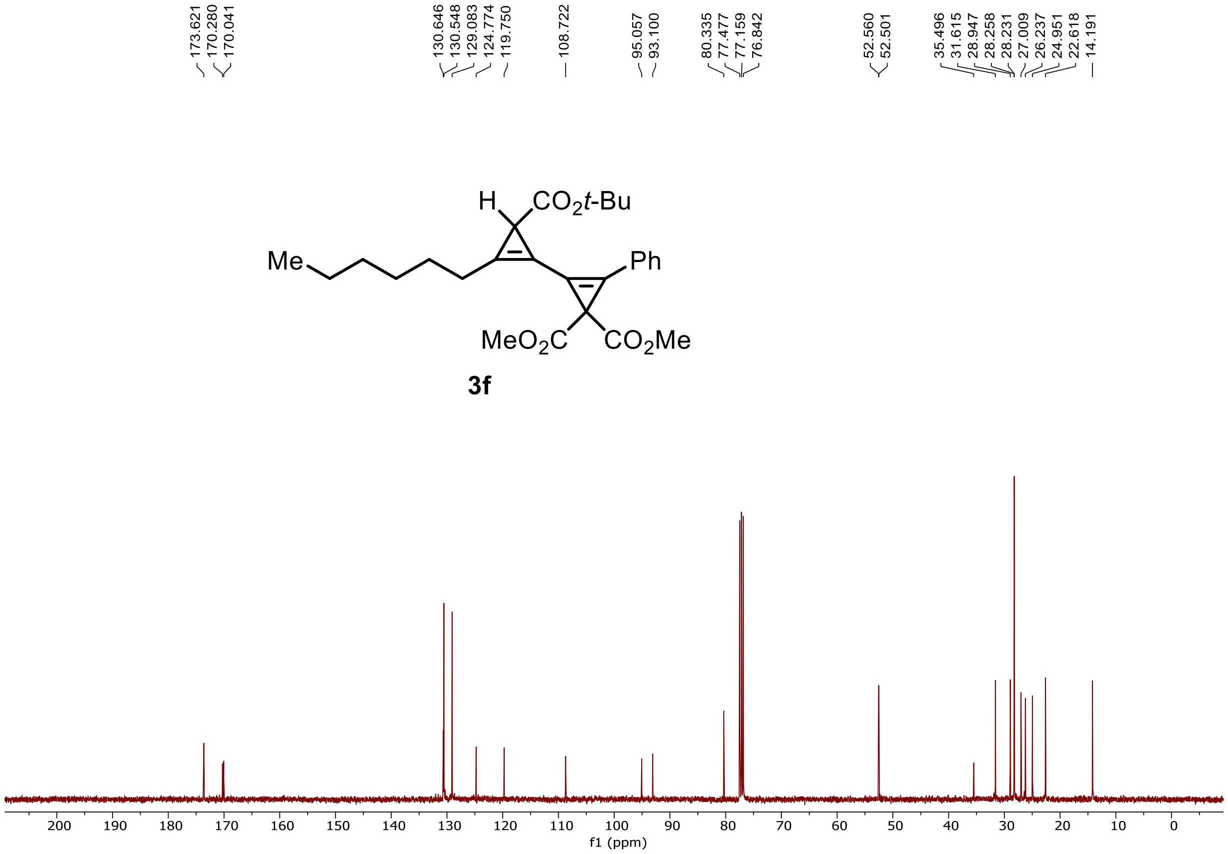

^{1}H NMR (400 MHz, $CDCl_3$) of 3g

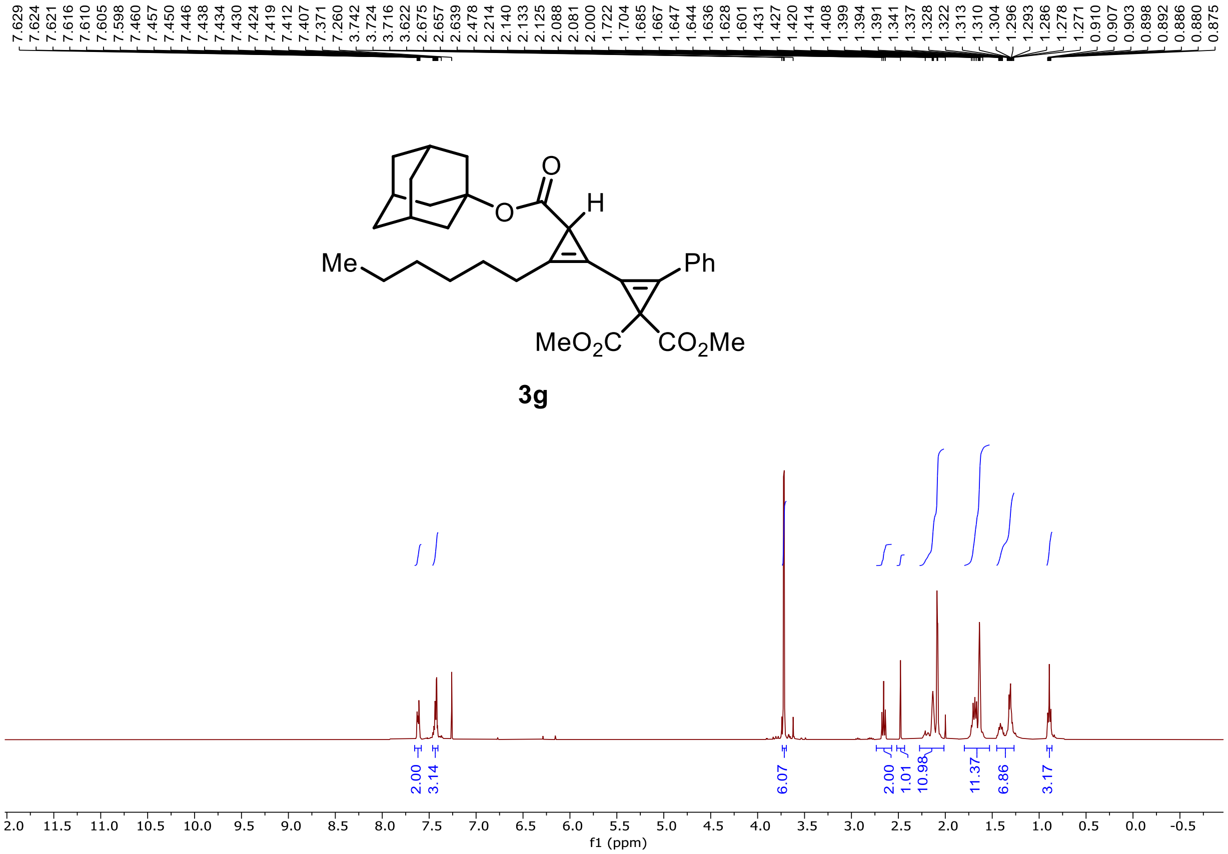

^{13}C NMR (101 MHz, $CDCl_3$) of 3g

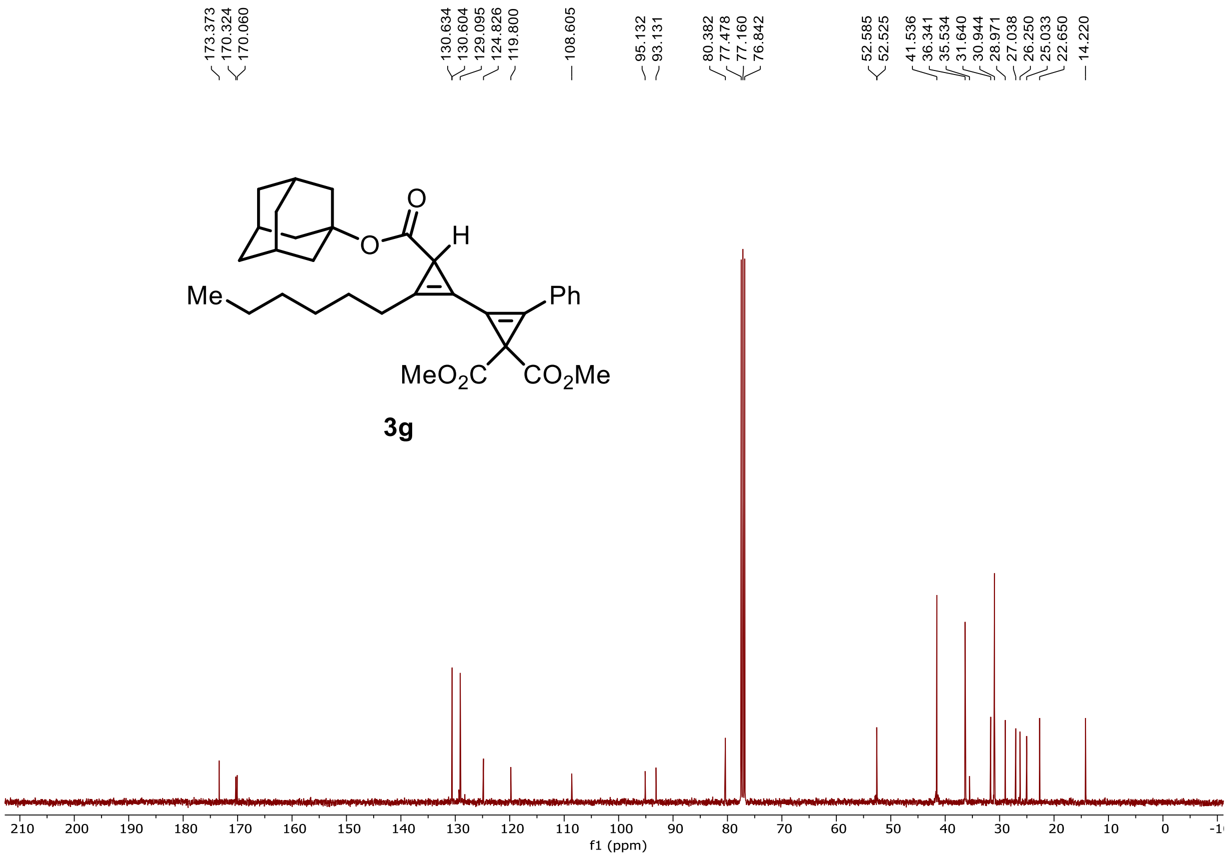

^{1}H NMR (400 MHz, $CDCl_3$) of 3h

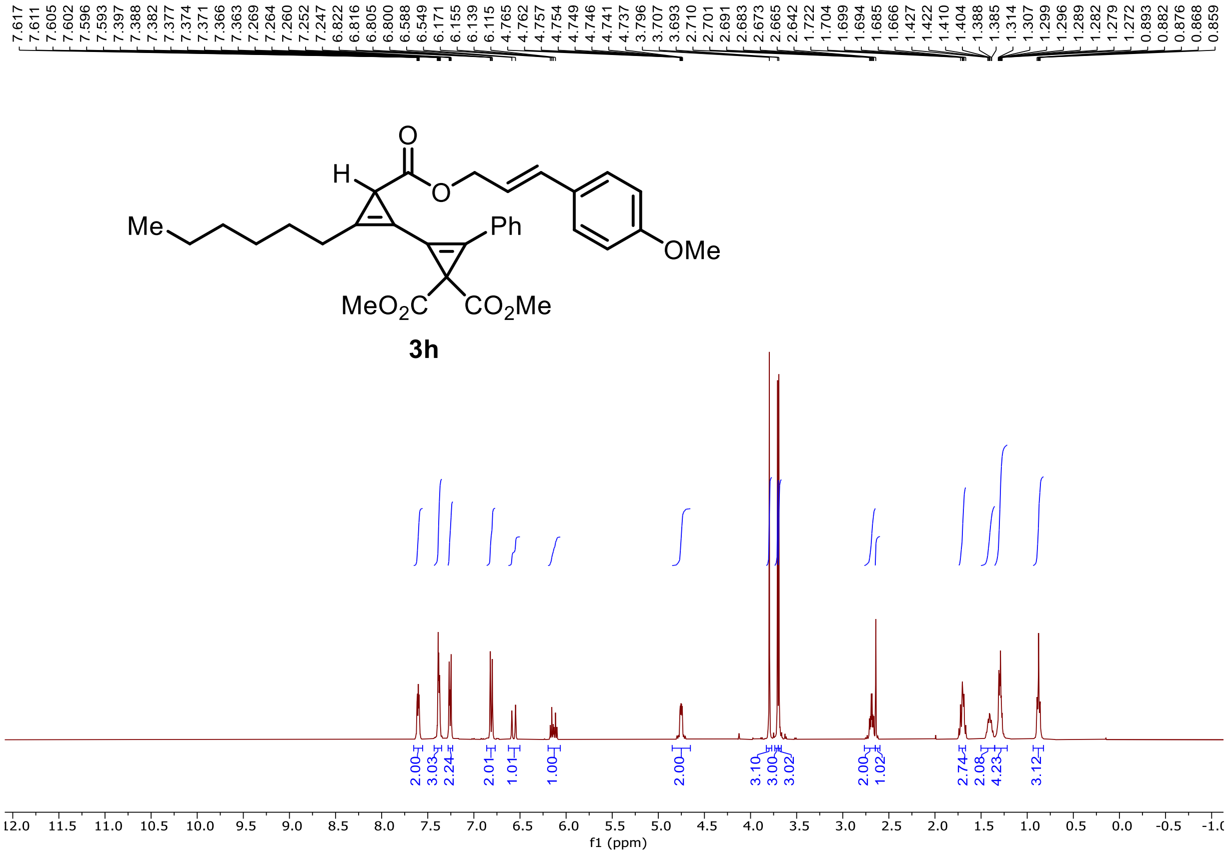

^{13}C NMR (101 MHz, $CDCl_3$) of 3h

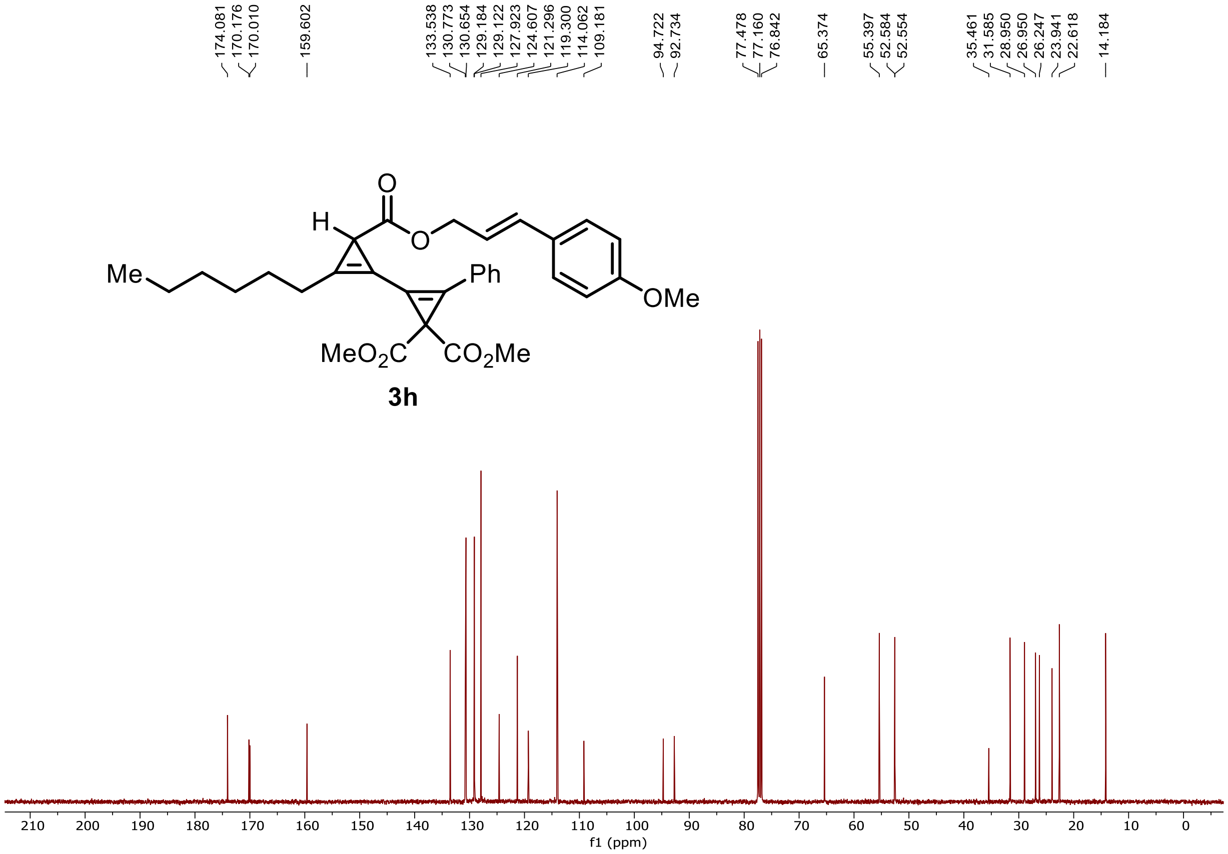

^{1}H NMR (400 MHz, $CDCl_3$) of 3i

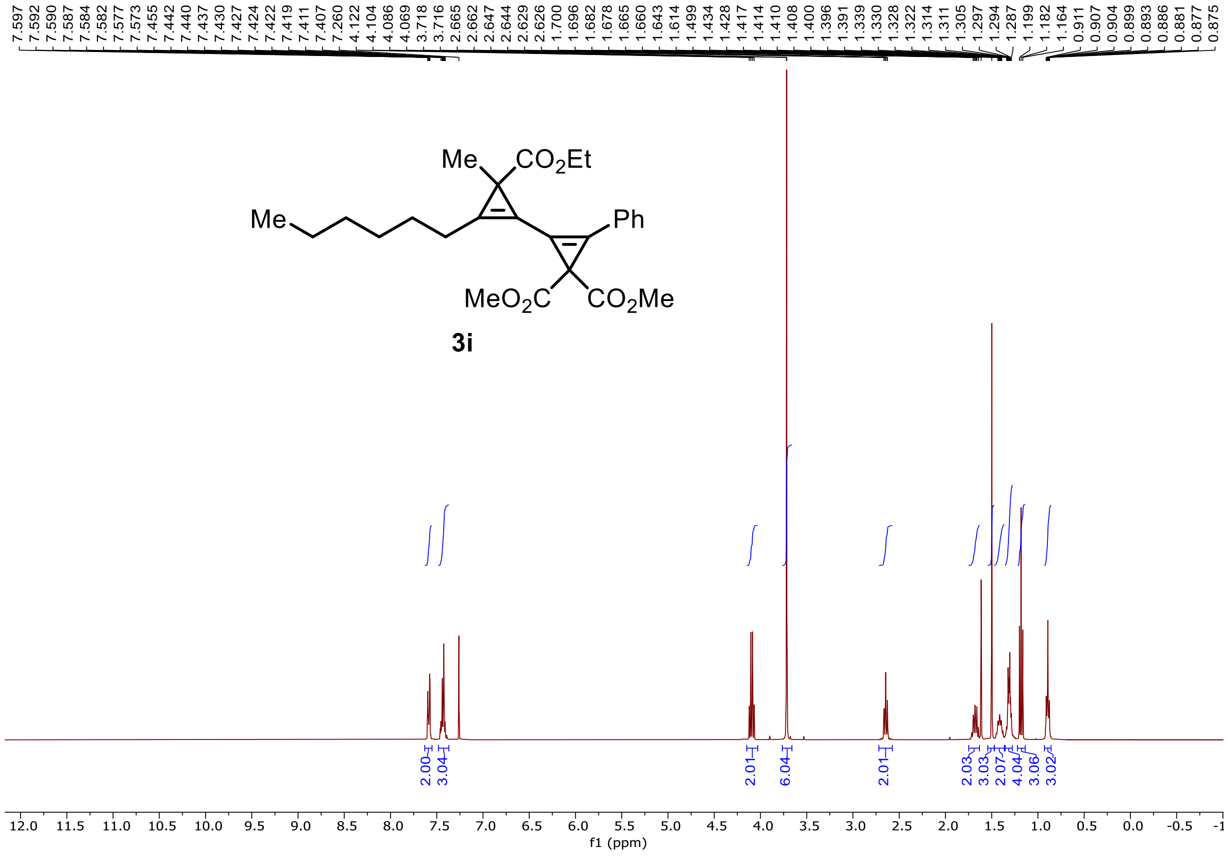

^{13}C NMR (101 MHz, $CDCl_3$) of 3i

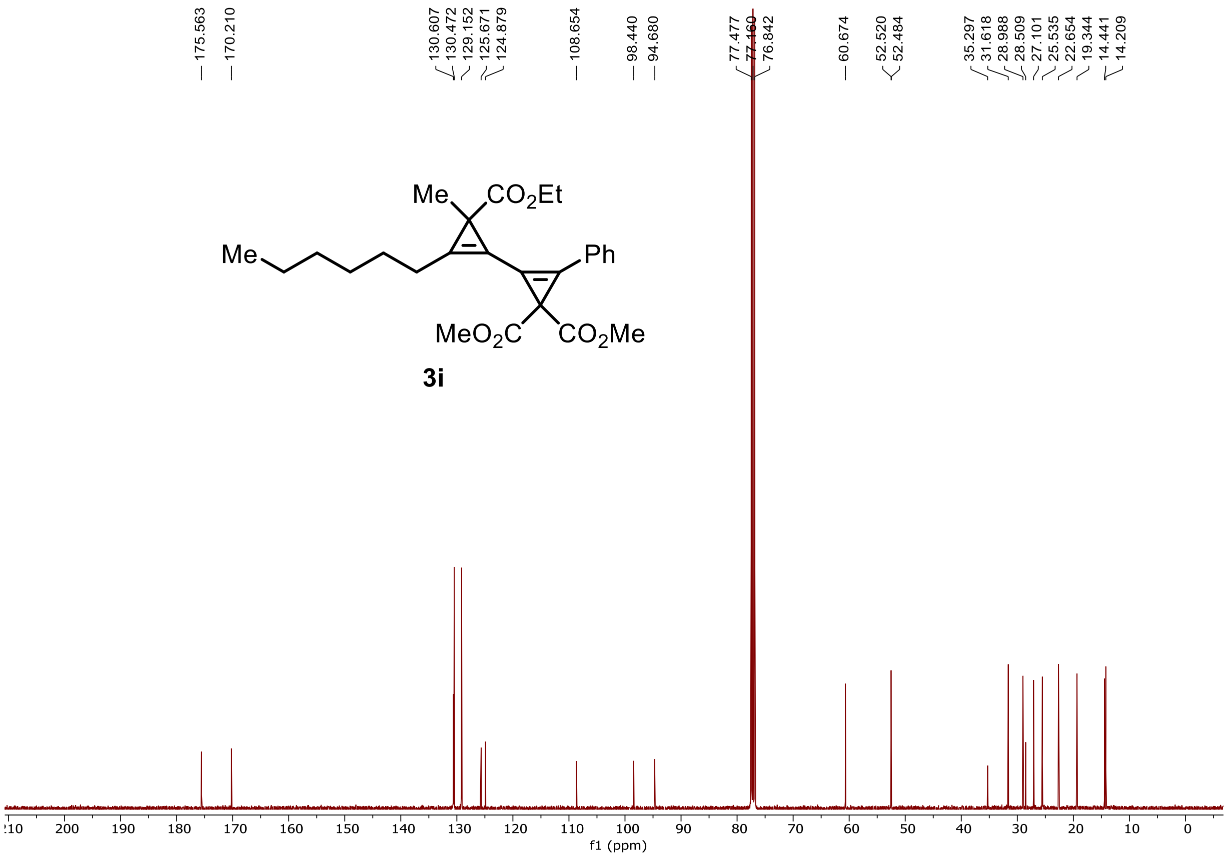

^{1}H NMR (400 MHz, $CDCl_3$) of 3j

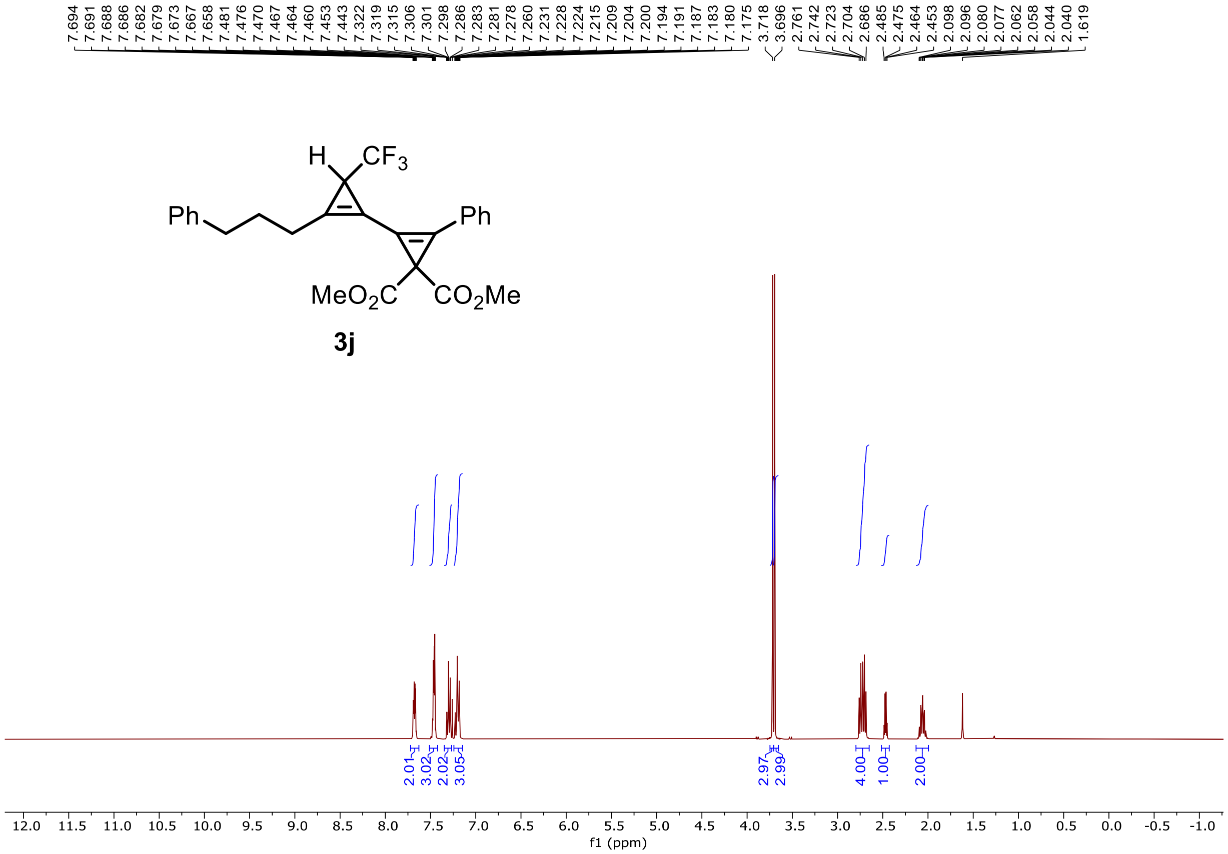

^{13}C NMR (101 MHz, $CDCl_3$) of 3j

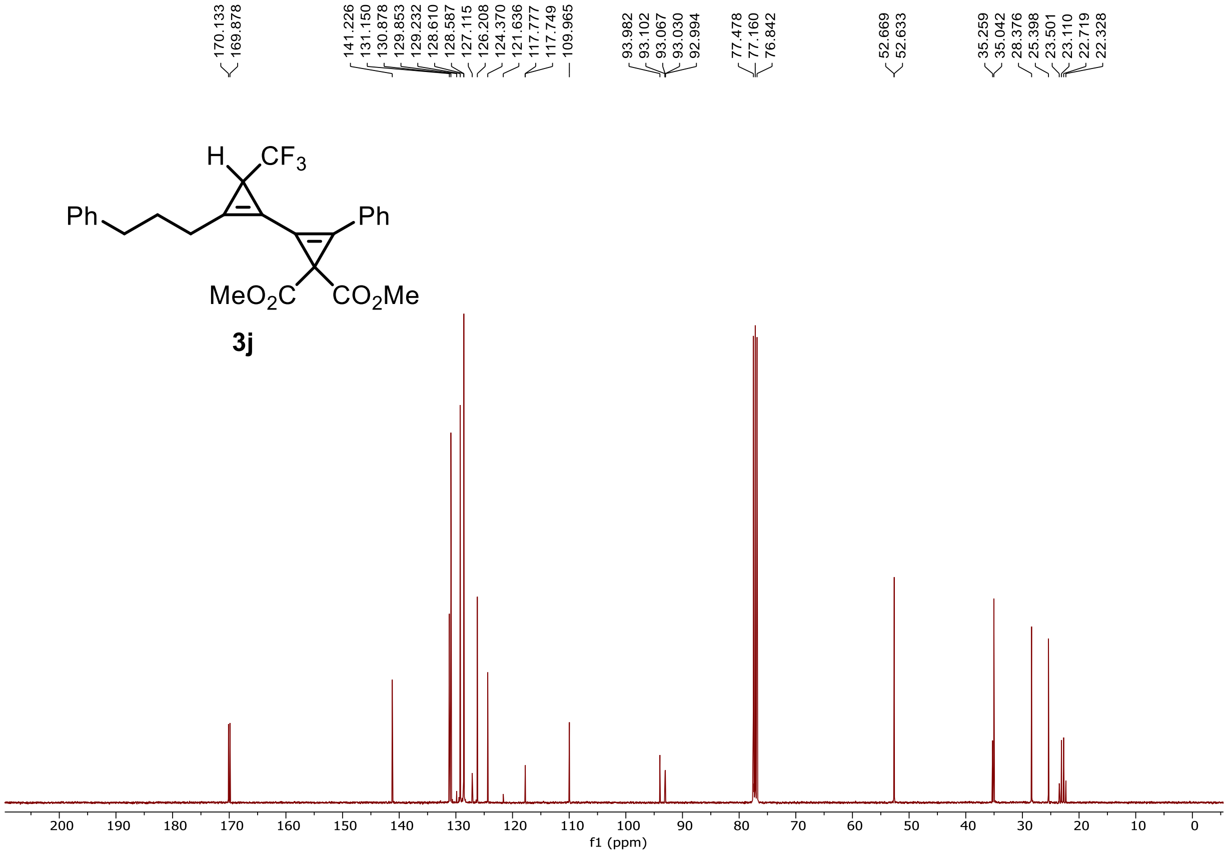

^{19}F NMR (377 MHz, CDCl$_3$) of 3j

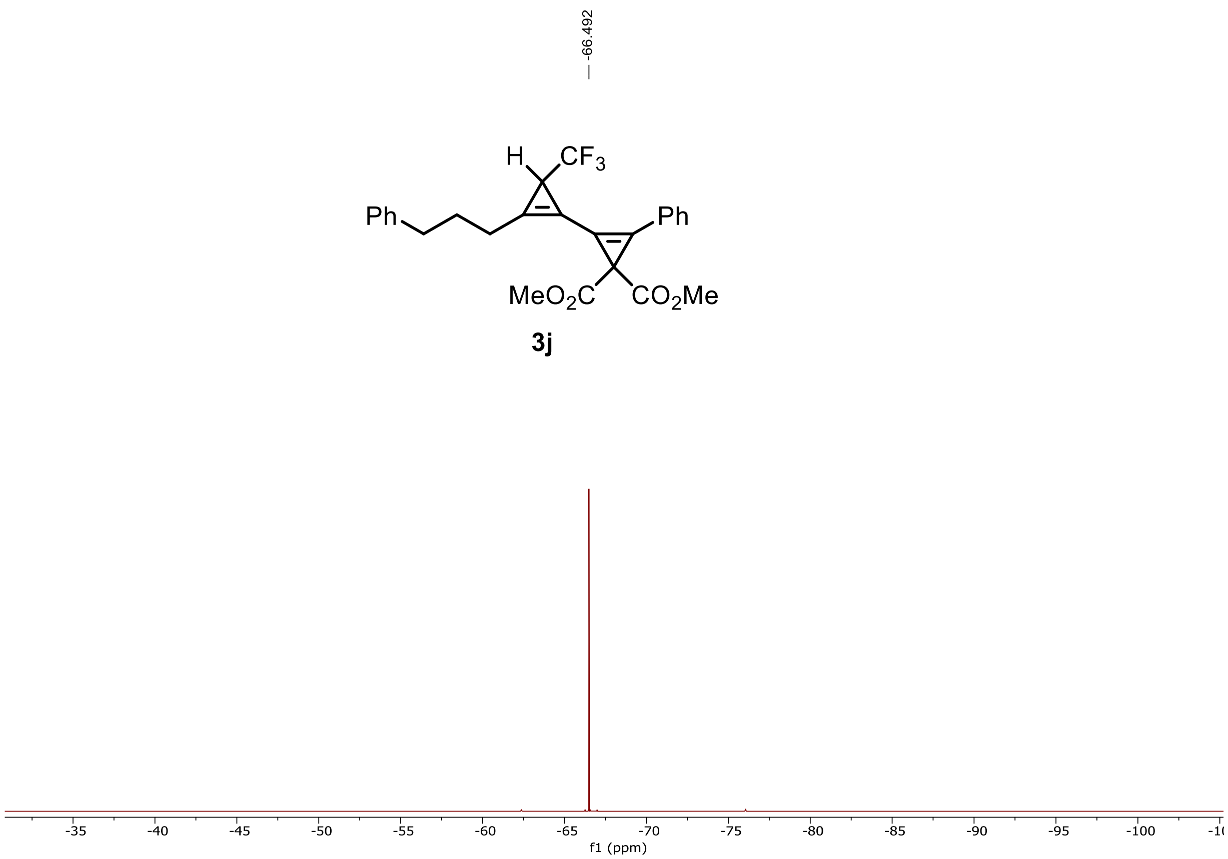

^{1}H NMR (400 MHz, CDCl$_3$) of 3k

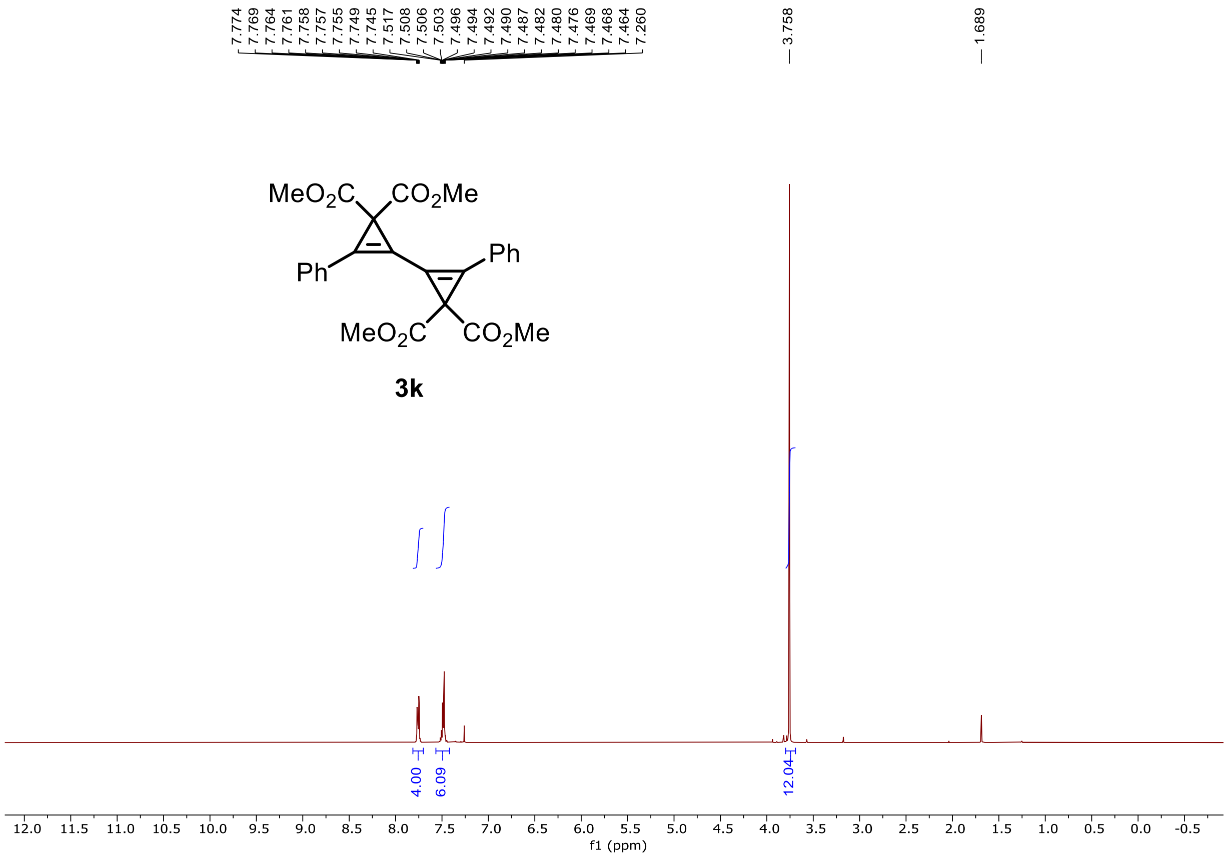

^{13}C NMR (101 MHz, $CDCl_3$) of 3k

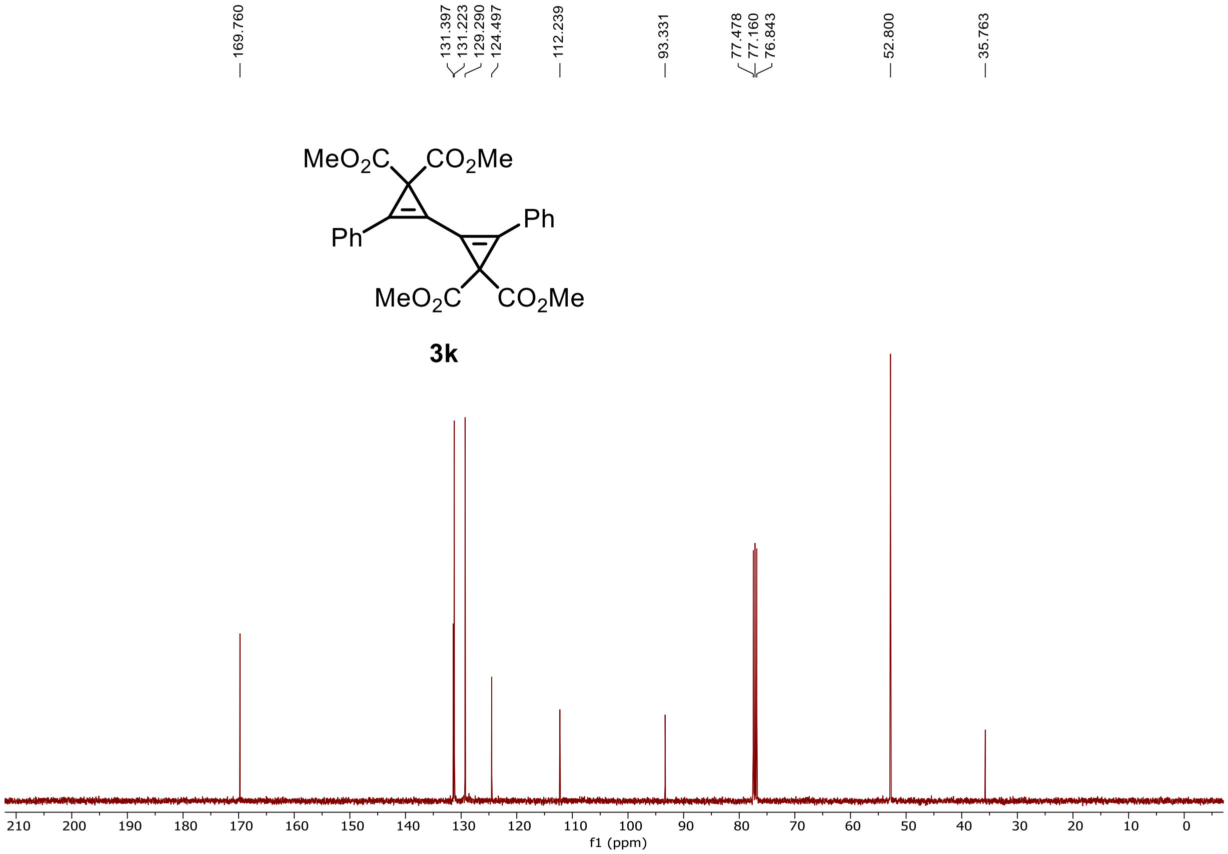

^{1}H NMR (400 MHz, $CDCl_3$) of 3l

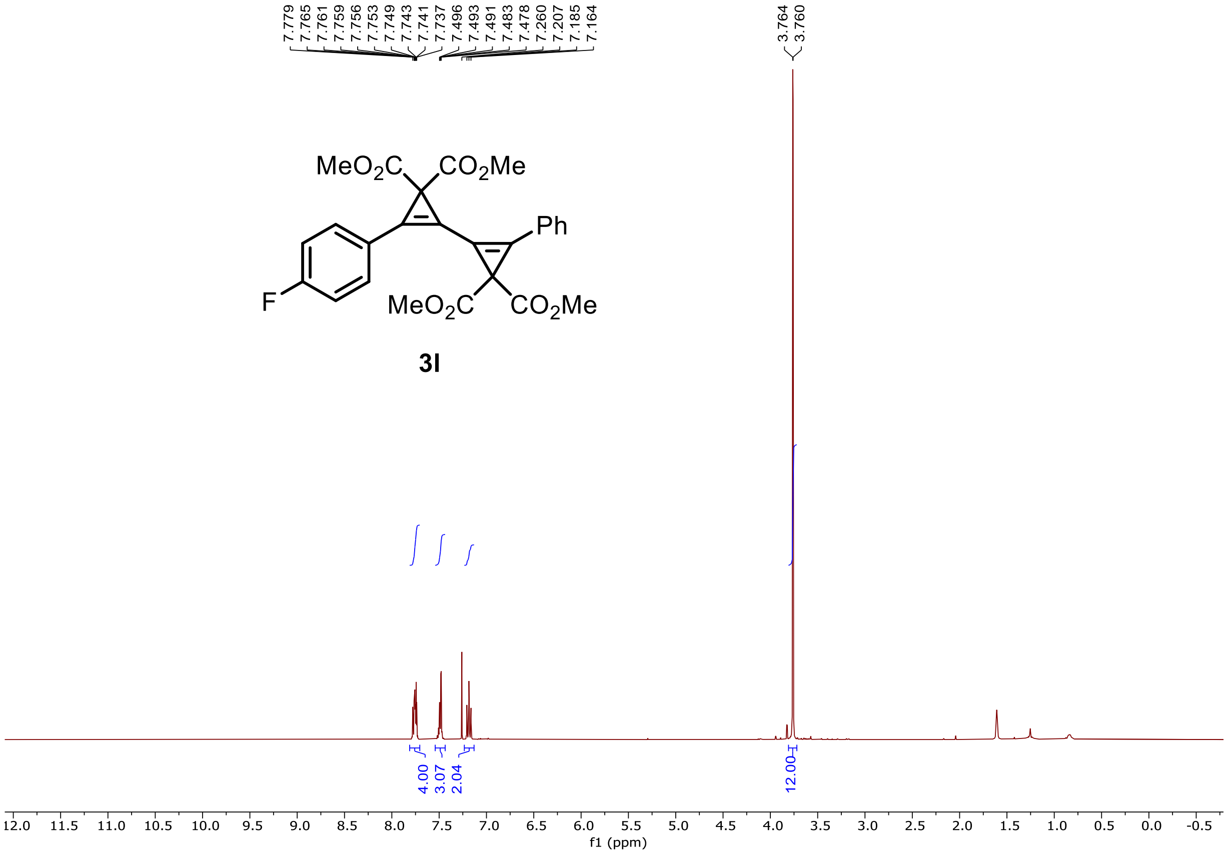

^{13}C NMR (101 MHz, $CDCl_3$) of 3l

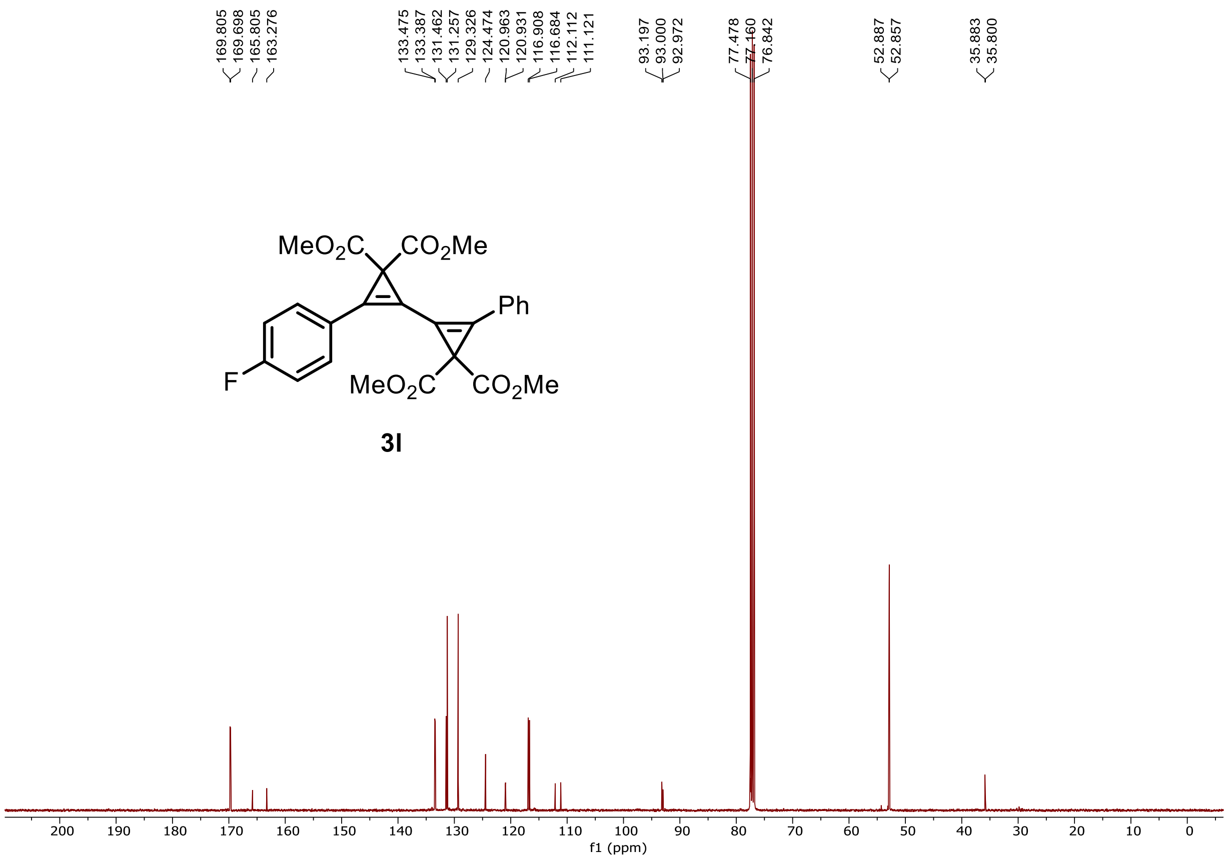

^{19}F NMR (377 MHz, $CDCl_3$) of 3l

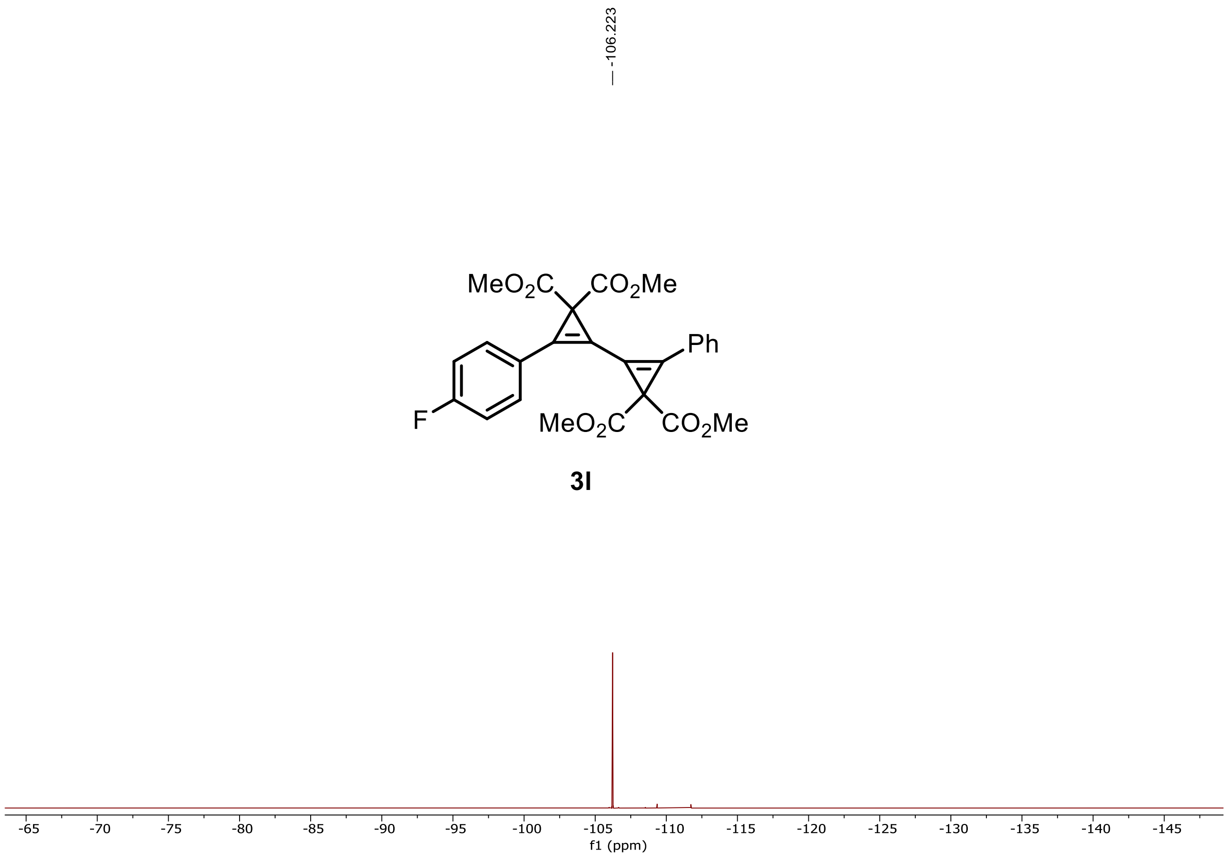

^{1}H NMR (400 MHz, $CDCl_3$) of 3m

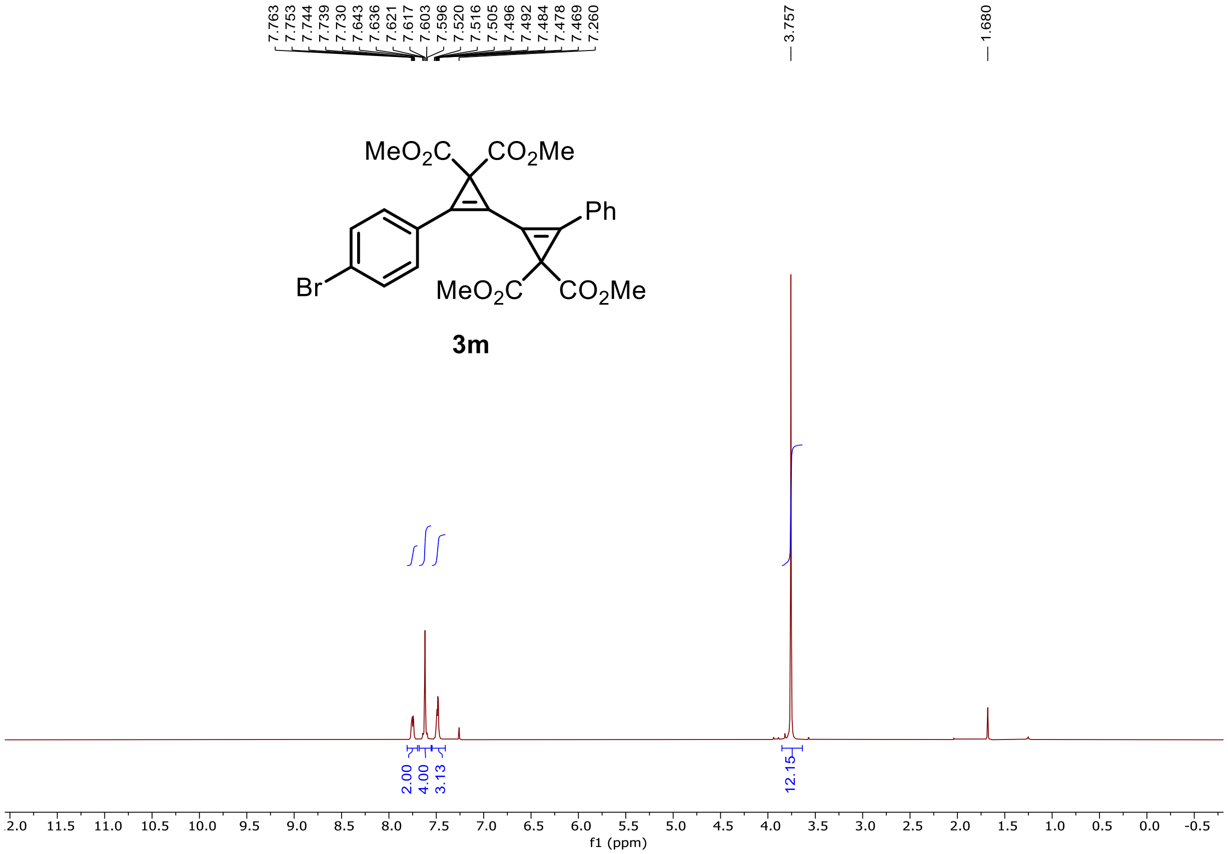

^{13}C NMR (101 MHz, $CDCl_3$) of 3m

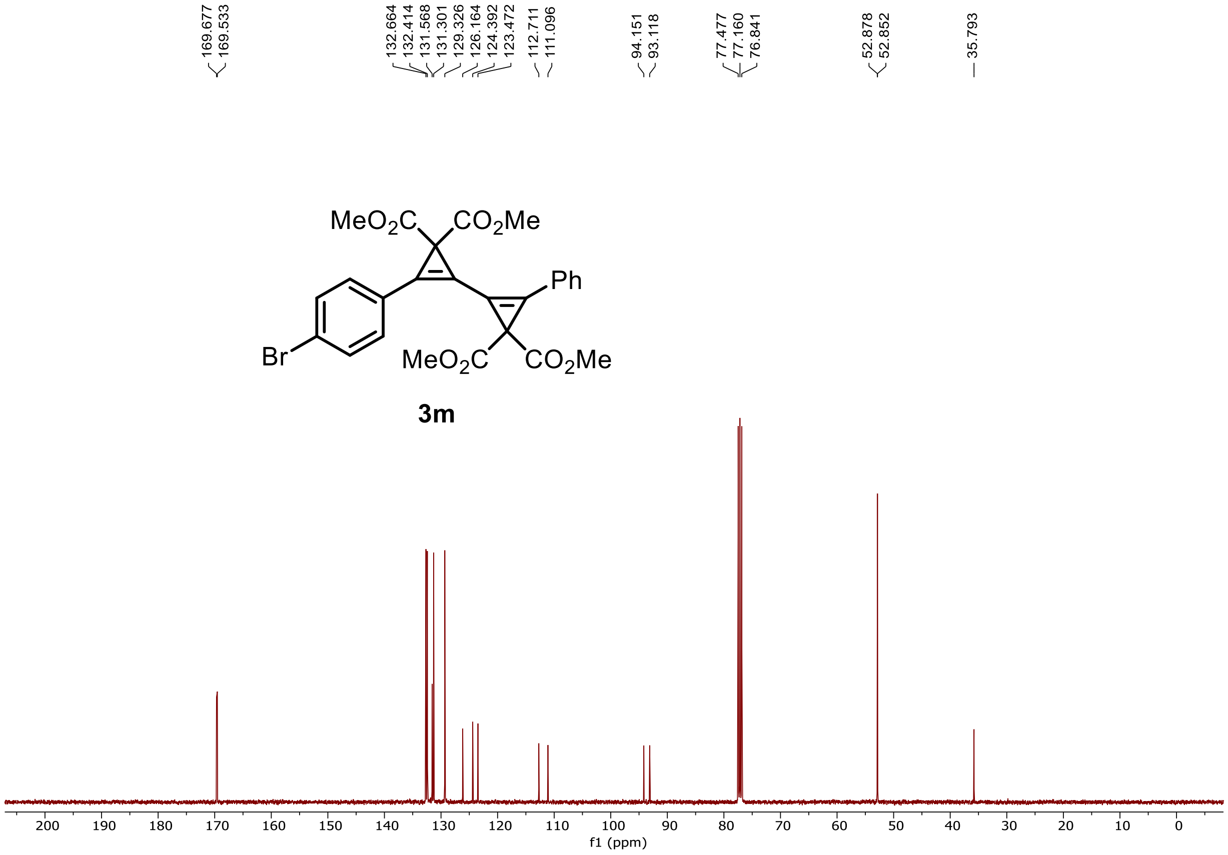

^{1}H NMR (400 MHz, $CDCl_3$) of 3n

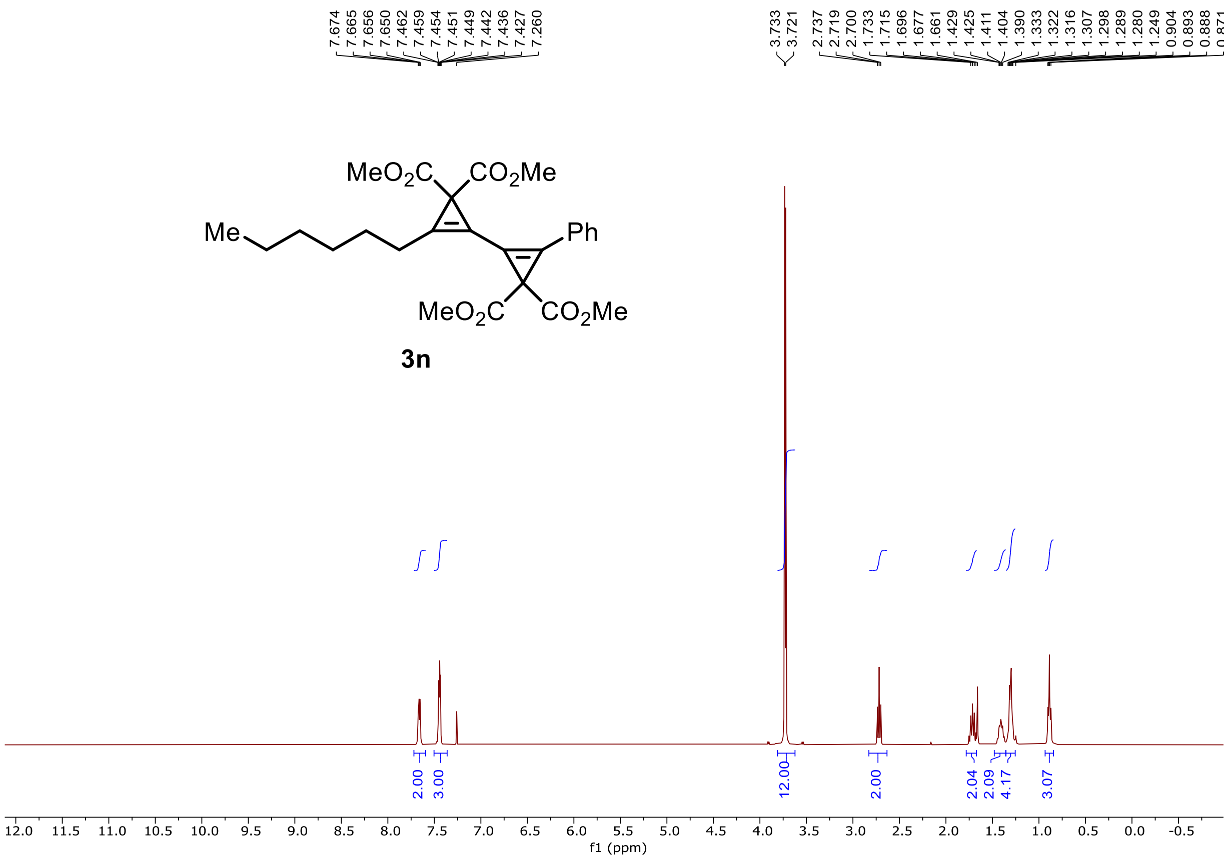

^{13}C NMR (101 MHz, $CDCl_3$) of 3n

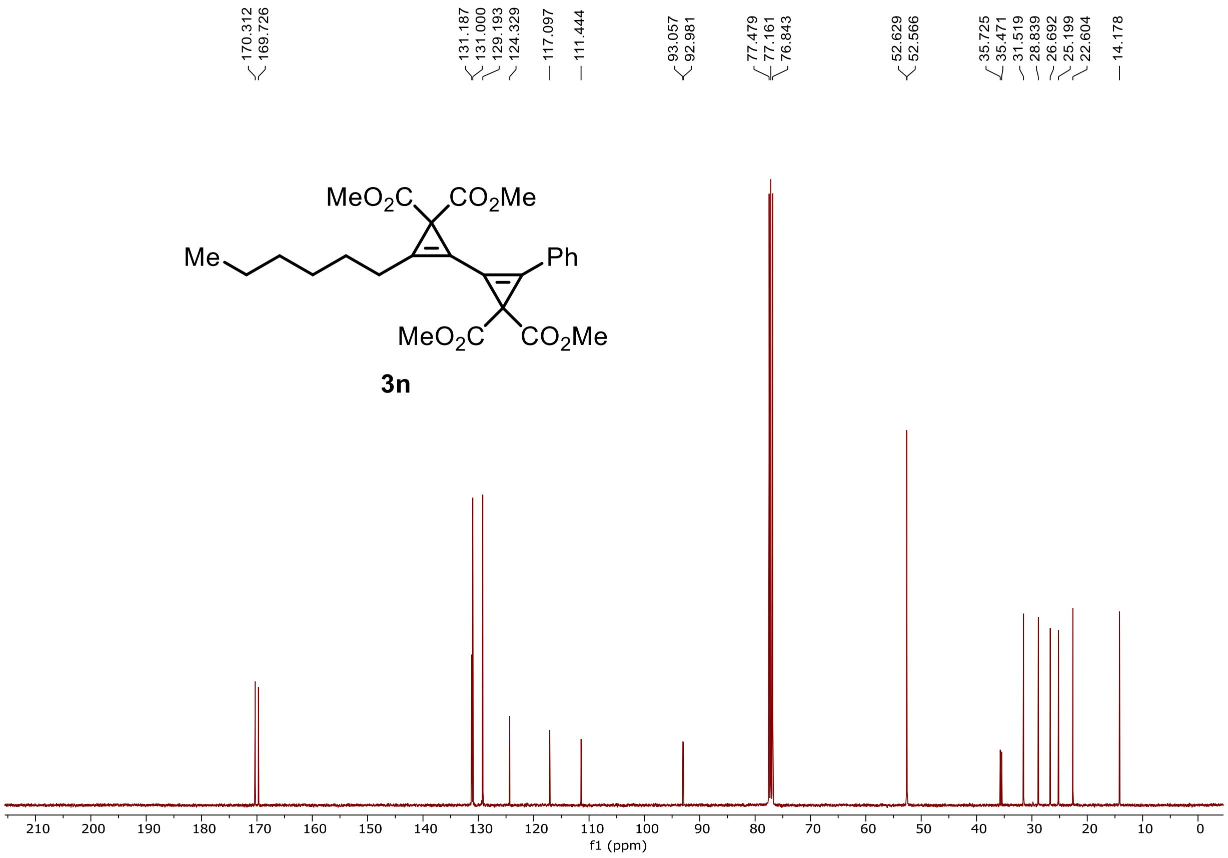

¹H NMR (400 MHz, $CDCl_3$) of 3o

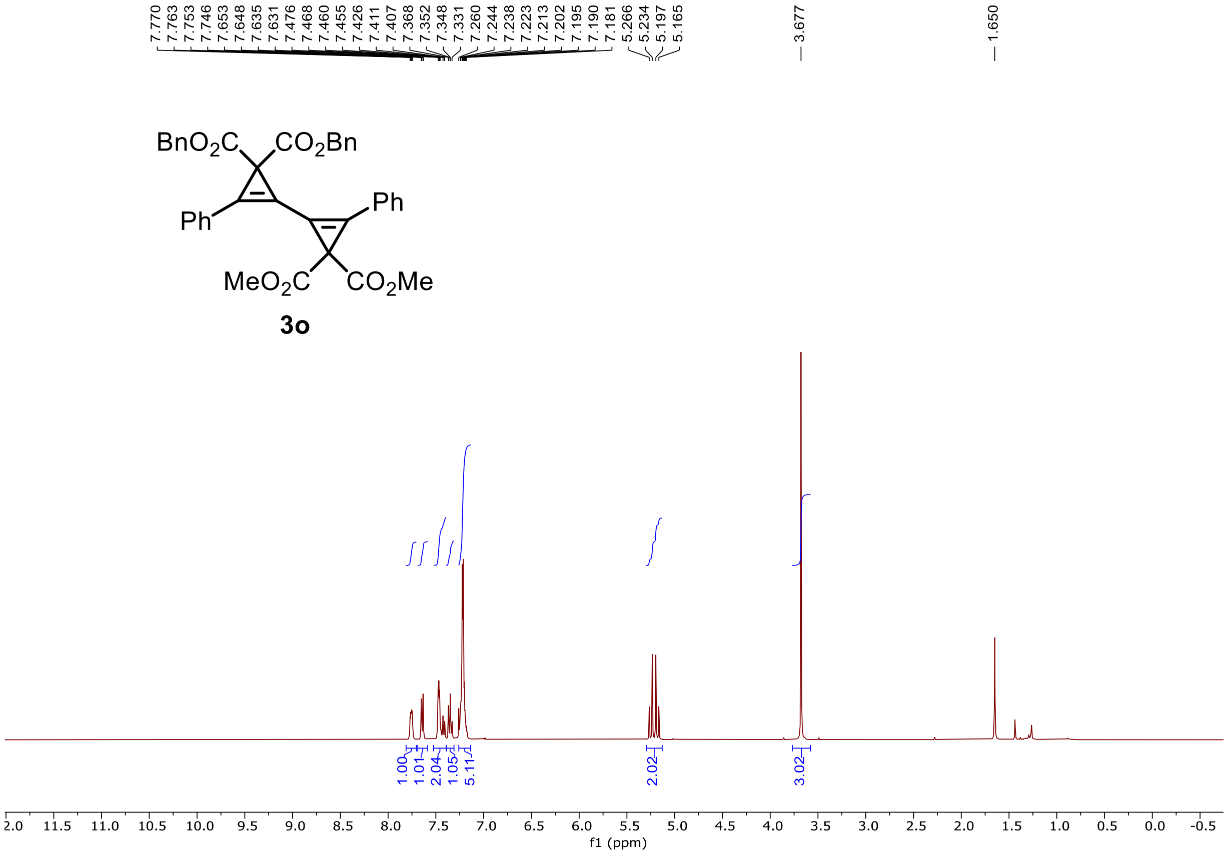

¹³C NMR (101 MHz, $CDCl_3$) of 3o

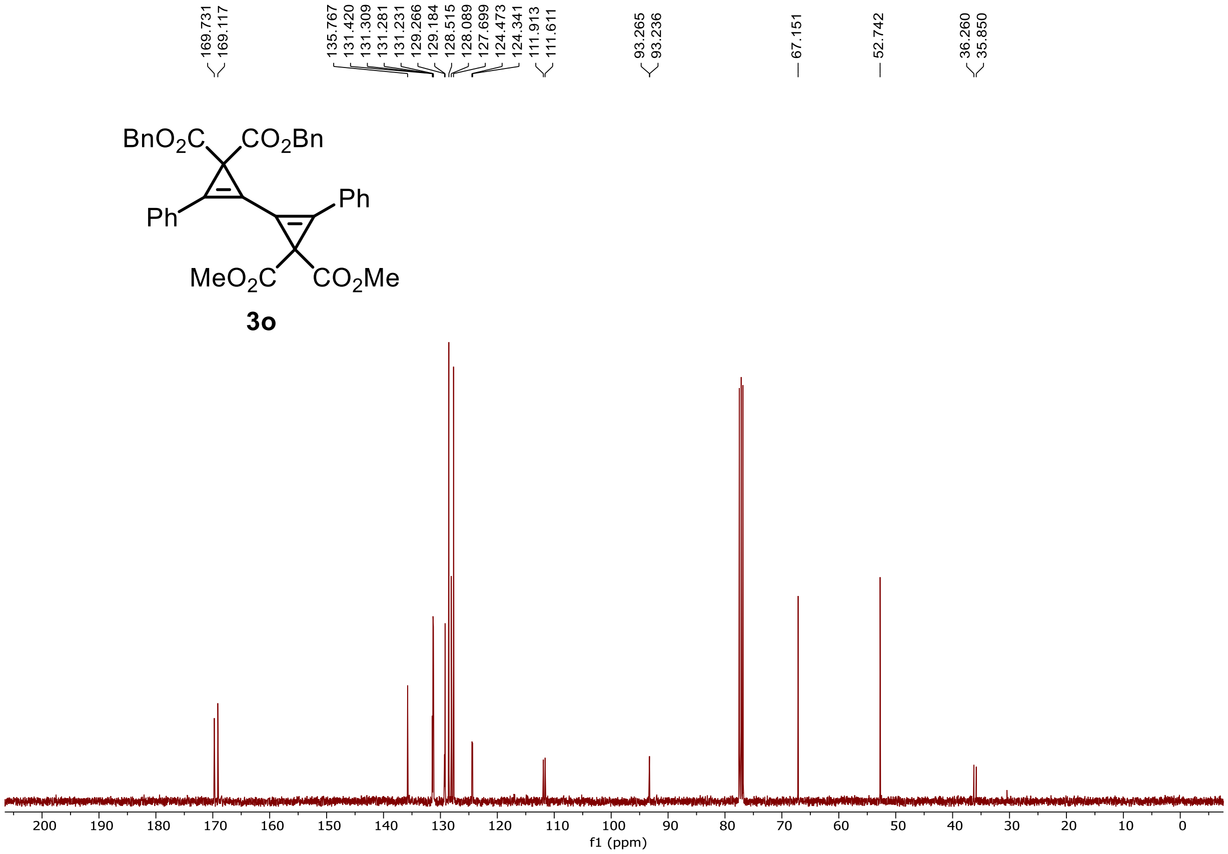

^{1}H NMR (400 MHz, $CDCl_3$) of 3p

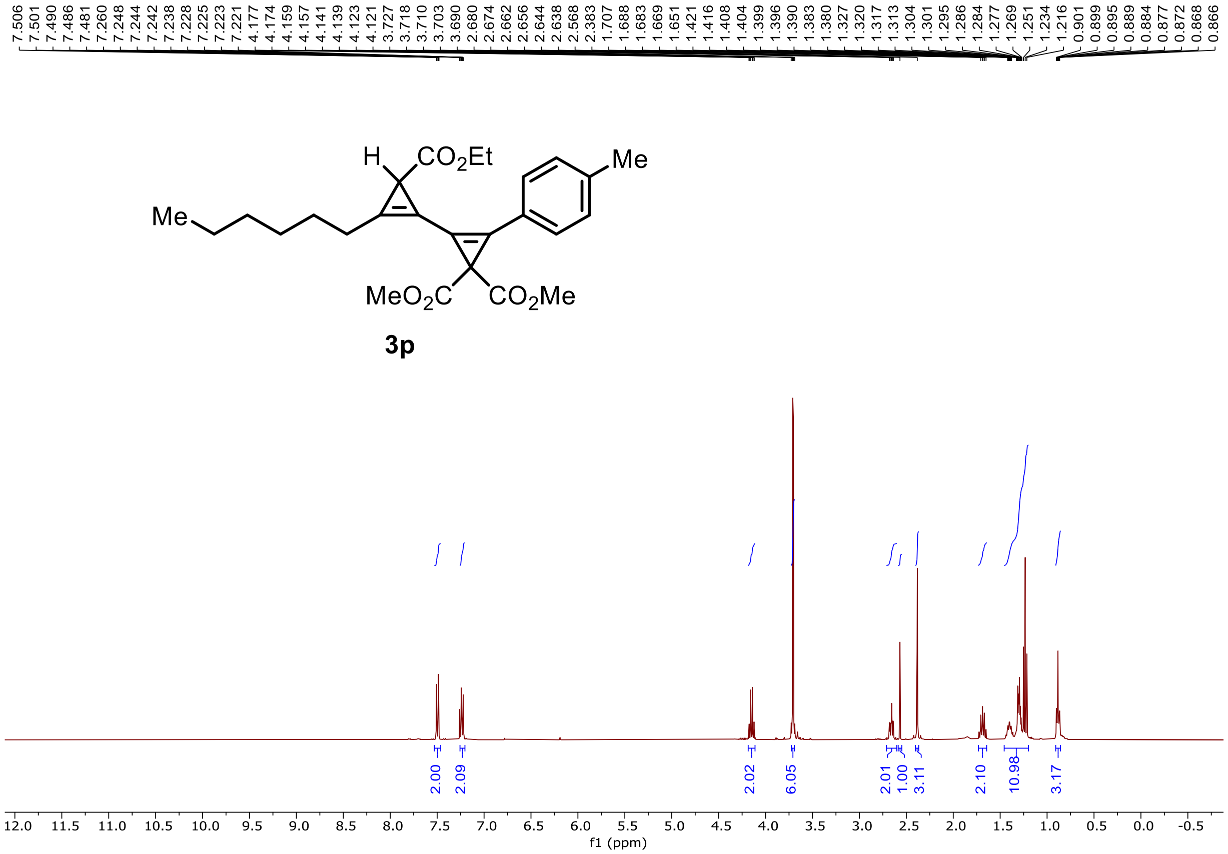

^{13}C NMR (101 MHz, $CDCl_3$) of 3p

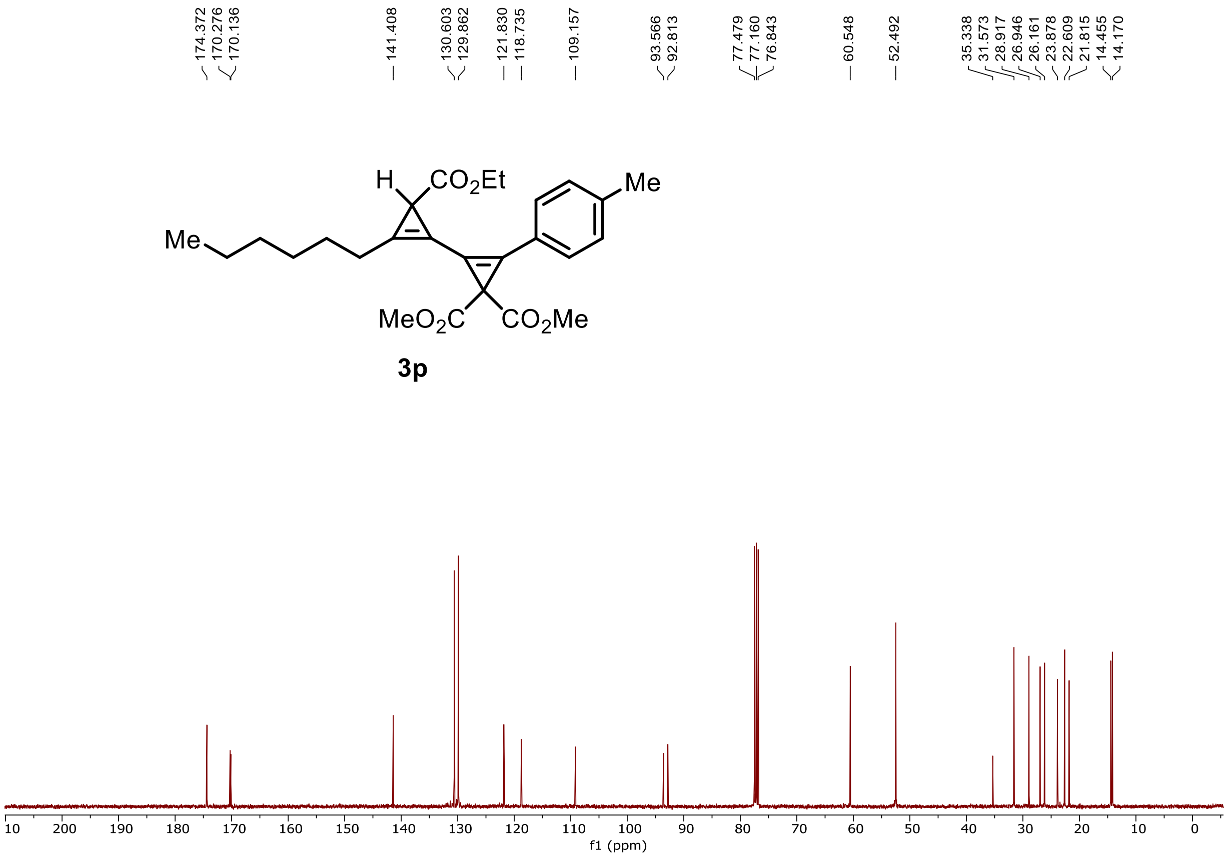

^{1}H NMR (400 MHz, $CDCl_3$) of 3q

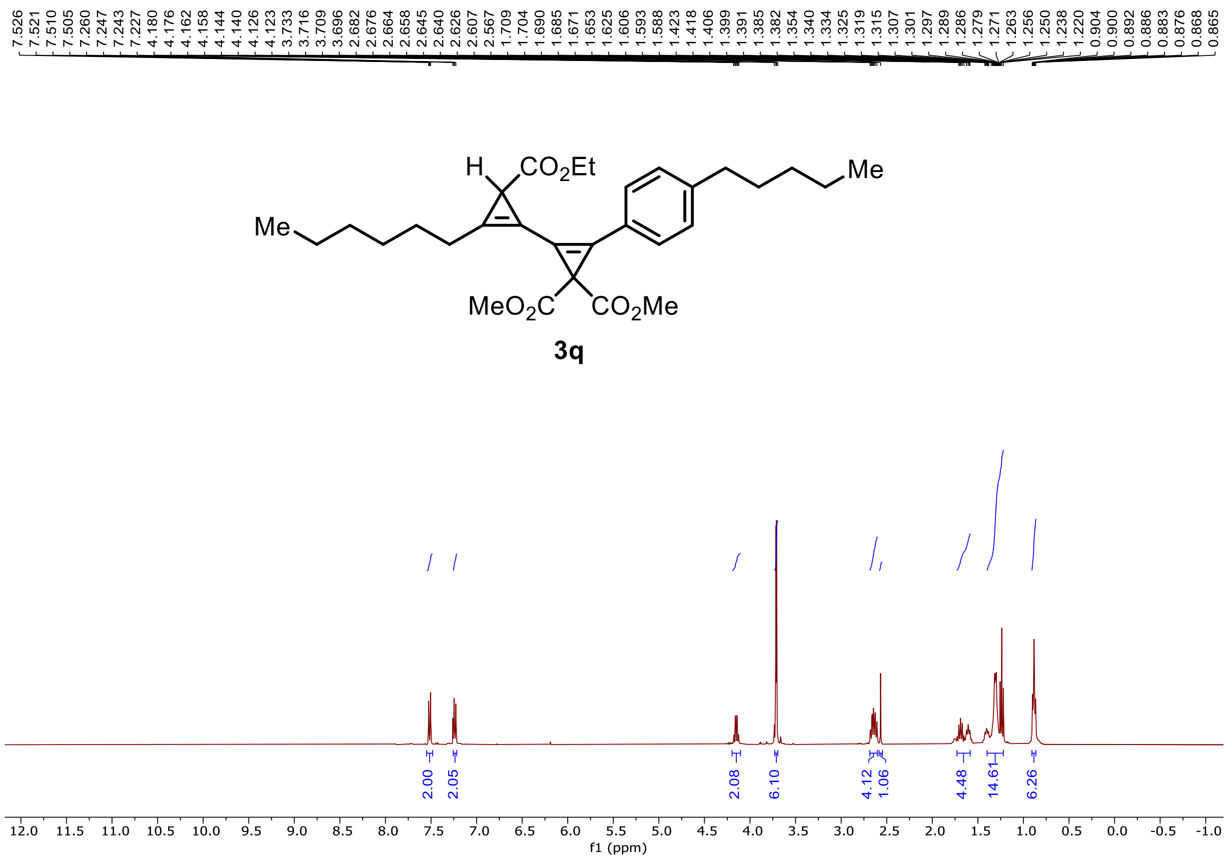

^{13}C NMR (101 MHz, $CDCl_3$) of 3q

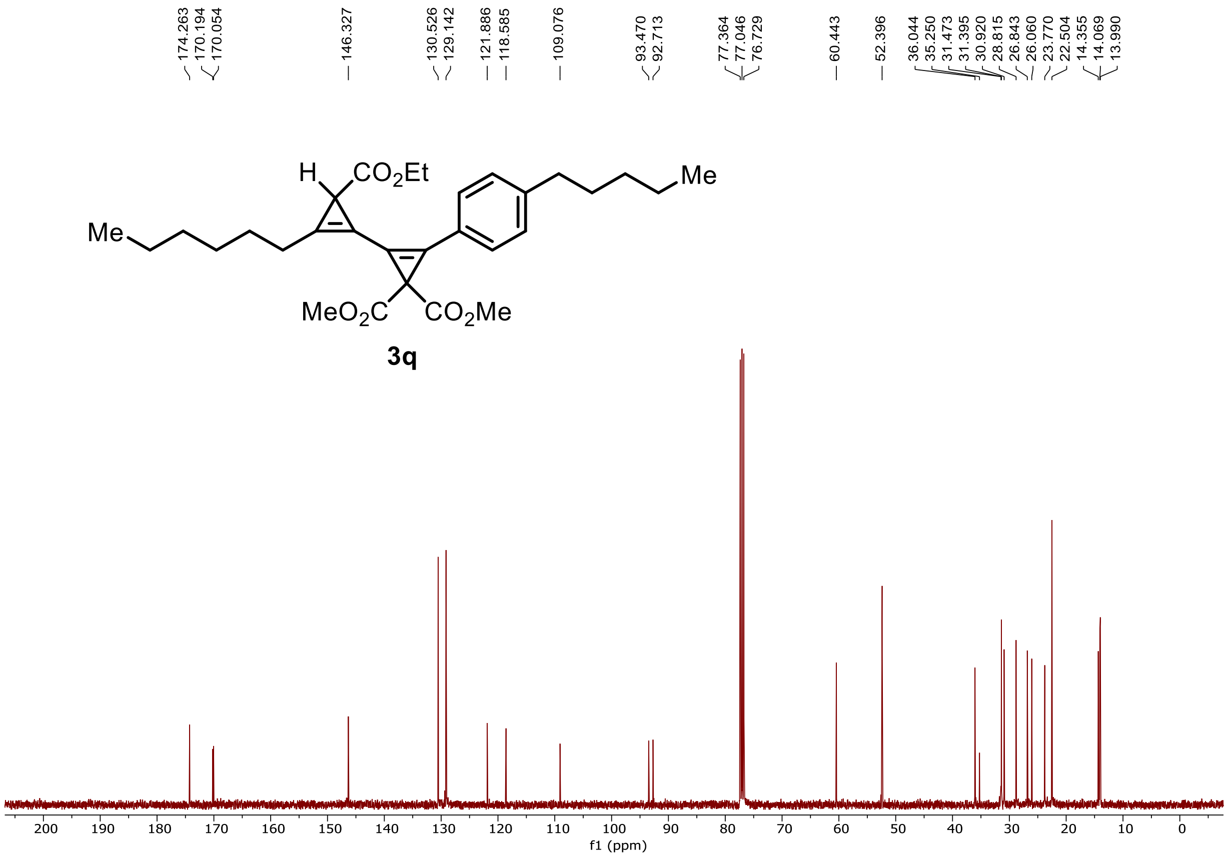

^1H NMR (400 MHz, $CDCl_3$) of 3r

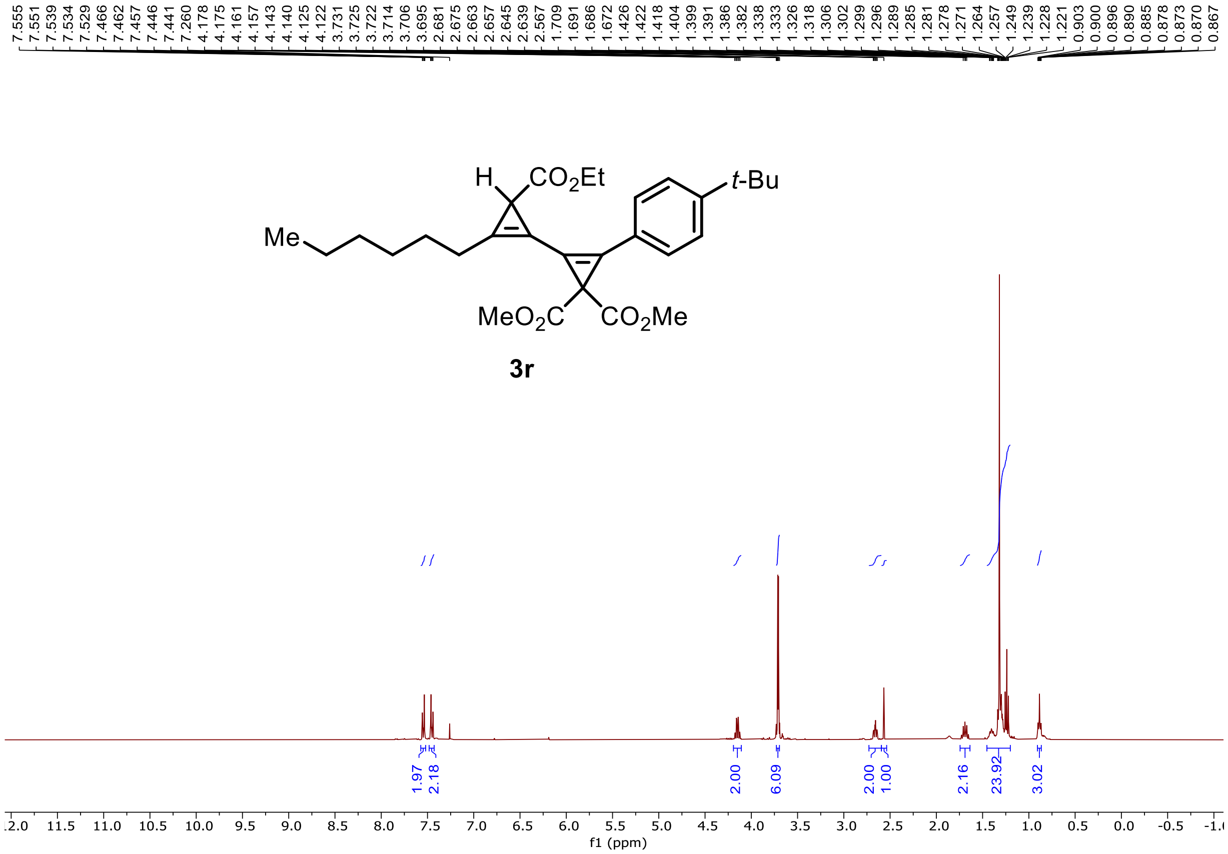

^{13}C NMR (101 MHz, $CDCl_3$) of 3r

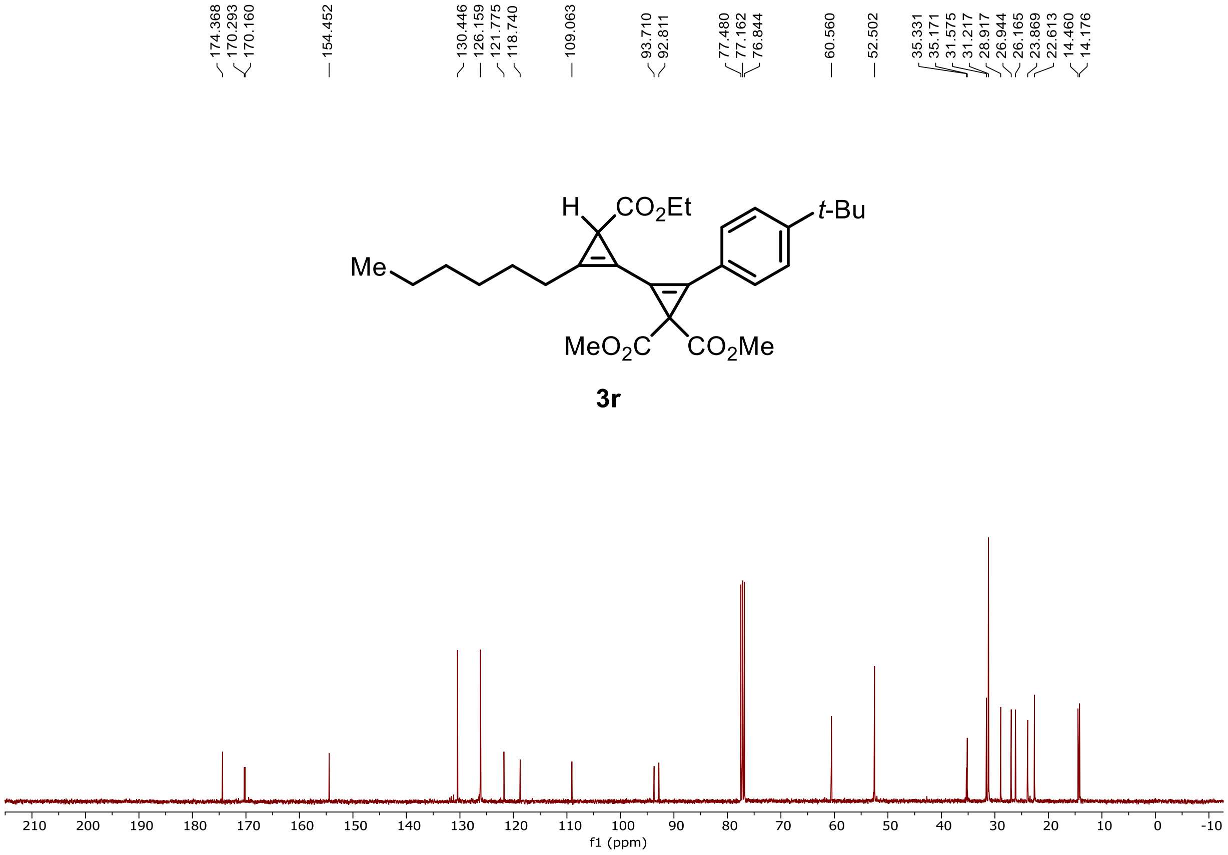

^{1}H NMR (400 MHz, $CDCl_3$) of 3s

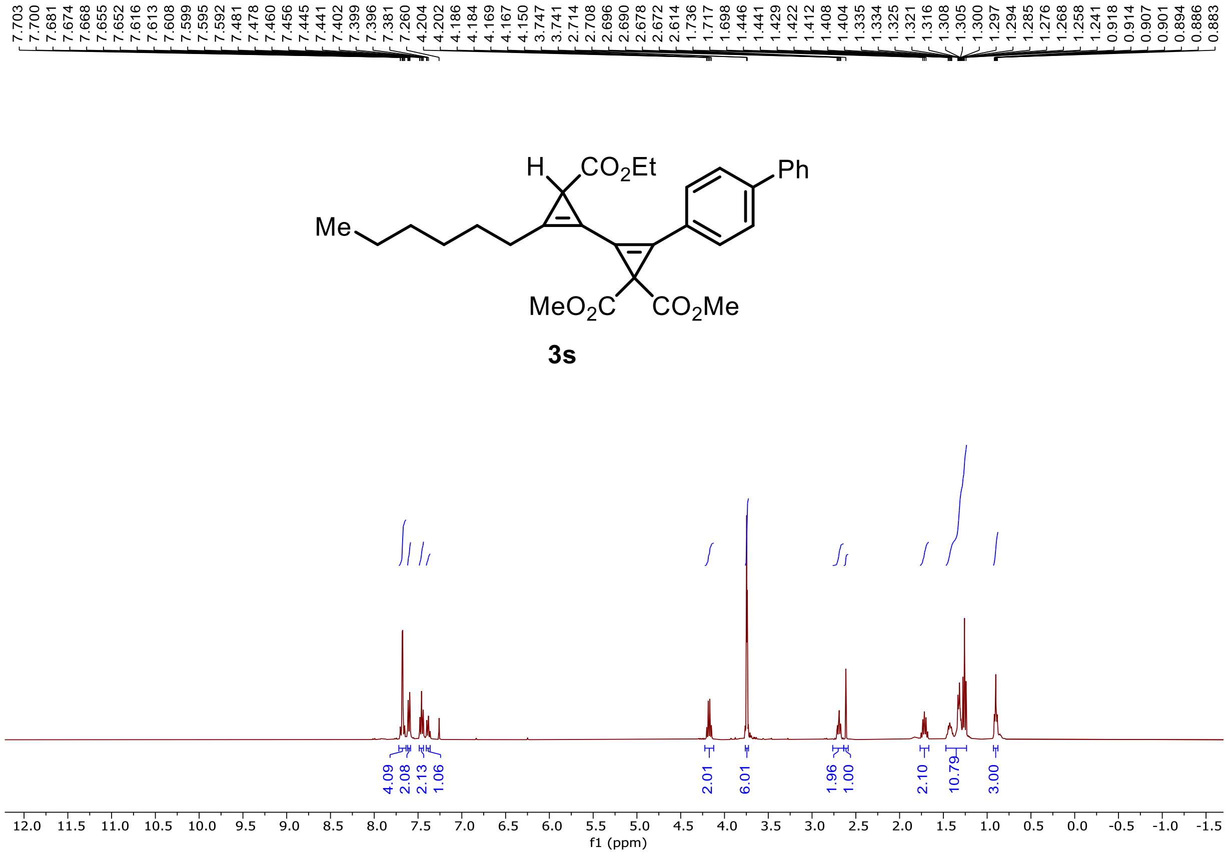

^{13}C NMR (101 MHz, $CDCl_3$) of 3s

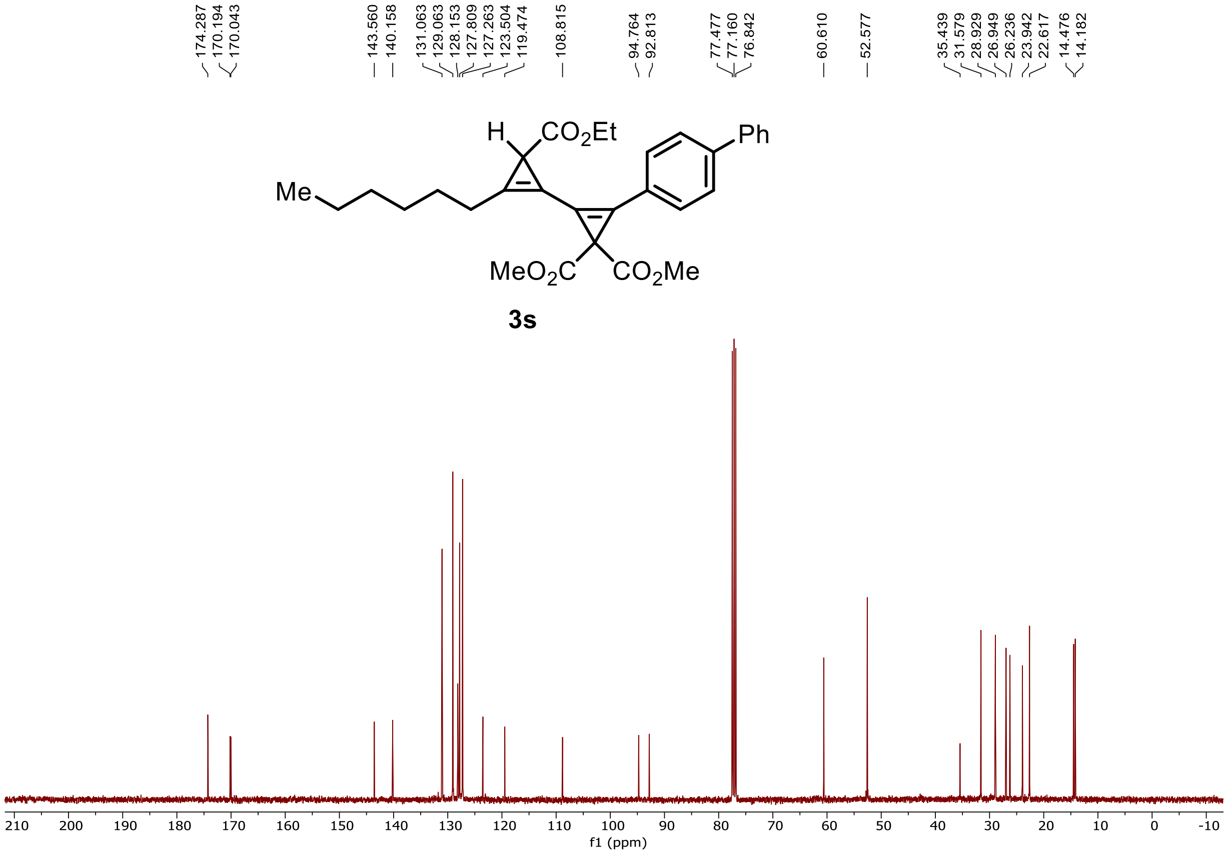

^{1}H NMR (400 MHz, $CDCl_3$) of 3t

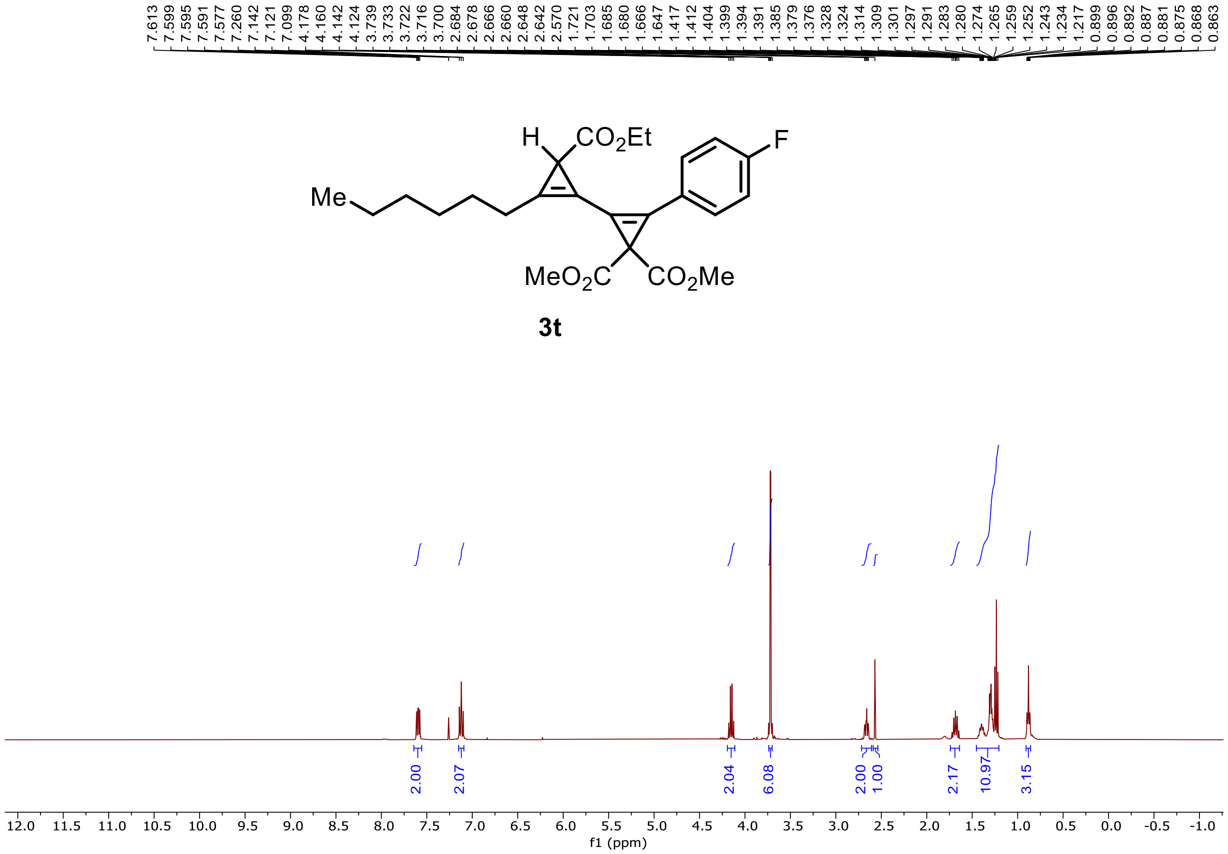

^{13}C NMR (101 MHz, $CDCl_3$) of 3t

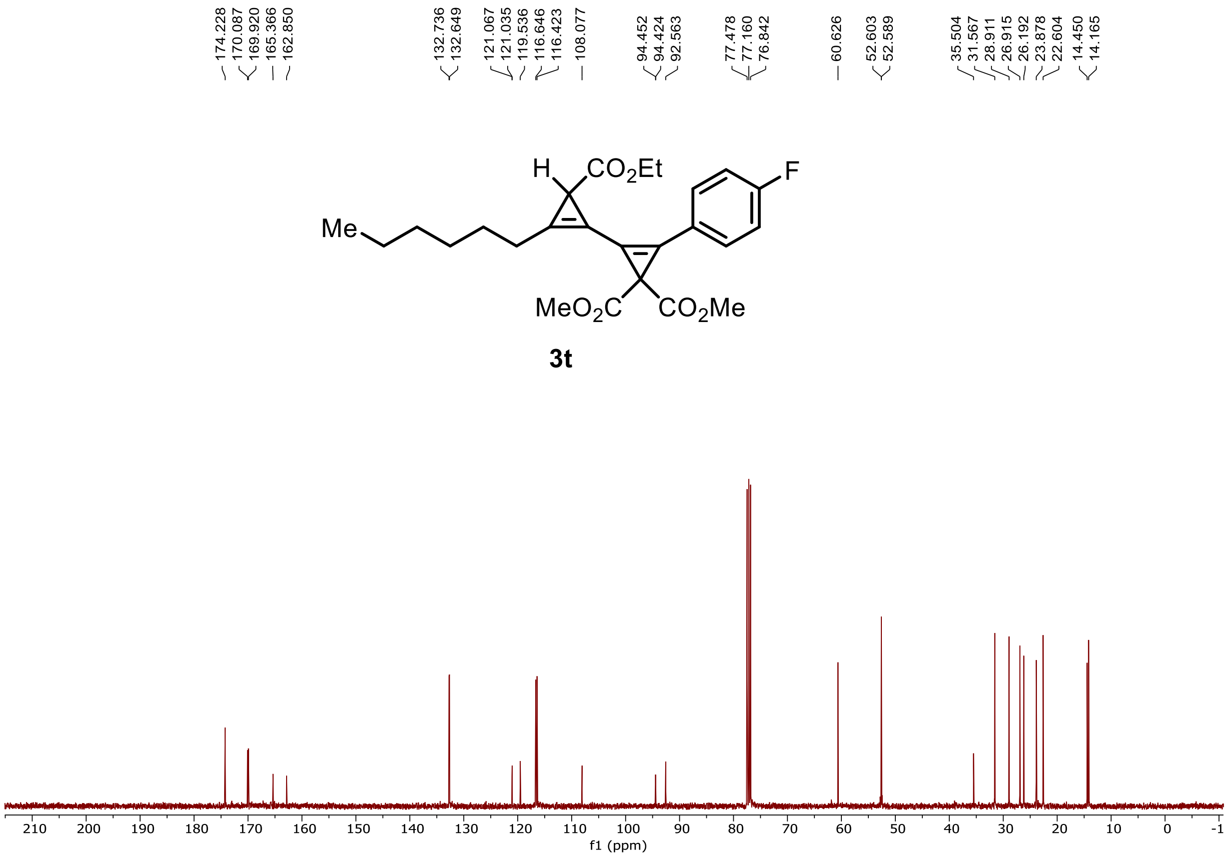

^{19}F NMR (377 MHz, $CDCl_3$) of 3t

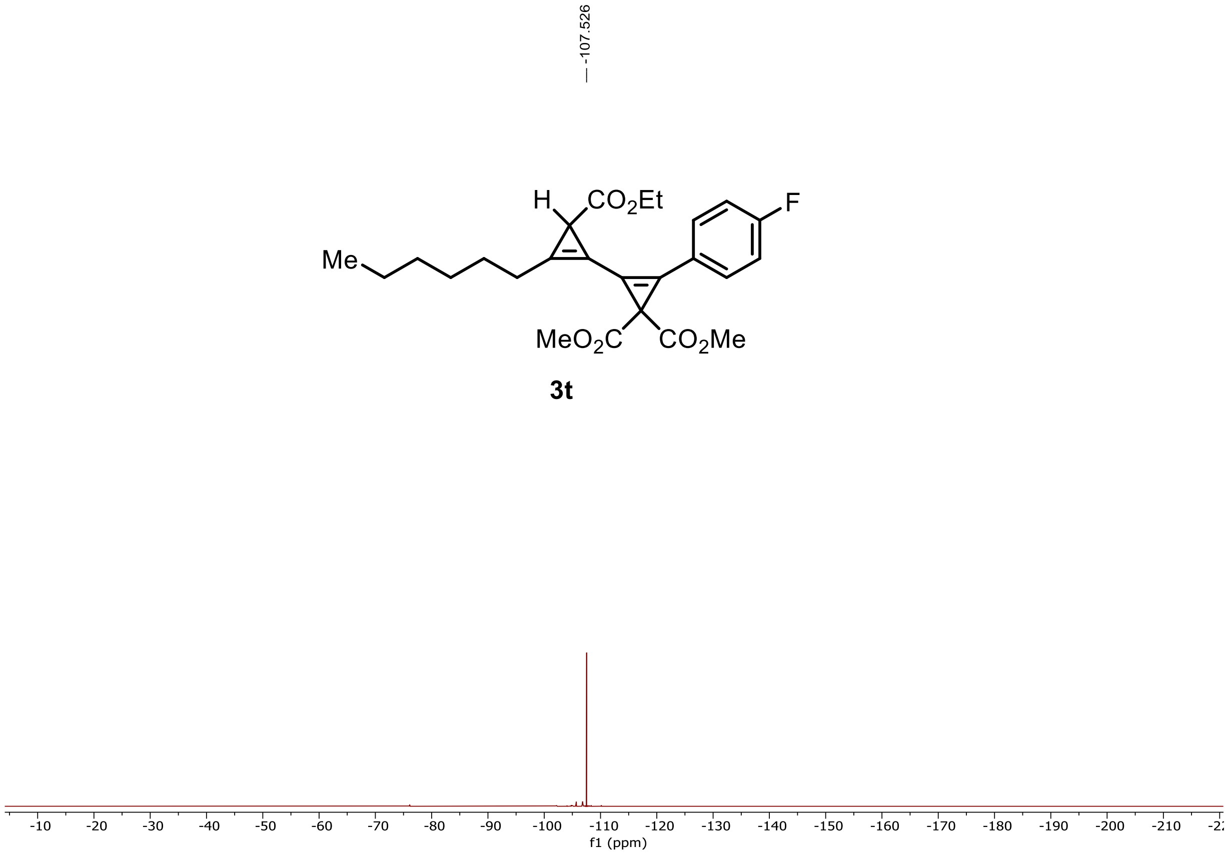

^{1}H NMR (400 MHz, $CDCl_3$) of 3u

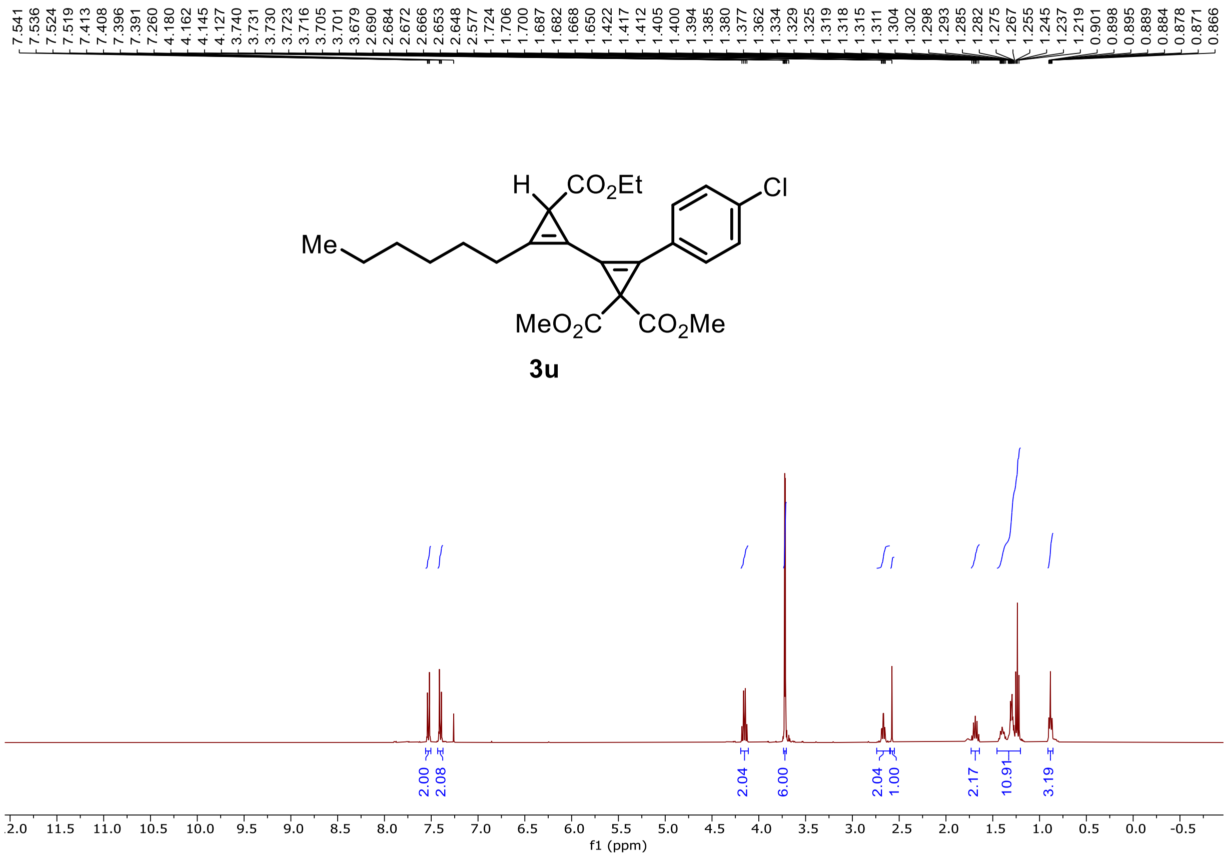

^{13}C NMR (101 MHz, $CDCl_3$) of 3u

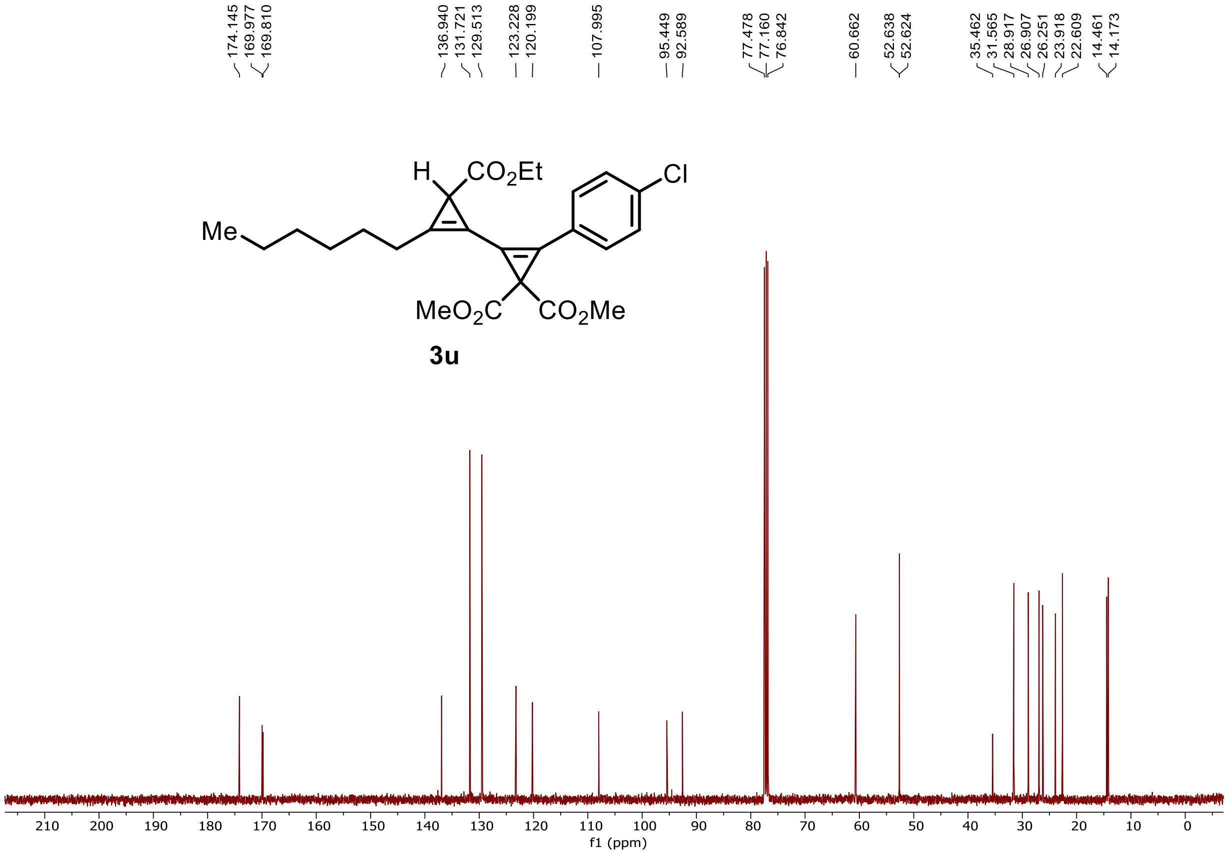

^{1}H NMR (400 MHz, $CDCl_3$) of 3v

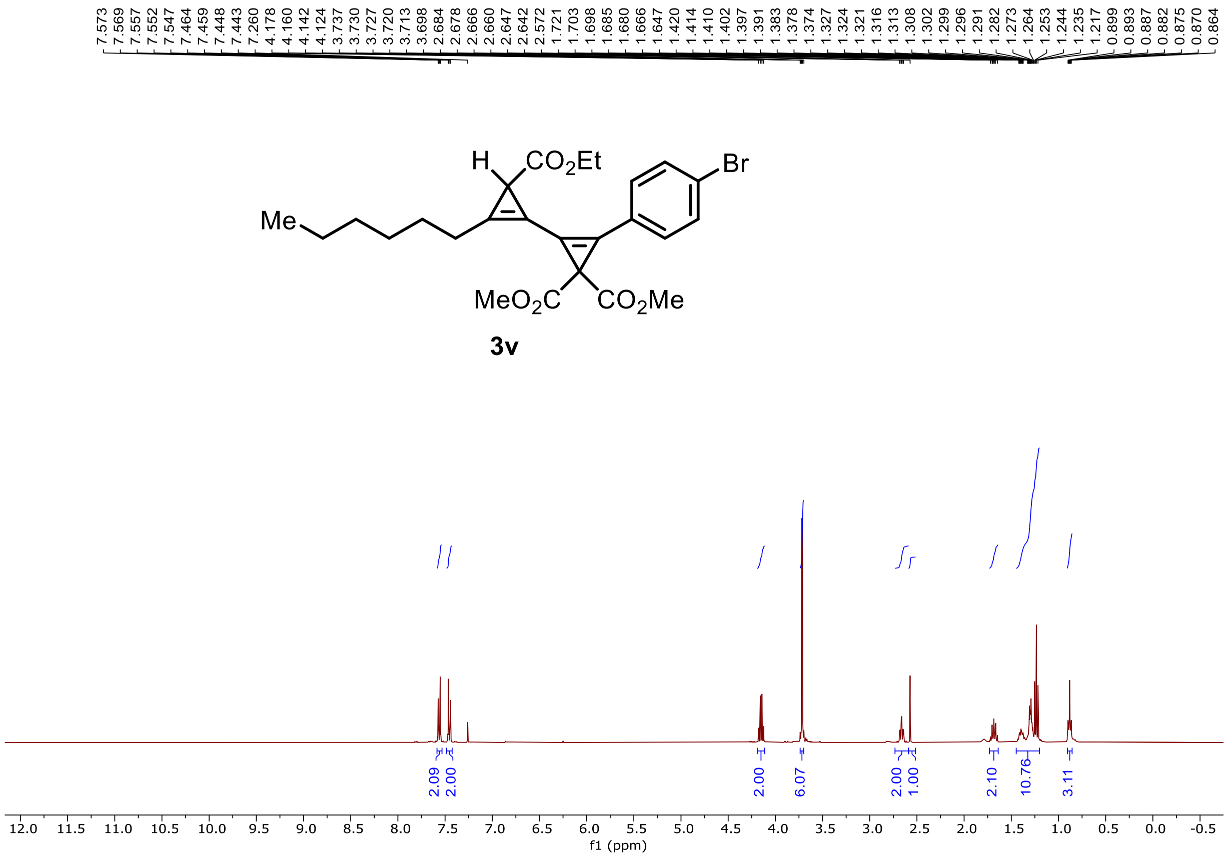

^{13}C NMR (101 MHz, $CDCl_3$) of 3v

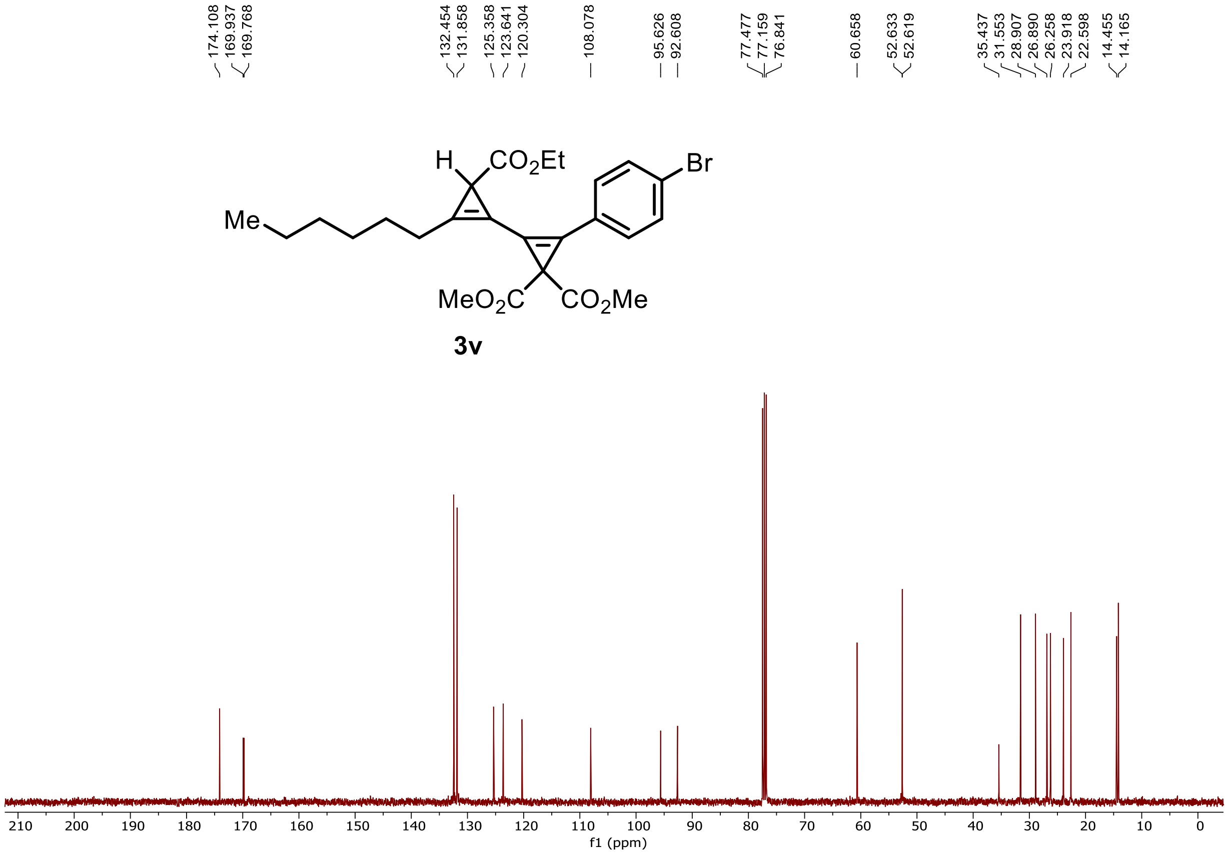

^{1}H NMR (400 MHz, $CDCl_3$) of 3w

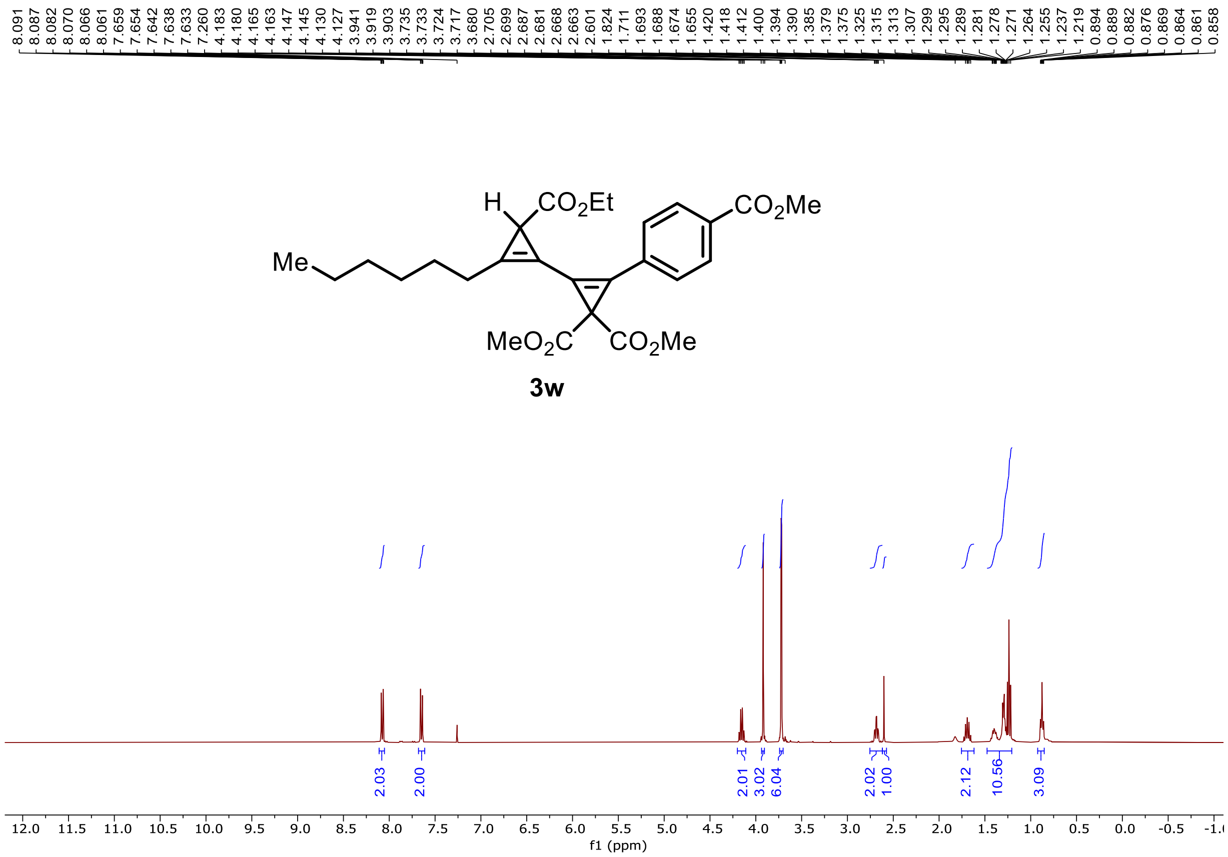

^{13}C NMR (101 MHz, CDCl$_3$) of 3w

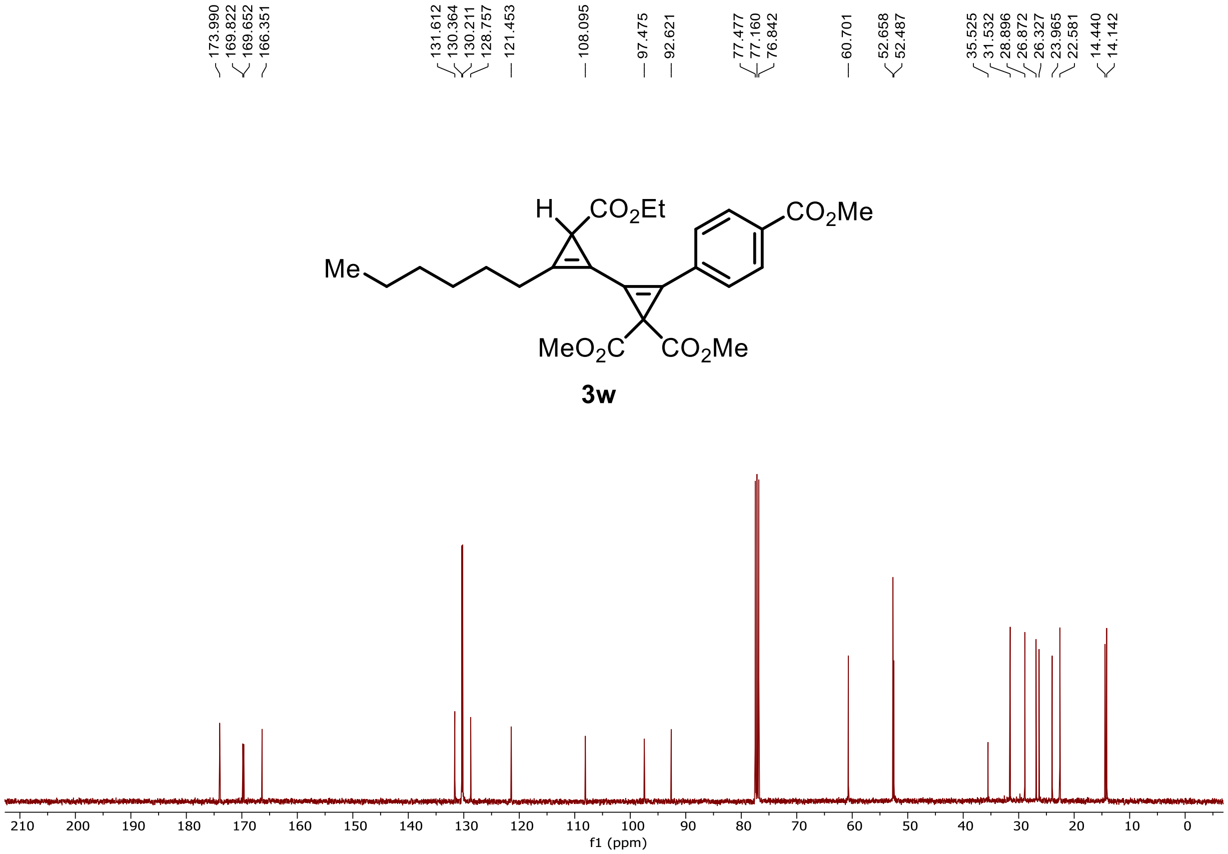

^{1}H NMR (400 MHz, CDCl$_3$) of 3x

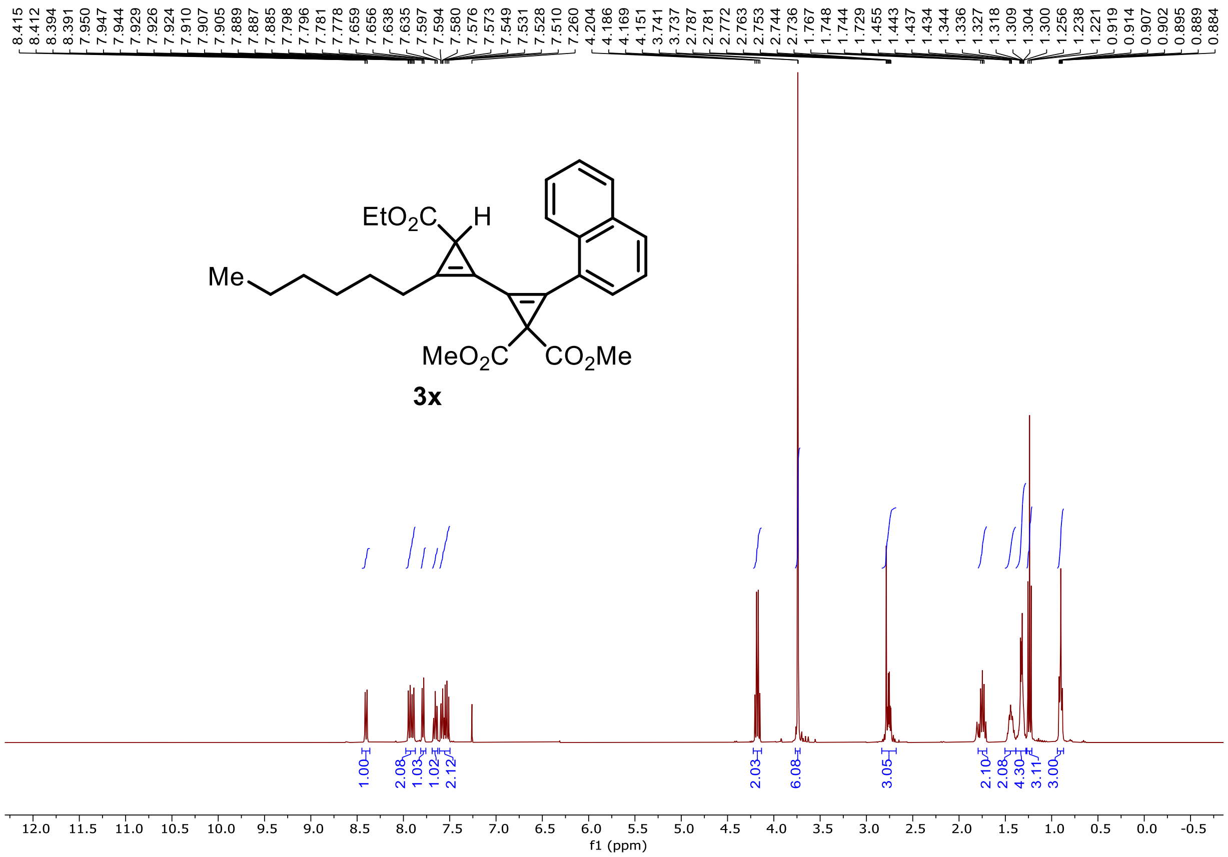

^{13}C NMR (101 MHz, $CDCl_3$) of 3x

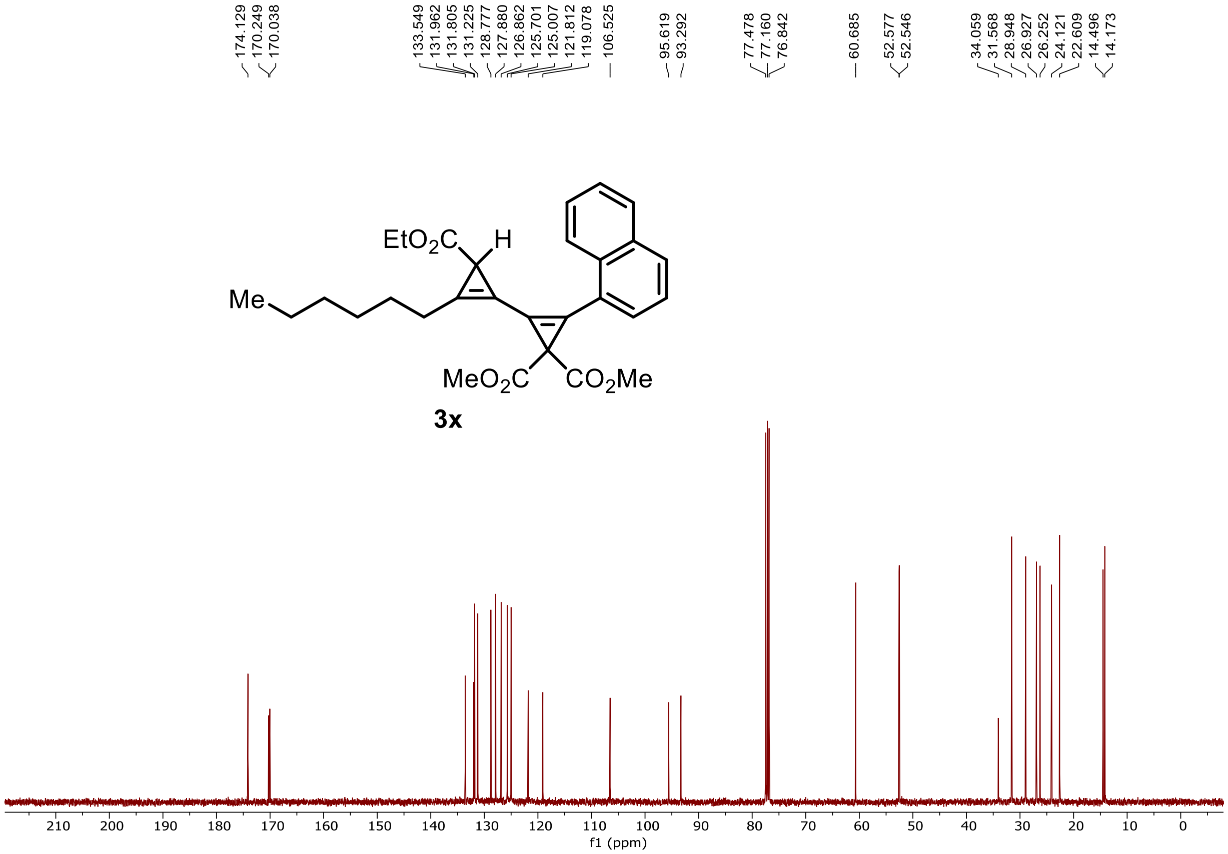

^{1}H NMR (400 MHz, $CDCl_3$) of 3y

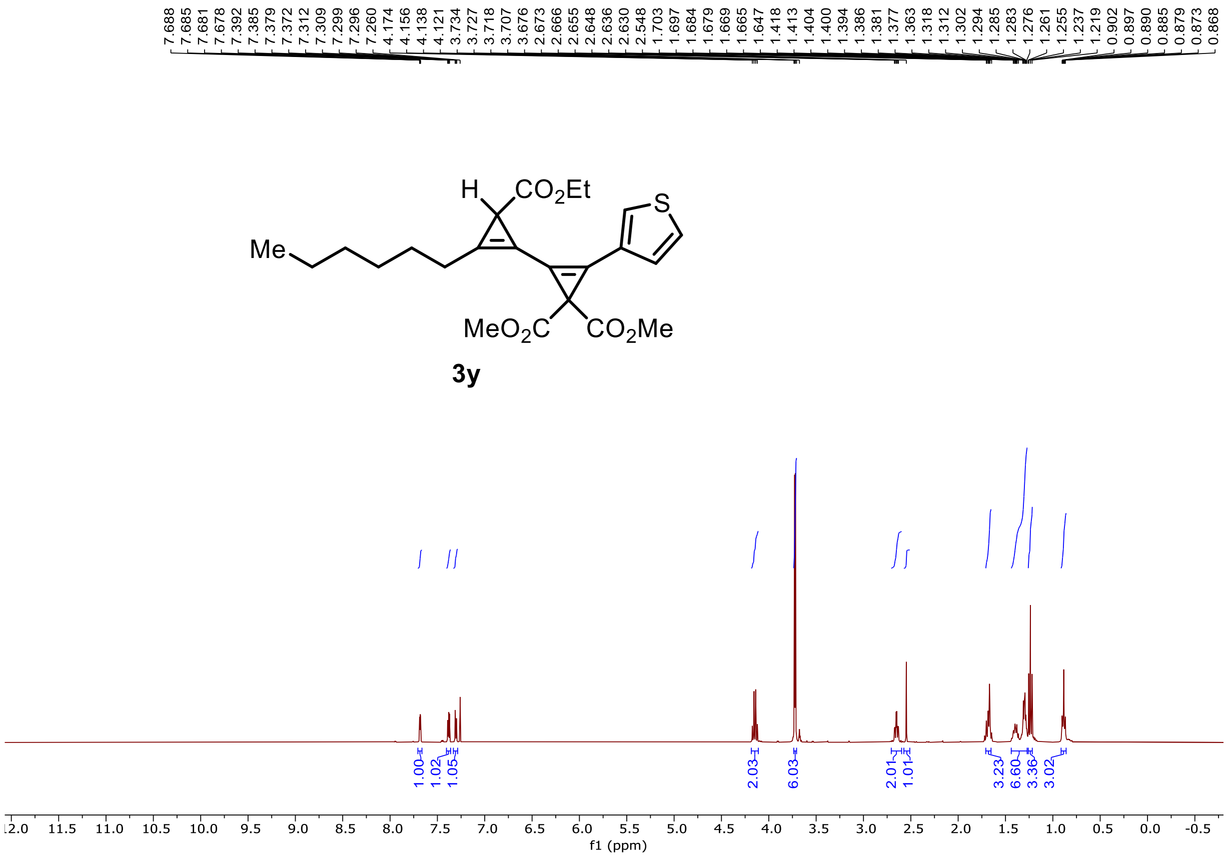

^{13}C NMR (101 MHz, $CDCl_3$) of 3y

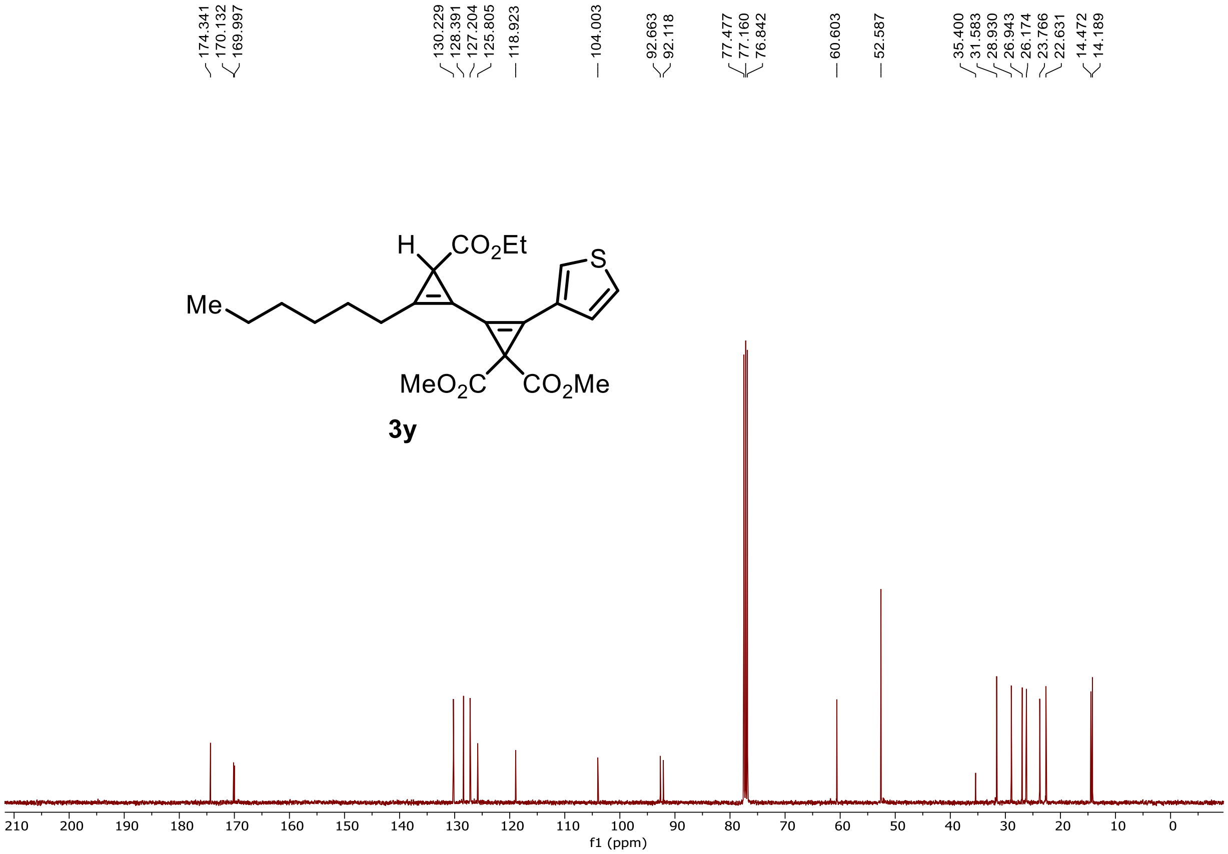

^{1}H NMR (400 MHz, $CDCl_3$) of 3z

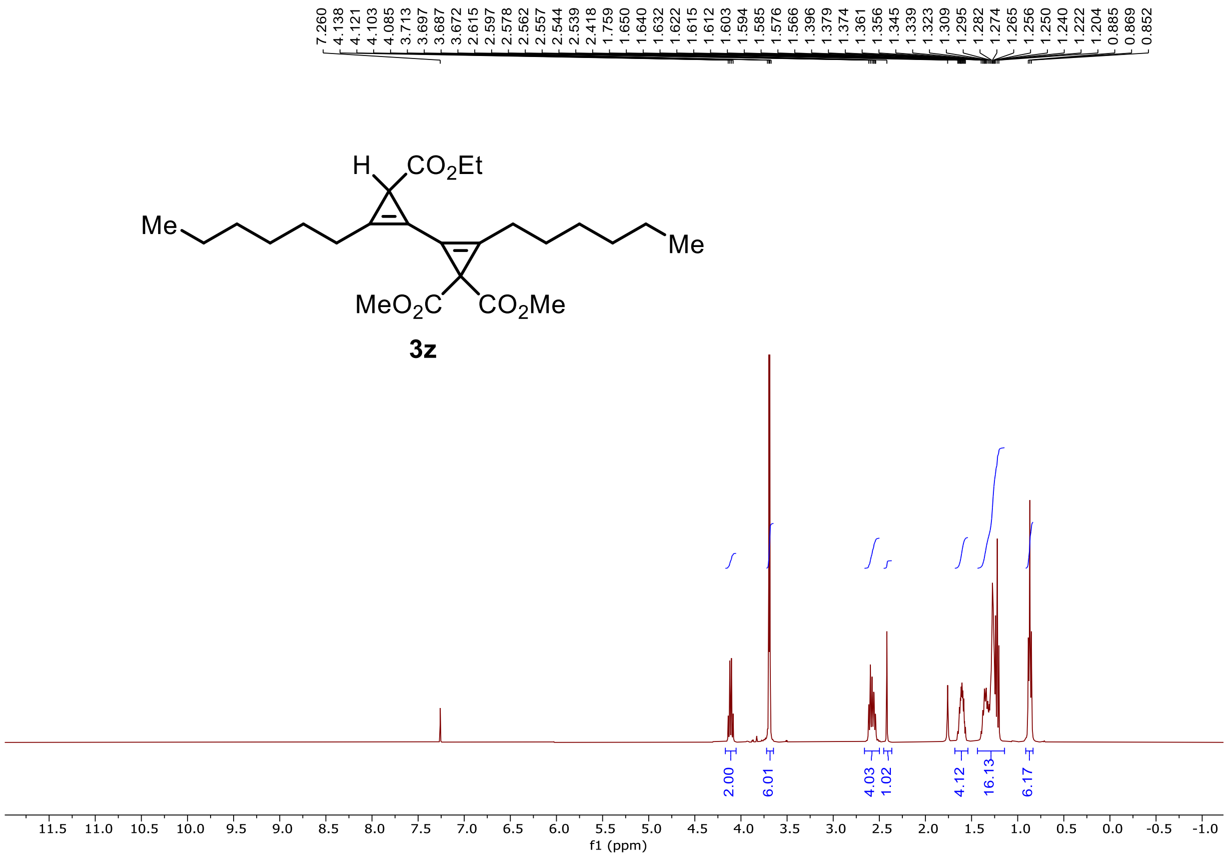

^{13}C NMR (101 MHz, $CDCl_3$) of 3z

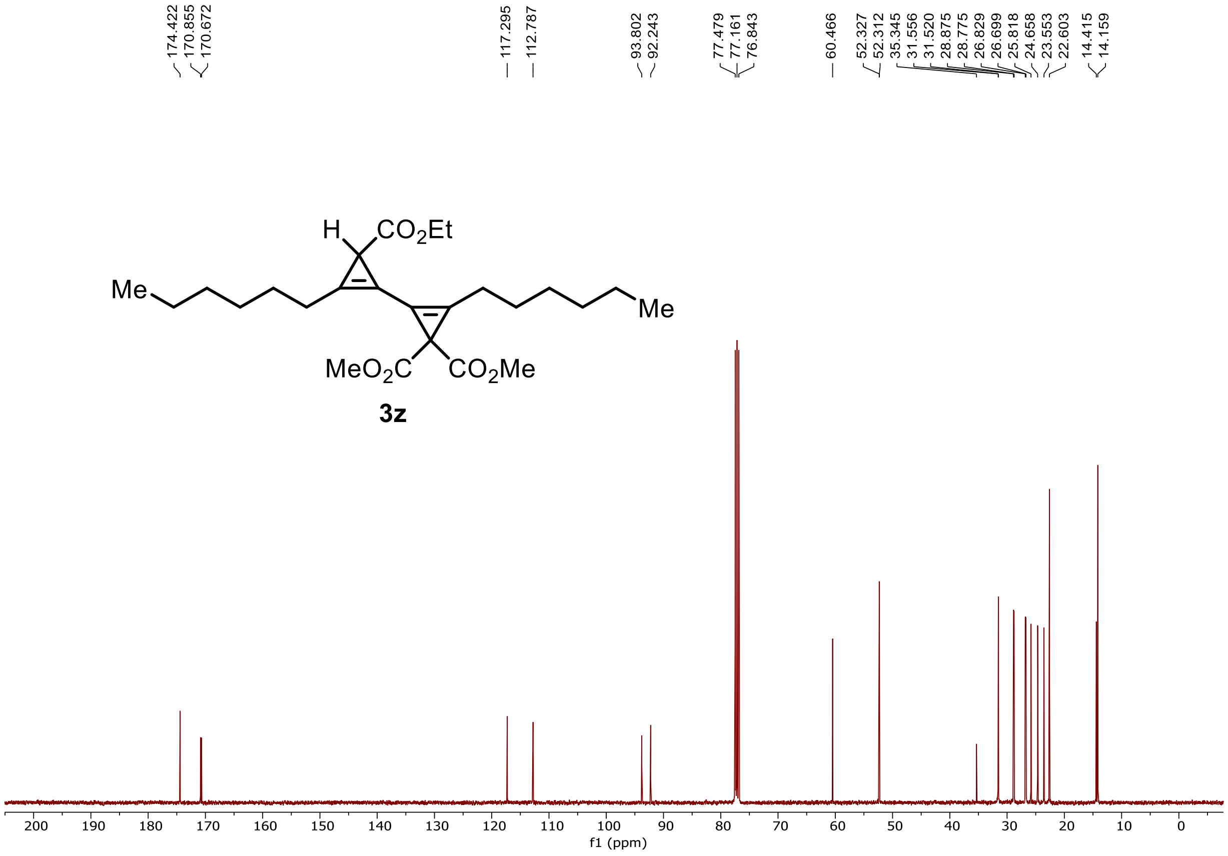

^{1}H NMR (400 MHz, $CDCl_3$) of 3aa

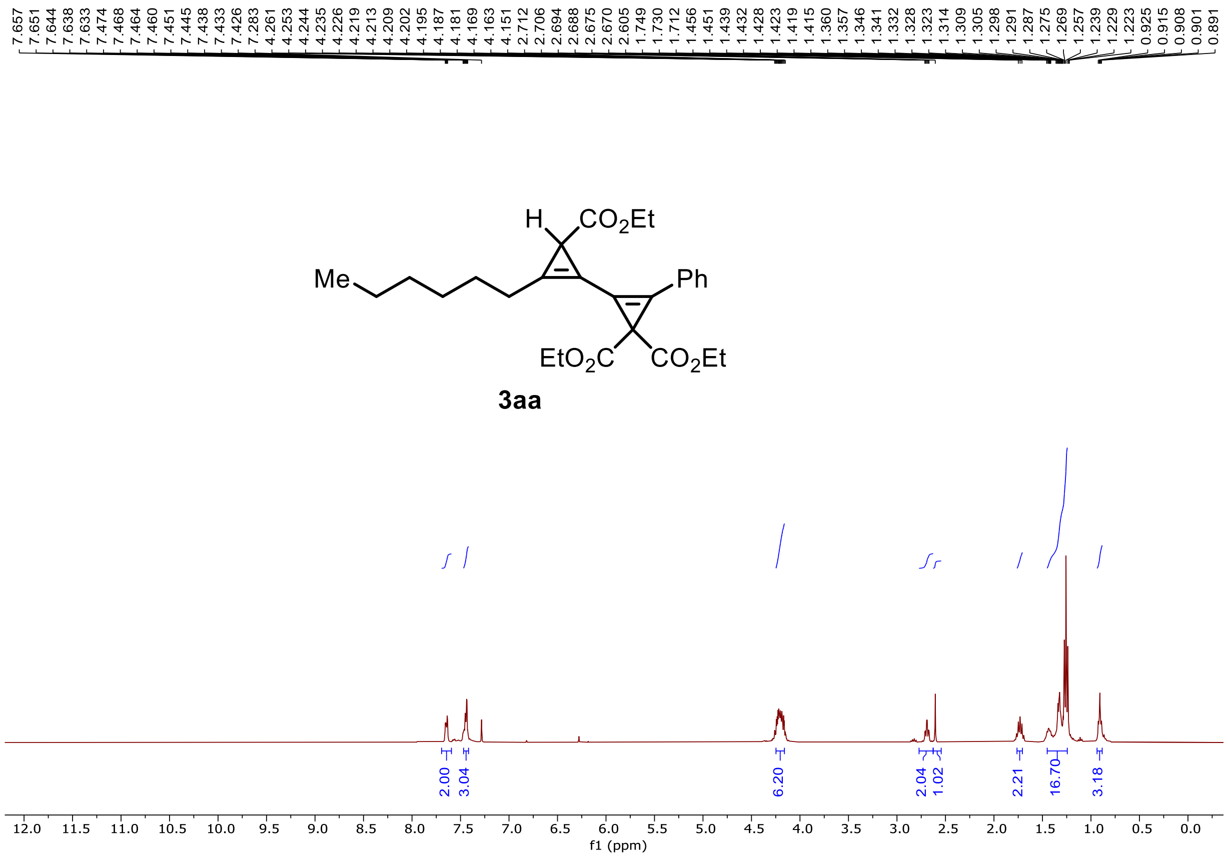

^{13}C NMR (101 MHz, $CDCl_3$) of 3aa

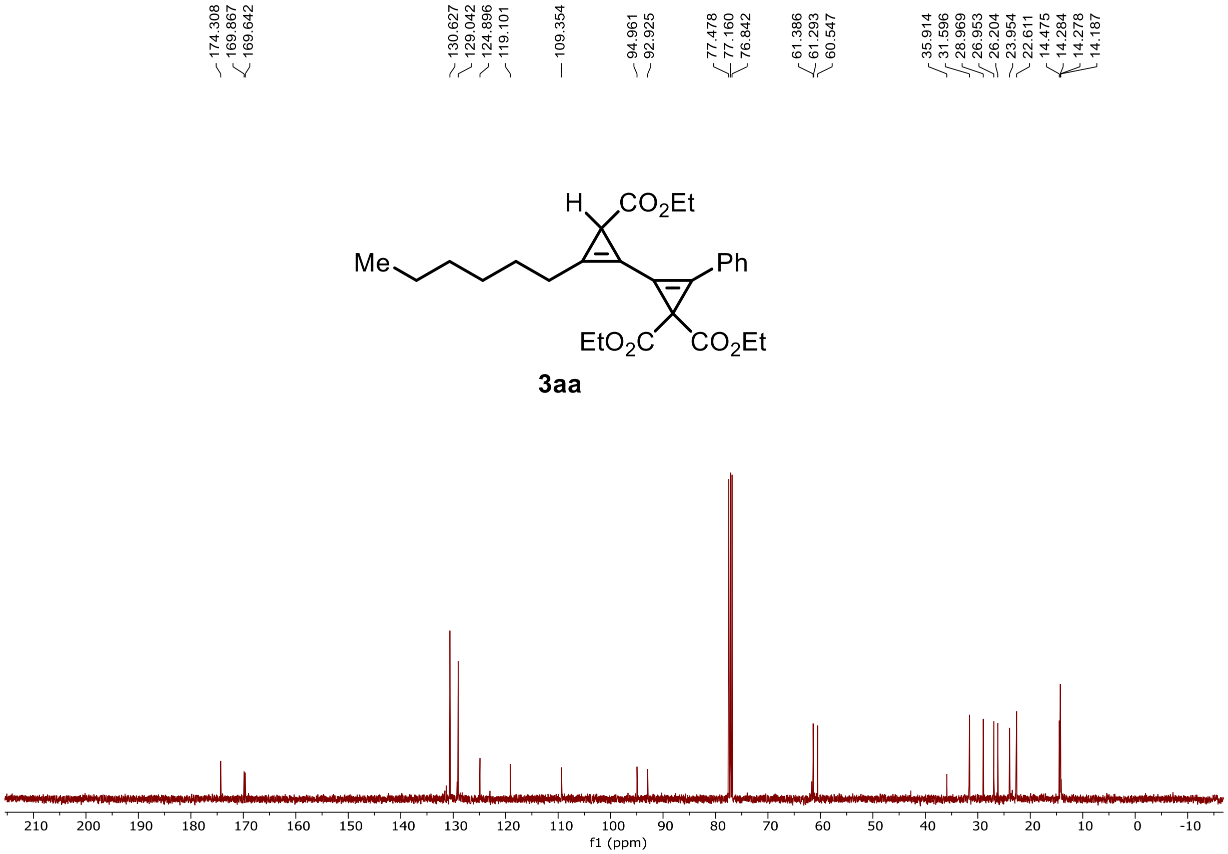

^{1}H NMR (400 MHz, $CDCl_3$) of 3ab

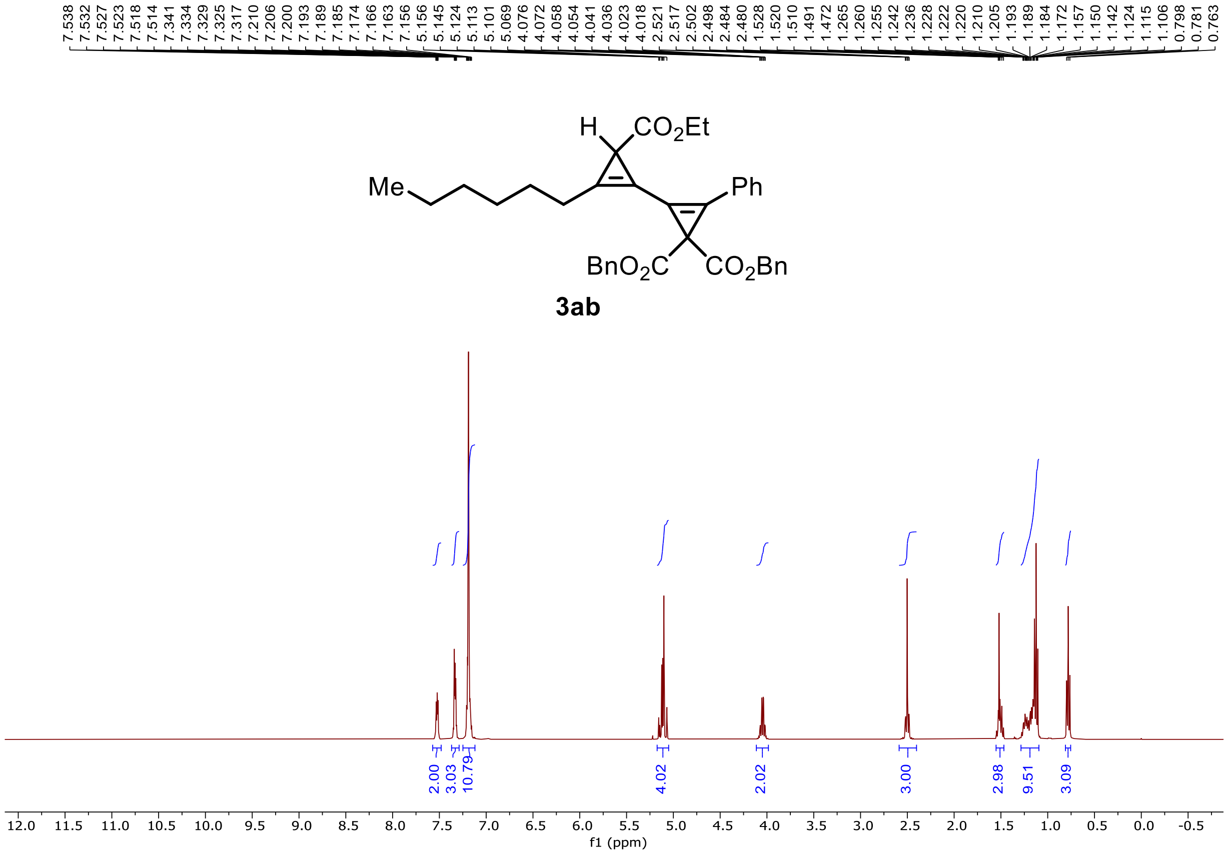

^{13}C NMR (101 MHz, $CDCl_3$) of 3ab

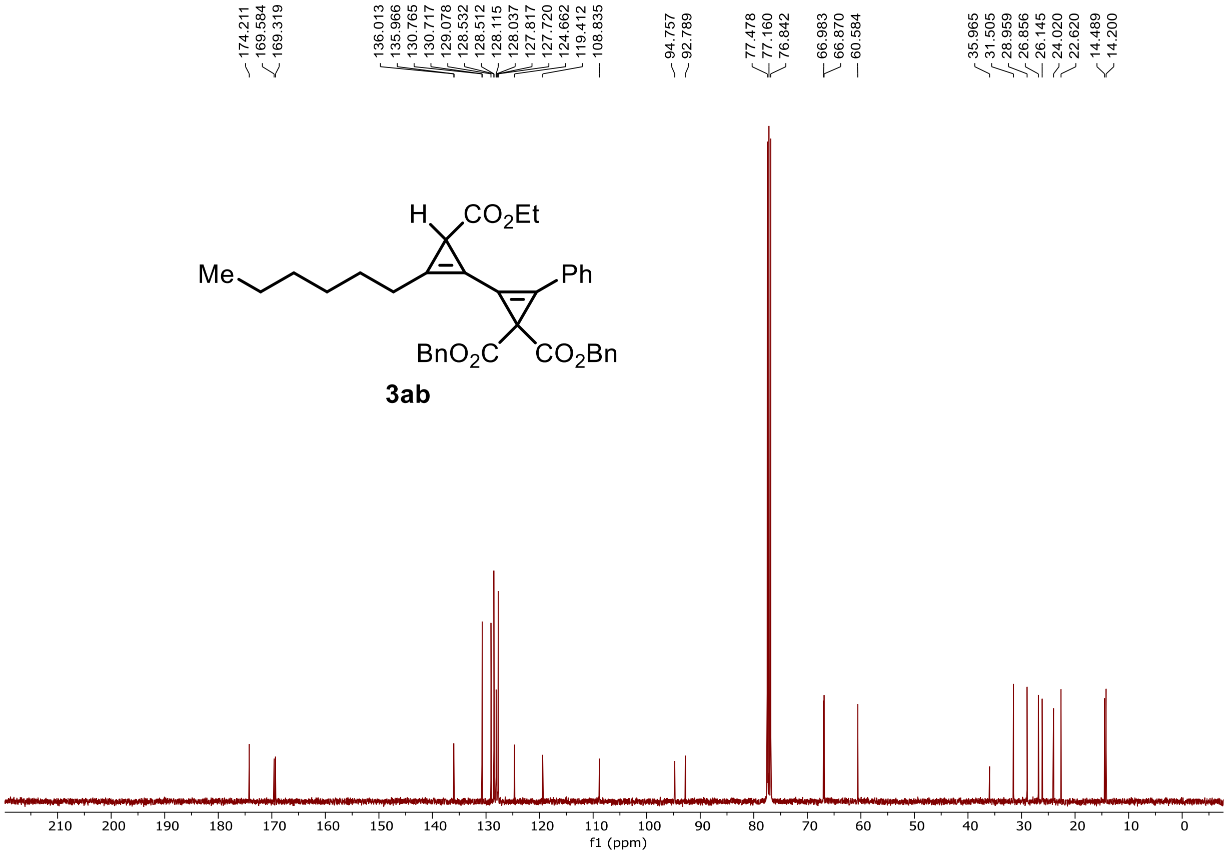

^{1}H NMR (400 MHz, $CDCl_3$) of 3ac

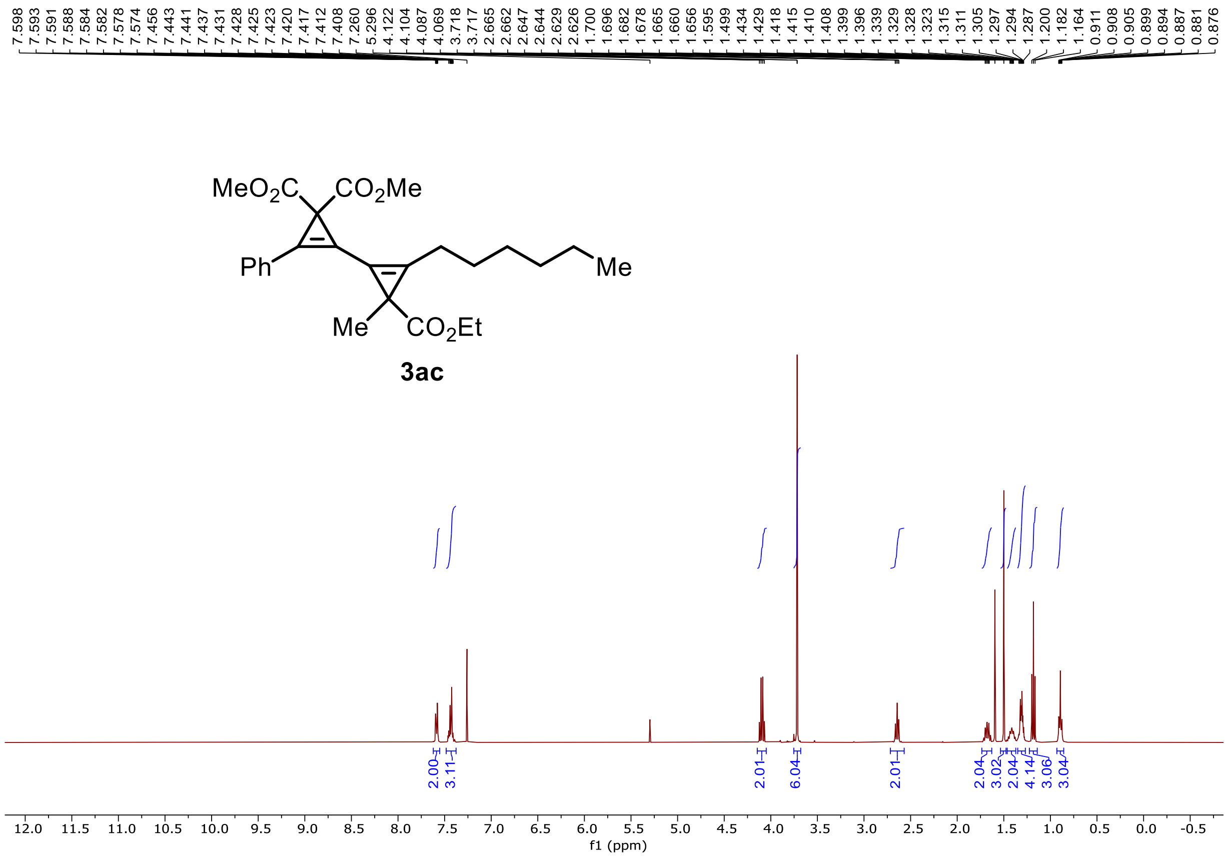

^{13}C NMR (101 MHz, CDCl$_3$) of 3ac

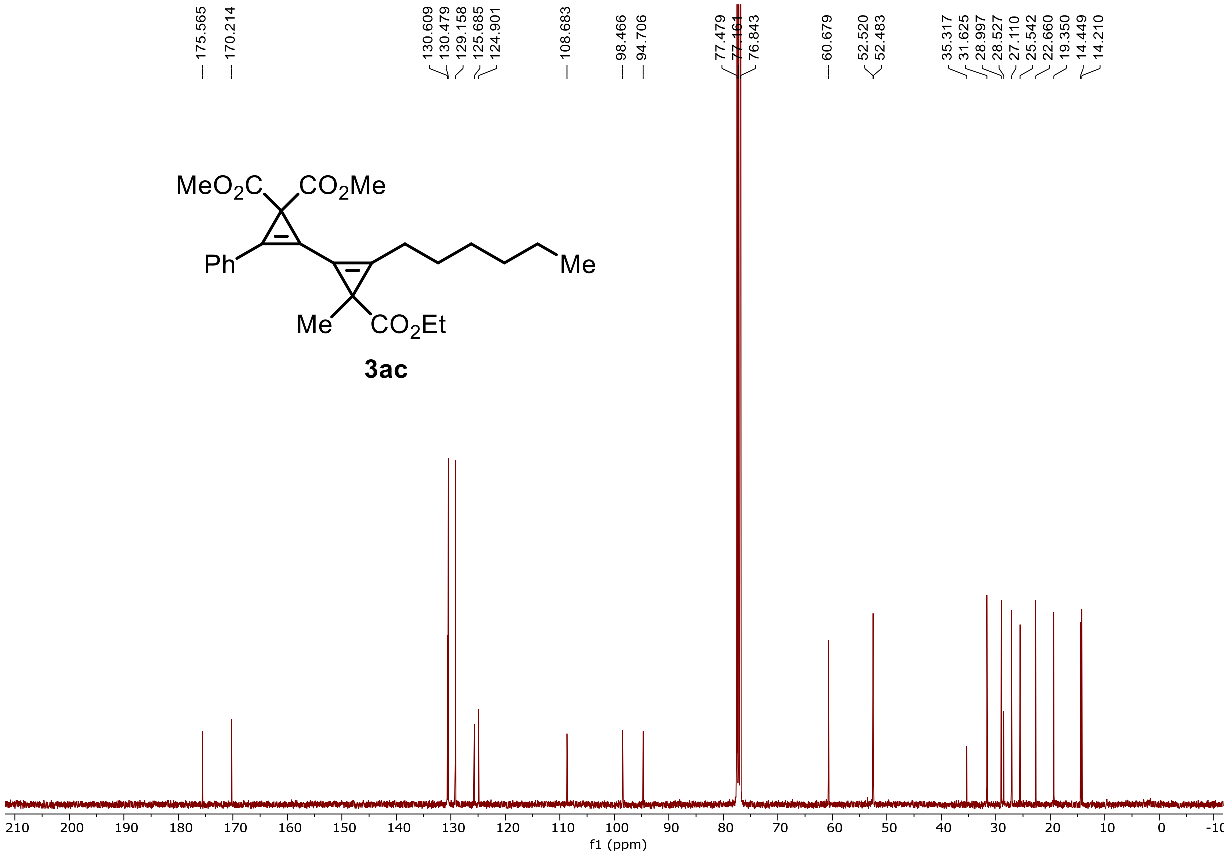

^{1}H NMR (400 MHz, CDCl$_3$) of 5

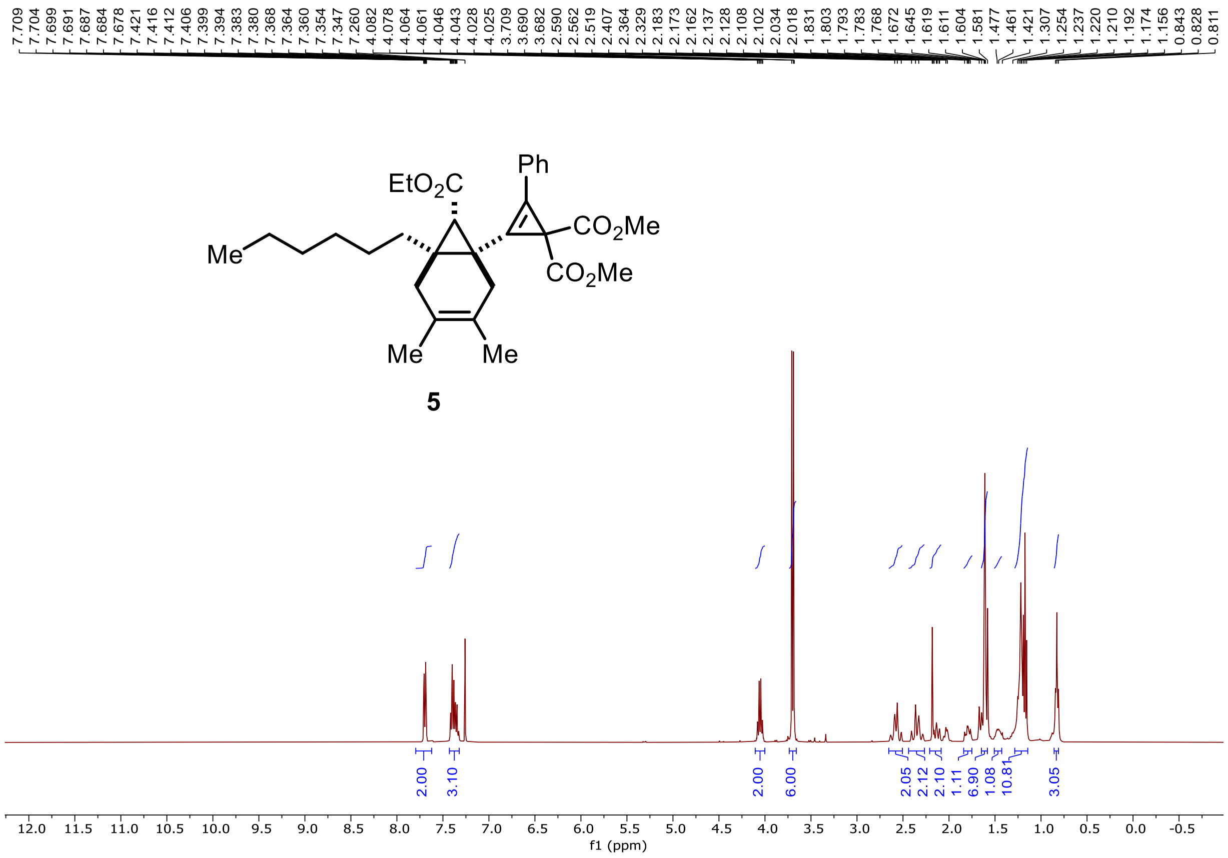

^{13}C NMR (101 MHz, $CDCl_3$) of 5

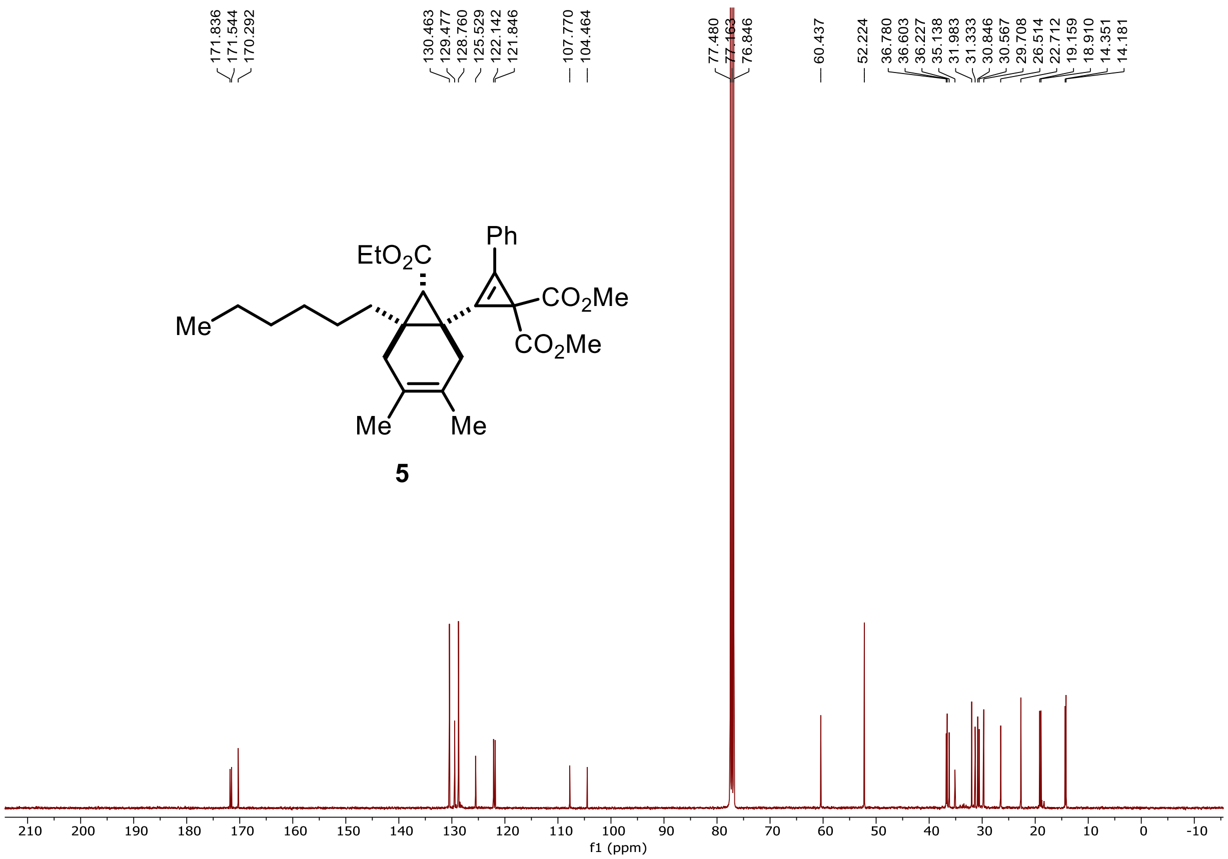

^{1}H-^{13}C HMBC NMR of 5

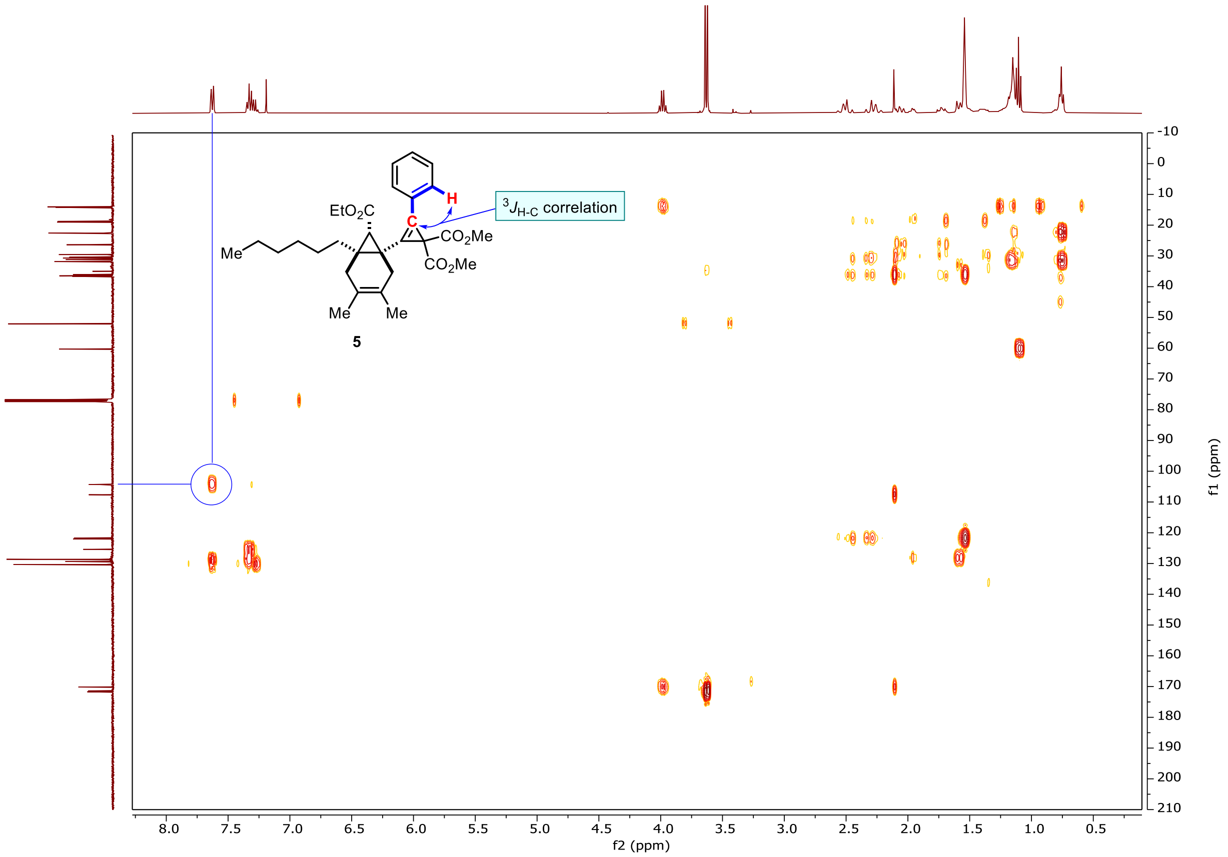

¹H NMR (400 MHz, $CDCl_3$) of 6

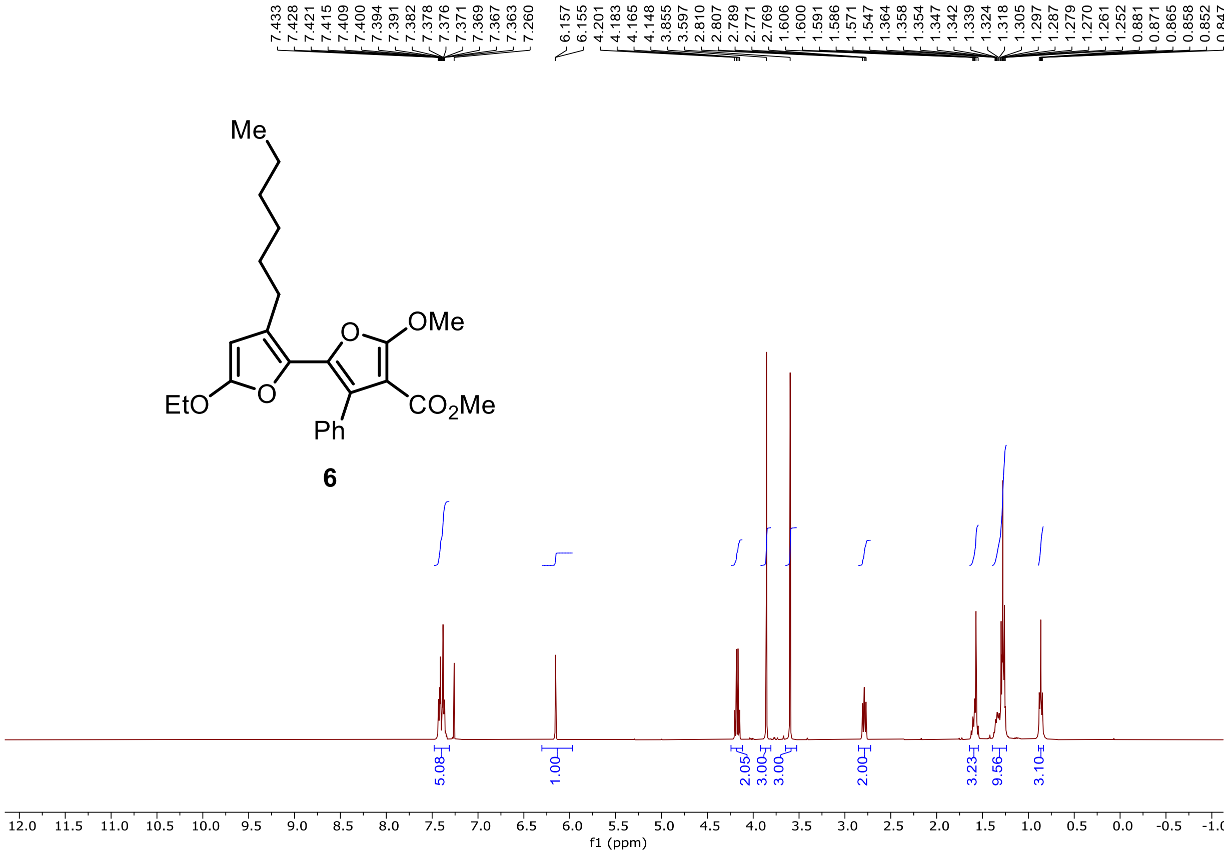

¹³C NMR (101 MHz, $CDCl_3$) of 6

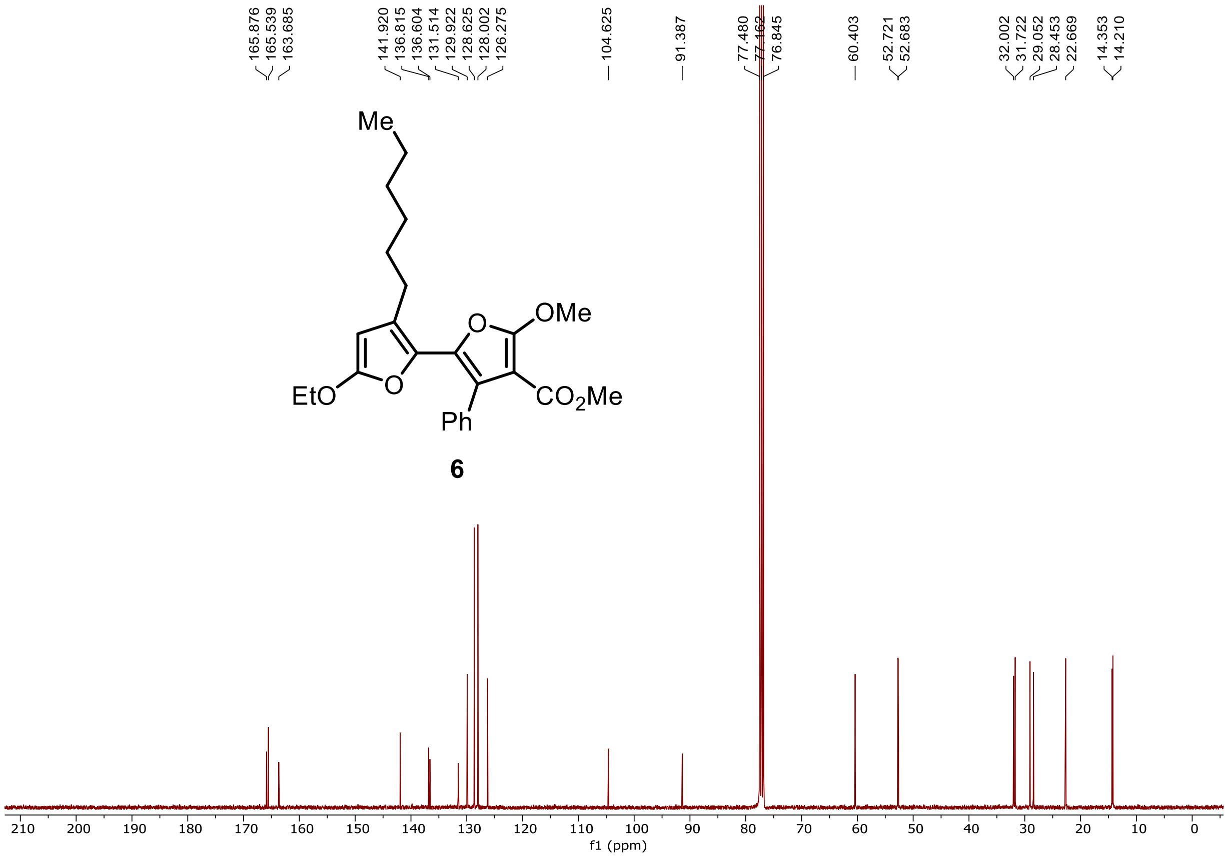

^{1}H NMR (400 MHz, $CDCl_3$) of 7

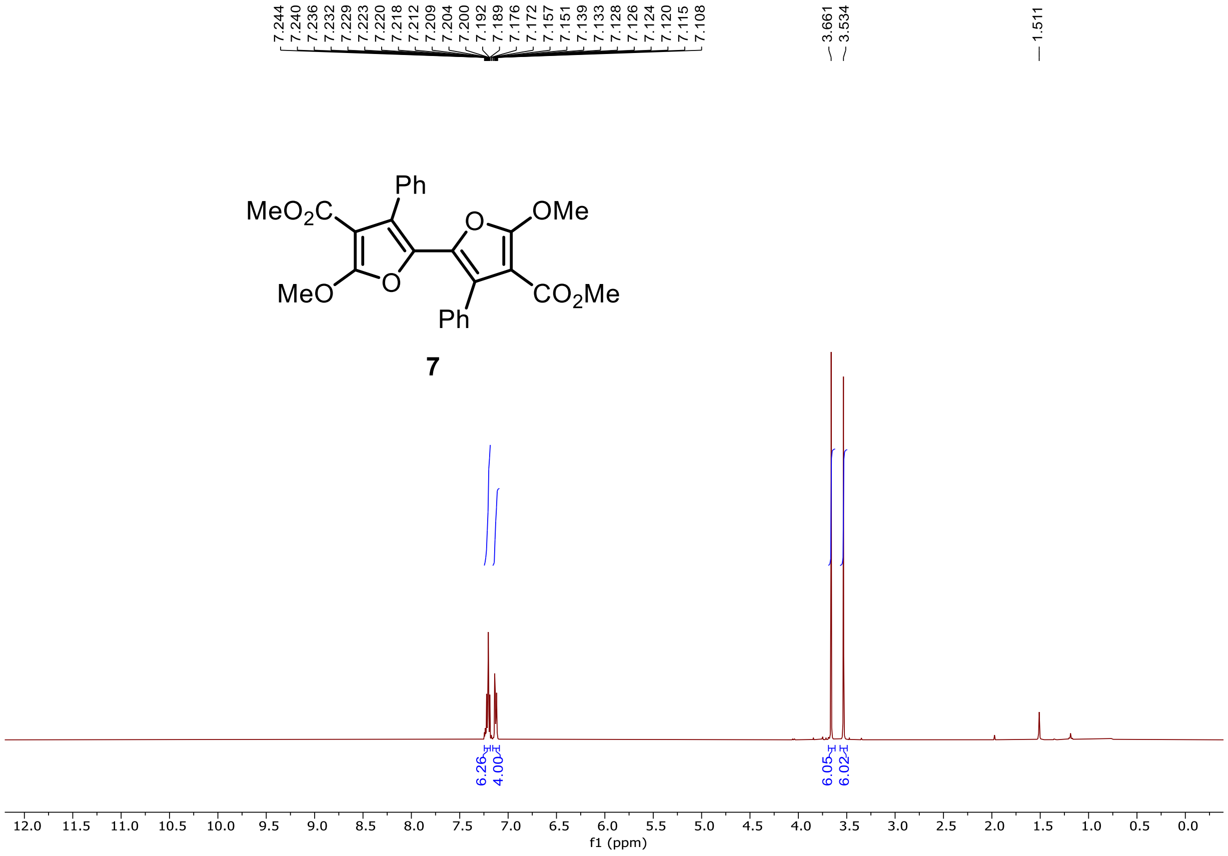

^{13}C NMR (101 MHz, $CDCl_3$) of 7

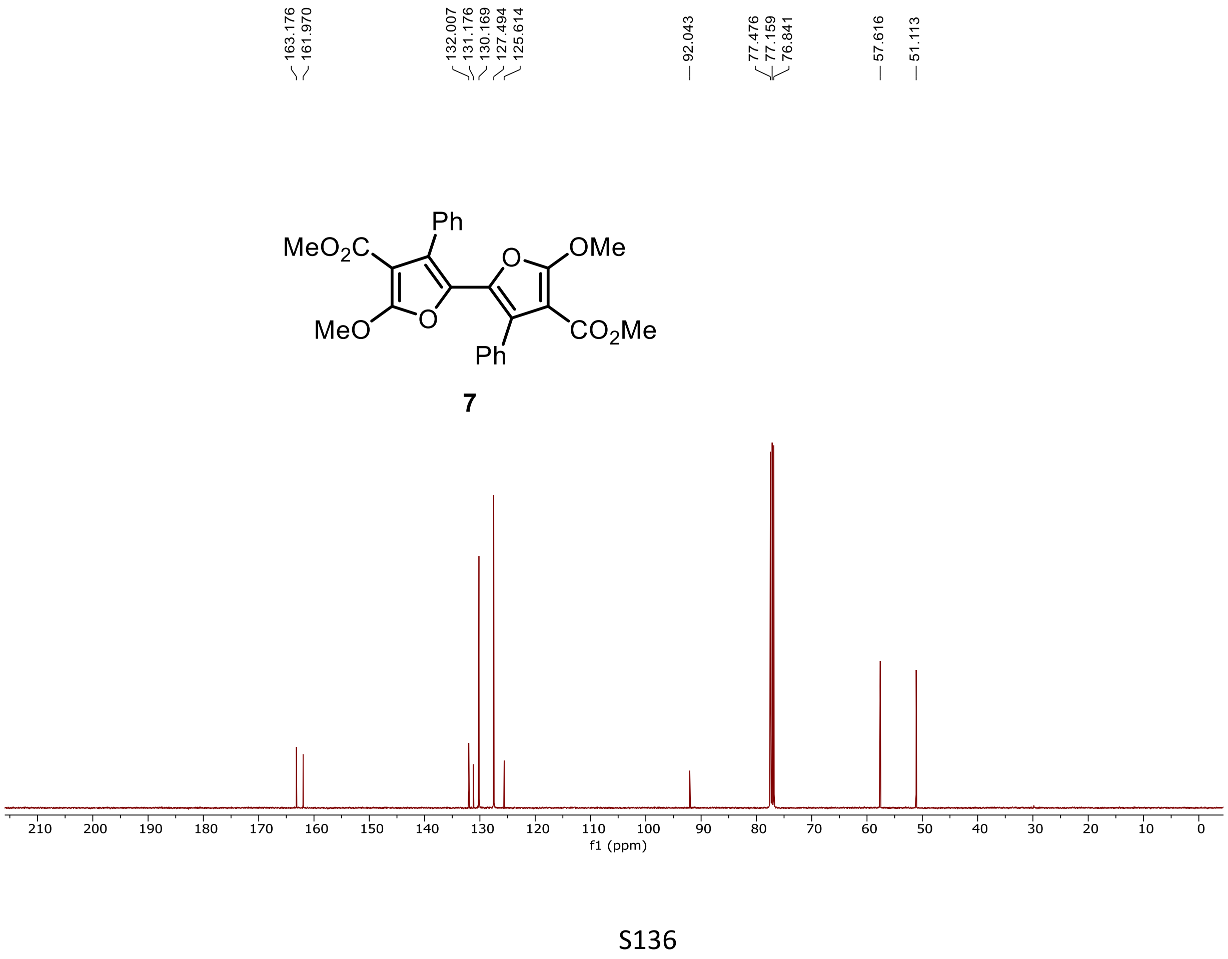

^{1}H NMR (400 MHz, DMSO-d_6) of 8

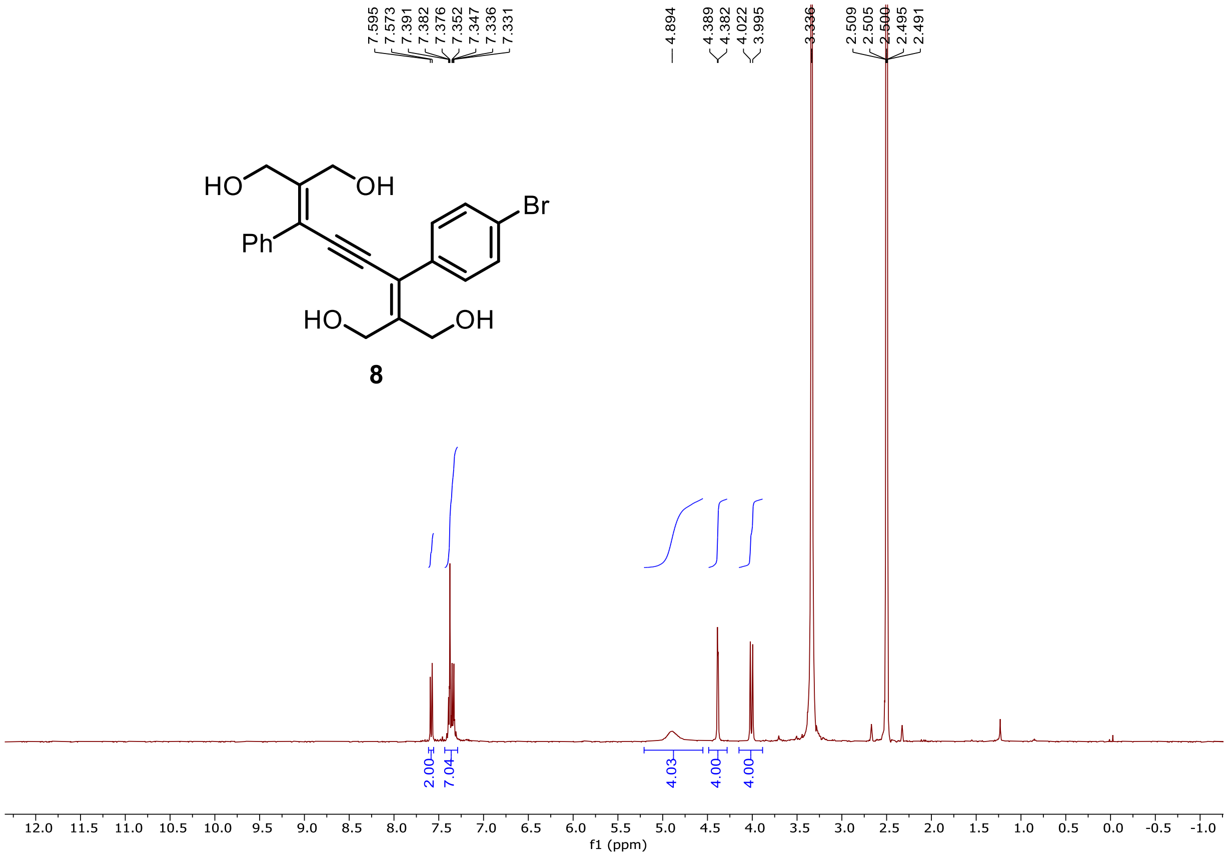

^{13}C NMR (101 MHz, DMSO-d_6) of 8

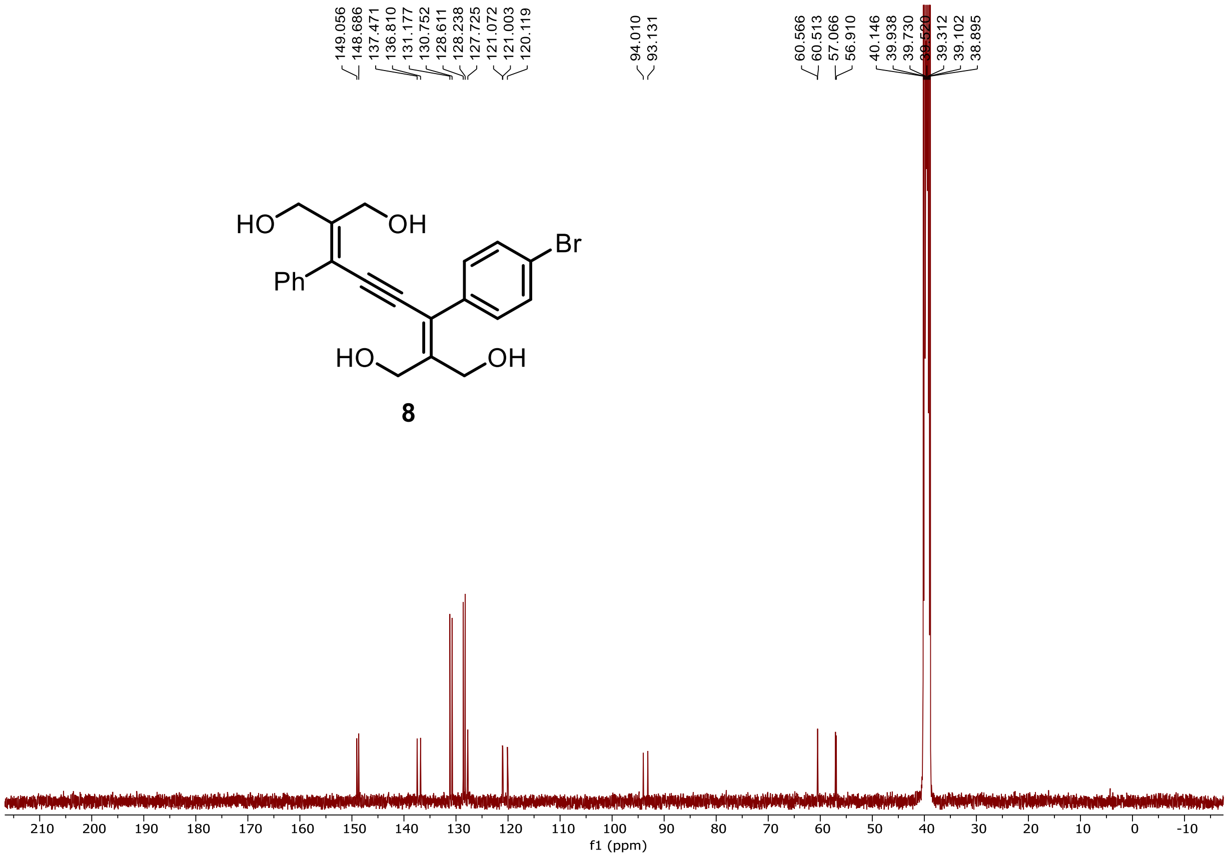

^{1}H NMR (400 MHz, CD_2Cl_2) of 9

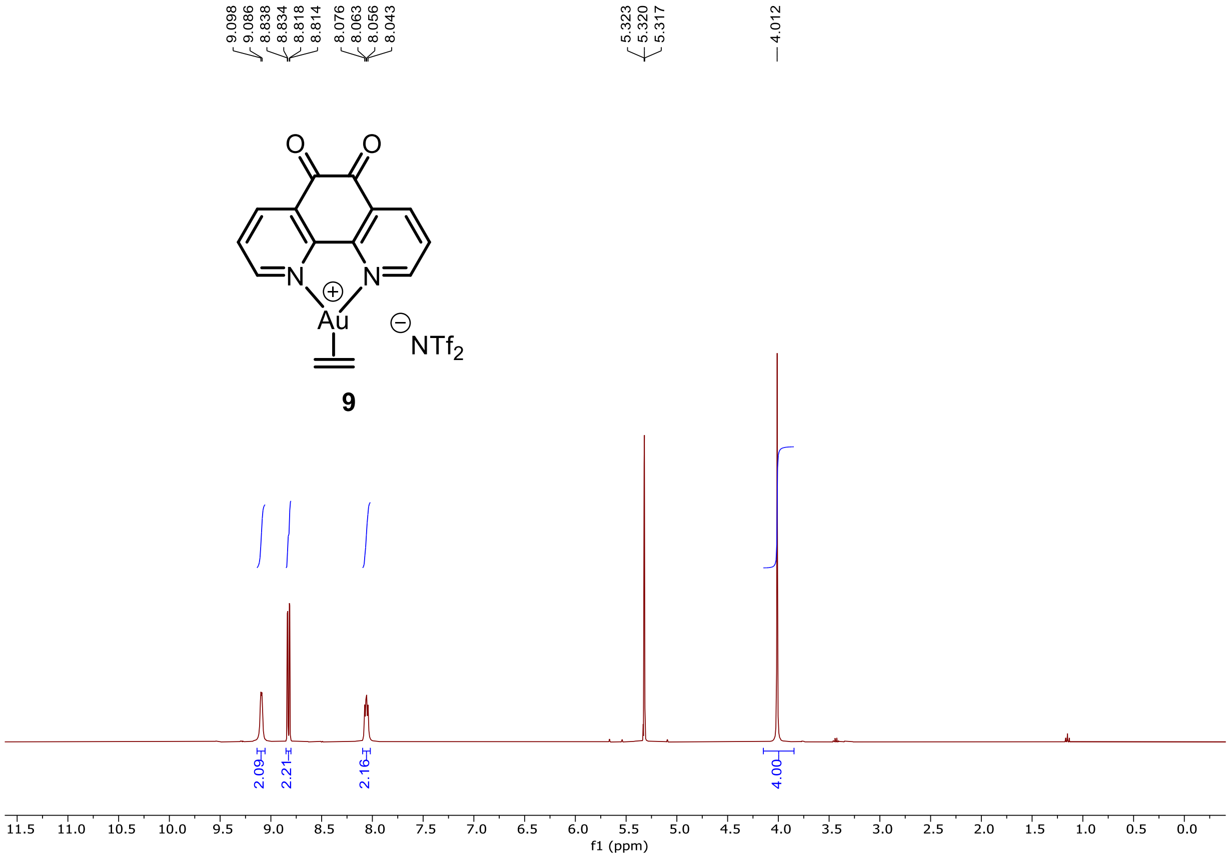

^{13}C NMR (101 MHz, CD_2Cl_2) of 9

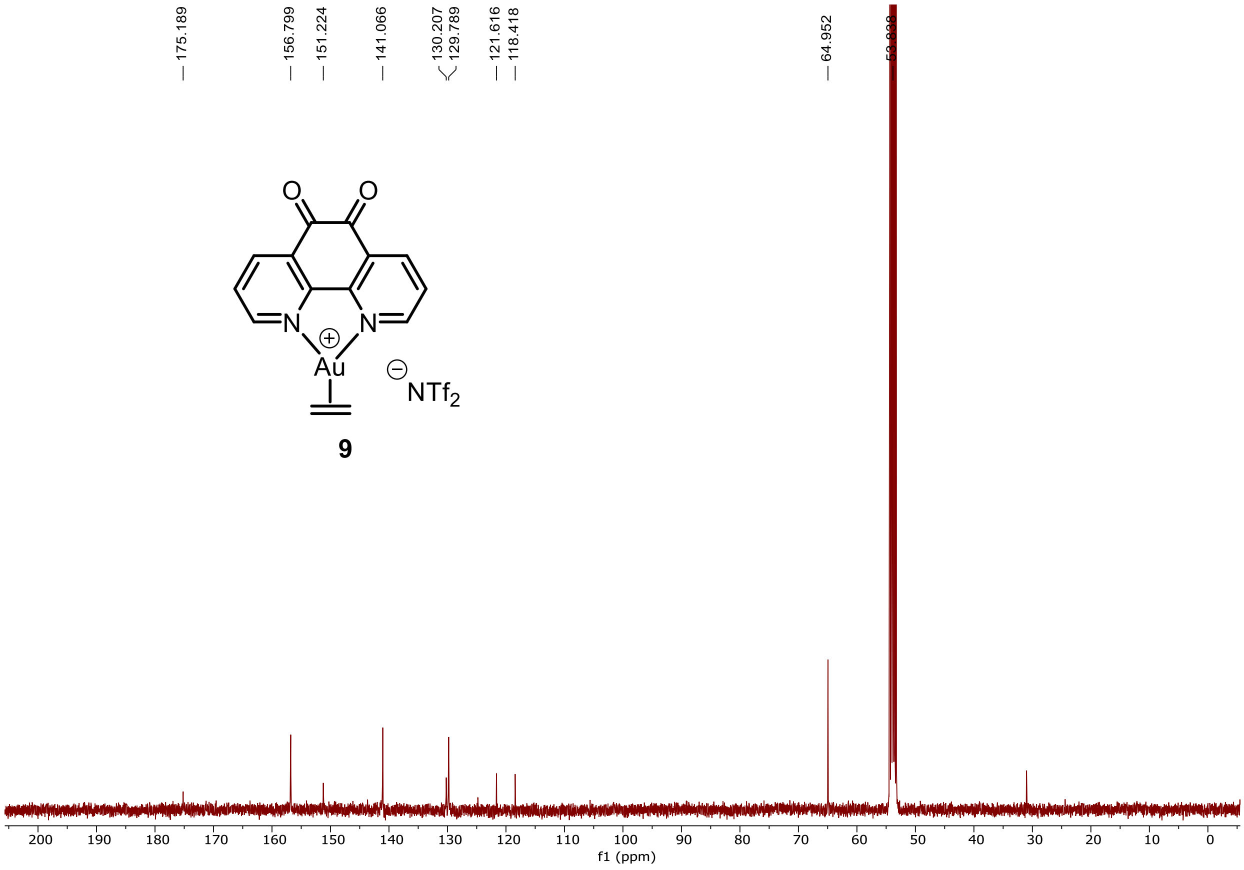

^{19}F NMR (376 MHz, CD_2Cl_2) of 9

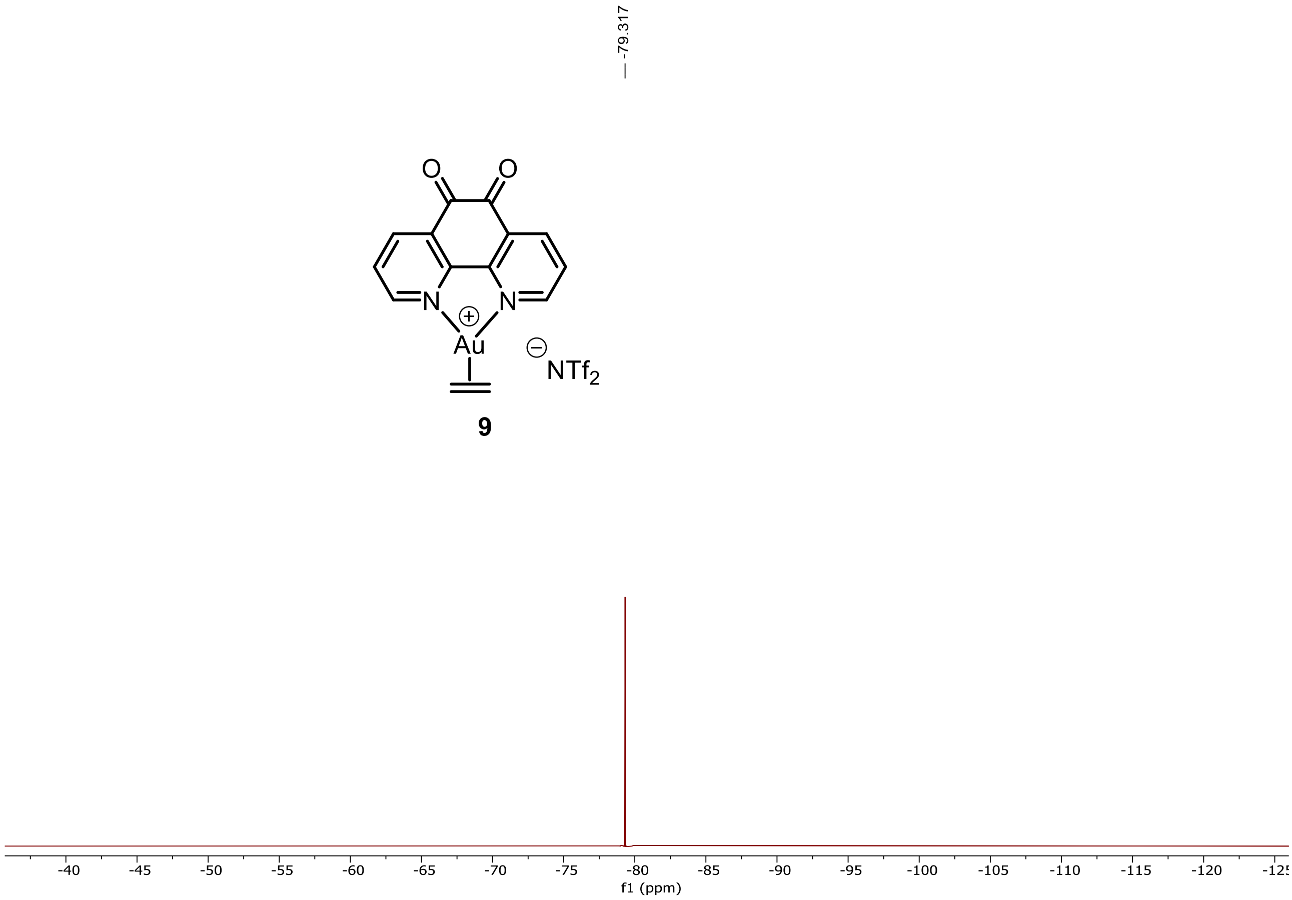

^{1}H NMR (400 MHz, $CDCl_3$) of 1a-1

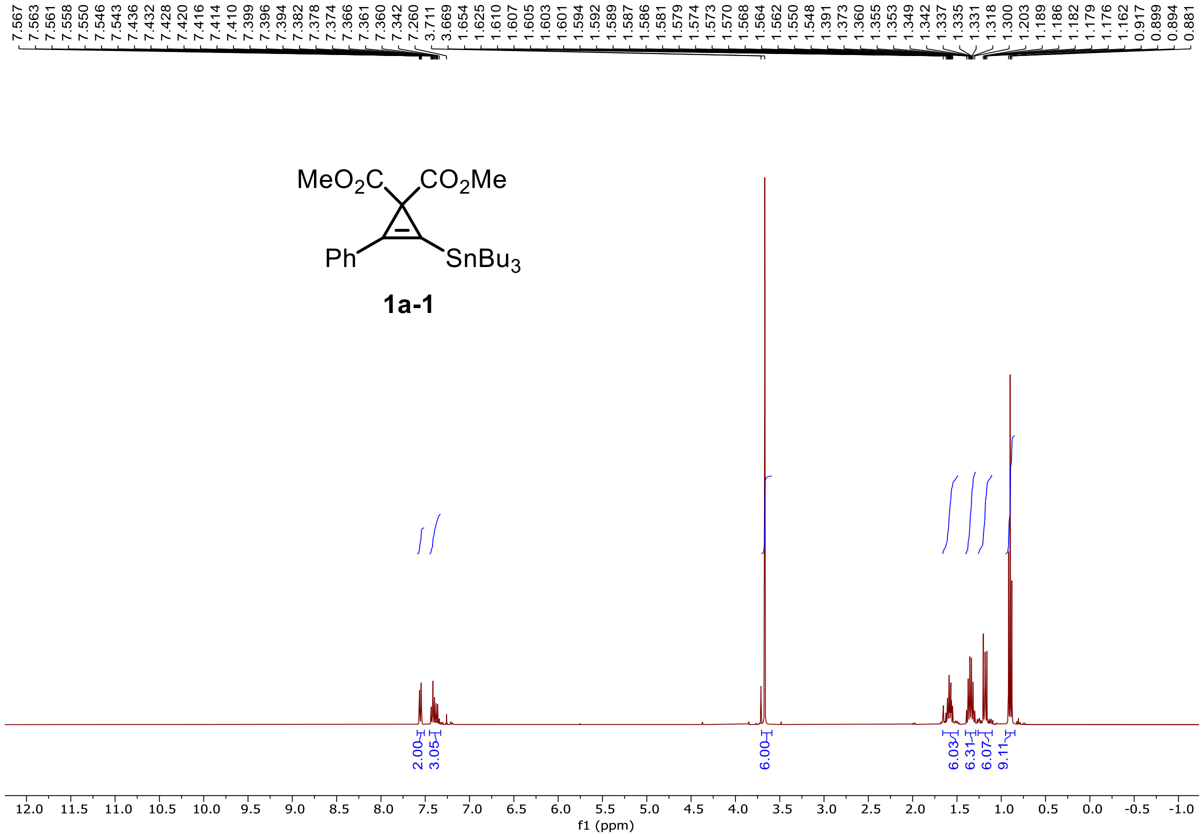

^{13}C NMR (101 MHz, $CDCl_3$) of 1a-1

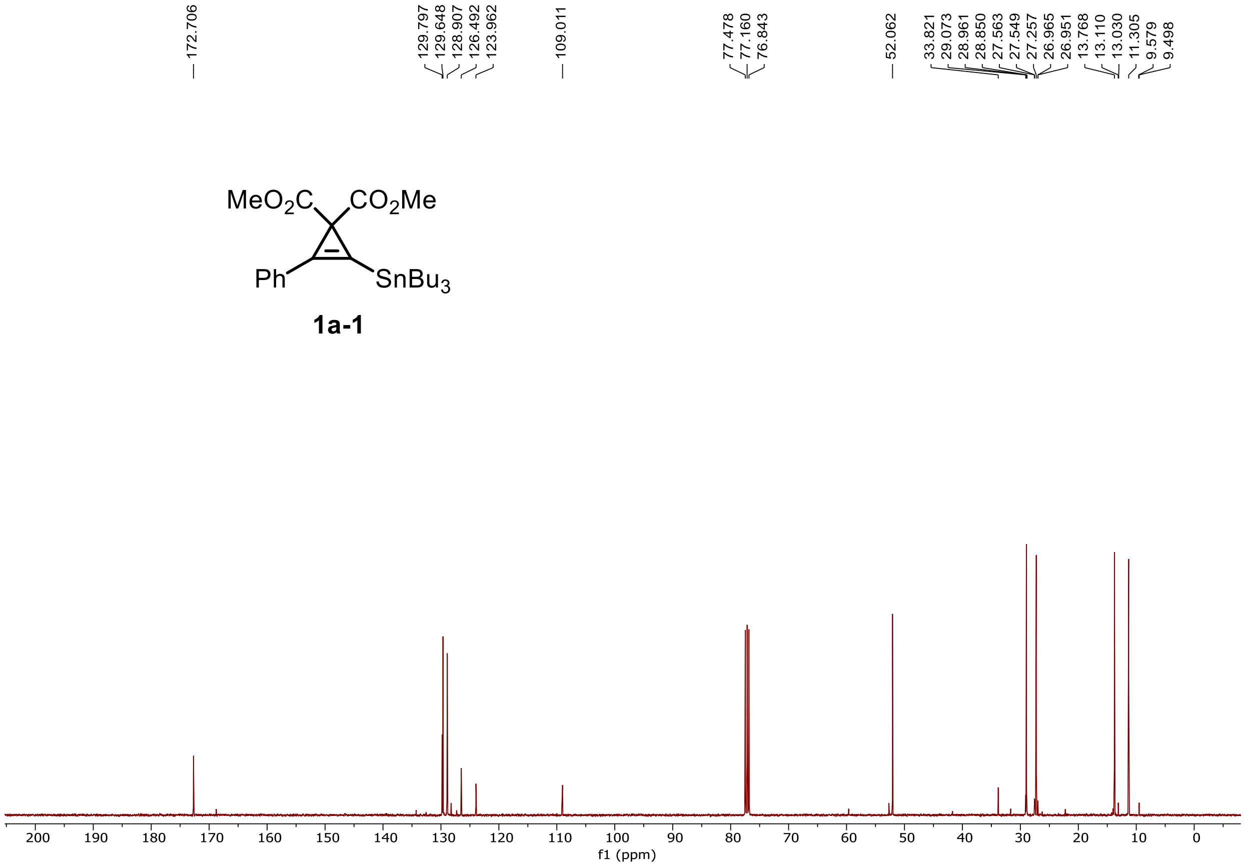

^{1}H NMR (400 MHz, $CDCl_3$) of 1q-1

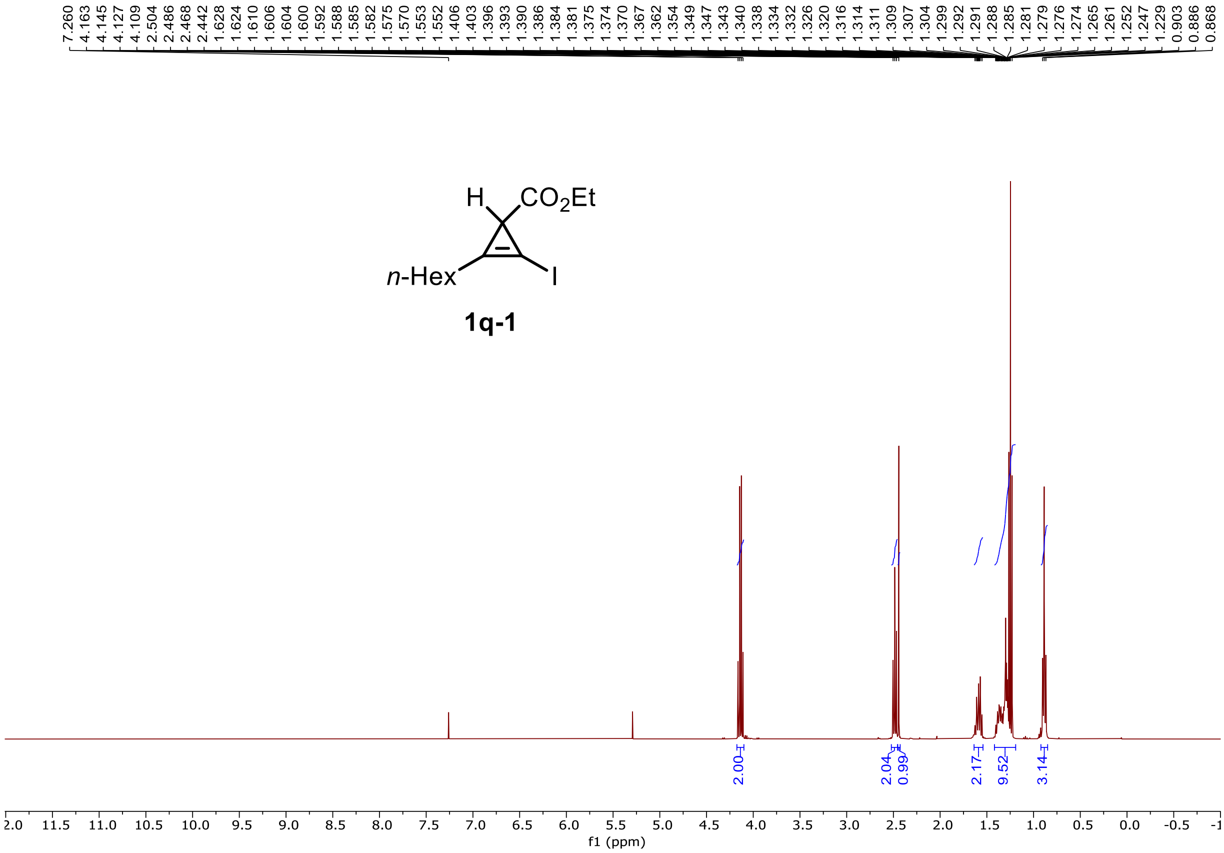

^{13}C NMR (101 MHz, CDCl$_3$) of 1q-1

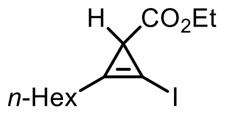

1q-1

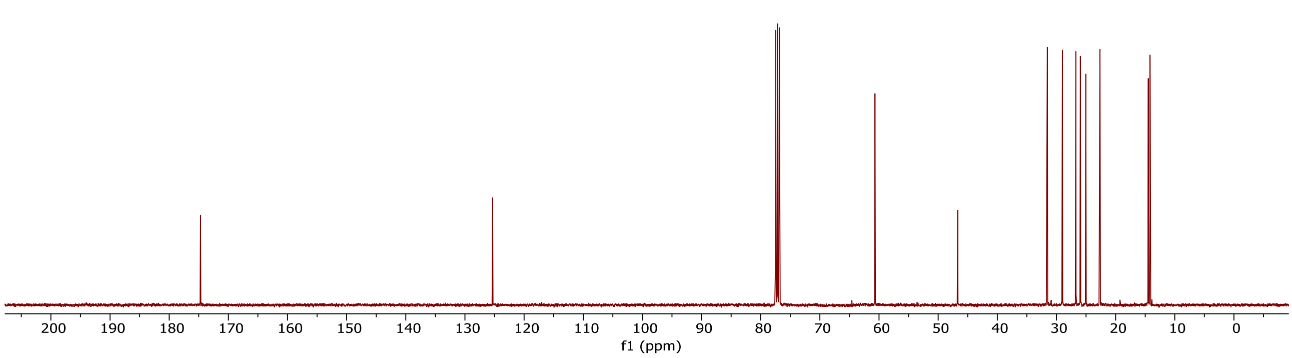

11. References

[1] Song, C.; Ju, L.; Wang, M.; Liu, P.; Zhang, Y.; Wang, J.; Xu, Z. From cyclopropenes to tetrasubstituted furans: tandem isomerization/alkenylation sequence with Cu/Pd relay catalysis. *Chem. - Eur. J.* **2013**, *19*, 3584–3589.
[2] Muriel, B.; Waser, J. Azide Radical Initiated Ring Opening of Cyclopropenes Leading to Alkenyl Nitriles and Polycyclic Aromatic Compounds. *Angew. Chem., Int. Ed.* **2021**, *60*, 4075–4079.
[3] Chuprakov, S.; Rubin, M.; Gevorgyan, V. Direct Palladium-Catalyzed Arylation of Cyclopropenes. *J. Am. Chem. Soc.* **2005**, *127*, 3714–3715.
[4] Ye, Q.; Ye, H.; Cheng, D.; Li, X.; Xu, X. Regioselective oxidative ring-opening of cyclopropenyl carboxylates by visible light photoredox catalysis. *Tetrahedron Lett.* **2018**, *59*, 2546–2549.
[5] Zhang, F.; Fox, J. M. Synthesis of Cyclopropene α-Amino Acids via Enantioselective Desymmetrization. *Org. Lett.* **2006**, *8*, 2965–2968.
[6] Wang, Y.; Fordyce, E. A. F.; Chen, F. Y.; Lam, H. W. Stereoselective Synthesis of Tri- and Tetrasubstituted Alkenes by Iron-Catalyzed Carbometallation Ring-Opening Reactions of Cyclopropenes. *Angew. Chem., Int. Ed.* **2008**, *47*, 7350–7353.
[7] Tarwade, V.; Liu, X.; Yan, N.; Fox, J. M. Directed Carbozincation Reactions of Cyclopropene Derivatives. *J. Am. Chem. Soc.* **2009**, *131*, 5382–5383.
[8] Dalling, A. G.; Yamauchi, T.; McCreanor, N. G.; Cox, L.; Bower, J. F. Carbonylative C−C Bond Activation of Electron-Poor Cyclopropanes: Rhodium-Catalyzed (3+1+2) Cycloadditions of Cyclopropylamides. *Angew. Chem., Int. Ed.* **2019**, *58*, 221–225.
[9] Dange, N. S.; Robert, F.; Landais, Y. Free-Radical Carbocyanation of Cyclopropenes: Stereocontrolled. *Org. Lett.* **2016**, *18*, 6156–6159.
[10] Ueda, M.; Doi, N.; Miyagawa, H.; Sugita, S.; Takeda, N.; Shinada, T.; Miyata, O. Reaction of cyclopropenes with a trichloromethyl radical: unprecedented ring-opening reaction of cyclopropanes with migration. *Chem. Commun.* **2015**, *51*, 4204–4207.
[11] Abu-Elfotoh, A.-M.; Nguyen, D. P. T.; Chanthamath, S.; Phomkeona, K.; Shibatomi, K.; Iwasa, S. Water-Soluble Chiral Ruthenium(II) Phenyloxazoline Complex: Reusable and Highly Enantioselective Catalyst for Intramolecular Cyclopropanation Reactions. *Adv. Synth. Catal.* **2012**, *354*, 3435–3439.
[12] Morandi, B.; Carreira, E. M. Rhodium-Catalyzed Cyclopropenation of Alkynes: Synthesis of Trifluoromethyl-Substituted Cyclopropenes. *Angew. Chem., Int. Ed.* **2010**, *49*, 4294–4296.
[13] Tsuzuki, S.; Sakamoto, R.; Maruoka, K. Practical Synthesis of α,β-Alkynyl Ketones by Oxidative Alkynylation of Aldehydes with Hypervalent Alkynyliodine Reagents. *Chem. Lett.* **2020**, *49*, 633–636.
[14] Maity, A.; Hyun, S.-M.; Powers, D. C. Oxidase Catalysis via Aerobically Generated Hypervalent Iodine Intermediates. *Nat. Chem*. **2018**, *10*, 200–204.
[15] Pisella, G.; Gagnebin, A.; Waser, J. Copper-Catalyzed Oxyvinylation of Diazo Compounds. *Org. Lett.* **2020**, *22*, 3884–3889.
[16] Zhang, Z.-Q.; Zheng, M.-M.; Xue, X.-S.; Marek, I.; Zhang, F.-G.; Ma, J.-A. Catalytic Enantioselective Cyclopropenation of Internal Alkynes: Access to Difluoromethylated Three-Membered Carbocycles. *Angew. Chem., Int. Ed.* **2019**, *58*, 18191–18196.
[17] Tu, H.-F.; Jeandin, A.; Bon, C.; Brocklehurst, C.; Lima, F.; Suero, M. G. Late-Stage Aryl C-H Bond Cyclopropenylation with Cyclopropenium Cations. *Angew. Chem., Int. Ed.* **2023**, *62*, No. e202308379.
[18] Song, C.; Sun, D.; Peng, X.; Bai, J.; Zhang, R.; Hou, S.; Wang, J.; Xu, Z. Dimerization of Cyclopropenes to Bifurans Using Tandem Metal Relay Catalysis. *Chem. Commun.* **2013**, *49*, 9167–9169.
[19] Li, C.; Zhang, H.; Feng, J.; Zhang, Y.; Wang, J. Rh(I)-Catalyzed Carbonylative Carbocyclization of Tethered Ene– and Yne–cyclopropenes. *Org. Lett*. **2010**, *12*, 3082–3085.
[20] Harper, M. J., Arthur, C. J., Crosby, J., Emmett, E. J., Falconer, R. A., Fensham-Smith, A. J., Gates, P. G., Leman, T., McGrady, J. E., Bower, J. F. & Russell, C. A. Oxidative addition, transmetalation, and reductive elimination at a 2,2-bipyridyl-ligated gold center. *J. Am. Chem. Soc.* **2018**, *140*, 4440–4445.
[21] Dias, H. V. R., Fianchini, M., Cundari, T. R. & Campana, C. F. Synthesis and characterization of the gold(I) tris(ethylene) complex $[Au(C_2H_4)_3][SbF_6]$. *Angew. Chem. Int. Ed*. **2008**, *47*, 556–559.
[22] Fordyce, E. A. F.; Luebbers, T.; Lam, H. W. Synthesis and Application of Alkenylstannanes Derived from Base-Sensitive Cyclopropenes. *Org. Lett*. **2008**, *10*, 3993–3996.
[23] Li, X.; Wodrich, M. D.; Waser, J. Accessing Elusive σ-Type Cyclopropenium Cation Equivalents through Redox Gold Catalysis. *Nat. Chem.* **2024**, *16*, 901–912.
